# Bicyclo[1.1.0]butyl Radical Cations: Synthesis and Application to [2π + 2σ] Cycloaddition Reactions

**DOI:** 10.1021/jacs.4c04403

**Published:** 2024-05-29

**Authors:** Jasper L. Tyler, Felix Schäfer, Huiling Shao, Colin Stein, Audrey Wong, Constantin G. Daniliuc, K. N. Houk, Frank Glorius

**Affiliations:** † Organisch-Chemisches Institut, Universität Münster, 48149 Münster, Germany; ‡ Department of Chemistry and Biochemistry, University of California, Los Angeles, California 90095-1569, United States

## Abstract

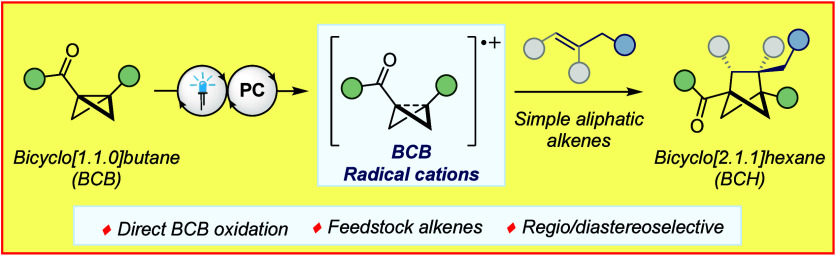

As the chemistry that surrounds the field of strained hydrocarbons, such as bicyclo[1.1.0]butane, continues to expand, it becomes increasingly advantageous to develop alternative reactivity modes that harness their unique properties to access new regions of chemical space. Herein, we report the use of photoredox catalysis to promote the single-electron oxidation of bicyclo[1.1.0]butanes. The synthetic utility of the resulting radical cations is highlighted by their ability to undergo highly regio- and diastereoselective [2π + 2σ] cycloaddition reactions. The most notable feature of this transformation is the breadth of alkene classes that can be employed, including nonactivated alkenes, which have so far been elusive for previous strategies. A rigorous mechanistic investigation, in conjunction with DFT computation, was undertaken in order to better understand the physical nature of bicyclo[1.1.0]butyl radical cations and thus provides a platform from which further studies into the synthetic applications of these intermediates can be built upon.

## Introduction

**Figure 1:**
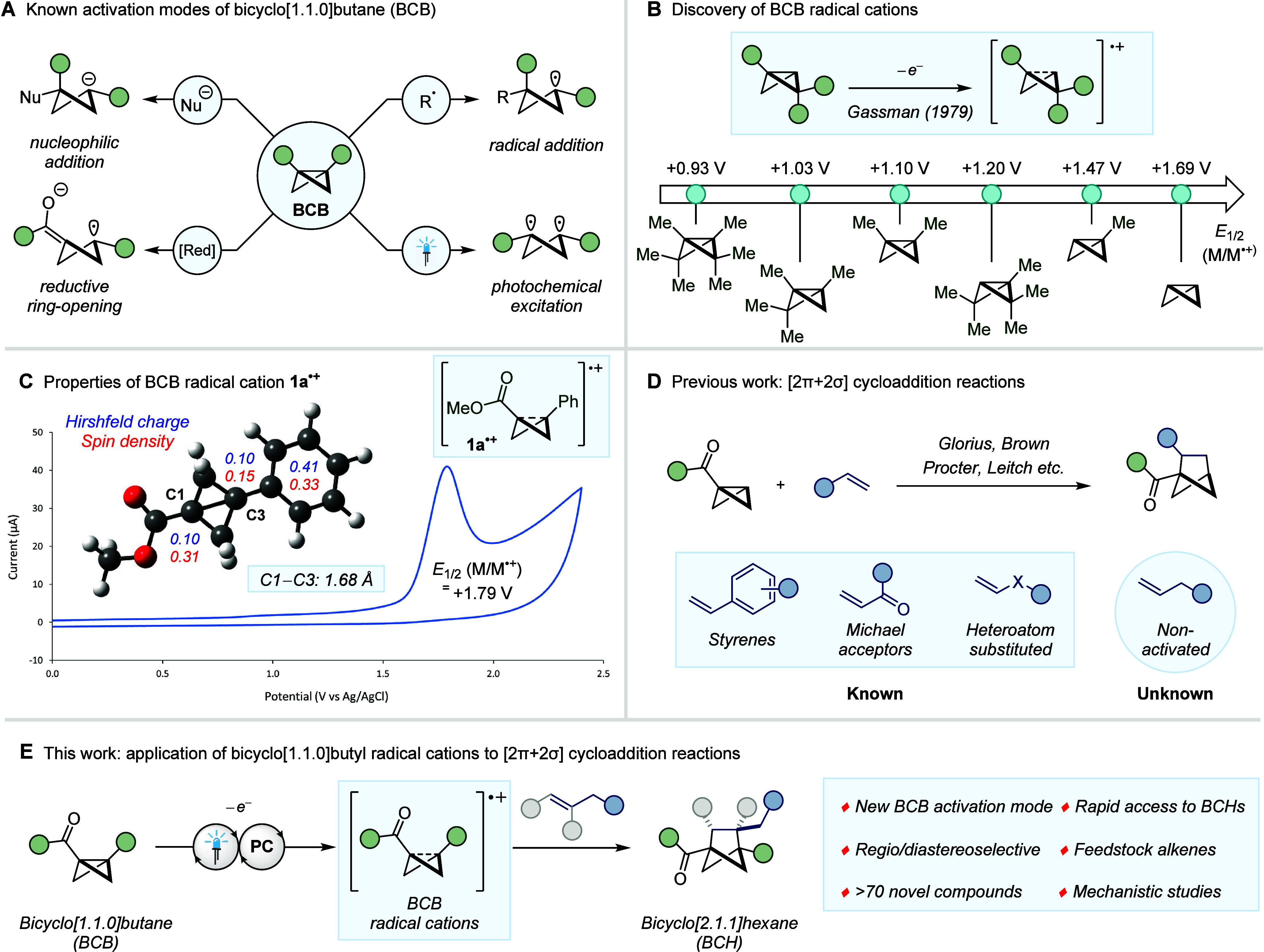
(A) Known activation modes of bicyclo[1.1.0]butane. (B) Discovery of the direct oxidation of substituted bicyclo[1.1.0]butane compounds.^27^ (C) Oxidation potential of BCB **1a** vs Ag/AgCl (2 M LiCl in EtOH) and physical properties of bicyclo[1.1.0]butyl radical cation **1a**^**•+**^. (D) Previous reports of [2π + 2σ] cycloaddition reactions. (E) This work: application of bicyclo[1.1.0]butyl radical cations to [2π + 2σ] cycloaddition reactions.

Since its first synthesis in 1959,^1^ bicyclo[1.1.0]butane (BCB) has captured the imagination of chemists due to its innate strain energy and relative ease of assembly and handling.^2,3^ The highly diverse reactivity of BCB-containing compounds, facilitated by the release of strain upon breaking the bridging C1–C3 bond, has allowed such structures to become valuable building blocks for the generation of sp^3^-rich carbocycles and heterocycles.^2−5^ Perhaps the most prominent reactivity mode that has been utilized in this context is the addition of nucleophiles and nucleophilic radicals to the bridgehead of electron-deficient BCB compounds (Figure 1A).^6−9^ In recent years, alternative strategies have also emerged, such as electrophilic addition,^10−12^ reduction^13,14^ or Lewis acid activation^15−18^ of adjacent carbonyl fragments to trigger ring-opening, pyridine-boryl radical transfer^19,20^ and photochemical excitation of the strained bridging bond to access the corresponding diradical.^21,22^ Employing these strategies has led to the development of many unique transformations, and as a consequence, BCB-containing compounds have become cemented as valuable synthetic building blocks. This is most clearly evidenced by their application in areas such as bioconjugation^23,24^ and in the assembly of rigid and sp^3^-rich arene isosteres, structures of particular interest in a medicinal chemistry context.^10,11,25^ However, in order to find new applications and access unexplored regions of chemical space, alternative reactivity modes that harness the unique properties of BCBs are required.

In 1979, Gassman reported a study on the relationship between alkyl substitution and the ease of oxidation of strained hydrocarbons.^26^ By measuring the half-wave potentials of a variety of substituted bicyclo[1.1.0]butanes, it was demonstrated that the σ-framework of the bicycle could readily undergo single-electron oxidation to the corresponding radical cation (Figure 1B).^27^ In addition, Hoz and co-workers postulated the existence of a radical cation intermediate, arising from single-electron oxidation, in the bromination of bicyclo[1.1.0]butane analogs.^28^ Despite these discoveries, the synthetic potential of bicyclo[1.1.0]butyl radical cations has been severely underexplored,^29^ with nonselective nucleophilic addition being the only transformation demonstrated for these intermediates.^30−32^ We therefore determined to assess the feasibility of employing single-electron oxidation via photoredox catalysis as a strategy to access alternative bicyclo[1.1.0]butane reactivity.

## Results and Discussion

To avoid the issues associated with handling low-molecular-weight strained hydrocarbons, electron-deficient BCB **1a**, known to be nonvolatile and easily synthesized, was investigated (Figure 1C). Although this species contains an electron-withdrawing group directly appended to the σ-framework, cyclic voltammetry studies clearly showed that this compound could be oxidized, with a half-wave potential of +1.79 V vs Ag/AgCl (2 M LiCl in EtOH). Density functional theory (DFT) calculations of the condensed Hirshfeld charges and spin densities of the radical cation revealed that both the overall charge and the spin were largely delocalized across the bridging bond of the BCB framework as well as the aromatic ring (Figure 1C). Analyzing these values revealed that the C1 and C3 carbon atoms have a greater contribution of the overall spin (0.31 and 0.15, respectively) compared to the positive charge (0.10 and 0.10, respectively), which is more concentrated on the aryl ring (0.41). Additionally, the bridging bond remains intact upon oxidation and shows an elongation of just 0.16 Å compared to the ground state,^33^ highlighting the difference between this activation strategy and energy transfer, where σ-bond cleavage occurs to form the corresponding diradical.^21^

With an understanding of the accessibility and physical properties of BCB radical cations, a reactivity regime that cannot be achieved using previously known strategies was pursued. Specifically, we targeted [2π + 2σ] cycloaddition reactions to access bicyclo[2.1.1]hexane (BCH) compounds, highly valuable isosteres of *ortho*- and *meta*-substituted benzene that are sp^3^-rich with well-defined substituent exit vectors.^34−37^ Although [2π + 2σ] cycloaddition reactions that harness the strained bond of BCB have been reported by our own research group,^18,38−40^ as well as those of Brown,^21^ Procter,^13^ Leitch^25^ and others,^14−17,19,20,41^ in all cases, alkenes that can be employed require a radical stabilizing group, electron-withdrawing group, or heteroatom directly appended to the double bond, depending on the respective mechanism (Figure 1D). Conversely, it is known that styrene-type radical cations,^42−44^ intermediates that display remarkably similar levels of charge and spin delocalization to **1a**^**•+**^ (see Supporting Information for details), have the ability to participate in [2π + 2π] cycloaddition reactions with olefins that are not stabilized by an adjacent π-system or carbonyl unit.^45−47^ Therefore, we believed that harnessing BCB radical cations could provide a general method for [2π + 2σ] cycloaddition reactions, allowing the transformation to occur with multiple distinct classes of olefins, including simple feedstock alkenes that have so far been elusive.

Herein, we report the successful application of bicyclo[1.1.0]butyl radical cations to [2π + 2σ] cycloaddition reactions to generate a unique selection of BCH structures (Figure 1E). The most notable features of this strategy are the breadth of alkene classes that can be employed and the remarkable levels of regio- and diastereoselectivity that can be achieved using single-electron oxidation as the activation mode for BCB. A rigorous experiment-based mechanistic study, in conjunction with DFT computation, was undertaken in order to better understand this process and illuminate how these strained radical cations interact with alkenes. Consequently, we believe that the work described here can serve as a platform from which further studies into the potential synthetic applications of BCB radical cations can be built upon.

## Reaction Development

**Figure 2:**
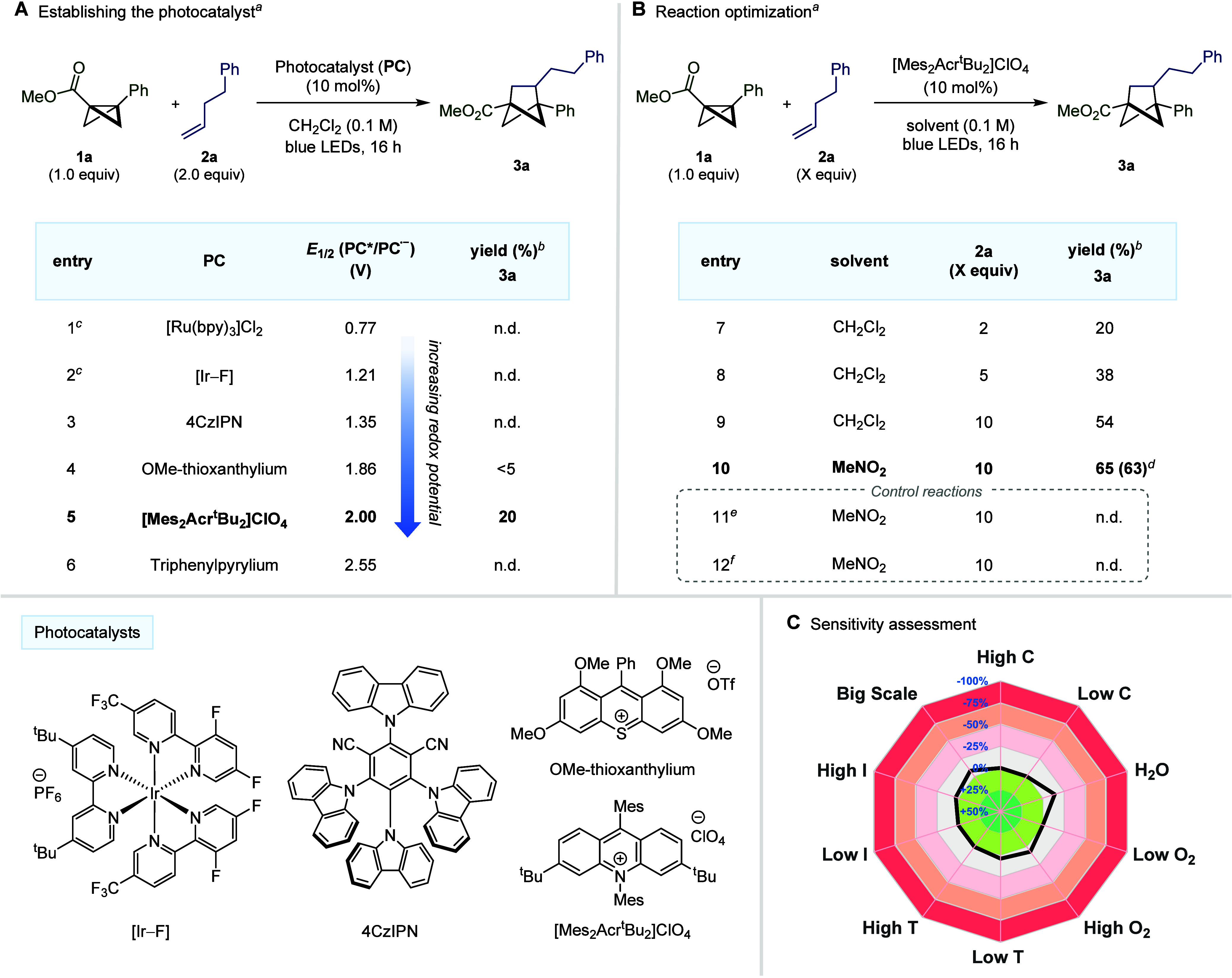
Optimization of the BCB radical cation [2π + 2σ] cycloaddition reaction. (A) Establishing the photocatalyst. (B) Optimization of the reaction conditions. (C) Sensitivity assessment of the reaction conditions. ^*a*^Reactions performed on 0.05 mmol scale under blue LED (λ_max_ = 425 nm) irradiation. ^*b*^Yields determined by ^1^H NMR analysis of the crude reaction mixture using CH_2_Br_2_ as an internal standard. ^*c*^2 mol % of photocatalyst. ^*d*^Isolated yield on 0.2 mmol scale. ^*e*^No photocatalyst. ^*f*^Reaction performed in absence of light.

Our investigation commenced with the exploration of suitable photocatalysts, capable of promoting the single-electron oxidation of the BCB moiety. Olefin **2a**, containing no adjacent heteroatom or radical stabilizing group, was selected as the model coupling partner due to its presumed inactivity under any currently known BCB [2π + 2σ] cycloaddition conditions. Upon screening photocatalysts across a wide range of oxidation potentials, it was demonstrated that [Mes_2_Acr^t^Bu_2_]ClO_4_ (*E*_1/2_ (PC*/PC^**•**–^) = +2.00 V vs SCE),^48^ irradiated with blue LEDs, could catalyze the desired [2π + 2σ] cycloaddition reaction with **2a** (Figure 2A, entry 5).^49−51^ Indeed, it was observed that employing photocatalysts that display an excited state oxidation potential below +1.86 V (or significantly greater than +2.00 V) failed to deliver any observable product (entries 1–6). After an extensive exploration of the reaction conditions (see Supporting Information for full optimization details), it was found that improvements to the yield could be achieved upon increasing the equivalents of the alkene coupling partner and performing the reaction in MeNO_2_ (Figure 2B, entries 7–10). Although this reaction represents the first example of a bicyclo[1.1.0]butane [2π + 2σ] cycloaddition with a simple alkyl substituted alkene, the overall yield is partially limited by the side reactions that can occur from the BCB radical cation, such as dimerization.^31,32^ Finally, control reactions, in which the photocatalyst and light source were omitted, were performed (entries 11–12). The inability to access any cycloaddition product under these conditions clearly shows that product formation is dependent on the generation of the excited state photocatalyst and does not arise as a result of direct excitation of either the BCB or alkene substrates.

In order to assess the robustness and reproducibility of the newly established protocol, a reaction condition-based sensitivity assessment was performed (Figure 2C).^52^ Interestingly, the reaction was shown to be remarkably tolerable toward perturbations in the temperature (*T*), concentration (*c*), oxygen level, and light intensity (*I*), with only a slight decrease in yield observed when H_2_O was added to the reaction mixture. Additionally, the photocatalyzed reaction could be performed on 4.0 mmol scale, showing a relatively small erosion in isolated yield compared to the standard reaction (53% vs 63%).

## Reaction Scope

**Figure 3:**
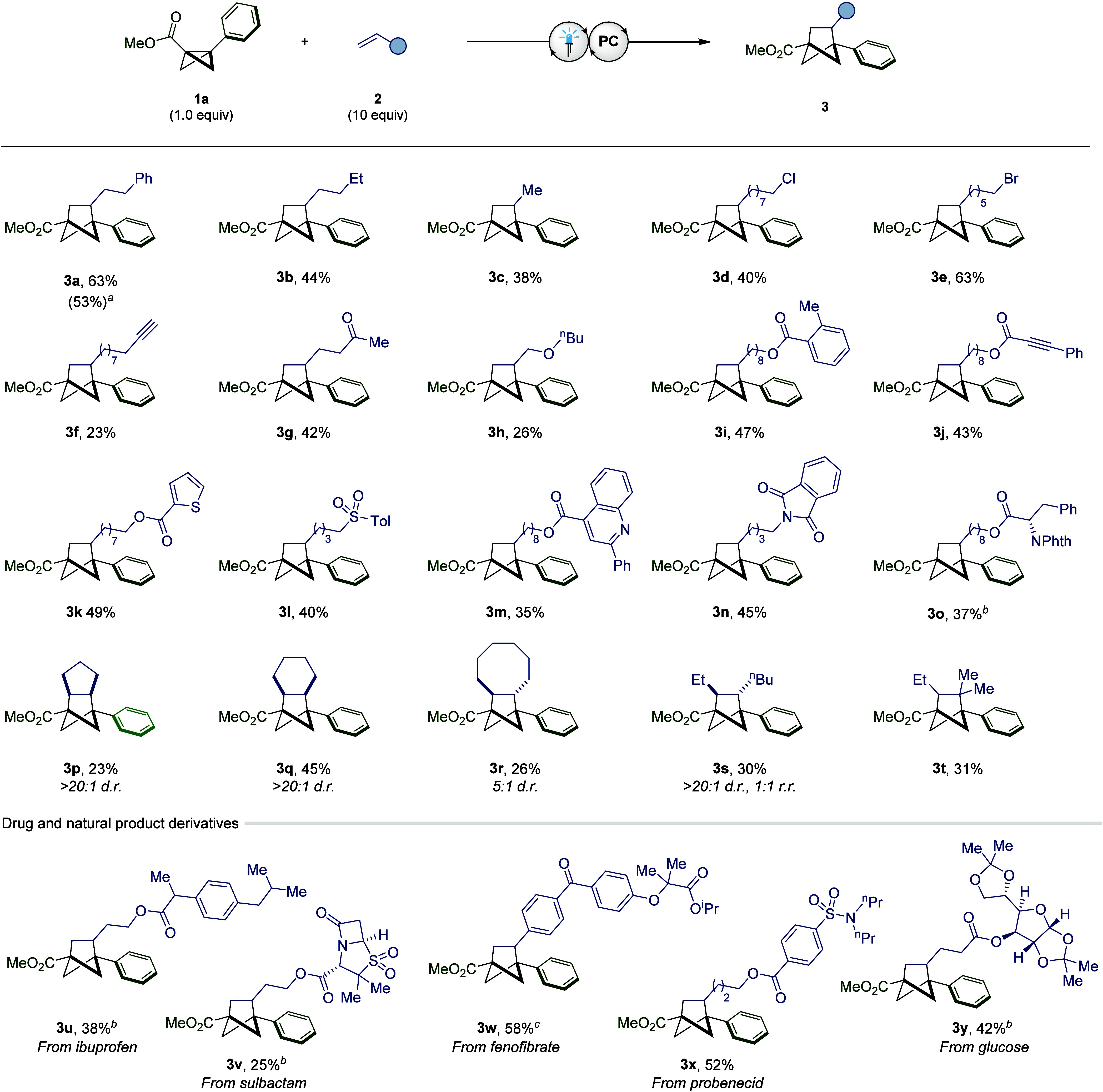
Bicyclo[1.1.0]butane [2π + 2σ] cycloaddition reaction with nonactivated alkenes. Reaction conditions: **1a** (0.2 mmol), **2** (2.0 mmol), [Mes_2_Acr^t^Bu_2_]ClO_4_ (10 mol %), MeNO_2_ (0.1 M), blue LEDs (λ_max_ = 425 nm), 16 h. Isolated yields given. The *d.r.* and *r.r.* values were determined by ^1^H NMR analysis of the crude reaction mixture. ^*a*^Reaction performed on 4.0 mmol of **1a**. ^*b*^Substrates that contain a pre-existing stereocenter were formed as a 1:1 mix of diastereomers. ^*c*^Using conditions from Figure 4 (see below).

With the optimized reaction conditions in hand, the scope of the reaction with respect to the olefin was systematically investigated to both assess the generality of the transformation and discover the limits of reactivity (Figure 3). As well as the simple hydrocarbon 1-hexene (**3b**), propene gas could also be employed under the same reaction conditions to access **3c** in 38% yield. Additionally, functional groups such as primary halides (**3d**–**e**), terminal alkynes (**3f**), ketones (**3g**), ethers (**3h**), esters (**3i**), internal alkynes (**3j**), thiophenes (**3k**) sulfones (**3l**), quinolines (**3m**), phthalimides (**3n**), and amino acid derivatives (**3o**) were all compatible with the transformation and provided a single regioisomer of the desired products. However, limitations to the reaction were discovered when it was observed that some nucleophilic fragments such as unprotected alcohols and amines could not be tolerated in the alkene fragment (see Supporting Information for all failed substrates). Exploring the scope of the alkene substitution pattern demonstrated that internal alkenes such as cyclopentene and cyclohexene could be used to access BCH structures **3p** and **3q** exclusively as the *cis*-diastereomer. However, increasing the ring size to cyclooctene resulted in isomerization to the *trans*-isomer (**3r**). Pleasingly, noncyclic 1,2-disubstituted alkenes such as (*E*)-oct-3-ene were also compatible, providing **3s** in 30% yield as a single diastereomer. Unsurprisingly, with no significant electronic or steric bias, a mix of regioisomers was observed for this substrate. On the other hand, when trisubstituted alkene 2-methylpent-2-ene was utilized, only a single regioisomer (**3t**) was detected in the reaction mixture, demonstrating the ability of the system to clearly distinguish between mono- and disubstituted sp^2^ carbon atoms. Given that bicyclo[2.1.1]hexane structures are seen as potential sp^3^-rich isosteres for *ortho*- and *meta*-substituted benzene, we next investigated the tolerance of natural product and approved pharmaceutical derived alkenes in the newly developed [2π + 2σ] cycloaddition reaction. Promisingly, substrates derived from the anti-inflammatory drug ibuprofen (**3u**) and the β-lactamase inhibitor sulbactam (**3v**) could be tolerated. In addition, derivatives of the cholesterol lowering pharmaceutical fenofibrate (**3w**), the gout medication probenecid (**3x**), and a protected glucose analogue (**3y**) were all capable of accessing the desired BCH products. As complex alkene substrates could be deemed more precious than the BCB coupling partner, we also demonstrated that inverting the stoichiometry of this reaction to have the olefin as the limiting reagent could also provide access to the desired products in comparable yields (see Supporting Information for details).

**Figure 4:**
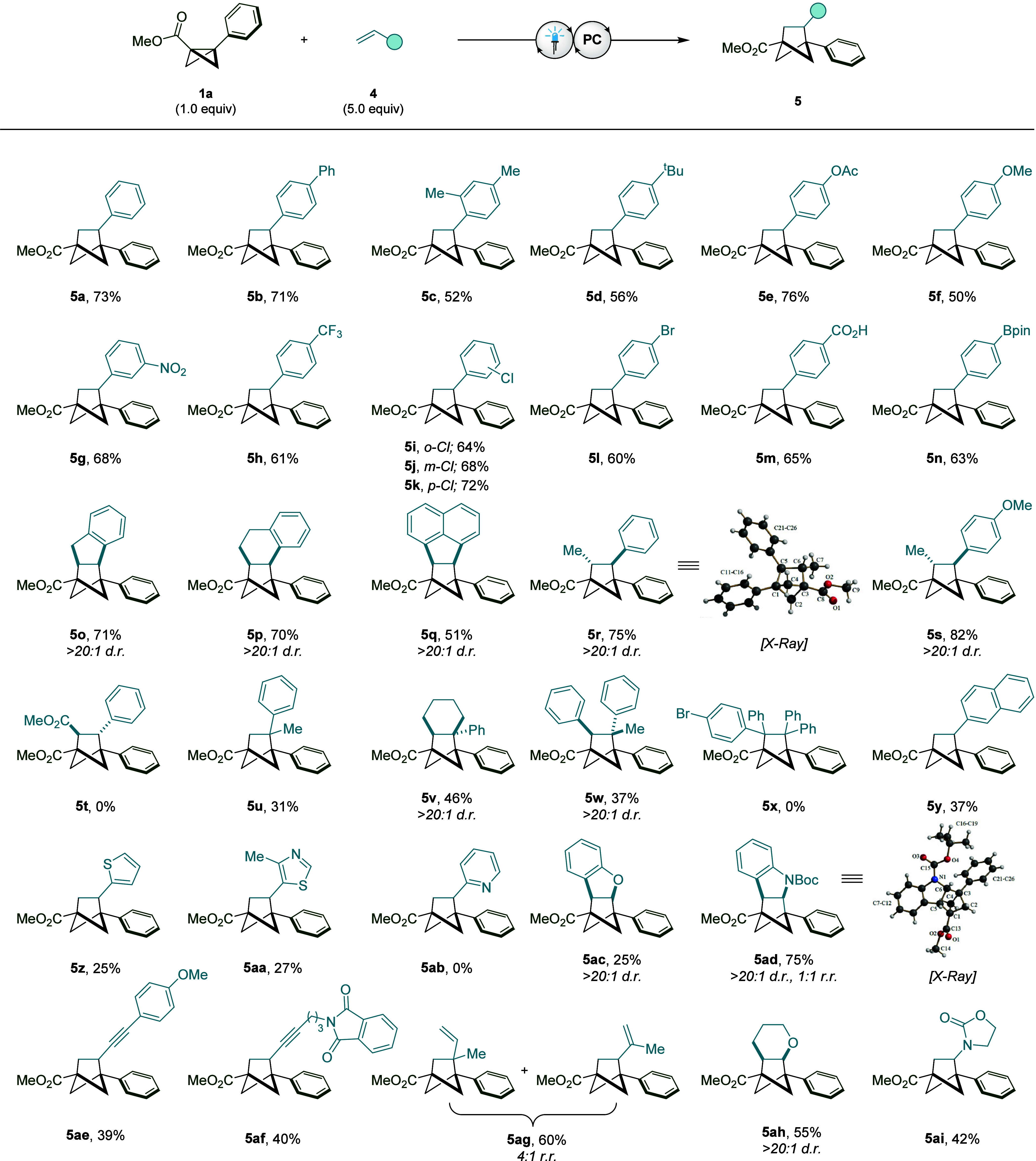
Bicyclo[1.1.0]butane [2π + 2σ] cycloaddition reaction with activated alkenes. Reaction conditions: **1a** (0.2 mmol), **4** (1.0 mmol), [Mes_2_Acr^t^Bu_2_]ClO_4_ (10 mol %), MeCN (0.1 M), blue LEDs (λ_max_ = 425 nm), 16 h. Isolated yields given. The *d.r.* and *r.r.* values were determined by ^1^H NMR analysis of the crude reaction mixture.

Initially, we hypothesized that the extension of the transformation to include “activated” alkenes, such as styrenes, would be challenging, as these compounds are known to be susceptible to oxidation by the photocatalyst. Although this was indeed observed, styrene-type substrates typically exhibited an improved yield in the reaction due to their enhanced reactivity with the BCB radical cation (Figure 4).

Despite involving a direct interaction with an electron-deficient radical cation intermediate, styrene-type alkenes bearing both electron-donating and electron-withdrawing groups were viable in this transformation, and all delivered the desired products as single regioisomers (**5a**–**h**). Additionally, highly versatile functional handles such as halides (**5i**–**l**), carboxylic acids (**5m**) and boronic esters (**5n**) could also be tolerated under the reaction conditions, providing the potential for further derivatization of these substrates. When exploring the effect of alkene substitution, it was again observed that cyclic 1,2-disubstituted substrates are capable of accessing the desired BCH products, exclusively as the *cis*-diastereomer (**5o**–**q**). In the case of acyclic (*E*)-1,2-disubstituted olefins, only the *trans*-isomer is detected (**5r**–**s**), with the stereochemistry confirmed by X-ray crystallography. The limit of reactivity was located when highly electron-deficient Michael-type alkenes were observed to be unsuitable for this transformation (**5t**), making this approach complementary to previously reported radical-based BCB [2π + 2σ] cycloaddition reactions.^13,14,19^ However, subjecting 1,1-disubstituted and trisubstituted alkenes to the newly developed reaction conditions could provide access to highly substituted BCH substrates **5u**, **5v**, and **5w**, although tetrasubstituted alkenes were too sterically hindered to deliver the desired cycloadduct (**5x**).

One of the key drawbacks of previously reported BCB [2π + 2σ] cycloaddition reactions is the limited generality with respect to the alkene coupling partner, and so we next turned our attention to other classes of olefin which could be employed. In addition to vinyl naphthalene (**5y**), heterocycles containing Lewis basic atoms such as vinyl thiophene (**5z**) and thiazole (**5aa**) were amenable to the cycloaddition reaction, although vinylpyridine was deemed unsuitable (**5ab**). Interestingly, this transformation could also be used to facilitate the dearomatization of heterocycles such as benzofuran (**5ac**) and indole (**5ad**), as well as being compatible with enynes (**5ae**–**af**), dienes (**5ag**), enol ethers (**5ah**), and enamine-type substrates (**5ai**). These results demonstrate the remarkable variety of olefins that can interact with BCB radical cations and highlight the inimitable reactivity of this synthetic intermediate.

**Figure 5:**
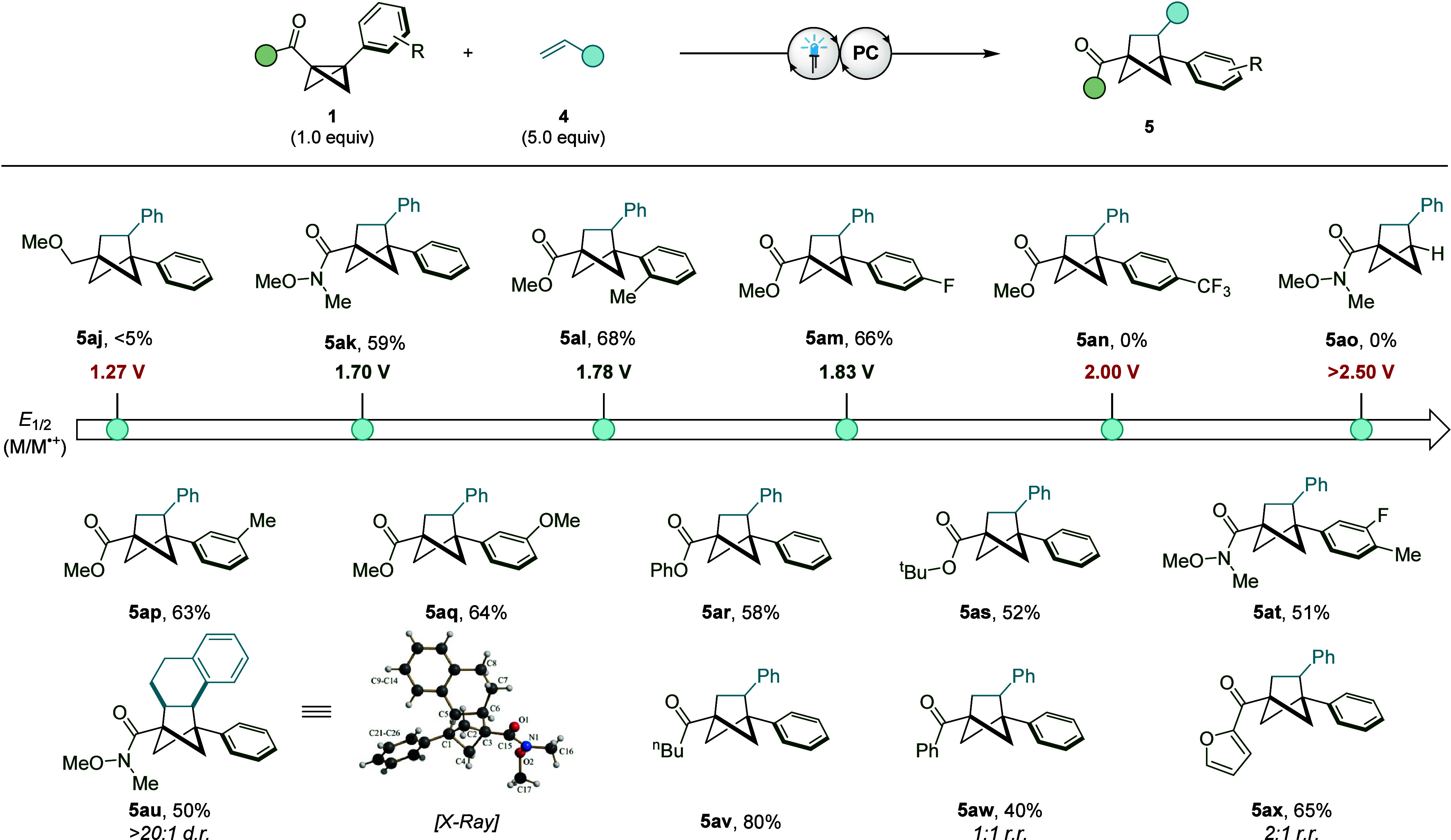
Effect of BCB oxidation potential on the [2π + 2σ] cycloaddition reaction. Reaction conditions: **1** (0.2 mmol), **4** (1.0 mmol), [Mes_2_Acr^t^Bu_2_]ClO_4_ (10 mol %), MeCN (0.1 M), blue LEDs (λ_max_ = 425 nm), 16 h. Isolated yields given. Oxidation potentials of the corresponding BCB starting materials are given in MeCN against the Ag/AgCl electrode (2 M LiCl in EtOH). The *d.r.* and *r.r.* values were determined by ^1^H NMR analysis of the crude reaction mixture.

When exploring the electronic effect of the BCB fragment, we were eager to discover whether a relationship between the aptitude for cycloaddition and the compound oxidation potential could be established (Figure 5). First, it was discovered that BCB substrates which do not bear an electron-withdrawing group possess a considerably lower oxidation potential and yield only trace product under the developed cycloaddition conditions (**5aj**). However, ester and amide containing BCB compounds, bearing aryl substitution that did not greatly perturb the substrate oxidation potential, could effectively deliver the desired BCH products (**5ak**–**am**). Considerably decreasing the electron density of the aromatic system, through the addition of a trifluoromethyl group, resulted in a BCB compound with an oxidation potential of +2.00 V which failed to deliver the desired cycloadduct and thus represents the upper limit of BCB oxidation by [Mes_2_Acr^t^Bu_2_]ClO_4_ under these conditions (**5an**). It must also be stated that removal of the aryl ring entirely resulted in a drastic increase in oxidation potential (**5ao**), presumably due to the inability of the corresponding radical cation to delocalize into the aromatic system. From the data obtained from these cyclic voltammetry studies, a redox window for reactivity was established allowing BCB compounds to first be analyzed using this technique and then only be employed if their oxidation potential falls within this potential range. Using this guiding principle, a variety of aryl substitution patterns, different ester groups, amides, and ketones were all shown to be suitable substrates in this transformation (**5ap**–**ax**).

## Mechanistic Studies

**Figure 6:**
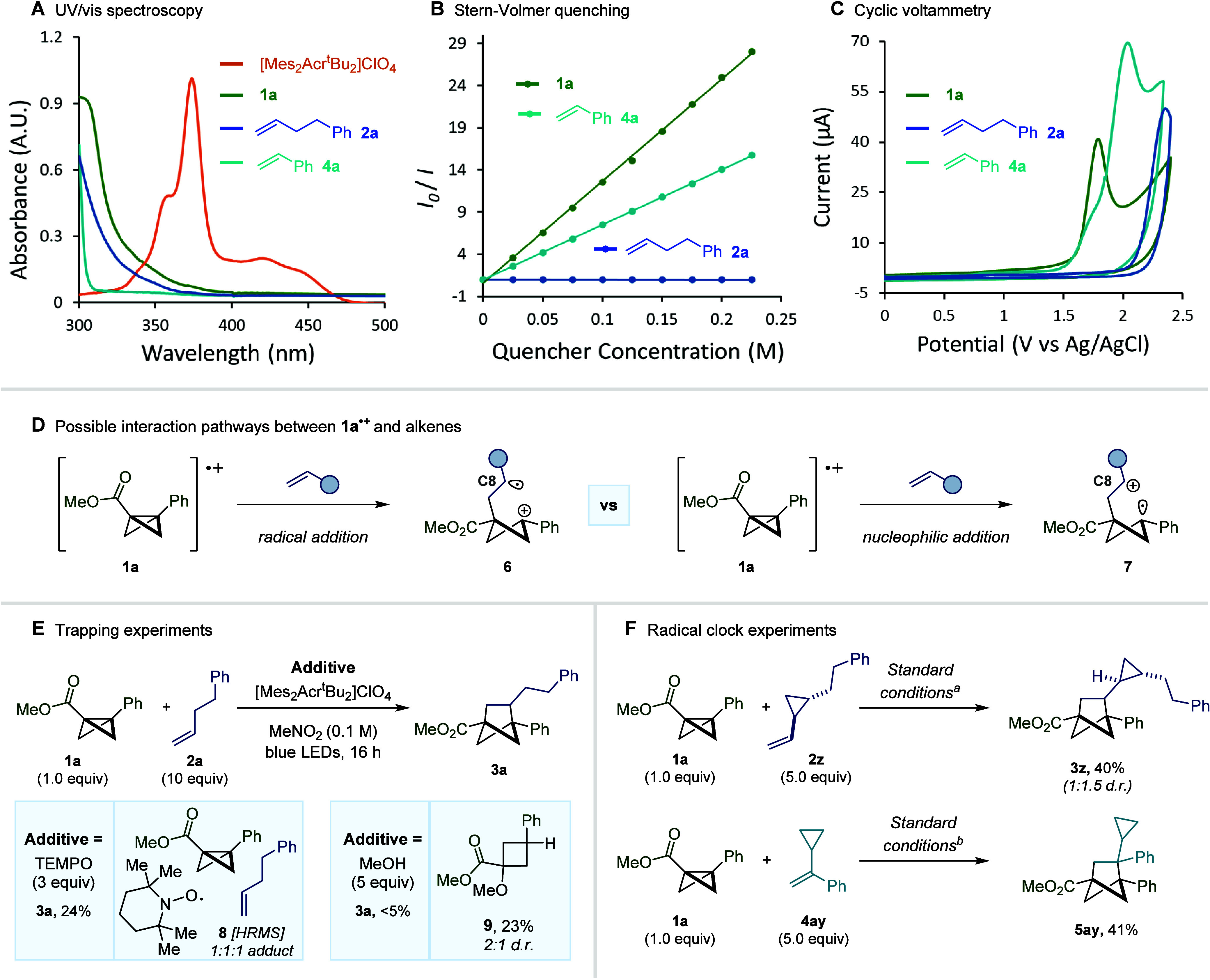
Mechanistic studies. (A) Ultraviolet–visible absorption spectra of the reaction components. (B) Stern–Volmer quenching studies. (C) Cyclic voltammetry measurements versus the Ag/AgCl reference electrode (2 M LiCl in EtOH). (D) Potential intermediates arising from the interaction of **1a**^**•+**^ and an alkene. (E) Trapping experiments. (F) Radical clock experiments. ^*a*^Standard conditions from Figure 3. ^*b*^Standard conditions from Figure 4.

To confirm that BCB radical cation **1**^**•+**^ is indeed responsible for reactivity, and to establish the mechanism of the [2π + 2σ] cycloaddition reaction, we set about designing experiments that could provide a deeper understanding of the transformation described. First, UV/vis spectroscopy of the individual reaction components revealed that the photocatalyst [Mes_2_Acr^t^Bu_2_]ClO_4_ is the only light absorbing species at λ = 425 nm, confirming that direct excitation of either BCB **1a** or alkene **2a** cannot be responsible for reactivity (Figure 6A). Furthermore, Stern–Volmer quenching studies clearly demonstrated that BCB **1a** is an effective quencher of the photocatalyst excited state, whereas alkene **2a** gave no indication that it can interact with this excited state species (Figure 6B). However, quenching was detected, albeit to a lesser extent than for **1a**, upon the addition of styrene (**4a**). These observations are in full corroboration with the cyclic voltammetry (CV) experiments that were performed (Figure 6C). Here, both BCB **1a** (+1.79 V vs Ag/AgCl) and styrene **4a** (+2.03 V vs Ag/AgCl) show oxidation peaks that were deemed accessible for the photocatalyst excited state, whereas **2a** was observed to have an oxidation potential well outside this range (+2.36 V vs Ag/AgCl). Additionally, the quantum yield for the standard reaction was calculated to be φ = 3.7, revealing that this cycloaddition transformation can proceed via a radical chain mechanism. Overall, these results strongly suggest that, in the case of nonactivated alkenes, BCB radical cation **1**^**•+**^ acts as the key intermediate in the [2π + 2σ] cycloaddition reaction. Despite undergoing oxidation by the photocatalyst, radical cations arising from styrene-type alkenes were found, during DFT studies, to be unable to lead to product formation and so this alternative pathway could be eliminated as a possibility (see Supporting Information for details). Given that the oxidation of the bicyclobutane framework constitutes an activation mode that has been underexplored in synthesis, we performed further mechanistic experiments to determine whether the initial interaction of the BCB radical cation and the alkene proceeds via radical addition to give an intermediate of type **6**, or occurs via alkene nucleophilic addition to give the corresponding “electromer” (**7**, Figure 6D).^53^ When radical trapping agent TEMPO was added to the standard reaction, product formation was not entirely suppressed, whereas the addition of MeOH resulted primarily in the formation of **9**, with only trace product being observed (Figure 6E). Furthermore, in the case of the TEMPO experiment, the observation of a 1:1:1 adduct of **1a**, alkene, and TEMPO (**8**) suggests the presence of a carbon-centered radical in the mechanism. In an attempt to establish whether a carbon-centered radical is present at the C8 position of the BCB-alkene adduct (**6**), cyclopropane-containing alkenes **2z** and **4ay** were subjected to standard reaction conditions. However, no cyclopropane ring opening could be detected in either case and cycloadducts **3z** and **5ay** were isolated in 40% and 41% yield, respectively (Figure 6F). Given that intramolecular cyclization may still occur at a faster rate than cyclopropane ring-opening, these results are not entirely conclusive in elucidating the nature of the initial interaction of the BCB radical cation and the alkene. Consequently, we turned to computational calculations to provide key insights into the operative mechanism.

## DFT Calculations

**Figure 7:**
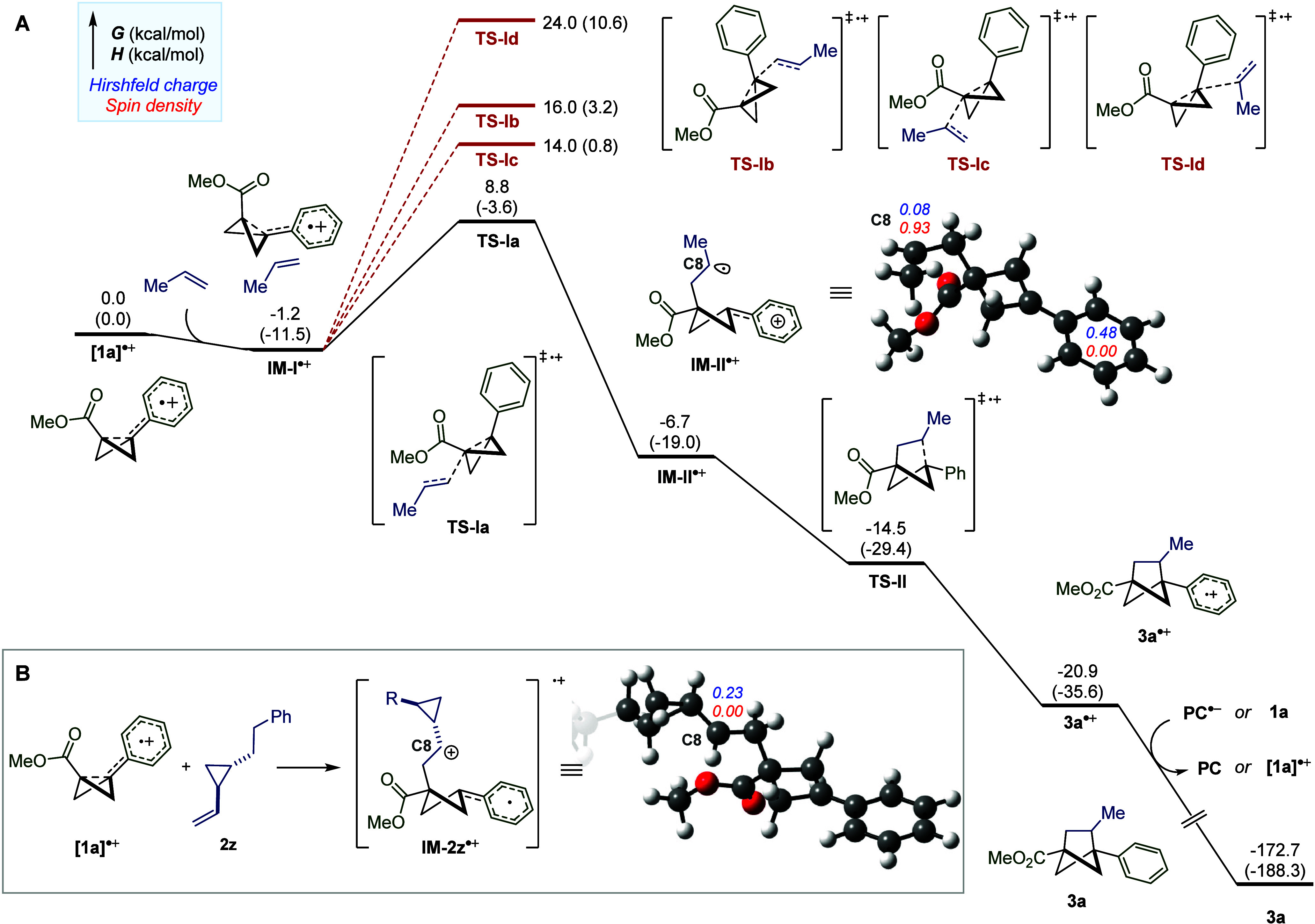
(A) Computed reaction coordinate profile of the [2π + 2σ] cycloaddition reaction between BCB radical cation **1a**^**•+**^ and propene. (B) Computed spin densities and Hirshfeld charges of radical clock intermediate **IM-2z**^**•+**^. All DFT calculations were conducted at either ωb97xd/def-TZVPP/CPCM (solvent = MeCN) or ωb97xd/def2SVP/CPCM (solvent = MeCN) levels of theory (see Supporting Information for full details).

When employing density functional theory (DFT) calculations to further study the reaction pathway, it was observed that the complexation of the radical cation **1a**^**•+**^ with a simple alkene (propene) to form **IM-I**^**•+**^ is exergonic by 1.2 kcal/mol (Figure 7A). Subsequent insertion of the alkene fragment into the BCB scaffold was found to be a kinetically facile process (**TS-Ia**), with a free energy barrier of 10.0 kcal/mol with respect to the preceding **IM-I**^**•+**^. To rationalize the regiochemistry of this initial bond forming process, all other possible transition states (**TS-Ib**, **TS-Ic**, and **TS-Id**) were computed and were all found to have significantly higher free energy barriers. From **TS-Ia**, formation of the subsequent intermediate **IM-II**^**•+**^ was determined to be exergonic by 5.5 kcal/mol.

Upon closer analysis of **IM-II**^**•+**^ we identified that the spin density of the radical cation is highly localized on the propene α-carbon (C8). In order to rationalize the results of the radical clock experiments, we also calculated the spin densities for the corresponding intermediate with substrate **2z** (**IM-2z**^**•+**^, Figure 7B) and found that, in this case, no spin density is localized on the carbon adjacent to the cyclopropane (C8). From this, we can conclude that the distribution of spin and charge density in the intermediate following the initial interaction of **1a**^**•+**^ with olefins is highly dependent on the nature of the alkene substituents. The final C–C bond formation step in the mechanism (**TS-II**) was found to be a barrierless process, with an estimated free energy of −14.5 kcal/mol using a restrained calculation (see Supporting Information). These results also indicate that the intramolecular ring-closure step would occur at a faster rate than cyclopropane ring-opening.^54^ Finally, reduction of the thermodynamically stable **3a**^**•+**^ (−20.9 kcal/mol) can then occur from either the reduced photocatalyst or a neutral BCB molecule to turn over the radical chain and generate BCH product **3a**.

## Conclusion

In conclusion, we have identified a new strategy for the single-electron oxidative activation of bicyclo[1.1.0]butane via photoredox catalysis. The synthetic utility of the resulting radical cation was highlighted by its ability to undergo [2π + 2σ] cycloaddition reactions in a highly regio- and diastereoselective fashion. The scope of the transformation with respect to the alkene coupling partner was remarkably broad, allowing the cycloaddition of styrene-type, heteroatom substituted, and, for the first time in this reaction class, nonactivated alkenes. A comprehensive experimental and computational mechanistic study was undertaken that confirmed the involvement of BCB radical cations and illuminated the nature of their interaction with olefins. We foresee that the work presented above can serve as a platform from which further studies into the potential synthetic applications of bicyclo[1.1.0]butyl radical cations can be built upon.

## Supporting Information Available

Experimental details, details on the mechanistic investigations and computational calculations, crystallographic data [**5r** (CCDC 2331780), **5ad** (CCDC 2331781), **5ad′** (CCDC 2331782), and **5au** (CCDC 2331783)], and characterization data including NMR spectra (PDF)
