# Supplementary material for: Bicyclo[1.1.0]butyl Radical Cations: Synthesis and Application to [2π + 2σ] Cycloaddition Reactions
Source: J Am Chem Soc. 2024 May 29;146(23):16237–47. doi: 10.1021/jacs.4c04403 (PMC11177261; doi:10.1021/jacs.4c04403)
Supplement: Supplementary file 1 — ja4c04403_si_001.pdf [file ja4c04403_si_001.pdf]

Supporting Information

Bicyclo[1.1.0]butyl Radical Cations: Synthesis and Application to [2π+2σ] Cycloaddition Reactions

Jasper L. Tyler[1,3], Felix Schäfer[1,3], Huiling Shao[2], Colin Stein[1], Audrey Wong[2], Constantin G. Daniliuc[1], K. N. Houk[2,*] and Frank Glorius[1,*]

[1]Organisch-Chemisches Institut, Universität Münster, 48149 Münster, Germany.

[2]Department of Chemistry and Biochemistry, University of California, Los Angeles, CA 90095-1569, USA.

[3]These authors contributed equally.

*Correspondence to: glorius@uni-muenster.de; houk@chem.ucla.edu

TABLE OF CONTENTS

1. MATERIALS AND GENERAL METHODS 3
1.1. Glassware, Solvents and Reagents 3
1.2. Chromatography and Data Analysis 3
1.3. Photochemical Set-up and Light Sources 3
1.4. Naming of Compounds 4
2. EXPERIMENTAL DATA 5
2.1. General Procedures 5
2.1.1. **General Procedure A**: Synthesis of bicyclo[2.1.1]hexanes from non-activated alkenes 5
2.1.2. **General Procedure B**: Synthesis of bicyclo[2.1.1]hexanes from activated alkenes 5
2.1.3. **General Procedure C**: Synthesis of alkenyl substrates 6
2.2. Synthesis of Alkene Starting Materials 7
2.3. Synthesis of Bicyclo[1.1.0]butanes 11
2.4. Synthesis of Bicyclo[2.1.1]hexanes 15
2.5. Reaction Optimization for Non-Activated Alkenes 58
2.5.1. Establishing the photocatalyst 58
2.5.2. Solvent and alkene stoichiometry 59
2.5.3. Catalyst loading, concentration and wavelength of light 60
2.6. Reaction Optimization for Activated Alkenes 61
2.6.1. Reaction time 61
2.6.2. Solvent and alkene stoichiometry 62
2.6.3. Catalyst counterion 63
2.7. Substrate Limitations 63
2.8. Inversion of Reaction Stoichiometry 65
2.9. Sensitivity Screen 66
3. MECHANISTIC INVESTIGATIONS 68
3.1. UV/vis Absorption Spectroscopy 68
3.2. Stern-Volmer Analysis 68
3.3. Cyclic Voltammetry 69
3.3.1. Redox potential measurements for **1a**, **2a** and **4a** 69
3.3.2. Redox potential measurements of BCB substrates 70
3.4. Quantum Yield Calculation 71
3.4.1. Determination of the photon flux 71
3.4.2. Determination of the reaction quantum yield 72
3.5. Trapping Experiments 73
3.6. Radical Clock Experiments 74
4. COMPUTATIONAL CALCULATIONS 76
4.1. Computational Methods 76

4.2. Nature of the BCB Radical Cation 76
4.3. Comparison of BCB and Styrene Radical Cations 77
4.4. Computed Reaction Coordinate Profile of BCB Radical Cation (**1a**$^{\bullet+}$) with Styrene 77
4.4.1. Interaction between styrene radical cations and ground state BCB 78
4.5. Scan of the **TS-II** reaction coordinate 79
4.6. Computed Cartesian Coordinates 79
4.6.1. Manuscript structures 79
4.6.2. Supporting information structures 95
5. X-RAY CRYSTALLOGRAPHY 109
5.1. X-Ray Diffraction Data 109
6. SPECTROSCOPIC DATA 117
7. REFERENCES 201

1. MATERIALS AND GENERAL METHODS

1.1. Glassware, Solvents and Reagents

All reactions were conducted under an inert atmosphere of argon using Schlenk manifold techniques unless stated otherwise. All glassware and Teflon-coated magnetic stir bars were dried in an oven at 120 °C prior to use. All anhydrous solvents were commercially supplied and stored over 3 Å mol sieves or dried using an activated alumina column drying system (MeCN, CH_2Cl_2, hexane, toluene, THF, Et_2O). Reagents were purchased from commercial sources and used as received. Exception: $MeNO_2$ was degassed before use. Photocatalysts $[Ru(bpy)_3]Cl_2$ (bpy = 2,2'-bipyridine),[1] $[Ir(ppy)_2(dtbbpy)](PF_6)$ (ppy = 2-phenylpyridin, dtbbpy = 4,4'-di-tert-butyl-2,2'-bipyridine),[2] 4CzIPN = 2,4,5,6-tetra(9H-carbazol-9-yl)isophthalonitrile,[3] OMe-thioxanthylium = 1,3,6,8-tetramethoxy-9-phenylthioxanthylium trifluoromethanesulfonate[4] and $[Mes_2Acr^tBu_2]ClO_4$ = 3,6-di-*tert*-butyl-9,10-dimesitylacridin-10-ium perchlorate[5] were synthesized according to the corresponding literature procedures.

1.2. Chromatography and Data Analysis

Thin layer chromatography (TLC) was performed to monitor reactions when practical using Merck silica gel 60 F_{254} aluminum plates and visualized under UV light, or by staining with aqueous basic potassium permanganate followed by heating. **Flash column chromatography** (FCC) was carried out using Acros Organics silica gel (35–70 mesh) or a Biotage Isolera™ flash purification system. **NMR spectra** were recorded on a Bruker Avance II 400, Agilent DD2 500 or DD2 600 spectrometers. All spectral data was acquired at 295 K. Deuterated solvents were purchased from Eurisotop ($CDCl_3$, deuteration > 99.8%). Chemical shifts (δ) are reported in parts per million (ppm) and referenced to $CDCl_3$ (^1H: 7.26 ppm; ^{13}C: 77.16 ppm). Coupling constants (*J*) are given in Hertz (Hz) and refer to corresponding multiplicities (s = singlet, d = doublet, t = triplet, q = quartet, quin = quintet, hex = hextet, h = heptet, m = multiplet, app = apparent, br. = broad signal, dd = doublet of doublets, etc.). The ^1H NMR spectra are reported as follows: chemical shift (multiplicity, coupling constants, number of protons). NMR assignments were made according to spin systems, using two-dimensional NMR spectroscopy (COSY, HSQC, HMBC) to assist the characterization. NMR yields were determined by ^1H NMR analysis using dibromomethane as an internal standard. The *d.r.* and *r.r.* values were determined by ^1H NMR analysis of the crude reaction mixture. When only a single regioisomer was detected, no *r.r.* is given. >20:1 *d.r.* indicates when only a single diastereomer could be detected. **High resolution mass spectra (HRMS)** were recorded using electrospray ionization (ESI) on a Bruker Daltonics, MicroToF spectrometer and calibrated using formate ion clusters.

1.3. Photochemical Set-up and Light Sources

Photochemical reactions were performed in a Hepatochem EvoluChemTM PhotoRedOx Box Duo device and irradiated with two EvoluChem™ HCK1012-02-012 LEDs (18 W, λ_{max} = 425 nm). When the internal fan was used, the reaction temperature was determined to be between 30 °C and 33 °C. When the fan was not used

the reaction temperature was determined to be between 45 °C and 50 °C.

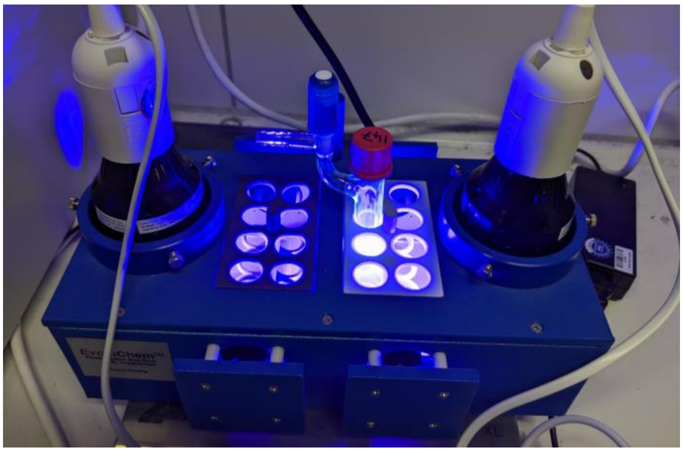

Figure S1: Experimental set-up for photochemical reactions

1.4. Naming of Compounds

Compound names are those generated by ChemDraw Professional 20.0 software (PerkinElmer), following the IUPAC nomenclature.

2. EXPERIMENTAL DATA

2.1. General Procedures

2.1.1. General Procedure A: Synthesis of bicyclo[2.1.1]hexanes from non-activated alkenes

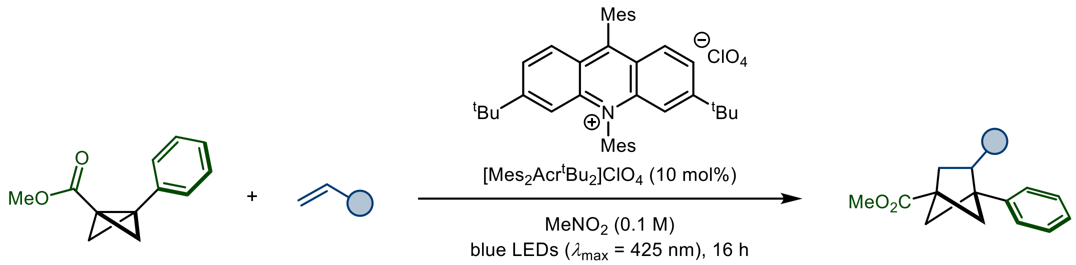

To an oven-dried 10 mL Schlenk tube equipped with a Teflon-coated magnetic stir bar was added [$Mes_2Acr^tBu_2$]ClO_4 (12.6 mg, 20.0 µmol, 10 mol%), the respective olefin **2** (2.0 mmol, 10 equiv), and bicyclo[1.1.0]butane (BCB) **1a** (37.7 mg, 0.200 mmol, 1.00 equiv). The Schlenk tube was evacuated and backfilled with argon three times before $MeNO_2$ (2.0 mL) was added under a positive argon pressure. The reaction mixture was stirred under irradiation with blue LEDs (18 W, λ_{max} = 425 nm) for 16 h.[A] After this time, the solvent was removed under reduced pressure and the crude product was purified by flash column chromatography on silica gel to yield the corresponding bicyclo[2.1.1]hexane (**3**).

Note: (**A**) No fan cooling was used during irradiation, allowing the reaction vessel to reach temperatures of approximately 45 °C.

2.1.2. General Procedure B: Synthesis of bicyclo[2.1.1]hexanes from activated alkenes

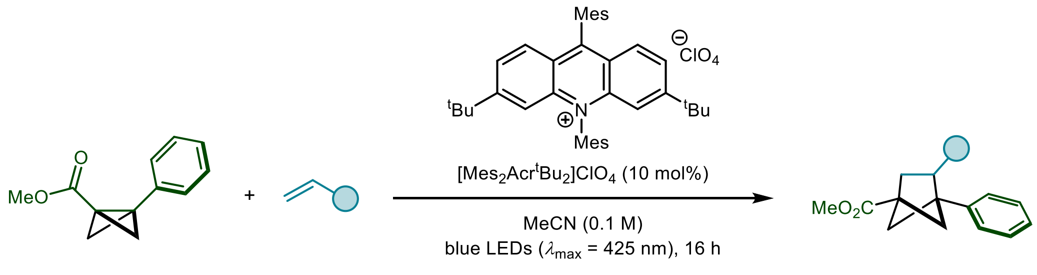

To an oven-dried 10 mL Schlenk tube equipped with a Teflon-coated magnetic stir bar was added [$Mes_2Acr^tBu_2$]ClO_4 (12.6 mg, 20.0 µmol, 10 mol%), the respective olefin **4** (1.0 mmol, 5.0 equiv), and bicyclo[1.1.0]butane (BCB) **1** (0.20 mmol, 1.0 equiv). The Schlenk tube was evacuated and backfilled with argon three times before MeCN (2.0 mL) was added under a positive argon pressure. The reaction mixture was stirred under irradiation with blue LEDs (18 W, λ_{max} = 425 nm) for 16 h.[A] After this time, the solvent was removed under reduced pressure and the crude product was purified by flash column chromatography on silica gel to yield the corresponding bicyclo[2.1.1]hexane (**5**).

Note: (**A**) No fan cooling was used during irradiation, allowing the reaction vessel to reach temperatures of

approximately 45 °C.

2.1.3. General Procedure C: Synthesis of alkenyl substrates

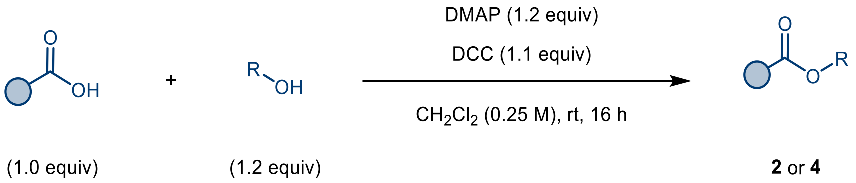

2 or **4**

To an oven-dried 50 mL Schlenk tube equipped with a Teflon-coated stir bar was added carboxylic acid (1.0 equiv), alcohol (1.2 equiv), dicyclohexylcarbodiimide (DCC, 1.1 equiv), 4-(dimethylamino)pyridine (DMAP, 1.2 equiv) and CH_2Cl_2 (0.25 M). The reaction mixture was stirred at room temperature for 16 h and then washed with 1 N HCl (3 × 10 mL), sat. aq. $NaHCO_3$ (10 mL) and brine (10 mL). The organic layer was dried over $MgSO_4$, filtered, concentrated and purified by flash column chromatography to yield the corresponding ester.

2.2. Synthesis of Alkene Starting Materials

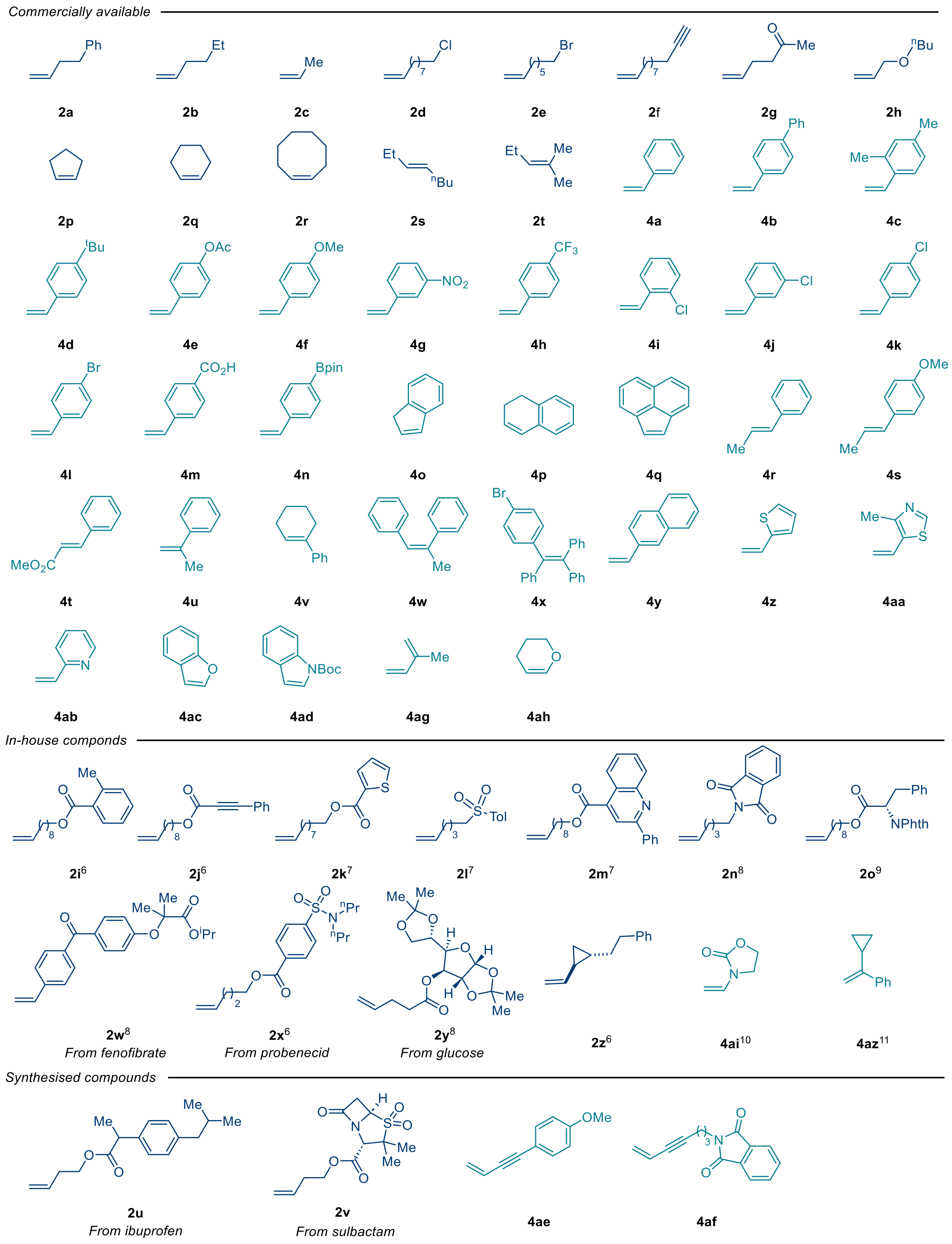

Figure S2: Preparation of alkene starting materials. In-house chemicals were previously synthesized according to the corresponding literature procedures[6–11]

But-3-en-1-yl 2-(4-isobutylphenyl)propanoate (2u)

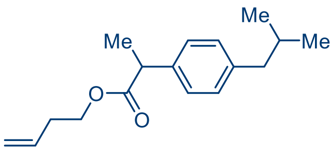

2u

Synthesized following **General Procedure C** using: ibuprofen (1.03 g, 5.00 mmol, 1.00 equiv), but-3-en-1-ol (0.516 mL, 6.00 mmol, 1.20 equiv), dicyclohexylcarbodiimide (1.13 g, 5.50 mmol, 1.10 equiv), 4-(dimethylamino)pyridine (0.733 g, 6.00 mmol, 1.20 equiv) and CH_2Cl_2 (20 mL). Purified by flash column chromatography (SiO_2; 100:0 to 95:5 pentane:EtOAc) to afford **2u** (0.978 g, 3.76 mmol, 75%) as a colourless liquid.

TLC: R_f = 0.53 (95:5 pentane:EtOAc).

NMR Spectroscopy (*see spectra*):

^{1}**H NMR** (500 MHz, $CDCl_3$): δ_H 7.20 (d, *J* = 8.1 Hz, 2H), 7.09 (d, *J* = 8.1 Hz, 2H), 5.69 (ddt, *J* = 17.1, 10.3, 6.8 Hz, 1H), 5.06 – 4.97 (m, 2H), 4.12 (t, *J* = 6.7 Hz, 2H), 3.68 (q, *J* = 7.2 Hz, 1H), 2.45 (d, *J* = 7.2 Hz, 2H), 2.36 – 2.26 (m, 2H), 1.92 – 1.79 (m, 1H), 1.48 (d, *J* = 7.2 Hz, 3H), 0.90 (d, *J* = 6.6 Hz, 6H) ppm;

^{13}**C NMR** (126 MHz, $CDCl_3$): δ_C 174.8, 140.6, 137.9, 134.1, 129.4, 127.3, 117.3, 63.8, 45.3, 45.2, 33.2, 30.3, 22.5, 18.6 ppm.

HRMS (ESI$^+$): m/z calc'd for $C_{17}H_{24}O_2Na$ $[M+Na]^+$: 283.16679, found: 283.16685.

But-3-en-1-yl-(2S,5R)-3,3-dimethyl-7-oxo-4-thia-1-azabicyclo[3.2.0]heptane-2-carboxylate 4,4-dioxide (2v)

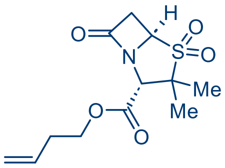

2v

Synthesized following **General Procedure C** using: sulbactam (1.17 g, 5.00 mmol, 1.00 equiv), but-3-en-1-ol (0.516 mL, 6.00 mmol, 1.20 equiv), dicyclohexylcarbodiimide (1.13 g, 5.50 mmol, 1.10 equiv), 4-(dimethylamino)pyridine (0.733 g, 6.00 mmol, 1.20 equiv) and CH_2Cl_2 (20 mL). Purified by flash column chromatography (SiO_2; 100:0 to 70:30 pentane:EtOAc) to afford **2v** (0.951 g, 3.31 mmol, 66%) as a colourless oil.

TLC: R_f = 0.46 (70:30 pentane:EtOAc).

NMR Spectroscopy (*see spectra*):

^{1}**H NMR** (400 MHz, $CDCl_3$): δ_H 5.77 (ddt, *J* = 17.0, 10.3, 6.7 Hz, 1H), 5.20 – 5.11 (m, 2H), 4.61 (dd, *J* = 4.1, 2.3 Hz, 1H), 4.38 (s, 1H), 4.36 – 4.20 (m, 2H), 3.54 – 3.38 (m, 2H), 2.53 – 2.34 (m, 2H), 1.61 (s, 3H), 1.42

(s, 3H) ppm;

^{13}C NMR (101 MHz, $CDCl_3$): δ_C 170.8, 167.1, 133.4, 118.3, 65.5, 63.4, 62.8, 61.2, 38.4, 33.0, 20.5, 18.6 ppm.

HRMS (ESI$^+$): m/z calc'd for $C_{12}H_{17}NO_5SNa$ [M+Na]$^+$: 310.07195, found: 310.07196.

1-(But-3-en-1-yn-1-yl)-4-methoxybenzene (4ae)

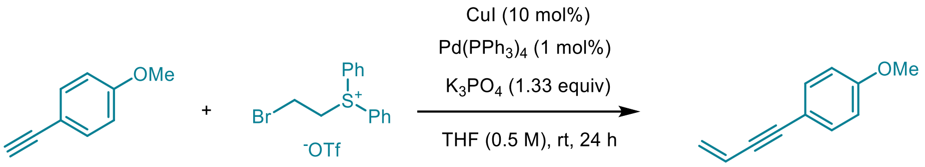

(1.00 equiv) (1.33 equiv) **4ae**

The title compound was prepared according to a modified literature procedure.[12] To an oven dried 50 mL Schlenk tube was added 2-bromoethyldiphenylsulfonium trifluoromethanesulfonate[13] (1.33 g, 3.00 mmol, 1.33 equiv), potassium phosphate tribasic (637 mg, 3.00 mmol, 1.33 equiv), tetrakis(triphenylphosphine)palladium (26.0 mg, 0.0225 mmol, 1 mol%) and copper iodide (43.0 mg, 0.225 mmol, 10 mol%). The tube was evacuated and backfilled with argon three times. 1-Ethynyl-4-methoxybenzene (297 mg, 2.25 mmol, 1.00 equiv), followed by THF (4.5 mL) were added under a positive argon pressure. The resulting suspension was stirred for 24 hours before diluting with diethyl ether (40 mL) and H_2O (25 mL). The layers were separated, and the aqueous layer was extracted with diethyl ether (25 mL). The combined organic layers were dried over $MgSO_4$, filtered, and concentrated under reduced pressure. The residue was purified by flash column chromatography (SiO_2; 100:0 to 95:5 pentane:Et_2O) to afford **4ae** (333 mg, 2.11 mmol, 94%) as a colourless oil.

TLC: R_f = 0.55 (pentane).

NMR Spectroscopy (*see spectra*):

^{1}H NMR (400 MHz, $CDCl_3$): δ_H 7.43 – 7.34 (m, 2H), 6.89 – 6.81 (m, 2H), 6.01 (dd, J = 17.5, 11.1 Hz, 1H), 5.69 (dd, J = 17.5, 2.1 Hz, 1H), 5.50 (dd, J = 11.1, 2.1 Hz, 1H), 3.81 (s, 3H) ppm;

^{13}C NMR (101 MHz, $CDCl_3$): δ_C 159.8, 133.2, 126.2, 117.5, 115.4, 114.1, 90.2, 87.0, 55.4 ppm.

GC-MS (EI): m/z calc'd for $C_{11}H_{10}O$ [M]$^+$: 158.1, found: 158.1.

2-(Hept-6-en-4-yn-1-yl)isoindoline-1,3-dione (4af)

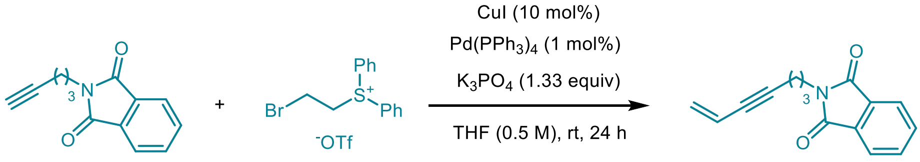

(1.00 equiv) (1.33 equiv) **4af**

The title compound was prepared according to a modified literature procedure.[12] To an oven dried 50 mL Schlenk tube was added 2-bromoethyldiphenylsulfonium trifluoromethanesulfonate[13] (1.77 g, 4.00 mmol, 1.33 equiv), potassium phosphate tribasic (850 mg, 4.00 mmol, 1.33 equiv), tetrakis(triphenylphosphin)palladium (35.0 mg, 0.0225 mmol, 1 mol%) and copper iodide (57.0 mg, 0.225 mmol, 10 mol%). The tube was evacuated and backfilled with argon three times. 2-Pent-4-yn-1-yl-isoindoline-1,3-dione (640 mg, 3.00 mmol, 1.00 equiv), followed by THF (6 mL) were added under a positive argon pressure. The resulting suspension was stirred for 24 hours before diluting with Et_2O (50 mL) and H_2O (30 mL). The layers were separated, and the aqueous layer extracted with Et_2O (30 mL). The combined organic layers were dried over $MgSO_4$, filtered, and concentrated under reduced pressure. The residue was purified by flash column chromatography (SiO_2; 49.5:49.5:1.0 CH_2Cl_2:pentane:EtOAc) to afford **4af** (605 mg, 2.53 mmol, 84%) as a yellow oil.

TLC: R_f = 0.55 (49.5:49.5:1.0 CH_2Cl_2:pentane:EtOAc).

NMR Spectroscopy (*see spectra*):

> **^{1}H NMR** (400 MHz, $CDCl_3$): δ_H 7.86 – 7.80 (m, 2H), 7.72 – 7.67 (m, 2H), 5.60 (ddt, *J* = 17.5, 11.0, 2.1 Hz, 1H), 5.42 (dd, *J* = 17.5, 2.3 Hz, 1H), 5.28 (dd, *J* = 11.0, 2.3 Hz, 1H), 3.79 (t, *J* = 7.0 Hz, 2H), 2.38 (td, *J* = 7.0, 2.1 Hz, 2H), 1.93 (p, *J* = 7.0 Hz, 2H) ppm;
>
> **^{13}C NMR** (101 MHz, $CDCl_3$): δ_C 168.5, 134.0, 132.3, 125.9, 123.3, 117.3, 89.5, 80.1, 37.5, 27.4, 17.4 ppm.

HRMS (ESI^+): m/z calc'd for $C_{15}H_{13}NO_2Na$ $[M+Na]^+$: 262.0839, found: 262.0837.

2.3. Synthesis of Bicyclo[1.1.0]butanes

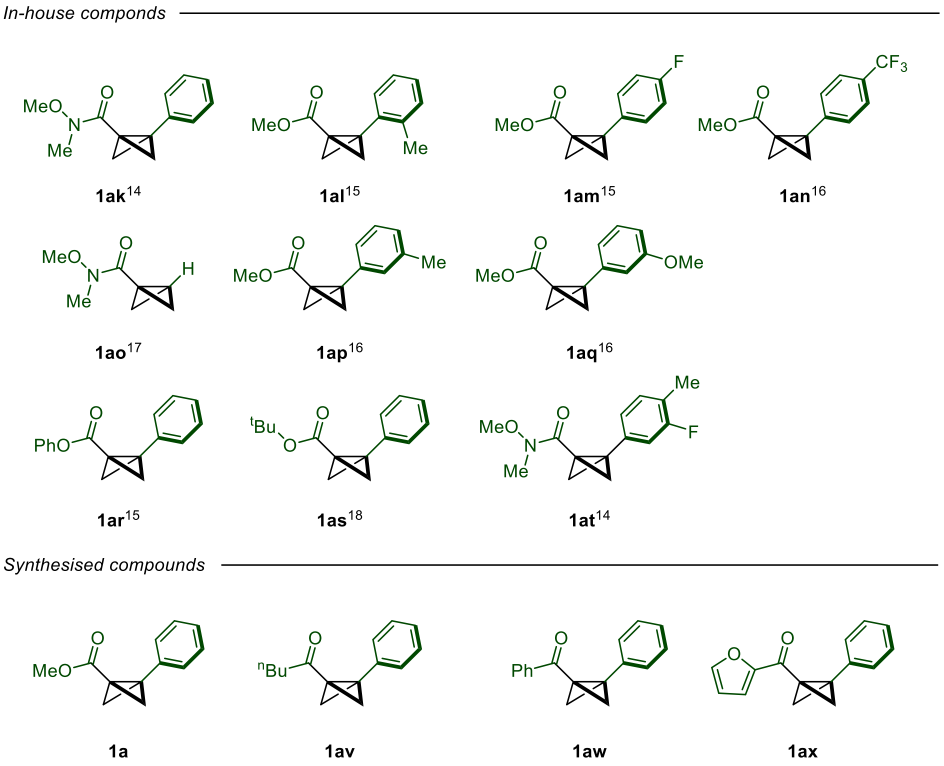

Figure S3: Preparation of bicyclo[1.1.0]butanes starting materials. In-house chemicals were previously Synthesized according to the corresponding literature procedures[14–18]

Methyl-3-phenylbicyclo[1.1.0]butane-1-carboxylate (1a)

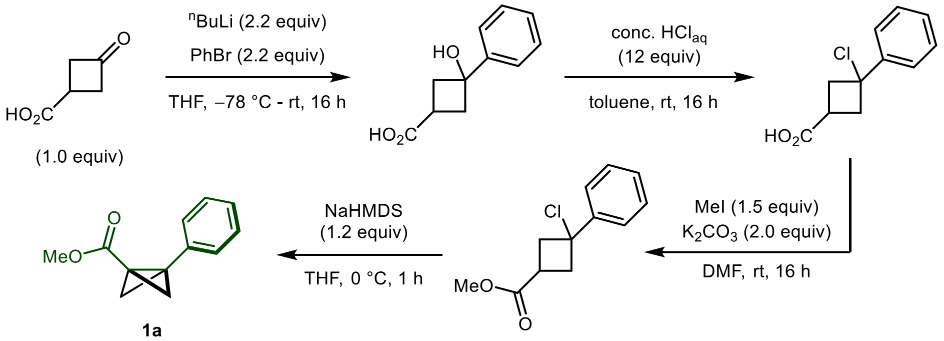

1a

Step 1:

To a solution of phenyl bromide (2.2 equiv)[A] in THF (0.7 M) was added ⁿBuLi (1.6 M, 2.2 equiv) dropwise at −78 °C. The mixture was stirred for 1 h at −78 °C before a solution of 3-oxocyclobutane-1-carboxylic acid (12.0 g, 90.5 mmol, 1.00 equiv) in THF (2.5 M) was added. The mixture was allowed to slowly warm to room temperature overnight and then quenched with saturated NH_4Cl solution before being basified with 4 M NaOH solution and washed with EtOAc. The aqueous layer was then acidified with 2 M HCl solution and extracted with EtOAc. The organic layer was dried over $MgSO_4$ and evaporated under reduced pressure to afford 3-hydroxy-3-

phenylcyclobutane-1-carboxylic acid as a white solid. This material was used directly in the next step without further purification.

Note: (**A**) Commercial PhLi solution can be used to yield similar results, but we do not recommend PhMgBr for practicality reasons (transfer problems, solid precipitate interfering with stirring).

Step 2:

To a solution of phenylcyclobutane-1-carboxylic acid (90.5 mmol, 1.0 equiv) in toluene (0.8 M) was added concentrated HCl (12 equiv) at room temperature. The resulting mixture was stirred at room temperature overnight. The organic layer was separated, washed with H_2O and brine, dried (Na_2SO_4), filtered, and concentrated under reduced pressure to provide 3-chloro-3-phenylcyclobutane-1-carboxylic acid as a white solid (19.0 g, 90.0 mmol, 99% over 2 steps). This material was used directly in the next step without further purification.

Step 3:

Phenyl-3-chlorocyclobutane-1-carboxylic acid (19.0 g, 90.0 mmol, 1.00 equiv) was added to a round-bottomed flask. The reaction vessel was evacuated and backfilled with argon three times. DMF (0.5 M) was added into the reaction vessel followed by K_2CO_3 (2.0 equiv) and MeI (1.5 equiv). The reaction mixture was stirred overnight at room temperature. After this time, the solution was diluted with EtOAc (100 mL), washed with brine (3×50 mL) and the organic layer was seperated and dried (Na_2SO_4). The resulting solution was then filtered and concentrated to dryness. The obtained residue was purified by flash column chromatography (SiO_2; 100:0 to 90:10 pentane:Et_2O) to afford methyl 3-chloro-3-phenylcyclobutane-1-carboxylate (8.20 g, 36.5 mmol, 41%) as a white solid.

Step 4:

To a solution of methyl 3-chloro-3-phenylcyclobutane-1-carboxylate (8.20 g, 36.5 mmol, 1.00 equiv) in THF (0.4 M) was added 2 M NaHMDS (1.2 equiv) at 0 °C. The resulting mixture was stirred for 1 h at 0 °C before the reaction was slowly quenched with NH_4Cl (sat. aq.). The mixture was then diluted with hexane (25 mL). The organic layer was separated and washed with brine (25 mL), dried over Na_2SO_4, filtered through SiO_2 and concentrated under reduced pressure. The crude reaction mixture was purified by flash column chromatography (SiO_2; 100:0 to 90:10 pentane: EtOAc) to yield methyl 3-phenylbicyclo[1.1.0]butane-1-carboxylate (6.50 g, 34.5 mmol, 95%).

TLC: R_f = 0.50 (95:5 pentane:EtOAc).

NMR Spectroscopy (*see spectra*):

> **^{1}H NMR** (400 MHz, $CDCl_3$): δ_H 7.28 – 7.25 (m, 4H), 7.24 – 7.17 (m, 1H), 3.44 (s, 3H), 2.90 (t, *J* = 1.2 Hz, 2H), 1.57 (t, *J* = 1.2 Hz, 2H) ppm;
>
> **^{13}C NMR** (101 MHz, $CDCl_3$): δ_C 170.1, 133.7, 128.5, 127.1, 126.0, 51.8, 35.8, 33.0, 23.3 ppm.

HRMS (ESI^+): m/z calc'd for $C_{12}H_{12}O_2$ $[M+Na]^+$: 211.07295, found: 211.07289.

1-3-Phenylbicyclo[1.1.0]butan-1-yl)pentan-1-one (1av)

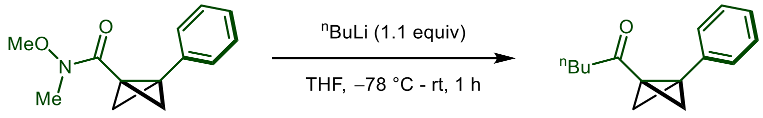

To an oven dried 10 mL Schlenk tube was added **1ak** (109 mg, 0.500 mmol, 1.00 equiv) and THF (1 mL). The solution was cooled to −78 °C and ^{n}BuLi 1.6 M in hexane (0.344 mL, 0.550 mmol, 1.10 equiv) was added dropwise. The resulting solution was warmed to room temperature and stirred for 1 h. The reaction was quenched with sat. aq. NH_4Cl and extracted with Et_2O (3×5 mL). The combined organic layers were dried over $MgSO_4$, filtered, and concentrated under reduced pressure to afford **1av** (107 mg, 0.500 mmol, 100%) as a white solid.

TLC: R_f = 0.49 (95:5 pentane:EtOAc).

NMR Spectroscopy (*see spectra*):

> **^{1}H NMR** (400 MHz, $CDCl_3$): δ_H 7.33 – 7.29 (m, 1H), 7.29 – 7.26 (m, 3H), 7.26 – 7.20 (m, 1H), 3.02 (t, *J* = 1.3 Hz, 2H), 2.06 (t, *J* = 7.3 Hz, 2H), 1.63 (t, *J* = 1.3 Hz, 2H), 1.39 – 1.26 (m, 2H), 1.13 – 0.98 (m, 2H), 0.73 (t, *J* = 7.3 Hz, 3H) ppm;

> **^{13}C NMR** (101 MHz, $CDCl_3$): δ_C 203.1, 133.4, 128.8, 127.3, 125.7, 39.2, 36.8, 35.5, 32.5, 25.7, 22.3, 13.9 ppm.

HRMS (ESI$^+$): m/z calc'd for $C_{15}H_{18}O$ $[M+Na]^+$: 237.12499, found: 237.12501.

Phenyl(-3-phenylbicyclo[1.1.0]butan-1-yl)methanone (1aw)

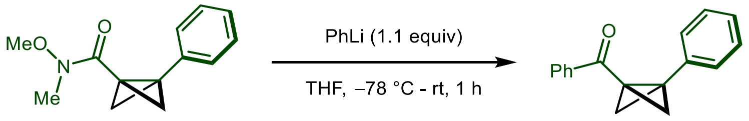

To an oven dried 10 mL Schlenk tube was added **1ak** (65.1 mg, 0.300 mmol, 1.00 equiv) and THF (2 mL). The solution was cooled to −78 °C and PhLi 1.9 M in Bu_2O (0.174 mL, 0.330 mmol, 1.10 equiv) was added dropwise. The resulting solution was warmed to room temperature and stirred for 1 h. The reaction was quenched with sat. aq. NH_4Cl and extracted with Et_2O (3×5 mL). The combined organic layers were dried over $MgSO_4$, filtered, and concentrated under reduced pressure to afford **1aw** (62.8 mg, 0.268 mmol, 89%) as a white solid.

NMR Spectroscopy (*see spectra*):

^{1}H NMR (400 MHz, $CDCl_3$): δ_H 7.57 – 7.50 (m, 2H), 7.48 – 7.42 (m, 1H), 7.38 – 7.32 (m, 2H), 7.24 – 7.19 (m, 3H), 7.16 – 7.12 (m, 2H), 3.16 (t, *J* = 1.3 Hz, 2H), 1.91 (t, *J* = 1.3 Hz, 2H) ppm;

^{13}C NMR (101 MHz, $CDCl_3$): δ_C 196.7, 138.5, 133.0, 132.0, 128.5, 128.5, 128.1, 127.4, 126.2, 38.7, 37.8, 31.4 ppm.

HRMS (ESI$^+$): m/z calc'd for $C_{17}H_{14}ONa$ $[M+Na]^+$: 257.09369, found: 257.09362.

Furan-2-yl-3-phenylbicyclo[1.1.0]butan-1-yl)methanone (1ax)

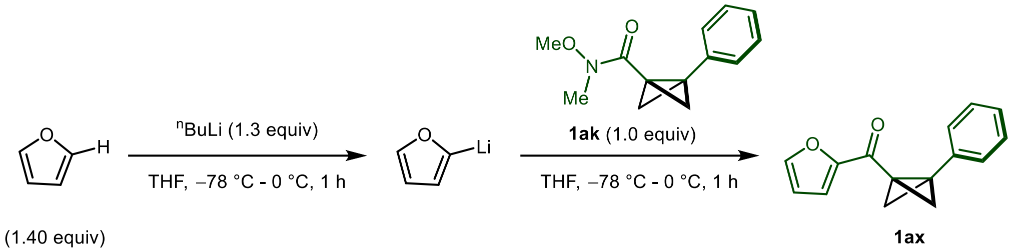

To an oven-dried 25 mL Schlenk tube was added THF (2.0 mL) and furan (101 µL, 1.40 mmol, 1.00 equiv). The solution was cooled to −78 °C before ^{n}BuLi (1.6 M in hexane, 813 µL, 1.30 mmol, 1.30 equiv) was added dropwise. The reaction mixture was gradually warmed to 0 °C and stirred for one hour, after which the reaction vessel was cooled again to −78 °C. N-methoxy-N-methyl-3-phenylbicyclo[1.1.0]butane-1-carboxamide **1ak** (217 mg, 1.00 mmol, 1.00 equiv), dissolved in THF (2.0 mL), was then added dropwise. The reaction mixture was then stirred for 1 hour while gradually warming to room temperature before being quenched with H_2O (1 mL), followed by sat. aq. NH_4Cl (5 mL). The solution was extracted with Et_2O (3×5 mL) and the combined organic layers were dried over $MgSO_4$, filtered, and concentrated under reduced pressure. The crude reaction mixture was purified by flash column chromatography (SiO_2; 90:10 pentane: Et_2O) to yield **1ax** (208 mg, 0.926 mmol, 93%) as a white solid.

TLC: R_f = 0.25 (90:10 pentane: Et_2O).

NMR Spectroscopy (*see spectra*):

^{1}H NMR (400 MHz, $CDCl_3$): δ_H 7.53 (dd, *J* = 1.7, 0.8 Hz, 1H), 7.31 – 7.18 (m, 5H), 7.08 (dd, *J* = 3.6, 0.8 Hz, 1H), 6.47 (dd, *J* = 3.5, 1.7 Hz, 1H), 3.40 (t, *J* = 1.2 Hz, 2H), 1.85 (t, *J* = 1.2 Hz, 2H) ppm;

^{13}C NMR (101 MHz, $CDCl_3$): δ_C 182.9, 153.2, 145.9, 133.1, 128.6, 127.4, 126.2, 117.4, 111.9, 39.2, 37.0, 30.9 ppm

HRMS (ESI$^+$): m/z calc'd for $C_{15}H_{12}O_2Na$ $[M+Na]^+$: 247.0730, found: 247.0728.

2.4. Synthesis of Bicyclo[2.1.1]hexanes

Methyl-3-phenethyl-4-phenylbicyclo[2.1.1]hexane-1-carboxylate (3a)

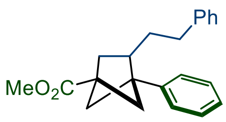

3a

Synthesized following **General Procedure A** using: BCB **1a** (37.7 mg, 0.200 mmol, 1.00 equiv) and but-3-en-1-ylbenzene (300 µL, 2.00 mmol, 10.0 equiv). Purified by flash column chromatography (SiO_2; 100:0 to 97:3 pentane:EtOAc) to afford **3a** (40.2 mg, 0.126 mmol, 63%) as a yellow oil.

TLC: R_f = 0.36 (97:3 pentane:EtOAc).

NMR Spectroscopy (*see spectra*):

^{1}**H NMR** (400 MHz, $CDCl_3$): δ_H 7.23 – 7.18 (m, 2H), 7.17 – 7.09 (m, 3H), 7.09 – 7.04 (m, 1H), 7.03 – 6.97 (m, 4H), 3.63 (s, 3H), 2.57 – 2.48 (m, 1H), 2.36 – 2.22 (m, 2H), 2.20 – 2.11 (m, 1H), 2.01 – 1.88 (m, 4H), 1.66 (ddd, *J* = 10.8, 3.9, 2.5 Hz, 1H), 1.55 (dddd, *J* = 13.2, 10.8, 6.6, 3.9 Hz, 1H), 1.42 – 1.31 (m, 1H) ppm;

^{13}**C NMR** (101 MHz, $CDCl_3$): δ_C 173.9, 142.4, 142.1, 128.5, 128.4, 128.3, 126.4, 126.1, 125.8, 54.5, 51.7, 48.7, 48.5, 45.1, 40.4, 37.7, 34.3, 33.8 ppm.

HRMS (ESI$^+$): m/z calc'd for $C_{22}H_{24}O_2Na$ $[M+Na]^+$: 343.1669, found: 343.1668.

Scale-up:

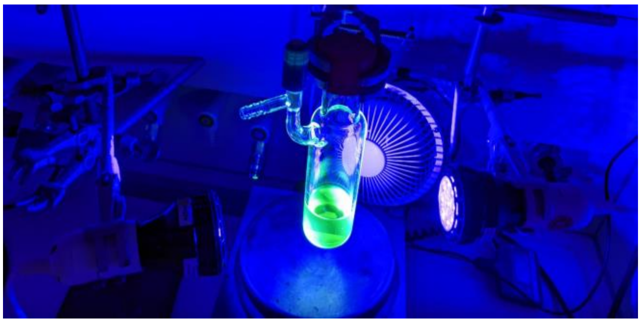

Figure S4: Scale-up reaction for substrate 2a

To an oven-dried 100 mL Schlenk tube equipped with a Teflon-coated magnetic stir bar was added $[Mes_2Acr^tBu_2]ClO_4$ (246 mg, 0.400 mmol, 10 mol%), **2a** (6.01 mL, 40.0 mmol, 10.0 equiv), and bicyclo[1.1.0]butane (BCB) **1a** (753 mg, 4.00 mmol, 1.00 equiv). The Schlenk tube was evacuated and backfilled with argon three times before $MeNO_2$ (20 mL) was added under a positive argon pressure. The reaction mixture was stirred under irradiation with blue LEDs (18 W, λ_{max} = 425 nm) for 16 h.[A] After this time, the solvent was removed under reduced pressure and the crude product was purified by flash column chromatography on silica gel to afford **3a** (675 mg, 2.11 mmol, 53%) as a colourless oil.

Note: (**A**) Fan cooling was used during irradiation.

Methyl-3-butyl-4-phenylbicyclo[2.1.1]hexane-1-carboxylate (3b)

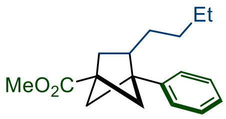

3b

Synthesized following **General Procedure A** using: BCB **1a** (37.7 mg, 0.200 mmol, 1.00 equiv) and hex-1-ene **2b** (255 µL, 2.00 mmol, 10.0 equiv). Purified by flash column chromatography (SiO_2; 100:0 to 97:3 pentane:EtOAc) to afford **3b** (24.1 mg, 0.0885 mmol, 44%) as a colourless oil.

TLC: R_f = 0.46 (97:3 pentane:EtOAc).

NMR Spectroscopy (*see spectra*):

^{1}**H NMR** (400 MHz, $CDCl_3$): δ_H 7.33 – 7.27 (m, 2H), 7.23 – 7.18 (m, 1H), 7.13 – 7.09 (m, 2H), 3.71 (s, 3H), 2.28 (ddd, *J* = 10.6, 8.5, 2.1 Hz, 1H), 2.22 – 2.14 (m, 1H), 2.07 – 1.95 (m, 4H), 1.66 (ddd, *J* = 10.7, 3.8, 2.4 Hz, 1H), 1.31 – 1.05 (m, 6H), 0.85 – 0.78 (m, 3H) ppm;

^{13}**C NMR** (101 MHz, $CDCl_3$): δ_C 174.1, 142.4, 128.3, 126.3, 126.1, 54.4, 51.7, 48.8, 48.5, 45.5, 40.3, 37.7, 31.3, 30.2, 23.0, 14.2 ppm.

HRMS (ESI^+): m/z calc'd for $C_{18}H_{24}O_2Na$ $[M+Na]^+$: 295.1669, found: 295.1668.

Methyl-3-methyl-4-phenylbicyclo[2.1.1]hexane-1-carboxylate (3c)

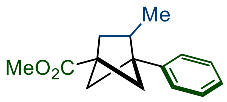

3c

Synthesized following a modified **General Procedure A** using: BCB **1a** (37.7 mg, 0.200 mmol, 1.00 equiv) and a 2 bar atmosphere of propene gas **2c**. Purified by flash column chromatography (SiO_2; 100:0 to 94:6 pentane:EtOAc) to afford **3c** (17.6 mg, 0.0764 mmol, 38%) as a pale pink oil.

TLC: R_f = 0.32 (95:5 pentane:EtOAc).

NMR Spectroscopy (*see spectra*):

^{1}**H NMR** (500 MHz, $CDCl_3$): δ_H 7.34 – 7.26 (m, 2H), 7.24 – 7.17 (m, 1H), 7.14 – 7.10 (m, 2H), 3.70 (s, 3H), 2.42 – 2.30 (m, 2H), 2.12 – 1.93 (m, 4H), 1.61 – 1.55 (m, 1H), 0.89 (d, *J* = 6.7 Hz, 3H) ppm;

^{13}**C NMR** (126 MHz, $CDCl_3$): δ_C 174.0, 142.2, 128.3, 126.3, 126.1, 54.6, 51.7, 48.7, 48.5, 39.7, 39.6, 39.4, 16.9 ppm.

HRMS (ESI+): m/z calc'd for $C_{15}H_{18}O_2Na$ [M+Na]+: 253.11990, found: 253.11978.

Methyl-3-(7-chloro-octyl)-4-phenylbicyclo[2.1.1]hexane-1-carboxylate (3d)

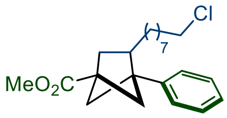

3d

Synthesized following **General Procedure A** using: BCB **1a** (37.7 mg, 0.200 mmol, 1.00 equiv) and 10-chlorodec-1-ene **2d** (349 mg, 2.00 mmol, 10.0 equiv). Purified by flash column chromatography (SiO_2; 100:0 to 97:3 pentane:EtOAc) to afford **3d** (28.8 mg, 0.0794 mmol, 40%) as a yellow oil.

TLC: R_f = 0.33 (98:2 pentane:EtOAc).

NMR Spectroscopy (*see spectra*):

> **^{1}H NMR** (400 MHz, $CDCl_3$): δ_H 7.33 – 7.27 (m, 2H), 7.23 – 7.18 (m, 1H), 7.13 – 7.08 (m, 2H), 3.71 (s, 3H), 3.51 (t, *J* = 6.7 Hz, 2H), 2.28 (ddd, *J* = 10.7, 8.4, 1.9 Hz, 1H), 2.22 – 2.13 (m, 1H), 2.07 – 1.94 (m, 4H), 1.73 (dt, *J* = 14.7, 6.7 Hz, 2H), 1.65 (ddd, *J* = 10.7, 3.9, 2.3 Hz, 1H), 1.43 – 1.05 (m, 12H) ppm;

> **^{13}C NMR** (101 MHz, $CDCl_3$): δ_C 174.0, 142.4, 128.3, 126.3, 126.1, 54.4, 51.7, 48.8, 48.5, 45.5, 45.3, 40.3, 37.7, 32.7, 31.7, 29.7, 29.5, 28.9, 28.0, 27.0 ppm.

HRMS (ESI+): m/z calc'd for $C_{22}H_{31}O_2Na$ [M+Na]+: 385.1905, found: 385.1904.

Methyl-3-(6-bromohexyl)-4-phenylbicyclo[2.1.1]hexane-1-carboxylate (3e)

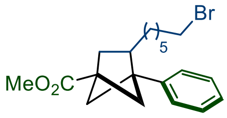

3e

Synthesized following **General Procedure A** using: BCB **1a** (37.7 mg, 0.200 mmol, 1.00 equiv) and 8-bromooct-1-ene **2e** (335 µL, 2.00 mmol, 10.0 equiv). Purified by flash column chromatography (SiO_2; 100:0 to 96:4 pentane:EtOAc) to afford **3e** (48.1 mg, 0.127 mmol, 63%) as a colourless oil.

TLC: R_f = 0.47 (96:4 pentane:EtOAc).

NMR Spectroscopy (*see spectra*):

> **^{1}H NMR** (500 MHz, $CDCl_3$): δ_H 7.30 (t, *J* = 7.6 Hz, 2H), 7.24 – 7.18 (m, 1H), 7.10 (dd, *J* = 8.2, 1.3 Hz, 2H), 3.70 (s, 3H), 3.36 (t, *J* = 6.9 Hz, 2H), 2.28 (ddd, *J* = 10.7, 8.5, 2.1 Hz, 1H), 2.23 – 2.12 (m, 1H), 2.06 – 1.94 (m, 4H), 1.78 (h, *J* = 7.0 Hz, 2H), 1.64 (ddd, *J* = 10.9, 4.0, 2.6 Hz, 1H), 1.42 – 1.17 (m, 6H), 1.16 – 1.06 (m, 2H) ppm;

^{13}C NMR (126 MHz, $CDCl_3$): δ_C 174.0, 142.3, 128.3, 126.3, 126.1, 54.4, 51.7, 48.7, 48.5, 45.5, 40.3, 37.7, 34.0, 32.8, 31.6, 29.0, 28.2, 27.8 ppm.

HRMS (ESI^+): m/z calc'd for $C_{20}H_{27}O_2BrNa$ $[M+Na]^+$: 401.10866, found: 401.10868.

Methyl-3-(dec-9-yn-1-yl)-4-phenylbicyclo[2.1.1]hexane-1-carboxylate (3f)

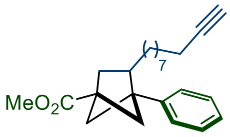

3f

Synthesized following **General Procedure A** using: BCB **1a** (37.7 mg, 0.200 mmol, 1.00 equiv) and dodec-1-en-11-yne **2f** (230 mg, 1.40 mmol, 7.00 equiv). Purified by flash column chromatography (SiO_2; 100:0 to 96:4 pentane:EtOAc) to afford **3f** (16.4 mg, 0.0465 mmol, 23%) as a yellow oil.

TLC: R_f = 0.45 (95:5 pentane:EtOAc).

NMR Spectroscopy (*see spectra*):

^{1}H NMR (400 MHz, $CDCl_3$): δ_H 7.33 – 7.27 (m, 2H), 7.23 – 7.18 (m, 1H), 7.13 – 7.08 (m, 2H), 3.70 (s, 3H), 2.32 – 2.23 (m, 1H), 2.16 (td, *J* = 7.1, 2.7 Hz, 2H), 2.07 – 1.94 (m, 4H), 1.93 (t, *J* = 2.7 Hz, 1H), 1.68 – 1.62 (m, 1H), 1.53 – 1.44 (m, 2H), 1.39 – 1.05 (m, 13H) ppm;

^{13}C NMR (101 MHz, $CDCl_3$): δ_C 174.1, 142.4, 128.3, 126.3, 126.1, 84.9, 68.2, 54.4, 51.7, 48.8, 48.5, 45.5, 40.3, 37.7, 31.7, 29.8, 29.6, 29.1, 28.8, 28.6, 28.0, 18.5 ppm.

HRMS (ESI^+): m/z calc'd for $C_{24}H_{32}O_2Na$ $[M+Na]^+$: 375.2295, found: 375.2293.

Methyl-3-(3-oxobutyl)-4-phenylbicyclo[2.1.1]hexane-1-carboxylate (3g)

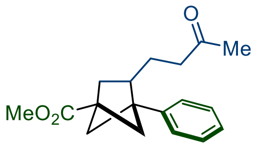

3g

Synthesized following **General Procedure A** using: BCB **1a** (37.7 mg, 0.200 mmol, 1.00 equiv) and hex-5-en-2-one **2g** (232 µL, 2.00 mmol, 10.0 equiv). Purified by flash column chromatography (SiO_2; 100:0 to 80:20 pentane:EtOAc) to afford **3g** (24.2 mg, 0.0845 mmol, 42%) as an orange oil.

TLC: R_f = 0.51 (80:20 pentane:EtOAc).

NMR Spectroscopy (*see spectra*):

^{1}H NMR (500 MHz, $CDCl_3$): δ_H 7.30 (dd, *J* = 8.3, 7.0 Hz, 2H), 7.23 – 7.18 (m, 1H), 7.14 – 7.09 (m, 2H),

3.70 (s, 3H), 2.38 – 2.25 (m, 2H), 2.25 – 2.13 (m, 2H), 2.09 – 1.95 (m, 7H), 1.67 – 1.52 (m, 2H), 1.48 – 1.37 (m, 1H) ppm;

^{13}C NMR (126 MHz, $CDCl_3$): δ_C 208.8, 173.7, 141.8, 128.4, 126.5, 126.1, 54.4, 51.7, 48.8, 48.4, 44.7, 42.1, 40.1, 37.5, 30.0, 26.0 ppm.

HRMS (ESI$^+$): m/z calc'd for $C_{18}H_{22}O_3Na$ [M+Na]$^+$: 309.14612, found: 309.14612.

Methyl-3-(butoxymethyl)-4-phenylbicyclo[2.1.1]hexane-1-carboxylate (3h)

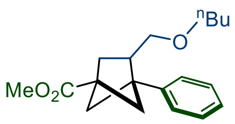

3h

Synthesized following **General Procedure A** using: BCB **1a** (37.7 mg, 0.200 mmol, 1.00 equiv) and 1-(allyloxy)butane **2h** (228 µL, 2.00 mmol, 8.00 equiv). Purified by flash column chromatography (SiO_2; 100:0 to 92:8 pentane:EtOAc) to afford **3h** (15.7 mg, 0.0519 mmol, 26%) as a colourless oil.

TLC: R_f = 0.46 (95:5 pentane:EtOAc).

NMR Spectroscopy (*see spectra*):

^{1}H NMR (400 MHz, $CDCl_3$): δ_H 7.32 – 7.27 (m, 2H), 7.23 – 7.18 (m, 1H), 7.15 – 7.11 (m, 2H), 3.71 (s, 3H), 3.37 – 3.20 (m, 4H), 2.57 – 2.46 (m, 1H), 2.31 (ddd, J = 11.2, 8.7, 2.5 Hz, 1H), 2.18 – 2.10 (m, 1H), 2.07 – 1.99 (m, 3H), 1.93 (ddd, J = 11.2, 4.2, 2.2 Hz, 1H), 1.49 – 1.38 (m, 2H), 1.33 – 1.22 (m, 2H), 0.86 (t, J = 7.3 Hz, 3H) ppm;

^{13}C NMR (101 MHz, $CDCl_3$): δ_C 173.8, 141.9, 128.4, 126.5, 126.0, 72.5, 71.1, 53.3, 51.7, 49.1, 48.4, 45.0, 40.7, 35.8, 31.9, 19.5, 14.0 ppm.

HRMS (ESI$^+$): m/z calc'd for $C_{19}H_{26}O_3Na$ [M+Na]$^+$: 325.1774, found: 325.1773.

Methyl-3-(7-((2-methylbenzoyl)oxy)heptyl)-4-phenylbicyclo[2.1.1]hexane-1-carboxylate (3i)

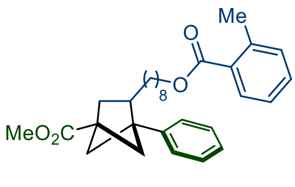

3i

Synthesized following **General Procedure A** using: BCB **1a** (37.7 mg, 0.200 mmol, 1.00 equiv) and dec-9-en-1-yl 2-methylbenzoate **2i** (549 mg, 2.00 mmol, 10.0 equiv). Purified by flash column chromatography (SiO_2; 100:0 to 92:8 pentane:EtOAc) to afford **3i** (43.6 mg, 0.0942 mmol, 47%) as a yellow oil.

TLC: R_f = 0.40 (95:5 pentane:EtOAc).

NMR Spectroscopy (*see spectra*):

^{1}**H NMR** (400 MHz, $CDCl_3$): δ_H 7.92 – 7.88 (m, 1H), 7.42 – 7.36 (m, 1H), 7.33 – 7.27 (m, 2H), 7.26 – 7.18 (m, 3H), 7.14 – 7.08 (m, 2H), 4.28 (t, *J* = 6.7 Hz, 2H), 3.71 (s, 3H), 2.60 (s, 3H), 2.32 – 2.25 (m, 1H), 2.22 – 2.14 (m, 1H), 2.07 – 1.95 (m, 4H), 1.78 – 1.69 (m, 2H), 1.66 (ddd, *J* = 10.7, 3.9, 2.4 Hz, 1H), 1.44 – 1.06 (m, 12H) ppm;

^{13}**C NMR** (101 MHz, $CDCl_3$): δ_C 174.0, 167.9, 142.3, 140.1, 131.9, 131.8, 130.6, 130.1, 128.3, 126.3, 126.1, 125.8, 65.0, 54.4, 51.6, 48.7, 48.5, 45.5, 40.3, 37.7, 31.7, 29.8, 29.6, 29.3, 28.8, 28.0, 26.2, 21.9 ppm.

HRMS (ESI$^+$): m/z calc'd for $C_{30}H_{38}O_4Na$ $[M+Na]^+$: 485.2662, found: 485.2663.

Methyl-4-phenyl-3-(8-((3-phenylpropioloyl)oxy)octyl)bicyclo[2.1.1]hexane-1-carboxylate (3j)

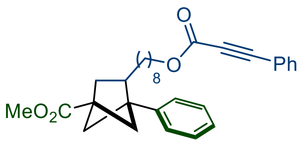

3j

Synthesized following **General Procedure A** using: BCB **1a** (37.7 mg, 0.200 mmol, 1.00 equiv) and dec-9-en-1-yl 3-phenylpropiolate **2j** (585 mg, 2.00 mmol, 10.0 equiv). Purified by flash column chromatography (SiO_2; 100:0 to 95:5 pentane:EtOAc) to afford **3j** (40.3 mg, 0.0855 mmol, 43%) as a colourless solid.

TLC: R_f = 0.40 (95:5 pentane:EtOAc).

NMR Spectroscopy (*see spectra*):

^{1}**H NMR** (600 MHz, $CDCl_3$): δ_H 7.61 – 7.57 (m, 2H), 7.47 – 7.42 (m, 1H), 7.40 – 7.35 (m, 2H), 7.32 – 7.27 (m, 2H), 7.20 (ddt, J = 7.8, 6.8, 1.3 Hz, 1H), 7.13 – 7.09 (m, 2H), 4.21 (t, J = 6.8 Hz, 2H), 3.70 (s, 3H), 2.28 (ddd, J = 10.8, 8.5, 2.2 Hz, 1H), 2.17 (t, J = 9.8 Hz, 1H), 2.06 – 1.92 (m, 4H), 1.72 – 1.61 (m, 3H), 1.39 – 1.32 (m, 2H), 1.30 – 1.17 (m, 8H). 1.09 (t, J = 8.1 Hz, 2H) ppm;

^{13}**C NMR** (151 MHz, $CDCl_3$): δ_C 174.0, 154.4, 142.4, 133.1, 130.7, 128.7, 128.3, 126.3, 126.1, 119.8, 86.2, 80.9, 66.4, 54.4, 51.7, 48.8, 48.5, 45.5, 40.3, 37.7, 31.7, 29.8, 29.6, 29.3, 28.6, 28.0, 25.9 ppm.

HRMS (ESI$^+$): m/z calc'd for $C_{31}H_{36}O_4Na$ $[M+Na]^+$: 495.25058, found: 495.25055.

Methyl-4-(methoxycarbonyl)-1-phenylbicyclo[2.1.1]hexan-2-yl)octyl thiophene-2-carboxylate (3k)

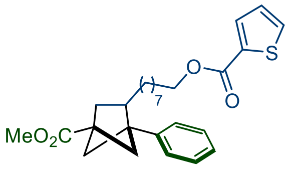

3k

Synthesized following **General Procedure A** using: BCB **1a** (37.7 mg, 0.200 mmol, 1.00 equiv) and dec-9-en-1-yl thiophene-2-carboxylate **2k** (533 mg, 2.00 mmol, 10.0 equiv). Purified by flash column chromatography (SiO_2; 100:0 to 94:6 pentane:EtOAc) to afford **3k** (44.5 mg, 0.0979 mmol, 49%) as a yellow oil.

TLC: R_f = 0.38 (92:8 pentane:EtOAc).

NMR Spectroscopy (*see spectra*):

^{1}**H NMR** (400 MHz, $CDCl_3$): δ_H 7.81 – 7.77 (m, 1H), 7.56 – 7.51 (m, 1H), 7.33 – 7.27 (m, 2H), 7.23 – 7.17 (m, 1H), 7.13 – 7.07 (m, 3H), 4.27 (td, *J* = 6.7, 1.5 Hz, 2H), 3.70 (s, 3H), 2.31 – 2.24 (m, 1H), 2.22 – 2.13 (m, 1H), 2.08 – 1.94 (m, 4H), 1.76 – 1.62 (m, 3H), 1.42 – 1.06 (m, 12H) ppm;

^{13}**C NMR** (101 MHz, $CDCl_3$): δ_C 174.01, 162.4, 142.3, 134.3, 133.3, 132.3, 128.3, 127.8, 126.3, 126.1, 65.4, 54.4, 51.6, 48.7, 48.5, 45.5, 40.3, 37.7, 31.7, 29.8, 29.6, 29.3, 28.8, 28.0, 26.0 ppm.

HRMS (ESI^+): m/z calc'd for $C_{27}H_{34}O_4SNa$ $[M+Na]^+$: 477.2070, found: 477.2070.

Methyl-4-phenyl-3-(4-tosylbutyl)bicyclo[2.1.1]hexane-1-carboxylate (3l)

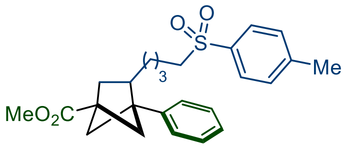

3l

Synthesized following **General Procedure A** using: BCB **1a** (37.7 mg, 0.200 mmol, 1.00 equiv) and 1-(hex-5-en-1-ylsulfonyl)-4-methylbenzene **2l** (477 mg, 2.00 mmol, 10.0 equiv). Purified by flash column chromatography (SiO_2; 90:10 to 75:25 pentane:EtOAc) to afford **3l** (34.1 mg, 0.0799 mmol, 40%) as a colourless oil.

TLC: R_f = 0.52 (75:25 pentane:EtOAc).

NMR Spectroscopy (*see spectra*):

^{1}**H NMR** (500 MHz, $CDCl_3$): δ_H 7.73 (d, *J* = 8.3 Hz, 2H), 7.33 (d, *J* = 7.9 Hz, 2H), 7.29 (t, *J* = 7.5 Hz, 2H), 7.22 – 7.18 (m, 1H), 7.10 – 7.03 (m, 2H), 3.69 (s, 3H), 2.95 (t, *J* = 8.0 Hz, 2H), 2.44 (s, 3H), 2.24 (ddd, *J* = 10.9, 8.5, 2.4 Hz, 1H), 2.16 – 2.08 (m, 1H), 2.03 – 1.94 (m, 4H), 1.64 – 1.52 (m, 3H), 1.38 – 1.28 (m, 1H), 1.27 – 1.19 (m, 1H), 1.16 – 1.10 (m, 1H), 1.10 – 1.04 (m, 1H) ppm;

^{13}C NMR (126 MHz, $CDCl_3$): δ_C 173.7, 144.7, 142.0, 136.3, 130.0, 128.4, 128.2, 126.5, 126.0, 56.4, 54.4, 51.7, 48.7, 48.5, 45.1, 40.2, 37.6, 31.2, 26.8, 23.0, 21.7 ppm.

HRMS (ESI$^+$): m/z calc'd for $C_{25}H_{30}O_4SNa$ $[M+Na]^+$: 449.17570, found: 449.17560.

4-(Methoxycarbonyl)-1-phenylbicyclo[2.1.1]hexan-2-yl)octyl 2-phenylquinoline-4-carboxylate (3m)

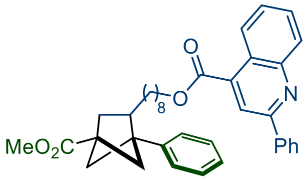

3m

Synthesized following **General Procedure A** using: BCB **1a** (37.7 mg, 0.200 mmol, 1.00 equiv) and dec-9-en-1-yl-2-phenylquinoline-4-carboxylate **2m** (775 mg, 2.00 mmol, 10.0 equiv). Purified by flash column chromatography (SiO_2; 100:0 to 92:8 pentane:EtOAc) to afford **3m** (40.4 mg, 0.0702 mmol, 35%) as a colourless oil.

TLC: R_f = 0.54 (92:8 pentane:EtOAc).

NMR Spectroscopy (*see spectra*):

^{1}H NMR (500 MHz, $CDCl_3$): δ_H 8.73 (d, *J* = 8.5 Hz, 1H), 8.38 (s, 1H), 8.27 – 8.16 (m, 3H), 7.77 (ddd, *J* = 8.5, 6.8, 1.4 Hz, 1H), 7.65 – 7.60 (m, 1H), 7.58 – 7.53 (m, 2H), 7.52 – 7.46 (m, 1H), 7.32 – 7.25 (m, 2H), 7.22 – 7.16 (m, 1H), 7.14 – 7.06 (m, 2H), 4.46 (t, *J* = 6.8 Hz, 2H), 3.70 (s, 3H), 2.27 (ddd, *J* = 10.7, 8.5, 2.0 Hz, 1H), 2.20 – 2.14 (m, 1H), 2.06 – 1.95 (m, 4H), 1.84 (p, *J* = 6.8 Hz, 2H), 1.64 (ddd, *J* = 10.7, 3.9, 2.6 Hz, 1H), 1.45 (p, *J* = 7.1 Hz, 2H), 1.37 – 1.19 (m, 8H), 1.15 – 1.06 (m, 2H) ppm;

^{13}C NMR (126 MHz, $CDCl_3$): δ_C 174.0, 166.7, 156.9, 149.4, 142.4, 139.0, 136.3, 130.5, 130.0, 129.9, 129.1, 128.3, 127.9, 127.6, 126.3, 126.1, 125.6, 124.2, 120.3, 66.2, 54.4, 51.7, 48.8, 48.5, 45.5, 40.3, 37.7, 31.7, 29.8, 29.6, 29.3, 28.8, 28.0, 26.2 ppm.

HRMS (ESI$^+$): m/z calc'd for $C_{38}H_{41}NO_4Na$ $[M+Na]^+$: 598.29338, found: 598.29278.

Methyl-3-(4-(1,3-dioxoisoindolin-2-yl)butyl)-4-phenylbicyclo[2.1.1]hexane-1-carboxylate (3n)

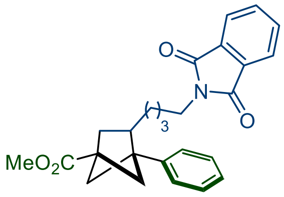

3n

Synthesized following **General Procedure A** using: BCB **1a** (37.7 mg, 0.200 mmol, 1.00 equiv) and 2-(hex-5-

en-1-yl)isoindoline-1,3-dione **2n** (460 mg, 2.00 mmol, 1.00 equiv). Purified by flash column chromatography (SiO_2; 100:0 to 80:20 pentane:EtOAc) to afford **3n** (37.2 mg, 0.0891 mmol, 45%) as a yellow oil.

TLC: R_f = 0.27 (85:15 pentane:EtOAc).

NMR Spectroscopy (*see spectra*):

^{1}**H NMR** (400 MHz, $CDCl_3$): δ_H 7.84 – 7.79 (m, 2H), 7.72 – 7.67 (m, 2H), 7.28 – 7.22 (m, 2H), 7.16 – 7.10 (m, 1H), 7.10 – 7.05 (m, 2H), 3.69 (s, 3H), 3.59 (t, *J* = 7.2 Hz, 2H), 2.31 – 2.22 (m, 1H), 2.23 – 2.13 (m, 1H), 2.05 – 1.93 (m, 4H), 1.66 – 1.49 (m, 3H), 1.38 – 1.21 (m, 2H), 1.21 – 1.08 (m, 2H) ppm;

^{13}**C NMR** (101 MHz, $CDCl_3$): δ_C 173.6, 168.5, 142.1, 133.9, 132.3, 128.3, 126.3, 126.0, 123.3, 54.4, 51.6, 48.7, 48.4, 45.3, 40.2, 38.1, 37.6, 31.3, 28.8, 25.3 ppm.

HRMS (ESI^+): m/z calc'd for $C_{26}H_{27}NO_4Na$ $[M+Na]^+$: 440.1832, found: 440.1832.

Methyl-3-(8-(((S)-2-(1,3-dioxoisoindolin-2-yl)-3-phenylpropanoyl)oxy)octyl)-4-phenylbicyclo[2.1.1]hexane-1-carboxylate (3o)

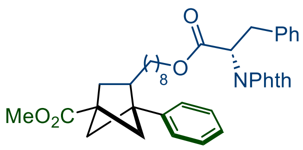

3o

Synthesized following **General Procedure A** using: BCB **1a** (37.7 mg, 0.200 mmol, 1.00 equiv) and dec-9-en-1-yl (S)-2-(1,3-dioxoisoindolin-2-yl)-3-phenylpropanoate **2o** (867 mg, 2.00 mmol, 10.0 equiv). Purified by flash column chromatography (SiO_2; 100:0 to 80:20 pentane:EtOAc) to afford **3o** as a 1:1 mix of diastereomers (46.4 mg, 0.0746 mmol, 37%) as a yellow oil.

TLC: R_f = 0.40 (85:15 pentane:EtOAc).

NMR Spectroscopy (*see spectra*):

^{1}**H NMR** (400 MHz, $CDCl_3$): δ_H 7.79 – 7.73 (m, 2H), 7.68 – 7.62 (m, 2H), 7.33 – 7.27 (m, 2H), 7.23 – 7.09 (m, 8H), 5.14 (dd, *J* = 11.1, 5.4 Hz, 1H), 4.23 – 4.08 (m, 2H), 3.70 (s, 3H), 3.64 – 3.49 (m, 2H), 2.33 – 2.23 (m, 1H), 2.21 – 2.12 (m, 1H), 2.08 – 1.93 (m, 4H), 1.69 – 1.51 (m, 3H), 1.32 – 1.02 (m, 12H) ppm;

^{13}**C NMR** (101 MHz, $CDCl_3$): δ_C 174.0, 169.0, 167.6, 142.3, 137.0, 134.2, 131.7, 128.9, 128.6, 128.3, 126.9, 126.3, 126.1, 123.5, 66.2, 54.4, 53.5, 51.6, 48.7, 48.4, 45.5, 40.3, 37.7, 34.8, 31.6, 29.7, 29.5, 29.1, 28.5, 28.0, 25.8 ppm.

HRMS (ESI^+): m/z calc'd for $C_{39}H_{43}NO_6Na$ $[M+Na]^+$: 644.2983, found: 644.2983.

Methyl-3-phenylhexahydro-1,3-methanopentalene-1(2H)-carboxylate (3p)

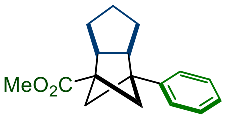

3p

Synthesized following **General Procedure A** using: BCB **1a** (37.7 mg, 0.200 mmol, 1.00 equiv) and cyclopentene **2p** (177 µL, 2.00 mmol, 10.0 equiv). Purified by flash column chromatography (SiO_2; 100:0 to 96:4 pentane:EtOAc) to afford **3p** as a single diastereomer (11.6 mg, 0.0453 mmol, 23%) as a colourless oil.

TLC: R_f = 0.42 (97:3 pentane:EtOAc).

NMR Spectroscopy (*see spectra*):

^{1}**H NMR** (400 MHz, $CDCl_3$): δ_H 7.33 – 7.27 (m, 2H), 7.24 – 7.18 (m, 3H), 3.70 (s, 3H), 2.98 – 2.78 (m, 2H), 2.25 – 2.21 (m, 1H), 2.15 – 2.04 (m, 2H), 2.04 – 1.99 (m, 1H), 1.98 – 1.83 (m, 2H), 1.74 – 1.55 (m, 2H), 1.38 – 1.25 (m, 1H), 1.21 – 1.10 (m, 1H) ppm;

^{13}**C NMR** (101 MHz, $CDCl_3$): δ_C 173.9, 142.4, 128.3, 126.4, 126.4, 53.5, 53.4, 52.0, 51.6, 51.4, 50.0, 37.1, 30.5, 28.4, 28.1 ppm.

HRMS (ESI$^+$): m/z calc'd for $C_{17}H_{20}O_2Na$ $[M+Na]^+$: 222.0925, found: 222.0926.

Methyl-3-phenyloctahydro-1H-1,3-methanoindene-1-carboxylate (3q)

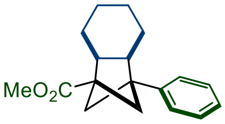

3q

Synthesized following **General Procedure A** using: BCB **1a** (37.7 mg, 0.200 mmol, 1.00 equiv) and cyclohexene **2q** (202 µL, 2.00 mmol, 10.0 equiv). Purified by flash column chromatography (SiO_2; 100:0 to 96:4 pentane:EtOAc) to afford **3q** as a single diastereomer (24.3 mg, 0.0899 mmol, 45%) as a colourless oil.

TLC: R_f = 0.44 (97:3 pentane:EtOAc).

NMR Spectroscopy (*see spectra*):

^{1}**H NMR** (400 MHz, $CDCl_3$): δ_H 7.32 – 7.26 (m, 2H), 7.22 – 7.17 (m, 1H), 7.15 – 7.10 (m, 2H), 3.70 (s, 3H), 2.47 – 2.37 (m, 1H), 2.23 – 2.14 (m, 2H), 2.03 – 1.96 (m, 2H), 1.92 (dt, J = 7.3, 1.5 Hz, 1H), 1.78 – 1.56 (m, 3H), 1.48 – 1.19 (m, 4H), 1.18 – 1.04 (m, 1H) ppm;

^{13}**C NMR** (101 MHz, $CDCl_3$): δ_C 173.8, 142.3, 128.3, 126.3, 126.1, 54.2, 52.4, 51.5, 49.3, 44.6, 42.6, 35.8, 21.2, 20.8, 19.1, 19.0 ppm.

HRMS (ESI^+): m/z calc'd for $C_{18}H_{22}O_2Na$ $[M+Na]^+$: 293.1512, found: 293.1511.

Methyl-3-phenyldecahydro-1H-1,3-methanocyclopenta[8]annulene-1-carboxylate (3r)

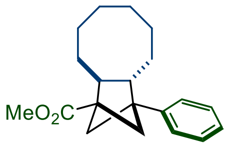

3r

Synthesized following **General Procedure A** using: BCB **1a** (37.7 mg, 0.200 mmol, 1.00 equiv) and (Z)-cyclooctene **2r** (257 µL, 2.00 mmol, 10.0 equiv). Purified by flash column chromatography (SiO_2; 100:0 to 97:3 pentane:EtOAc) to afford **3r** as a 5:1 mixture of diastereomers (15.8 mg, 0.0529 mmol, 26%) as a colourless oil.

TLC: R_f = 0.35 (98:2 pentane:EtOAc).

NMR Spectroscopy (*see spectra*): *in cases where diastereomer peaks could be distinguished, they are labelled as d^1 and d^2.*

^{1}H NMR (400 MHz, $CDCl_3$): δ_H; 7.33 – 7.27 (m, 2H), 7.22 – 7.17 (m, 1H), 7.13 – 7.07 (m, 2H), 3.71 (s, 2.5H, d^1), 3.70 (s, 0.5H, d^2), 2.44 (ddt, J = 10.3, 8.5, 1.7 Hz, 0.84H, d^1), 2.36 – 2.30 (m, 0.16H, d^2), 2.17 (ddt, J = 10.4, 8.5, 1.7 Hz, 0.84H, d^1), 2.08 (t, J = 5.3 Hz, 0.32H, d^2), 2.06 – 2.01 (m, 1H), 1.99 – 1.94 (m, 1H), 1.93 – 1.89 (m, 1H), 1.89 – 1.86 (m, 0.84H, d^1), 1.79 – 1.22 (m, 11H), 1.15 – 1.05 (m, 1H) ppm.

^{13}C NMR (101 MHz, $CDCl_3$): δ_C (*d^1, major diastereomer)* 174.1, 142.8, 128.3, 126.6, 126.3, 54.7, 53.5, 51.6, 50.8, 48.9, 47.2, 35.7, 30.8, 30.7, 26.4, 26.3, 25.4, 23.5 ppm. δ_C (*d^2, minor diastereomer)* 173.9, 142.4, 182.2, 126.2, 126.1, 53.6, 52.0, 51.5, 50.3, 48.7, 43.4, 43.0, 31.3, 30.9, 27.7, 27.6, 25.1, 25.0 ppm.

HRMS (ESI^+): m/z calc'd for $C_{20}H_{26}O_2Na$ $[M+Na]^+$: 321.1825, found: 321.1824.

Methyl-3-butyl-2-ethyl-4-phenylbicyclo[2.1.1]hexane-1-carboxylate (3s)

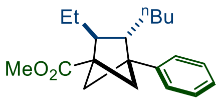

3s

Synthesized following **General Procedure A** using: BCB **1a** (37.7 mg, 0.200 mmol, 1.00 equiv) and (E)-oct-3-ene **2s** (314 µL, 2.00 mmol, 10.0 equiv). Purified by flash column chromatography (SiO_2; 100:0 to 96:4 pentane:EtOAc) to afford a single diastereomer of **3s** as a 1:1 mix of regioisomers (17.9 mg, 0.0596 mmol, 30%) as a yellow oil.

TLC: R_f = 0.32 (96:4 pentane:EtOAc).

NMR Spectroscopy (see spectra): *in cases where regioisomeric peaks could be distinguished, they are labelled as r^1 and r^2. ^{13}C signals relating to the same carbon are given together.*

> **^{1}H NMR** (400 MHz, $CDCl_3$): 7.30 – 7.26 (m, 2H), 7.21 – 7.16 (m, 1H), 7.11 – 7.06 (m, 2H), 3.69 (s, 3H), 2.19 – 2.09 (m, 2H), 2.03 – 1.96 (m, 1.5H), 1.96 – 1.88 (m, 1.5H), 1.71 – 1.65 (m, 0.5H), 1.65 – 1.58 (m, 1H), 1.53 – 1.48 (m, 0.5H), 1.48 – 1.41 (m, 0.5H), 1.38 – 1.13 (m, 6.5H), 0.94 (t, *J* = 7.5 Hz, 1.5H, r^1), 0.90 (t, *J* = 7.0 Hz, 1.5H, r^2), 0.77 (t, *J* = 7.1 Hz, 1.5H, r^1), 0.72 (t, *J* = 7.5 Hz, 1.5H, r^2) ppm;
>
> **^{13}C NMR** (101 MHz, $CDCl_3$): δ_C 174.5 and 174.4, 142.6 and 142.6, 128.2 and 128.2, 126.2, 126.1, 54.7 and 51.5, 53.3 and 51.4, 53.2 and 51.3, 52.8 and 52.5, 51.5 and 50.7, 43.8 and 43.5, 43.7 and 43.6, 33.2 and 30.7, 32.2 and 30.6, 26.1 and 23.2, 25.3 and 23.3, 14.2 and 13.0, 14.1 and 13.1 ppm

HRMS (ESI$^+$): m/z calc'd for $C_{20}H_{28}O_2Na$ $[M+Na]^+$: 323.1982, found: 323.1981.

Methyl-2-ethyl-3,3-dimethyl-4-phenylbicyclo[2.1.1]hexane-1-carboxylate (3t)

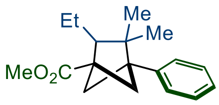

3t

Synthesized following **General Procedure A** using: BCB **1a** (37.7 mg, 0.200 mmol, 1.00 equiv) and 2-methylpent-2-ene **2t** (244 µL, 2.00 mmol, 10.0 equiv). Purified by flash column chromatography (SiO_2; 100:0 to 95:5 pentane:EtOAc) to afford **3t** (17.1 mg, 0.0628 mmol, 31%) as a colourless oil.

TLC: R_f = 0.45 (95:5 pentane:EtOAc).

NMR Spectroscopy (see spectra):

> **^{1}H NMR** (500 MHz, $CDCl_3$): δ_H 7.31 – 7.26 (m, 2H), 7.23 – 7.19 (m, 1H), 7.08 – 7.02 (m, 2H), 3.68 (s, 3H), 2.38 (dd, *J* = 9.6, 6.5 Hz, 1H), 2.32 (dd, *J* = 9.6, 7.1 Hz, 1H), 2.03 (ddd, *J* = 7.8, 6.3, 2.0 Hz, 1H), 1.87 – 1.81 (m, 2H), 1.58 – 1.50 (m, 1H), 1.44 – 1.36 (m, 1H), 1.03 (s, 3H), 0.95 (t, *J* = 7.4 Hz, 3H), 0.87 (s, 3H) ppm;
>
> **^{13}C NMR** (126 MHz, $CDCl_3$): δ_C 174.5, 141.3, 127.7, 127.2, 126.2, 57.0, 55.6, 51.5, 51.4, 45.1, 43.2, 39.6, 26.7, 21.8, 19.7, 14.1 ppm.

HRMS (ESI$^+$): m/z calc'd for $C_{18}H_{24}O_2Na$ $[M+Na]^+$: 295.16685, found: 295.16685.

Methyl-3-(2-((2-(4-isobutylphenyl)propanoyl)oxy)ethyl)-4-phenylbicyclo[2.1.1]hexane-1-carboxylate (3u)

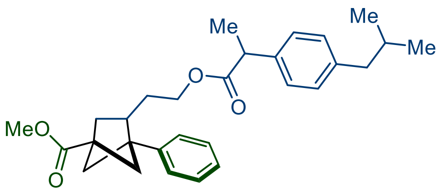

3u

Synthesized following **General Procedure A** using: BCB **1a** (37.7 mg, 0.200 mmol, 1.00 equiv) and ibuprofen derivative **2u** (521 mg, 2.00 mmol, 10.0 equiv). Purified by flash column chromatography (SiO_2; 100:0 to 85:15 pentane:EtOAc) to afford **3u** as a 1:1 mix of diastereomers (33.8 mg, 0.0754 mmol, 38%) as a colourless oil.

TLC: R_f = 0.46 (85:15 pentane:EtOAc).

NMR Spectroscopy (*see spectra*): *'2×' indicates where individual diastereomer peaks are non-coincident. ^{13}C signals relating to the same carbon are given together.*

^{1}H NMR (500 MHz, $CDCl_3$): δ_H 7.30 – 7.26 (m, 2H), 7.22 – 7.19 (m, 1H), 7.19 – 7.16 (m, 2H), 7.12 – 7.06 (m, 2H), 7.05 – 7.00 (m, 2H), 3.99 – 3.92 (m, 2H), 3.70 (2×s, 3H), 3.67 – 3.61 (m, 1H), 2.45 (2×d, *J* = 7.2 2H), 2.23 – 2.09 (m, 2H), 2.05 – 2.01 (m, 1H), 2.00 – 1.94 (m, 2H), 1.92 – 1.81 (m, 2H), 1.69 – 1.63 (m, 1H), 1.62 – 1.52 (m, 1H), 1.46 (2×d, *J* = 7.2 Hz, 3H), 1.43 – 1.34 (m, 1H), 0.92 – 0.88 (m, 6H) ppm;

^{13}C NMR (126 MHz, $CDCl_3$): δ_C 174.8 and 174.7, 173.6 and 173.6, 141.6 and 141.6, 140.7 and 140.6, 138.0 and 137.9, 129.4 and 129.4, 128.4 and 128.4, 127.3 and 127.3, 126.6 and 126.6, 126.1 and 126.0, 63.7 and 63.4, 54.2 and 54.2, 51.7, 48.7 and 48.6, 48.5, 45.3 and 45.3, 45.2 and 45.2, 42.0 and 41.8, 40.1 and 40.0, 37.5 and 37.4, 30.8 and 30.7, 30.3, 22.6, 22.5 and 22.5, 18.5 and 18.5 ppm.

HRMS (ESI$^+$): m/z calc'd for $C_{29}H_{36}O_4Na$ $[M+Na]^+$: 471.25058, found: 471.25012.

Methyl-4-(methoxycarbonyl)-1-phenylbicyclo[2.1.1]hexan-2-yl)ethyl (2S,5R)-3,3-dimethyl-7-oxo-4-thia-1-azabicyclo[3.2.0]heptane-2-carboxylate 4,4-dioxide (3v)

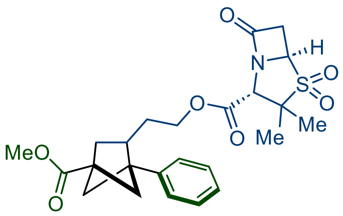

3v

Synthesized following **General Procedure A** using: BCB **1a** (37.7 mg, 0.200 mmol, 1.00 equiv) and sulbactam derivative **2v** (575 mg, 2.00 mmol, 10.0 equiv). Purified by flash column chromatography (SiO_2; 100:0 to 60:40 pentane:EtOAc) to afford **3v** as a 1:1 mix of diastereomers (27.5 mg, 0.0504 mmol, 25%) as a colourless oil.

TLC: R_f = 0.39 (60:40 pentane:EtOAc).

NMR Spectroscopy (see spectra): *'2×' indicates where individual diastereomer peaks are non-coincident. ^{13}C signals relating to the same carbon are given together.*

^{1}H NMR (500 MHz, $CDCl_3$): δ_H 7.34 – 7.28 (m, 2H), 7.25 – 7.20 (m, 1H), 7.12 – 7.07 (m, 2H), 4.57 (t, *J* = 4.4 Hz, 1H), 4.32 (2×s, 1H), 4.18 – 4.08 (m, 1H), 4.07 – 3.95 (m, 1H), 3.71 (s, 3H), 3.53 – 3.36 (m, 2H), 2.41 – 2.22 (m, 2H), 2.16 – 1.97 (m, 4H), 1.76 – 1.63 (m, 2H), 1.59 – 1.49 (m, 4H), 1.33 (2×s, 3H) ppm;

^{13}C NMR (126 MHz, $CDCl_3$): δ_C 173.3 and 173.3, 170.8 and 170.9, 167.0 and 167.0, 141.3 and 141.3, 128.6 and 128.6, 126.9, 125.9, 65.5 and 65.4, 63.4 and 63.3, 62.8 and 62.8, 61.2, 54.4 and 54.4, 51.8, 48.6 and 48.6, 48.5 and 48.5, 42.1 and 42.1, 40.1 and 40.1, 38.5 and 38.4, 37.6 and 37.5, 31.0 and 30.8, 20.5 and 20.4, 18.8 ppm.

HRMS (ESI$^+$): m/z calc'd for $C_{24}H_{29}NO_4SNa$ $[M+Na]^+$: 498.15569, found: 498.15563.

Methyl-3-(4-(4-((1-isopropoxy-2-methyl-1-oxopropan-2-yl)oxy)benzoyl)phenyl)-4-phenylbicyclo[2.1.1]hexane-1-carboxylate (3w)

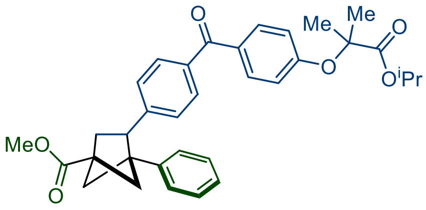

3w

Synthesized following General Procedure B using: BCB **1a** (37.7 mg, 0.200 mmol, 1.00 equiv) and fenofibrate derivative **2w** (352 mg, 1.00 mmol, 5.00 equiv). Purified by flash column chromatography (SiO_2; 95:5 to 80:20 pentane:EtOAc) to afford **3w** (62.9 mg, 0.116 mmol, 58%) as a colourless oil.

TLC: R_f = 0.49 (80:20 pentane:EtOAc).

NMR Spectroscopy (see spectra):

^{1}H NMR (500 MHz, $CDCl_3$): δ_H 7.68 (d, *J* = 8.9 Hz, 2H), 7.54 (d, *J* = 8.3 Hz, 2H), 7.22 – 7.11 (m, 3H), 7.07 (d, *J* = 8.3 Hz, 2H), 6.92 – 6.87 (m, 2H), 6.86 – 6.81 (m, 2H), 5.08 (p, *J* = 6.3 Hz, 1H), 3.76 (s, 3H), 3.66 (dd, *J* = 9.0, 4.4 Hz, 1H), 2.64 (ddd, *J* = 11.5, 9.0, 2.4 Hz, 1H), 2.47 (ddd, *J* = 11.5, 4.5, 2.2 Hz, 1H), 2.38 – 2.31 (m, 1H), 2.26 – 2.20 (m, 2H), 2.15 – 2.08 (m, 1H), 1.65 (s, 6H), 1.19 (d, *J* = 6.3 Hz, 6H) ppm;

^{13}C NMR (126 MHz, $CDCl_3$): δ_C 195.4, 173.4, 173.3, 159.5, 146.1, 141.0, 136.1, 132.0, 130.9, 129.5, 128.4, 128.2, 126.6, 126.2, 117.3, 79.5, 69.4, 56.4, 51.9, 50.5, 49.5, 48.3, 40.2, 37.4, 25.5, 21.7 ppm.

HRMS (ESI$^+$): m/z calc'd for $C_{34}H_{36}O_6Na$ $[M+Na]^+$: 563.24041, found: 563.24038.

Methyl-3-(3-((4-(N,N-dipropylsulfamoyl)benzoyl)oxy)propyl)-4-phenylbicyclo[2.1.1]hexane-1-carboxylate (3x)

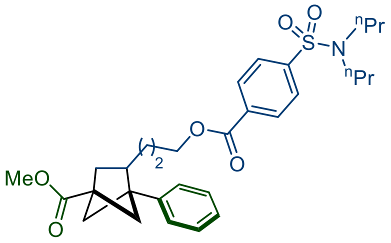

3x

Synthesized following **General Procedure A** using: BCB **1a** (37.7 mg, 0.200 mmol, 1.00 equiv) and probenecid derivative **2x** (707 mg, 2.00 mmol, 10.0 equiv). Purified by flash column chromatography (SiO_2; 95:5 to 80:20 pentane:EtOAc) to afford **3x** (56.3 mg, 0.104 mmol, 52%) as a colourless oil.

TLC: R_f = 0.44 (80:20 pentane:EtOAc).

NMR Spectroscopy (*see spectra*):

^{1}H NMR (500 MHz, $CDCl_3$): δ_H 8.04 (d, *J* = 8.8 Hz, 2H), 7.84 (d, *J* = 8.8 Hz, 2H), 7.33 – 7.27 (m, 2H), 7.23 – 7.18 (m, 1H), 7.14 – 7.09 (m, 2H), 4.25 (qt, *J* = 10.8, 6.7 Hz, 2H), 3.71 (s, 3H), 3.14 – 3.08 (m, 4H), 2.34 (ddd, *J* = 10.7, 8.5, 2.1 Hz, 1H), 2.30 – 2.21 (m, 1H), 2.15 – 1.95 (m, 4H), 1.83 – 1.75 (m, 1H), 1.68 (ddd, *J* = 10.9, 3.9, 2.6 Hz, 1H), 1.63 – 1.52 (m, 5H), 1.49 – 1.39 (m, 1H), 1.33 – 1.21 (m, 1H), 0.87 (t, *J* = 7.4 Hz, 6H) ppm;

^{13}C NMR (126 MHz, $CDCl_3$): δ_C 173.7, 165.3, 144.3, 141.9, 133.8, 130.2, 128.4, 127.1, 126.5, 126.1, 65.6, 54.5, 51.7, 50.1, 48.7, 48.5, 45.0, 40.2, 37.6, 28.1, 27.2, 22.1, 11.3 ppm.

HRMS (ESI^+): m/z calc'd for $C_{30}H_{39}NO_6SNa$ $[M+Na]^+$: 564.23903, found: 564.23893.

Methyl-(3-(((3aR,5R,6S,6aR)-5-((S)-2,2-dimethyl-1,3-dioxolan-4-yl)-2,2-dimethyltetrahydrofuro[2,3-d][1,3]dioxol-6-yl)oxy)-3-oxopropyl)-4-phenylbicyclo[2.1.1]hexane-1-carboxylate (3y)

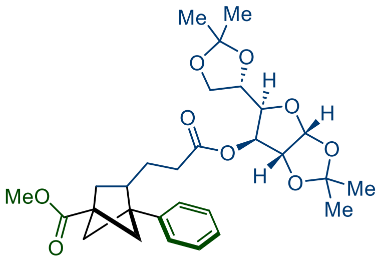

3y

Synthesized following **General Procedure A** using: BCB **1a** (37.7 mg, 0.200 mmol, 1.00 equiv) and glucose derivative **2y** (741 mg, 2.00 mmol, 10.0 equiv). Purified by flash column chromatography (SiO_2; 90:10 to 75:25 pentane:EtOAc) to afford **3y** as a 1:1 mix of diastereomers (44.9 mg, 0.0846 mmol, 42%) as a colourless oil.

TLC: R_f = 0.50 (75:25 pentane:EtOAc).

NMR Spectroscopy (see spectra): *'2×' indicates where individual diastereomer peaks are non-coincident. ^{13}C signals relating to the same carbon are given together.*

^{1}H NMR (500 MHz, $CDCl_3$): δ_H 7.32 – 7.28 (m, 2H), 7.25 – 7.18 (m, 1H), 7.14 – 7.09 (m, 2H), 5.80 (2×d, *J* = 3.6 Hz, 1H), 5.20 (t, *J* = 3.1 Hz, 1H), 4.40 (dd, *J* = 7.7, 3.7 Hz, 1H), 4.20 – 4.07 (m, 2H), 4.07 – 3.95 (m, 2H), 3.70 (2×s, 3H), 2.33 – 2.19 (m, 3H), 2.19 – 2.10 (m, 1H), 2.08 – 1.97 (m, 4H), 1.65 – 1.60 (m, 2H), 1.53 – 1.42 (m, 4H), 1.38 (2×s, 3H), 1.33 – 1.24 (m, 6H) ppm;

^{13}C NMR (126 MHz, $CDCl_3$): δ_C 173.5 and 173.5, 172.1 and 172.1, 141.6, 128.5 and 128.5, 126.7 and 126.6, 126.0 and 126.0, 112.4 and 112.4, 109.5 and 109.4, 105.2, 83.5, 79.9, 76.1 and 76.0, 72.6 and 72.5, 67.4 and 67.4, 54.5 and 54.4, 51.8 and 51.8, 48.8 and 48.7, 48.5 and 48.4, 44.8 and 44.7, 40.2 and 40.1, 37.3 and 37.3, 32.7, 27.2 and 27.0, 27.0, 26.9 and 26.9, 26.4 and 26.3, 25.4 and 25.4 ppm.

HRMS (ESI^+): m/z calc'd for $C_{29}H_{38}O_9Na$ $[M+Na]^+$: 553.24080, found: 553.24075.

Methyl-3-((1S,2S)-2-phenethylcyclopropyl)-4-phenylbicyclo[2.1.1]hexane-1-carboxylate (3z)

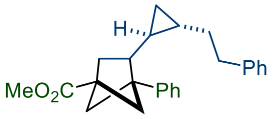

3z

Synthesized following a modified General Procedure A using: BCB **1a** (37.7 mg, 0.200 mmol, 1.00 equiv) and *trans*-(2-(2-vinylcyclopropyl)ethyl)benzene **2z** (172 mg, 1.00 mmol, 5.00 equiv). Purified by flash column chromatography (SiO_2; 100:0 to 95:5 pentane:EtOAc) to afford **3z** as a 1:1.5 mix of diastereomers (28.7 mg, 0.0796 mmol, 40%) as a colourless oil.

TLC: R_f = 0.40 (95:5 pentane:EtOAc).

NMR Spectroscopy (see spectra): *in cases where diastereomer peaks could be distinguished, they are labelled as d^1 and d^2. ^{13}C signals relating to the same carbon are given together.*

^{1}H NMR (500 MHz, $CDCl_3$): δ_H 7.32 – 7.24 (m, 3H), 7.24 – 7.11 (m, 6.4H), 6.98 – 6.90 (m, 0.6H, d^1), 3.71 (2×s, 3H), 2.66 (2×t, *J* = 7.7 Hz, 1H), 2.35 – 1.98 (m, 5H), 1.93 (m, 1H), 1.85 – 1.75 (m, 1H), 1.68 – 1.62 (m, 0.4H, d^2), 1.61 – 1.56 (m, 0.4H, d^2), 1.54 – 1.48 (m, 0.6H, d^1), 1.48 – 1.37 (m, 0.6H, d^1), 1.30 – 1.17 (m, 0.6H, d^1), 1.12 – 1.04 (m, 0.4H, d^2), 0.53 – 0.35 (m, 1.6H), 0.21 (m, 0.4H, d^2), 0.18 – 0.05 (m, 0.8H), −0.05 (dt, *J* = 8.1, 5.0 Hz, 0.6H, d^1), −0.22 (dt, *J* = 9.3, 5.0 Hz, 0.6H, d^1) ppm;

^{13}C NMR (126 MHz, $CDCl_3$): δ_C 174.0 and 174.0, 142.8 and 142.7, 142.6 and 142.6, 128.5 and 128.3, 128.4 and 128.2, 128.2 and 128.0, 126.3 and 126.2, 126.2 and 126.1, 125.8 and 125.5, 55.0 and 54.9, 51.7 and 51.7, 50.7 and 50.5, 48.6 and 48.2, 48.1, 40.8 and 40.6, 38.1 and 37.8, 36.2 and 35.7, 35.0, 21.1 and 21.0, 19.6 and 16.4, 12.6 and 9.7 ppm.

HRMS (ESI^+): m/z calc'd for $C_{25}H_{28}O_2Na$ $[M+Na]^+$: 383.19815, found: 383.19819.

Methyl-2,4-diphenylbicyclo[2.1.1]hexane-1-carboxylate (5a)

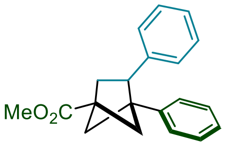

5a

Synthesized following **General Procedure B** using: BCB **1a** (37.7 mg, 0.200 mmol, 1.00 equiv) and styrene **4a** (115 µL, 1.00 mmol, 5.00 equiv). Purified by flash column chromatography (SiO_2; 100:0 to 96:4 pentane:EtOAc) to afford **5a** (42.7 mg, 0.146 mmol, 73%) as a colourless oil.

TLC: R_f = 0.43 (96:4 pentane:EtOAc).

NMR Spectroscopy (*see spectra*):

^{1}**H NMR** (400 MHz, $CDCl_3$): δ_H 7.20 – 7.10 (m, 6H), 7.03 – 6.93 (m, 2H), 6.88 – 6.80 (m, 2H), 3.75 (s, 3H), 3.58 (dd, *J* = 9.1, 4.6 Hz, 1H), 2.59 (ddd, *J* = 11.5, 9.0, 2.3 Hz, 1H), 2.45 (ddd, *J* = 11.5, 4.6, 2.3 Hz, 1H), 2.41 – 2.32 (m, 1H), 2.27 – 2.16 (m, 2H), 2.05 (dt, *J* = 7.0, 1.9 Hz, 1H) ppm;

^{13}**C NMR** (126 MHz, $CDCl_3$): δ_C 173.7, 141.5, 141.4, 128.7, 128.0, 127.9, 126.4, 126.3, 126.2, 56.3, 51.8, 50.5, 49.4, 48.2, 40.1, 37.3 ppm.

HRMS (ESI^+): m/z calc'd for $C_{20}H_{20}O_2Na$ $[M+Na]^+$: 315.13555, found: 315.13547.

Methyl-3-([1,1'-biphenyl]-4-yl)-4-phenylbicyclo[2.1.1]hexane-1-carboxylate (5b)

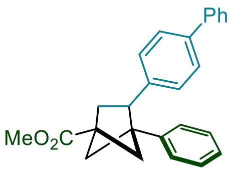

5b

Synthesized following **General Procedure B** using: BCB **1a** (37.7 mg, 0.200 mmol, 1.00 equiv) and 4-vinyl-1,1'-biphenyl **4b** (180 mg, 1.00 mmol, 5.00 equiv). Purified by flash column chromatography (SiO_2; 100:0 to 95:5 pentane:EtOAc) to afford **5b** (30.7 mg, 0.142 mmol, 71%) as a white solid.

TLC: R_f = 0.46 (95:5 pentane:EtOAc).

NMR Spectroscopy (*see spectra*):

^{1}**H NMR** (500 MHz, $CDCl_3$): δ_H 7.54 (d, *J* = 7.1 Hz, 2H), 7.43 – 7.36 (m, 4H), 7.34 – 7.29 (m, 1H), 7.22 – 7.12 (m, 3H), 7.05 (d, *J* = 8.0 Hz, 2H), 6.91 (d, *J* = 6.7 Hz, 2H), 3.77 (s, 3H), 3.63 (dd, *J* = 8.8, 4.2 Hz, 1H),

2.63 (ddd, J = 11.6, 8.8, 2.4 Hz, 1H), 2.49 (ddd, J = 11.6, 4.2, 2.0 Hz, 1H), 2.43 – 2.33 (m, 1H), 2.27 – 2.18 (m, 2H), 2.14 – 2.05 (m, 1H) ppm;

^{13}C NMR (126 MHz, $CDCl_3$): δ_C 173.7, 141.5, 140.9, 140.6, 139.0, 129.1, 128.8, 128.1, 127.2, 127.0, 126.5, 126.4, 126.3, 56.3, 51.8, 50.1, 49.5, 48.2, 40.2, 37.4 ppm.

HRMS (ESI^+): m/z calc'd for $C_{26}H_{24}O_2Na$ $[M+Na]^+$: 391.16685, found: 391.16666.

Methyl-3-(2,4-dimethylphenyl)-4-phenylbicyclo[2.1.1]hexane-1-carboxylate (5c)

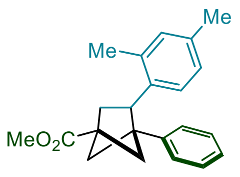

5c

Synthesized following **General Procedure B** using: BCB **1a** (37.7 mg, 0.200 mmol, 1.00 equiv) and 2,4-dimethyl-1-vinylbenzene **4c** (146 µL, 1.00 mmol, 5.00 equiv). Purified by flash column chromatography (SiO_2; 100:0 to 95:5 pentane:EtOAc) to afford **5c** (33.4 mg, 0.104 mmol, 52%) as a colourless oil.

TLC: R_f = 0.38 (95:5 pentane:EtOAc).

NMR Spectroscopy (*see spectra*):

^{1}H NMR (500 MHz, $CDCl_3$): δ_H 7.20 – 7.10 (m, 3H), 7.00 (s, 1H), 6.99 – 6.94 (m, 2H), 6.91 (d, J = 7.6 Hz, 1H), 6.86 (d, J = 7.6 Hz, 1H), 3.79 (dd, J = 8.4, 3.8 Hz, 1H), 3.75 (s, 3H), 2.68 (ddd, J = 11.6, 9.0, 2.4 Hz, 1H), 2.48 (dd, J = 9.1, 7.0 Hz, 1H), 2.25 – 2.12 (m, 7H), 1.89 (s, 3H) ppm;

^{13}C NMR (126 MHz, $CDCl_3$): δ_C 173.8, 141.7, 140.1, 134.9, 134.7, 130.3, 128.1, 127.7, 126.7, 126.4, 126.4, 55.2, 51.8, 50.1, 48.2, 45.2, 40.9, 38.8, 21.4, 19.4 ppm.

HRMS (ESI^+): m/z calc'd for $C_{22}H_{24}O_2Na$ $[M+Na]^+$: 343.16685, found: 343.16685.

Methyl-3-(4-(tert-butyl)phenyl)-4-phenylbicyclo[2.1.1]hexane-1-carboxylate (5d)

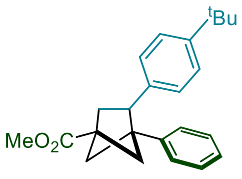

5d

Synthesized following **General Procedure B** using: BCB **1a** (37.7 mg, 0.200 mmol, 1.00 equiv) and 1-(*tert*-butyl)-4-vinylbenzene **4d** (183 µL, 1.00 mmol, 5.00 equiv). Purified by flash column chromatography (SiO_2;

100:0 to 95:5 pentane:EtOAc) to afford **5d** (24.2 mg, 0.112 mmol, 56%) as a colourless oil.

TLC: R_f = 0.34 (95:5 pentane:EtOAc).

NMR Spectroscopy (*see spectra*):

[1]**H NMR** (500 MHz, $CDCl_3$): δ_H 7.20 – 7.12 (m, 5H), 6.93 – 6.86 (m, 4H), 3.75 (s, 3H), 3.57 (dd, *J* = 8.6, 4.0 Hz, 1H), 2.59 (ddd, *J* = 11.4, 9.0, 2.4 Hz, 1H), 2.42 (ddd, *J* = 11.4, 4.7, 2.2 Hz, 1H), 2.39 – 2.29 (m, 1H), 2.22 – 2.13 (m, 2H), 2.10 – 2.04 (m, 1H), 1.26 (s, 9H) ppm;

[13]**C NMR** (126 MHz, $CDCl_3$): δ_C 173.8, 149.0, 141.7, 138.5, 128.3, 128.0, 126.3, 126.3, 124.7, 56.1, 51.8, 49.8, 49.5, 48.2, 40.2, 37.6, 34.4, 31.5 ppm.

HRMS (ESI^+): m/z calc'd for $C_{24}H_{28}O_2Na$ $[M+Na]^+$: 371.19815, found: 371.19819.

Methyl-3-(4-acetoxyphenyl)-4-phenylbicyclo[2.1.1]hexane-1-carboxylate (5e)

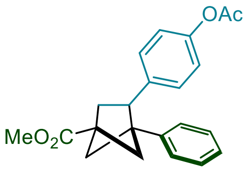

5e

Synthesized following **General Procedure B** using: BCB **1a** (37.7 mg, 0.200 mmol, 1.00 equiv) and 4-vinylphenyl acetate **4e** (153 µL, 1.00 mmol, 5.00 equiv). Purified by flash column chromatography (SiO_2; 100:0 to 80:20 pentane:EtOAc) to afford **5e** (53.2 mg, 0.152 mmol, 76%) as a pale yellow oil.

TLC: R_f = 0.44 (80:20 pentane:EtOAc).

NMR Spectroscopy (*see spectra*):

[1]**H NMR** (500 MHz, $CDCl_3$): δ_H 7.22 – 7.10 (m, 3H), 7.00 – 6.92 (m, 2H), 6.91 – 6.85 (m, 4H), 3.75 (s, 3H), 3.58 (dd, *J* = 9.0, 4.0 Hz, 1H), 2.59 (ddd, *J* = 11.5, 9.0, 2.4 Hz, 1H), 2.41 (ddd, *J* = 11.5, 4.5, 2.2 Hz, 1H), 2.35 – 2.28 (m, 1H), 2.25 (s, 3H), 2.22 – 2.16 (m, 2H), 2.09 – 2.04 (m, 1H) ppm;

[13]**C NMR** (126 MHz, $CDCl_3$): δ_C 173.6, 169.6, 149.1, 141.3, 139.0, 129.5, 128.1, 126.5, 126.2, 120.9, 56.21, 51.8, 49.9, 49.4, 48.2, 40.1, 37.5, 21.2 ppm.

HRMS (ESI^+): m/z calc'd for $C_{22}H_{22}O_4Na$ $[M+Na]^+$: 373.14103, found: 373.14116.

Methyl-3-(4-methoxyphenyl)-4-phenylbicyclo[2.1.1]hexane-1-carboxylate (5f)

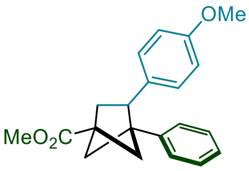

5f

Synthesized following **General Procedure B** using: BCB **1a** (37.7 mg, 0.200 mmol, 1.00 equiv) and 1-methoxy-4-vinylbenzene **4f** (133 µL, 1.00 mmol, 5.00 equiv). Purified by flash column chromatography (SiO_2; 100:0 to 95:5 pentane:EtOAc) to afford **5f** (32.2 mg, 0.0999 mmol, 50%) as a colourless oil.

TLC: R_f = 0.37 (95:5 pentane:EtOAc).

NMR Spectroscopy (*see spectra*):

> **^{1}H NMR** (400 MHz, $CDCl_3$): δ_H 7.22 – 7.09 (m, 3H), 6.96 – 6.80 (m, 4H), 6.74 – 6.65 (m, 2H), 3.75 (s, 3H), 3.74 (s, 3H), 3.52 (dd, *J* = 9.0, 4.4 Hz, 1H), 2.57 (ddd, *J* = 11.5, 9.0, 2.4 Hz, 1H), 2.40 (ddd, *J* = 11.5, 4.4, 2.2 Hz, 1H), 2.36 – 2.29 (m, 1H), 2.23 – 2.13 (m, 2H), 2.03 (ddd, *J* = 7.0, 2.4, 1.7 Hz, 1H) ppm;
>
> **^{13}C NMR** (126 MHz, $CDCl_3$): δ_C 173.8, 158.1, 141.6, 133.4, 129.6, 128.0, 126.3, 126.2, 113.3, 56.3, 55.3, 51.8, 49.8, 49.2, 48.1, 40.1, 37.4 ppm.

HRMS (ESI^+): m/z calc'd for $C_{21}H_{22}O_3Na$ $[M+Na]^+$: 345.14612, found: 345.14609.

Methyl-3-(3-nitrophenyl)-4-phenylbicyclo[2.1.1]hexane-1-carboxylate (5g)

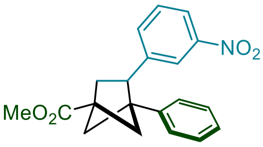

5g

Synthesized following **General Procedure A** using: BCB **1a** (37.7 mg, 0.200 mmol, 1.00 equiv) and 1-nitro-3-vinylbenzene **4g** (279 µL, 2.00 mmol, 10.0 equiv). Purified by flash column chromatography (SiO_2; 100:0 to 90:10 pentane:EtOAc) to afford **5g** (45.8 mg, 0.136 mmol, 68%) as a pale yellow oil.

TLC: R_f = 0.43 (90:10 pentane:EtOAc).

NMR Spectroscopy (*see spectra*):

> **^{1}H NMR** (500 MHz, $CDCl_3$): δ_H 7.98 (ddd, *J* = 8.0, 2.1, 1.1 Hz, 1H), 7.91 (t, *J* = 2.1 Hz, 1H), 7.26 (t, *J* = 8.0 Hz, 1H), 7.22 – 7.12 (m, 4H), 6.87 – 6.84 (m, 2H), 3.77 (s, 3H), 3.67 (dd, *J* = 9.0, 4.5 Hz, 1H), 2.66 (ddd, *J* = 11.7, 9.0, 2.5 Hz, 1H), 2.47 (ddd, *J* = 11.7, 4.5, 2.2 Hz, 1H), 2.32 – 2.20 (m, 3H), 2.13 (ddd, *J* = 6.9, 2.5, 1.6 Hz, 1H) ppm;

^{13}**C NMR** (126 MHz, $CDCl_3$): δ_C 173.1, 148.0, 143.6, 140.5, 135.0, 128.7, 128.4, 126.9, 126.0, 123.0, 121.5, 56.5, 51.9, 50.3, 49.3, 48.3, 39.9, 37.0 ppm.

HRMS (ESI$^+$): m/z calc'd for $C_{20}H_{19}NO_4Na$ $[M+Na]^+$: 360.12063, found: 360.12055.

Methyl-4-phenyl-3-(4-(trifluoromethyl)phenyl)bicyclo[2.1.1]hexane-1-carboxylate (5h)

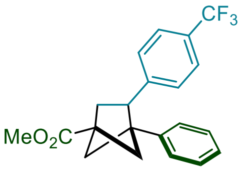

5h

Synthesized following **General Procedure B** using: BCB **1a** (37.7 mg, 0.200 mmol, 1.00 equiv) and 1-(trifluoromethyl)-4-vinylbenzene **4h** (133 µL, 1.00 mmol, 5.00 equiv). Purified by flash column chromatography (SiO_2; 100:0 to 95:5 pentane:EtOAc) to afford **5h** (44.0 mg, 0.122 mmol, 61%) as a colourless oil.

TLC: R_f = 0.38 (95:5 pentane:EtOAc).

NMR Spectroscopy (*see spectra*):

^{1}**H NMR** (400 MHz, $CDCl_3$): δ_H 7.39 (d, *J* = 8.2 Hz, 2H), 7.24 – 7.13 (m, 3H), 7.08 (d, *J* = 8.1 Hz, 2H), 6.92 – 6.84 (m, 2H), 3.76 (s, 3H), 3.70 – 3.60 (m, 1H), 2.63 (ddd, *J* = 11.6, 9.0, 2.5 Hz, 1H), 2.43 (ddd, *J* = 11.6, 4.5, 2.1 Hz, 1H), 2.29 (ddd, *J* = 9.0, 7.1, 3.7 Hz, 1H), 2.26 – 2.17 (m, 2H), 2.12 (dt, *J* = 7.1, 2.1 Hz, 1H) ppm;

^{13}**C NMR** (101 MHz, $CDCl_3$): δ_C 173.3, 145.6, 140.9, 128.9, 128.6, 128.3, 126.7, 126.1, 124.8, 124.4, 56.3, 51.9, 50.2, 49.5, 48.3, 40.0, 37.3 ppm;

^{19}**F NMR** (376 MHz, $CDCl_3$): δ_F –62.4 ppm.

HRMS (ESI$^+$): m/z calc'd for $C_{21}H_{19}F_3O_2Na$ $[M+Na]^+$: 383.12294, found: 383.12305.

Methyl-3-(2-chlorophenyl)-4-phenylbicyclo[2.1.1]hexane-1-carboxylate (5i)

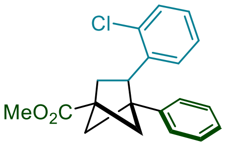

5i

Synthesized following **General Procedure B** using: BCB **1a** (37.7 mg, 0.200 mmol, 1.00 equiv) and 1-chloro-2-vinylbenzene **4i** (127 µL, 1.00 mmol, 5.00 equiv). Purified by flash column chromatography (SiO_2; 100:0 to 93:7 pentane:EtOAc) to afford **5i** (41.8 mg, 0.128 mmol, 64%) as a colourless oil.

TLC: R_f = 0.40 (93:7 pentane:EtOAc).

NMR Spectroscopy (*see spectra*):

^{1}H NMR (500 MHz, $CDCl_3$): δ_H 7.29 (dd, *J* = 7.7, 1.6 Hz, 1H), 7.25 – 7.21 (m, 2H), 7.20 – 7.15 (m, 1H), 7.13 (d, *J* = 7.3 Hz, 2H), 7.09 (dd, *J* = 7.4, 2.1 Hz, 1H), 7.07 – 7.00 (m, 2H), 4.11 (dd, *J* = 9.0, 4.2 Hz, 1H), 3.73 (s, 3H), 2.89 (ddd, *J* = 11.3, 9.0, 2.5 Hz, 1H), 2.51 – 2.38 (m, 1H), 2.37 – 2.30 (m, 1H), 2.17 – 2.09 (m, 2H), 2.04 (ddd, *J* = 11.3, 4.4, 2.5 Hz, 1H).ppm;

^{13}C NMR (126 MHz, $CDCl_3$): δ_C 173.4, 141.1, 140.3, 135.7, 129.8, 128.4, 128.4, 127.2, 126.6, 126.6, 126.3, 54.4, 51.8, 51.1, 48.3, 45.0, 40.9, 39.6 ppm.

HRMS (ESI^+): m/z calc'd for $C_{20}H_{19}O_2ClNa$ $[M+Na]^+$: 349.09658, found: 349.09649.

Methyl-3-(3-chlorophenyl)-4-phenylbicyclo[2.1.1]hexane-1-carboxylate (5j)

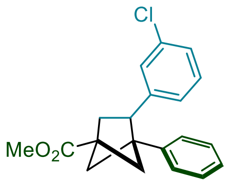

5j

Synthesized following **General Procedure B** using: BCB **1a** (37.7 mg, 0.200 mmol, 1.00 equiv) and 1-chloro-3-vinylbenzene **4j** (127 µL, 1.00 mmol, 5.00 equiv). Purified by flash column chromatography (SiO_2; 100:0 to 93:7 pentane:EtOAc) to afford **5j** (44.4 mg, 0.136 mmol, 68%) as a colourless oil.

TLC: R_f = 0.44 (93:7 pentane:EtOAc).

NMR Spectroscopy (*see spectra*):

^{1}H NMR (400 MHz, $CDCl_3$): δ_H 7.22 – 7.15 (m, 3H), 7.11 (d, *J* = 8.5 Hz, 2H), 6.90 (d, *J* = 8.5 Hz, 2H), 6.87 – 6.82 (m, 2H), 3.75 (s, 3H), 3.54 (dd, *J* = 9.0, 3.9 Hz, 1H), 2.59 (ddd, *J* = 11.6, 9.0, 2.4 Hz, 1H), 2.39 (ddd, *J* = 11.6, 4.5, 2.4 Hz, 1H), 2.28 (ddd, *J* = 8.2, 6.8, 3.4 Hz, 1H), 2.24 – 2.14 (m, 2H), 2.06 (ddd, *J* = 6.8, 2.4, 1.6 Hz, 1H) ppm;

^{13}C NMR (126 MHz, $CDCl_3$): δ_C 173.4, 143.6, 141.0, 133.8, 129.0, 128.6, 128.2, 127.0, 126.6, 126.5, 126.1, 56.3, 51.9, 50.3, 49.3, 48.2, 40.1, 37.2 ppm.

HRMS (ESI^+): m/z calc'd for $C_{20}H_{19}O_2ClNa$ $[M+Na]^+$: 349.09658, found: 349.09648.

Methyl-3-(4-chlorophenyl)-4-phenylbicyclo[2.1.1]hexane-1-carboxylate (5k)

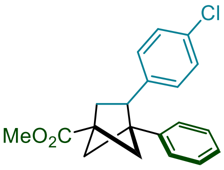

5k

Synthesized following **General Procedure B** using: BCB **1a** (37.7 mg, 0.200 mmol, 1.00 equiv) and 1-chloro-4-vinylbenzene **4k** (120 µL, 1.00 mmol, 5.00 equiv). Purified by flash column chromatography (SiO_2; 100:0 to 93:7 pentane:EtOAc) to afford **5k** (47.1 mg, 0.144 mmol, 72%) as a colourless oil.

TLC: R_f = 0.40 (93:7 pentane:EtOAc).

NMR Spectroscopy (*see spectra*):

^{1}**H NMR** (400 MHz, $CDCl_3$): δ_H 7.22 – 7.09 (m, 5H), 6.92 – 6.83 (m, 4H), 3.75 (s, 3H), 3.54 (dd, *J* = 9.0, 4.4 Hz, 1H), 2.59 (ddd, *J* = 11.5, 9.0, 2.5 Hz, 1H), 2.38 (ddd, *J* = 11.5, 4.4, 2.2 Hz, 1H), 2.32 – 2.23 (m, 1H), 2.22 – 2.15 (m, 2H), 2.06 (ddd, *J* = 7.0, 2.5, 1.6 Hz, 1H) ppm;

^{13}**C NMR** (101 MHz, $CDCl_3$): δ_C 173.5, 141.1, 139.9, 132.1, 129.9, 128.2, 128.0, 126.6, 126.2, 56.3, 51.9, 50.0, 49.3, 48.2, 40.0, 37.3 ppm.

HRMS (ESI$^+$): m/z calc'd for $C_{20}H_{19}O_2ClNa$ $[M+Na]^+$: 349.09658, found: 349.09645.

Methyl-3-(4-bromophenyl)-4-phenylbicyclo[2.1.1]hexane-1-carboxylate (5l)

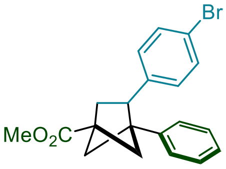

5l

Synthesized following **General Procedure B** using: BCB **1a** (37.7 mg, 0.200 mmol, 1.00 equiv) and 1-bromo-4-vinylbenzene **4l** (131 µL, 1.00 mmol, 5.00 equiv). Purified by flash column chromatography (SiO_2; 100:0 to 95:5 pentane:EtOAc) to afford **5l** (44.6 mg, 0.120 mmol, 60%) as a white solid.

TLC: R_f = 0.48 (95:5 pentane:EtOAc).

NMR Spectroscopy (*see spectra*):

^{1}**H NMR** (400 MHz, $CDCl_3$): δ_H 7.25 (d, *J* = 8.5 Hz, 2H), 7.22 – 7.14 (m, 3H), 6.88 – 6.82 (m, 4H), 3.75 (s, 3H), 3.52 (dd, *J* = 9.0, 4.5 Hz, 1H), 2.59 (ddd, *J* = 11.6, 9.0, 2.5 Hz, 1H), 2.38 (ddd, *J* = 11.6, 4.5, 2.3 Hz, 1H), 2.31 – 2.22 (m, 1H), 2.23 – 2.13 (m, 2H), 2.06 (ddd, *J* = 7.0, 2.5, 1.7 Hz, 1H) ppm;

^{13}**C NMR** (101 MHz, $CDCl_3$): δ_C 173.5, 141.1, 140.4, 130.9, 130.3, 128.2, 126.6, 126.2, 120.3, 56.2, 51.9,

50.0, 49.4, 48.2, 40.0, 37.3 ppm.

HRMS (ESI$^+$): m/z calc'd for $C_{20}H_{19}O_2BrNa$ [M+Na]$^+$: 393.04716, found: 393.04603.

Methyl-4-(methoxycarbonyl)-1-phenylbicyclo[2.1.1]hexan-2-yl)benzoic acid (5m)

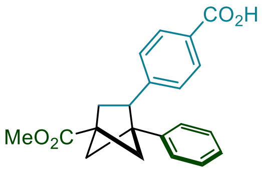

5m

Synthesized following **General Procedure B** using: BCB **1a** (37.7 mg, 0.200 mmol, 1.00 equiv) and 4-vinylbenzoic acid **4m** (148 mg, 1.00 mmol, 5.00 equiv). Purified by flash column chromatography (SiO_2; 90:10 to 70:30 pentane:EtOAc) to afford **5m** (43.7 mg, 0.120 mmol, 65%) as a white solid.

TLC: R_f = 0.39 (70:30 pentane:EtOAc).

NMR Spectroscopy (*see spectra*):

^{1}**H NMR** (500 MHz, $CDCl_3$): δ_H 7.87 (d, *J* = 8.3 Hz, 2H), 7.20 – 7.12 (m, 3H), 7.07 (d, *J* = 8.3 Hz, 2H), 6.92 – 6.83 (m, 2H), 3.76 (s, 3H), 3.64 (dd, *J* = 9.0, 4.4 Hz, 1H), 2.63 (ddd, *J* = 11.5, 9.0, 2.4 Hz, 1H), 2.46 (ddd, *J* = 11.5, 4.4, 2.1 Hz, 1H), 2.37 – 2.28 (m, 1H), 2.24 – 2.19 (m, 2H), 2.10 (dt, *J* = 7.2, 2.1 Hz, 1H) ppm (*carboxylic acid OH signal not observed due to peak broadening*);

^{13}**C NMR** (126 MHz, $CDCl_3$): δ_C 173.4, 148.0, 140.9, 129.8, 128.8, 128.2, 127.3, 126.7, 126.1, 56.5, 51.9, 50.7, 49.5, 48.3, 40.1, 37.3 ppm (*carboxylic acid C=O signal not observed due to peak broadening)*.

HRMS (ESI$^-$): m/z calc'd for $C_{21}H_{19}O_4$ [M–H]$^-$: 335.12779, found: 335.12874.

Methyl-4-phenyl-3-(4-(4,4,5,5-tetramethyl-1,3,2-dioxaborolan-2-yl)phenyl)bicyclo[2.1.1]hexane-1-carboxylate (5n)

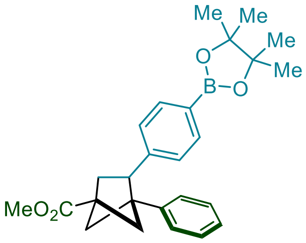

5n

Synthesized following **General Procedure B** using: BCB **1a** (37.7 mg, 0.200 mmol, 1.00 equiv) and 4,4,5,5-tetramethyl-2-(4-vinylphenyl)-1,3,2-dioxaborolane **4n** (230 mg, 1.00 mmol, 5.00 equiv). Purified by flash column chromatography (SiO_2; 100:0 to 94:6 pentane:EtOAc) to afford **5n** (52.7 mg, 0.126 mmol, 63%) as a colourless

oil.

TLC: R_f = 0.36 (94:6 pentane:EtOAc).

NMR Spectroscopy (*see spectra*):

> **^{1}H NMR** (500 MHz, $CDCl_3$): δ_H δ 7.58 (d, *J* = 8.0 Hz, 2H), 7.18 – 7.14 (m, 2H), 7.14 – 7.10 (m, 1H), 6.99 (d, *J* = 8.0 Hz, 2H), 6.90 – 6.83 (m, 2H), 3.75 (s, 3H), 3.59 (dd, *J* = 9.0, 4.4 Hz, 1H), 2.59 (ddd, *J* = 11.5, 9.0, 2.4 Hz, 1H), 2.45 (ddd, *J* = 11.5, 4.4, 2.1 Hz, 1H), 2.40 – 2.32 (m, 1H), 2.23 – 2.16 (m, 2H), 2.06 (ddd, *J* = 7.0, 2.4, 2.1 Hz, 1H), 1.32 (s, 12H) ppm;

> **^{13}C NMR** (126 MHz, $CDCl_3$): δ_C 173.7, 144.8, 141.3, 134.4, 128.1, 128.1, 126.4, 126.2, 83.8, 56.3, 51.8, 50.6, 49.6, 48.2, 40.1, 37.3, 25.0 ppm (*aromatic carbon adjacent to boron not observed due to quadrupolar relaxation)*.

HRMS (ESI^+): m/z calc'd for $C_{26}H_{31}O_4BNa$ $[M+Na]^+$: 441.22123, found: 441.22062.

Methyl-3-phenyl-3,3a,8,8a-tetrahydro-1,3-methanocyclopenta[a]indene-1(2H)-carboxylate (5o)

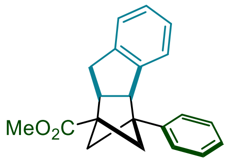

5o

Synthesized following **General Procedure B** using: BCB **1a** (37.7 mg, 0.200 mmol, 1.00 equiv) and 1*H*-indene **4o** (117 µL, 1.00 mmol, 5.00 equiv). Purified by flash column chromatography (SiO_2; 100:0 to 95:5 pentane:EtOAc) to afford **5o** as a single diastereomer (43.2 mg, 0.142 mmol, 71%) as a pale yellow oil.

TLC: R_f = 0.41 (95:5 pentane:EtOAc).

NMR Spectroscopy (*see spectra*):

> **^{1}H NMR** (500 MHz, $CDCl_3$): δ_H 7.36 – 7.29 (m, 2H), 7.30 – 7.22 (m, 1H), 7.19 (d, *J* = 7.5 Hz, 1H), 7.14 (t, *J* = 7.5 Hz, 1H), 7.11 – 7.06 (m, 2H), 6.94 (t, *J* = 7.4 Hz, 1H), 6.37 (d, *J* = 7.5 Hz, 1H), 3.92 (d, *J* = 8.0 Hz, 1H), 3.75 (s, 3H), 3.40 (tdd, *J* = 8.0, 3.6, 1.7 Hz, 1H), 3.19 (dd, *J* = 17.5, 9.7 Hz, 1H), 2.89 (dd, *J* = 17.5, 3.6 Hz, 1H), 2.26 (dd, *J* = 9.7, 6.4 Hz, 1H), 2.16 (d, *J* = 6.4 Hz, 1H), 1.92 (dt, *J* = 7.4, 1.7 Hz, 1H), 1.71 (dd, *J* = 9.7, 7.4 Hz, 1H) ppm;

> **^{13}C NMR** (126 MHz, $CDCl_3$): δ_C 173.5, 146.2, 141.8, 141.4, 128.2, 127.1, 126.6, 126.5, 125.9, 125.3, 124.6, 58.8, 55.1, 52.3, 51.8, 50.5, 46.4, 35.6, 34.4 ppm.

HRMS (ESI^+): m/z calc'd for $C_{21}H_{20}O_2Na$ $[M+Na]^+$: 327.13555, found: 327.13562.

Methyl-1-phenyl-1,2,3a,4,5,9b-hexahydro-3H-1,3-methanocyclopenta[a]naphthalene-3-carboxylate (5p)

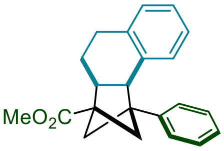

5p

Synthesized following **General Procedure B** using: BCB **1a** (37.7 mg, 0.200 mmol, 1.00 equiv) and 1,2-dihydronaphthalene **4p** (131 µL, 1.00 mmol, 5.00 equiv). Purified by flash column chromatography (SiO_2; 100:0 to 95:5 pentane:EtOAc) to afford **5p** as a single diastereomer (44.7 mg, 0.140 mmol, 70%) as a colourless oil.

TLC: R_f = 0.42 (95:5 pentane:EtOAc).

NMR Spectroscopy (*see spectra*):

^{1}**H NMR** (500 MHz, $CDCl_3$): δ_H 7.33 – 7.26 (m, 2H), 7.27 – 7.21 (m, 1H), 7.09 (d, *J* = 7.5 Hz, 1H), 7.07 – 7.00 (m, 3H), 6.77 (t, *J* = 7.5 Hz, 1H), 6.23 (d, *J* = 7.8 Hz, 1H), 3.76 (s, 3H), 3.51 (d, *J* = 9.5 Hz, 1H), 3.07 – 3.00 (m, 1H), 2.87 (ddd, *J* = 15.5, 9.5, 4.7 Hz, 1H), 2.64 (ddd, *J* = 15.5, 7.0, 4.7 Hz, 1H), 2.28 (dd, *J* = 9.5, 6.3 Hz, 1H), 2.09 – 1.95 (m, 3H), 1.91 (d, *J* = 7.4 Hz, 1H), 1.86 – 1.76 (m, 1H) ppm;

^{13}**C NMR** (126 MHz, $CDCl_3$): δ_C 173.6, 142.6, 139.3, 136.9, 129.7, 128.6, 128.3, 126.6, 126.5, 125.8, 125.3, 56.2, 52.2, 51.7, 51.0, 46.9, 43.1, 37.1, 28.8, 24.8 ppm.

HRMS (ESI$^+$): m/z calc'd for $C_{22}H_{22}O_2Na$ $[M+Na]^+$: 341.15120, found: 341.15117.

Methyl-9-phenyl-6b,8,9,9a-tetrahydro-7H-7,9-methanocyclopenta[a]acenaphthylene-7-carboxylate (5q)

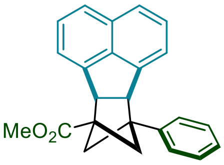

5q

Synthesized following **General Procedure B** using: BCB **1a** (37.7 mg, 0.200 mmol, 1.00 equiv) and 1,2-acenaphthylene **4q** (152 mg, 1.00 mmol, 5.00 equiv). Purified by flash column chromatography (SiO_2; 100:0 to 96:4 pentane:EtOAc) to afford **5q** as a single diastereomer (34.7 mg, 0.102 mmol, 51%) as a colourless oil.

TLC: R_f = 0.36 (96:4 pentane:EtOAc).

NMR Spectroscopy (*see spectra*):

^{1}**H NMR** (500 MHz, $CDCl_3$): δ_H 7.66 (d, *J* = 8.0 Hz, 1H), 7.62 (d, *J* = 8.2 Hz, 1H), 7.48 (dd, *J* = 8.2, 6.9 Hz, 1H), 7.41 – 7.34 (m, 3H), 7.33 – 7.27 (m, 2H), 7.21 – 7.13 (m, 2H), 6.57 (d, *J* = 6.9 Hz, 1H), 4.57 (d, *J* = 6.5 Hz, 1H), 4.30 (d, *J* = 6.5 Hz, 1H), 3.82 (s, 3H), 2.51 (dd, *J* = 9.4, 6.3 Hz, 1H), 2.34 (d, *J* = 6.3 Hz, 1H),

1.93 (d, J = 7.5 Hz, 1H), 1.30 (dd, J = 9.4, 7.5 Hz, 1H) ppm;

^{13}C NMR (126 MHz, $CDCl_3$): δ_C 172.6, 142.9, 142.7, 142.4, 141.0, 131.3, 128.4, 128.0, 127.6, 126.8, 126.6, 123.8, 123.5, 120.8, 120.6, 57.0, 54.1, 53.8, 52.4, 52.3, 51.8, 35.3 ppm.

HRMS (ESI$^+$): m/z calc'd for $C_{24}H_{21}O_2Na$ [M+H]$^+$: 341.14916, found: 341.14919.

Methyl-2-methyl-3,4-diphenylbicyclo[2.1.1]hexane-1-carboxylate (5r)

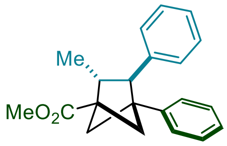

5r

Synthesized following **General Procedure B** using: BCB **1a** (37.7 mg, 0.200 mmol, 1.00 equiv) and (*E*)-prop-1-en-1-ylbenzene **4r** (130 µL, 1.00 mmol, 5.00 equiv). Purified by flash column chromatography (SiO_2; 100:0 to 96:4 pentane:EtOAc) to afford **5r** as a single diastereomer (46.0 mg, 0.150 mmol, 75%) as a white solid.

TLC: R_f = 0.40 (96:4 pentane:EtOAc).

NMR Spectroscopy (*see spectra*):

^{1}H NMR (500 MHz, $CDCl_3$): δ_H 7.18 – 7.12 (m, 6H), 7.06 – 7.02 (m, 2H), 6.92 – 6.86 (m, 2H), 3.75 (s, 3H), 2.99 (dd, J = 4.7, 1.9 Hz, 1H), 2.92 – 2.82 (m, 1H), 2.50 (dd, J = 9.4, 6.9 Hz, 1H), 2.33 (dd, J = 9.4, 6.9 Hz, 1H), 2.15 – 2.12 (m, 2H), 1.28 (d, J = 6.7 Hz, 3H) ppm;

^{13}C NMR (126 MHz, $CDCl_3$): δ_C 173.5, 141.5, 141.2, 128.6, 128.0, 128.0, 126.3, 126.3, 126.2, 59.2, 55.2, 51.7, 51.6, 44.7, 44.4, 43.4, 17.0 ppm.

HRMS (ESI$^+$): m/z calc'd for $C_{21}H_{22}O_2Na$ [M+Na]$^+$: 329.15120, found: 329.15109.

Methyl-3-(4-methoxyphenyl)-2-methyl-4-phenylbicyclo[2.1.1]hexane-1-carboxylate (5s)

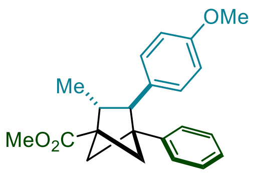

5s

Synthesized following **General Procedure B** using: BCB **1a** (37.7 mg, 0.200 mmol, 1.00 equiv) and (*E*)-1-methoxy-4-(prop-1-en-1-yl)benzene **4s** (150 µL, 1.00 mmol, 5.00 equiv). Purified by flash column chromatography (SiO_2; 100:0 to 95:5 pentane:EtOAc) to afford **5s** as a single diastereomer (55.2 mg, 0.164 mmol, 82%) as a white solid.

TLC: R_f = 0.36 (95:5 pentane:EtOAc).

NMR Spectroscopy (*see spectra*):

^{1}**H NMR** (500 MHz, $CDCl_3$): δ_H 7.19 – 7.09 (m, 3H), 6.95 (d, *J* = 8.8 Hz, 2H), 6.91 – 6.84 (m, 2H), 6.70 (d, *J* = 8.8 Hz, 2H), 3.75 (s, 3H), 3.74 (s, 3H), 2.93 (dd, *J* = 4.8, 1.9 Hz, 1H), 2.87 – 2.78 (m, 1H), 2.47 (dd, *J* = 9.4, 6.9 Hz, 1H), 2.31 (dd, *J* = 9.4, 7.0 Hz, 1H), 2.12 (t, *J* = 1.9 Hz, 1H), 2.11 (t, *J* = 2.0 Hz, 1H), 1.26 (d, *J* = 6.7 Hz, 3H) ppm;

^{13}**C NMR** (126 MHz, $CDCl_3$): δ_C 173.5, 158.1, 141.6, 133.2, 129.6, 128.0, 126.2, 126.2, 113.4, 58.5, 55.3, 55.2, 51.6, 51.5, 44.8, 44.2, 43.4, 17.0 ppm.

HRMS (ESI^+): m/z calc'd for $C_{22}H_{24}O_3Na$ $[M+Na]^+$: 359.16177, found: 359.16176.

Methyl-3-methyl-3,4-diphenylbicyclo[2.1.1]hexane-1-carboxylate (5u)

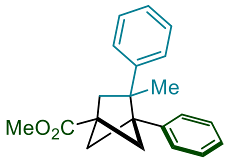

5u

Synthesized following **General Procedure B** using: BCB **1a** (37.7 mg, 0.200 mmol, 1.00 equiv) and prop-1-en-2-ylbenzene **4u** (130 µL, 1.00 mmol, 5.00 equiv). Purified by flash column chromatography (SiO_2; 100:0 to 95:5 pentane:EtOAc) to afford **5u** (19.0 mg, 0.0620 mmol, 31%) as a colourless oil.

TLC: R_f = 0.42 (95:5 pentane:EtOAc).

NMR Spectroscopy (*see spectra*):

^{1}**H NMR** (500 MHz, $CDCl_3$): δ_H 7.24 – 7.18 (m, 3H), 7.17 – 7.10 (m, 3H), 6.99 – 6.91 (m, 2H), 6.86 – 6.80 (m, 2H), 3.74 (s, 3H), 3.13 (dd, *J* = 11.7, 2.8 Hz, 1H), 2.56 (dd, *J* = 9.7, 6.9 Hz, 1H), 2.21 (dd, *J* = 9.7, 6.9 Hz, 1H), 2.16 – 2.06 (m, 2H), 1.92 (dd, *J* = 6.9, 2.8 Hz, 1H), 1.56 (s, 3H) ppm;

^{13}**C NMR** (126 MHz, $CDCl_3$): δ_C 173.8, 145.1, 141.2, 128.2, 127.7, 127.6, 127.3, 126.5, 125.9, 58.5, 51.8, 49.1, 47.4, 45.8, 43.6, 42.9, 26.7 ppm.

HRMS (ESI^+): m/z calc'd for $C_{21}H_{22}O_2Na$ $[M+Na]^+$: 329.15120, found: 329.15117.

Methyl-3,3a-diphenyloctahydro-1H-1,3-methanoindene-1-carboxylate (5v)

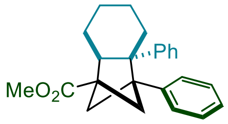

5v

Synthesized following **General Procedure B** using: BCB **1a** (37.7 mg, 0.200 mmol, 1.00 equiv) and 2,3,4,5-tetrahydro-1,1'-biphenyl **4v** (160 µL, 1.00 mmol, 5.00 equiv). Purified by flash column chromatography (SiO_2; 100:0 to 95:5 pentane:EtOAc) to afford **5v** as a single diastereomer (31.9 mg, 0.0921 mmol, 46%) as a colourless oil.

TLC: R_f = 0.40 (95:5 pentane:EtOAc).

NMR Spectroscopy (*see spectra*):

^{1}**H NMR** (500 MHz, $CDCl_3$): δ_H 7.24 – 7.19 (m, 3H), 7.15 – 7.09 (m, 3H), 6.97 – 6.92 (m, 2H), 6.83 – 6.76 (m, 2H), 3.73 (s, 3H), 3.29 (dd, *J* = 12.2, 5.8 Hz, 1H), 2.72 (dd, *J* = 9.7, 7.5 Hz, 1H), 2.10 – 1.99 (m, 3H), 1.86 – 1.75 (m, 3H), 1.74 – 1.64 (m, 1H), 1.58 – 1.40 (m, 2H), 1.31 – 1.21 (m, 1H), 0.97 – 0.86 (m, 1H) ppm;

^{13}**C NMR** (126 MHz, $CDCl_3$): δ_C 173.6, 143.0, 141.7, 128.9, 127.6, 127.4, 127.1, 126.5, 125.9, 58.5, 52.4, 51.6, 51.0, 44.7, 43.9, 41.2, 28.2, 22.6, 18.3, 16.7 ppm.

HRMS (ESI$^+$): m/z calc'd for $C_{24}H_{26}O_2Na$ $[M+Na]^+$: 369.18250, found: 369.18242.

Methyl-3-methyl-2,3,4-triphenylbicyclo[2.1.1]hexane-1-carboxylate (5w)

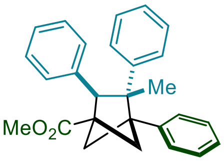

5w

Synthesized following **General Procedure B** using: BCB **1a** (37.7 mg, 0.200 mmol, 1.00 equiv) and (*E*)-prop-1-ene-1,2-diyldibenzene **4w** (194 mg, 1.00 mmol, 5.00 equiv). Purified by flash column chromatography (SiO_2; 100:0 to 95:5 pentane:EtOAc) to afford **5w** as a single diastereomer (28.3 mg, 0.0740 mmol, 37%) as a pale yellow oil.

TLC: R_f = 0.48 (95:5 pentane:EtOAc).

NMR Spectroscopy (*see spectra*):

^{1}**H NMR** (500 MHz, $CDCl_3$): δ_H 7.41 – 7.28 (m, 11H), 7.27 – 7.23 (m, 2H), 6.90 – 6.81 (m, 2H), 4.95 (s, 1H), 3.76 (s, 3H), 3.01 (dd, *J* = 9.7, 7.6 Hz, 1H), 2.52 (dd, *J* = 9.7, 6.8 Hz, 1H), 2.46 (dd, *J* = 7.6, 1.9 Hz, 1H), 2.08 (d, *J* = 6.8 Hz, 1H), 1.11 (s, 3H) ppm;

^{13}**C NMR** (126 MHz, $CDCl_3$): δ_C 173.5, 145.1, 141.0, 140.7, 128.4, 128.4, 128.1, 127.8, 127.5, 127.5, 126.6, 126.3, 126.2, 57.8, 53.3, 52.3, 51.9, 50.6, 46.3, 41.6, 23.3 ppm.

HRMS (ESI$^+$): m/z calc'd for $C_{27}H_{26}O_2Na$ $[M+Na]^+$: 405.18250, found: 405.18220.

Methyl-3-(naphthalen-2-yl)-4-phenylbicyclo[2.1.1]hexane-1-carboxylate (5y)

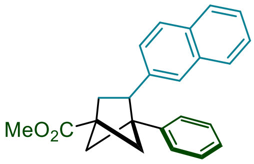

5y

Synthesized following **General Procedure B** using: BCB **1a** (37.7 mg, 0.200 mmol, 1.00 equiv) and 2-vinylnaphthalene **4y** (154 mg, 1.00 mmol, 5.00 equiv). Purified by flash column chromatography (SiO_2; 100:0 to 96:4 pentane:EtOAc) to afford **5y** (25.4 mg, 0.0742 mmol, 37%) as a colourless oil.

TLC: R_f = 0.42 (96:4 pentane:EtOAc).

NMR Spectroscopy (*see spectra*):

^{1}H NMR (500 MHz, $CDCl_3$): δ_H 7.74 – 7.66 (m, 2H), 7.59 – 7.50 (m, 2H), 7.45 – 7.37 (m, 2H), 7.18 – 7.07 (m, 3H), 6.93 (dd, *J* = 8.5, 1.9 Hz, 1H), 6.91 – 6.84 (m, 2H), 3.78 (s, 3H), 3.74 (dd, *J* = 8.4, 4.7 Hz, 1H), 2.70 – 2.54 (m, 2H), 2.44 (t, *J* = 8.4 Hz, 1H), 2.29 – 2.21 (m, 2H), 2.10 (dt, *J* = 7.3, 2.0 Hz, 1H) ppm;

^{13}C NMR (126 MHz, $CDCl_3$): δ_C 173.8, 141.5, 139.1, 133.2, 132.2, 128.2, 128.1, 127.9, 127.5, 127.1, 126.4, 126.4, 126.3, 125.9, 125.5, 56.4, 51.9, 50.6, 49.5, 48.2, 40.3, 37.3 ppm.

HRMS (ESI$^+$): m/z calc'd for $C_{24}H_{23}O_2Na$ $[M+H]^+$: 343.16482, found: 343.16849.

Methyl-4-phenyl-3-(thiophen-2-yl)bicyclo[2.1.1]hexane-1-carboxylate (5z)

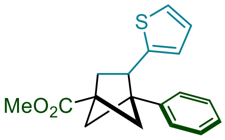

5z

Synthesized following **General Procedure B** using: BCB **1a** (37.7 mg, 0.200 mmol, 1.00 equiv) and 2-vinylthiophene **4z** (100 µL, 1.00 mmol, 5.00 equiv). Purified by flash column chromatography (SiO_2; 100:0 to 95:5 pentane:EtOAc) to afford **5z** (10.8 mg, 0.0499 mmol, 25%) as a colourless oil.

TLC: R_f = 0.53 (95:5 pentane:EtOAc).

NMR Spectroscopy (*see spectra*):

^{1}H NMR (500 MHz, $CDCl_3$): δ_H 7.25 – 7.14 (m, 3H), 7.04 (d, *J* = 5.1 Hz, 1H), 6.99 – 6.93 (m, 2H), 6.81 (dd, *J* = 5.1, 3.5 Hz, 1H), 6.54 (d, *J* = 3.5 Hz, 1H), 3.78 (dd, *J* = 9.2, 4.4 Hz, 1H), 3.75 (s, 3H), 2.72 (ddd, *J* = 11.5, 9.2, 2.6 Hz, 1H), 2.39 – 2.31 (m, 2H), 2.24 – 2.11 (m, 3H) ppm;

^{13}C NMR (126 MHz, $CDCl_3$): δ_C 173.4, 145.7, 141.1, 128.1, 126.7, 126.5, 126.3, 125.4, 123.5, 56.5, 51.9,

48.8, 48.0, 46.6, 40.9, 40.4 ppm.

HRMS (ESI+): m/z calc'd for $C_{18}H_{18}O_2SNa$ [M+Na]+: 321.09307, found: 321.09185.

Methyl-3-(4-methylthiazol-5-yl)-4-phenylbicyclo[2.1.1]hexane-1-carboxylate (5aa)

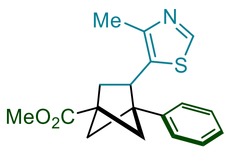

5aa

Synthesized following **General Procedure B** using: BCB **1a** (37.7 mg, 0.200 mmol, 1.00 equiv) and 4-methyl-5-vinylthiazole **4aa** (115 µL, 1.00 mmol, 5.00 equiv). Purified by flash column chromatography (SiO_2; 100:0 to 70:30 pentane:EtOAc) to afford **5aa** (16.9 mg, 0.0539 mmol, 27%) as a colourless oil.

TLC: R_f = 0.40 (70:30 pentane:EtOAc).

NMR Spectroscopy (*see spectra*):

^{1}H NMR (500 MHz, $CDCl_3$): δ_H 8.52 (s, 1H), 7.21 – 7.14 (m, 3H), 6.91 – 6.81 (m, 2H), 3.78 – 3.69 (m, 4H), 2.81 (ddd, *J* = 11.6, 8.9, 2.7 Hz, 1H), 2.43 (dd, *J* = 9.4, 7.5 Hz, 1H), 2.26 (dd, *J* = 6.6, 2.7 Hz, 1H), 2.20 (dd, *J* = 9.4, 6.6 Hz, 1H), 2.16 – 2.10 (m, 2H), 1.78 (s, 3H) ppm;

^{13}C NMR (126 MHz, $CDCl_3$): δ_C 173.0, 151.0, 148.8, 140.5, 132.7, 128.3, 126.9, 125.9, 56.6, 51.9, 48.2, 47.9, 43.9, 42.2, 41.6, 14.9 ppm.

HRMS (ESI+): m/z calc'd for $C_{18}H_{20}NO_2S$ [M+H]+: 321.09307, found: 321.09185.

Methyl-3-phenyl-2,3,3a,8b-tetrahydro-1H-1,3-methanocyclopenta[b]benzofuran-1-carboxylate (5ac)

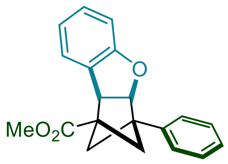

5ac

Synthesized following **General Procedure B** using: BCB **1a** (37.7 mg, 0.200 mmol, 1.00 equiv) and benzofuran **4ac** (110 µL, 1.00 mmol, 5.00 equiv). Purified by flash column chromatography (SiO_2; 100:0 to 90:10 pentane:EtOAc) to afford **5ac** as a single diastereomer (15.3 mg, 0.0499 mmol, 25%) as a colourless oil.

TLC: R_f = 0.44 (90:10 pentane:EtOAc).

NMR Spectroscopy (*see spectra*):

^{1}H NMR (400 MHz, $CDCl_3$): δ_H 7.38 – 7.32 (m, 2H), 7.31 – 7.27 (m, 1H), 7.16 – 7.07 (m, 3H), 6.82 (d, *J* =

8.1 Hz, 1H), 6.70 (td, J = 7.4, 1.0 Hz, 1H), 6.53 (d, J = 7.4 Hz, 1H), 5.58 (dd, J = 7.6, 1.4 Hz, 1H), 4.02 (d, J = 7.6 Hz, 1H), 3.80 (s, 3H), 2.21 – 2.12 (m, 3H), 2.08 – 1.96 (m, 1H) ppm;

^{13}C NMR (126 MHz, $CDCl_3$): δ_C 171.3, 163.3, 139.8, 129.0, 128.5, 127.1, 126.3, 125.7, 125.4, 120.4, 109.5, 88.0, 55.1, 55.0, 54.8, 52.2, 47.8, 35.4 ppm.

HRMS (ESI$^+$): m/z calc'd for $C_{20}H_{18}O_3$ [M+Na]$^+$: 329.11463, found: 329.11482.

4-(*tert*-butyl) 1-methyl-3-phenyl-2,3,3a,8b-tetrahydro-1,3-methanocyclopenta[b]indole-1,4-dicarboxylate (5ad)

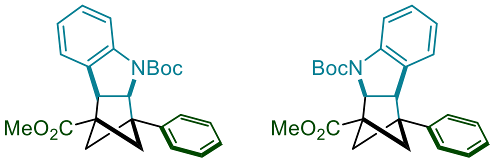

5ad **5ad′**

Synthesized following **General Procedure B** using: BCB **1a** (37.7 mg, 0.200 mmol, 1.00 equiv) and *tert*-butyl 1*H*-indole-1-carboxylate **4ad** (203 µL, 1.00 mmol, 5.00 equiv). Purified by flash column chromatography (SiO_2; 100:0 to 90:10 pentane:EtOAc) to afford a separable 1:1 mix of **5ad** and **5ad′** (60.8 mg, 0.150 mmol, 75%). Both regioisomers were formed as a single diastereomer as a white solid.

TLC: R_f = 0.40 (90:10 pentane:EtOAc).

5ad NMR Spectroscopy (*see spectra*): *Spectra display peak broadening due to the slow rotation of the NBoc C–N bond.*

^{1}H NMR (400 MHz, $CDCl_3$): δ_H 7.87 (br. s, 1H), 7.35 – 7.28 (m, 2H), 7.25 – 7.19 (m, 3H), 7.17 – 7.11 (m, 2H), 6.97 (td, J = 7.5, 1.1 Hz, 1H), 4.86 (d, J = 8.2 Hz, 1H), 4.38 (d, J = 8.2 Hz, 1H), 3.77 (s, 3H), 2.26 (dd, J = 9.1, 7.5 Hz, 1H), 2.11 (d, J = 6.8 Hz, 1H), 1.98 – 1.87 (m, 2H), 1.06 (br. s, 9H) ppm;

^{13}C NMR (126 MHz, $CDCl_3$): δ_C 171.8, 153.0, 146.5, 141.4, 129.8, 128.5, 128.3, 126.5, 126.0, 124.8, 122.7, 115.4, 80.8, 68.7, 56.8, 52.4, 51.8, 50.6, 46.8, 35.9, 27.7 ppm.

HRMS (ESI$^+$): m/z calc'd for $C_{25}H_{27}NO_4Na$ [M+Na]$^+$: 428.18323, found: 428.18322.

5ad′ NMR Spectroscopy (*see spectra*): *Spectra display peak broadening due to the slow rotation of the NBoc C–N bond.*

^{1}H NMR (500 MHz, $CDCl_3$): δ_H 7.77 (br. s, 1H), 7.37 – 7.27 (m, 3H), 7.16 (t, J = 7.9 Hz, 1H), 7.09 – 7.05 (m, 2H), 6.75 (t, J = 7.4 Hz, 1H), 6.35 (d, J = 7.4 Hz, 1H), 5.14 (d, J = 8.5 Hz, 1H), 4.01 (d, J = 8.5 Hz, 1H), 3.75 (s, 3H), 2.27 (dd, J = 8.5, 7.2 Hz, 1H), 2.01 (d, J = 7.2 Hz, 1H), 1.93 – 1.83 (m, 2H), 1.57 (s, 9H) ppm;

^{13}C NMR (126 MHz, $CDCl_3$): δ_C 172.6, 153.0, 145.4, 139.9, 129.6, 128.4, 128.3, 127.0, 126.6, 125.1, 122.1, 114.9, 81.9, 68.0, 54.0, 53.7, 52.8, 52.0, 47.6, 35.2, 28.5 ppm.

HRMS (ESI$^+$): m/z calc'd for $C_{25}H_{27}NO_4Na$ [M+Na]$^+$: 428.18323, found: 428.18321.

Methyl-3-((4-methoxyphenyl)ethynyl)-4-phenylbicyclo[2.1.1]hexane-1-carboxylate (5ae)

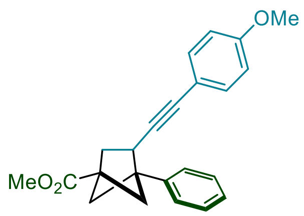

5ae

Synthesized following **General Procedure B** using: BCB **1a** (37.7 mg, 0.200 mmol, 1.00 equiv) and 1-(but-3-en-1-yn-1-yl)-4-methoxybenzene **4ae** (158 mg, 1.00 mmol, 5.00 equiv). Purified by flash column chromatography (SiO_2; 100:0 to 93:7 pentane:EtOAc) to afford **5ae** (27.0 mg, 0.0779 mmol, 39%) as a yellow oil.

TLC: R_f = 0.45 (95:5 pentane:EtOAc).

NMR Spectroscopy (*see spectra*):

^{1}**H NMR** (400 MHz, $CDCl_3$): δ_H 7.37 – 7.30 (m, 4H), 7.28 – 7.22 (m, 3H), 6.81 – 6.76 (m, 2H), 3.78 (s, 3H), 3.74 (s, 3H), 3.21 (ddd, *J* = 8.9, 3.9, 1.9 Hz, 1H), 2.58 (ddd, *J* = 11.2, 8.9, 2.5 Hz, 1H), 2.40 (dd, *J* = 9.4, 6.9 Hz, 1H), 2.25 (ddd, *J* = 10.9, 3.9, 2.9 Hz, 1H), 2.16 (ddd, *J* = 11.2, 6.9, 2.5 Hz, 2H), 1.98 (dd, *J* = 9.4, 6.8 Hz, 1H) ppm;

^{13}**C NMR** (101 MHz, $CDCl_3$): δ_C 173.1, 159.3, 140.9, 133.0, 128.2, 126.9, 126.4, 116.0, 113.9, 90.2, 83.0, 55.4, 55.2, 51.8, 48.4, 46.4, 42.9, 40.1, 37.6 ppm.

HRMS (ESI^+): m/z calc'd for $C_{23}H_{22}O_3Na$ $[M+Na]^+$: 369.1461, found: 369.1461.

Methyl-3-(5-(1,3-dioxoisoindolin-2-yl)pent-1-yn-1-yl)-4-phenylbicyclo[2.1.1]hexane-1-carboxylate (5af)

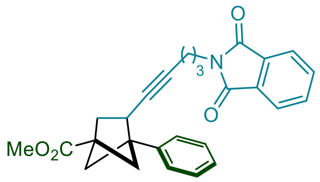

5af

Synthesized following **General Procedure B** using: BCB **1a** (37.7 mg, 0.200 mmol, 1.00 equiv) and 2-(hept-6-en-4-yn-1-yl)isoindoline-1,3-dione **4af** (239 mg, 2.00 mmol, 10.0 equiv). Purified by flash column chromatography (SiO_2; 95:5 to 85:15 pentane:EtOAc) to afford **5af** (33.9 mg, 0.0793 mmol, 40%) as a yellow oil.

TLC: R_f = 0.33 (80:20 pentane:EtOAc).

NMR Spectroscopy (*see spectra*):

^{1}H NMR (400 MHz, $CDCl_3$): δ_H 7.86 – 7.80 (m, 2H), 7.73 – 7.67 (m, 2H), 7.34 – 7.18 (m, 5H), 3.71 (s, 3H), 3.68 (td, J = 7.0, 1.4 Hz, 2H), 2.95 – 2.89 (m, 1H), 2.42 (ddd, J = 11.2, 8.9, 2.4 Hz, 1H), 2.29 (dd, J = 9.4, 6.8 Hz, 1H), 2.18 (td, J = 7.0, 2.4 Hz, 2H), 2.12 – 2.01 (m, 3H), 1.87 (dd, J = 9.4, 6.8 Hz, 1H), 1.80 (p, J = 7.0 Hz, 2H) ppm;

^{13}C NMR (101 MHz, $CDCl_3$): δ_C 173.1, 168.4, 141.0, 134.0, 132.3, 128.1, 126.7, 126.4, 123.3, 82.8, 81.5, 54.9, 51.8, 48.3, 46.7, 42.6, 39.9, 37.4, 36.9, 27.9, 16.6 ppm.

HRMS (ESI$^+$): m/z calc'd for $C_{27}H_{25}NO_4Na$ [M+Na]$^+$: 450.1676, found: 450.1675.

Methyl-3-methyl-4-phenyl-3-vinylbicyclo[2.1.1]hexane-1-carboxylate (5ag)

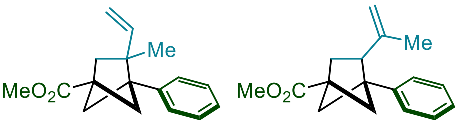

5ag **5ag′**

Synthesized following **General Procedure B** using: BCB **1a** (37.7 mg, 0.200 mmol, 1.00 equiv) and isoprene **4ag** (100 µL, 1.00 mmol, 5.00 equiv). Purified by flash column chromatography (SiO_2; 100:0 to 96:4 pentane:EtOAc) to afford **5ag** and **5ag′** as a 4:1 mix of inseparable regioisomers (30.8 mg, 0.120 mmol, 60%) as a colourless oil.

TLC: R_f = 0.49 (96:4 pentane:EtOAc).

NMR Spectroscopy (*see spectra*): *Only peaks corresponding to **5ag** (major regioisomer) are given.*

^{1}H NMR (500 MHz, $CDCl_3$): δ_H 7.30 – 7.26 (m, 2H), 7.23 – 7.20 (m, 1H), 7.06 – 7.03 (m, 2H), 5.86 (dd, J = 17.5, 10.8 Hz, 1H), 5.07 (dd, J = 10.8, 1.2 Hz, 1H), 4.98 (dd, J = 17.5, 1.2 Hz, 1H), 3.71 (s, 3H), 2.42 – 2.37 (m, 2H), 2.28 (dd, J = 9.6, 6.6 Hz, 1H), 2.05 (dd, J = 6.8, 2.7 Hz, 1H), 1.95 (dd, J = 6.6, 2.8 Hz, 1H), 1.90 (dd, J = 11.2, 2.8 Hz, 1H), 1.12 (s, 3H) ppm;

^{13}C NMR (126 MHz, $CDCl_3$): δ_C 173.8, 143.9, 140.5, 127.7, 127.2, 126.4, 113.8, 57.5, 51.7, 47.4, 47.0, 44.2, 43.9, 42.1, 23.0 ppm.

HRMS (ESI$^+$): m/z calc'd for $C_{17}H_{20}O_2Na$ [M+Na]$^+$: 279.13555, found: 428.18321.

Methyl-7-phenylhexahydro-5,7-methanocyclopenta[b]pyran-5(2H)-carboxylate (5ah)

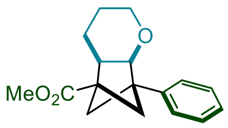

5ah

Synthesized following **General Procedure B** using: BCB **1a** (37.7 mg, 0.200 mmol, 1.00 equiv) and 3,4-dihydro-2*H*-pyran **4ah** (91.2 µL, 1.00 mmol, 5.00 equiv). Purified by flash column chromatography (SiO_2; 100:0 to 85:15 pentane:EtOAc) to afford **5ah** as a single diastereomer (30.0 mg, 0.110 mmol, 55%) as a white solid.

TLC: R_f = 0.42 (85:15 pentane:EtOAc).

NMR Spectroscopy (*see spectra*):

^{1}**H NMR** (500 MHz, $CDCl_3$): δ_H 7.33 – 7.29 (m, 2H), 7.27 – 7.25 (m, 2H), 7.24 – 7.19 (m, 1H), 3.94 – 3.87 (m, 2H), 3.71 (s, 3H), 3.58 (ddd, *J* = 11.2, 8.9, 4.0 Hz, 1H), 2.53 (dd, *J* = 9.3, 7.0 Hz, 1H), 2.43 – 2.32 (m, 1H), 2.12 – 2.02 (m, 2H), 1.96 – 1.84 (m, 2H), 1.82 – 1.76 (m, 1H), 1.57 – 1.36 (m, 2H) ppm;

^{13}**C NMR** (126 MHz, $CDCl_3$): δ_C 173.0, 140.6, 128.3, 126.8, 126.6, 78.9, 63.3, 54.8, 51.7, 51.2, 45.8, 44.4, 37.7, 20.9, 18.5 ppm.

HRMS (ESI^+): m/z calc'd for $C_{17}H_{20}O_3Na$ $[M+Na]^+$: 295.13047 found: 295.13045.

Methyl-3-(2-oxooxazolidin-3-yl)-4-phenylbicyclo[2.1.1]hexane-1-carboxylate (5ai)

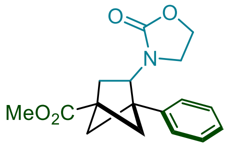

5ai

Synthesized following **General Procedure B** using: BCB **1a** (37.7 mg, 0.200 mmol, 1.00 equiv) and 3-vinyloxazolidin-2-one **4ai** (113 mg, 1.00 mmol, 5.00 equiv). Purified by flash column chromatography (SiO_2; 100:0 to 70:30 pentane:EtOAc) to afford **5ai** (25.3 mg, 0.0840 mmol, 42%) as a pale yellow oil.

TLC: R_f = 0.33 (70:30 pentane:EtOAc).

NMR Spectroscopy (*see spectra*):

^{1}**H NMR** (500 MHz, $CDCl_3$): δ_H 7.37 – 7.32 (m, 2H), 7.30 – 7.22 (m, 3H), 4.82 (dd, *J* = 8.8, 4.3 Hz, 1H), 4.20 (td, *J* = 8.8, 6.9 Hz, 1H), 4.08 (td, *J* = 8.8, 6.4 Hz, 1H), 3.74 (s, 3H), 3.57 (ddd, *J* = 9.4, 8.1, 6.4 Hz, 1H), 3.31 (ddd, *J* = 9.6, 8.1, 6.9 Hz, 1H), 2.57 (ddd, *J* = 11.5, 8.8, 2.6 Hz, 1H), 2.39 (dt, *J* = 7.4, 2.1 Hz, 1H), 2.23 – 2.11 (m, 2H), 2.06 (dd, *J* = 6.9, 3.4 Hz, 1H), 1.99 (dd, *J* = 9.6, 6.9 Hz, 1H) ppm;

^{13}C NMR (126 MHz, $CDCl_3$): δ_C 172.5, 158.6, 138.9, 128.8, 127.4, 126.3, 61.9, 56.9, 54.7, 52.0, 49.2, 47.5, 42.2, 40.9, 35.0 ppm.

HRMS (ESI^+): m/z calc'd for $C_{17}H_{19}NO_4Na$ $[M+Na]^+$: 324.12063 found: 324.12040.

N-methoxy-N-methyl-3,4-diphenylbicyclo[2.1.1]hexane-1-carboxamide (5ak)

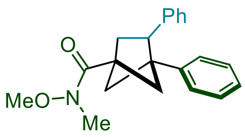

5ak

Synthesized following **General Procedure B** using: BCB **1ak** (43.5 mg, 0.200 mmol, 1.00 equiv) and styrene **4a** (115 µL, 1.00 mmol, 1.00 equiv). Purified by flash column chromatography (SiO_2; 99:1 to 70:30 pentane:EtOAc) to afford **5ak** (38.1 mg, 0.119 mmol, 59%) as a colourless oil.

TLC: R_f = 0.24 (70:30 pentane:EtOAc).

NMR Spectroscopy (*see spectra*):

^{1}H NMR (600 MHz, $CDCl_3$): δ_H 7.20 – 7.10 (m, 6H), 7.02 – 6.97 (m, 2H), 6.88 – 6.85 (m, 2H), 3.76 (s, 3H), 3.62 – 3.55 (m, 1H), 3.26 (s, 3H), 2.61 (ddd, *J* = 11.4, 9.1, 2.4 Hz, 1H), 2.51 – 2.44 (m, 2H), 2.30 (dd, *J* = 9.1, 6.5 Hz, 1H), 2.17 (dd, *J* = 6.5, 2.9 Hz, 1H), 2.01 (dt, *J* = 7.2, 2.1 Hz, 1H) ppm;

^{13}C NMR (151 MHz, $CDCl_3$): δ_C 174.6, 141.7, 141.6, 128.7, 128.0, 127.8, 126.3, 126.2, 126.2, 61.7, 55.2, 50.8, 49.8, 49.4, 40.4, 37.9, 32.7 ppm.

HRMS (ESI^+): m/z calc'd for $C_{21}H_{23}NO_2Na$ $[M+Na]^+$: 344.16210, found: 344.16192

Methyl-3-phenyl-4-(o-tolyl)bicyclo[2.1.1]hexane-1-carboxylate (5al)

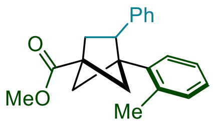

5al

Synthesized following **General Procedure B** using: BCB **1al** (40.4 mg, 0.200 mmol, 1.00 equiv) and styrene **4a** (115 µL, 1.00 mmol, 1.00 equiv). Purified by flash column chromatography (SiO_2; 100:0 to 96:4 pentane:EtOAc) to afford **5al** (41.7 mg, 0.136 mmol, 68%) as a colourless oil.

TLC: R_f = 0.41 (95:5 pentane:EtOAc).

NMR Spectroscopy (*see spectra*):

^{1}**H NMR** (500 MHz, $CDCl_3$): δ_H 7.11 (dd, *J* = 5.1, 2.0 Hz, 3H), 7.04 (td, *J* = 7.4, 1.4 Hz, 1H), 7.00 – 6.89 (m, 4H), 6.69 – 6.63 (m, 1H), 3.77 (d, *J* = 0.8 Hz, 3H), 3.65 (dt, *J* = 9.6, 3.0 Hz, 1H), 2.58 (ddd, *J* = 11.5, 4.5, 2.9 Hz, 1H), 2.49 (ddd, *J* = 11.5, 8.8, 2.5 Hz, 1H), 2.42 (dd, *J* = 9.4, 6.9 Hz, 1H), 2.35 (dd, *J* = 9.4, 6.3 Hz, 1H), 2.26 (dd, *J* = 6.3, 2.9 Hz, 1H), 2.02 (ddd, *J* = 6.9, 2.5, 1.6 Hz, 1H), 1.94 (s, 3H) ppm;

^{13}**C NMR** (101 MHz, $CDCl_3$): δ_C 173.8, 140.8, 139.4, 136.3, 130.6, 128.5, 127.8, 127.4, 126.6, 126.4, 125.5, 57.5, 51.8, 49.6, 49.5, 48.0, 41.2, 35.7, 20.0 ppm.

HRMS (ESI$^+$): m/z calc'd for $C_{21}H_{22}O_2Na$ $[M+Na]^+$: 329.15120, found: 329.15119.

Methyl-4-(4-fluorophenyl)-3-phenylbicyclo[2.1.1]hexane-1-carboxylate (5am)

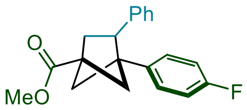

5am

Synthesized following **General Procedure B** using: BCB **1am** (41.2 mg, 0.200 mmol, 1.00 equiv) and styrene **4a** (115 µL, 1.00 mmol, 5.00 equiv). Purified by flash column chromatography (SiO_2; 100:0 to 95:5 pentane:EtOAc) to afford **5am** (41.0 mg, 0.132 mmol, 66%) as a colourless oil.

TLC: R_f = 0.45 (95:5 pentane:EtOAc).

NMR Spectroscopy (*see spectra*):

^{1}**H NMR** (400 MHz, $CDCl_3$): δ_H 7.19 – 7.12 (m, 3H), 7.00 – 6.96 (m, 2H), 6.89 – 6.82 (m, 2H), 6.80 – 6.75 (m, 2H), 3.75 (s, 3H), 3.52 (dd, *J* = 9.0, 4.5 Hz, 1H), 2.58 (ddd, *J* = 11.6, 9.0, 2.4 Hz, 1H), 2.45 (ddd, *J* = 11.6, 4.6, 2.4 Hz, 1H), 2.38 – 2.29 (m, 1H), 2.20 – 2.15 (m, 2H), 2.01 (ddd, *J* = 7.0, 2.4, 1.7 Hz, 1H) ppm;

^{13}**C NMR** (126 MHz, $CDCl_3$): δ_C 173.6, 161.5, 141.1, 137.3, 128.7, 128.0, 127.7, 126.4, 114.8, 55.7, 51.8, 50.6, 49.3, 48.1, 40.3, 37.1 ppm;

^{19}**F NMR** (376 MHz, $CDCl_3$): δ_F –116.5 ppm.

HRMS (ESI$^+$): m/z calc'd for $C_{20}H_{19}FO_2Na$ $[M+Na]^+$: 333.12613 found: 333.12606.

Methyl-3-phenyl-4-(m-tolyl)bicyclo[2.1.1]hexane-1-carboxylate (5ap)

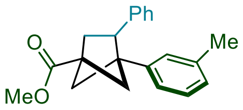

5ap

Synthesized following **General Procedure B** using: BCB **1ap** (40.4 mg, 0.200 mmol, 1.00 equiv) and styrene

4a (115 µL, 1.00 mmol, 1.00 equiv). Purified by flash column chromatography (SiO_2; 100:0 to 96:4 pentane:EtOAc) to afford **5ap** (38.7 mg, 0.126 mmol, 63%) as a colourless oil.

TLC: R_f = 0.42 (95:5 pentane:EtOAc).

NMR Spectroscopy (*see spectra*):

^{1}H NMR (600 MHz, $CDCl_3$): δ_H 7.19 – 7.10 (m, 3H), 7.06 (t, *J* = 7.6 Hz, 1H), 7.02 – 6.98 (m, 2H), 6.95 (ddt, *J* = 7.6, 1.8, 1.0 Hz, 1H), 6.67 (tt, *J* = 1.8, 0.7 Hz, 1H), 6.65 (dddd, *J* = 7.6, 1.8, 1.2, 0.7 Hz, 1H), 3.76 (s, 3H), 3.58 (ddd, *J* = 9.1, 4.6, 1.6 Hz, 1H), 2.59 (ddd, *J* = 11.5, 9.0, 2.5 Hz, 1H), 2.44 (ddd, *J* = 11.5, 4.6, 2.3 Hz, 1H), 2.34 (tt, *J* = 7.3, 3.3 Hz, 1H), 2.23 (d, *J* = 0.8 Hz, 3H), 2.21 – 2.16 (m, 2H), 2.05 (ddd, *J* = 7.0, 2.5, 1.7 Hz, 1H) ppm;

^{13}C NMR (151 MHz, $CDCl_3$): δ_C 173.8, 141.5, 141.4, 137.5, 128.7, 127.9, 127.8, 127.1, 127.0, 126.2, 123.3, 56.2, 51.8, 50.3, 49.5, 48.2, 40.2, 37.4, 21.5 ppm.

HRMS (ESI$^+$): m/z calc'd for $C_{21}H_{22}O_2Na$ $[M+Na]^+$: 329.15120, found: 329.15121.

Methyl-4-(3-methoxyphenyl)-3-phenylbicyclo[2.1.1]hexane-1-carboxylate (5aq)

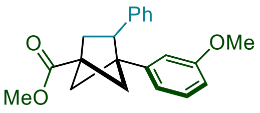

5aq

Synthesized following **General Procedure B** using: BCB **1aq** (43.7 mg, 0.200 mmol, 1.00 equiv) and styrene **4a** (115 µL, 1.00 mmol, 1.00 equiv). Purified by flash column chromatography (SiO_2; 100:0 to 96:4 pentane:EtOAc) to afford **5aq** (41.3 mg, 0.128 mmol, 64%) as a colourless oil.

TLC: R_f = 0.37 (95:5 pentane:EtOAc).

NMR Spectroscopy (*see spectra*):

^{1}H NMR (600 MHz, $CDCl_3$): δ_H 7.19 – 7.11 (m, 3H), 7.09 (dd, *J* = 8.2, 7.6 Hz, 1H), 7.03 – 6.98 (m, 2H), 6.68 (ddd, *J* = 8.2, 2.6, 1.0 Hz, 1H), 6.48 (ddd, *J* = 7.6, 1.6, 1.0 Hz, 1H), 6.33 (dd, *J* = 2.6, 1.6 Hz, 1H), 3.75 (s, 3H), 3.64 (s, 3H), 3.58 – 3.54 (m, 1H), 2.59 (ddd, *J* = 11.5, 9.0, 2.5 Hz, 1H), 2.47 – 2.41 (m, 1H), 2.37 – 2.31 (m, 1H), 2.20 – 2.14 (m, 2H), 2.03 (ddd, *J* = 7.0, 2.5, 1.7 Hz, 1H) ppm;

^{13}C NMR (151 MHz, $CDCl_3$): δ_C 173.7, 159.3, 143.2, 141.5, 129.0, 128.7, 127.9, 126.3, 118.6, 112.0, 111.9, 56.3, 55.2, 51.8, 50.5, 49.3, 48.1, 40.3, 37.3 ppm.

HRMS (ESI$^+$): m/z calc'd for $C_{21}H_{22}O_3$ $[M+Na]^+$: 345.14612, found: 345.14610.

Phenyl-3,4-diphenylbicyclo[2.1.1]hexane-1-carboxylate (5ar)

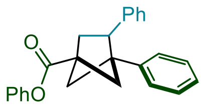

5ar

Synthesized following **General Procedure B** using: BCB **1ar** (50.1 mg, 0.200 mmol, 1.00 equiv) and styrene **4a** (115 µL, 1.00 mmol, 1.00 equiv). Purified by flash column chromatography (SiO_2; 100:0 to 95:5 pentane:EtOAc) to afford **5ar** (41.4 mg, 0.117 mmol, 58%) as a colourless solid.

TLC: R_f = 0.34 (95:5 pentane:EtOAc).

NMR Spectroscopy (*see spectra*):

^{1}**H NMR** (400 MHz, $CDCl_3$): δ_H 7.35 – 7.24 (m, 3H), 7.19 – 7.02 (m, 8H), 6.98 – 6.92 (m, 2H), 6.84 – 6.79 (m, 2H), 3.58 (ddd, *J* = 9.1, 4.6, 1.6 Hz, 1H), 2.67 (ddd, *J* = 11.5, 9.1, 2.5 Hz, 1H), 2.52 (ddd, *J* = 11.5, 4.6, 2.5 Hz, 1H), 2.46 – 2.39 (m, 1H), 2.31 – 2.20 (m, 2H), 2.18 – 2.10 (m, 1H) ppm;

^{13}**C NMR** (101 MHz, $CDCl_3$): δ_C 171.6, 150.9, 141.3, 141.3, 129.6, 128.7, 128.1, 127.9, 126.5, 126.4, 126.2, 125.9, 121.7, 56.4, 50.5, 49.6, 48.4, 40.4, 37.4 ppm.

HRMS (ESI$^+$): m/z calc'd for $C_{25}H_{22}O_2Na$ $[M+Na]^+$: 377.15120, found: 377.15117.

***tert*-Butyl-3,4-diphenylbicyclo[2.1.1]hexane-1-carboxylate (5as)**

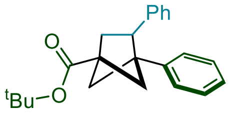

5as

Synthesized following **General Procedure B** using: BCB **1as** (46.1 mg, 0.200 mmol, 1.00 equiv) and styrene **4a** (115 µL, 1.00 mmol, 1.00 equiv). Purified by flash column chromatography (SiO_2; 100:0 to 95:5 pentane:EtOAc) to afford **5as** (36.8 mg, 0.104 mmol, 52%) as a colourless oil.

TLC: R_f = 0.46 (95:5 pentane:EtOAc).

NMR Spectroscopy (*see spectra*):

^{1}**H NMR** (400 MHz, $CDCl_3$): δ_H 7.21 – 7.09 (m, 6H), 7.02 – 6.95 (m, 2H), 6.89 – 6.83 (m, 2H), 3.56 (ddd, *J* = 9.0, 4.6, 1.6 Hz, 1H), 2.55 (ddd, *J* = 11.6, 9.0, 2.5 Hz, 1H), 2.39 (ddd, *J* = 11.6, 4.6, 2.5 Hz, 1H), 2.34 – 2.28 (m, 1H), 2.19 – 2.11 (m, 2H), 2.01 (ddd, *J* = 7.0, 2.5, 1.7 Hz, 1H), 1.49 (s, 9H) ppm;

^{13}**C NMR** (101 MHz, $CDCl_3$): δ_C 172.8, 141.8, 141.7, 128.7, 128.0, 127.8, 126.3, 126.3, 126.2, 80.4, 55.9, 50.6, 49.5, 49.4, 40.0, 37.3, 28.3 ppm.

HRMS (ESI$^+$): m/z calc'd for $C_{23}H_{26}O_2Na$ $[M+Na]^+$: 357.18250 found: 357.18259.

4-(3-Fluoro-4-methylphenyl)-N-methoxy-N-methyl-3-phenylbicyclo[2.1.1]hexane-1-carboxamide (5at)

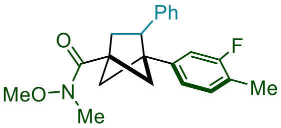

5at

Synthesized following **General Procedure B** using: BCB **1at** (49.9 mg, 0.200 mmol, 1.00 equiv) and styrene **4a** (115 µL, 1.00 mmol, 1.00 equiv). Purified by flash column chromatography (SiO_2; 90:0 to 60:40 pentane:EtOAc) to afford **5at** (36.1 mg, 0.102 mmol, 51%) as a colourless oil.

TLC: R_f = 0.48 (60:40 pentane:EtOAc).

NMR Spectroscopy (*see spectra*):

^{1}H NMR (600 MHz, $CDCl_3$): δ_H 7.20 – 7.12 (m, 3H), 7.04 – 6.93 (m, 3H), 6.54 – 6.49 (m, 2H), 3.75 (s, 3H), 3.55 (dd, *J* = 9.1, 4.5 Hz, 1H), 3.25 (s, 3H), 2.59 (ddd, *J* = 11.3, 9.1, 2.4 Hz, 1H), 2.49 – 2.39 (m, 2H), 2.24 (dd, *J* = 9.4, 6.5 Hz, 1H), 2.19 (d, *J* = 1.8 Hz, 3H), 2.11 (dd, *J* = 6.5, 2.9 Hz, 1H), 1.99 – 1.94 (m, 1H) ppm;

^{13}C NMR (126 MHz, $CDCl_3$): δ_C 174.4, 161.1 (d, *J* = 244.4 Hz), 141.6 (d, *J* = 7.3 Hz), 141.5, 131.0 (d, *J* = 5.5 Hz), 128.6, 128.0, 126.37, 122.5 (d, *J* = 17.2 Hz), 121.8 (d, *J* = 3.1 Hz), 113.0 (d, *J* = 22.1 Hz), 61.7, 54.7, 50.6, 50.0, 49.3, 40.5, 38.0, 32.7, 14.4 ppm;

^{19}F NMR (376 MHz, $CDCl_3$): δ_F –118.3 ppm.

HRMS (ESI$^+$): m/z calc'd for $C_{22}H_{24}NFO_2Na$ $[M+Na]^+$: 376.16833, found: 376.16823.

N-methoxy-N-methyl-1-phenyl-1,2,3a,4,5,9b-hexahydro-3H-1,3-methanocyclopenta[a]naphthalene-3-carboxamide (5au)

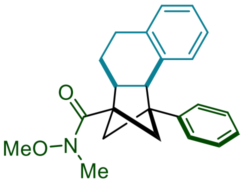

5au

Synthesized following **General Procedure B** using: BCB **1ak** (43.4 mg, 0.200 mmol, 1.00 equiv) and 1,2-dihydronaphthalene **4p** (131 µL, 1.00 mmol, 5.00 equiv). Purified by flash column chromatography (SiO_2; 100:0 to 70:30 pentane:EtOAc) to afford **5au** as a single diastereomer (34.7 mg, 0.100 mmol, 50%) as a colourless solid.

TLC: R_f = 0.26 (70:30 pentane:EtOAc).

NMR Spectroscopy (*see spectra*):

^{1}H NMR (400 MHz, $CDCl_3$): δ_H 7.33 – 7.18 (m, 3H), 7.08 (dd, *J* = 7.6, 1.5 Hz, 1H), 7.04 – 6.98 (m, 3H), 6.76 (td, *J* = 7.6, 1.5 Hz, 1H), 6.22 (d, *J* = 7.8 Hz, 1H), 3.71 (s, 3H), 3.49 (d, *J* = 8.7 Hz, 1H), 3.25 (s, 3H), 3.16 – 3.08 (m, 1H), 2.92 (ddd, *J* = 15.5, 9.6, 4.9 Hz, 1H), 2.61 (dt, *J* = 15.5, 5.6 Hz, 1H), 2.35 (dd, *J* = 9.6, 6.3 Hz, 1H), 2.09 (dd, *J* = 10.2, 6.9 Hz, 2H), 1.95 – 1.77 (m, 3H) ppm;

^{13}C NMR (101 MHz, $CDCl_3$): δ_C 142.8, 139.3, 137.1, 129.8, 128.6, 128.2, 126.8, 126.4, 125.8, 125.2, 61.6, 55.5, 53.7, 51.4, 47.1, 43.2, 38.1, 32.7, 28.8, 24.8 ppm. *Carbonyl carbon not observed due to peak broadening.*

HRMS (ESI$^+$): m/z calc'd for $C_{23}H_{25}NO_2Na$ [M+Na]$^+$: 370.17775 found: 370.17771.

1-3,4-Diphenylbicyclo[2.1.1]hexan-1-yl)pentan-1-one (5av)

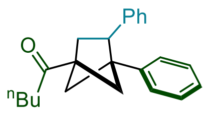

5av

Synthesized following **General Procedure B** using: BCB **1av** (42.9 mg, 0.200 mmol, 1.00 equiv) and styrene **4a** (115 µL, 1.00 mmol, 5.00 equiv). Purified by flash column chromatography (SiO_2; 100:0 to 96:4 pentane:EtOAc) to afford **5av** (50.9 mg, 0.160 mmol, 80%) as a white solid.

TLC: R_f = 0.37 (96:4 pentane:EtOAc).

NMR Spectroscopy (*see spectra*):

^{1}H NMR (500 MHz, $CDCl_3$): δ_H 7.20 – 7.12 (m, 6H), 7.01 – 6.96 (m, 2H), 6.89 – 6.87 (m, 2H), 3.61 (dd, *J* = 9.2, 4.4 Hz, 1H), 2.59 – 2.52 (m, 3H), 2.41 – 2.35 (m, 2H), 2.20 (dd, *J* = 9.2, 6.4 Hz, 1H), 2.12 (dd, *J* = 6.4, 3.0 Hz, 1H), 1.99 (ddd, *J* = 7.1, 2.6, 1.7 Hz, 1H), 1.68 – 1.60 (m, 2H), 1.40 – 1.31 (m, 2H), 0.94 (t, *J* = 7.4 Hz, 3H) ppm;

^{13}C NMR (126 MHz, $CDCl_3$): δ_C 211.8, 141.5, 141.4, 128.6, 128.0, 127.9, 126.4, 126.3, 126.2, 55.8, 55.7, 50.6, 49.2, 39.89, 38.8, 37.6, 25.7, 22.6, 14.1 ppm.

HRMS (ESI$^+$): m/z calc'd for $C_{23}H_{26}ONa$ [M+Na]$^+$: 341.18759 found: 341.18746.

3,4-Diphenylbicyclo[2.1.1]hexan-1-yl)(phenyl)methanone (5aw)

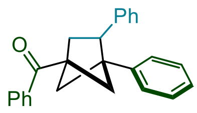

5aw

Synthesized following **General Procedure B** using: BCB **1aw** (46.9 mg, 0.200 mmol, 1.00 equiv) and styrene **4a** (115 µL, 1.00 mmol, 5.00 equiv). Purified by flash column chromatography (SiO_2; 100:0 to 94:6 pentane:EtOAc) to afford **5aw** as a 1:1 mix of regioisomers (27.1 mg, 0.0801 mmol, 40%) as a colourless oil.

TLC: R_f = 0.49 (94:6 pentane:EtOAc).

NMR Spectroscopy (see spectra): *in cases where the regioisomer peaks could be distinguished they are designated as r^1 and r^2. ^{13}C signals relating to the same carbon are given together.*

^{1}**H NMR** (500 MHz, $CDCl_3$): δ_H 8.02 – 7.98 (m, 1H, r^1), 7.74 – 7.66 (m, 1H, r^2), 7.62 – 7.55 (m, 0.5H, r^1), 7.53 – 7.46 (m, 1.5H), 7.41 – 7.30 (m, 3H), 7.30 – 7.23 (m, 1H), 7.23 – 7.12 (m, 5H), 7.08 – 7.00 (m, 1H), 6.96 – 6.89 (m, 1H), 4.13 (dd, J = 9.2, 3.9 Hz, 0.5H, r^1), 3.74 (dd, J = 9.3, 3.8 Hz, 0.5H, r^1), 2.75 (ddd, J = 11.5, 9.2, 2.5 Hz, 0.5H, r^1), 2.69 – 2.63 (m, 1H), 2.61 – 2.46 (m, 2H), 2.45 – 2.36 (m, 1H), 2.32 (dd, J = 6.6, 2.9 Hz, 0.5H, r^2), 2.24 – 2.16 (m, 1H) ppm;

^{13}**C NMR** (126 MHz, $CDCl_3$): δ_C 202.2 and 202.1, 142.4 and 141.6, 141.4, 137.3 and 136.6, 133.1 and 132.7, 128.8 and 128.1, 128.6 and 128.6, 128.6 and 128.5, 128.5 and 128.4, 128.0, 126.7 and 126.7, 126.4 and 126.4, 126.3 and 126.1, 61.1 and 49.9, 55.5 and 55.3, 52.1 and 51.2, 50.9 and 50.5, 41.7 and 40.2, 41.4 and 41.4 ppm.

HRMS (ESI$^+$): m/z calc'd for $C_{25}H_{22}ONa$ $[M+Na]^+$: 361.15629 found: 361.15617.

2,4-Diphenylbicyclo[2.1.1]hexan-1-yl)(furan-2-yl)methanone (5ax)

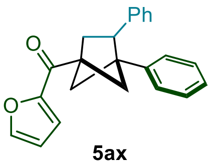

5ax

Synthesized following **General Procedure B** using: BCB **1ax** (44.9 mg, 0.200 mmol, 1.00 equiv) and styrene **4a** (115 µL, 1.00 mmol, 5.00 equiv). Purified by flash column chromatography (SiO_2; 100:0 to 93:7 pentane:EtOAc) to afford **5ax** as a 2:1 mix of regioisomers (42.9 mg, 0.131 mmol, 65%) as a white solid.

TLC: R_f = 0.40 (93:7 pentane:EtOAc).

NMR Spectroscopy (see spectra): *in cases where the regioisomer peaks could be distinguished they are designated as r^1 and r^2. ^{13}C signals relating to the same carbon are given together.*

^{1}**H NMR** (400 MHz, $CDCl_3$): δ_H 7.63 (d, J = 1.4 Hz, 0.67H, r^1), 7.50 (d, J = 1.4 Hz, 0.33H, r^2), 7.38 – 7.30 (m, 1.33H), 7.29 – 7.26 (m, 0.67H), 7.23 – 7.12 (m, 6H), 7.05 – 7.01 (m, 1.67H), 6.93 – 6.86 (m, 1.33H), 6.58 (dd, J = 3.6, 1.7 Hz, 0.67H, r^1), 6.45 (dd, J = 3.6, 1.7 Hz, 0.33H, r^2), 4.21 (dd, J = 9.2, 4.4 Hz, 0.33H, r^2), 3.69 (dd, J = 8.2, 3.8 Hz, 0.67H, r^1), 2.74 (ddd, J = 11.4, 9.0, 2.5 Hz, 0.67H, r^1), 2.67 – 2.58 (m, 1.33H), 2.49 – 2.33 (m, 2H), 2.29 (dt, J = 6.1, 3.0 Hz, 1H), 2.24 – 2.19 (m, 0.33H, r^2), 2.15 (dt, J = 7.3, 2.1 Hz, 0.67H, r^1) ppm;

^{13}**C NMR** (126 MHz, $CDCl_3$): δ_C 190.5 and 190.2, 152.9 and 152.7, 146.3 and 146.1, 142.5 and 141.5,

141.9 and 141.5, 128.7 and 127.9, 128.5 and 128.1, 128.5 and 127.9, 126.7 and 126.4, 126.6 and 126.4, 126.3 and 126.1, 117.7 and 117.7, 112.3 and 112.2, 59.6 and 56.1, 54.1 and 51.0, 51.0 and 50.20, 49.8 and 49.7, 41.7 and 40.6, 41.0 and 39.2 ppm.

HRMS (ESI+): m/z calc'd for $C_{23}H_{20}O_2Na$ $[M+Na]^+$: 351.13553 found: 351.13555.

Methyl-3-cyclopropyl-3,4-diphenylbicyclo[2.1.1]hexane-1-carboxylate (5ay)

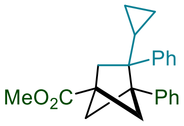

5ay

Synthesized following **General Procedure B** using: BCB **1a** (37.7 mg, 0.200 mmol, 1.00 equiv) and (1-cyclopropylvinyl)benzene **4ay** (144 mg, 1.00 mmol, 5.00 equiv). Purified by flash column chromatography (SiO_2; 100:0 to 96:4 pentane:EtOAc) to afford **5ay** (27.3 mg, 0.0821 mmol, 41%) as a white solid.

TLC: R_f = 0.35 (96:4 pentane:EtOAc).

NMR Spectroscopy (*see spectra*):

^{1}H NMR (500 MHz, $CDCl_3$): δ_H 7.30 – 7.24 (m, 3H), 7.13 – 7.10 (m, 3H), 7.07 – 7.05 (m, 2H), 7.00 – 6.96 (m, 2H), 3.72 (s, 3H), 2.80 (dd, *J* = 9.5, 6.8 Hz, 1H), 2.73 (dd, *J* = 11.8, 2.8 Hz, 1H), 2.08 (dd, *J* = 9.5, 6.8 Hz, 1H), 2.05 – 1.94 (m, 3H), 1.42 (tt, *J* = 8.5, 5.7 Hz, 1H), 0.69 (dddd, *J* = 9.3, 8.2, 6.0, 4.8 Hz, 1H), 0.57 (dq, *J* = 9.3, 5.3 Hz, 1H), 0.36 – 0.24 (m, 1H), −0.16 (dtd, *J* = 9.3, 6.0, 4.8 Hz, 1H) ppm;

^{13}C NMR (126 MHz, $CDCl_3$): δ_C 173.7, 144.6, 142.2, 128.8, 127.9, 127.7, 127.2, 126.5, 125.9, 59.5, 52.6, 51.8, 47.4, 45.3, 44.6, 36.8, 18.9, 5.0, 1.4 ppm.

HRMS (ESI+): m/z calc'd for $C_{23}H_{24}O_2Na$ $[M+Na]^+$: 355.16685 found: 355.16683.

2.5. Reaction Optimization for Non-Activated Alkenes

Reactions were performed using **1a** (0.05 mmol) according to a modified **General Procedure A**. Modifications to standard conditions and key observations from each study are stated.

2.5.1. Establishing the photocatalyst

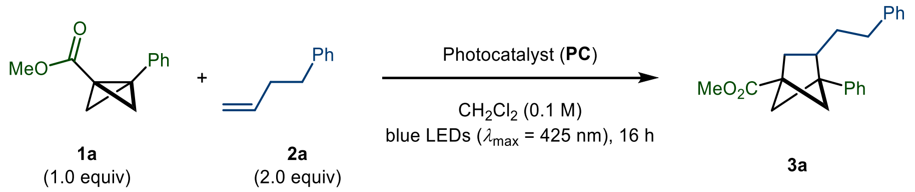

Table S1: Establishing the photocatalyst

entry	PC (mol%)	$E_{1/2}$ (PC*/PC•-) (V)	% Yield[a]
**1**	$[Ru(bpy)_3]Cl_2$ (2 mol%)	0.77	n.d.
**2**	[Ir-F] (2 mol%)	1.21	n.d.
**3**	4CzIPN (10 mol%)	1.43	n.d.
**4**	OMe-thioxanthylium (10 mol%)	1.86	<5
**5**	**$[Mes_2Acr^tBu_2]ClO_4$ (10 mol%)**	**2.00**	**20**
**6**	Triphenylpyrylium BF_4 (10 mol%)	2.55	n.d.

[a] Yields were determined by ^1H NMR analysis using dibromomethane as an internal standard.

Key observations: Upon screening photocatalysts across a wide range of oxidation potentials, it was demonstrated that $[Mes_2Acr^tBu_2]ClO_4$ was capable of efficiently accessing the desired BCH product (entry 5). It was observed that employing photocatalysts that display an excited state oxidation potential below 1.86 V or greater than 2.00 V failed to deliver any observable product. This is presumably due to an inability to effectively activate the BCB fragment (1.79 V vs Ag/AgCl) or a result of the undesired oxidation of the alkene hindering reactivity.

2.5.2. Solvent and alkene stoichiometry

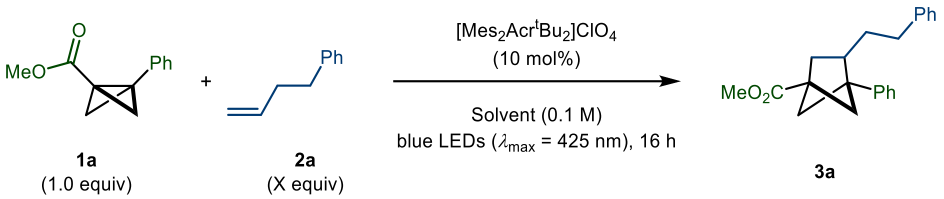

Table S2: Optimization of solvent and alkene stoichiometry

entry	Solvent	2a (X equiv)	% Yield[a]
**1**	CH_2Cl_2	2	20
**2**	CH_2Cl_2	5	38
**3**	CH_2Cl_2	10	54
**4**	MeCN	1	10
**5**	MeCN	2	19
**6**	MeCN	5	28
**7**	MeCN	10	39
**8**	**$MeNO_2$**	**10**	**65**
9	$MeNO_2$	20	67
10	$MeNO_2$	0.25	44

[a] Yields were determined by ^{1}H NMR analysis using dibromomethane as an internal standard.

Key observations: Increasing the equivalents of alkene was found to be beneficial to the reaction outcome. $MeNO_2$ was determined to be the optimal solvent. When increasing the equivalents further (entry 9), only a small improvement to the yield was observed so 10 equivalents of alkene was taken forward. As a large excess of alkene is required, we also demonstrated that inverting the stoichiometry of this reaction, to have the olefin as the limiting reagent, could still provide the desired product in an acceptable yield (entry 10).

2.5.3. Catalyst loading, concentration and wavelength of light

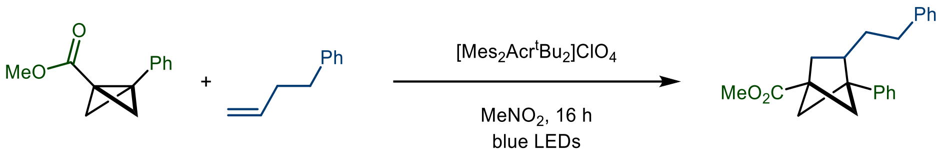

Table S3: Optimization of catalyst loading, concentration and wavelength of light

entry	$[Mes_2Acr^tBu_2]ClO_4$ (mol%)	Concentration (M)	Blue LEDs λ_{max} (nm)	% Yield[a]
**1**	**10**	**0.1**	**425**	**65**
**2**	5	0.1	425	59
**3**	2	0.1	425	52
**4**	20	0.1	425	65
**5**	10	0.2	425	62
**6**	10	0.05	425	64
**7**	10	0.1	405	61
**8**	10	0.1	450	65

[a] Yields were determined by ^1H NMR analysis using dibromomethane as an internal standard.

Key observations: No improvement in the yield could be achieved upon altering the catalyst loading, concentration or the wavelength of light used. Interestingly, decreasing the loading of catalyst had only a minor effect on the yield of product formation (entries 1-3). However, due to the ease of synthesis of the organic photocatalyst that was employed, we proceeded with 10 mol% as the optimal loading.

2.6. Reaction Optimization for Activated Alkenes

Reactions were performed using **1a** (0.05 mmol) according to a modified General Procedure B. Modifications to standard conditions and key observations from each study are stated.

2.6.1. Reaction time

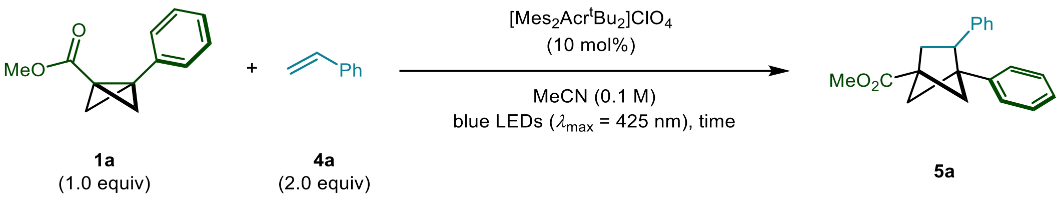

Table S4: Optimization of reaction time

entry	Time (h)	% Yield (RSM)[a]
**1**	**0.5**	**23 (37)**
**2**	3	45 (11)
**3**	16	53 (0)
**4**	48	51 (0)

[a] Yields were determined by ^1H NMR analysis using dibromomethane as an internal standard.

Key observations: After 30 minutes of irradiation, only 63% conversion was observed (entry 1). After 16 h, full BCB consumption could be achieved (entries 2-3). Increasing the reaction time further did not affect the yield (entry 4), indicating that the intended product is stable under the reaction conditions.

2.6.2. Solvent and alkene stoichiometry

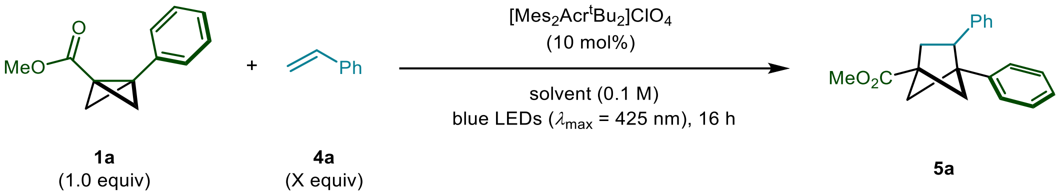

Table S5: Optimization of solvent and alkene stoichiometry

entry	Solvent	4a (X equiv)	% Yield[a]
**1**	MeCN	1	42
**2**	MeCN	2	53
**3**	**MeCN**	**5**	**75**
**4**	MeCN	10	67
**5**	CH_2Cl_2	5	38
**6**	EtOAc	5	**7**
**7**	$PhCF_3$	5	56
**8**	$MeNO_2$	5	71
**9**	Acetone	5	20
**10**	DCE	5	45

[a] Yields were determined by ^{1}H NMR analysis using dibromomethane as an internal standard.

Key observations: Increasing the equivalents of alkene up to 5 was found to be beneficial to the reaction (entries 1-3), however a decrease in yield was observed when increasing the equivalents further (entry 4). Presumably, this is due to the photocatalyst oxidising styrene rather than the BCB compound. Screening a variety of solvents showed that MeCN was optimal for the cycloaddition reaction with styrene (entries 5-10).

2.6.3. Catalyst counterion

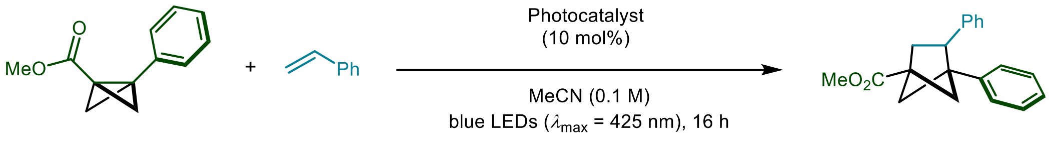

1a (1.0 equiv) **4a** (5.0 equiv) **5a**

Table S6: Effect of catalyst counterion

entry	PC (mol%)	% Yield[a]
**1**	**[$Mes_2Acr^tBu_2$]ClO_4**	**75**
**2**	[$Mes_2Acr^tBu_2$]BF_4	74
**3**	[$Mes_2Acr^tBu_2$]OTf	71
**4**	[Mes_2Acr]ClO_4	32

[a] Yields were determined by ^{1}H NMR analysis using dibromomethane as an internal standard.

Key observations: Changing the counterion of the photocatalyst had little effect on the yield of the desired product and so these photocatalysts could be used interchangeably (entries 1-3). However, removing the ^{t}Bu groups from the catalyst was found to be significantly deleterious to the outcome of the reaction (entry 4).

2.7. Substrate Limitations

Table S7: Non-activated alkene substrate limitations in the [2π+2σ] cycloaddition reaction

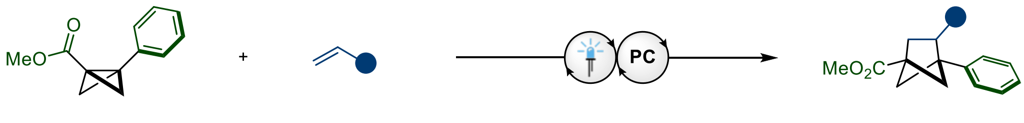

1a (1.0 equiv) (10 equiv) **3**

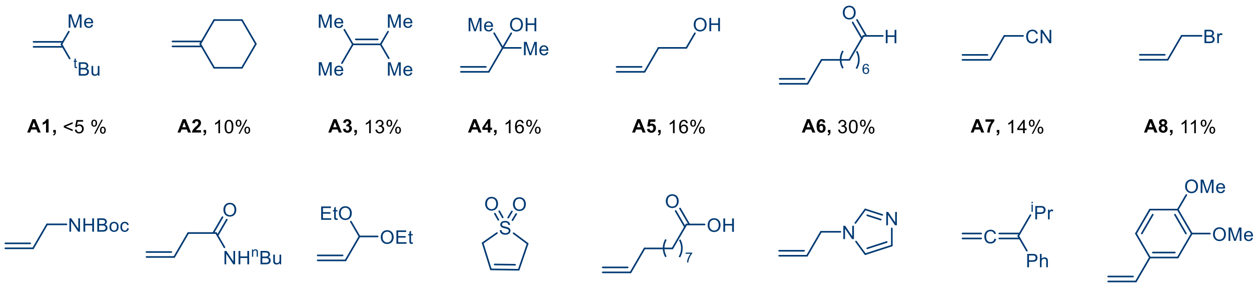

A1, <5 % **A2,** 10% **A3,** 13% **A4,** 16% **A5,** 16% **A6,** 30% **A7,** 14% **A8,** 11%

A9, 0% **A10,** 6% **A11,** 9% **A12,** <5% **A13,** 46% **A14,** <5% **A15,** 15% **A16,** 0%

Reaction conditions: **1a** (0.2 mmol), alkene (2.0 mmol), [$Mes_2Acr^tBu_2$]ClO_4 (10 mol%), $MeNO_2$ (0.1 M), blue LEDs (λ_{max} = 425 nm), rt, 16 h. Yields given were determined by ^{1}H NMR analysis of the crude reaction using CH_2Br_2 as an internal standard. For the cases in which >20% yield was observed by quantitative ^{1}H NMR analysis, isolation was attempted but unsuccessful due to an inability to separate impurities from the desired product.

Table S8: Activated alkene substrate limitations in the [2π+2σ] cycloaddition reaction

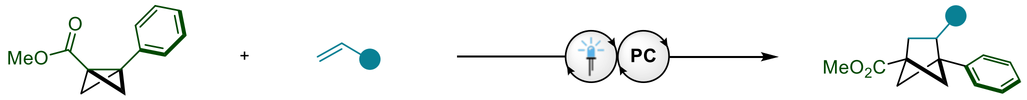

1a (1.0 equiv) + (5.0 equiv) → **5**

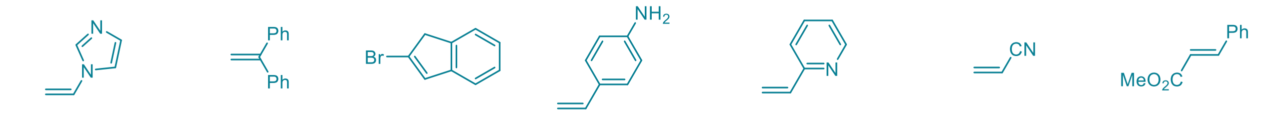

A17, 15% (44%) **A18,** 20% **A19,** 0% **A20,** 0% (83%) **A21,** 0% (32%) **A22,** 0% **A23,** 0%

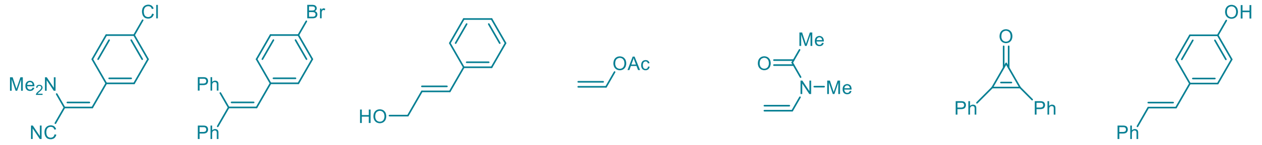

A24, 0% (99%) **A25,** 0% **A26,** 15% **A27,** 0% **A28,** 0% **A29,** 0% **A30,** 0%

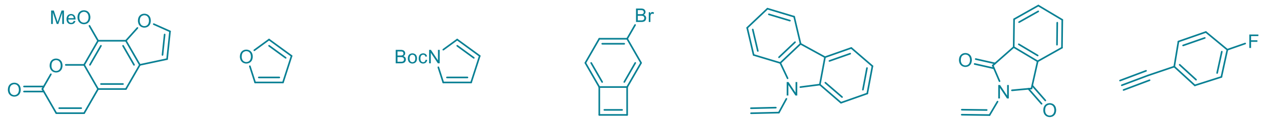

A31, 0% **A32,** 0% **A33,** 0% **A34,** 0% **A35,** 16% **A36,** 27% **A37,** 0%

Insoluble alkenes[a]

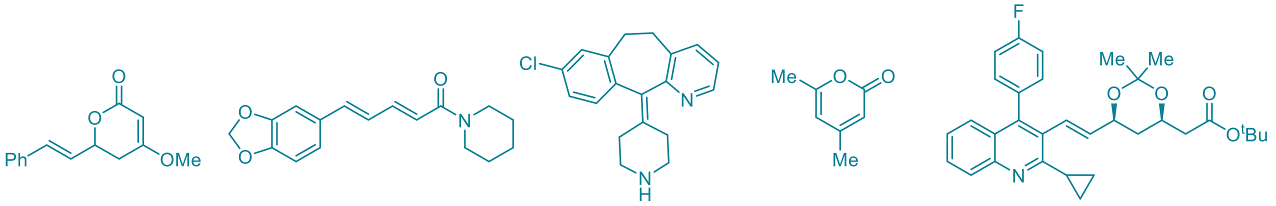

A38, 0% **A39,** 0% **A40,** 0% **A41,** 0% **A42,** 0%

Reaction conditions: **1a** (0.2 mmol), alkene (1.0 mmol), [$Mes_2Acr^tBu_2$]ClO_4 (10 mol%), MeCN (0.1 M), blue LEDs (λ_{max} = 425 nm), rt, 16 h. Yields given were determined by ^{1}H NMR analysis of the crude reaction using CH_2Br_2 as an internal standard. Value in brackets indicates the returned BCB starting material that was observed. [a] Insoluble alkenes showed no signs of reactivity and fully returned the BCB starting material.

Table S9: BCB substrate limitations in the [2π+2σ] cycloaddition reaction

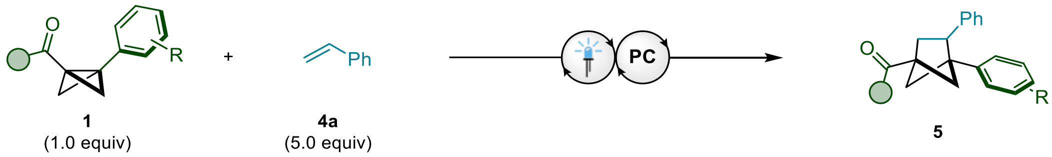

1 (1.0 equiv) + **4a** (5.0 equiv) → **5**

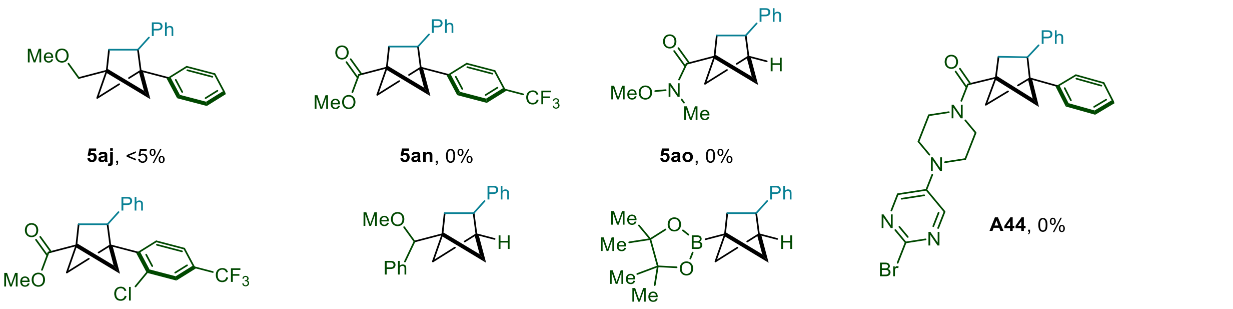

5aj, <5%; **5an**, 0%; **5ao**, 0%; **A44**, 0%; **A45**, 0%; **A46**, 0%; **A47**, 0%

Reaction conditions: **1** (0.2 mmol), **4a** (1.0 mmol), [$Mes_2Acr^tBu_2$]ClO_4 (10 mol%), MeCN (0.1 M), blue LEDs (λ_{max} = 425 nm), rt, 16 h. Yields given were determined by ^{1}H NMR analysis of the crude reaction using CH_2Br_2 as an internal standard.

2.8. Inversion of Reaction Stoichiometry

Table S10: Inversion of reaction stoichiometry in the [2π+2σ] cycloaddition reaction

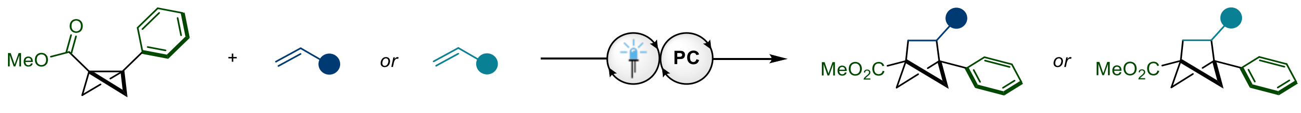

1a + **2** (1.0 equiv) or **4** (1.0 equiv) → **3** or **5**

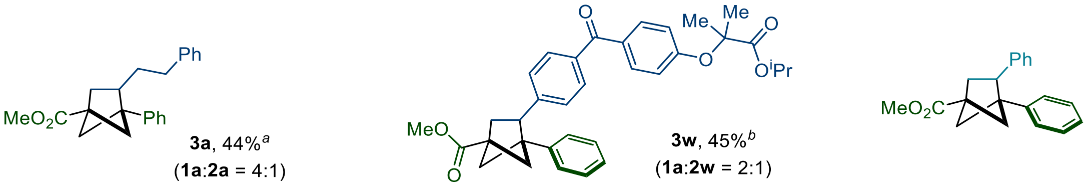

3a, 44%[a] (**1a**:**2a** = 4:1); **3w**, 45%[b] (**1a**:**2w** = 2:1); **5a**, 54%[b] (**1a**:**4a** = 2:1)

[a]Using reaction conditions from **General Procedure A** with a 4:1 ratio of **1a** to alkene. [b]Using reaction conditions from **General Procedure B** with a 2:1 ratio of **1a** to alkene. Yields given were determined by ^{1}H NMR analysis of the crude reaction using CH_2Br_2 as an internal standard.

2.9. Sensitivity Screen

The sensitivity assessment was conducted in a similar manner as reported by Glorius and coworkers[19] using conditions modified from **General Procedure A**. During the sensitivity screen a reaction with unmodified conditions was performed and the yield obtained (61%) was taken as the benchmark.

Table S11: Sensitivity assessment of the [2π+2σ] cycloaddition reaction

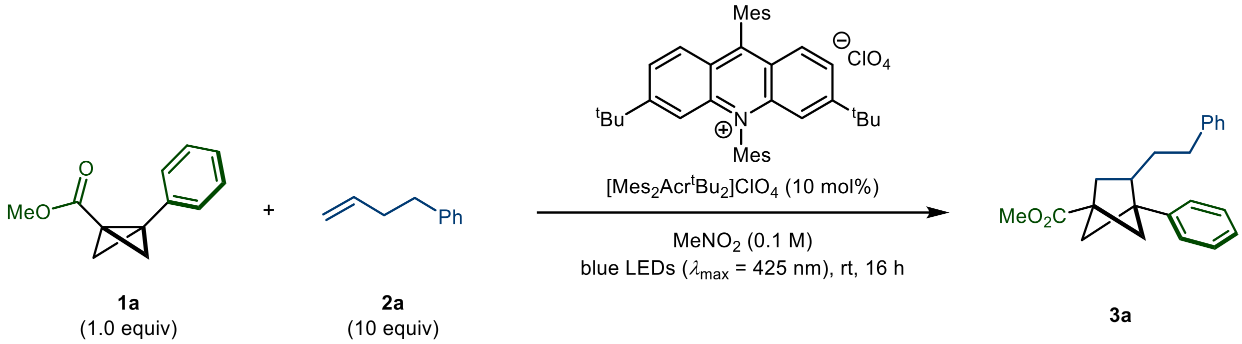

Entry	Modification	Deviation from standard conditions	Yield[a]	Deviation from benchmark
1	high H_2O	H_2O (20 µL)	47%	-14%
2	high O_2	air	54%	-7%
3	medium O_2	bubbling Ar for 10 s	61%	0%
4	low concentration	3 mL $MeNO_2$	61%	0%
5	high concentration	1 mL $MeNO_2$	61%	0%
6	high intensity	2x LEDs	58%	-3%
7	low intensity	20 cm distance to LED	61%	0%
8	big scale	standard scale x20	53%[b]	-8%
9	low temperature	fan on	57%	-4%
10	high temperature	fan off	61%	0%

[a] Yields were determined by ^1H NMR analysis using dibromomethane as an internal standard. [b] Isolated yield.

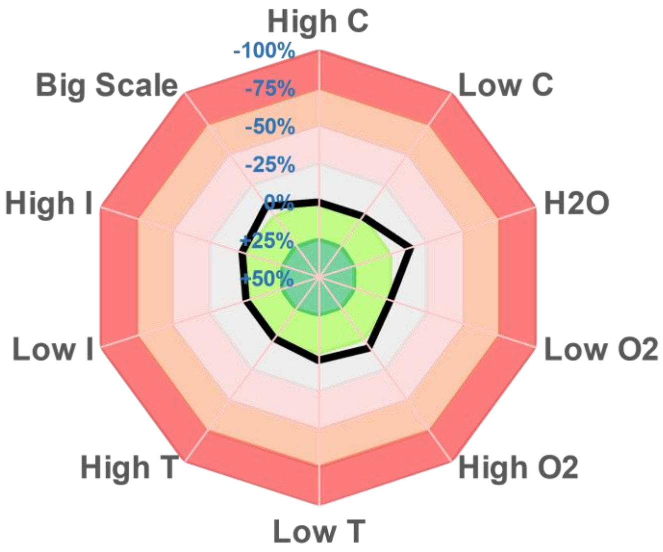

Figure S5: Radar diagram representation of sensitivity screen

3. MECHANISTIC INVESTIGATIONS

3.1. UV/vis Absorption Spectroscopy

UV/vis absorption spectra were recorded on a Jasco V-730 spectrophotometer, equipped with a temperature control unit at 25 °C. The samples were measured in Starna® fluorescence quartz cuvettes (type: 29-F, chamber volume = 1.400 mL, H × W × D = 48 mm × 12.5 mm × 12.5 mm, path length = 10 mm).

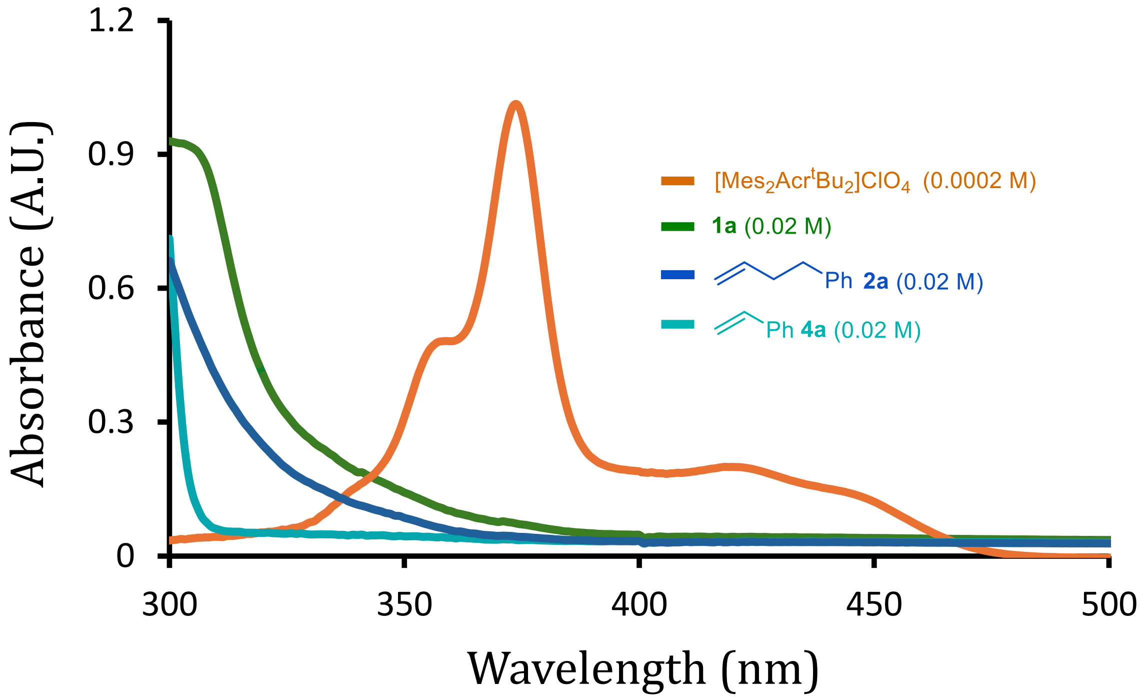

Figure S6: UV/vis absorption spectra of BCB (1a), [$Mes_2Acr^tBu_2$]ClO_4 and the alkenes 2a and 4a in MeCN

UV/vis spectroscopy of the individual reaction components revealed that the photocatalyst [$Mes_2Acr^tBu_2$]ClO_4 is the only light absorbing species near $\lambda = 425$ nm, eliminating the possibility that direct excitation of either BCB **1a** or the alkenes are responsible for reactivity.

3.2. Stern-Volmer Analysis

Quenching studies were carried out on a JASCO FP-8300 spectrofluorometer using Starna® fluorescence quartz cuvettes (type: 29-F, chamber volume = 1.400 mL, H × W × D = 48 mm × 12.5 mm × 12.5 mm, path length = 10 mm). The following parameters were set: data interval = 0.5 nm, scan-speed = 500 nm/min, excitation wavelength λ_{ex} = 420 nm, measured luminescence wavelength λ = 472 nm. All samples were prepared in an argon-filled glovebox with degassed and dry MeCN. The quenching studies were performed using a solution of [$Mes_2Acr^tBu_2$]ClO_4 ($1 \cdot 10^{-4}$ M). The varying concentrations of the potential quencher were achieved by dilution of the respective stock solutions in the cuvettes. The samples were sealed with PTFE stoppers and removed from the glovebox for the measurement.

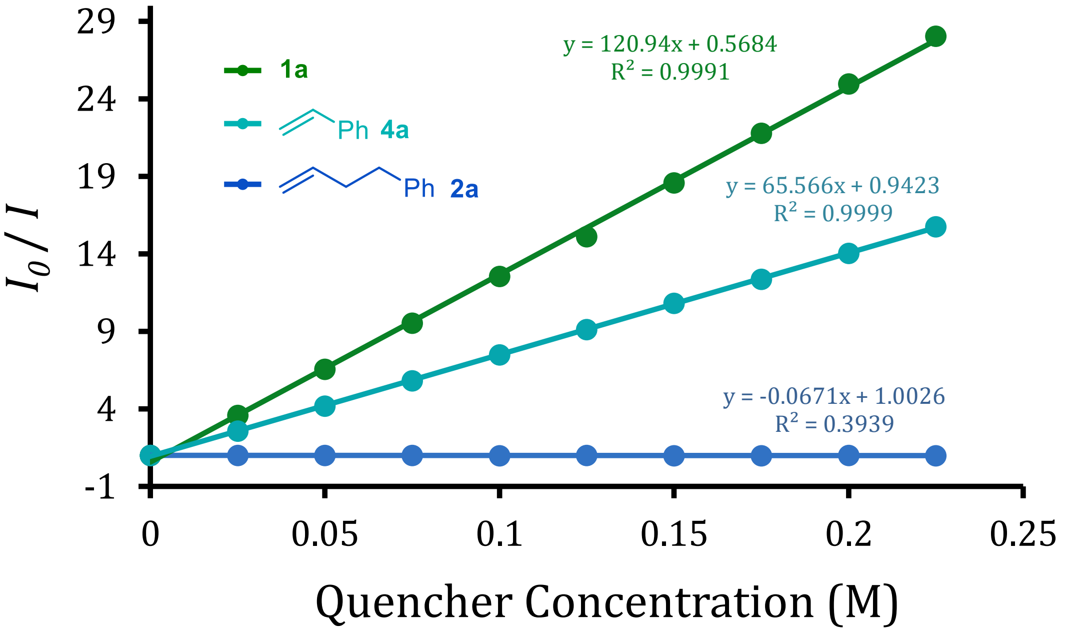

Figure S7: Luminescence quenching of the excited state of [Mes$_2$Acr^{t}Bu$_2$]ClO$_4$ using BCB 1a, 2a and 4a in MeCN

Stern–Volmer quenching studies clearly demonstrated that BCB **1a** is an effective quencher of the photocatalyst excited state, whereas alkene **2a** gave no indication that it can interact with this excited state species. However, quenching was detected, albeit to a lesser extent than for **1a**, upon the addition of styrene (**4a**).

3.3. Cyclic Voltammetry

3.3.1. Redox potential measurements for 1a, 2a and 4a

Cyclic voltammograms (CVs) were collected at room temperature using a Metrohm Dropsens µStat-i 400s potentiostat. A 2 mm glassy carbon disc electrode, an Ag/AgCl (2 M LiCl in ethanol) electrode, and a platinum sheet electrode were used as the working, reference, and counter electrodes, respectively and were all supplied by Metrohm. The electrolyte solution contained 0.1 M tetrabutylammonium hexafluorophosphate (TBAPF$_6$) and 1 mM of the given substrate in MeCN. Before each measurement, the solution was purged with N_2 gas to avoid the interference of atmospheric oxygen. The scan rate was set at 0.1 V/s (E_{step} = 0.002 V) and 10 scans were taken for each compound in the potential window of −2.0 V to +2.5 V.

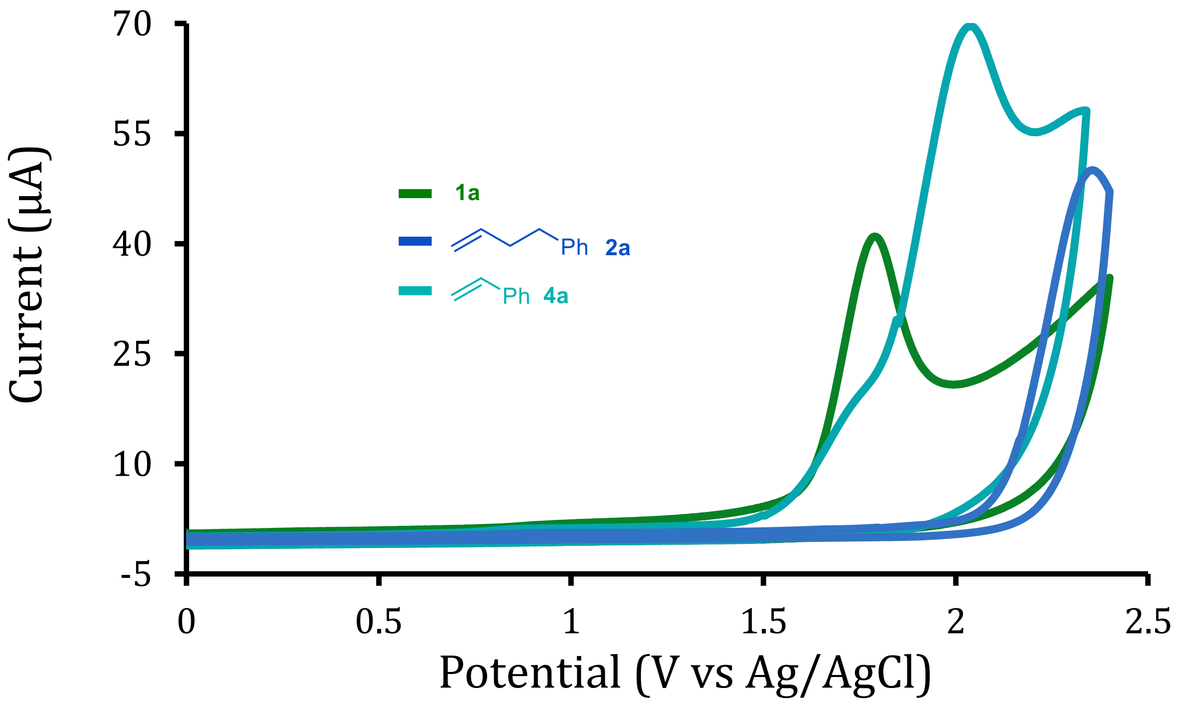

Figure S8: Cyclic voltammetry of 1a, 2a and 4a in 0.1 M $TBAPF_6$ (MeCN), using a 2 mm glassy carbon disk working electrode, Pt sheet counter electrode and an Ag/AgCl (2 M LiCl in ethanol) reference electrode. Set at 0.1 V/s scan rate

3.3.2. Redox potential measurements of BCB substrates

Using the method outlined above, the redox potentials of a selection of alternative BCB compounds (**1**) were analyzed. By correlating the measured oxidation potential with the ability of the substrates to participate in the desired [2π+2σ] cycloaddition reaction, an approximate window of reactivity could be established.

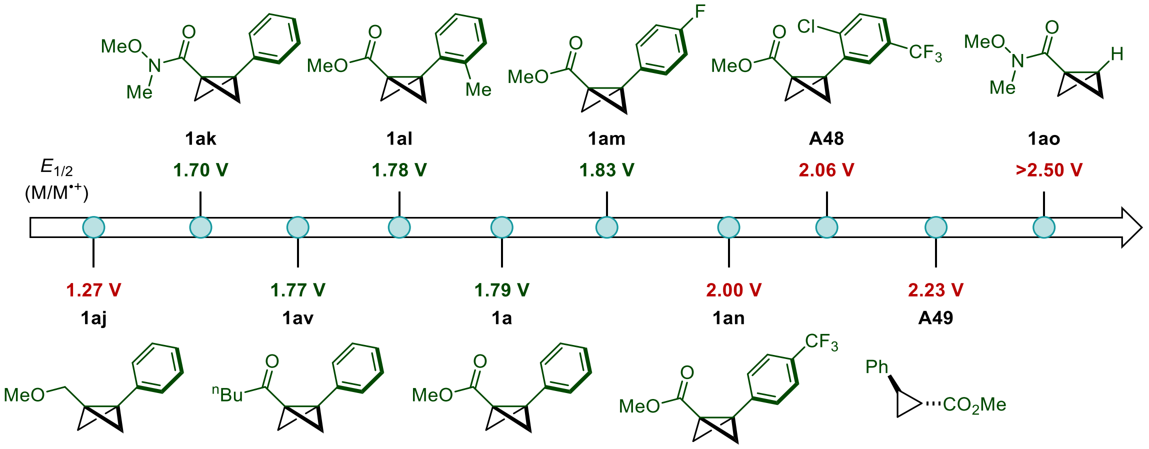

Figure S9: Oxidation potentials of BCB substrates. Green values indicate where the substrate was successful in the [2π+2σ] cycloaddition reaction and red indicates where the substrate was unsuccessful

3.4. Quantum Yield Calculation

3.4.1. Determination of the photon flux

First, the photon flux of a blue LED (3 W, λ_{max} = 420 nm) was determined by standard ferrioxalate actinometry according to a modified literature procedure by Yoon and coworkers.[20] Two solutions were prepared and stored in the dark. All following steps were also carried out in a darkened lab to prevent undesired irradiation.

Solution 1: Potassium ferrioxalate hydrate (737 mg, 1.50 mmol) was dissolved in aq. H_2SO_4 (0.05 M, 10 mL) to afford a 0.15 M ferrioxalate solution (attention: light sensitive!).

Solution 2: 1,10-Phenanthroline monohydrate (20 mg, 0.10 mmol) and NaOAc (4.50 g) were dissolved in aq. H_2SO_4 (0.5 M, 20 mL).

To determine the photon flux, the reduction of $[Fe(C_2O_4)_3]^{3-}$ to $[Fe(C_2O_4)_2]^{2-}$ over time is measured.[21,22] Accordingly, **Solution 1** (1 mL) was irradiated for 60 s at λ_{max} = 420 nm (distance: 5 cm) in a 10 mL Schlenk tube. Subsequently, **Solution 2** (175 µL) was added and the mixture was stirred for 1 h to ensure that all Fe(II)-ions were coordinated by phenanthroline. The absorbance of the solution was then measured at λ = 510 nm. In addition, the absorbance of a non-irradiated control sample was measured. The same procedure was repeated two times. The average absorbance of the three irradiated samples and the three control samples were used to calculate the generated amount of Fe(II) ($n_{Fe(II)}$) according to the Lambert–Beer law (Equation 1), where V is the total volume ($1.175 \cdot 10^{-3}$ L), ΔA(510 nm) is the difference in absorbance between the irradiated and non-irradiated control samples (at λ = 510 nm), l is the path length of the cuvette (1.0 cm), and ε is the molar attenuation coefficient of the ferrioxalate actinometer at λ = 510 nm (11100 $L \cdot mol^{-1} \cdot cm^{-1}$).[21]

$$n_{\mathrm{Fe(II)}} = \frac{\mathrm{V} \bullet \Delta\mathrm{A}(510\ \mathrm{nm})}{\mathrm{l} \bullet \varepsilon} \quad (1)$$

The photonflux (ϕ_q) can be calculated using Equation 2, where ϕ_F is the quantum yield of the ferrioxalate actinometer (1.13 at λ = 392 nm) and t is the irradiation time (60 s).[21,23]

$$\phi_q = \frac{n_{Fe(II)}}{\phi_F \bullet t \bullet f} \quad (2)$$

The fraction of light absorbed at λ = 420 nm by the actinometer (f) is calculated by using Equation 3, where A(420 nm) is the absorbance of **Solution 1** at λ = 420 nm.

$$\mathrm{f} = 1 - 10^{-A(420\ nm)} \quad (3)$$

In this case, the absorbance A(420 nm) of **Solution 1** was > 3, which indicates that > 99.9% of the photons were absorbed (f > 0.999).

Table S12: Determination of the photon flux

	Run 1	Run 2	Run 3	Average
A(510 nm)	2.298	2.249	2.383	2.310
$A_{control}$(510 nm)	0.527	0.574	0.559	0.553
ΔA(510 nm) =	1.757			
ϕ_q =	$2.75 \cdot 10^{-9}$ mol·s^{-1}			
f =	0.999			

3.4.2. Determination of the reaction quantum yield

To benchmark the quantum yield of our [2π+2σ] cycloaddition reaction, the quantum yield of the standard reaction with BCB **1a** and alkene **2a** was determined.

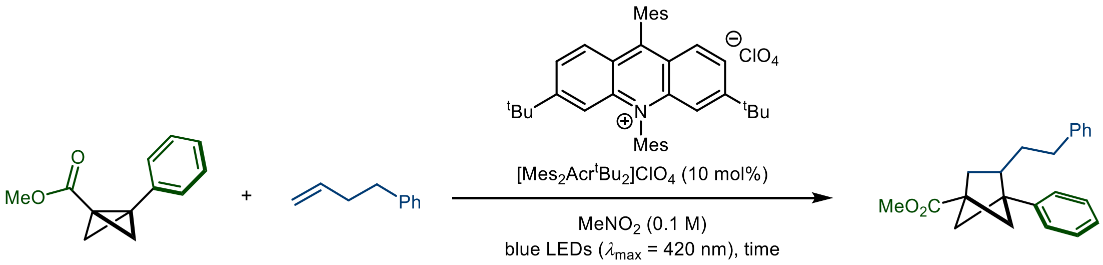

1a (1.0 equiv) **2a** (10 equiv) **3a**

To an oven-dried 10 mL Schlenk tube equipped with a Teflon-coated magnetic stir bar was added [$Mes_2Acr^tBu_2$]ClO_4 (6.3 mg, 10 µmol, 10 mol%), **2a** (0.150 mL, 1.00 mmol, 10.0 equiv), and bicyclo[1.1.0]butane (BCB) **1a** (18.8 mg, 0.100 mmol, 1.00 equiv). The Schlenk tube was evacuated and backfilled with argon three times before $MeNO_2$ (1.0 mL) was added under a positive argon pressure. The reaction mixture was then stirred under irradiation in the calibrated set-up (3 W, λ = 420 nm) for the specified time. After this time, the solvent was removed under reduced pressure and the yield was determined by quantitative NMR spectroscopy using CH_2Br_2 as an internal standard. The quantum yield (ϕ) of the reaction can be calculated using Equation 4, where ϕ_q is the photon flux and t is the irradiation time. The fraction of light absorbed (f_R) by the reaction was determined by measuring the absorbance of a non-irradiated control reaction (Equation 3).

$$\phi = \frac{n_{product}}{\phi_q \bullet t \bullet f_R} \quad (4)$$

Table S13: Determination of the reaction quantum yield

	Experiment 1	Experiment 2	Experiment 3	Experiment 4
Time (s)	300	600	900	1200
$n_{product}$ (mol)	$3.0{\cdot}10^{-6}$	$6.0{\cdot}10^{-6}$	$1.0{\cdot}10^{-5}$	$1.2{\cdot}10^{-5}$
ϕ =	3.64	3.64	4.04	3.64
**ϕ (Average) =**	**3.74**			

3.5. Trapping Experiments

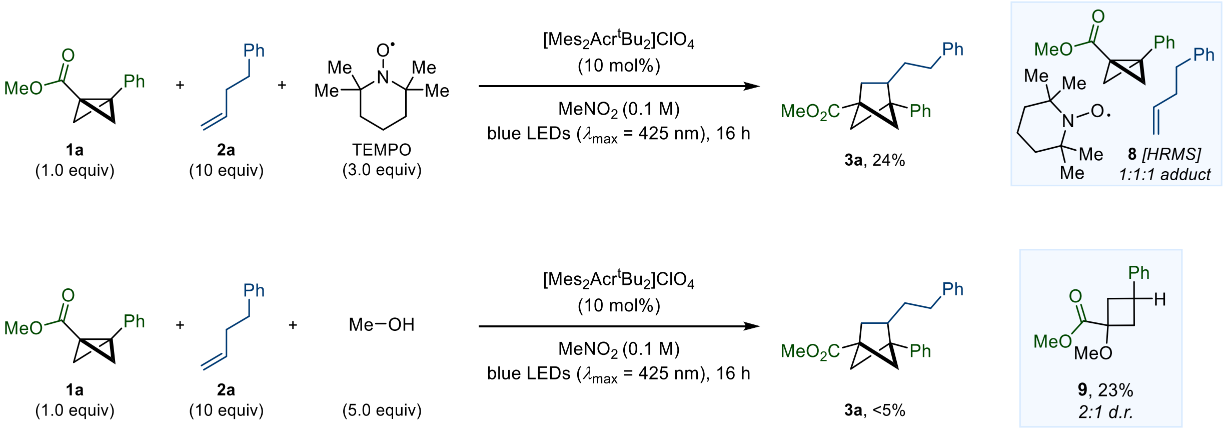

Figure S10: Effect of the trapping agents TEMPO and MeOH on the yield of BCH formation

When the radical trapping agent 2,2,6,6-tetramethylpiperidin-1-yloxyl (TEMPO) was added to the standard reaction, product formation was not entirely suppressed, whereas addition of the nucleophile MeOH resulted in only trace product being observed. In the case of TEMPO, the observation of a 1:1:1 trapping adduct (**8**) by high resolution mass spectrometry (HRMS), suggests that a carbon-centered radical is present in the reaction mechanism. In the case of MeOH, nucleophilic addition product **9** was isolated from the reaction mixture.

8: HRMS (ESI$^+$): m/z calc'd for $C_{31}H_{41}NO_3Na$ [M+Na]$^+$: 498.29787 found: 498.29779.

Methyl-1-methoxy-3-phenylcyclobutane-1-carboxylate (9)

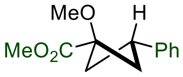

7

Synthesized following a modified General Procedure A using: BCB **1a** (37.7 mg, 0.200 mmol, 1.00 equiv), but-3-en-1-ylbenzene (300 µL, 2.00 mmol, 10.0 equiv) and MeOH (40.5 µL, 1.00 mmol, 5.00 equiv). Purified by flash column chromatography (SiO_2; 100:0 to 92:8 pentane:EtOAc) to afford **9** as a 2:1 mix of diastereomers

(10.1 mg, 0.0459 mmol, 23%) as a colorless oil.

TLC: R_f = 0.41 (92:8 pentane:EtOAc).

NMR Spectroscopy (<u>*see spectra*</u>): *in cases where diastereomer peaks could be distinguished, they are labelled as d^1 and d^2.*

^{1}H NMR (600 MHz, $CDCl_3$): δ_H; 7.35 – 7.29 (m, 2H), 7.30 – 7.25 (m, 2H), 7.24 – 7.20 (m, 1H), 3.86 (s, 1H, d^2), 3.79 (s, 2H, d^1), 3.73 (p, *J* = 9.1 Hz, 0.66H, d^1), 3.42 (p, *J* = 9.1 Hz, 0.33H, d^2), 3.37 (s, 2H, d^1), 3.27 (s, 1H, d^2), 2.95 – 2.87 (m, 0.66H, d^2), 2.71 – 2.58 (m, 2.68H, d^1), 2.39 – 2.31 (m, 0.66H, d^2) ppm.

^{13}C NMR (151 MHz, $CDCl_3$): δ_C (*d^1, major diastereomer)* 173.2, 144.5, 128.5, 126.7, 126.4, 78.8, 53.3, 52.3, 37.1, 33.7 ppm. δ_C (*d^2, minor diastereomer)* 174.3, 144.6, 128.6, 126.7, 126.4, 76.7, 52.5, 52.5, 39.2, 30.6 ppm.

HRMS (ESI$^+$): m/z calc'd for $C_{13}H_{16}O_3Na$ $[M+Na]^+$: 243.09917 found: 243.09921.

3.6. Radical Clock Experiments

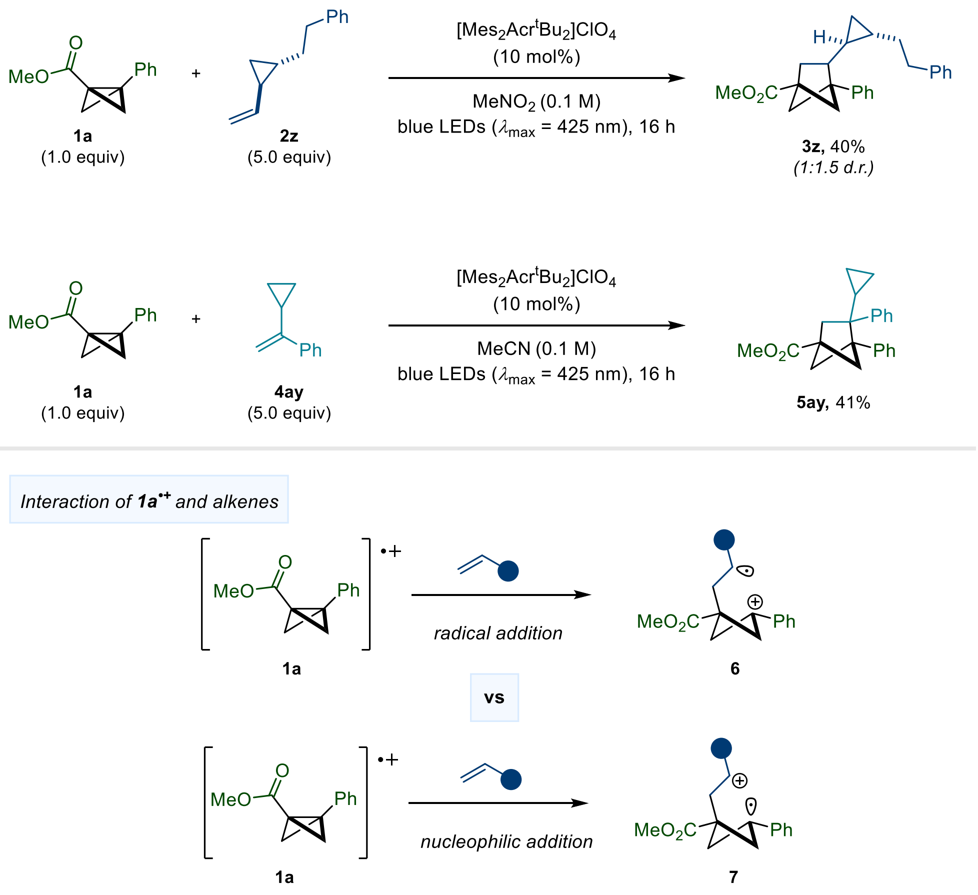

Figure S11: The [2π+2σ] cycloaddition reaction of cyclopropane substrates 2z and 4ay

Upon subjecting cyclopropane-containing alkenes **2z** and **4ay** to standard reaction conditions, no cyclopropane ring opening could be detected in either case and cycloadducts **3z** and **5ay** were isolated in 40% and 41% yield

respectively. From these results it could be suggested that the initial interaction between **1a** and the alkene does not proceed via radical addition and may instead proceed in a fashion more similar to alkene nucleophilic addition. However, it was shown during DFT studies that in the case of **2z**, the initial interaction of the BCB radical cation and the alkene proceeds via a nucleophilic addition-type pathway to give intermediate **7**, whereas the reaction with propene occurs in a manner more similar to radical addition to give intermediate **6** (see Fig. **7**). Additionally, the DFT studies suggested that the final C−C bond formation step in the mechanism (**TS-II**) was found to be a barrierless process, with an estimated free energy of −14.5 kcal/mol using a restrained calculation (see Fig. **7**). These results indicate that the rapidity of the intramolecular ring-closure step would occur at a faster rate than cyclopropane ring-opening.

4. COMPUTATIONAL CALCULATIONS

4.1. Computational Methods

All geometry optimizations were performed in water using the dispersion-corrected ωb97xd[24] functional with the def2SVP[25] basis set. Single point energies were calculated with the ωb97xd functional and the def2TZVPP basis set. Solvation effects were included by performing single point energy calculations with the SMD[26] solvation model for both optimization and single point energy calculations. To obtain more accurate Gibbs free energies and enthalpies, we applied the quasiharmonic approximation developed by Grimme[27] to compute the thermal corrections with a 50 cm^{-1} cut-off frequency. The quasiharmonic approximations were calculated using GoodVibes.[28] Intrinsic reaction coordinate (IRC) calculations were performed to confirm the nature of all transition states. All wavefunctions were checked to be stable. All calculations were performed with Gaussian 16[29] on UCLA Hoffman2 and XSEDE[30] supercomputers.

4.2. Nature of the BCB Radical Cation

We conducted DFT calculations to reveal the nature of the bicyclobutane (BCB) radical cation (Fig. **S12**). We calculated the condensed Hirshfeld charges and spin densities of these radical cation intermediates, in which the charges and spin densities of the hydrogen atoms are condensed on to their connecting heavy atoms. In **1a**$^{•+}$, we identified that the charge and spin density is delocalized across the benzene ring and the bridgehead atoms of the BCB fragment (C1 and C3). The C1 carbon has a higher spin density (0.31), as compared to that on the C3 carbon (0.15). In addition, the strained BCB σ-bond is elongated (1.68 Å) compared to the ground state of **1a** (1.52 Å).

We also calculated the nature of **1a**$^{•+}$ in which the strained BCB σ-bond has been formerly broken (**1a**$^{•+}$ **open**). In this intermediate, we identified that the charge is delocalized over the C3 carbon and the benzene ring, while the spin density is largely localized on the C1 carbon. However, we identified that the BCB radical cation in the “opened” cyclobutane form (**1a**$^{•+}$ **open**) is higher in energy by 1.4 kcal/mol.

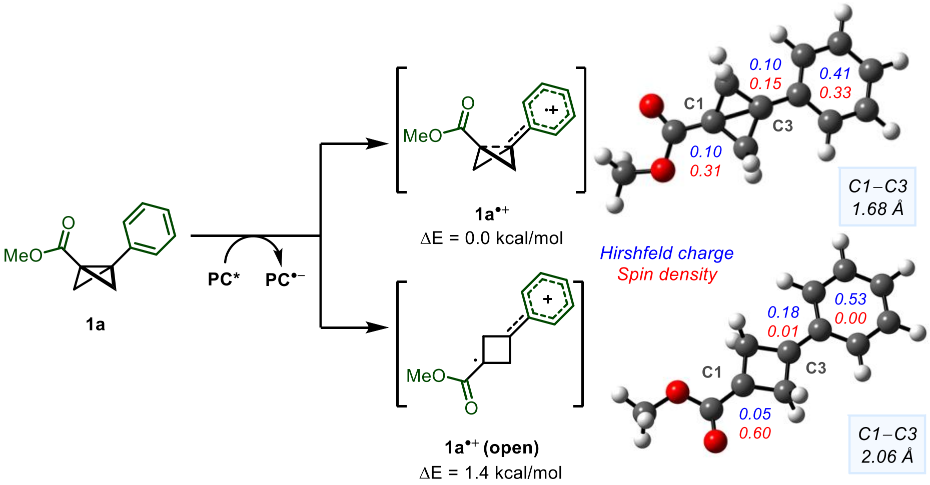

Figure S12: DFT computed condensed Hirshfeld charges and spin densities of 1a$^{•+}$

4.3. Comparison of BCB and Styrene Radical Cations

Upon comparing the spin and charge densities for the BCB and styrene radical cations we observed some considerable similarities (Fig. **S13**). Namely, the spin and charge are effectively delocalized across the benzene ring and the bridgehead/alkene carbons. Additionally, a large proportion of the spin density is localized at the C1/homobenzylic position compared to the benzylic position. From this we concluded that these species might show some levels of similarity with respect to their reactivity.

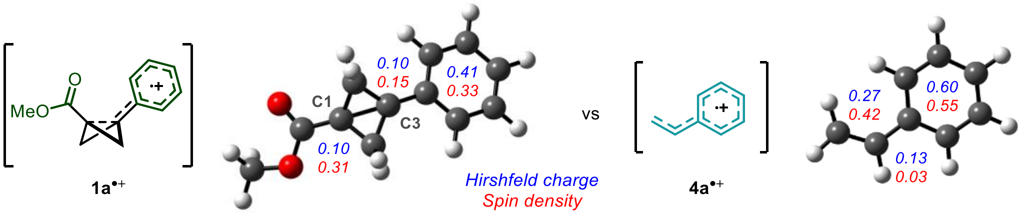

Figure S13: Comparison of condensed Hirshfeld charges and spin densities of 1a•+ and 4a•+

4.4. Computed Reaction Coordinate Profile of BCB Radical Cation (1a•+) with Styrene

Fig. **S14** presents our DFT computed reaction coordinate profile of BCB radical cation (**1a•+**) with styrene (solvent = MeCN). Complexation of the radical cation **1a•+** with styrene to form **IM-SI•+**, is exergonic by 6.8 kcal/mol. Subsequent insertion of the alkene fragment into the BCB scaffold was found to be a kinetically facile process (**TS-SIa**), with a low free energy barrier of 1.2 kcal/mol with respect to the preceding **IM-SI•+**. The free energy barrier of **TS-SIa** is estimated using a constrained calculation. To rationalize the regiochemistry of this initial bond forming process, all other possible regioisomeric transition states (**TS-SIb, TS-SIc,** and **TS-SId**) were computed and were all found to have significantly higher free energy barriers. From **TS-Ia**, formation of the subsequent intermediate **IM-SII•+** was determined to be exergonic by 21.3 kcal/mol. After the formation of the thermodynamically stable **5a•+** (−19.3 kcal/mol), reduction can then occur from either the reduced photocatalyst or a neutral BCB molecule to turn over the radical chain and generate BCH product **5a**.

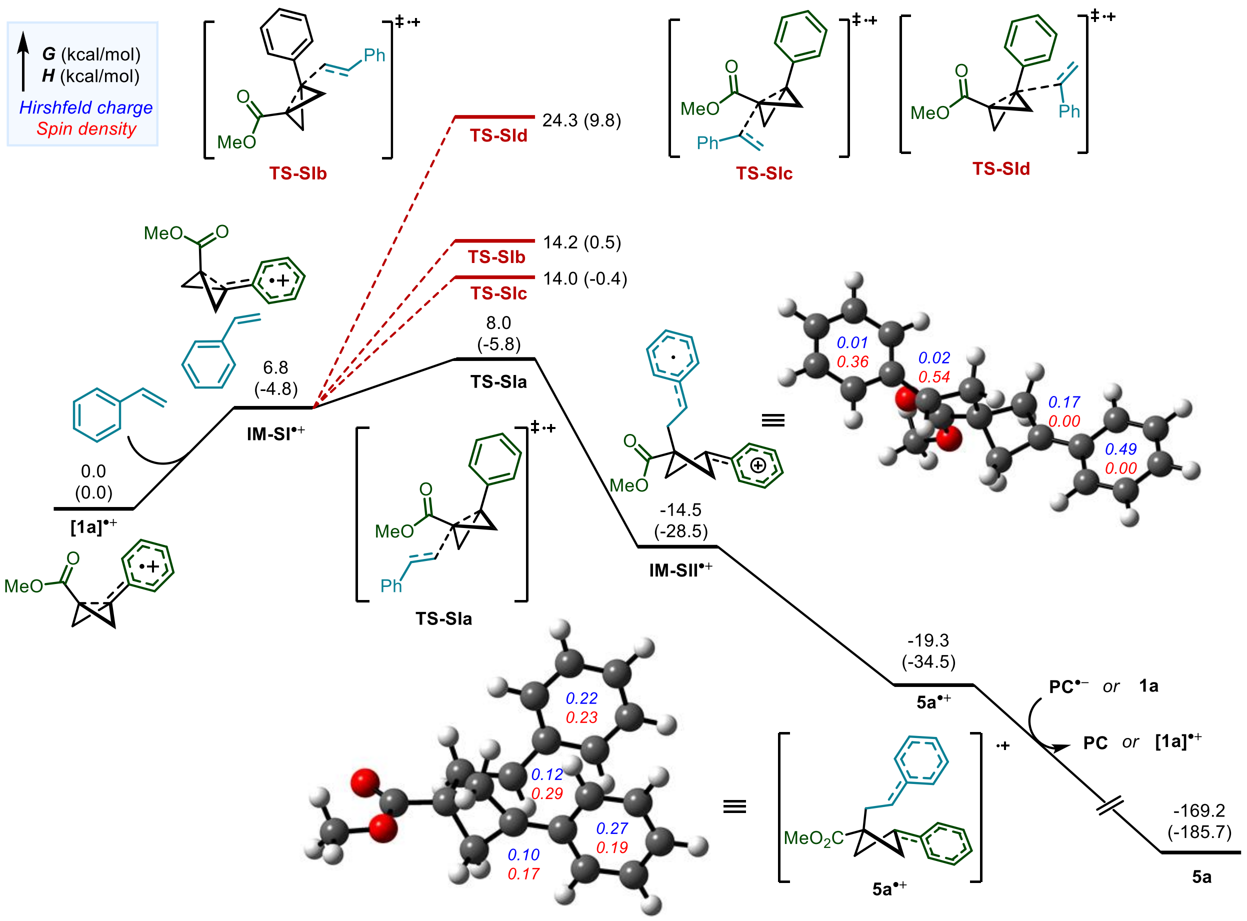

Figure S14: Computed reaction coordinate profile of BCB radical cation (1a•+) with styrene

4.4.1. Interaction between styrene radical cations and ground state BCB

As styrene was discovered to also undergo oxidation under the reaction conditions, we attempted to calculate the possibility of styrene radical cations participating in a direct reaction with ground state **1a** to access the observed products. However, it was found that upon complexation of the alkene with **1a** (**IM-SI•+**), the computed spin densities and Hirshfeld charges of the radical cation are located almost entirely on the BCB moiety, rather than the styrene, indicating that the alkene radical cation effectively oxidizes the BCB fragment. In addition, we observed an elongation of the BCB strained σ-bond in **IM-SI•+**. Therefore, we conclude that styrene radical cations are unable to directly react with ground state BCB and so this potential pathway can be discounted.

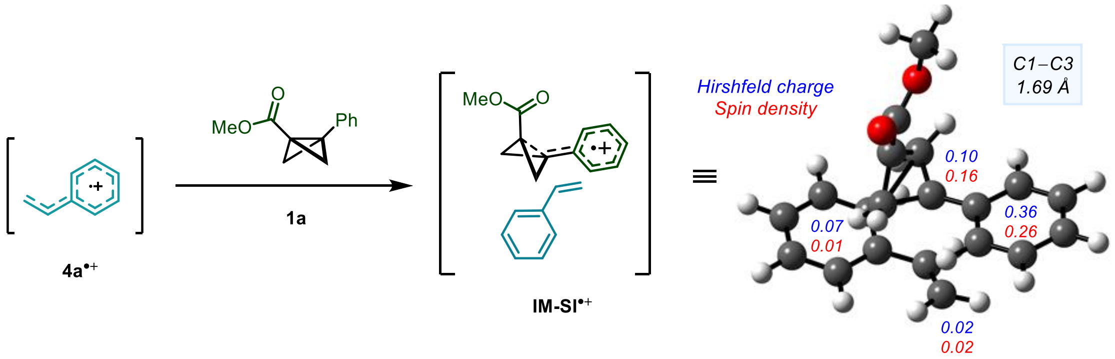

Figure S15: Interaction between styrene radical cation 4a•+ and ground state BCB

4.5. Scan of the TS-II reaction coordinate

The scan of the TS-II reaction coordinate (see Fig. **7**) revealed that the C−C bond formation transition state is a barrierless process (Fig. **S16**).

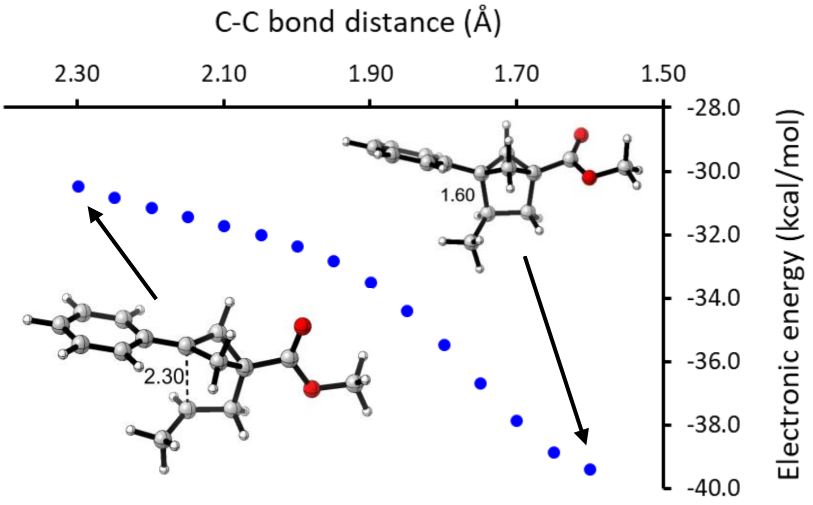

Figure S16: Scan of the TS-II reaction coordinate

4.6. Computed Cartesian Coordinates

4.6.1. Manuscript structures

1a Eopt -614.928304

C	-0.106881	-1.449544	0.498472
C	-0.882267	-2.265654	-0.485265
C	-1.552473	-1.102683	0.175398
H	-1.206168	-3.275298	-0.203682
H	-0.630467	-2.142065	-1.542795
C	-1.127885	-1.367725	1.587098
H	-1.473940	-2.296579	2.057742
H	-1.082249	-0.505706	2.257694
C	-1.916155	0.164450	-0.467167
O	-1.974482	0.332294	-1.664029
C	-2.404850	2.427846	-0.087004
H	-1.559527	2.796510	-0.684971

H	-2.559823	3.075194	0.782302
H	-3.307620	2.414870	-0.713343
C	1.087680	-0.616009	0.268534
C	1.854721	-0.779901	-0.893967
C	1.466336	0.371624	1.190727
C	2.958612	0.035660	-1.137560
H	1.592128	-1.559193	-1.612065
C	2.568998	1.186282	0.944150
H	0.897857	0.504635	2.113044
C	3.318352	1.024329	-0.222054
H	3.543938	-0.107077	-2.048567
H	2.847414	1.951365	1.672187
H	4.184753	1.661352	-0.412016
O	-2.129446	1.131450	0.427316

**1a•+**		Eopt	-614.703543
C	-0.125168	1.363167	0.537239
C	0.712319	2.190395	-0.403922
C	1.512118	1.138316	0.262209
H	0.896467	3.213602	-0.045638
H	0.556035	2.111674	-1.483068
C	0.916426	1.220696	1.616712
H	1.120295	2.157412	2.155918
H	0.929878	0.327914	2.246708
C	2.085002	-0.056609	-0.424895
O	2.227692	-0.105177	-1.619100
C	2.951767	-2.206172	-0.087682
H	2.236226	-2.681782	-0.771524
H	3.144787	-2.850906	0.774209
H	3.885307	-1.990510	-0.623798

C	-1.234512	0.523310	0.246437
C	-1.916376	0.640882	-0.995259
C	-1.694285	-0.416750	1.208578
C	-3.002033	-0.169204	-1.266627
H	-1.583432	1.373021	-1.731653
C	-2.781302	-1.220498	0.923989
H	-1.190523	-0.501040	2.172054
C	-3.433132	-1.100848	-0.311631
H	-3.523183	-0.083524	-2.220721
H	-3.132761	-1.945508	1.658871
H	-4.290836	-1.739398	-0.531895
O	2.398158	-0.998707	0.440305

**3a**		Eopt	-732.923824
C	0.589978	-0.026708	-1.005358
C	1.601242	0.327599	0.116416
C	0.601184	-0.358786	1.082152
C	-0.444492	0.255434	0.114489
C	1.316998	1.815037	0.350256
C	-0.238983	1.774588	0.383611
H	0.601755	-0.024839	2.131296
H	0.639833	-1.454677	1.021855
H	0.592090	0.604262	-1.906706
H	0.625416	-1.089021	-1.282339
H	1.703018	2.435920	-0.472190
H	-0.606199	1.998596	1.398764
C	3.036307	-0.097908	0.033206
O	3.985217	0.647191	0.102528
O	3.155855	-1.415469	-0.133676
C	4.474719	-1.936171	-0.232767

H	5.048644	-1.718638	0.678955
H	5.000055	-1.503693	-1.095888
H	4.368988	-3.018431	-0.361005
C	-1.856082	-0.235400	0.050427
C	-2.487442	-0.462583	-1.177531
C	-2.585936	-0.439741	1.228953
C	-3.817202	-0.883145	-1.228877
H	-1.930625	-0.309287	-2.105493
C	-3.913675	-0.861329	1.181079
H	-2.105372	-0.268667	2.196132
C	-4.534404	-1.084108	-0.049661
H	-4.294394	-1.056788	-2.196185
H	-4.467453	-1.019118	2.109533
H	-5.574333	-1.416019	-0.088389
H	1.759810	2.178476	1.288420
C	-0.933591	2.705405	-0.598362
H	-2.027455	2.595962	-0.541709
H	-0.687048	3.755026	-0.377200
H	-0.627040	2.504064	-1.636861

3a$^{•+}$ Eopt -732.679545

C	0.596115	-0.012314	-1.032513
C	1.584690	0.332254	0.113330
C	0.600528	-0.388006	1.070863
C	-0.445785	0.213806	0.086590
C	1.274433	1.807526	0.375340
C	-0.280390	1.760615	0.407745
H	0.579526	-0.059117	2.119159
H	0.656559	-1.481617	0.998615
H	0.589551	0.647230	-1.910897

H	0.655580	-1.065132	-1.337833
H	1.645749	2.450394	-0.435439
H	-0.656137	1.938418	1.426558
C	3.027859	-0.079019	0.030828
O	3.960965	0.682551	0.101041
O	3.154603	-1.391749	-0.136920
C	4.477768	-1.906700	-0.239128
H	5.049950	-1.685512	0.672386
H	4.996446	-1.470157	-1.103774
H	4.375666	-2.988956	-0.367317
C	-1.823041	-0.259028	0.029469
C	-2.482789	-0.452804	-1.227514
C	-2.543932	-0.490148	1.253090
C	-3.791975	-0.850406	-1.258124
H	-1.927538	-0.277899	-2.149906
C	-3.852264	-0.890090	1.215768
H	-2.030877	-0.340243	2.204273
C	-4.488023	-1.073576	-0.038046
H	-4.309725	-0.999425	-2.205926
H	-4.410275	-1.068556	2.135027
H	-5.532124	-1.390905	-0.069281
H	1.701861	2.159228	1.323776
C	-0.987976	2.696606	-0.556580
H	-2.081547	2.594542	-0.490832
H	-0.734425	3.737914	-0.308843
H	-0.684104	2.521079	-1.599425

IM-2z$^{\bullet+}$ Eopt -1119.752467

C	-1.150050	0.767856	1.062491
H	-0.669290	0.427495	1.991191

C	-1.886516	-0.375355	0.357516
C	-4.002458	-0.291531	0.140149
C	-2.873524	0.093856	-0.771143
H	-2.805309	-0.497141	-1.700697
H	-2.801948	1.159222	-1.050507
C	-3.098930	-0.905180	1.169605
H	-3.179138	-0.522703	2.200667
H	-3.130102	-2.006418	1.206751
C	-0.929906	-1.452949	-0.116001
O	-1.124655	-2.637881	-0.047134
C	1.111558	-1.821340	-1.237960
H	0.630661	-2.480817	-1.971983
H	1.571494	-2.427279	-0.445879
H	1.869291	-1.203837	-1.730535
C	-5.398449	-0.112453	0.064738
C	-6.251326	-0.569351	1.106916
C	-5.997890	0.531300	-1.052604
C	-7.624692	-0.388337	1.029667
H	-5.814254	-1.068354	1.975304
C	-7.372933	0.705304	-1.116711
H	-5.364090	0.890194	-1.867289
C	-8.196328	0.248817	-0.079324
H	-8.263721	-0.746162	1.840268
H	-7.815264	1.201893	-1.983435
H	-9.277854	0.388479	-0.135035
O	0.156629	-0.919473	-0.680313
H	-1.904840	1.524296	1.362730
C	-0.206571	1.523971	0.223590
H	-0.501896	1.720260	-0.814102
C	0.988693	2.037320	0.673328

C	2.154294	1.709144	-0.453795
C	1.799088	3.072795	-0.142716
H	1.297467	1.853088	1.703564
H	1.743400	1.289682	-1.376393
H	2.465475	3.645255	0.506368
H	1.226780	3.650726	-0.869869
C	3.341626	0.986358	0.114835
H	3.056974	-0.051515	0.346298
H	3.661998	1.456928	1.056506
C	4.506701	0.987365	-0.890212
H	4.800636	2.026281	-1.103652
H	4.170031	0.547029	-1.841844
C	5.687386	0.210938	-0.359366
C	5.748456	-1.180002	-0.511749
C	6.717598	0.855966	0.334410
C	6.814998	-1.908746	0.013176
H	4.950752	-1.698239	-1.051904
C	7.786727	0.130066	0.860363
H	6.683739	1.941618	0.461206
C	7.838127	-1.254882	0.701418
H	6.849083	-2.992618	-0.118401
H	8.584662	0.649999	1.395372
H	8.675407	-1.824116	1.111151

IM-I$^{•+}$ Eopt -732.623010

C	3.653116	-1.367509	-0.676317
C	3.447573	-0.092383	-0.336895
H	3.284737	0.680577	-1.092670
H	3.654555	-1.633530	-1.740829
C	0.406409	-0.369397	-0.043033

C	-0.765798	-1.580088	0.106718
C	0.329000	-1.365260	1.081642
H	0.066351	-1.031263	2.089054
H	1.128850	-2.119634	1.051944
C	-0.005640	-1.314351	-1.138706
H	0.765962	-2.061702	-1.376112
H	-0.545915	-0.939504	-2.011648
H	3.439434	0.226191	0.711294
C	0.280129	1.047524	0.008516
C	0.438408	1.734237	1.240146
C	0.046247	1.788228	-1.178968
C	0.346615	3.113254	1.278824
H	0.639819	1.174890	2.154388
C	-0.044720	3.166834	-1.126705
H	-0.055759	1.270698	-2.133295
C	0.102801	3.829173	0.099203
H	0.465869	3.642534	2.224847
H	-0.228201	3.737441	-2.037789
H	0.031076	4.917956	0.135129
C	-2.209506	-1.326387	0.380667
O	-2.648534	-1.248073	1.499055
O	-2.894542	-1.211227	-0.738708
C	-4.295795	-0.957001	-0.618291
H	-4.463547	-0.010629	-0.087185
H	-4.782781	-1.776142	-0.072866
H	-4.679570	-0.895825	-1.640449
C	3.893628	-2.491651	0.283504
H	3.839159	-2.154920	1.329323
H	3.163275	-3.304254	0.135427
H	4.886495	-2.939824	0.116827

IM-II•+ Eopt -732.649633

C	-2.211977	1.032651	-1.385943
C	-3.358443	1.493277	-0.551176
H	-4.300414	0.940525	-0.618268
H	-2.572692	0.482635	-2.269341
C	-1.206618	0.108298	-0.654577
C	0.894138	0.215577	-0.360068
C	-0.261121	0.780644	0.383934
H	-0.348280	0.494366	1.442631
H	-0.259757	1.883778	0.333470
C	0.051045	-0.278397	-1.479220
H	0.189595	0.336817	-2.386087
H	0.180883	-1.333252	-1.765733
C	-1.945059	-1.112967	-0.136939
O	-2.142556	-2.103624	-0.795034
C	-3.190692	-1.978641	1.655193
H	-2.622838	-2.917798	1.695910
H	-4.097085	-2.127941	1.052788
H	-3.456537	-1.651756	2.665084
C	2.256088	0.123917	-0.079555
C	3.131066	-0.515834	-1.002271
C	2.775648	0.681479	1.122848
C	4.482086	-0.587248	-0.727407
H	2.728180	-0.944017	-1.921776
C	4.128746	0.602274	1.385001
H	2.101132	1.168083	1.829408
C	4.973697	-0.030171	0.461738
H	5.163704	-1.073096	-1.425840
H	4.540289	1.025383	2.301609

H	6.043010	-0.091669	0.675214
O	-2.392072	-0.937459	1.099026
H	-1.630418	1.897474	-1.755390
C	-3.182838	2.472246	0.555135
H	-2.438649	3.246098	0.302627
H	-4.128995	2.972751	0.809610
H	-2.828707	1.977652	1.481998

TS-II Eopt -732.667377

C	0.538288	-0.932689	-0.879739
C	1.530545	-0.066234	-0.053576
C	0.525785	-0.198133	1.127138
C	-0.509761	-0.349725	0.039381
C	1.366890	1.371371	-0.565801
C	-0.120126	1.580384	-0.718047
H	0.455373	0.618191	1.858222
H	0.652588	-1.157894	1.651684
H	0.477210	-0.780359	-1.965420
H	0.668665	-2.003071	-0.657127
H	1.864423	1.497410	-1.538442
C	-1.928123	-0.405880	0.054316
C	-2.640729	-0.819094	-1.102483
C	-2.645468	-0.017914	1.216137
C	-4.022784	-0.855353	-1.086090
H	-2.090364	-1.110528	-1.999001
C	-4.027439	-0.059944	1.220646
H	-2.098895	0.305182	2.103951
C	-4.716552	-0.474827	0.071604
H	-4.573392	-1.177480	-1.970863
H	-4.581924	0.229507	2.114238

H	-5.807996	-0.500305	0.078234
C	2.925671	-0.602009	0.153657
O	3.144034	-1.724825	0.536618
O	3.863694	0.289712	-0.124811
C	5.218991	-0.118797	0.045785
H	5.446310	-0.973688	-0.605279
H	5.405685	-0.399676	1.091130
H	5.833458	0.743267	-0.231120
H	1.793234	2.088138	0.149547
H	-0.540987	1.381436	-1.709189
C	-0.827800	2.611648	0.072752
H	-0.522847	3.588041	-0.349298
H	-1.918722	2.538794	-0.020052
H	-0.527971	2.617109	1.130250

TS-Ia Eopt -732.624440

C	3.266160	-1.263987	-0.555687
C	3.754646	-0.139895	0.023574
H	3.939669	0.732168	-0.614415
H	3.149549	-1.329787	-1.639808
C	0.990185	-0.749916	-0.182791
C	-0.562808	-1.336942	0.345214
C	0.593428	-1.021410	1.240615
H	0.520243	-0.179606	1.933996
H	1.088417	-1.902017	1.673754
C	0.232760	-1.898914	-0.788448
H	0.683905	-2.880916	-0.591166
H	-0.137059	-1.785310	-1.811279
C	0.949713	0.605192	-0.817098
O	0.777475	0.770587	-1.997056

C	1.127947	2.905620	-0.396704
H	0.166116	3.137442	-0.872665
H	1.941199	3.053929	-1.119692
H	1.280675	3.535997	0.483885
C	-1.841241	-0.693961	0.294953
C	-2.684038	-0.865100	-0.828777
C	-2.285812	0.099869	1.378859
C	-3.923720	-0.247144	-0.868636
H	-2.362677	-1.489184	-1.663690
C	-3.527115	0.713662	1.328162
H	-1.653909	0.226086	2.258982
C	-4.344779	0.543948	0.205676
H	-4.570569	-0.379917	-1.736976
H	-3.865871	1.326514	2.164645
H	-5.322174	1.029123	0.169886
O	1.131054	1.558798	0.078637
H	3.149639	-2.186716	0.019624
C	4.005423	0.037235	1.476359
H	3.838154	-0.886162	2.046946
H	5.036850	0.384171	1.646342
H	3.345116	0.829575	1.867346

TS-Ib Eopt -732.613649

C	2.586843	-1.081067	-1.277483
C	3.274204	-0.718845	-0.158319
H	3.456777	0.348807	0.010645
H	2.410332	-0.357256	-2.075565
C	0.624777	-0.727136	-0.384834
C	-0.929696	-1.652755	-0.080931
C	0.325729	-1.738849	0.711554

H	0.297467	-1.420470	1.757702
H	0.866301	-2.686888	0.578830
C	-0.294000	-1.408781	-1.403255
H	0.143763	-2.308859	-1.855819
H	-0.829215	-0.795140	-2.133784
H	2.458837	-2.138467	-1.524044
C	0.543818	0.728580	-0.156347
C	0.300585	1.592165	-1.236754
C	0.722836	1.268492	1.127884
C	0.223607	2.965587	-1.033854
H	0.179367	1.192286	-2.245253
C	0.640321	2.642030	1.326453
H	0.927581	0.619796	1.980586
C	0.390036	3.491876	0.247754
H	0.033439	3.628089	-1.879750
H	0.773718	3.052161	2.328771
H	0.327909	4.570163	0.406769
C	-2.186799	-1.069017	0.430764
O	-2.415660	-0.937539	1.609584
O	-2.998915	-0.720061	-0.553894
C	-4.244147	-0.130803	-0.183928
H	-4.073804	0.799359	0.374997
H	-4.826358	-0.826494	0.435265
H	-4.770378	0.078411	-1.119852
C	3.739085	-1.645319	0.896099
H	4.823177	-1.509554	1.043779
H	3.276356	-1.384122	1.862973
H	3.538141	-2.697971	0.659411

TS-Ic Eopt -732.617217

C	-3.362582	-0.431210	0.292226
C	-3.615505	0.811540	-0.247573
H	-3.503591	1.718937	0.349577
H	-3.238670	-0.484294	1.379637
C	-1.217499	-0.418879	0.071147
C	0.264082	-1.318876	-0.281433
C	-0.761785	-0.930897	-1.284306
H	-0.506814	-0.205881	-2.061169
H	-1.385857	-1.756809	-1.651120
C	-0.663091	-1.608879	0.842838
H	-1.270209	-2.516471	0.728369
H	-0.326623	-1.442456	1.870039
C	-0.836000	0.949466	0.555612
O	-0.715160	1.220841	1.722888
C	-0.298545	3.129916	-0.121802
H	0.650123	3.137270	0.430981
H	-1.081808	3.602460	0.485955
H	-0.188613	3.654016	-1.075527
C	1.621093	-0.852966	-0.193593
C	2.355657	-1.018038	1.001820
C	2.238369	-0.235227	-1.304199
C	3.663674	-0.564277	1.084002
H	1.899261	-1.510387	1.861537
C	3.545996	0.217128	-1.211690
H	1.690541	-0.115334	-2.239570
C	4.257708	0.056358	-0.018946
H	4.227206	-0.693786	2.009129
H	4.018000	0.695946	-2.070842
H	5.287143	0.413538	0.049819
O	-0.667240	1.789957	-0.449535

H	-3.890883	0.924936	-1.299656
C	-3.784279	-1.691498	-0.396548
H	-3.807388	-1.576324	-1.488853
H	-3.153964	-2.551410	-0.130953
H	-4.802414	-1.942290	-0.059499

TS-Id Eopt -732.601219

C	2.900961	0.017635	-0.489205
C	3.066513	1.110487	0.368059
H	2.658815	2.091052	0.117389
H	2.638101	0.264856	-1.523920
C	0.989710	-0.462227	-0.069564
C	-0.059956	-1.831397	0.145625
C	0.960956	-1.463174	1.120396
H	0.618722	-1.131498	2.103814
H	1.860978	-2.086904	1.153371
C	0.614925	-1.559838	-1.118031
H	1.466300	-2.200547	-1.371933
H	-0.004064	-1.289555	-1.976712
H	3.546880	0.988596	1.342952
C	0.202085	0.810608	-0.034536
C	-0.082817	1.444943	1.181268
C	-0.229503	1.396400	-1.230211
C	-0.802526	2.636407	1.198714
H	0.263534	1.018344	2.124219
C	-0.947648	2.590257	-1.209323
H	-0.001034	0.927875	-2.189493
C	-1.238831	3.210858	0.004417
H	-1.021095	3.119712	2.152656
H	-1.279367	3.036320	-2.148640

H	-1.802354	4.145730	0.020501
C	-1.514923	-1.604583	0.410502
O	-1.961558	-1.520061	1.525816
O	-2.199502	-1.503620	-0.710734
C	-3.594880	-1.221030	-0.595220
H	-3.741486	-0.258403	-0.087034
H	-4.097808	-2.016382	-0.029668
H	-3.979238	-1.176050	-1.618052
C	3.758065	-1.198238	-0.307809
H	3.969400	-1.402289	0.750778
H	3.340464	-2.096968	-0.780880
H	4.718944	-1.007077	-0.810489

propene Eopt -117.913402

C	0.131615	-0.452816	0.000027
C	1.283658	0.220153	0.000003
H	2.247093	-0.296954	0.000065
H	0.163969	-1.549817	0.000110
H	1.305638	1.315443	-0.000072
C	-1.234052	0.163511	-0.000027
H	-1.184712	1.262414	-0.000209
H	-1.809684	-0.158230	-0.883010
H	-1.809626	-0.157946	0.883098

4.6.2. Supporting information structures

1a•+ open Eopt -614.701263

C	0.495515	0.085310	0.000009
C	-0.461922	1.241401	0.000062
C	-1.563231	0.221939	0.000045
H	-0.385034	1.893776	-0.889293
H	-0.385006	1.893723	0.889454
C	-0.605034	-0.935487	-0.000023
H	-0.614860	-1.592079	-0.889420
H	-0.614836	-1.592168	0.889310
C	-3.001439	0.359596	0.000089
O	-3.574971	1.430245	0.000098
C	-5.037733	-0.808484	-0.000049
H	-5.420051	-0.297420	0.894765
H	-5.356079	-1.855814	-0.000135
H	-5.420005	-0.297287	-0.894805
C	1.879277	-0.003588	-0.000010
C	2.670999	1.183292	0.000021
C	2.512870	-1.281664	-0.000064
C	4.046671	1.086294	-0.000001
H	2.183426	2.159631	0.000062
C	3.889694	-1.361628	-0.000085
H	1.903853	-2.187175	-0.000088
C	4.648209	-0.181279	-0.000054
H	4.665002	1.983981	0.000022
H	4.387947	-2.331182	-0.000126
H	5.738157	-0.251054	-0.000071
O	-3.615750	-0.825389	-0.000013

4a Eopt -309.651919

C	2.262705	0.264414	0.000295
C	1.358838	1.329912	-0.000178
C	-0.012121	1.089701	-0.000525
C	-0.512857	-0.223448	-0.000376
C	0.407788	-1.282676	0.000015
C	1.781736	-1.044029	0.000376
H	3.337932	0.456288	0.000552
H	1.727101	2.358349	-0.000322
H	-0.699838	1.938136	-0.001011
H	0.037749	-2.311549	0.000064
H	2.478786	-1.885021	0.000703
C	-1.957868	-0.533284	-0.000615
H	-2.195202	-1.603272	-0.002041
C	-2.971596	0.338822	0.000852
H	-4.006321	-0.011269	0.000505
H	-2.819959	1.421856	0.002484

4a$^{\bullet+}$ Eopt -309.419448

C	2.225430	0.286344	0.000081
C	1.314738	1.361771	0.000083
C	-0.036482	1.113644	-0.000001
C	-0.520464	-0.237893	-0.000087
C	0.429704	-1.311857	-0.000148
C	1.778831	-1.049743	-0.000043
H	3.297448	0.493260	0.000167
H	1.686190	2.386693	0.000147
H	-0.736615	1.948682	-0.000025
H	0.062615	-2.339678	-0.000254
H	2.502420	-1.865223	-0.000059

C	-1.901143	-0.566144	-0.000087
H	-2.153562	-1.630196	-0.000243
C	-2.940463	0.338203	0.000155
H	-3.972084	-0.018417	0.000143
H	-2.787321	1.418928	0.000409

5a Eopt -924.658040

C	1.094720	0.237687	0.907529
C	2.010215	-0.275899	-0.232852
C	1.472316	0.921485	-1.053074
C	0.159500	0.612663	-0.275045
C	1.204831	-1.450282	-0.791914
C	-0.200975	-0.770733	-0.880625
H	1.458385	0.826320	-2.149736
H	1.917757	1.877629	-0.749233
H	0.751838	-0.497845	1.649748
H	1.519192	1.118488	1.406764
H	1.208861	-2.309360	-0.106001
H	-0.466125	-0.633868	-1.940901
C	3.478180	-0.490949	-0.015718
O	4.066040	-1.523587	-0.234763
O	4.074995	0.602712	0.457679
C	5.472003	0.518881	0.707513
H	6.019031	0.287235	-0.217113
H	5.687429	-0.258701	1.453664
H	5.775388	1.499671	1.088058
C	-0.918048	1.652391	-0.173485
C	-1.118701	2.385175	1.001572
C	-1.736433	1.926207	-1.278255
C	-2.113042	3.361669	1.075778

H	-0.495522	2.186720	1.876638
C	-2.727039	2.903686	-1.209084
H	-1.598018	1.367735	-2.207418
C	-2.920538	3.625124	-0.029593
H	-2.256335	3.918600	2.004541
H	-3.354241	3.102371	-2.081184
H	-3.699165	4.388994	0.026725
C	-1.318653	-1.567922	-0.243376
C	-1.923175	-1.230252	0.972705
C	-1.765736	-2.722586	-0.903552
C	-2.938267	-2.023141	1.513354
H	-1.612350	-0.334139	1.510789
C	-2.775140	-3.517768	-0.366767
H	-1.311047	-3.000655	-1.858815
C	-3.367563	-3.169970	0.848963
H	-3.396182	-1.736515	2.462960
H	-3.104467	-4.411774	-0.901458
H	-4.161364	-3.788970	1.272904
H	1.577923	-1.787735	-1.768697

5a•+ Eopt -924.414870

C	-1.190474	-0.143098	0.974233
C	-2.080404	0.110370	-0.280987
C	-1.569204	-1.267365	-0.806353
C	-0.390468	-1.042762	0.090043
C	-1.443935	1.240299	-1.109571
C	0.000597	0.916538	-1.309664
H	-1.432803	-1.421058	-1.884723
H	-2.187431	-2.082482	-0.396464
H	-0.708510	0.704996	1.479443

H	-1.736255	-0.746567	1.717405
H	-1.596155	2.199427	-0.598623
H	0.242186	0.255163	-2.146336
C	-3.567749	0.297275	-0.075962
O	-4.198587	1.235685	-0.492977
O	-4.089311	-0.700567	0.625285
C	-5.487501	-0.646254	0.896898
H	-6.059961	-0.642801	-0.040506
H	-5.731080	0.255973	1.474045
H	-5.719431	-1.543118	1.479354
C	0.912598	-1.584722	0.179698
C	1.777636	-1.168319	1.219820
C	1.383017	-2.499181	-0.794252
C	3.069676	-1.665226	1.288732
H	1.424319	-0.451171	1.963267
C	2.676774	-2.986967	-0.716813
H	0.722207	-2.815651	-1.603665
C	3.519027	-2.570921	0.322473
H	3.736575	-1.343644	2.089895
H	3.040660	-3.693097	-1.464569
H	4.539320	-2.955907	0.376360
C	1.107637	1.553832	-0.689981
C	0.966476	2.536155	0.324404
C	2.415896	1.129415	-1.043423
C	2.084990	3.056706	0.955570
H	-0.023459	2.897132	0.607197
C	3.527901	1.658755	-0.409471
H	2.536629	0.367381	-1.817148
C	3.365116	2.620209	0.593259
H	1.966615	3.812363	1.733769

H	4.527372	1.320382	-0.687139
H	4.241230	3.035204	1.095465
H	-1.944471	1.298094	-2.087410

IM-SI•+ Eopt -924.365046

C	-2.083487	-2.073585	-0.593831
C	-1.379671	-2.777549	0.301077
H	-0.687588	-3.557262	-0.024567
H	-1.937509	-2.291651	-1.657580
C	0.468233	-0.134941	0.033528
C	0.421051	1.546208	0.230973
C	-0.118805	0.582041	1.216923
H	0.364624	0.511590	2.194944
H	-1.219630	0.531051	1.250516
C	0.003928	0.844114	-1.007816
H	-1.086292	0.821722	-1.168952
H	0.588646	0.992811	-1.919035
C	-3.029602	-0.978296	-0.306058
C	-3.490945	-0.181930	-1.367017
C	-3.469342	-0.673068	0.994447
C	-4.341198	0.899084	-1.137556
H	-3.171056	-0.412338	-2.386842
C	-4.319934	0.405074	1.223210
H	-3.149421	-1.287561	1.838837
C	-4.755187	1.198823	0.159415
H	-4.681758	1.508617	-1.977130
H	-4.649253	0.626260	2.240631
H	-5.420973	2.044634	0.342631
H	-1.453157	-2.605397	1.378664
C	1.578707	-1.028670	-0.011162

C	2.056411	-1.631670	1.178893
C	2.174554	-1.364255	-1.252165
C	3.110322	-2.526017	1.125511
H	1.587728	-1.397625	2.135458
C	3.227932	-2.260644	-1.293028
H	1.798387	-0.923153	-2.175994
C	3.697488	-2.838817	-0.107250
H	3.480964	-2.988777	2.040910
H	3.689165	-2.518117	-2.247205
H	4.528598	-3.545798	-0.144489
C	1.617621	2.407611	0.449748
O	2.062103	2.626945	1.547188
O	2.086057	2.871931	-0.690820
C	3.241231	3.710618	-0.623760
H	4.081350	3.161540	-0.178185
H	3.025350	4.603476	-0.022248
H	3.470688	3.990179	-1.655793

IM-SII$^{\bullet+}$ Eopt -924.403780

C	-1.008967	-0.707119	-1.507187
C	-2.282110	-0.097137	-1.022094
H	-3.149824	-0.757923	-0.936614
H	-1.213717	-1.406714	-2.333642
C	-0.252829	-1.504551	-0.414985
C	1.661704	-0.750917	0.049933
C	0.339915	-0.613595	0.712484
H	0.350715	-1.120214	1.693347
H	-0.054219	0.401844	0.865519
C	1.202923	-1.851451	-0.838504
H	1.479964	-1.788879	-1.900870

H	1.542296	-2.829410	-0.454604
C	-1.085569	-2.694929	0.012354
O	-1.097838	-3.744025	-0.580996
C	-2.704291	-3.451111	1.535301
H	-2.139610	-4.352763	1.807838
H	-3.432134	-3.701061	0.751531
H	-3.216250	-3.044048	2.412523
C	2.848231	-0.026045	0.135517
C	3.955975	-0.368173	-0.691470
C	2.959895	1.049078	1.062812
C	5.135226	0.341115	-0.583703
H	3.866873	-1.190428	-1.403478
C	4.145141	1.749950	1.159111
H	2.107845	1.311967	1.691941
C	5.224574	1.394421	0.337736
H	5.991401	0.088782	-1.209589
H	4.243971	2.574504	1.865351
H	6.159183	1.953966	0.416232
O	-1.820078	-2.428215	1.082395
H	-0.324051	0.055548	-1.908712
C	-2.449822	1.245026	-0.596031
C	-1.415451	2.223186	-0.650561
C	-3.710040	1.661752	-0.077162
C	-1.631893	3.519570	-0.204120
H	-0.434457	1.963757	-1.053493
C	-3.914760	2.959183	0.364563
H	-4.523889	0.933944	-0.026267
C	-2.877763	3.899712	0.307738
H	-0.819830	4.248245	-0.256793
H	-4.891037	3.248001	0.760393

H	-3.040424	4.921002	0.657796

TS-SIa Eopt -924.366391

C	-1.945009	-2.172288	-1.221989
C	-2.548907	-1.611176	-0.146474
C	-3.461024	0.459228	-1.246485
C	-3.144739	-0.278631	-0.090258
H	-2.531683	-2.163661	0.798677
C	-3.961588	1.752458	-1.142472
C	-3.363084	0.309217	1.168051
C	-3.857863	1.605642	1.269491
C	-4.155238	2.330807	0.114309
H	-4.013506	2.053314	2.252918
H	-4.546139	3.347386	0.192709
H	-4.206438	2.313285	-2.046472
H	-3.126059	-0.256683	2.071967
H	-1.526363	-3.178575	-1.159727
C	0.163401	-0.940383	-0.747703
C	1.363363	0.140555	-1.335844
C	-0.049010	0.517049	-1.007610
H	-0.251267	1.175864	-0.159340
H	-0.680589	0.726503	-1.883572
C	1.066025	-1.207304	-1.914412
H	0.561655	-1.188513	-2.889984
H	1.805071	-2.007311	-1.816066
C	0.423736	-1.538341	0.596373
O	0.988800	-2.591050	0.746192
C	0.125954	-1.226820	2.902060
H	1.193152	-1.347418	3.130402
H	-0.390609	-2.185964	3.041392

H	-0.317710	-0.458491	3.541743
C	2.566449	0.585301	-0.698375
C	3.780808	-0.113143	-0.898404
C	2.558466	1.739630	0.120391
C	4.942988	0.322313	-0.282396
H	3.804714	-0.992354	-1.543394
C	3.726189	2.165443	0.732610
H	1.634112	2.299559	0.267403
C	4.916777	1.457343	0.535334
H	5.877154	-0.219324	-0.437199
H	3.716301	3.053734	1.365736
H	5.834594	1.796542	1.019621
O	-0.043255	-0.766739	1.561156
H	-1.956863	-1.701375	-2.207557
H	-3.330053	0.014396	-2.235225

TS-SIb Eopt -924.356334

C	-1.064035	-1.238878	2.271883
C	-1.902998	-0.244604	1.834269
C	-2.986716	-1.478581	-0.057782
C	-2.771388	-0.296207	0.684993
H	-1.822446	0.730186	2.325246
C	-3.752880	-1.449779	-1.213166
C	-3.367249	0.904413	0.241040
C	-4.135761	0.927481	-0.915298
C	-4.325017	-0.247607	-1.645940
H	-4.585264	1.862356	-1.253939
H	-4.926135	-0.230791	-2.557273
H	-3.912292	-2.366669	-1.782959
H	-3.202057	1.823422	0.807842

H	-0.479311	-1.075749	3.178418
C	0.616223	-0.851528	0.800474
C	1.887268	-1.739190	0.051928
C	0.462005	-1.936716	-0.268960
H	0.130317	-1.663913	-1.274082
H	0.016881	-2.867696	0.103540
C	1.796363	-1.531446	1.507867
H	1.505109	-2.408849	2.097713
H	2.560523	-0.912799	1.984741
H	-1.167178	-2.271460	1.936183
H	-2.558553	-2.425270	0.275557
C	0.598056	0.588949	0.483054
C	1.232663	1.507913	1.334030
C	-0.080132	1.059650	-0.652443
C	1.200144	2.866925	1.045005
H	1.742911	1.160969	2.234284
C	-0.109102	2.422232	-0.937110
H	-0.596188	0.364338	-1.316125
C	0.530786	3.326982	-0.091854
H	1.696816	3.573613	1.712147
H	-0.639553	2.776499	-1.822620
H	0.506325	4.395251	-0.315692
C	2.824065	-1.047451	-0.875246
O	2.626795	-0.971650	-2.062241
O	3.855852	-0.535955	-0.229457
C	4.803700	0.198669	-1.003778
H	4.316045	1.060367	-1.479336
H	5.244858	-0.445049	-1.776233
H	5.572051	0.534719	-0.301614

TS-SIc Eopt -924.357339

C	2.067689	1.371830	-0.444032
C	1.921303	2.495838	0.364323
H	1.362438	3.362749	0.007699
H	1.835840	1.510497	-1.505624
C	0.201372	0.451534	-0.171216
C	-0.741289	-1.010151	0.182036
C	0.069241	-0.261766	1.168600
H	-0.427416	0.249498	1.996716
H	1.015260	-0.744530	1.456808
C	0.160242	-0.843859	-0.979978
H	1.112331	-1.391041	-0.919427
H	-0.262173	-0.816037	-1.988484
C	-0.750422	1.540395	-0.568374
O	-1.004956	1.801540	-1.716962
C	-2.161276	3.231916	0.239984
H	-3.041107	2.853276	-0.296865
H	-1.681334	4.022120	-0.352545
H	-2.450029	3.614485	1.223189
C	-2.170939	-1.174625	0.166922
C	-2.826922	-1.598968	-1.008822
C	-2.929844	-0.924261	1.330826
C	-4.205582	-1.753205	-1.020031
H	-2.251699	-1.815003	-1.910062
C	-4.308096	-1.079263	1.309860
H	-2.435255	-0.612551	2.251558
C	-4.946212	-1.489734	0.135754
H	-4.709020	-2.081185	-1.930609
H	-4.891431	-0.882523	2.210541
H	-6.031218	-1.610885	0.122954

O	-1.240200	2.168962	0.486302
C	3.018536	0.281992	-0.151383
C	3.491185	-0.505071	-1.214054
C	3.457746	-0.004808	1.152254
C	4.383508	-1.549035	-0.982346
H	3.160534	-0.289155	-2.232945
C	4.344929	-1.052316	1.381575
H	3.109292	0.590283	1.998923
C	4.809378	-1.826068	0.316356
H	4.745899	-2.148542	-1.819393
H	4.676706	-1.265957	2.399281
H	5.505957	-2.646417	0.500399
H	2.310377	2.534658	1.383655

TS-SId Eopt -924.340524

C	1.427100	-1.358353	0.209360
C	1.163544	-2.399288	-0.720943
H	0.357008	-3.109372	-0.533768
H	1.079796	-1.580403	1.225021
C	-0.006576	-0.111332	-0.091677
C	-0.363545	1.554211	-0.386855
C	0.280761	0.727493	-1.385678
H	-0.273216	0.528159	-2.306020
H	1.361430	0.857988	-1.523691
C	0.255704	1.127174	0.849132
H	1.333406	1.302040	0.956317
H	-0.316790	1.240043	1.772347
C	2.720634	-0.636683	0.174845
C	3.264220	-0.164999	1.381137
C	3.423737	-0.407985	-1.020301

C	4.475449	0.521573	1.393686
H	2.735251	-0.348540	2.319574
C	4.630564	0.285324	-1.005717
H	3.037489	-0.775430	-1.973385
C	5.157694	0.752108	0.199301
H	4.887219	0.877375	2.339743
H	5.164778	0.458588	-1.941536
H	6.105767	1.293449	0.206957
H	1.701166	-2.477429	-1.667680
C	-1.284450	-0.883996	0.054481
C	-1.935223	-1.410987	-1.067476
C	-1.818734	-1.114445	1.327259
C	-3.111517	-2.141327	-0.917773
H	-1.523687	-1.260972	-2.067431
C	-2.995468	-1.845897	1.474029
H	-1.316758	-0.725461	2.215641
C	-3.646118	-2.357813	0.352253
H	-3.611267	-2.544585	-1.800368
H	-3.403692	-2.015884	2.471929
H	-4.568658	-2.930033	0.467618
C	-1.794211	1.987141	-0.531877
O	-2.343629	2.037531	-1.601242
O	-2.329130	2.275032	0.635217
C	-3.712141	2.634824	0.644710
H	-4.316524	1.804803	0.254800
H	-3.874902	3.530270	0.030909
H	-3.964286	2.834447	1.689846

5. X-RAY CRYSTALLOGRAPHY

5.1. X-Ray Diffraction Data

Data sets for compounds **5r**, **5ad**, **5ad′** and **5au** were collected with a Bruker D8 Venture Photon III Diffractometer. Programs used: data collection: *APEX4* Version 2021.4-0;[31] cell refinement: *SAINT* Version 8.40B (Bruker AXS Inc., **2021**); data reduction: *SAINT* Version 8.40B (Bruker AXS Inc., **2021**); absorption correction, *SADABS* Version 2016/2 (Bruker AXS Inc., **2021**); structure solution *SHELXT*-Version 2018-3;[32] structure refinement *SHELXL*- Version 2018-3;[33] and graphics, *XP.*[34] *R*-values are given for observed reflections, and wR^2 values are given for all reflections.

Methyl-2-methyl-3,4-diphenylbicyclo[2.1.1]hexane-1-carboxylate (5r)

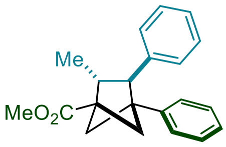

5r

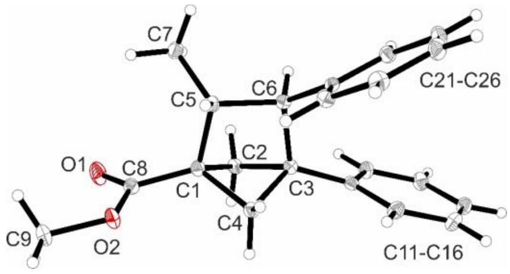

Figure S17: Crystal structure of compound 5r. Thermal ellipsoids are shown at 30% probability

A colourless, prism-like specimen of **5r** ($C_{21}H_{22}O_2$), approximate dimensions 0.047 mm x 0.085 mm x 0.106 mm, was used for the X-ray crystallographic analysis. The X-ray intensity data were measured on a single crystal diffractometer Bruker D8 Venture Photon III system equipped with a micro focus tube Mo ImS (MoKα, λ = 0.71073 Å) and a MX mirror monochromator. A total of 664 frames were collected. The total exposure time was 4.80 hours. The frames were integrated with the Bruker SAINT software package using a narrow-frame algorithm. The integration of the data using a monoclinic unit cell yielded a total of 39483 reflections to a maximum θ angle of 26.82° (0.79 Å resolution), of which 3564 were independent (average redundancy 11.078, completeness = 99.7%, R_{int}= 12.82%, R_{sig}= 6.45%) and 2601 (72.98%) were greater than 2σ(F^2). The final cell constants of a = 26.9897(15) Å, b = 5.8766(3) Å, c = 23.9259(13) Å, β = 118.878(2)°, volume = 3322.9(3) $Å^3$, are based upon the refinement of the XYZ-centroids of 5924 reflections above 20 σ(I) with 6.322° < 2θ < 53.45°. Data were corrected for absorption effects using the multi-scan method (SADABS). The ratio of minimum to maximum apparent transmission was 0.618. The calculated minimum and maximum transmission coefficients (based on crystal size) are 0.9920 and 0.9960.

The structure was solved and refined using the Bruker SHELXTL Software Package, using the space group C 1 2/c 1, with Z = 8 for the formula unit, $C_{21}H_{22}O_2$. The final anisotropic full-matrix least-squares refinement on F^2 with 210 variables converged at R1 = 4.95%, for the observed data and wR^2 = 13.20% for all data. The goodness-of-fit was 1.048. The largest peak in the final difference electron density synthesis was 0.359 e^-/ and $Å^3$ the largest hole was -0.252 e^-/ $Å^3$ with an RMS deviation of 0.052 e^-/ $Å^3$. On the basis of the final model, the

calculated density was 1.225 g/cm^3 and F(000), 1312 e$^-$.

Table S14: Sample and crystal data for 5r

**CCDC number**	2331780	
**Chemical formula**	$C_{21}H_{22}O_2$	
**Formula weight**	306.38 g/mol	
**Temperature**	100(2) K	
**Wavelength**	0.71073 Å	
**Crystal size**	0.047 x 0.085 x 0.106 mm	
**Crystal habit**	colourless prism	
**Crystal system**	monoclinic	
**Space group**	C 1 2/c 1	
**Unit cell dimensions**	a = 26.9897(15) Å	α = 90°
	b = 5.8766(3) Å	β = 118.878(2)°
	c = 23.9259(13) Å	γ = 90°
**Volume**	3322.9(3) Å^3	
**Z**	8	
**Density (calculated)**	1.225 g/cm^3	
**Absorption coefficient**	0.077 mm^{-1}	
**F(000)**	1312	

Table S15: Data collection and structure refinement for 5r

**Diffractometer**	single crystal diffractometer Bruker D8 Venture Photon III	
**Radiation source**	micro focus tube Mo ImS (MoK α, λ = 0.71073 Å)	
**Theta range for data collection**	3.03 to 26.82°	
**Index ranges**	-34<=h<=34, -7<=k<=7, -30<=l<=30	
**Reflections collected**	39483	
**Independent reflections**	3564 [R(int) = 0.1282]	
**Coverage of independent reflections**	99.7%	
**Absorption correction**	multi-scan	
**Max. and min. transmission**	0.9960 and 0.9920	
**Structure solution technique**	direct methods	
**Structure solution program**	SHELXT 2019/1 (Sheldrick, 2018)	
**Refinement method**	Full-matrix least-squares on F^2	
**Refinement program**	SHELXL-2019/1 (Sheldrick, 2018)	
**Function minimized**	Σ w(F_o^2 - F_c^2)^2	
**Data / restraints / parameters**	3564 / 0 / 210	
**Goodness-of-fit on F^2**	1.048	
**Final R indices**	2601 data; I>2σ(I)	R1 = 0.0495, wR2 = 0.1170
	all data	R1 = 0.0719, wR2 = 0.1320
**Weighting scheme**	w=1/[σ^2(F_{o2})+(0.0437P)^2+3.3277P]where P=(F_o^2+2F_c^2)/3	
**Largest diff. peak and hole**	0.359 and -0.252 eÅ^{-3}	
**R.M.S. deviation from mean**	0.052 eÅ^{-3}	

4-(*tert*-Butyl)-1-methyl-3-phenyl-2,3,3a,8b-tetrahydro-1,3-methanocyclopenta[b]indole-1,4-dicarboxylate (5ad)

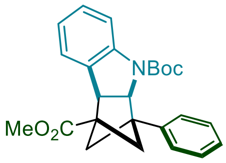

5ad

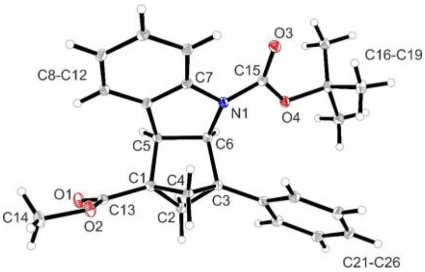

Figure S18: Crystal structure of compound 5ad. Thermal ellipsoids are shown at 30% probability

A colourless, prism-like specimen of **5ad** ($C_{25}H_{27}NO_4$), approximate dimensions 0.060 mm x 0.071 mm x 0.141 mm, was used for the X-ray crystallographic analysis. The X-ray intensity data were measured on a single crystal diffractometer Bruker D8 Venture Photon III system equipped with a micro focus tube Cu ImS (CuKα, λ = 1.54178 Å) and a MX mirror monochromator. A total of 1673 frames were collected. The total exposure time was 21.53 hours. The frames were integrated with the Bruker SAINT software package using a wide-frame algorithm. The integration of the data using a monoclinic unit cell yielded a total of 34902 reflections to a maximum θ angle of 68.30° (0.83 Å resolution), of which 3827 were independent (average redundancy 9.120, completeness = 99.7%, R_{int} = 8.50%, R_{sig} = 3.70%) and 2943 (76.90%) were greater than $2\sigma(F^2)$. The final cell constants of a = 17.7129(3) Å, b = 5.93870(10) Å, c = 21.6964(4) Å, β = 113.5340(10)°, volume = 2092.45(6) $Å^3$, are based upon the refinement of the XYZ-centroids of 9960 reflections above 20 σ(I) with 5.441° < 2θ < 136.2°. Data were corrected for absorption effects using the multi-scan method (SADABS). The ratio of minimum to maximum apparent transmission was 0.886. The calculated minimum and maximum transmission coefficients (based on crystal size) are 0.9080 and 0.9590. The structure was solved and refined using the Bruker SHELXTL Software Package, using the space group P 1 21/c 1, with Z = 4 for the formula unit, $C_{25}H_{27}NO_4$. The final anisotropic full-matrix least-squares refinement on F^2 with 275 variables converged at R1 = 4.10%, for the observed data and wR^2 = 9.78% for all data. The goodness-of-fit was 1.053. The largest peak in the final difference electron density synthesis was 0.195 e-/$Å^3$ and the largest hole was -0.225 e-/$Å^3$ with an RMS deviation of 0.047 $e^-/Å^3$. On the basis of the final model, the calculated density was 1.287 g/cm^3 and F(000), 864 e^-.

Table S16: Sample and crystal data for 5ad

**CCDC number**	2331781	
**Chemical formula**	$C_{25}H_{27}NO_4$	
**Formula weight**	405.47 g/mol	
**Temperature**	102(2) K	
**Wavelength**	1.54178 Å	
**Crystal size**	0.060 x 0.071 x 0.141 mm	
**Crystal habit**	colourless prism	
**Crystal system**	monoclinic	
**Space group**	P 1 21/c 1	
**Unit cell dimensions**	a = 17.7129(3) Å	α = 90°
	b = 5.93870(10) Å	β = 113.5340(10)°
	c = 21.6964(4) Å	λ = 90°
**Volume**	2092.45(6) $Å^3$	
**Z**	4	
**Density (calculated)**	1.287 g/cm^3	
**Absorption coefficient**	0.699 mm^{-1}	
**F(000)**	864	

Table S17: Data collection and structure refinement for 5ad

**Diffractometer**	single crystal diffractometer Bruker D8 Venture Photon III	
**Radiation source**	micro focus tube Cu ImS (CuKα, λ = 1.54178 Å)	
**Theta range for data collection**	2.72 to 68.30°	
**Index ranges**	-21<=h<=20, -7<=k<=7, -26<=l<=25	
**Reflections collected**	34902	
**Independent reflections**	3827 [R(int) = 0.0850]	
**Coverage of independent reflections**	99.7%	
**Absorption correction**	multi-scan	
**Max. and min. transmission**	0.9590 and 0.9080	
**Structure solution technique**	direct methods	
**Structure solution program**	SHELXT 2019/1 (Sheldrick, 2018)	
**Refinement method**	Full-matrix least-squares on F^2	
**Refinement program**	SHELXL-2019/1 (Sheldrick, 2018)	
**Function minimized**	$\Sigma w(F_o^2 - F_c^2)^2$	
**Data / restraints / parameters**	3827 / 0 / 275	
**Goodness-of-fit on F^2**	1.053	
**Final R indices**	2943 data; I>2σ(I)	R1 = 0.0410, wR2 = 0.0881
	all data	R1 = 0.0605, wR2 = 0.0978
**Weighting scheme**	$w=1/[\sigma^2 (F_o^2)+(0.0340P)^2+1.3157P]$ where $P=(F_o^2+2F_c^2)/3$	
**Largest diff. peak and hole**	0.195 and -0.225 $eÅ^{-3}$	
**R.M.S. deviation from mean**	0.047 $eÅ^{-3}$	

4-(*tert*-Butyl)-3-methyl-1-phenyl-1,2,3a,8b-tetrahydro-1,3-methanocyclopenta[b]indole-3,4-dicarboxylate (5ad′)

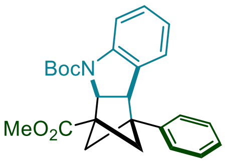

5ad′

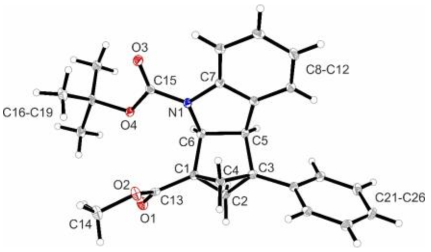

Figure S19: Crystal structure of compound 5ad′. Thermal ellipsoids are shown at 30% probability

A colourless, needle-like specimen of **5ad′** ($C_{25}H_{27}NO_4$), approximate dimensions 0.041 mm x 0.050 mm x 0.245 mm, was used for the X-ray crystallographic analysis. The X-ray intensity data were measured on a single crystal diffractometer Bruker D8 Venture Photon III system equipped with a micro focus tube Mo ImS (MoKα, λ = 0.71073 Å) and a MX mirror monochromator. A total of 558 frames were collected. The total exposure time was 3.88 hours. The frames were integrated with the Bruker SAINT software package using a wide-frame algorithm. The integration of the data using a monoclinic unit cell yielded a total of 41403 reflections to a maximum θ angle of 27.57° (0.77 Å resolution), of which 4735 were independent (average redundancy 8.744, completeness = 99.6%, R_{int} = 13.93%, R_{sig} = 7.23%) and 3076 (64.96%) were greater than 2σ(F^2). The final cell constants of a = 18.6543(11) Å, b = 6.0536(4) Å, c = 18.4219(11) Å, β = 98.653(2)°, volume = 2056.6(2) $Å^3$, are based upon the refinement of the XYZ-centroids of 5413 reflections above 20 σ(I) with 4.681° < 2θ < 54.95°. Data were corrected for absorption effects using the multi-scan method (SADABS). The ratio of minimum to maximum apparent transmission was 0.861. The calculated minimum and maximum transmission coefficients (based on crystal size) are 0.9790 and 9960. The structure was solved and refined using the Bruker SHELXTL Software Package, using the space group P 1 21/c 1, with Z = 4 for the formula unit, $C_{25}H_{27}NO_4$. The final anisotropic full-matrix least-squares refinement on F^2 with 276 variables converged at R1 = 5.80%, for the observed data and wR^2 = 13.78% for all data. The goodness-of-fit was 1.027. The largest peak in the final difference electron density synthesis was 0.321 e-/$Å^3$ and the largest hole was -0.326 e-/$Å^3$ with an RMS deviation of 0.067 e^-/$Å^3$. On the basis of the final model, the calculated density was 1.310 g/cm^3 and F(000), 864 e^-.

Table S18: Sample and crystal data for 5ad′

**CCDC number**	2331782	
**Chemical formula**	$C_{25}H_{27}NO_4$	
**Formula weight**	405.47 g/mol	
**Temperature**	102(2) K	
**Wavelength**	0.71073 Å	
**Crystal size**	0.041 x 0.050 x 0.245 mm	
**Crystal habit**	colourless needle	
**Crystal system**	monoclinic	
**Space group**	P 1 21/c 1	
**Unit cell dimensions**	a = 18.6543(11) Å	α = 90°
	b = 6.0536(4) Å	β = 98.653(2)°
	c = 18.4219(11) Å	λ = 90°
**Volume**	2056.6(2) $Å^3$	
**Z**	4	
**Density (calculated)**	1.310 g/cm^3	
**Absorption coefficient**	0.088 mm^{-1}	
**F(000)**	864	

Table S19: Data collection and structure refinement for 5ad′

**Diffractometer**	single crystal diffractometer Bruker D8 Venture Photon III	
**Radiation source**	micro focus tube Mo ImS (MoKα, λ = 0.71073 Å)	
**Theta range for data collection**	2.64 to 27.57°	
**Index ranges**	-24<=h<=24, -7<=k<=7, -23<=l<=23	
**Reflections collected**	41403	
**Independent reflections**	4735 [R(int) = 0.1393]	
**Coverage of independent reflections**	99.6%	
**Absorption correction**	multi-scan	
**Max. and min. transmission**	0.9960 and 0.9790	
**Structure solution technique**	direct methods	
**Structure solution program**	SHELXT 2019/1 (Sheldrick, 2018)	
**Refinement method**	Full-matrix least-squares on F^2	
**Refinement program**	SHELXL-2019/1 (Sheldrick, 2018)	
**Function minimized**	$\Sigma\ w(F_o^2 - F_c^2)^2$	
**Data / restraints / parameters**	4735 / 0 / 276	
**Goodness-of-fit on F^2**	1.027	
**Final R indices**	3076 data; I>2σ(I)	R1 = 0.0580, wR2 = 0.1191
	all data	R1 = 0.1030, wR2 = 0.1378
**Weighting scheme**	$w=1/[\sigma^2(F_o^2)+(0.0504P)^2+1.3157P]$ where $P=(F_o^2+2F_c^2)/3$	
**Largest diff. peak and hole**	0.321 and -0.326 $eÅ^{-3}$	
**R.M.S. deviation from mean**	0.067 $eÅ^{-3}$	

N-methoxy-N-methyl-1-phenyl-1,2,3a,4,5,9b-hexahydro-3H-1,3-methanocyclopenta[a]naphthalene-3-carboxamide (5au)

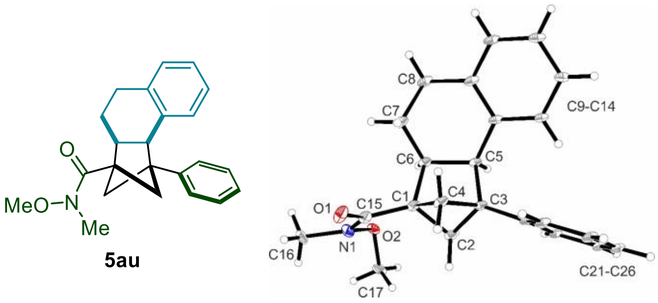

Figure S20: Crystal structure of compound 5au. Thermal ellipsoids are shown at 30% probability

A colourless, plate-like specimen of **5au** ($C_{23}H_{25}NO_2$), approximate dimensions 0.074 mm x 0.168 mm x 0.220 mm, was used for the X-ray crystallographic analysis. The X-ray intensity data were measured on a single crystal diffractometer Bruker D8 Venture Photon III system equipped with a micro focus tube Cu ImS (CuKα, λ = 1.54178 Å) and a MX mirror monochromator. A total of 2080 frames were collected. The total exposure time was 22.22 hours. The frames were integrated with the Bruker SAINT software package using a wide-frame algorithm. The integration of the data using a monoclinic unit cell yielded a total of 23821 reflections to a maximum θ angle of 66.72° (0.84 Å resolution), of which 6517 were independent (average redundancy 3.655, completeness = 99.4%, R_{int} = 7.40%, R_{sig} = 5.71%) and 4798 (73.62%) were greater than 2σ(F^2). The final cell constants of a = 11.9235(11) Å, b = 8.0701(6) Å, c = 19.558(2) Å, β = 98.861(7)°, volume = 1859.5(3) $Å^3$, are based upon the refinement of the XYZ-centroids of 6961 reflections above 20 σ(I) with 4.572° < 2θ < 132.7°. Data were corrected for absorption effects using the multi-scan method (SADABS). The ratio of minimum to maximum apparent transmission was 0.893. The calculated minimum and maximum transmission coefficients (based on crystal size) are 0.8760 and 0.9560.

The structure was solved and refined using the Bruker SHELXTL Software Package, using the space group P 1 21 1, with Z = 4 for the formula unit, $C_{23}H_{25}NO_2$. The final anisotropic full-matrix least-squares refinement on F^2 with 474 variables converged at R1 = 5.07%, for the observed data and wR^2 = 13.12% for all data. The goodness-of-fit was 1.037. The largest peak in the final difference electron density synthesis was 0.177 $e^-/Å^3$ and the largest hole was -0.185 $e^-/Å^3$ with an RMS deviation of 0.037 $e^-/Å^3$. On the basis of the final model, the calculated density was 1.241 g/cm^3 and F(000), 744 e^-.

Table S20: Sample and crystal data for 5au

**CCDC number**	2331783	
**Chemical formula**	$C_{23}H_{25}NO_2$	
**Formula weight**	347.44 g/mol	
**Temperature**	100(2) K	
**Wavelength**	1.54178 Å	
**Crystal size**	0.074 x 0.168 x 0.220 mm	
**Crystal habit**	colourless plate	
**Crystal system**	monoclinic	
**Space group**	P 1 21 1	
**Unit cell dimensions**	a = 11.9235(11) Å	α = 90°
	b = 8.0701(6) Å	β = 98.861(7)°
	c = 19.558(2) Å	λ = 90°
**Volume**	1859.5(3) Å^3	
**Z**	4	
**Density (calculated)**	1.241 g/cm^3	
**Absorption coefficient**	0.616 mm^{-1}	
**F(000)**	744	

Table S21: Data collection and structure refinement for 5au

**Diffractometer**	single crystal diffractometer Bruker D8 Venture Photon III	
**Radiation source**	micro focus tube Cu ImS (CuKα, λ = 1.54178 Å)	
**Theta range for data collection**	2.29 to 66.72°	
**Index ranges**	-14<=h<=14, -9<=k<=9, -22<=l<=23	
**Reflections collected**	23821	
**Independent reflections**	6517 [R(int) = 0.0740]	
**Coverage of independent reflections**	99.4%	
**Absorption correction**	multi-scan	
**Max. and min. transmission**	0.9560 and 0.8760	
**Structure solution technique**	direct methods	
**Structure solution program**	SHELXT 2019/1 (Sheldrick, 2018)	
**Refinement method**	Full-matrix least-squares on F^2	
**Refinement program**	SHELXL-2019/1 (Sheldrick, 2018)	
**Function minimized**	Σ w(F_o^2 - F_c^2) ^{2}	
**Data / restraints / parameters**	6517 / 313 / 474	
**Goodness-of-fit on F^2**	1.037	
**Final R indices**	4798 data; I>2σ(I)	R1 = 0.0507, wR2 = 0.1144
	all data	R1 = 0.0786, wR2 = 0.1312
**Weighting scheme**	w=1/[σ^2 (F_o^2)+(0.0499P)^2+0.6338P]where P=(F_o^2+2F_c^2)/3	
**Absolute structure parameter**	0.0(2)	
**Extinction coefficient**	0.0072(8)	
**Largest diff. peak and hole**	0.177 and -0.185 eÅ^{-3}	
**R.M.S. deviation from mean**	0.037 eÅ^{-3}	

6. SPECTROSCOPIC DATA

^{1}H NMR (500 MHz, $CDCl_3$) of **2u** (*see procedure*)

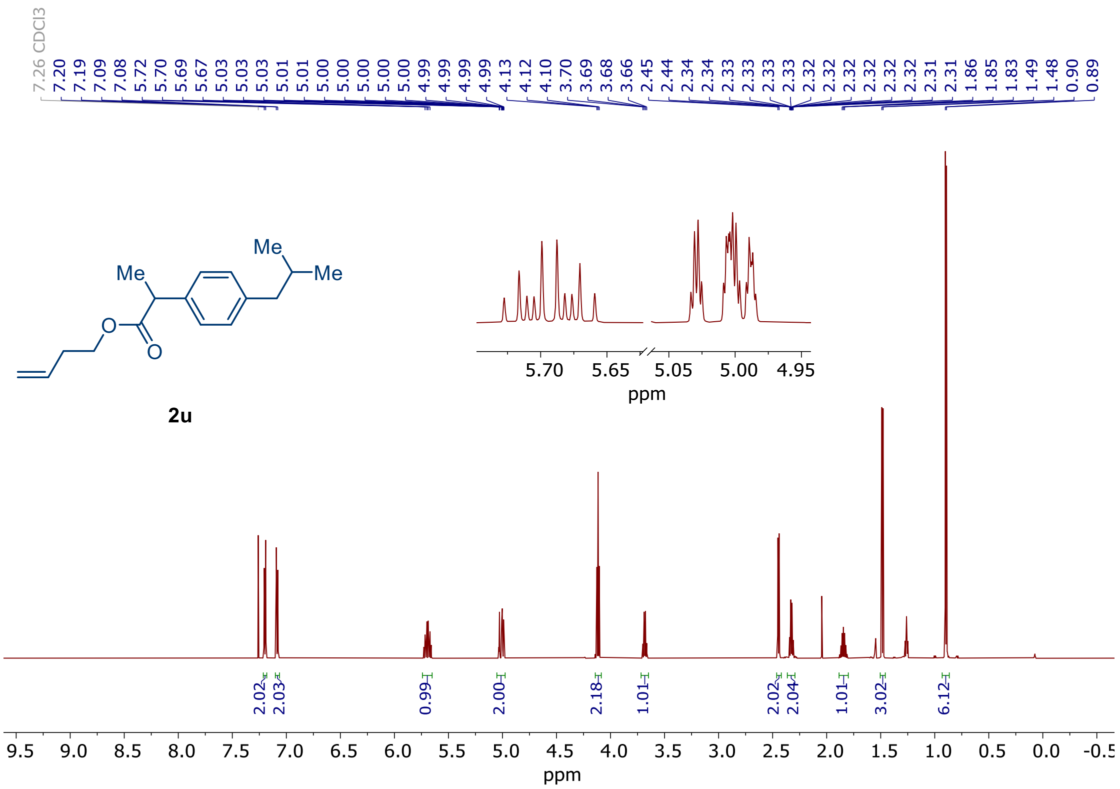

^{13}C NMR (126 MHz, $CDCl_3$) of **2u**

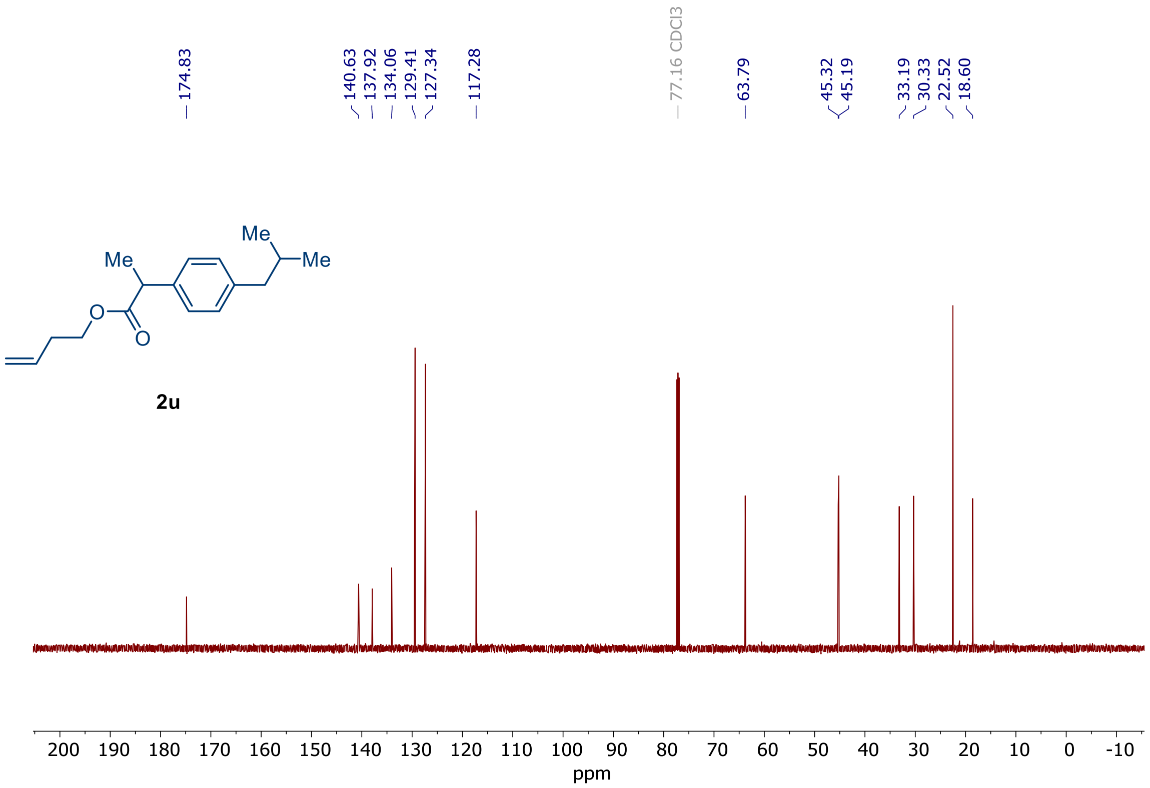

^{1}H NMR (400 MHz, $CDCl_3$) of **2v** (*see procedure*)

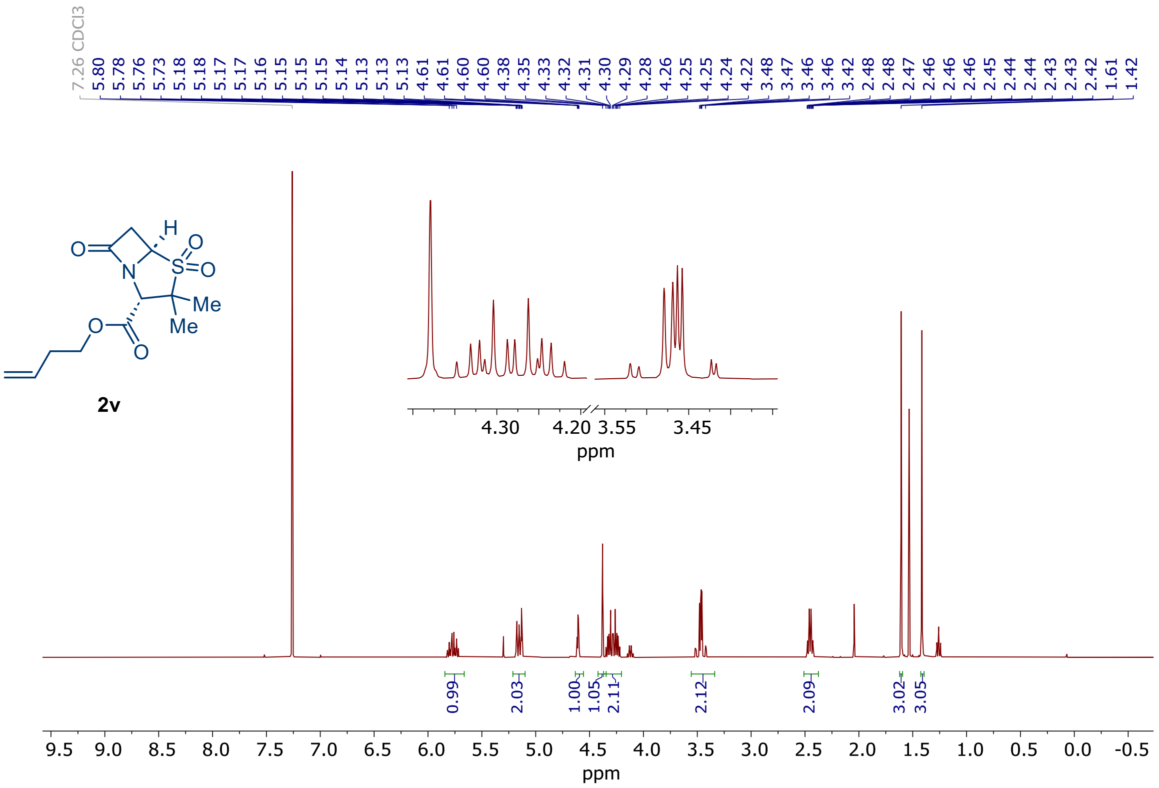

^{13}C NMR (101 MHz, $CDCl_3$) of **2v**

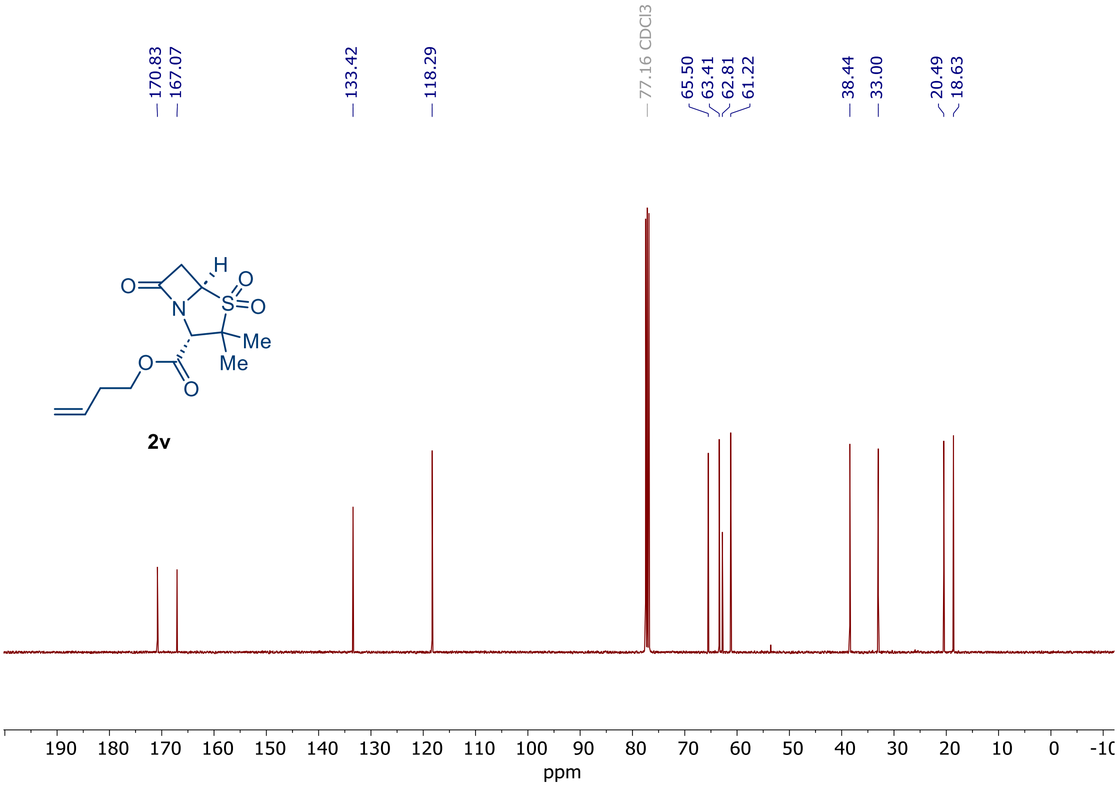

^{1}H NMR (400 MHz, $CDCl_3$) of **4ae** (*see procedure*)

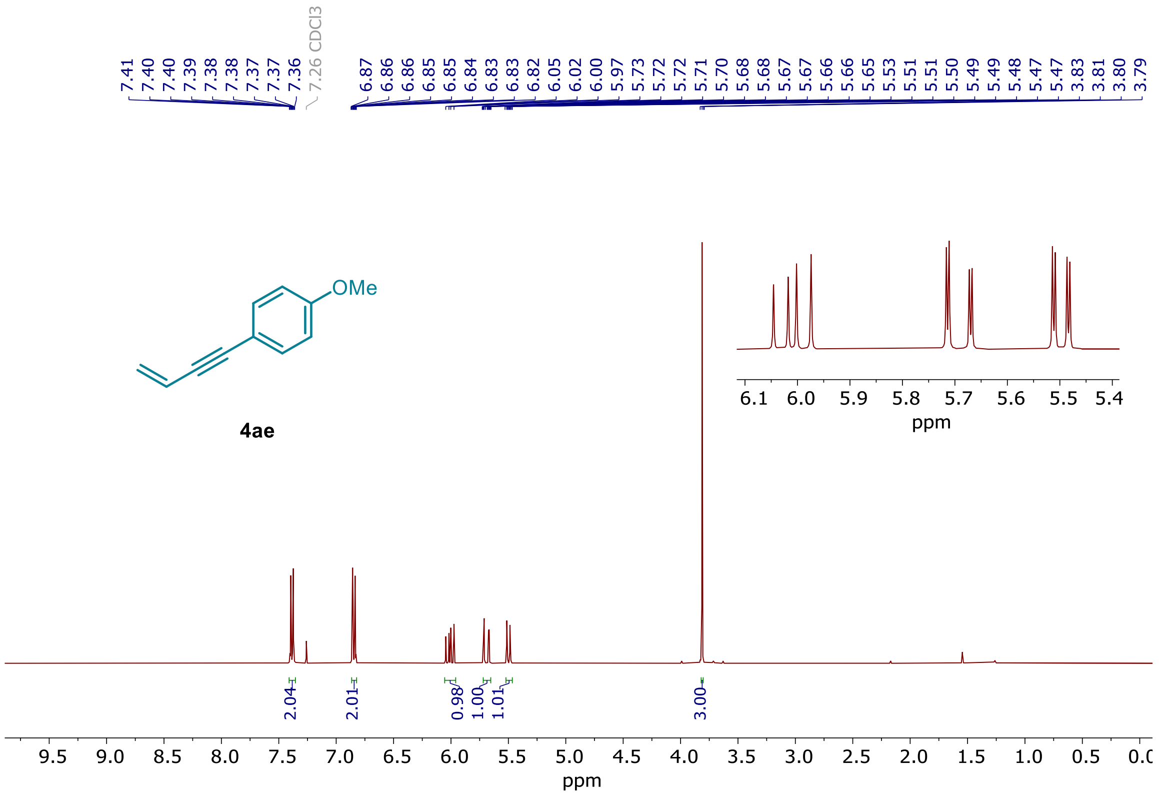

^{13}C NMR (101 MHz, $CDCl_3$) of **4ae**

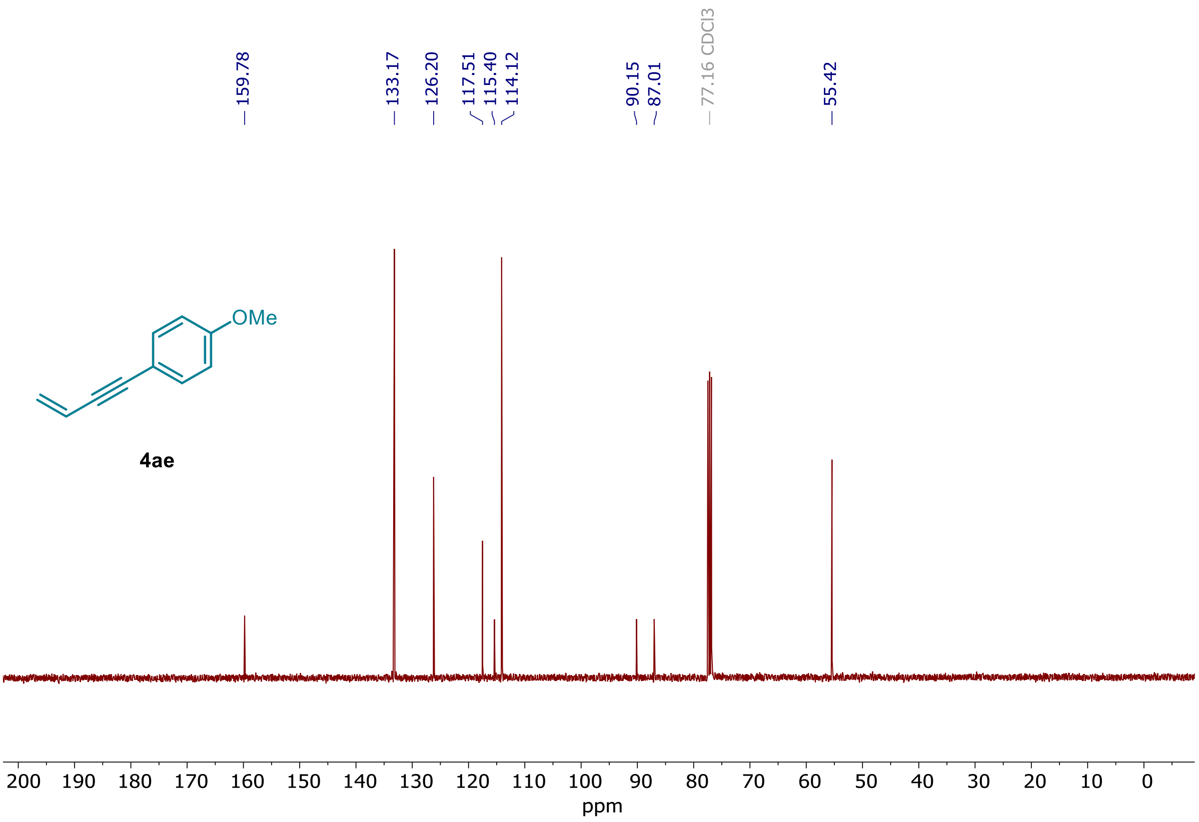

^{1}H NMR (400 MHz, $CDCl_3$) of **4af** (*see procedure*)

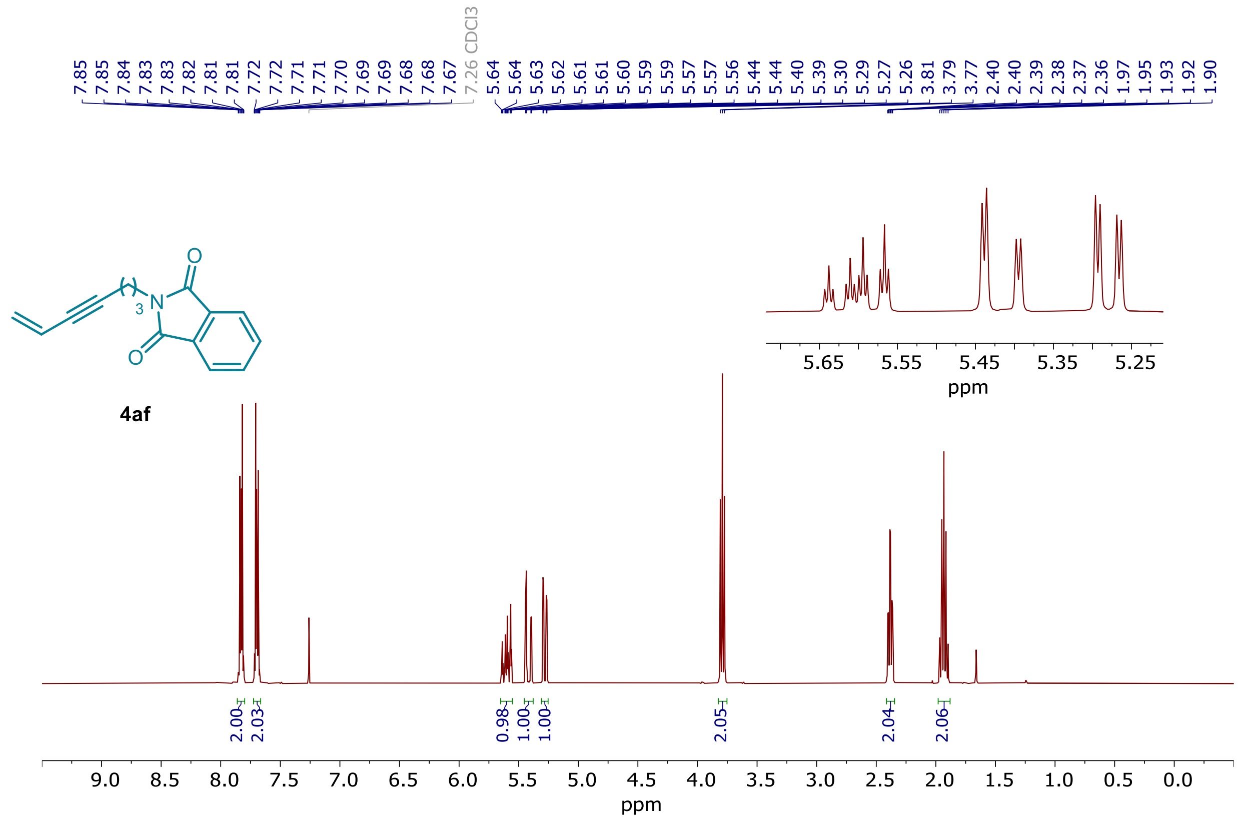

^{13}C NMR (101 MHz, $CDCl_3$) of **4af**

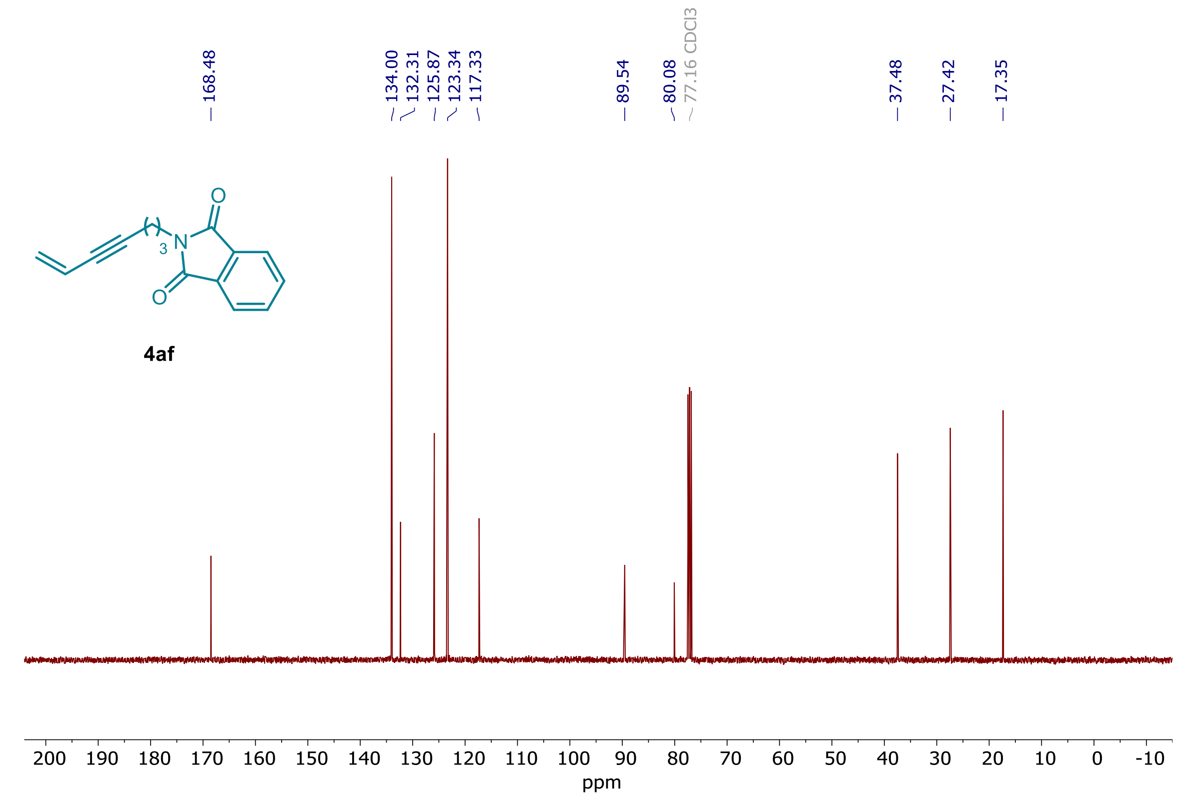

^{1}H NMR (400 MHz, $CDCl_3$) of **1a** (see procedure)

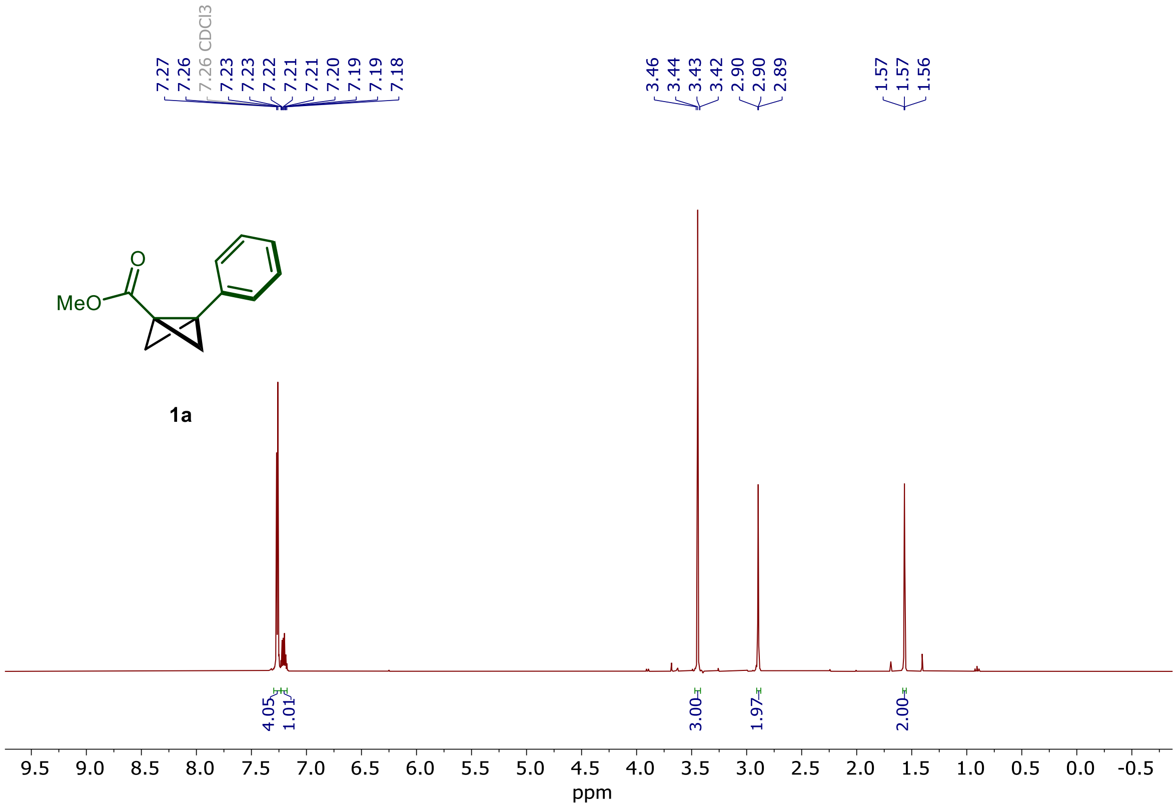

^{13}C NMR (101 MHz, $CDCl_3$) of **1a**

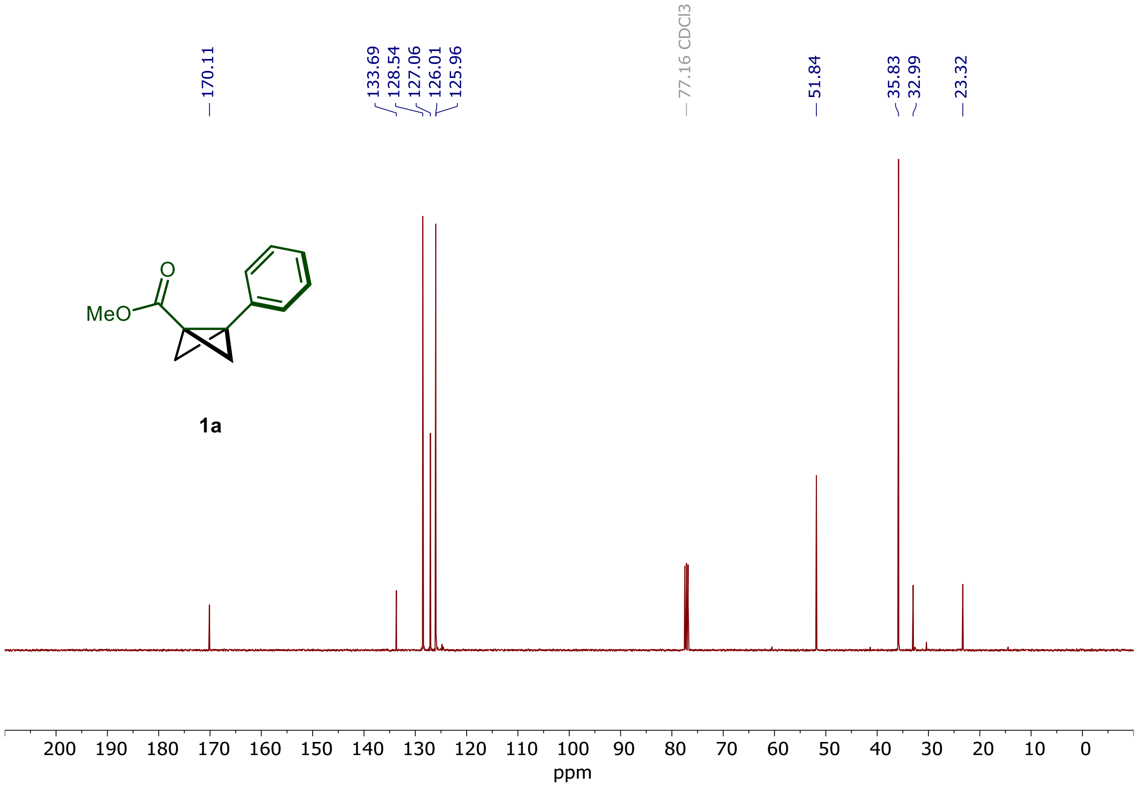

^{1}H NMR (400 MHz, $CDCl_3$) of **1av** (*see procedure*)

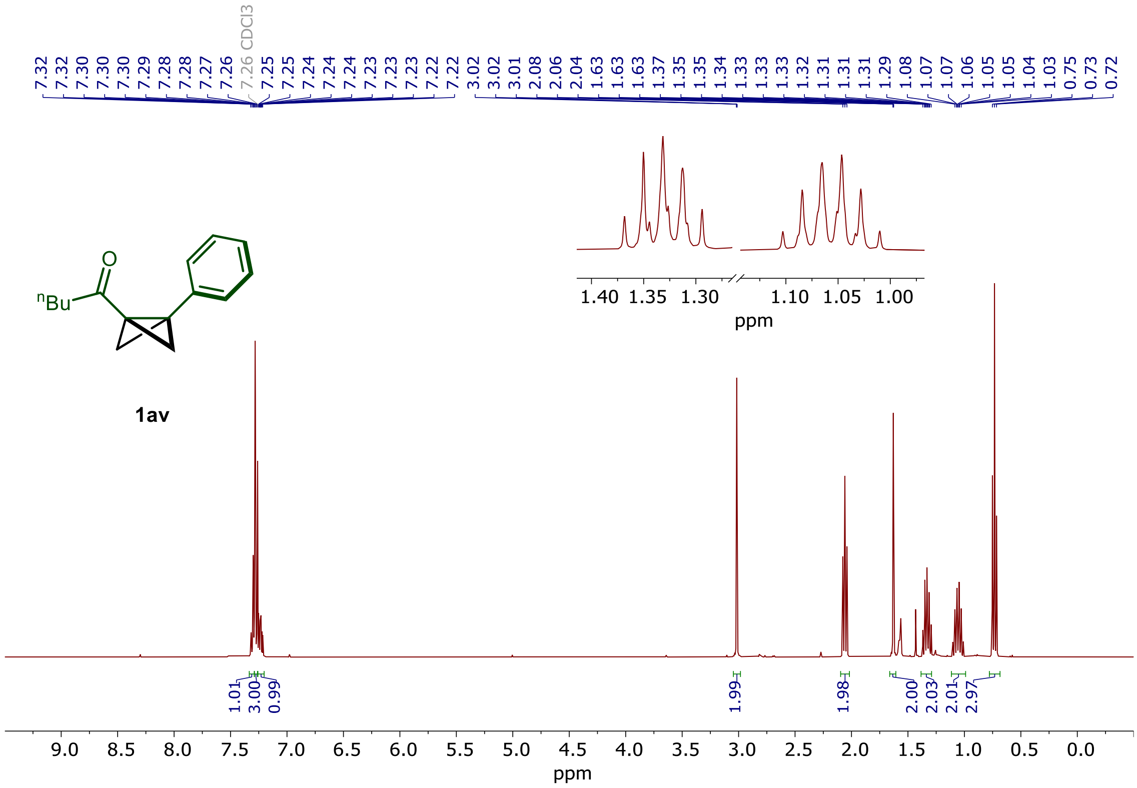

^{13}C NMR (101 MHz, $CDCl_3$) of **1av**

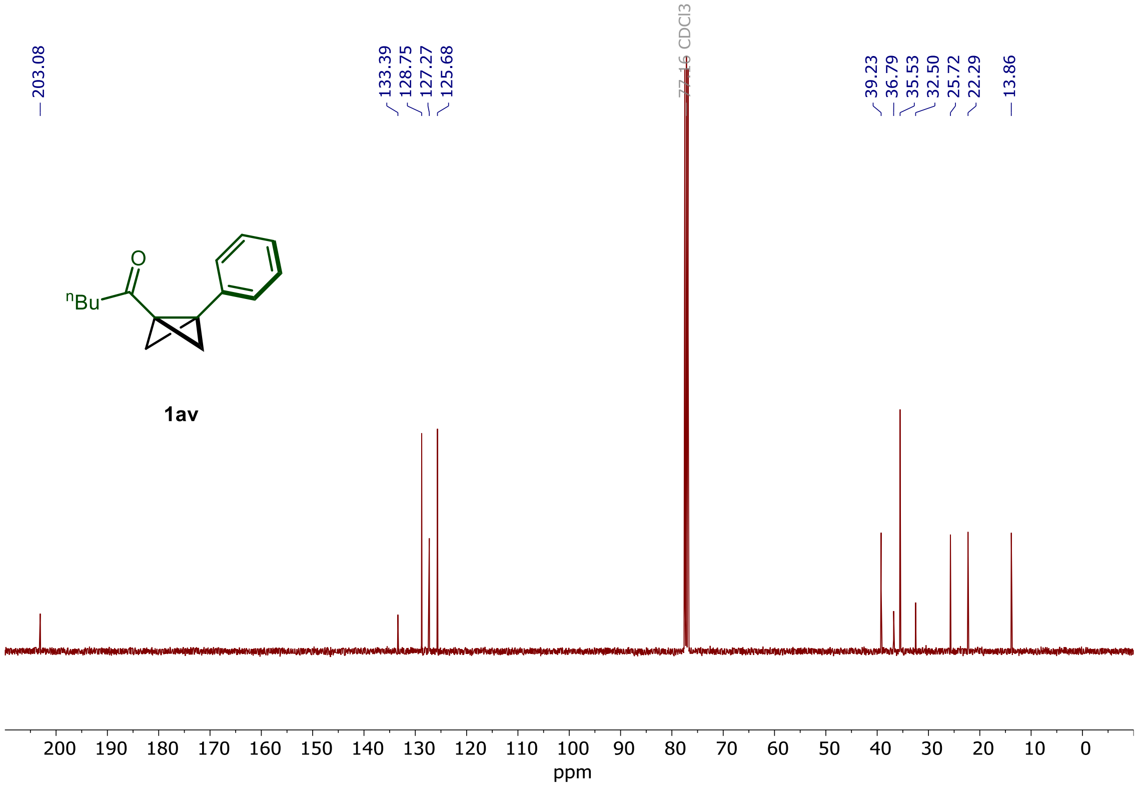

^{1}H NMR (400 MHz, CDCl3) of **1aw** (*see procedure*)

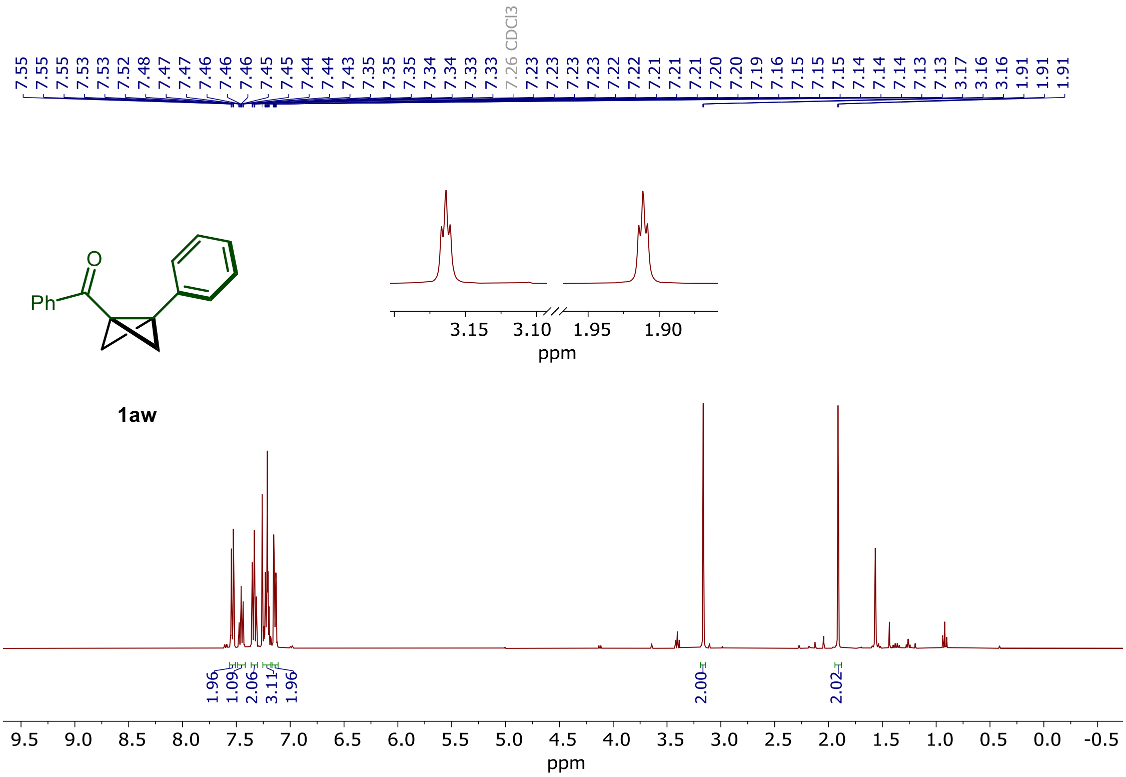

^{13}C NMR (101 MHz, CDCl3) of **1aw**

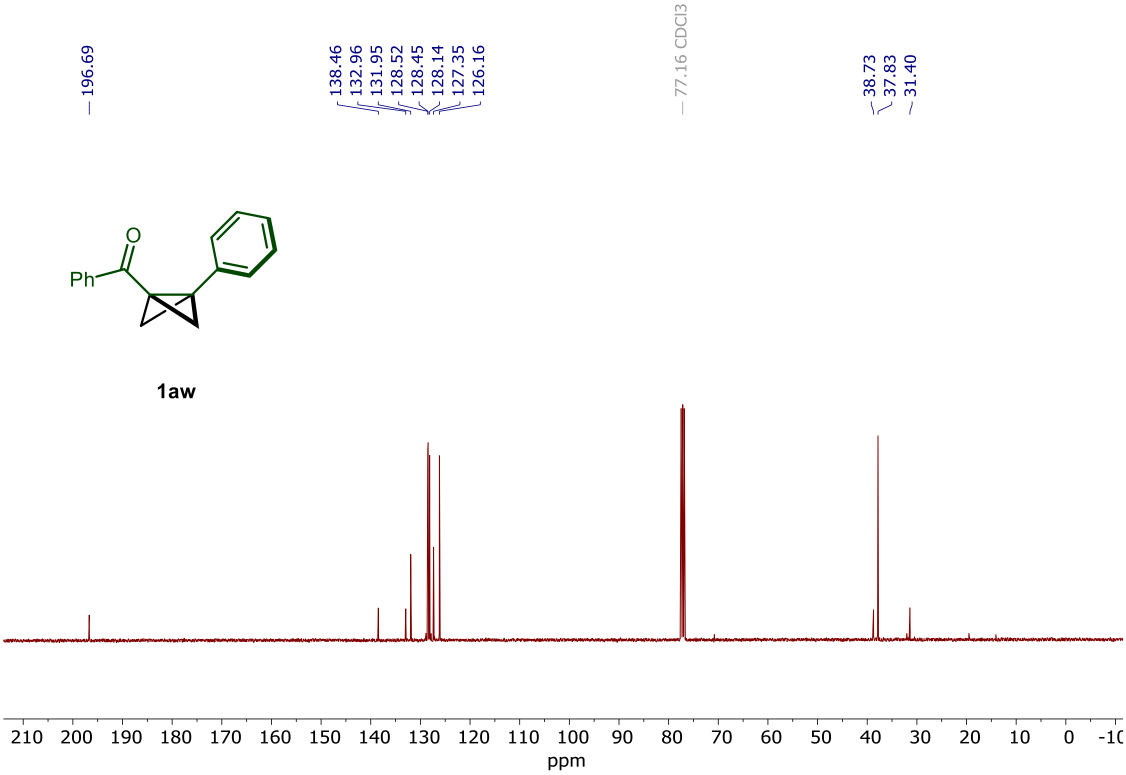

^{1}H NMR (400 MHz, $CDCl_3$) of **1ax** (*see procedure*)

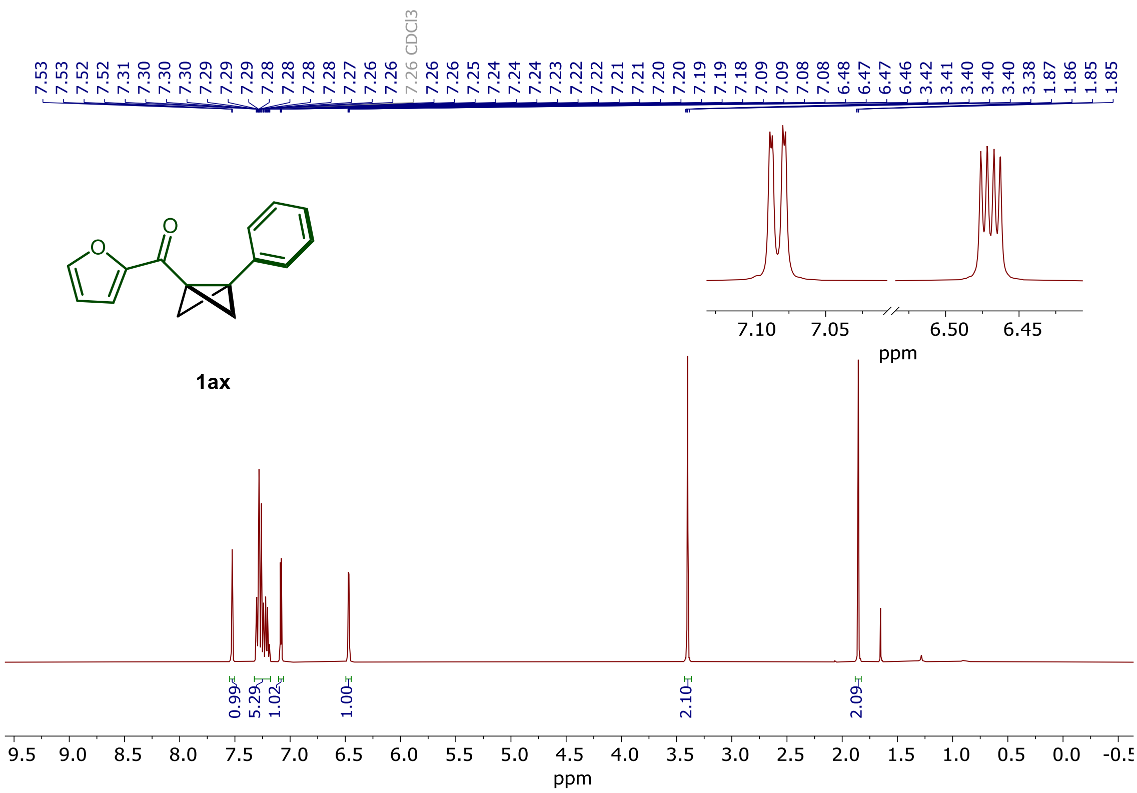

^{13}C NMR (101 MHz, $CDCl_3$) of **1ax**

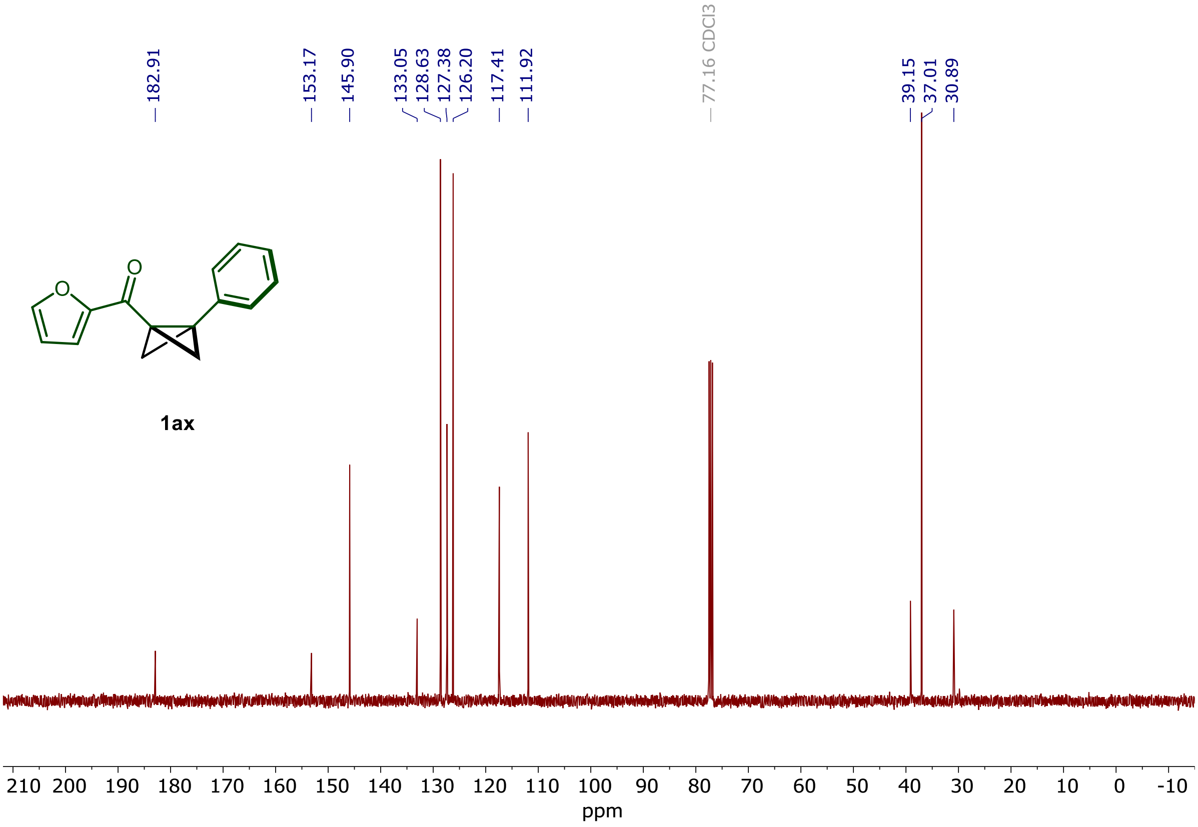

^{1}H NMR (400 MHz, $CDCl_3$) of **3a** (*see procedure*)

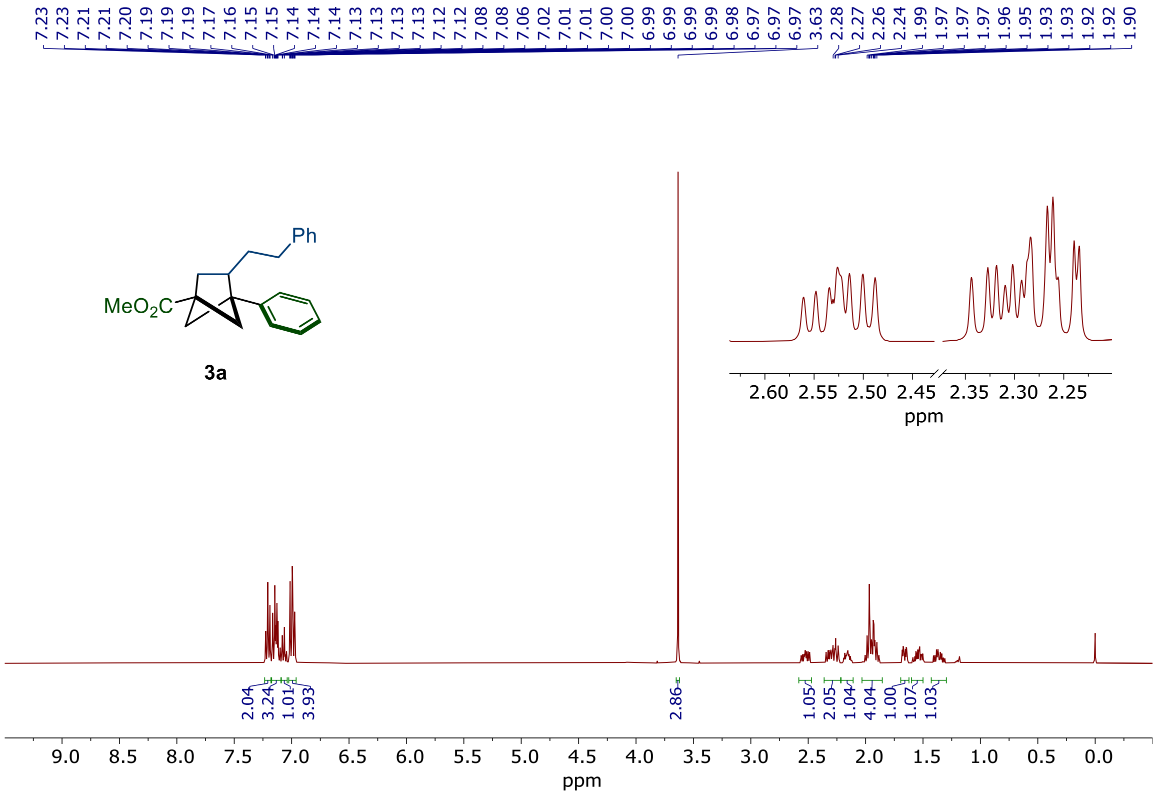

^{13}C NMR (101 MHz, $CDCl_3$) of **3a**

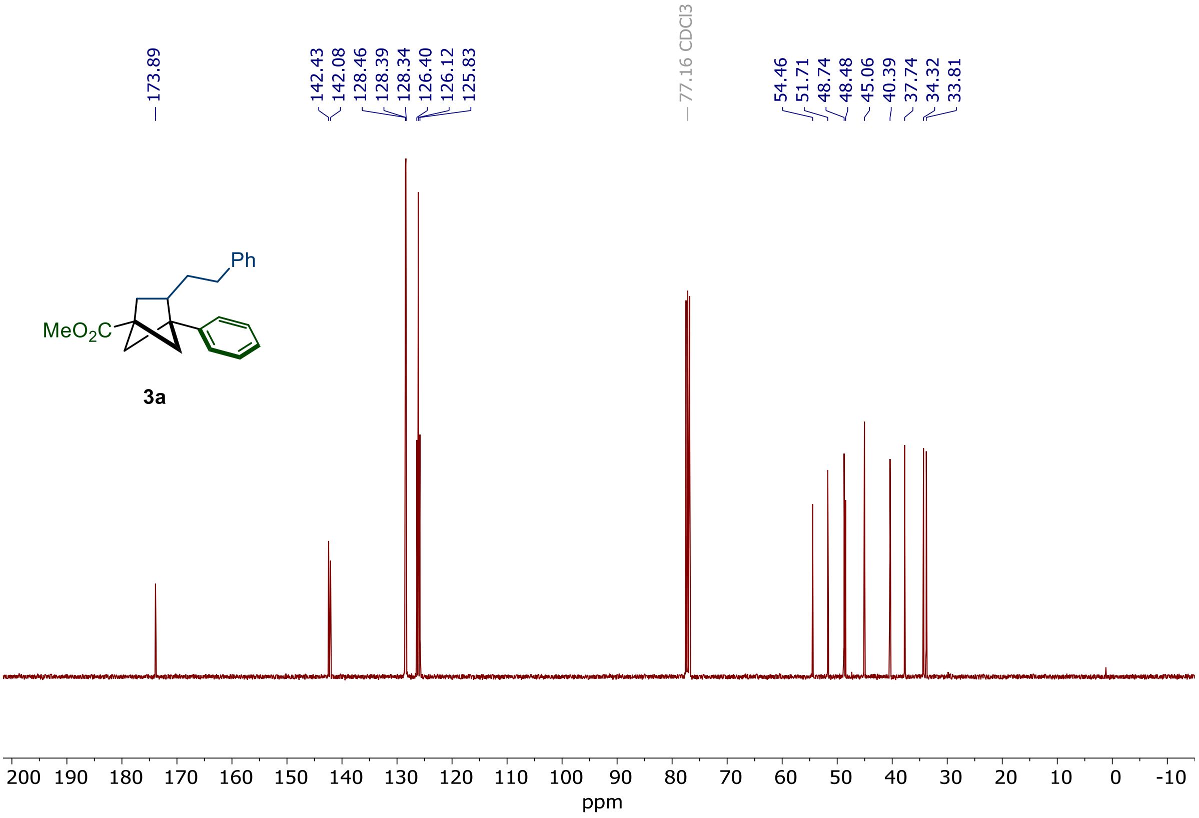

^{1}H NMR (400 MHz, $CDCl_3$) of **3b** (*see procedure*)

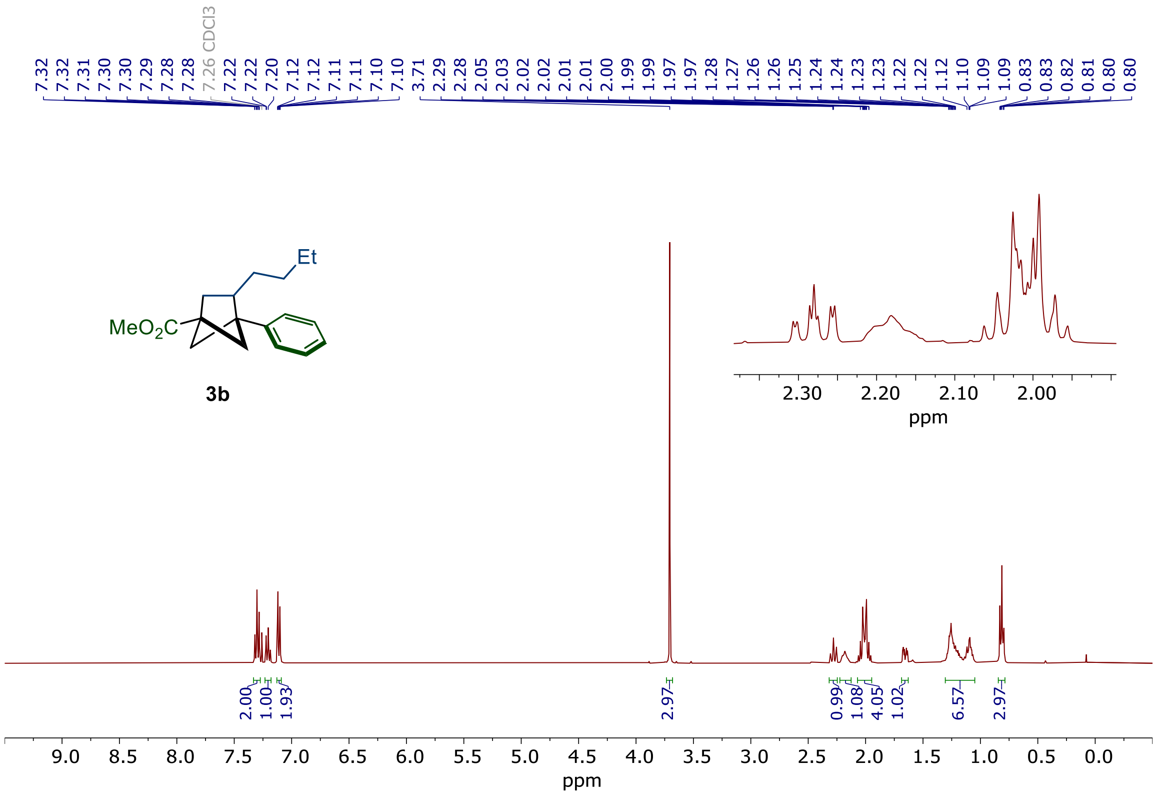

^{13}C NMR (101 MHz, $CDCl_3$) of **3b**

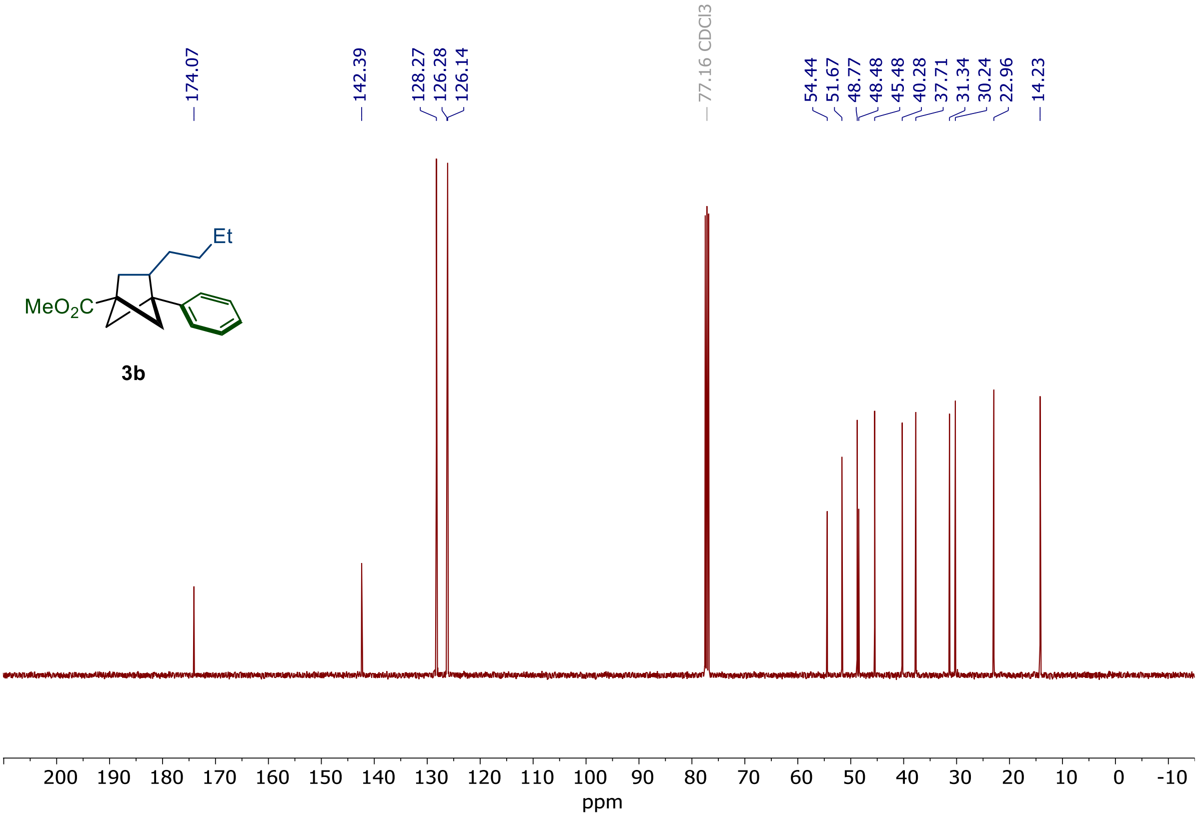

^{1}H NMR (500 MHz, $CDCl_3$) of **3c** (*see procedure*)

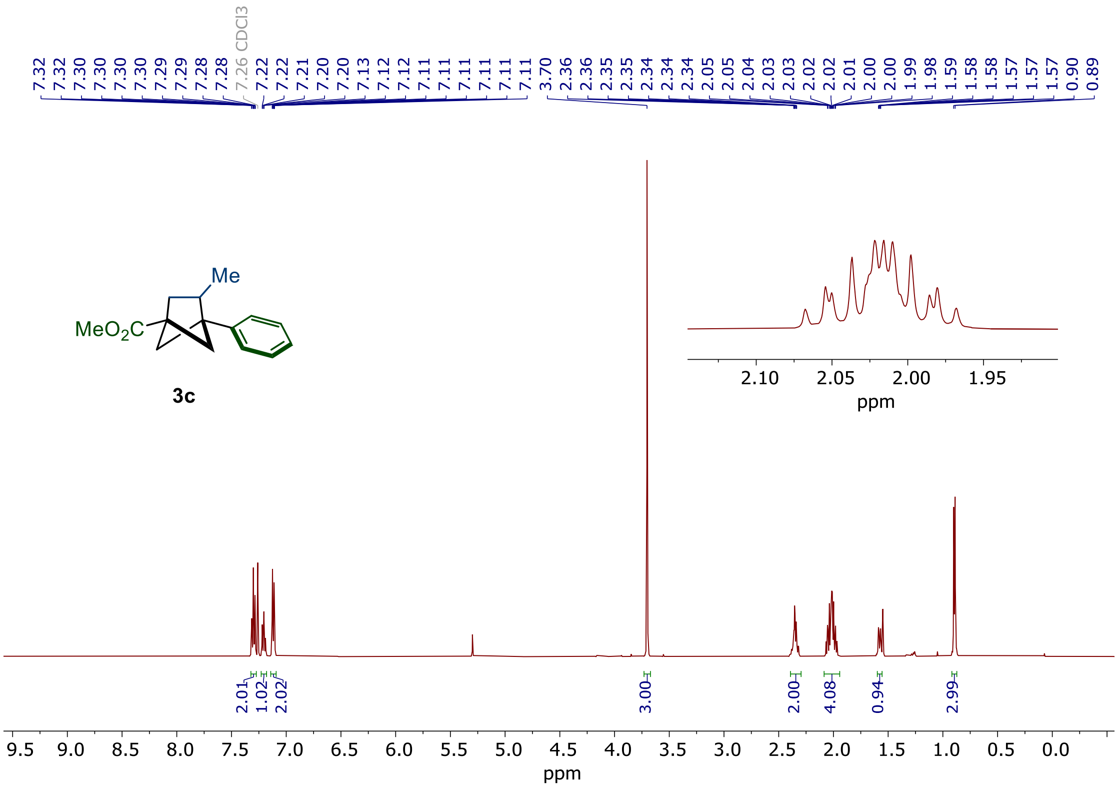

^{13}C NMR (126 MHz, $CDCl_3$) of **3c**

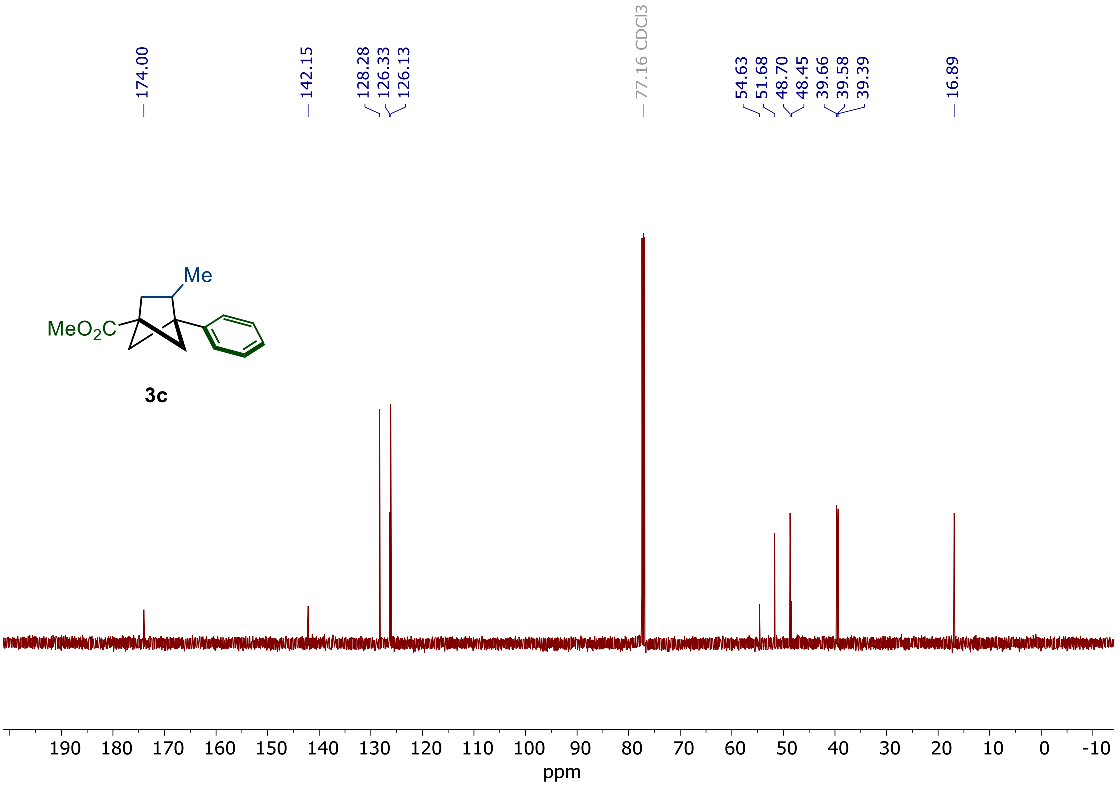

^{1}H NMR (400 MHz, $CDCl_3$) of **3d** (*see procedure*)

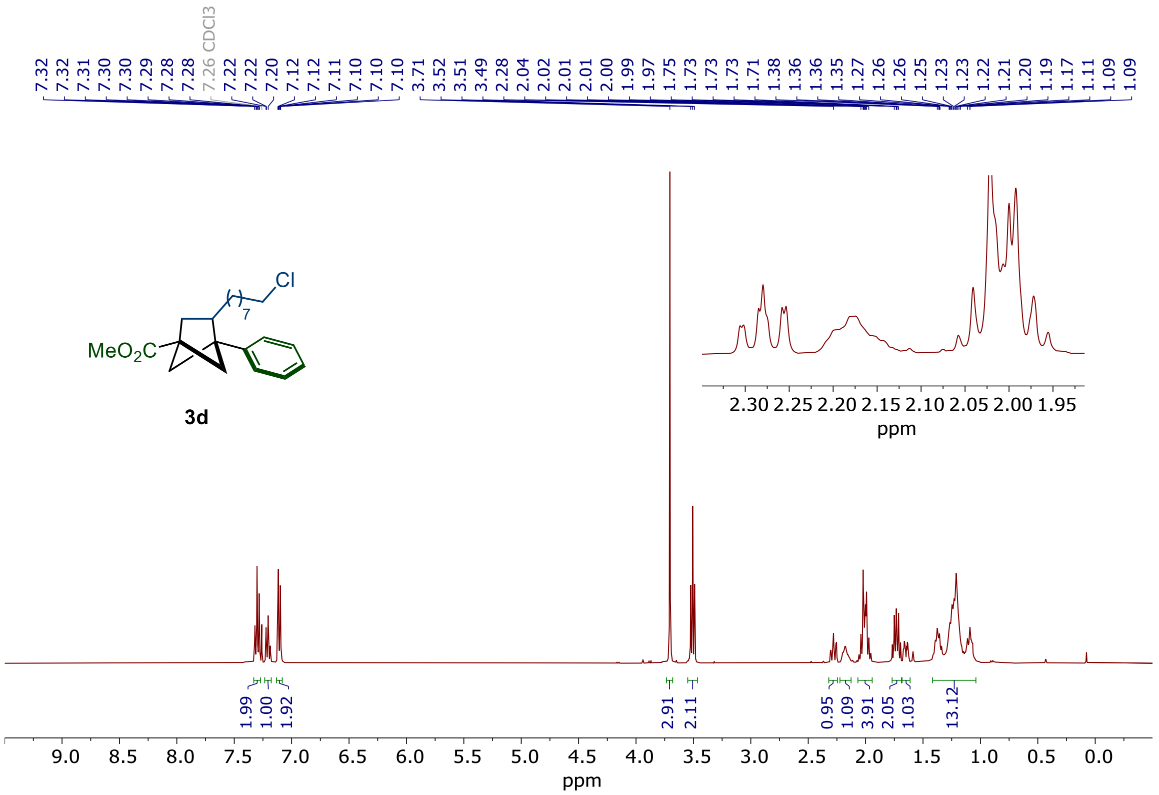

^{13}C NMR (101 MHz, $CDCl_3$) of **3d**

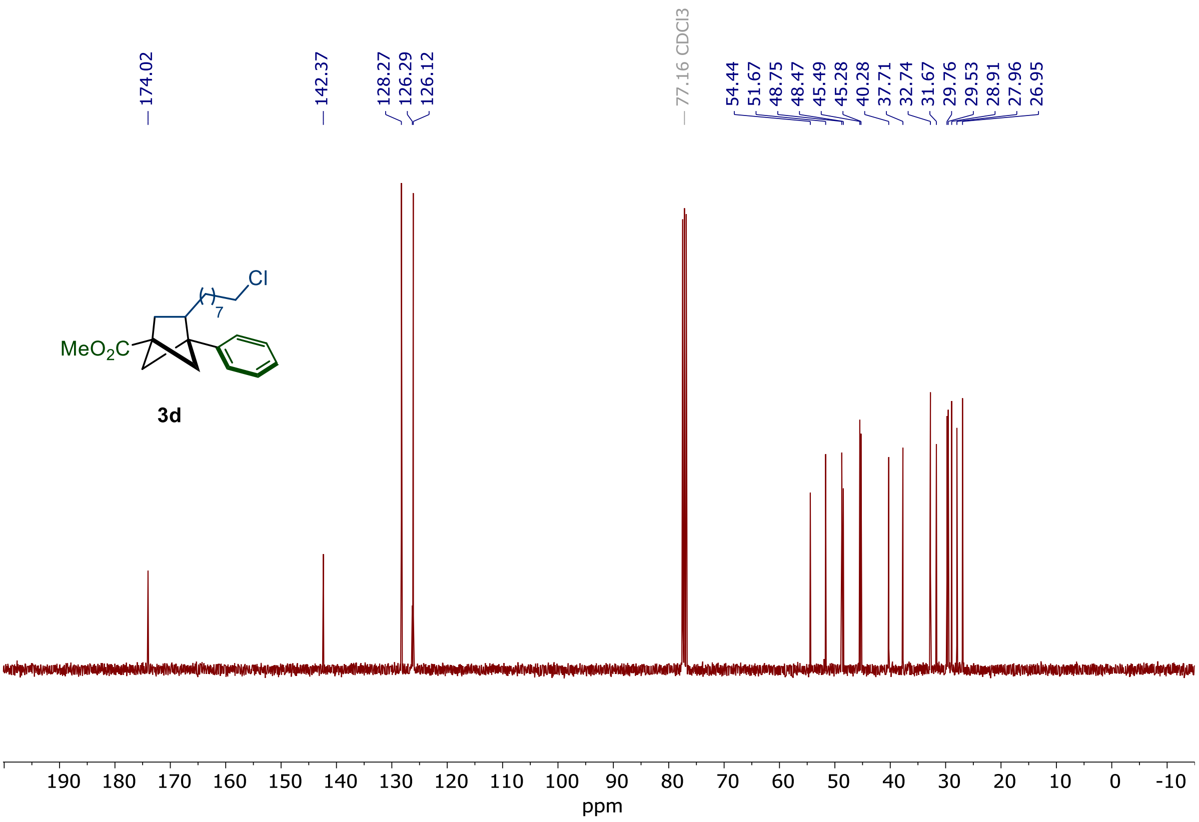

^{1}H NMR (500 MHz, $CDCl_3$) of **3e** (see procedure)

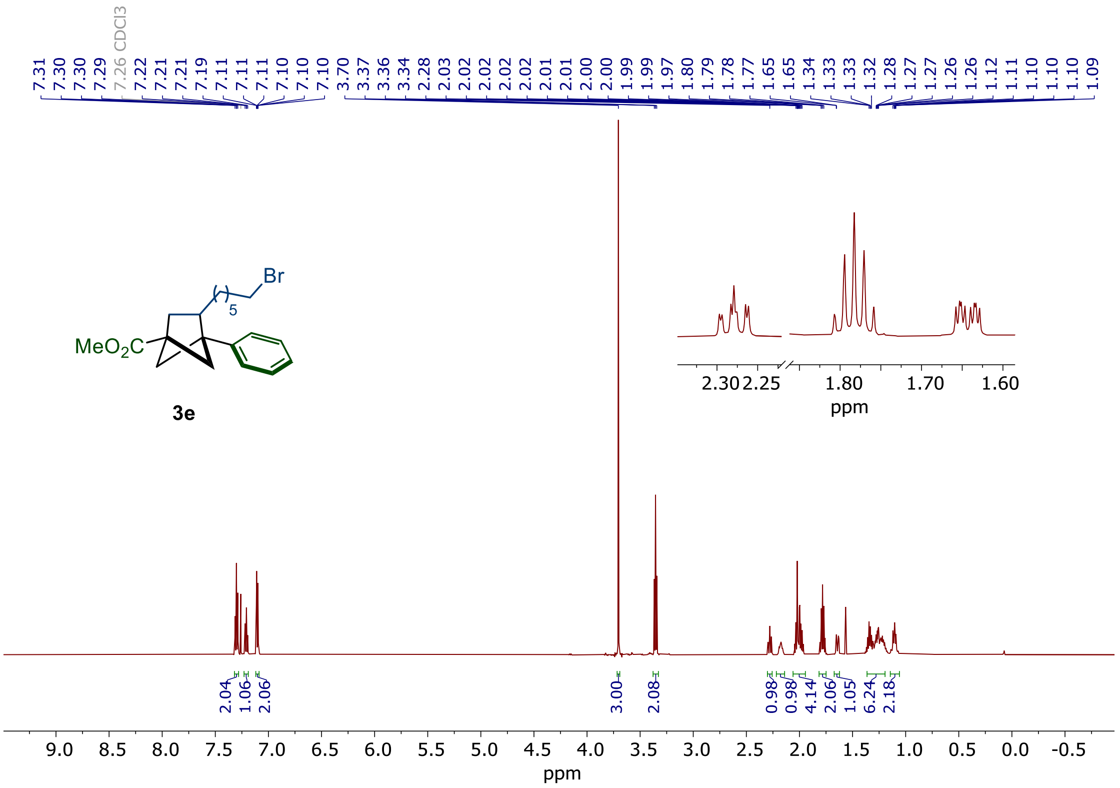

^{13}C NMR (126 MHz, $CDCl_3$) of **3e**

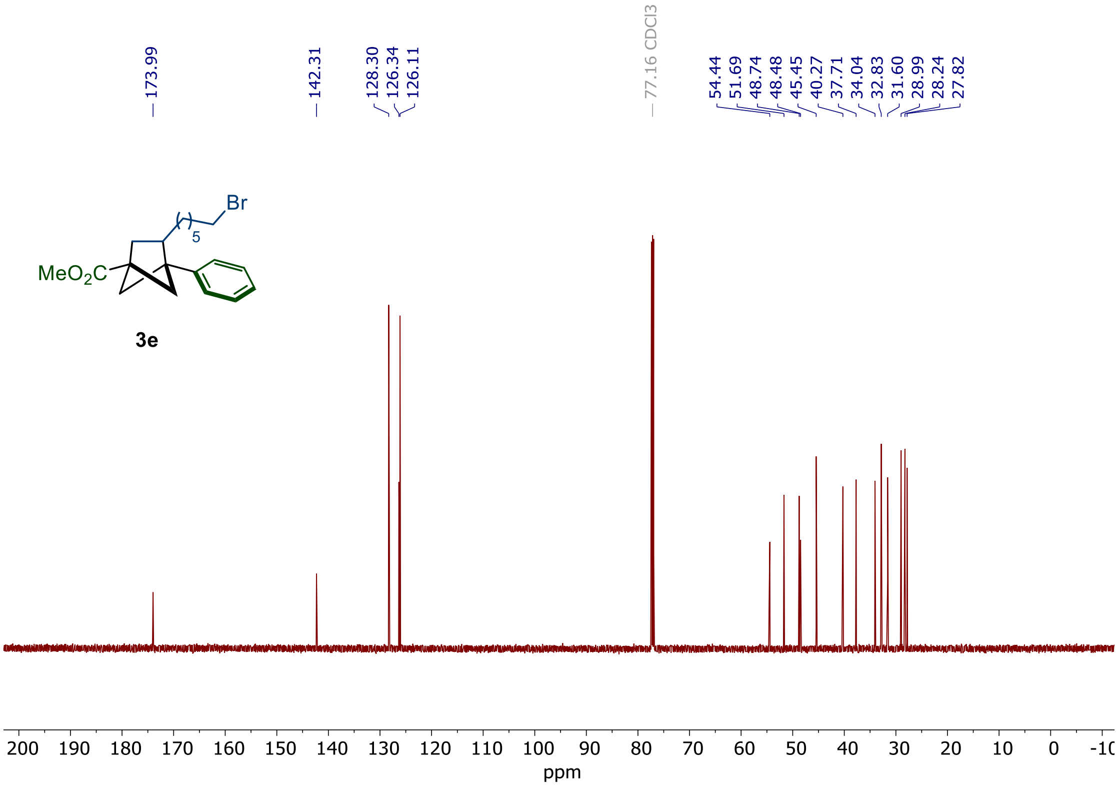

^{1}H NMR (400 MHz, $CDCl_3$) of **3f** (*see procedure*)

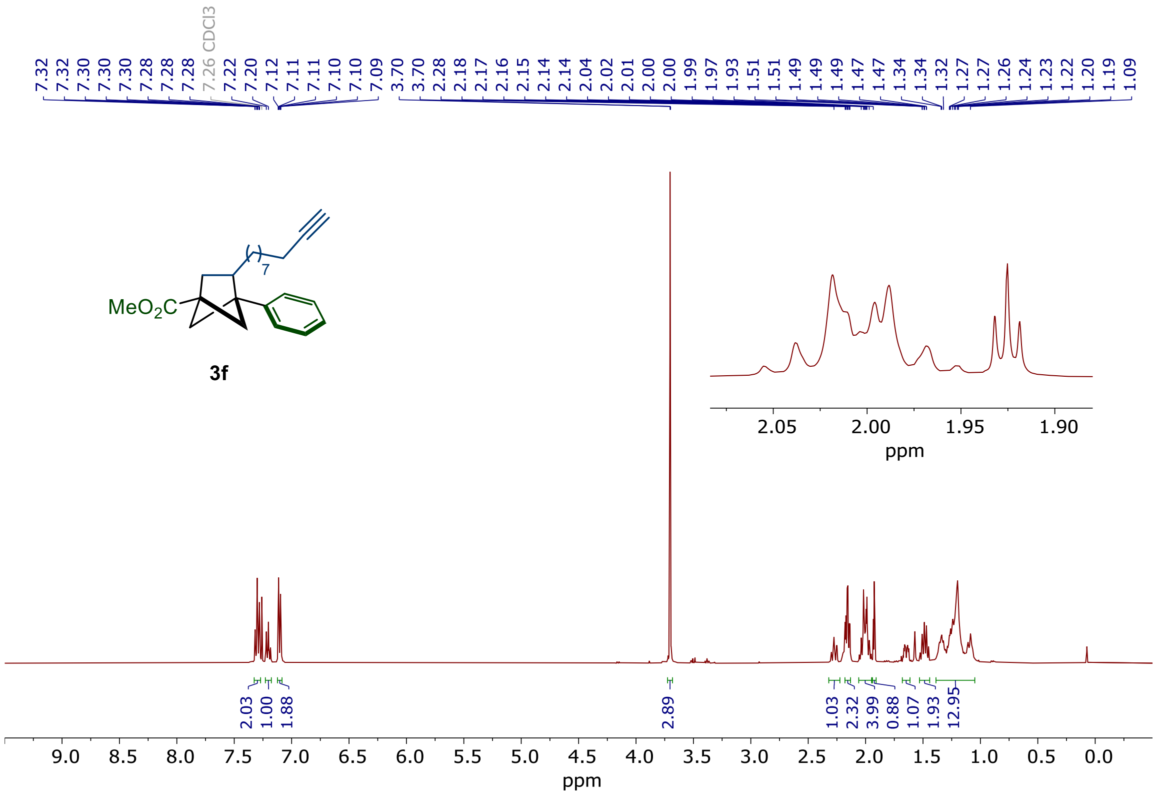

^{13}C NMR (101 MHz, $CDCl_3$) of **3f**

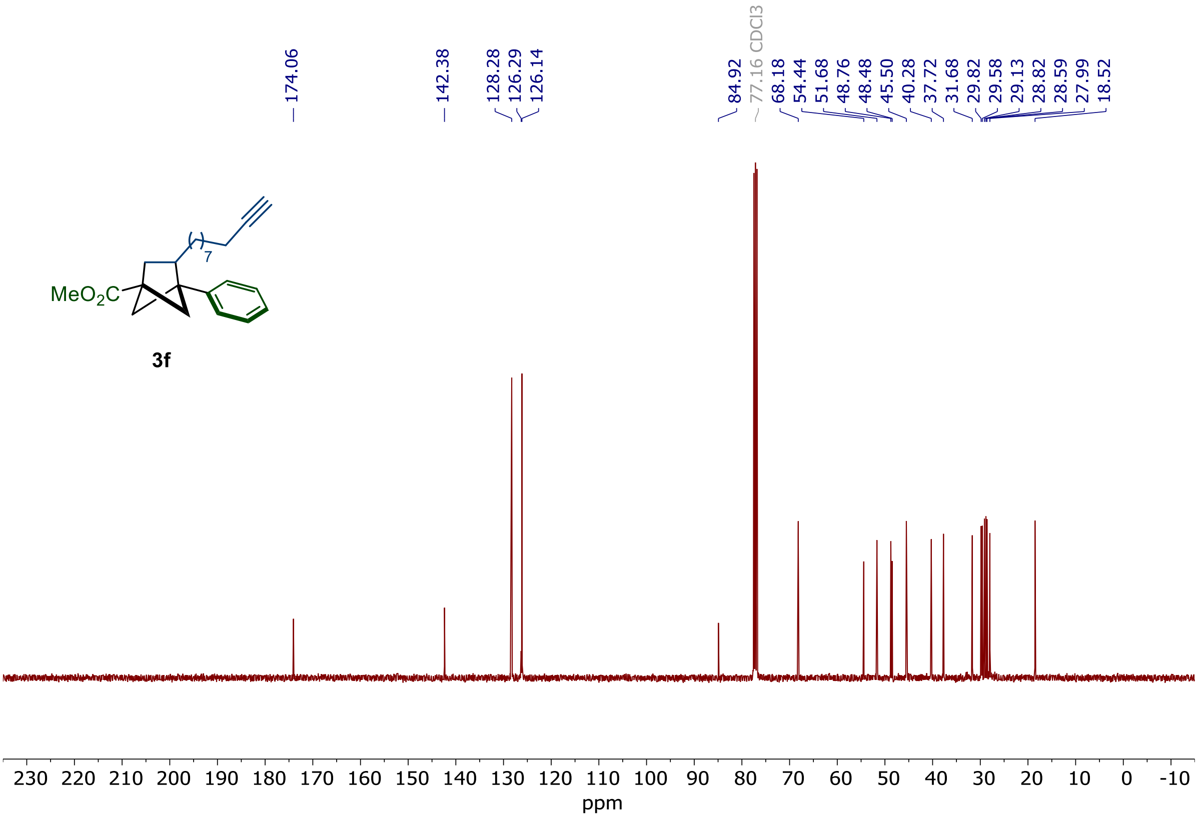

^{1}H NMR (500 MHz, $CDCl_3$) of **3g** (*see procedure*)

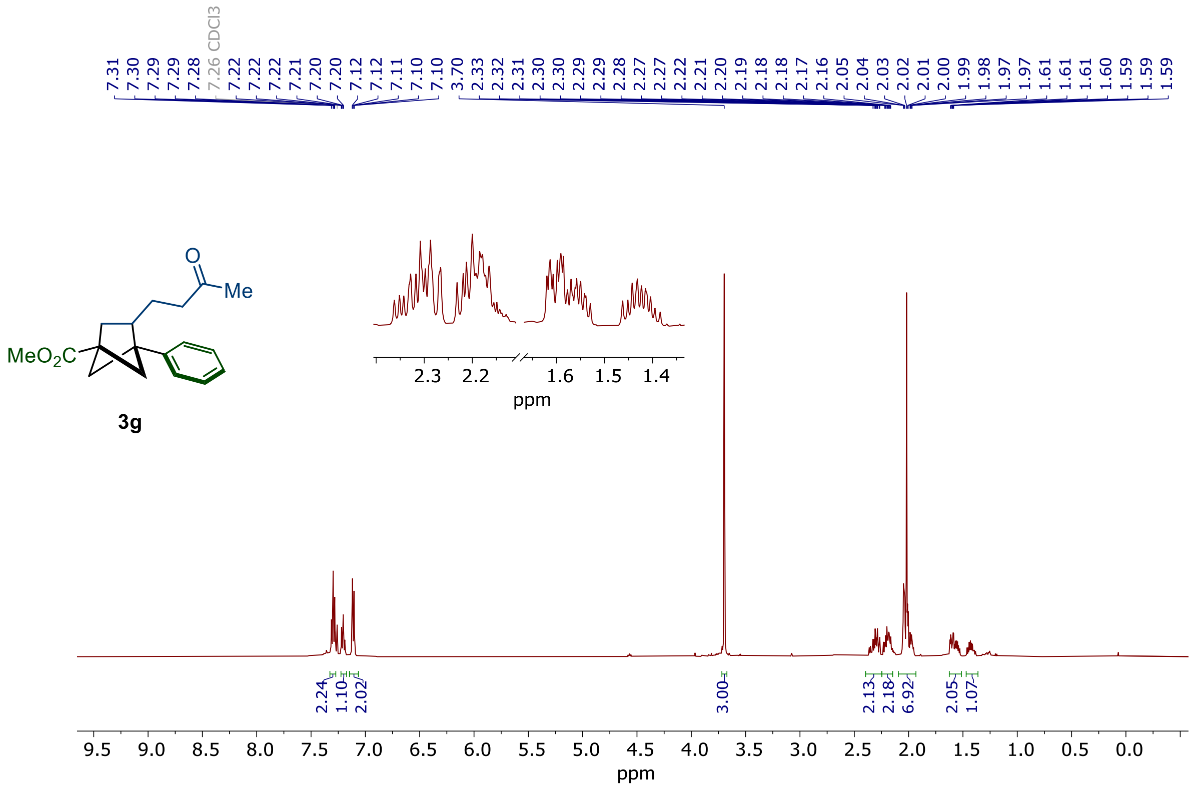

^{13}C NMR (126 MHz, $CDCl_3$) of **3g**

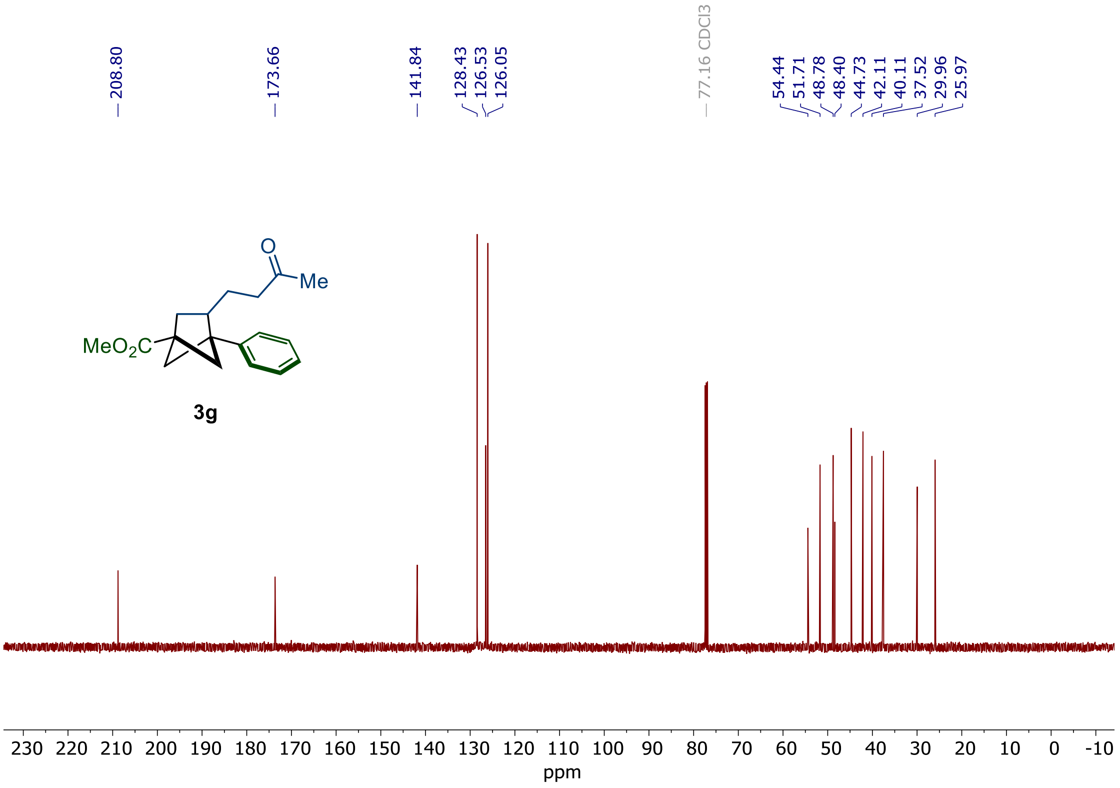

^{1}H NMR (400 MHz, $CDCl_3$) of **3h** (*see procedure*)

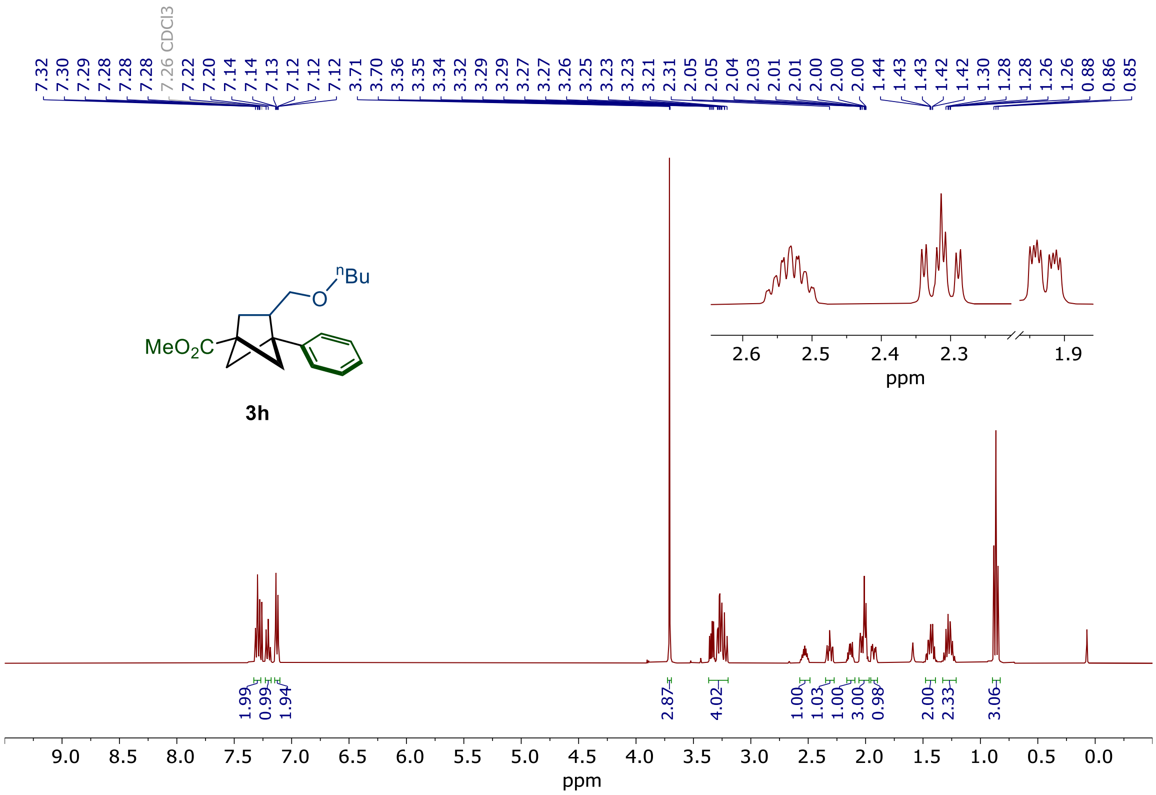

^{13}C NMR (101 MHz, $CDCl_3$) of **3h**

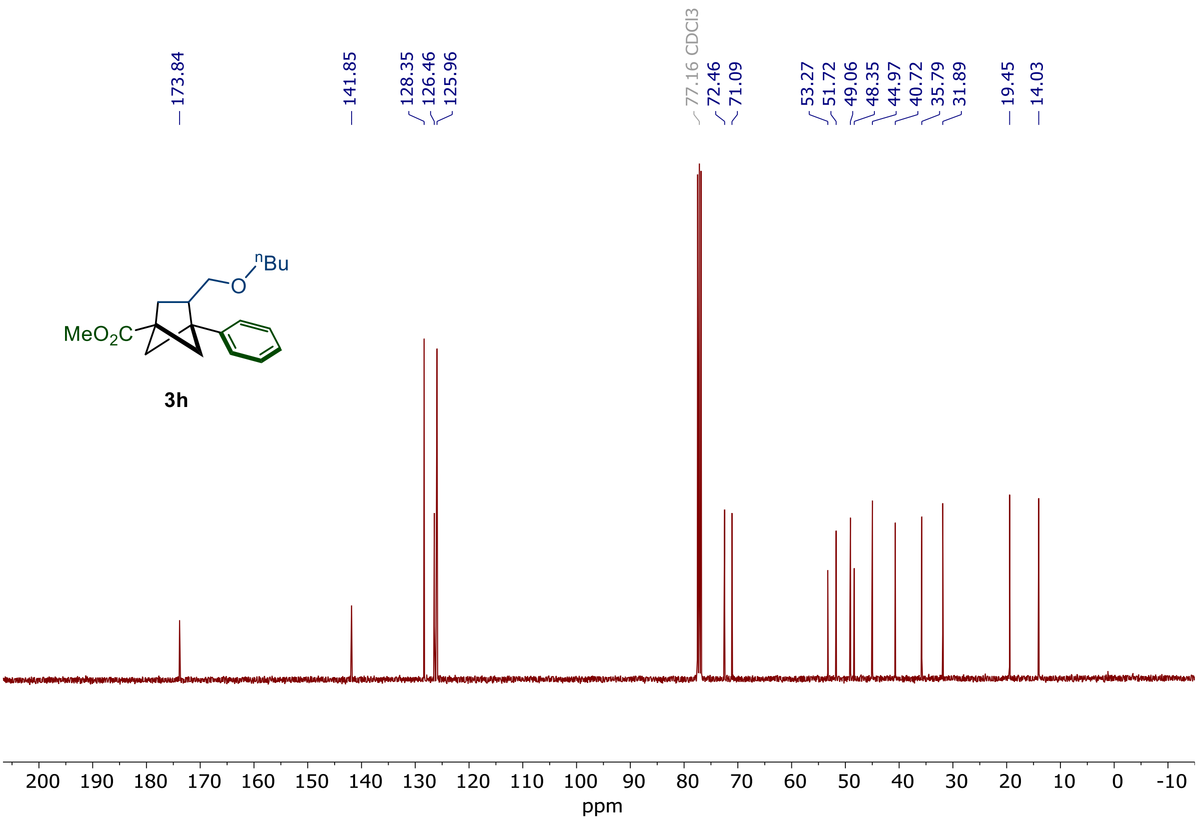

^{1}H NMR (400 MHz, $CDCl_3$) of **3i** (*see procedure*)

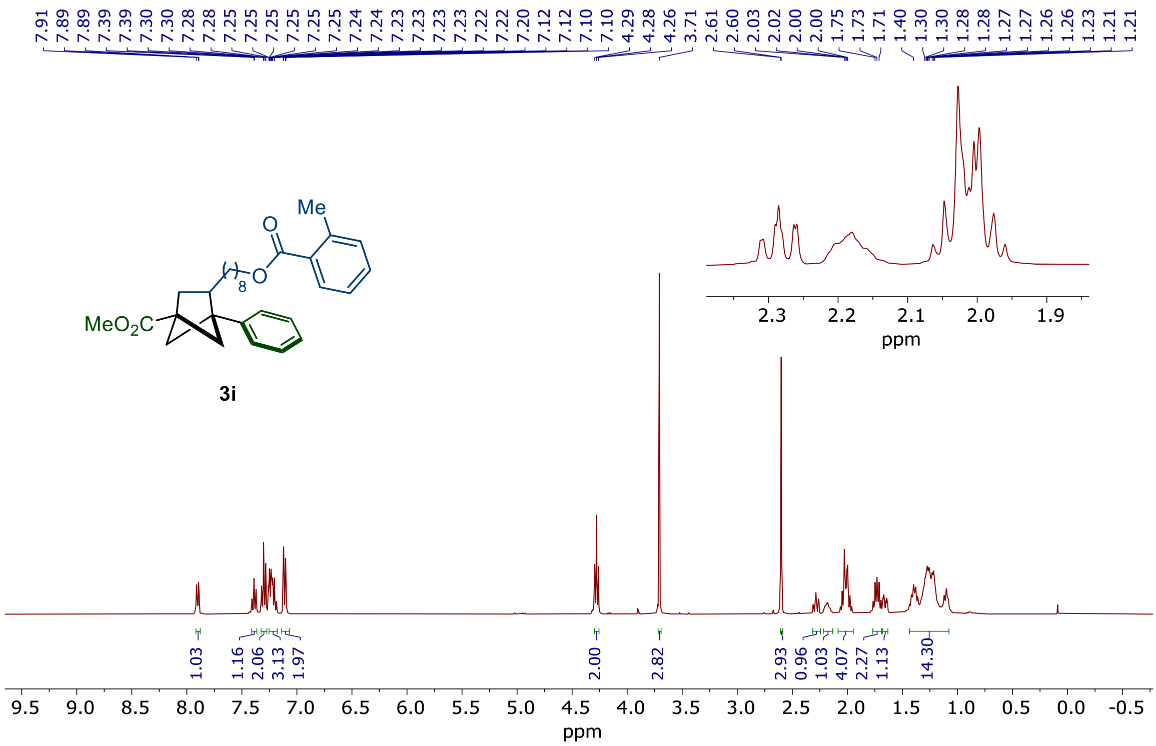

^{13}C NMR (101 MHz, $CDCl_3$) of **3i**

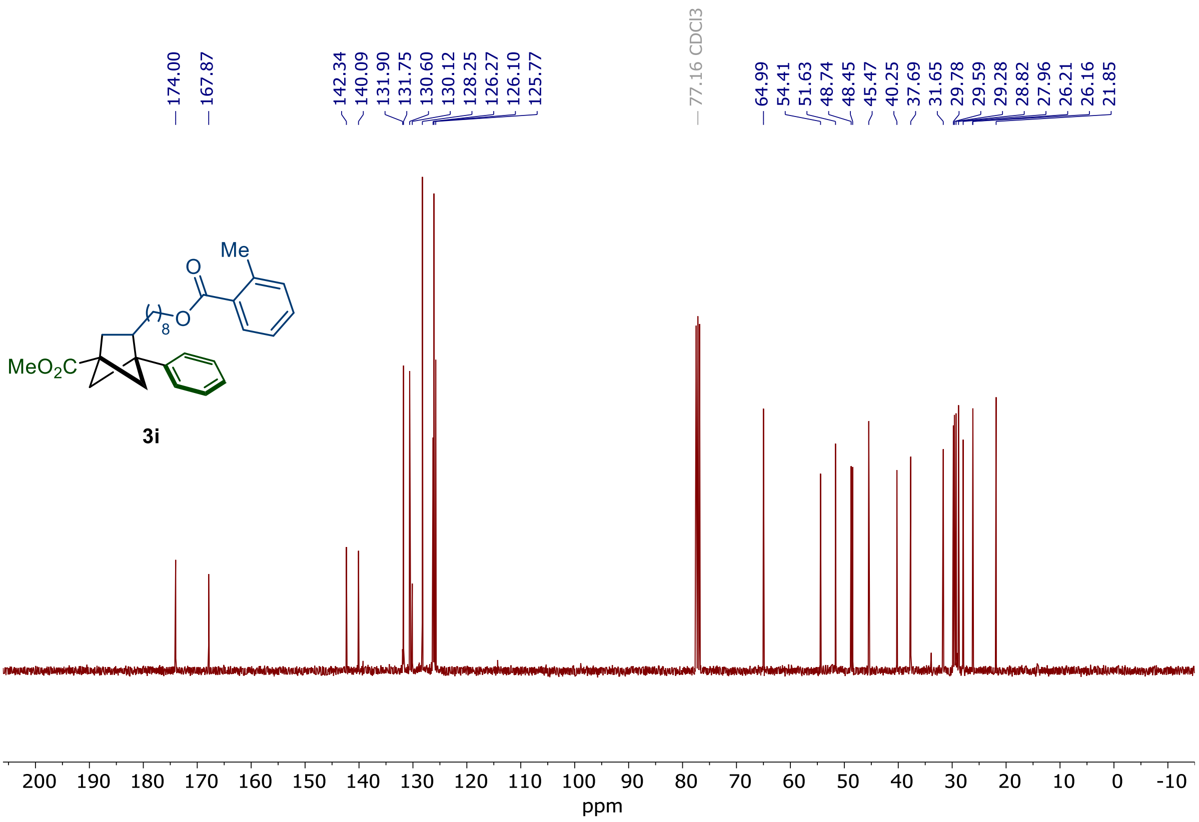

^{1}H NMR (600 MHz, $CDCl_3$) of **3j** (*see procedure*)

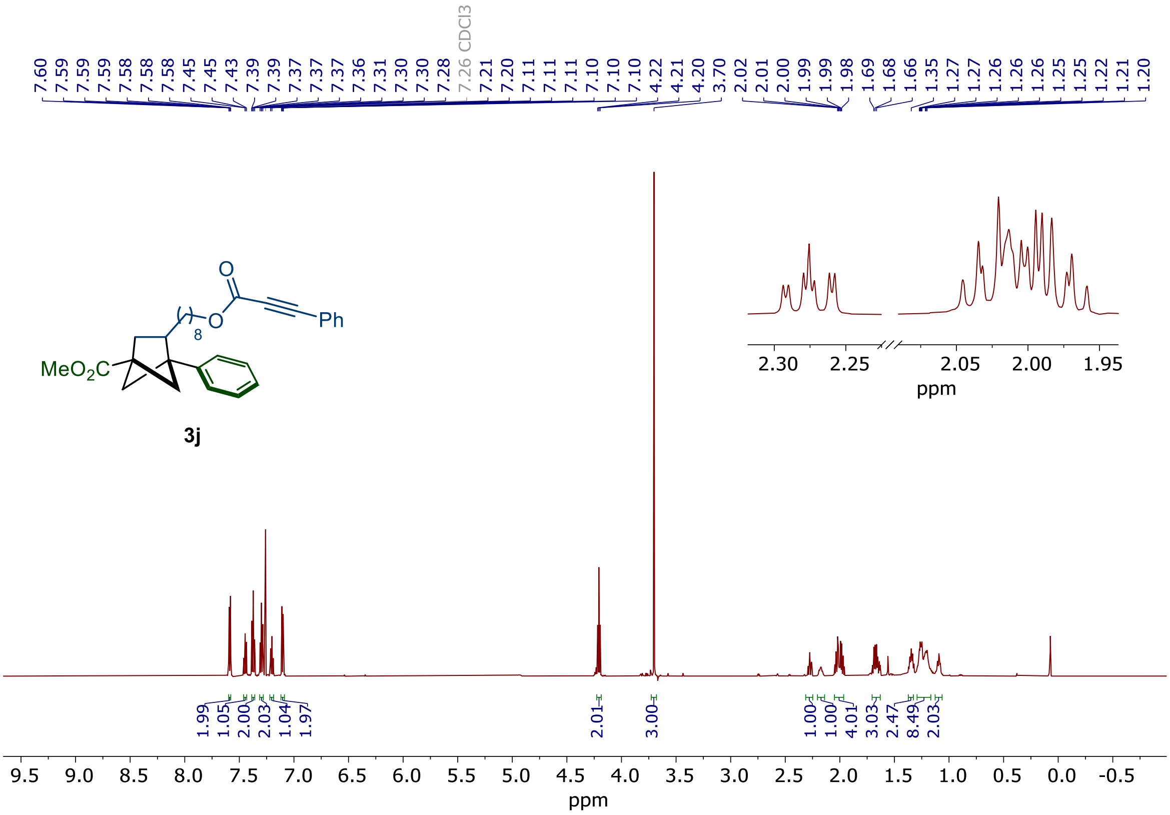

^{13}C NMR (151 MHz, $CDCl_3$) of **3j**

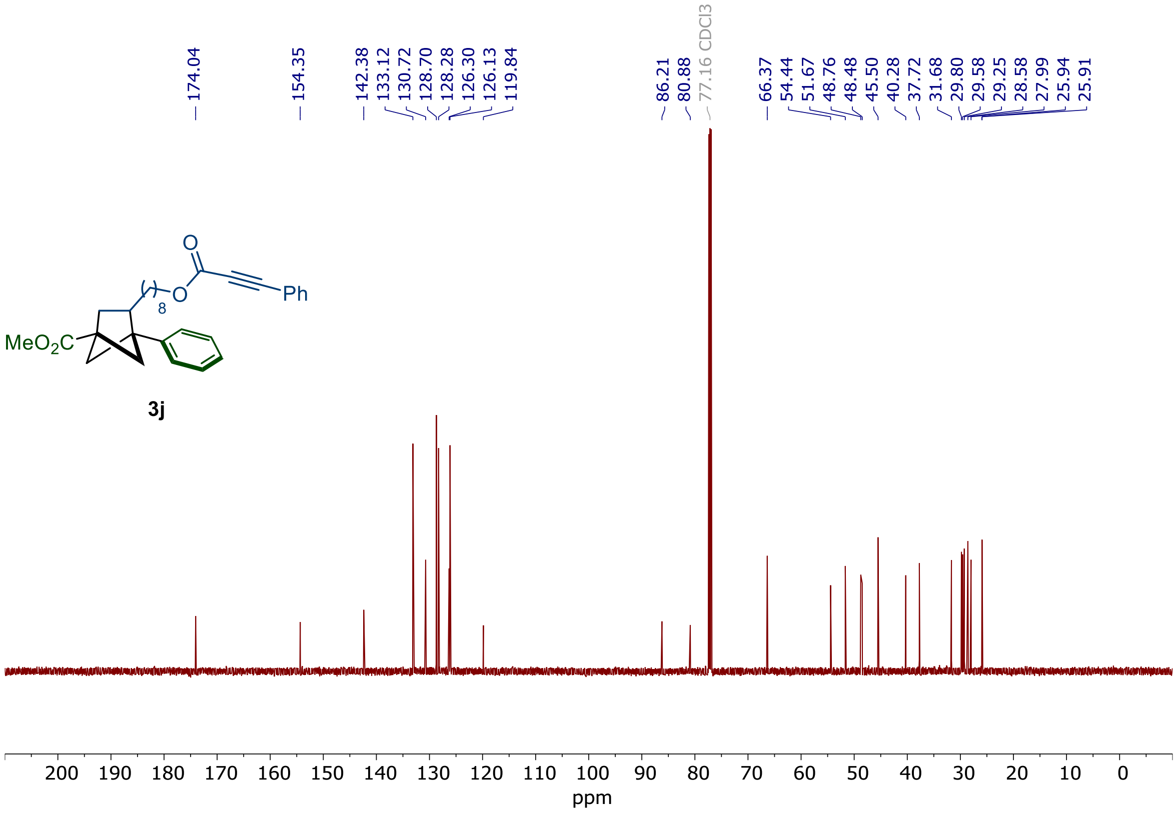

^{1}H NMR (400 MHz, $CDCl_3$) of **3k** (*see procedure*)

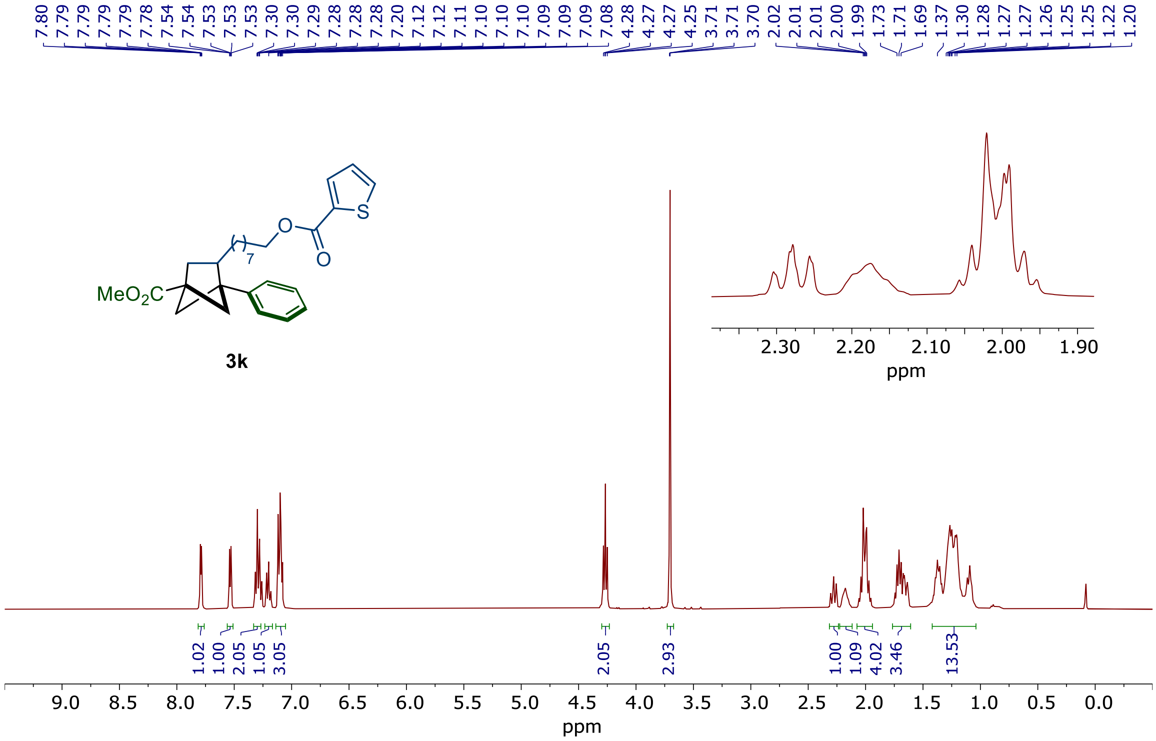

^{13}C NMR (101 MHz, $CDCl_3$) of **3k**

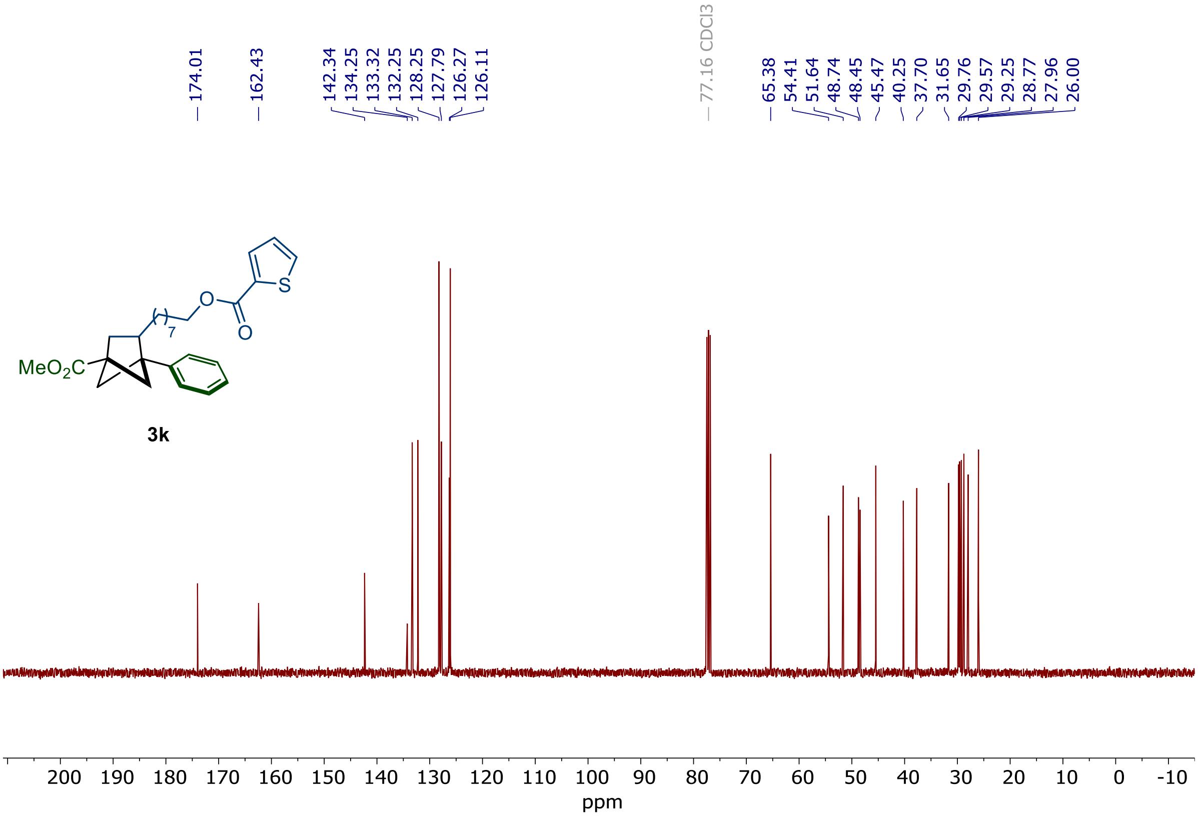

^{1}H NMR (500 MHz, $CDCl_3$) of **3l** (*see procedure*)

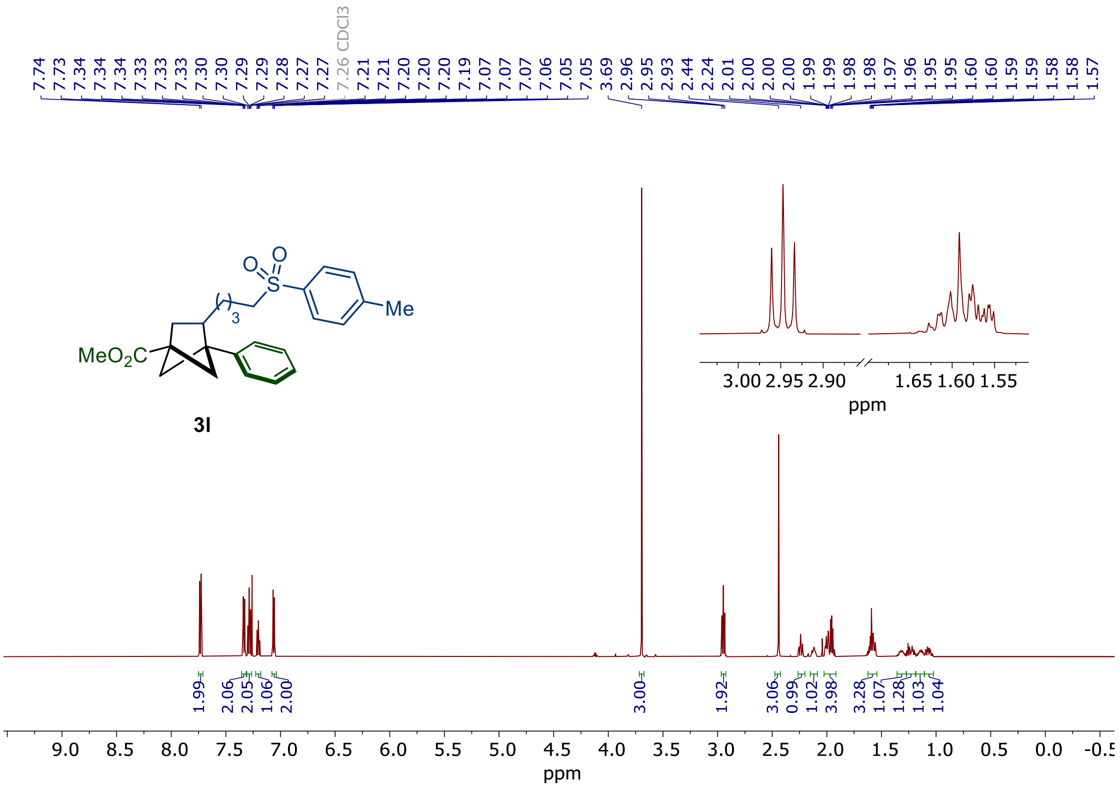

^{13}C NMR (126 MHz, $CDCl_3$) of **3l**

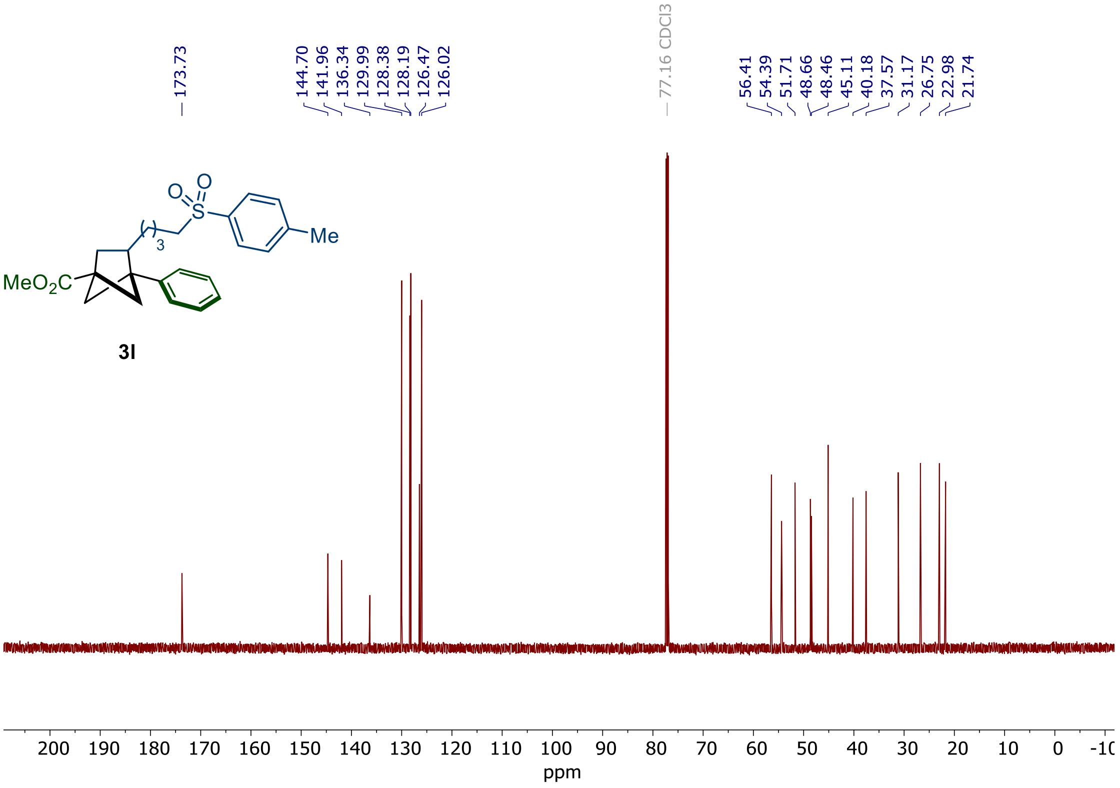

^{1}H NMR (500 MHz, $CDCl_3$) of **3m** (see procedure)

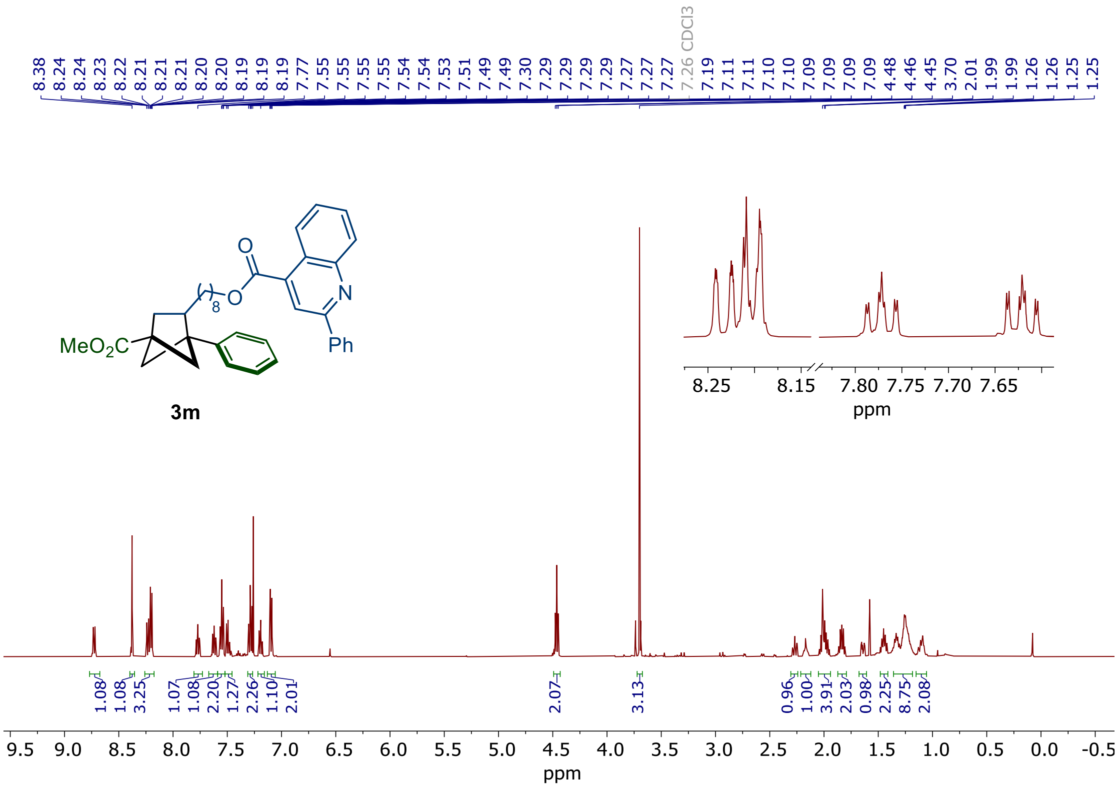

^{13}C NMR (126 MHz, $CDCl_3$) of **3m**

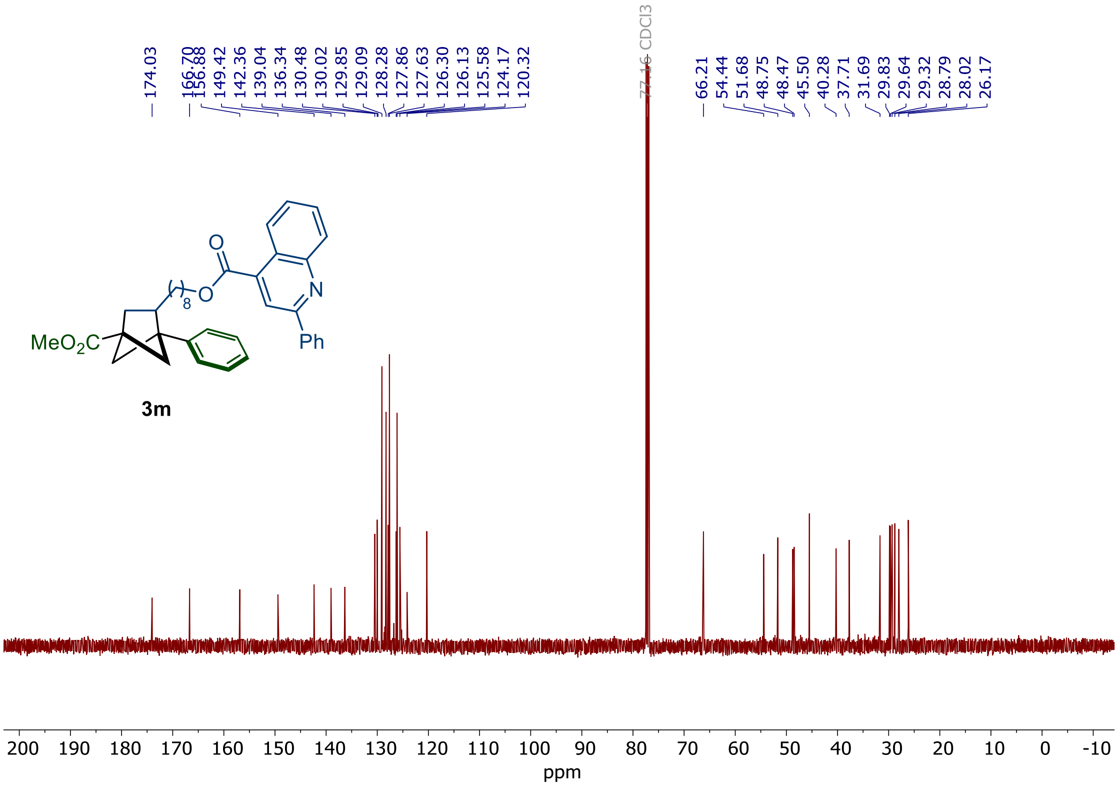

^{1}H NMR (400 MHz, $CDCl_3$) of **3n** (*see procedure*)

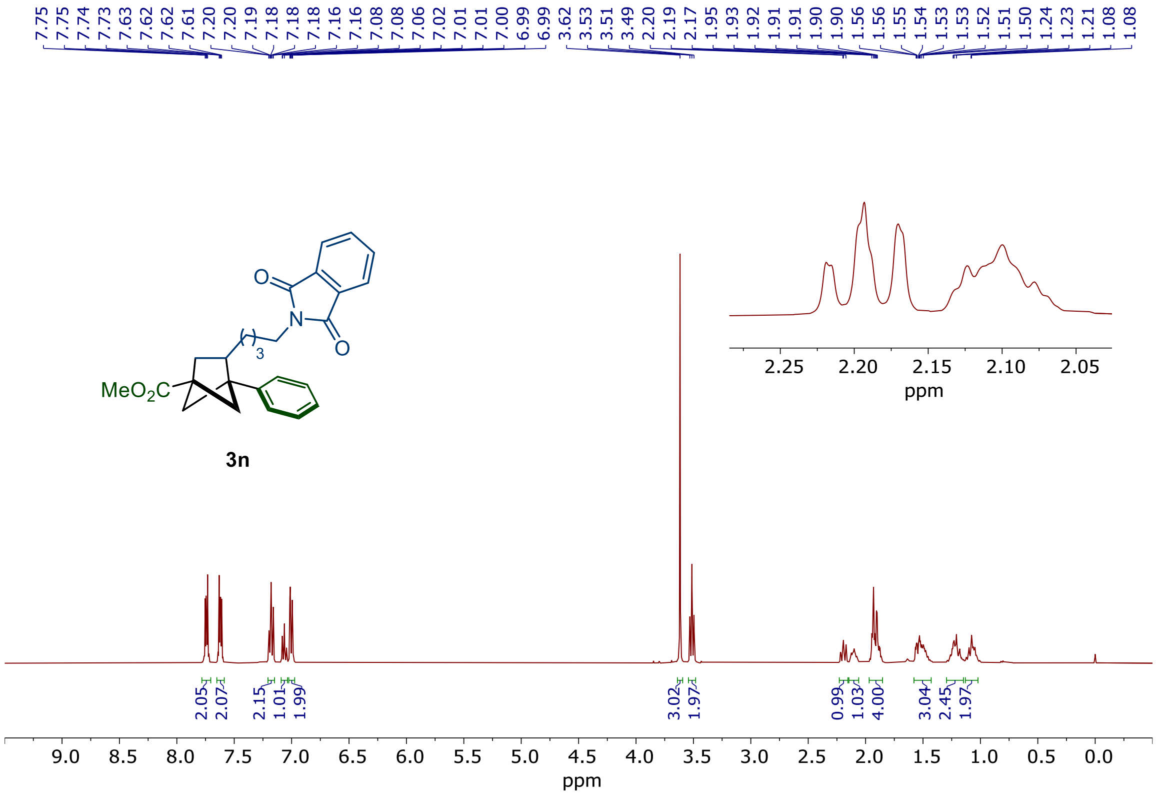

^{13}C NMR (101 MHz, $CDCl_3$) of **3n**

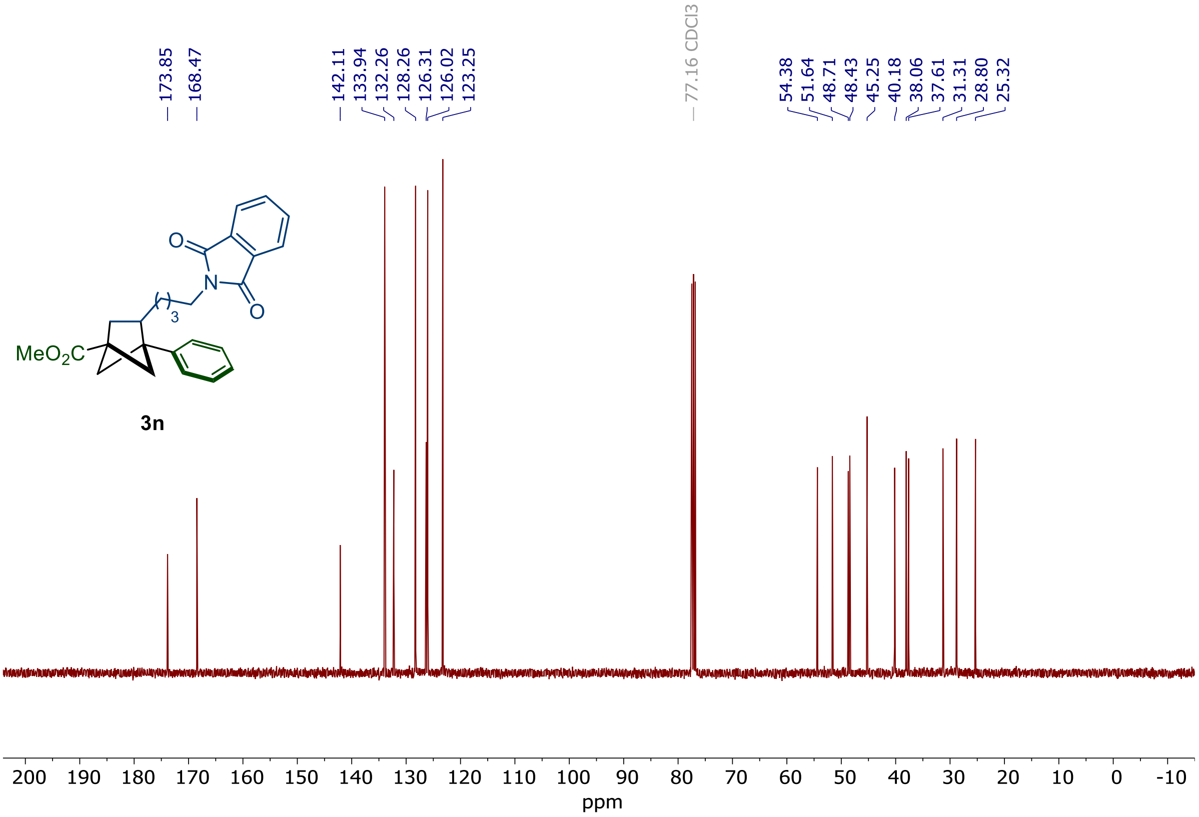

^{1}H NMR (400 MHz, $CDCl_3$) of **3o** (*see procedure*)

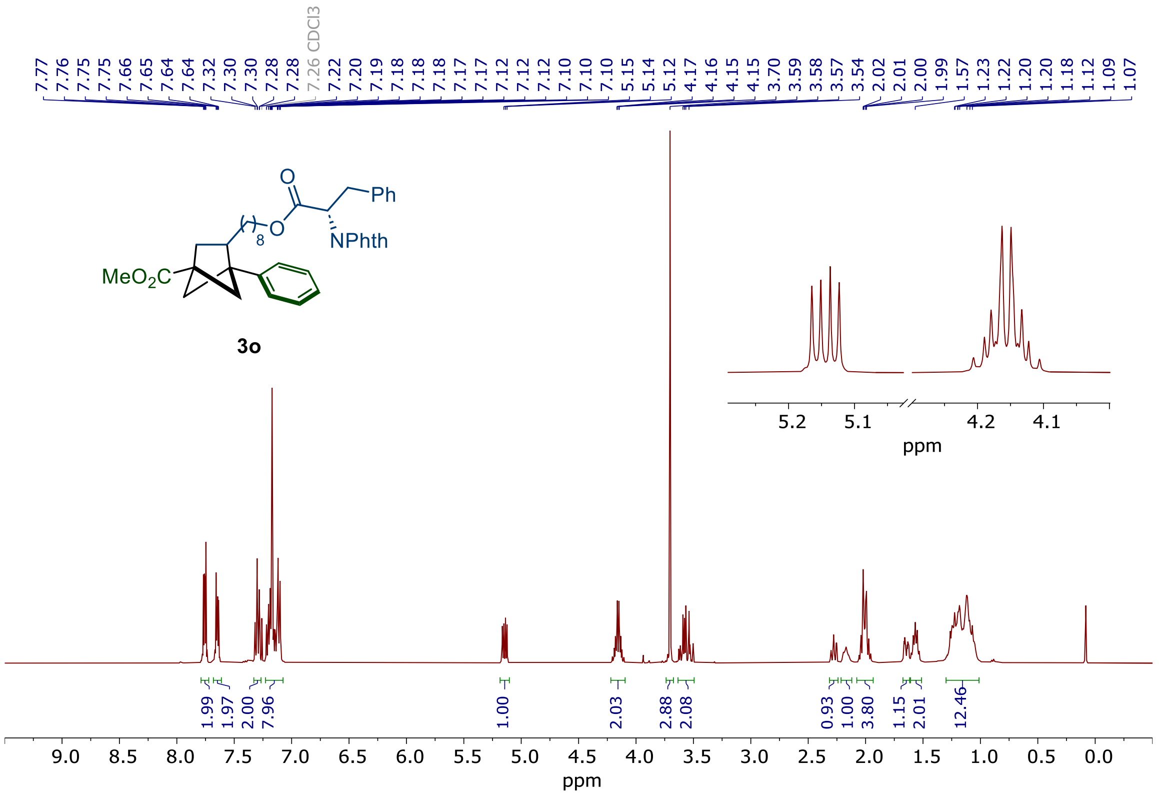

^{13}C NMR (101 MHz, $CDCl_3$) of **3o**

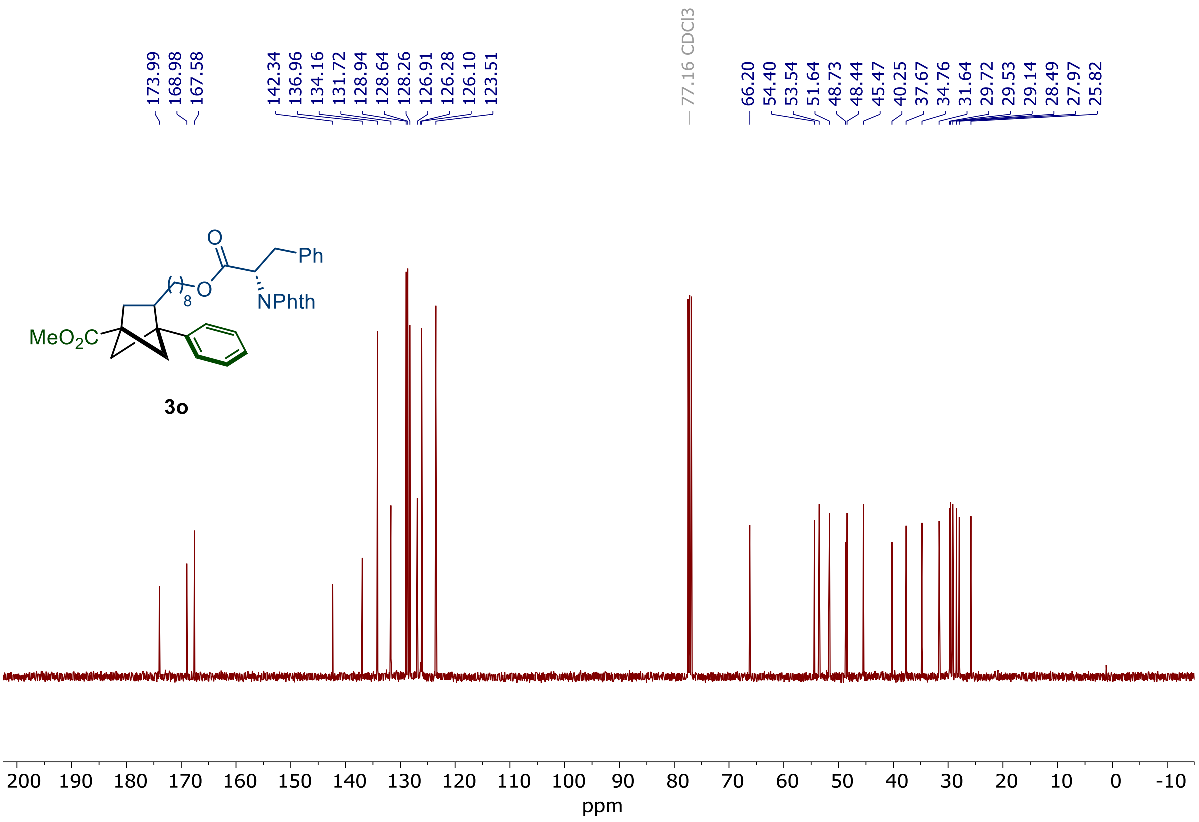

^{1}H NMR (400 MHz, $CDCl_3$) of **3p** (*see procedure*)

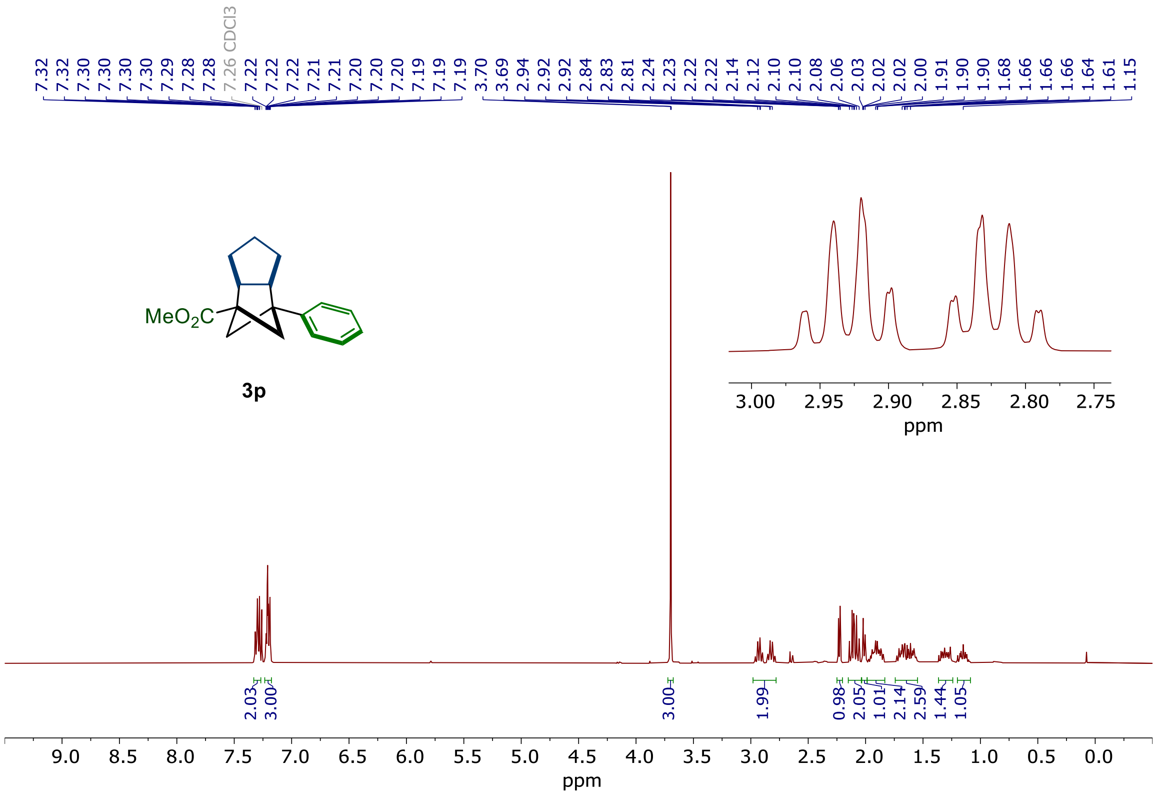

^{13}C NMR (101 MHz, $CDCl_3$) of **3p**

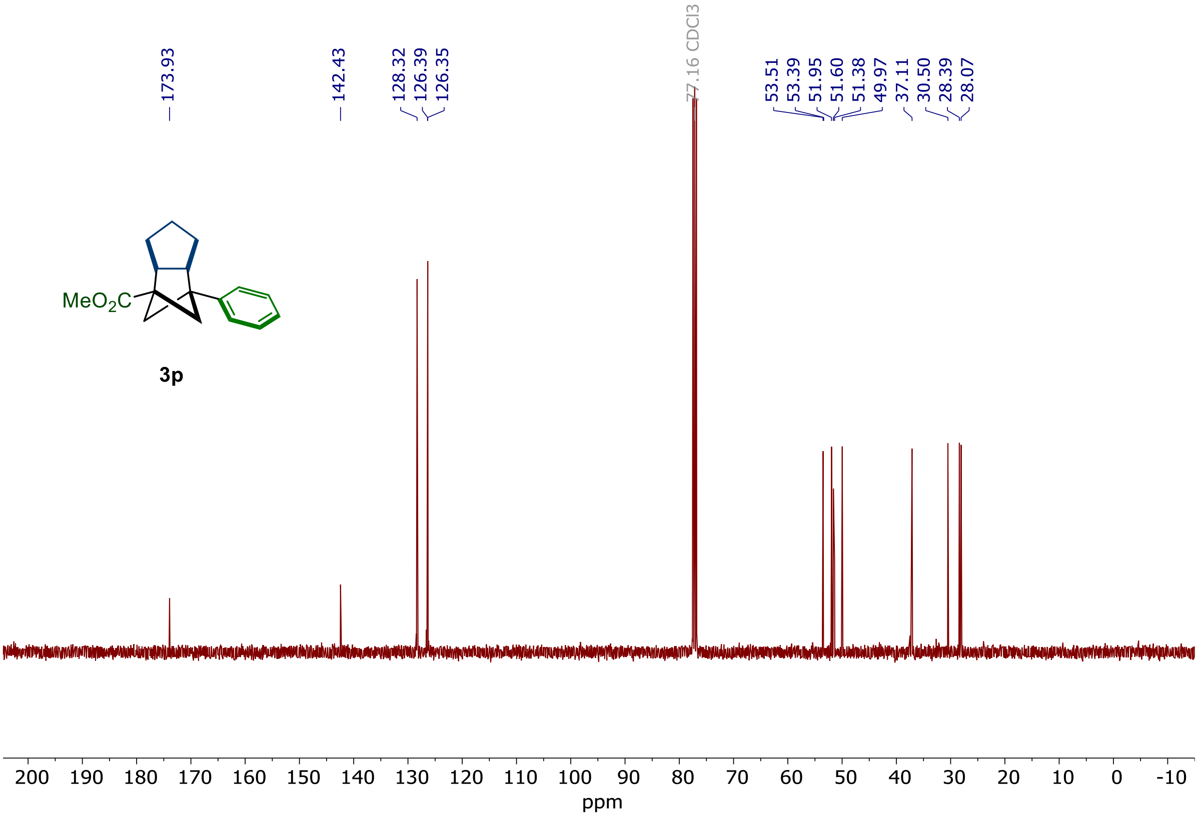

^{1}H NMR (400 MHz, $CDCl_3$) of **3q** (*see procedure*)

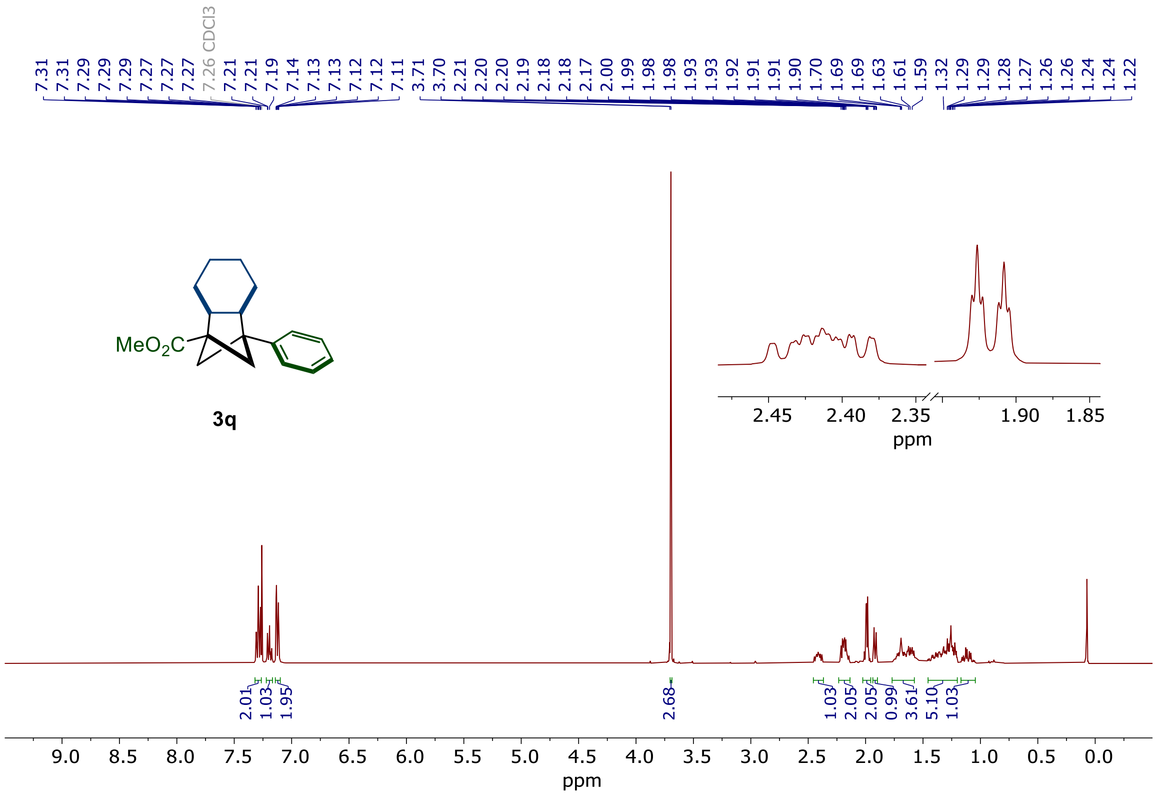

^{13}C NMR (101 MHz, $CDCl_3$) of **3q**

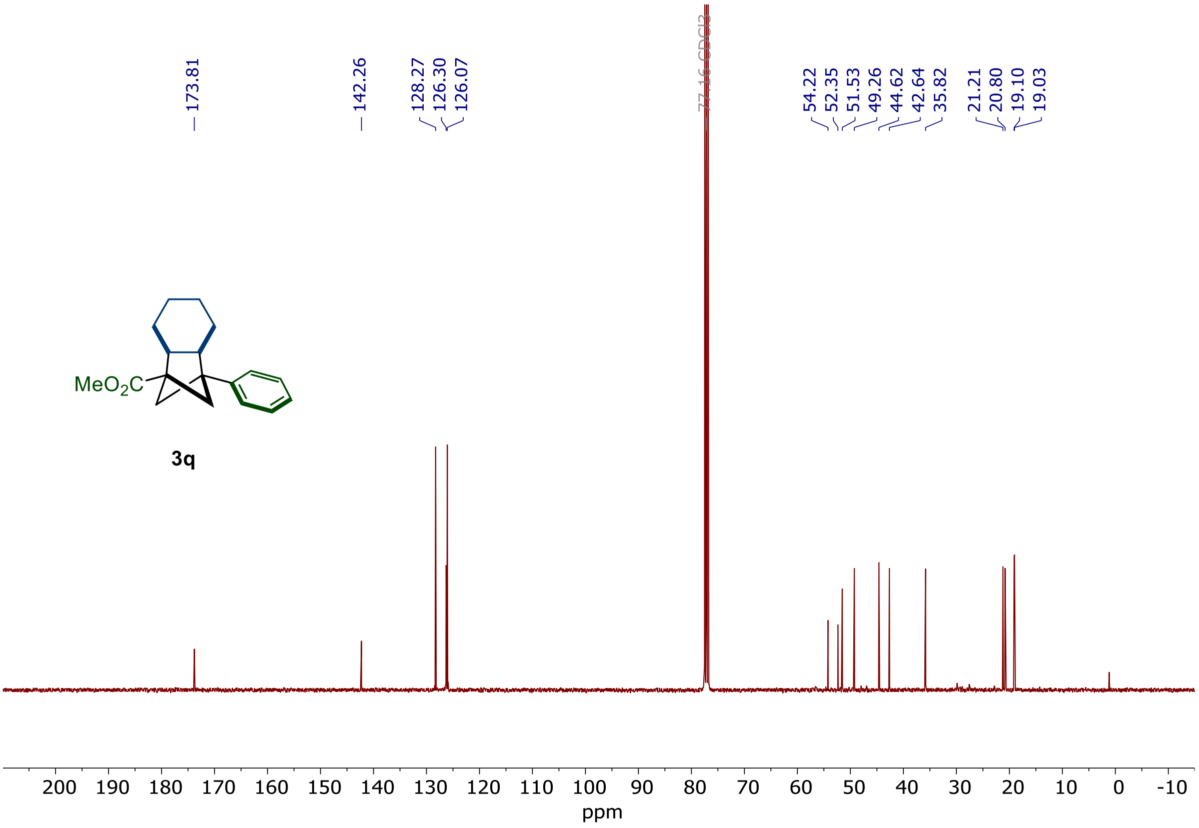

^{1}H NMR (400 MHz, $CDCl_3$) of **3r** (*see procedure*)

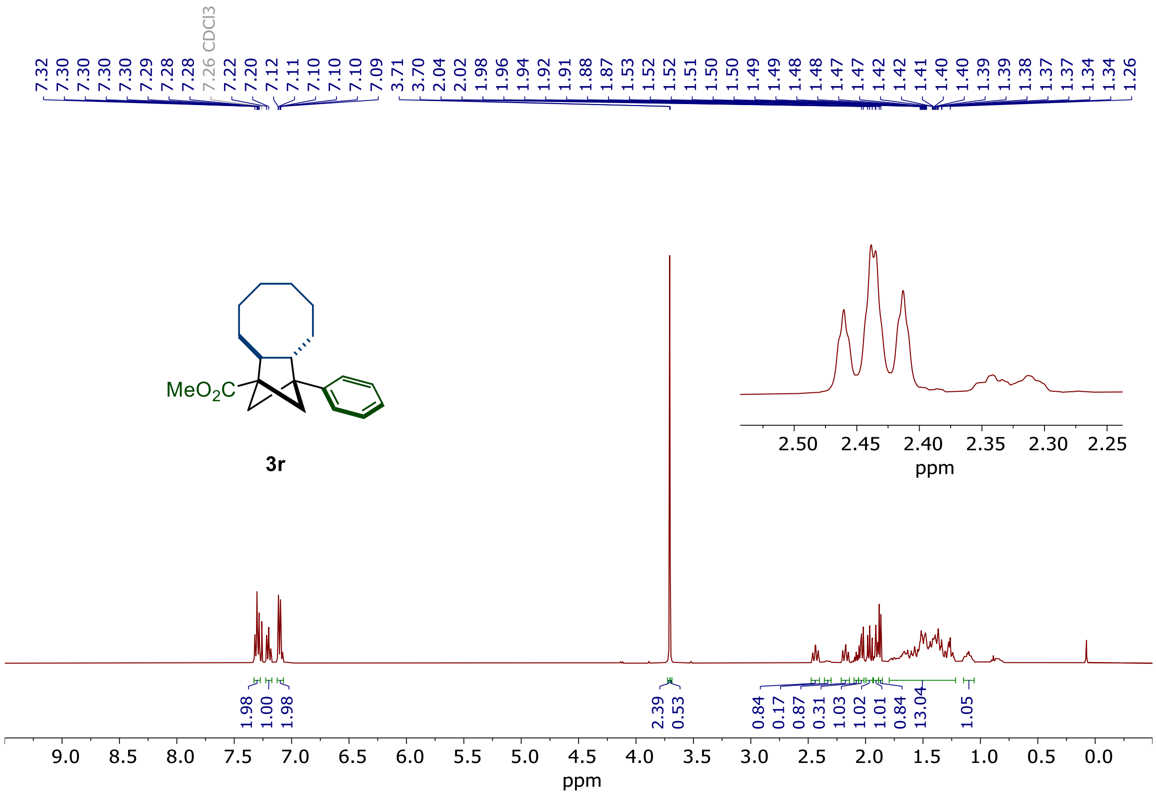

^{13}C NMR (101 MHz, $CDCl_3$) of **3r**

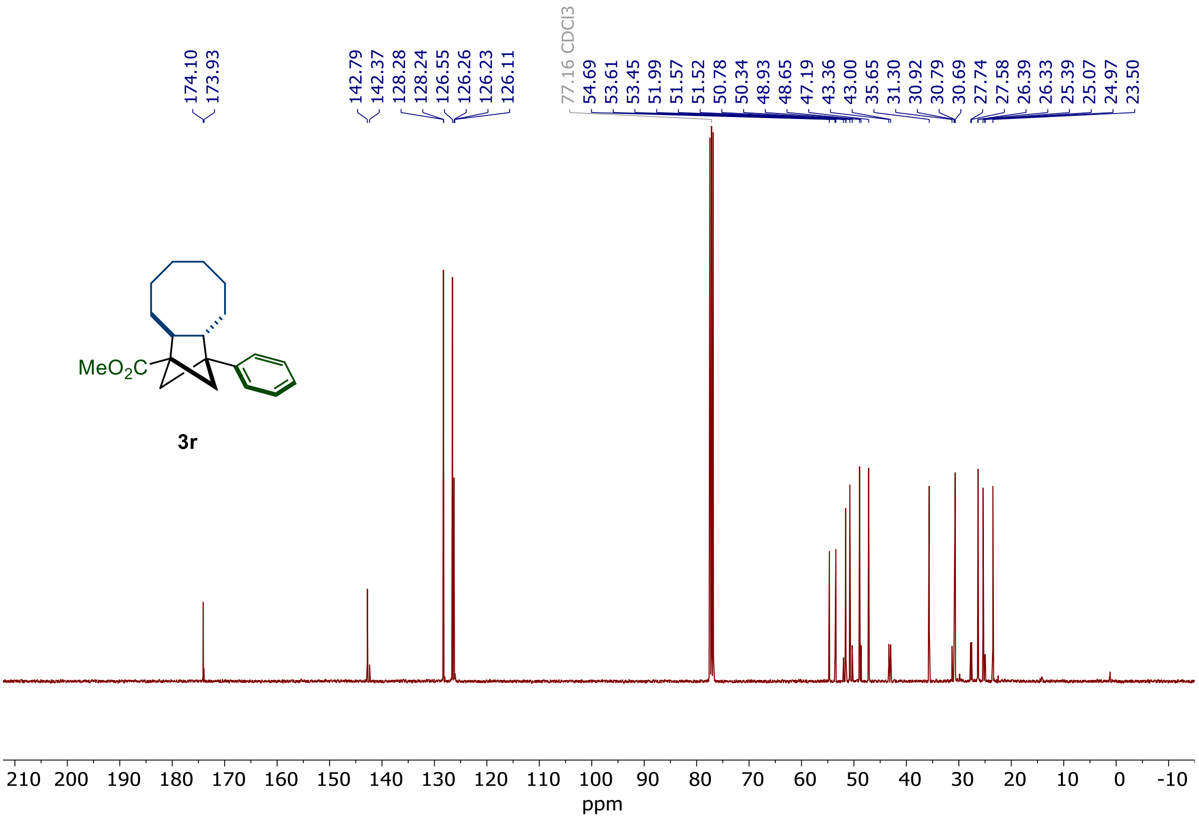

^{1}H NMR (400 MHz, $CDCl_3$) of **3s** (*see procedure*)

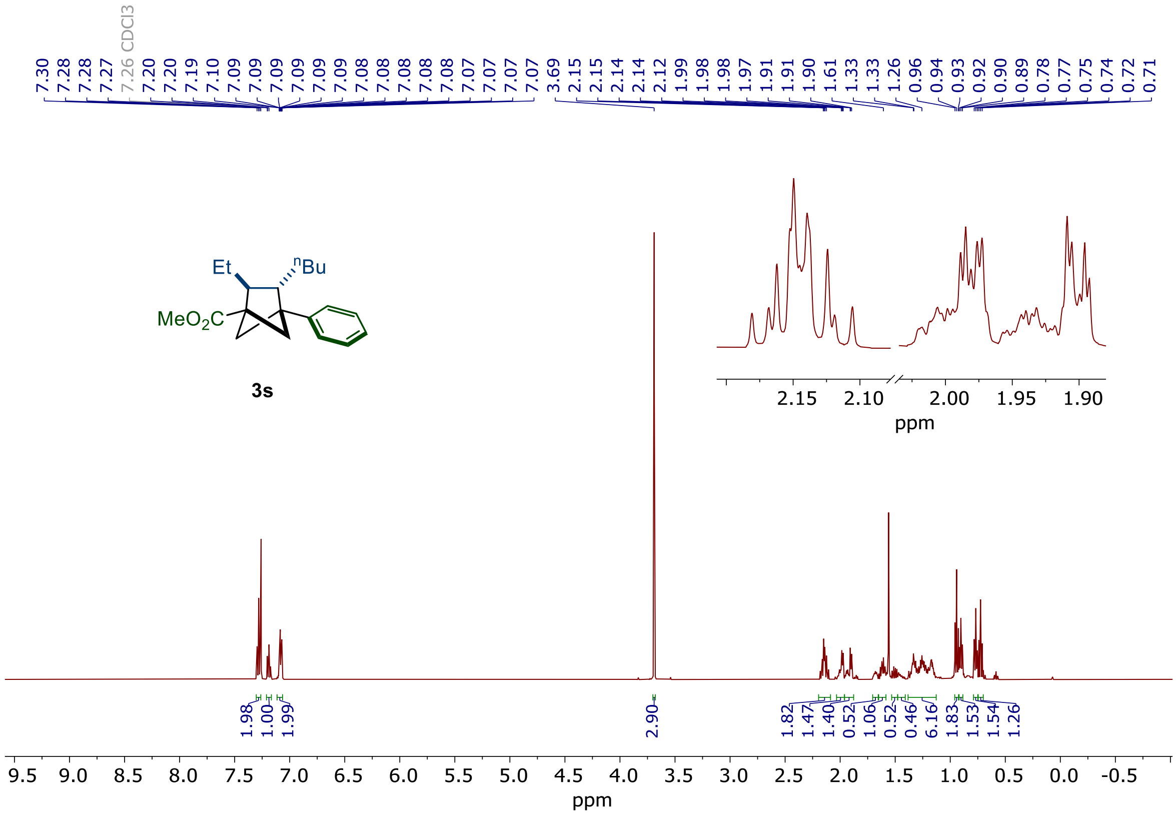

^{13}C NMR (101 MHz, $CDCl_3$) of **3s**

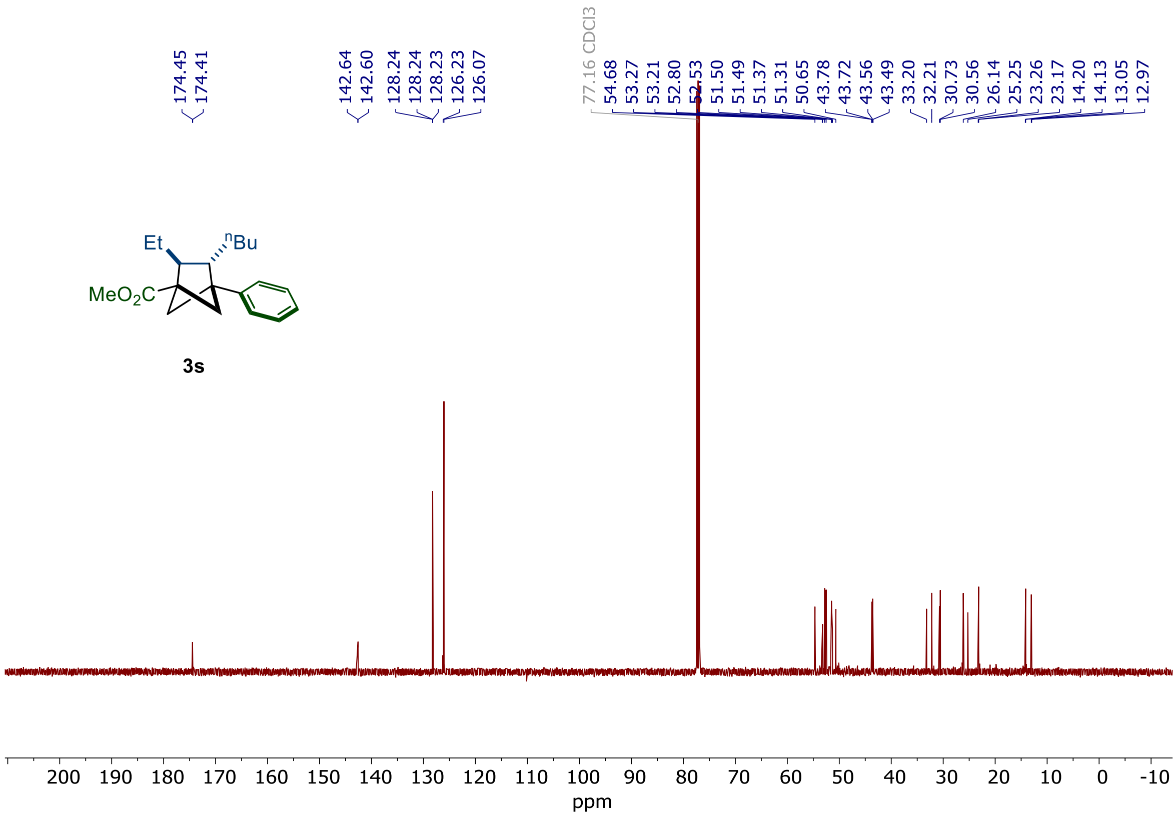

^{1}H NMR (500 MHz, $CDCl_3$) of **3t** (*see procedure*)

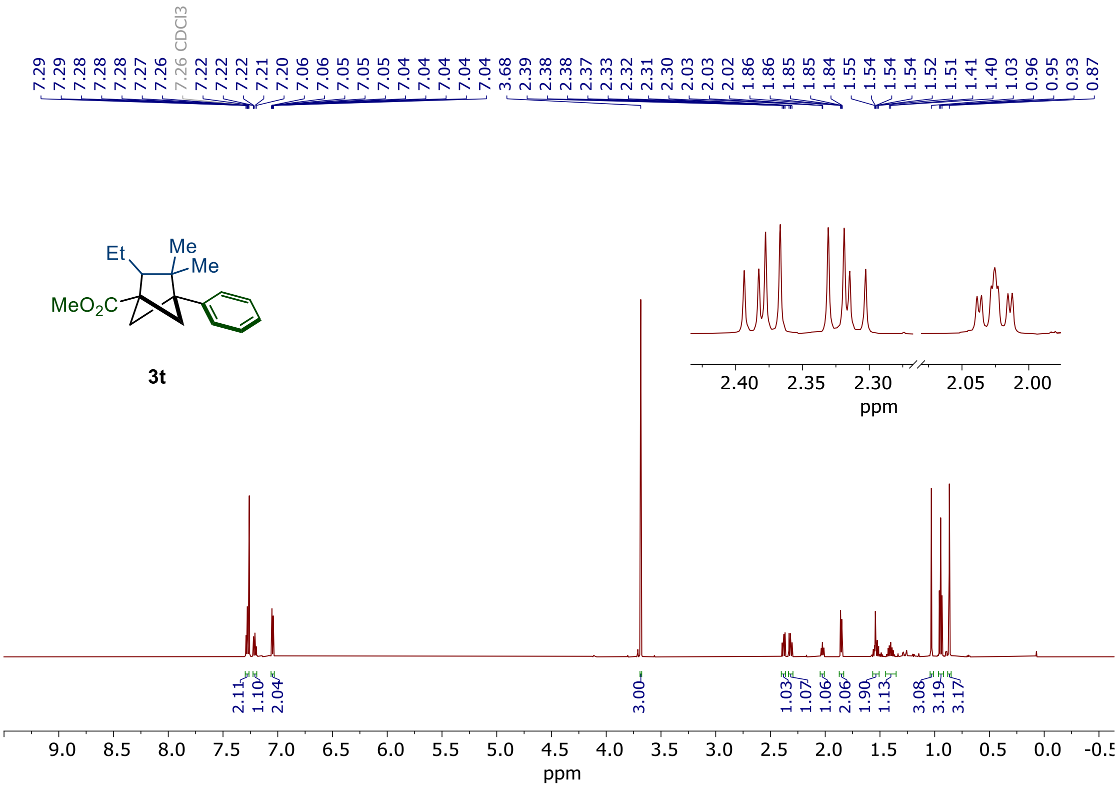

^{13}C NMR (126 MHz, $CDCl_3$) of **3t**

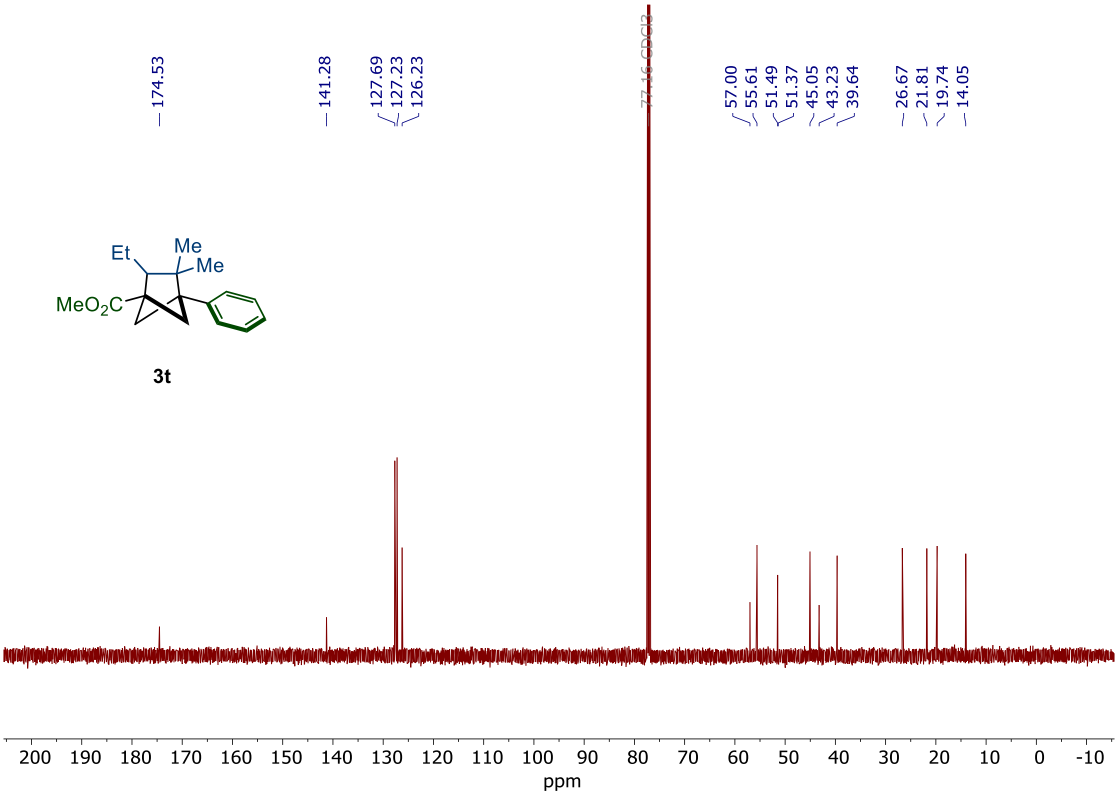

^{1}H NMR (500 MHz, $CDCl_3$) of **3u** (*see procedure*)

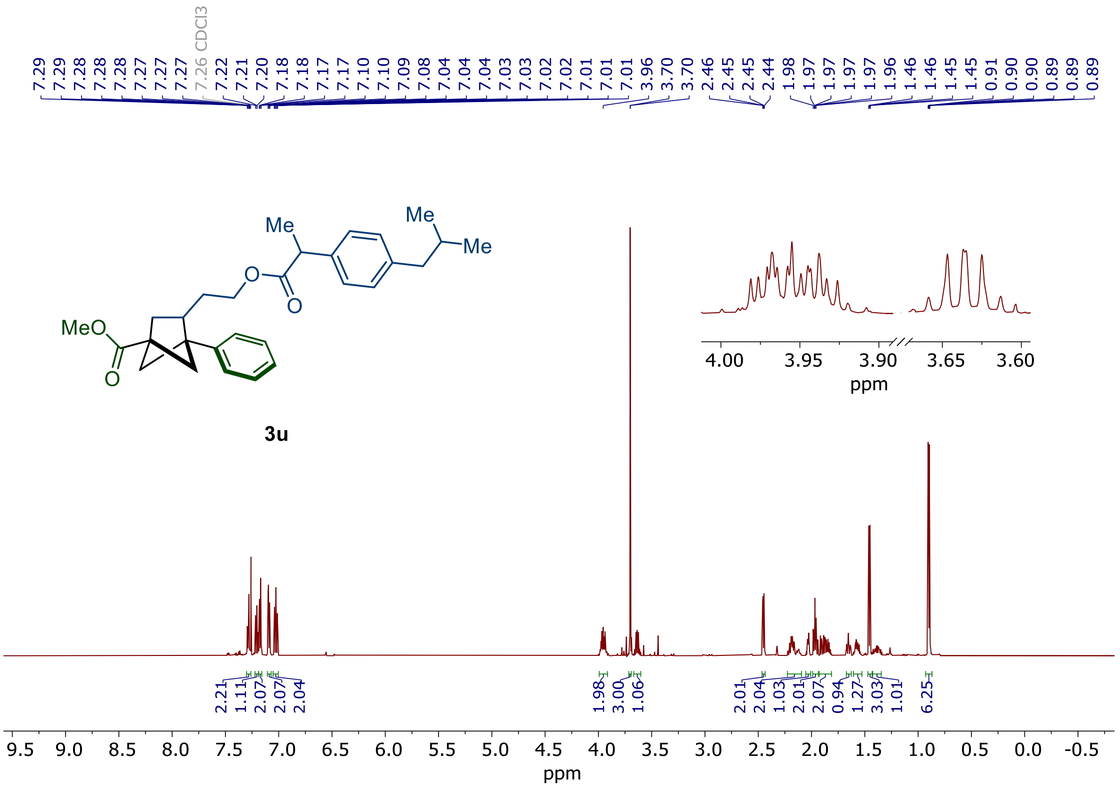

^{13}C NMR (126 MHz, $CDCl_3$) of **3u**

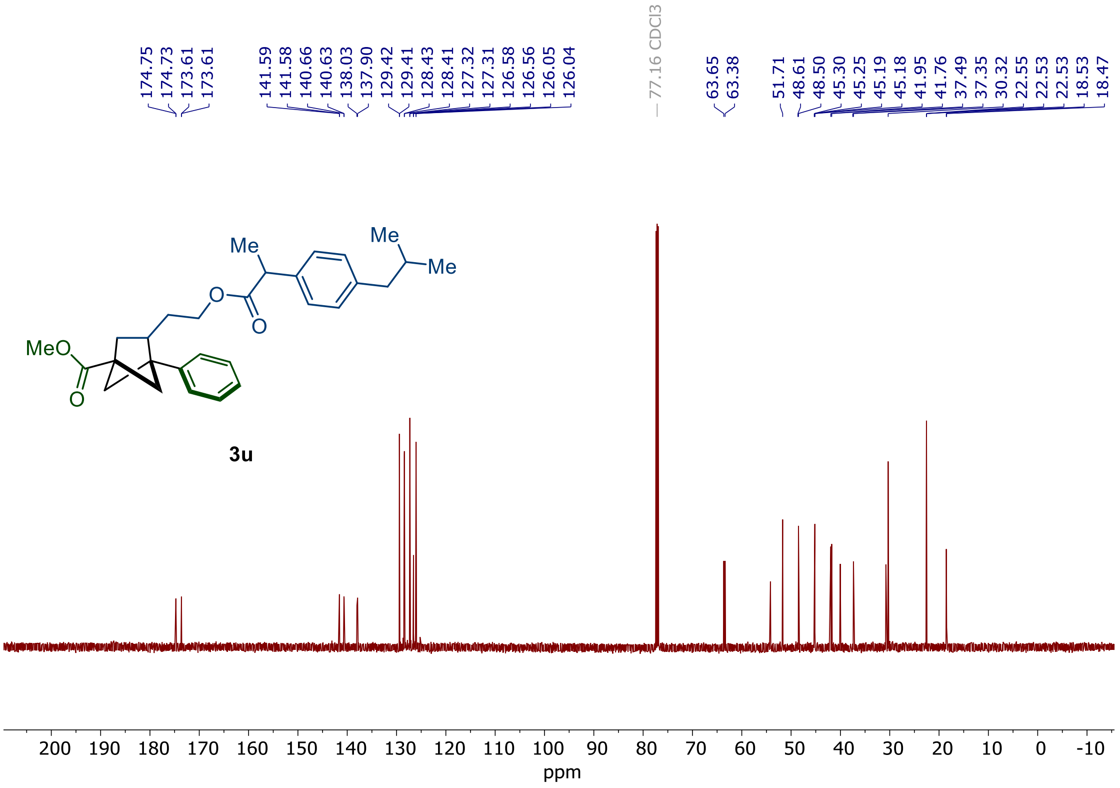

^{1}H NMR (500 MHz, $CDCl_3$) of **3v** (*see procedure*)

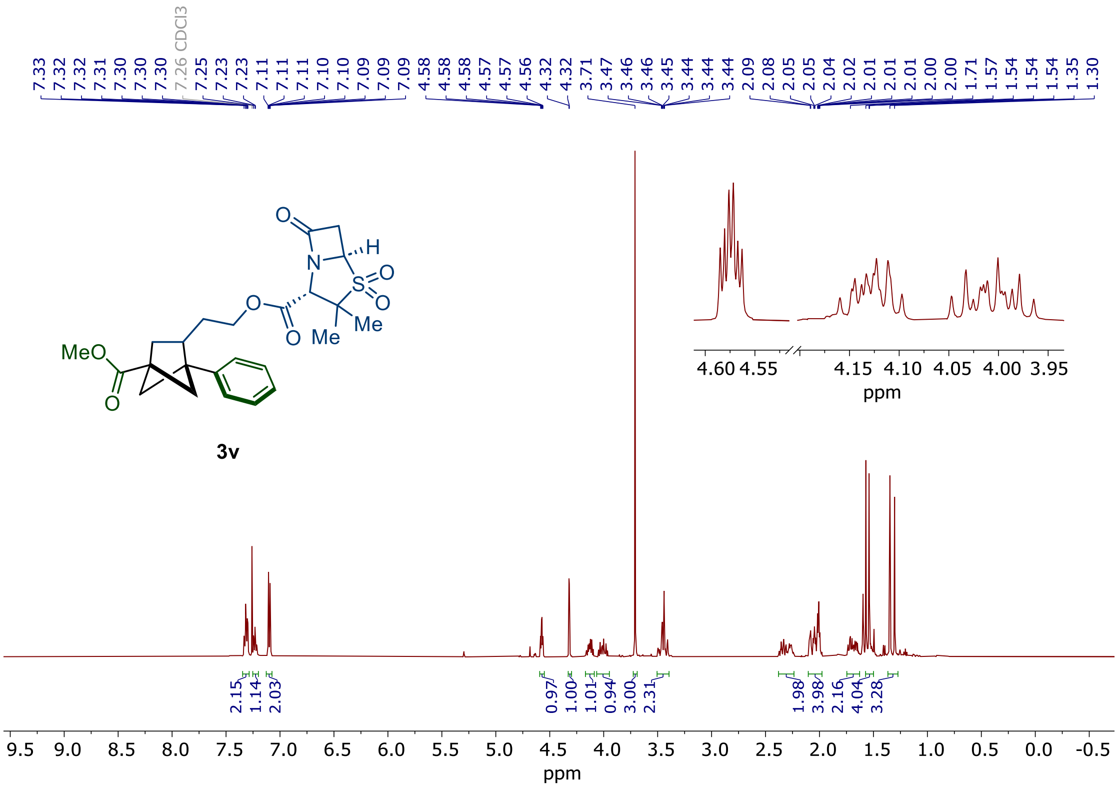

^{13}C NMR (126 MHz, $CDCl_3$) of **3v**

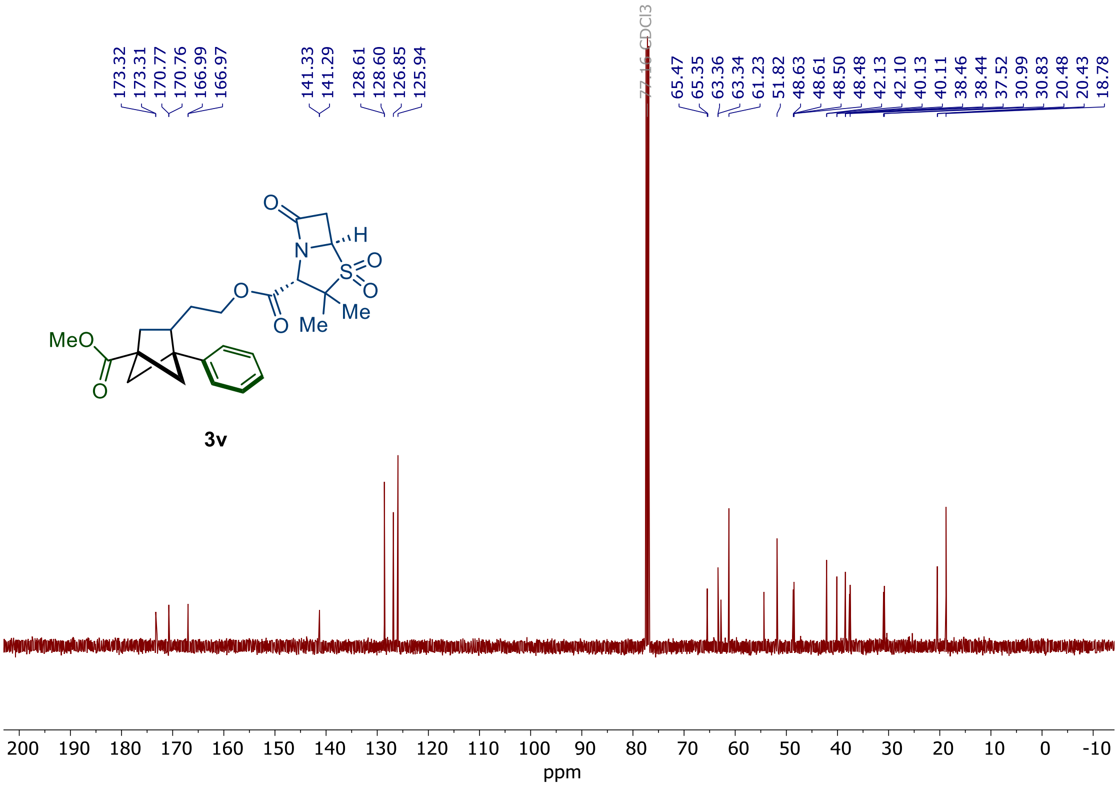

^{1}H NMR (500 MHz, $CDCl_3$) of **3w** (*see procedure*)

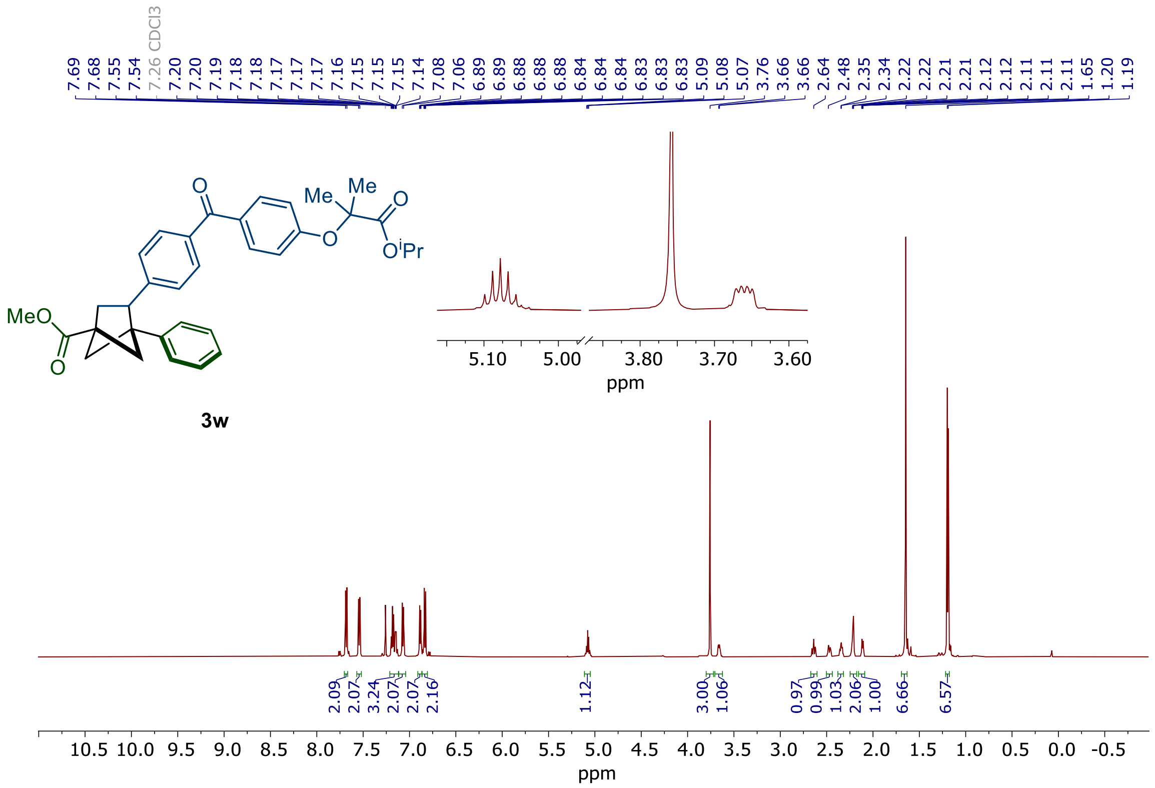

^{13}C NMR (126 MHz, $CDCl_3$) of **3w**

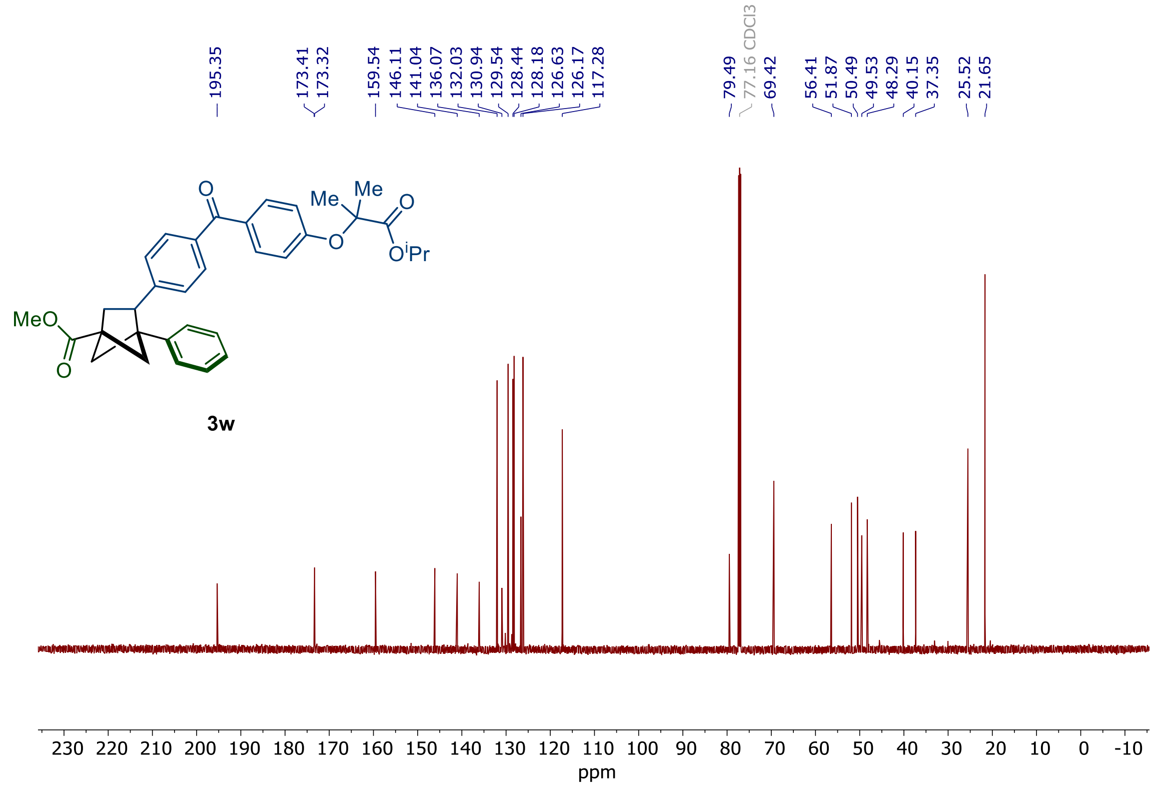

^{1}H NMR (500 MHz, $CDCl_3$) of **3x** (*see procedure*)

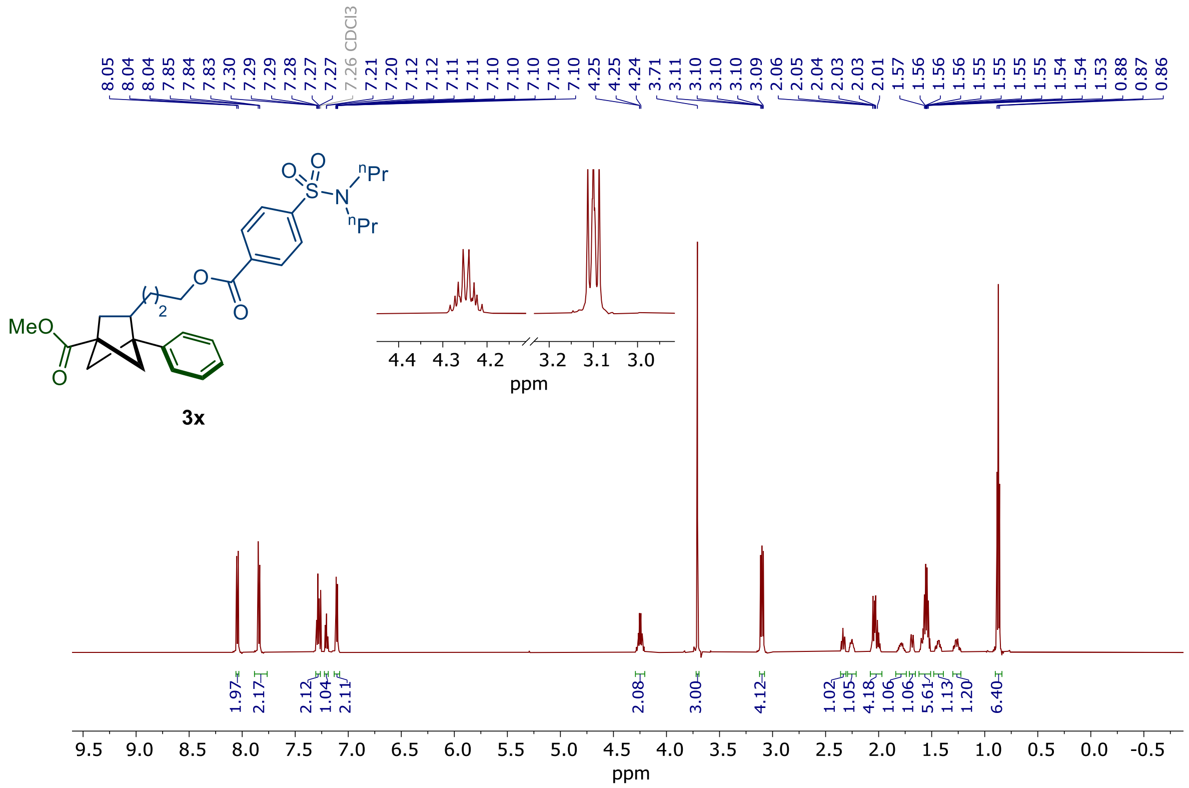

^{13}C NMR (126 MHz, $CDCl_3$) of **3x**

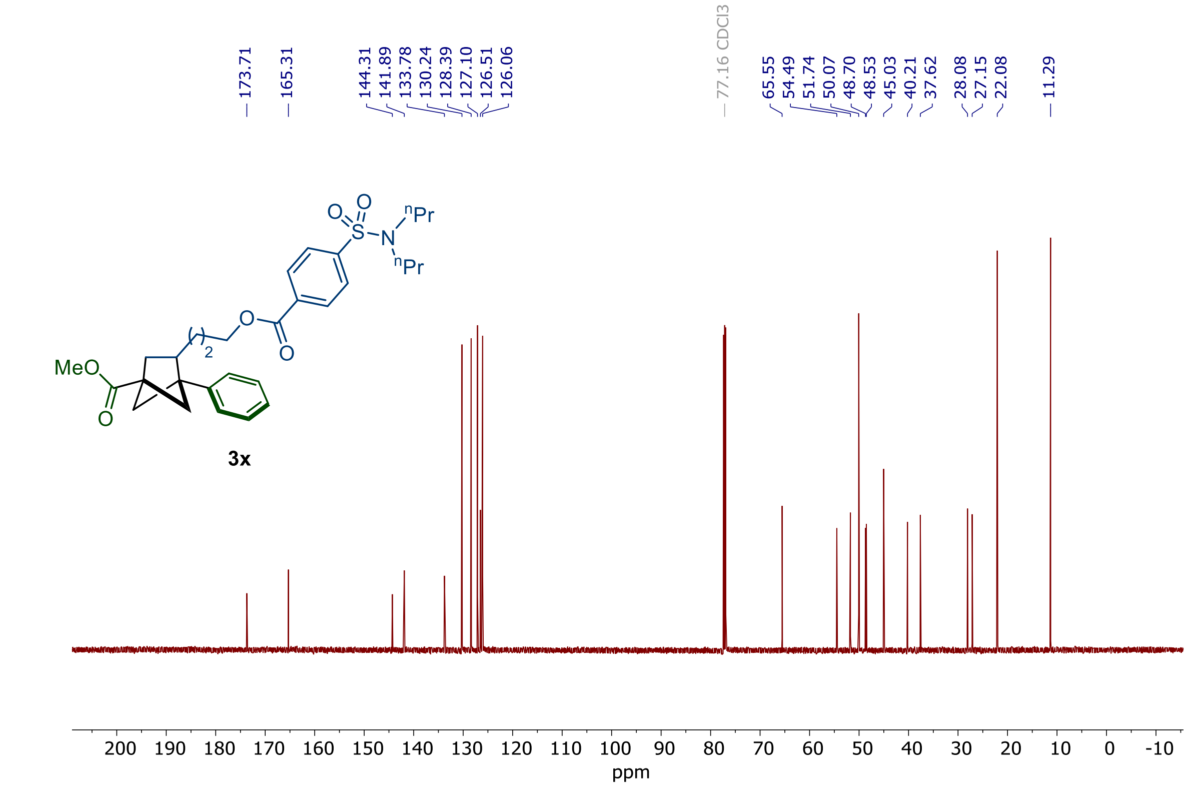

^{1}H NMR (500 MHz, $CDCl_3$) of **3y** (*see procedure*)

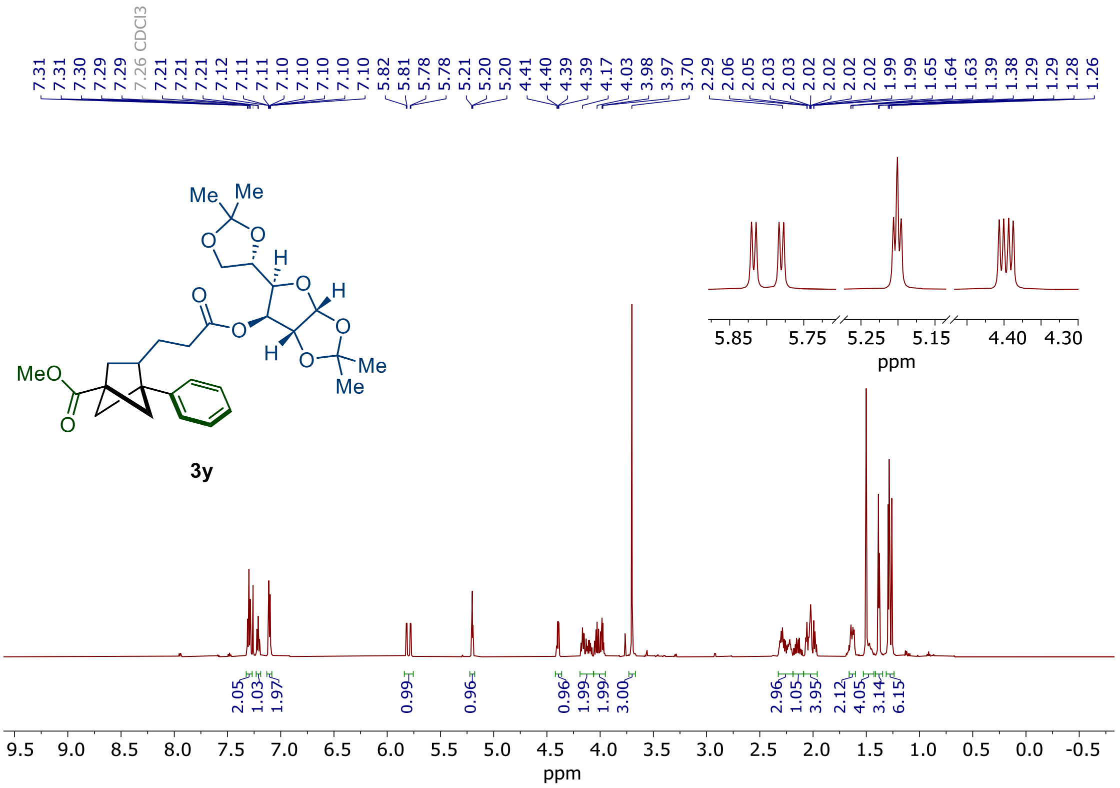

^{13}C NMR (126 MHz, $CDCl_3$) of **3y**

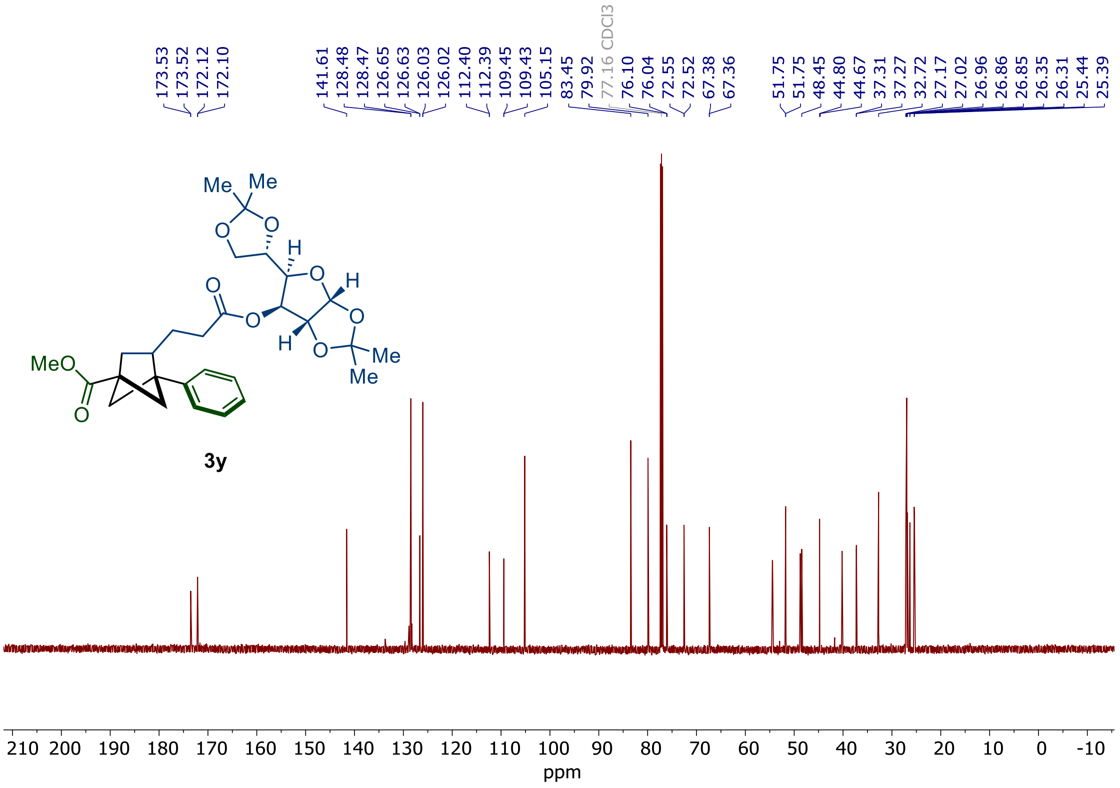

^{1}H NMR (500 MHz, $CDCl_3$) of **3z** (*see procedure*)

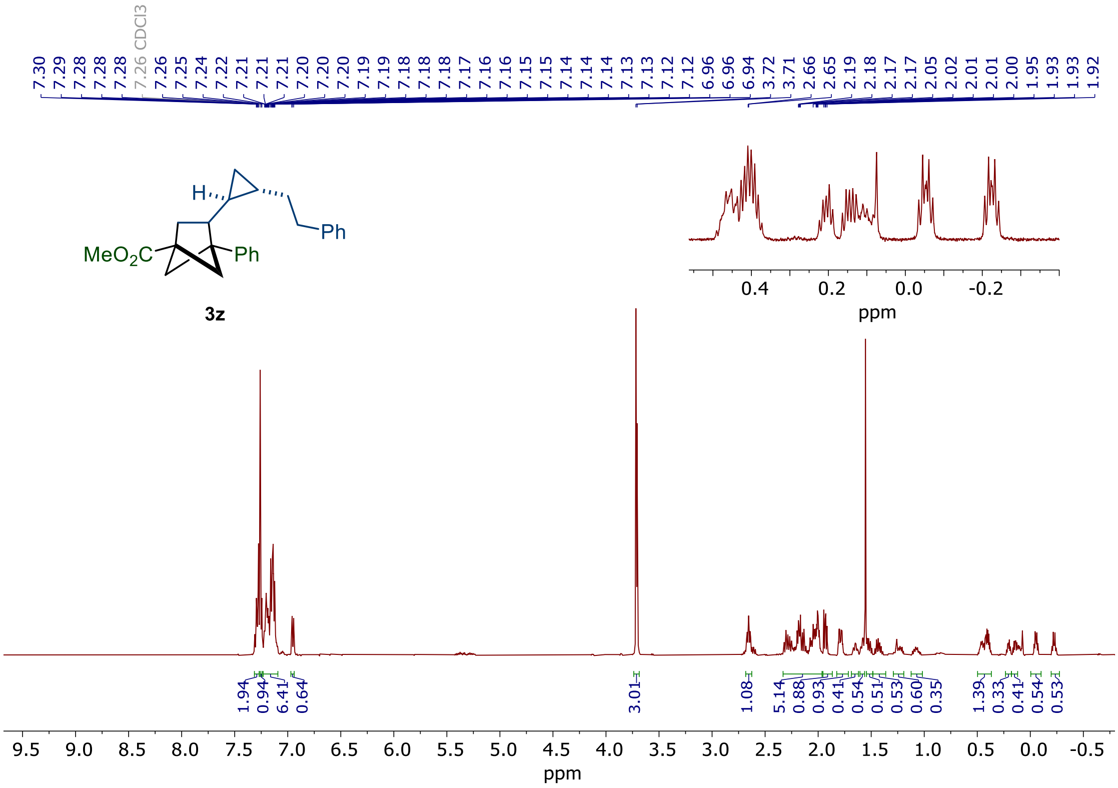

^{13}C NMR (126 MHz, $CDCl_3$) of **3z**

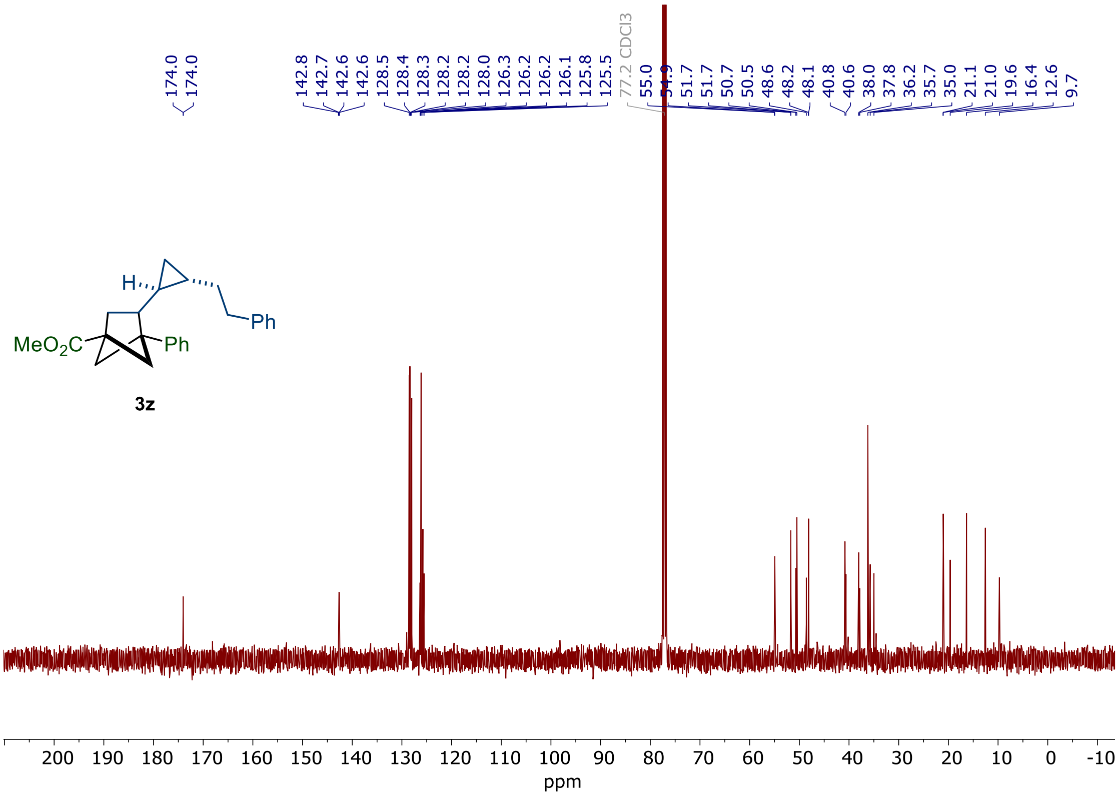

^{1}H NMR (400 MHz, $CDCl_3$) of **5a** (see procedure)

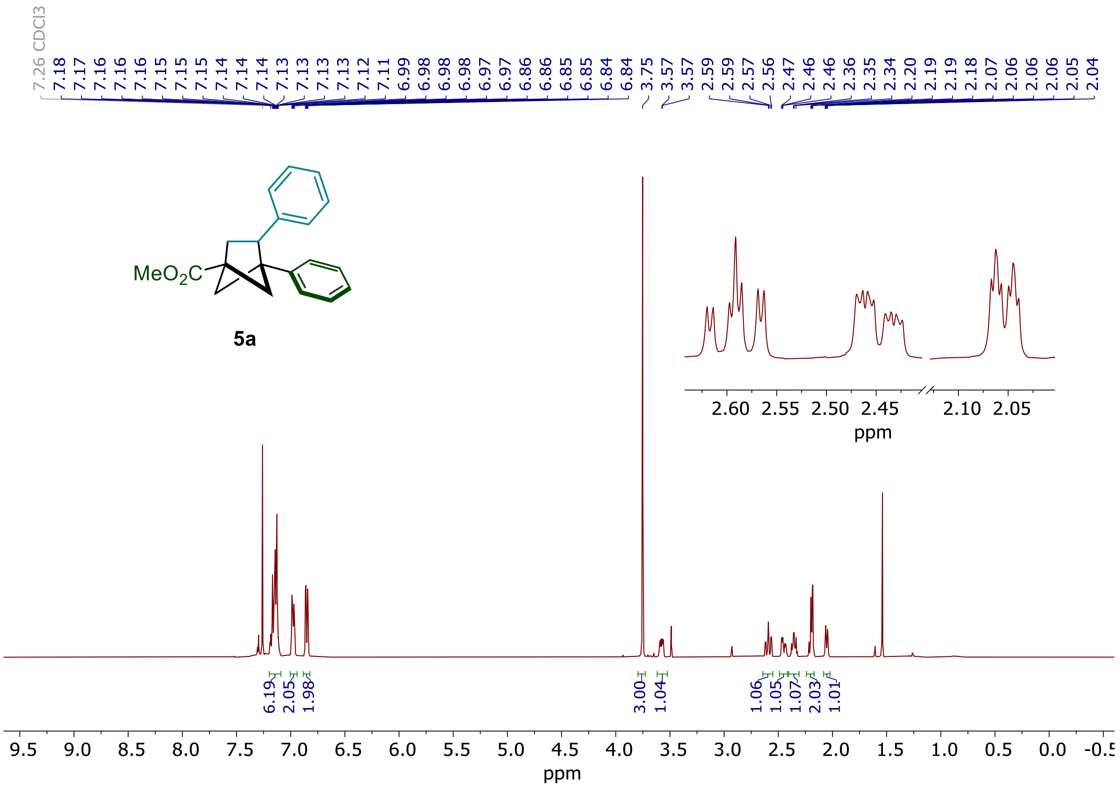

^{13}C NMR (126 MHz, $CDCl_3$) of **5a**

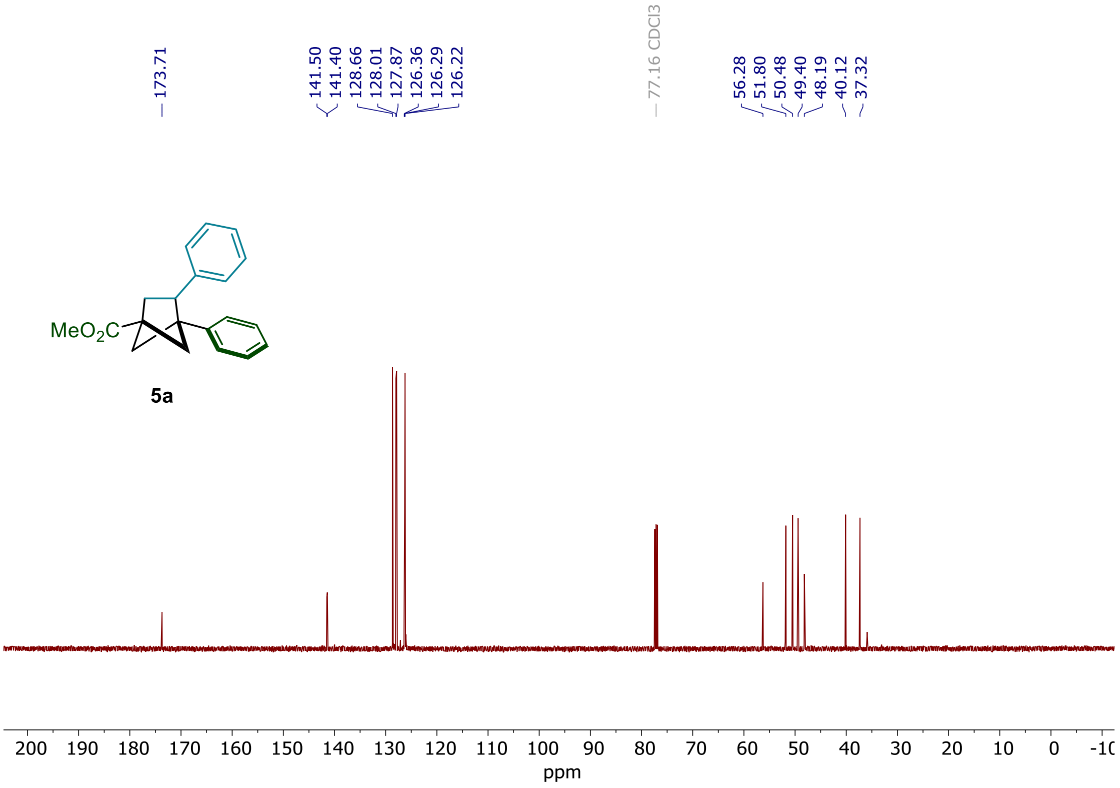

^{1}H NMR (500 MHz, $CDCl_3$) of **5b** (*see procedure*)

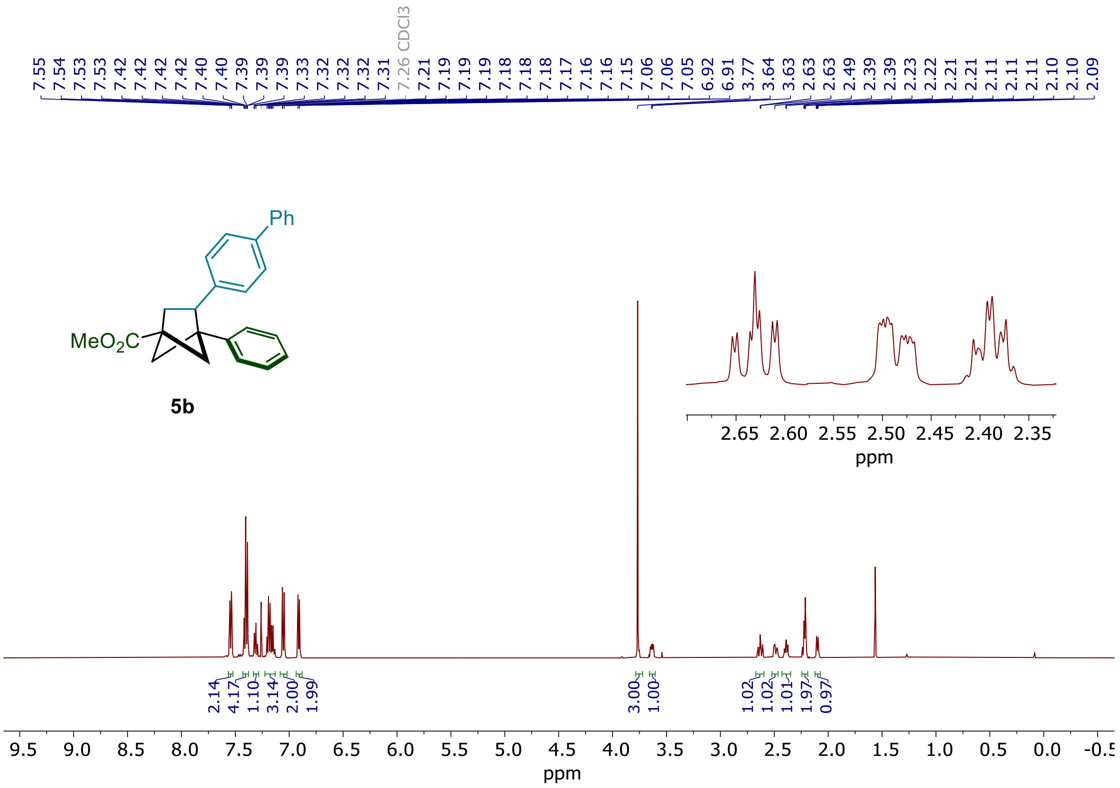

^{13}C NMR (126 MHz, $CDCl_3$) of **5b**

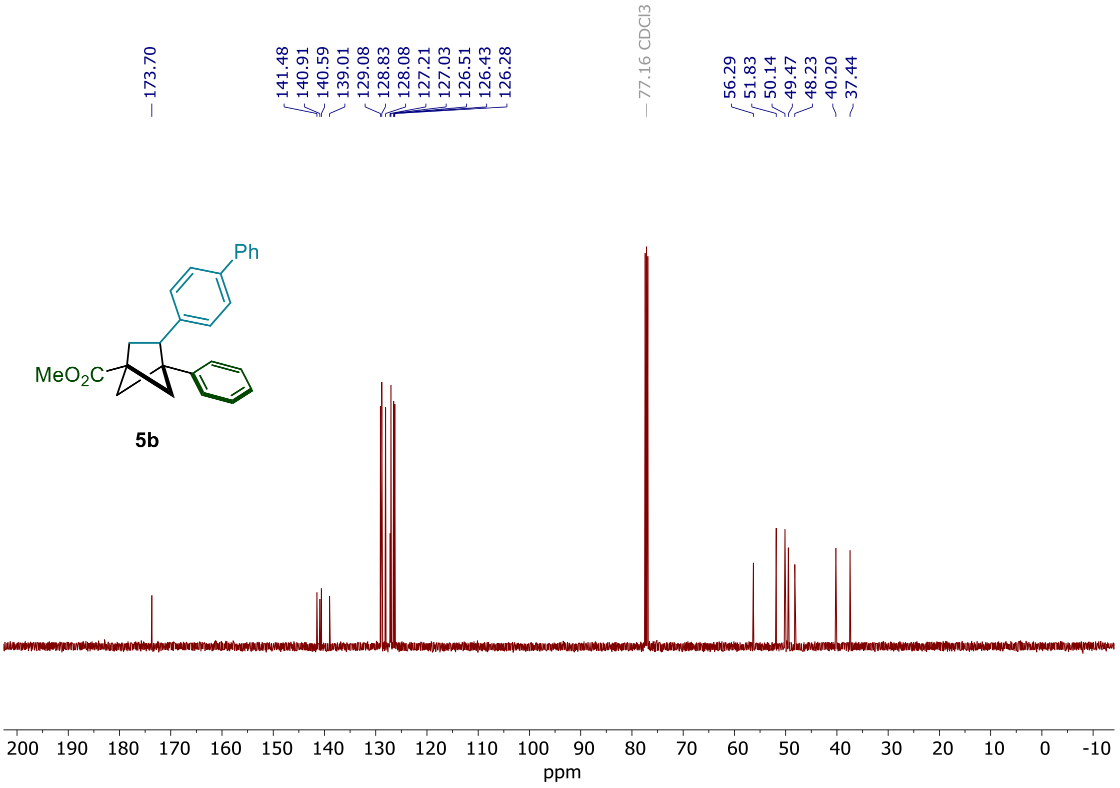

^1H NMR (500 MHz, $CDCl_3$) of **5c** (*see procedure*)

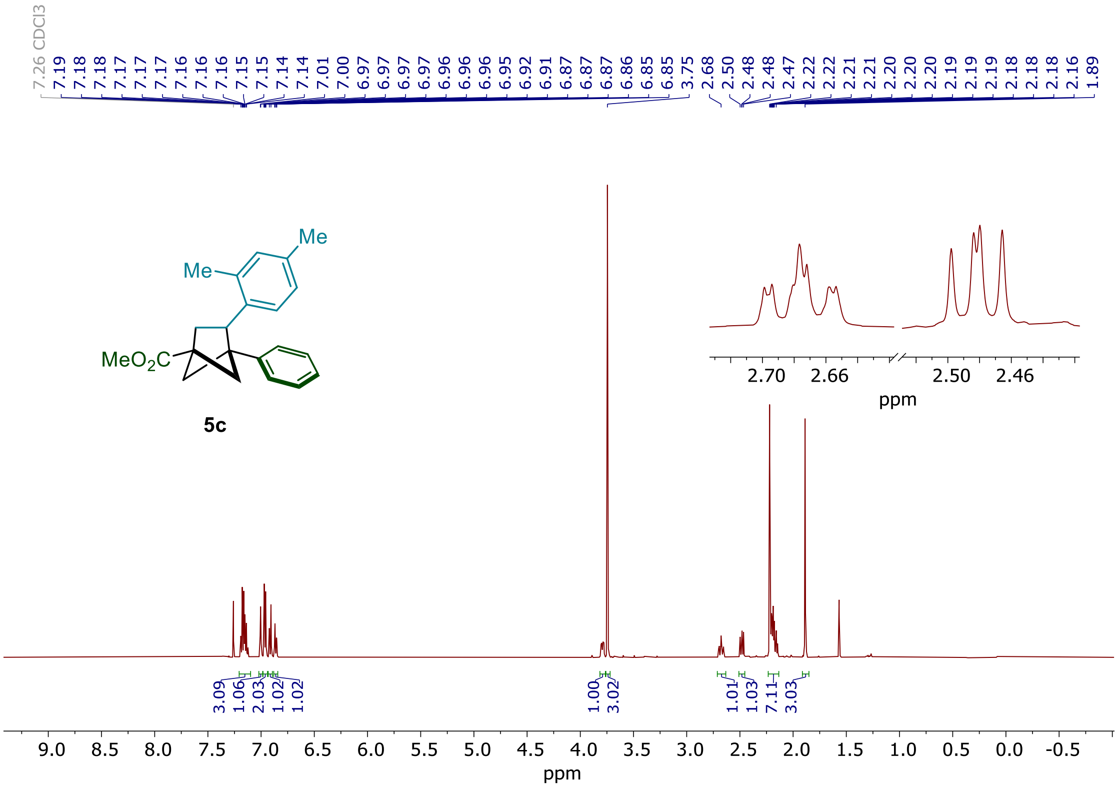

^{13}C NMR (126 MHz, $CDCl_3$) of **5c**

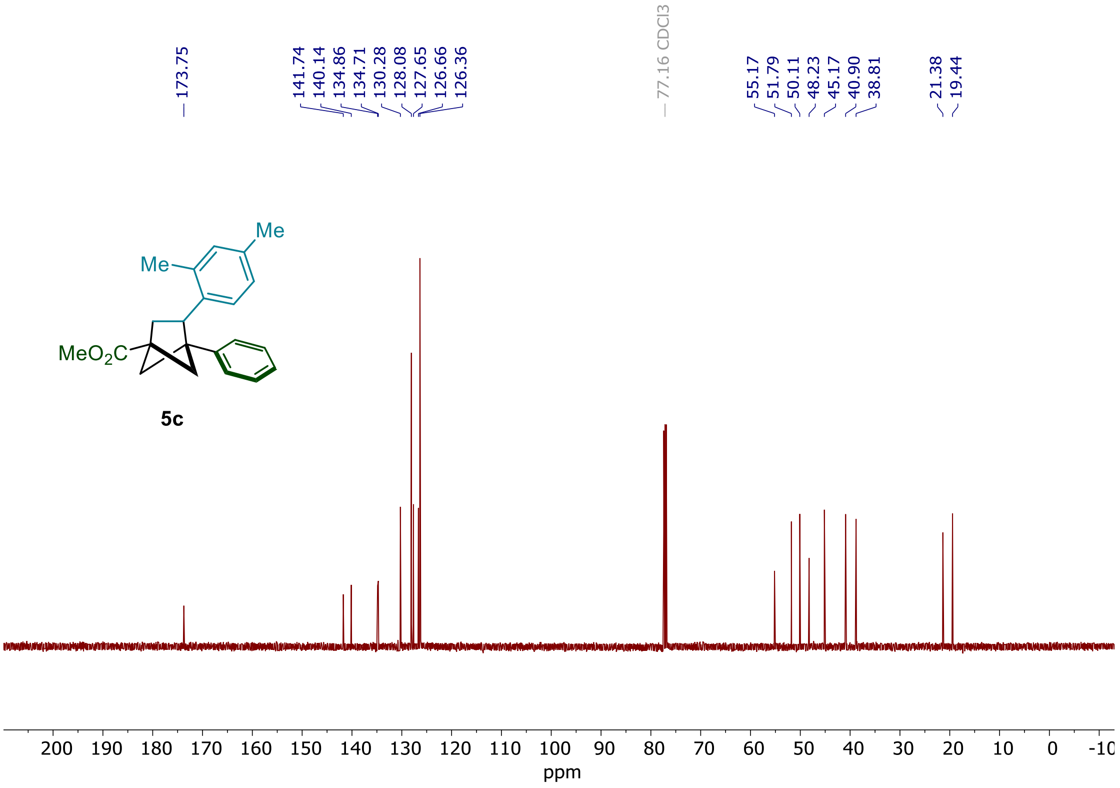

^{1}H NMR (500 MHz, $CDCl_3$) of **5d** (*see procedure*)

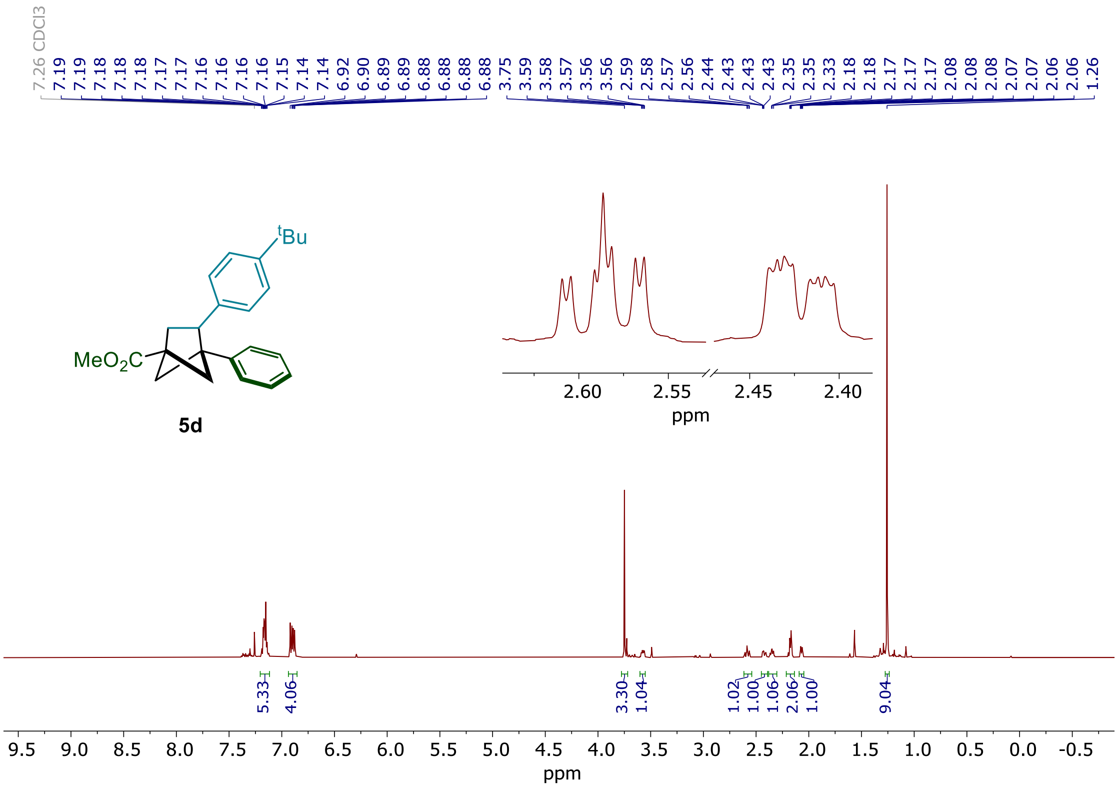

^{13}C NMR (126 MHz, $CDCl_3$) of **5d**

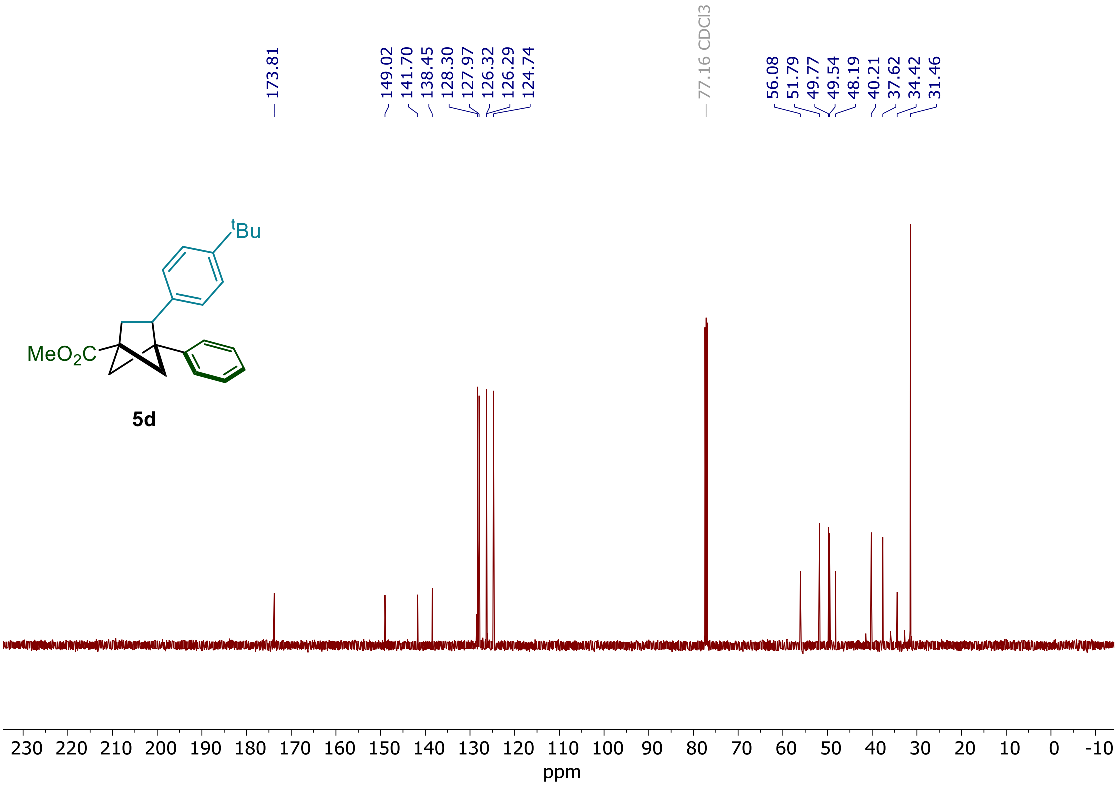

^{1}H NMR (500 MHz, $CDCl_3$) of **5e** (*see procedure*)

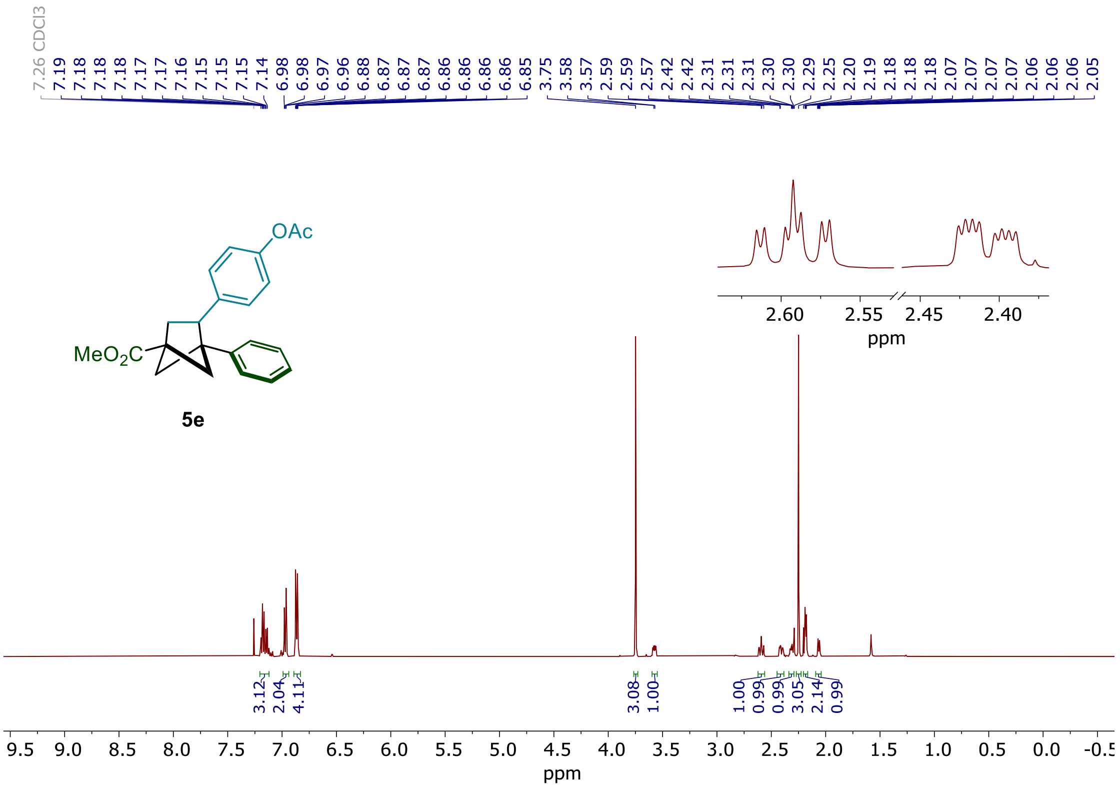

^{13}C NMR (126 MHz, $CDCl_3$) of **5e**

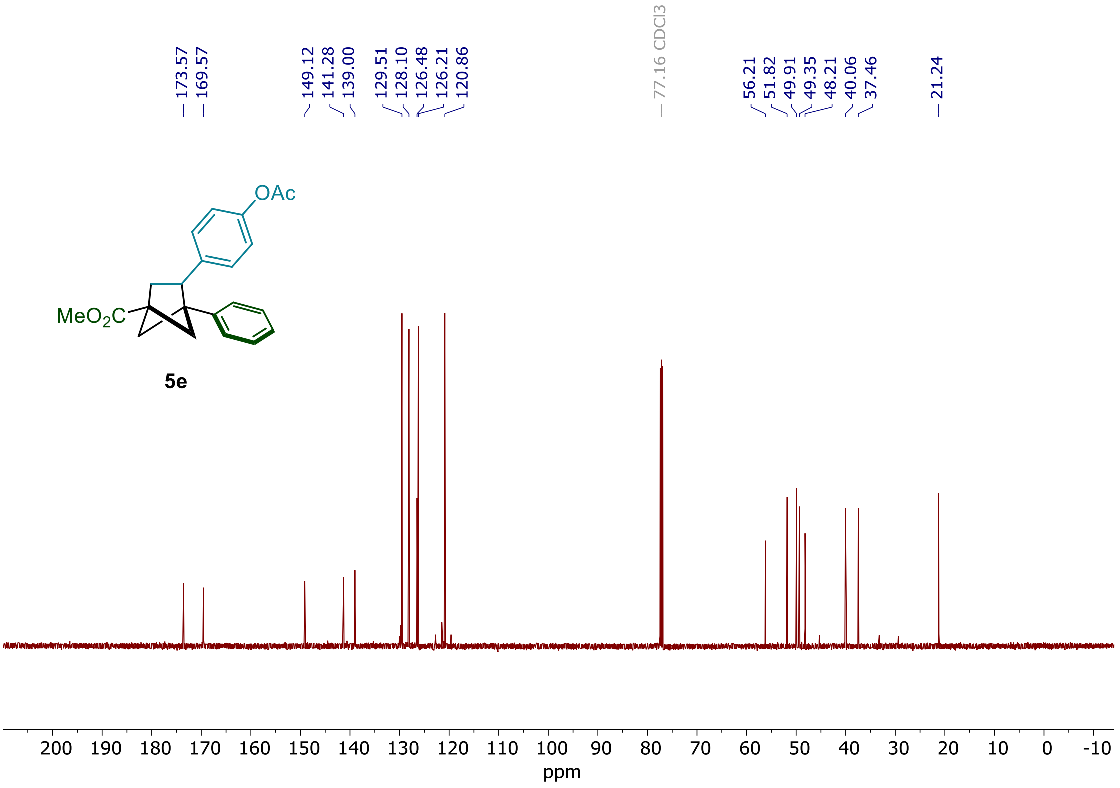

^{1}H NMR (400 MHz, $CDCl_3$) of **5f** (*see procedure*)

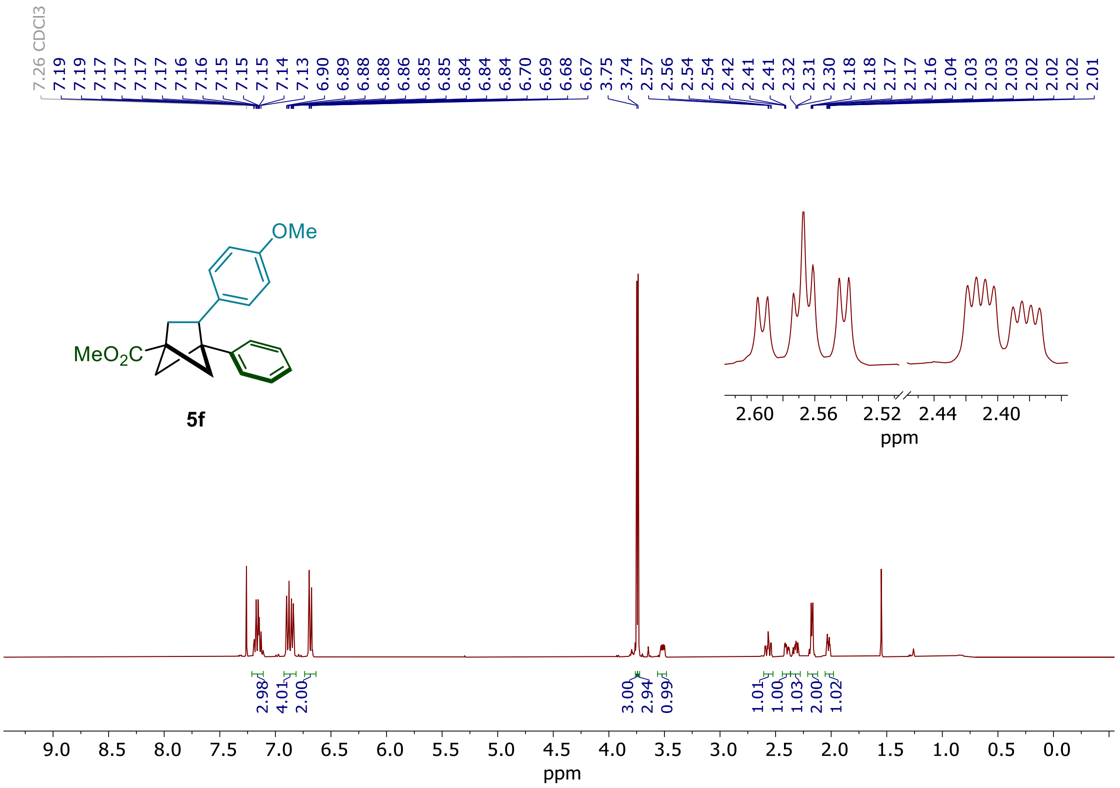

^{13}C NMR (126 MHz, $CDCl_3$) of **5f**

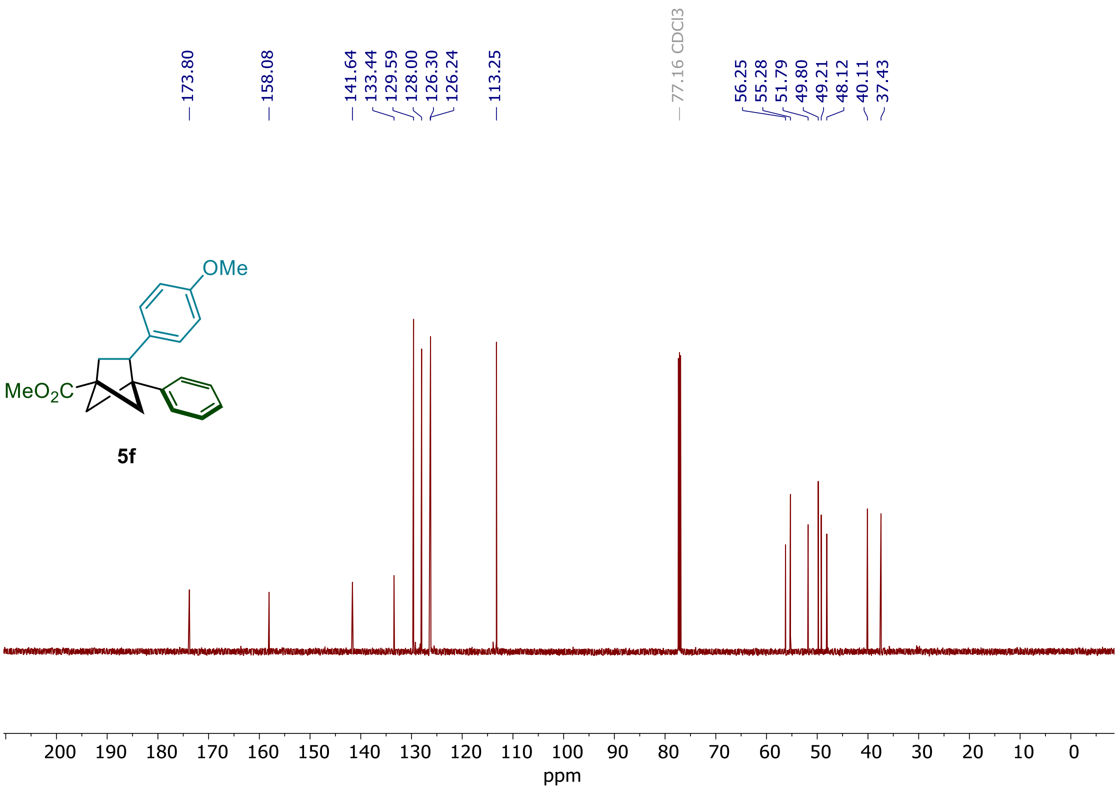

^{1}H NMR (500 MHz, $CDCl_3$) of **5g** (*see procedure*)

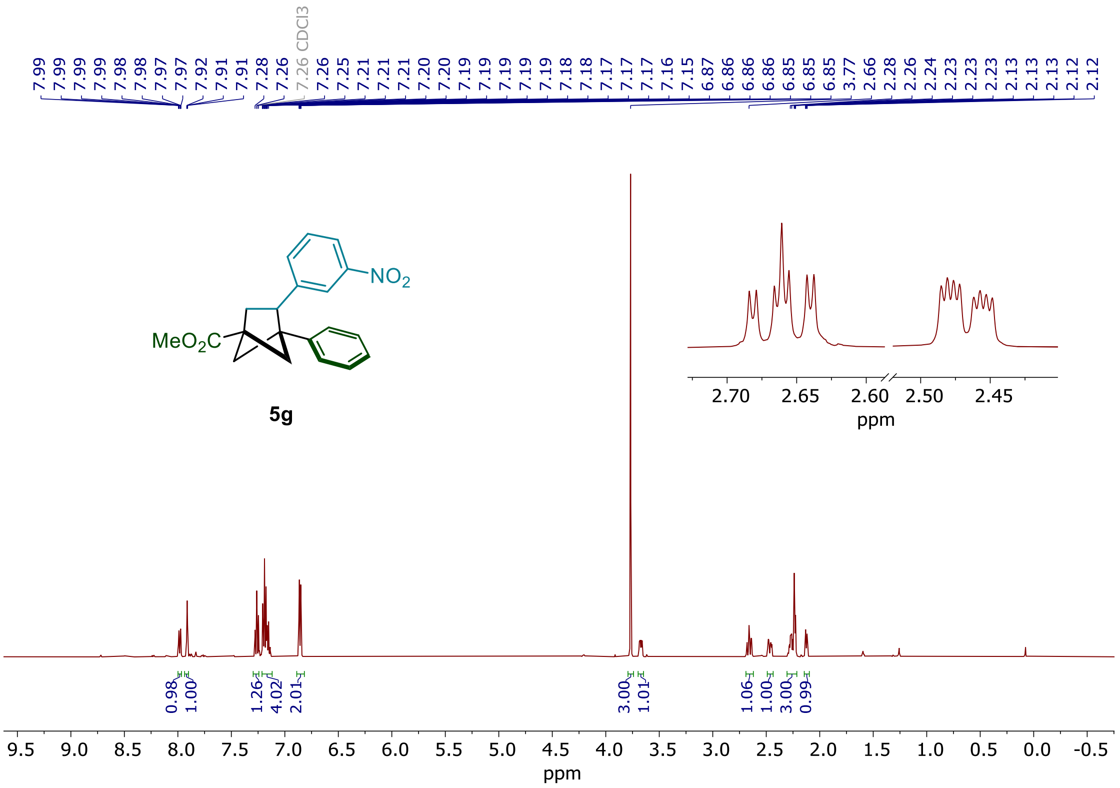

^{13}C NMR (126 MHz, $CDCl_3$) of **5g**

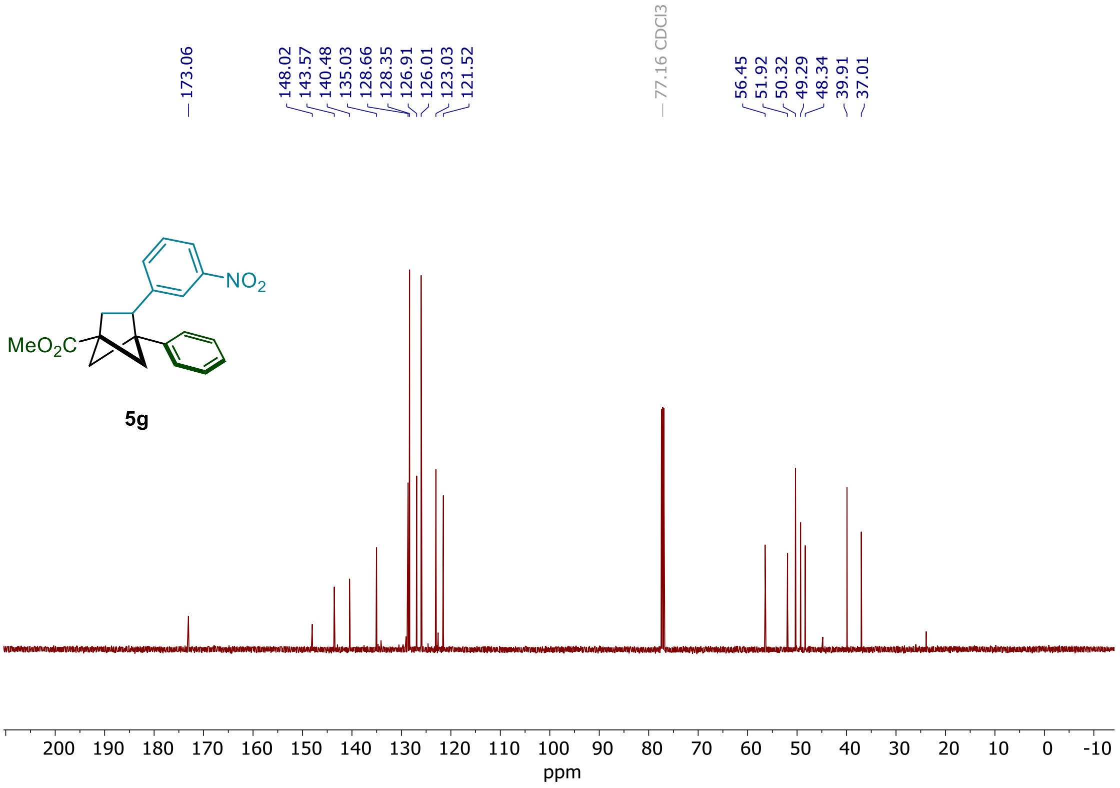

^{1}H NMR (400 MHz, $CDCl_3$) of **5h** (*see procedure*)

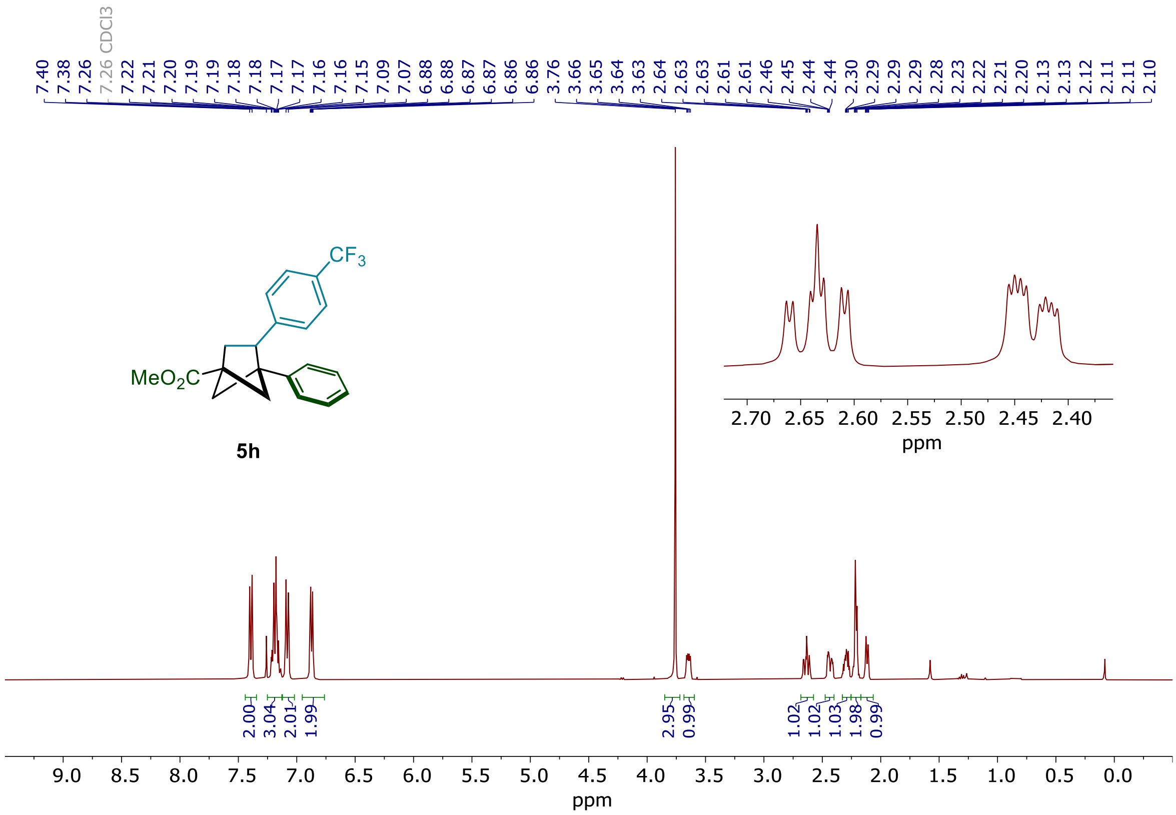

^{13}C NMR (101 MHz, $CDCl_3$) of **5h**

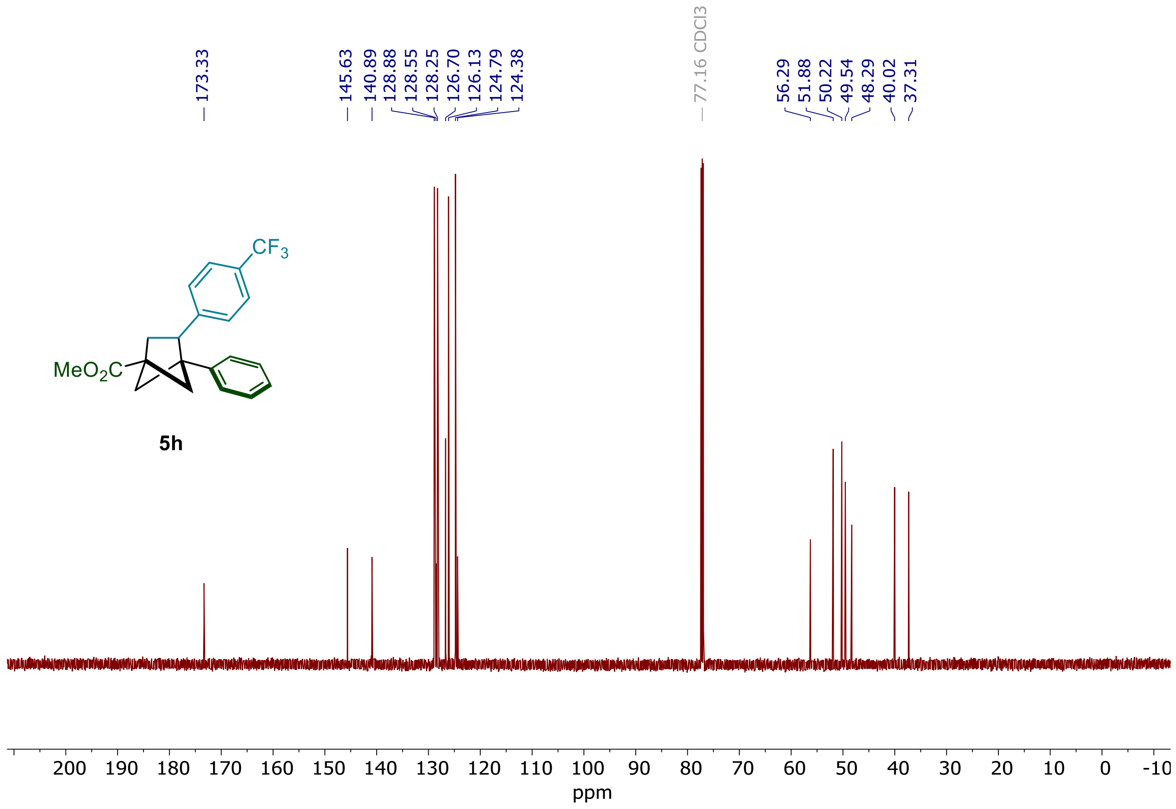

^{19}F NMR (376 MHz, $CDCl_3$) of **5h**

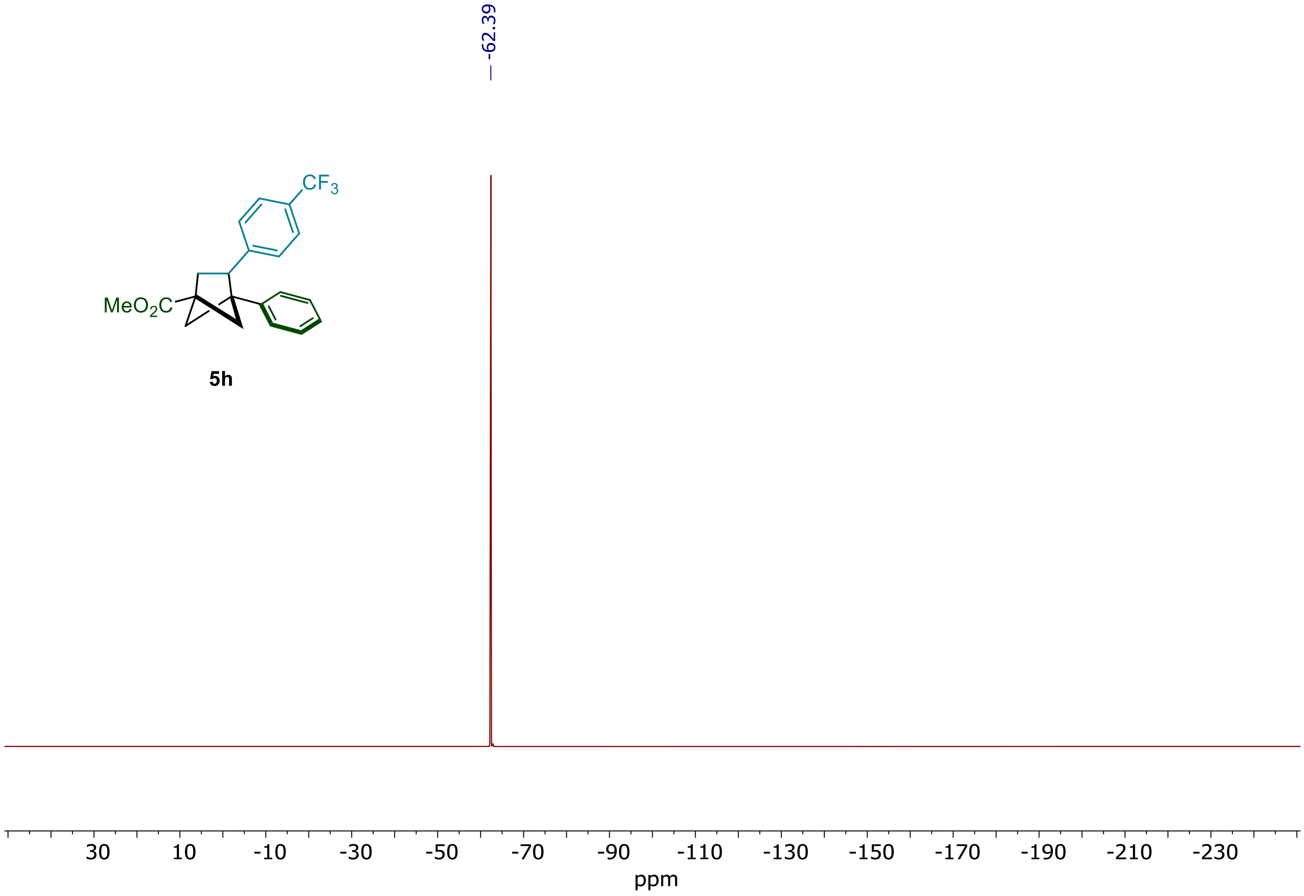

^{1}H NMR (500 MHz, $CDCl_3$) of **5i** (*see procedure*)

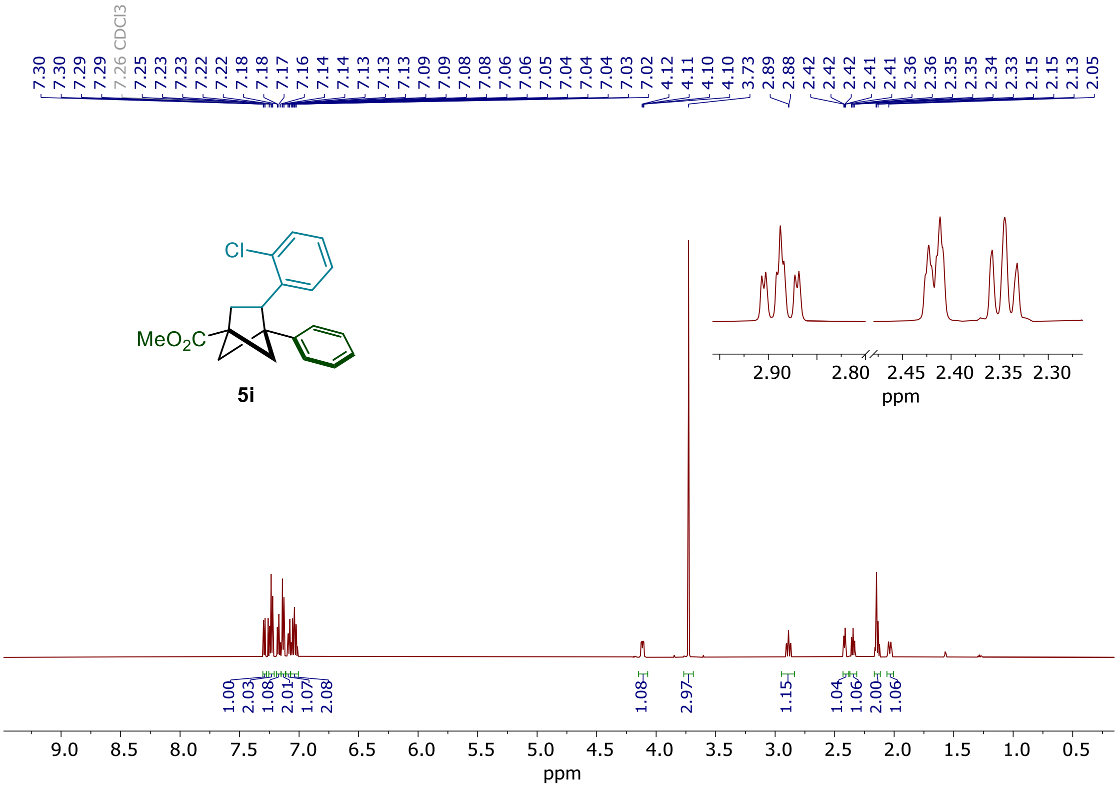

^{13}C NMR (126 MHz, $CDCl_3$) of **5i**

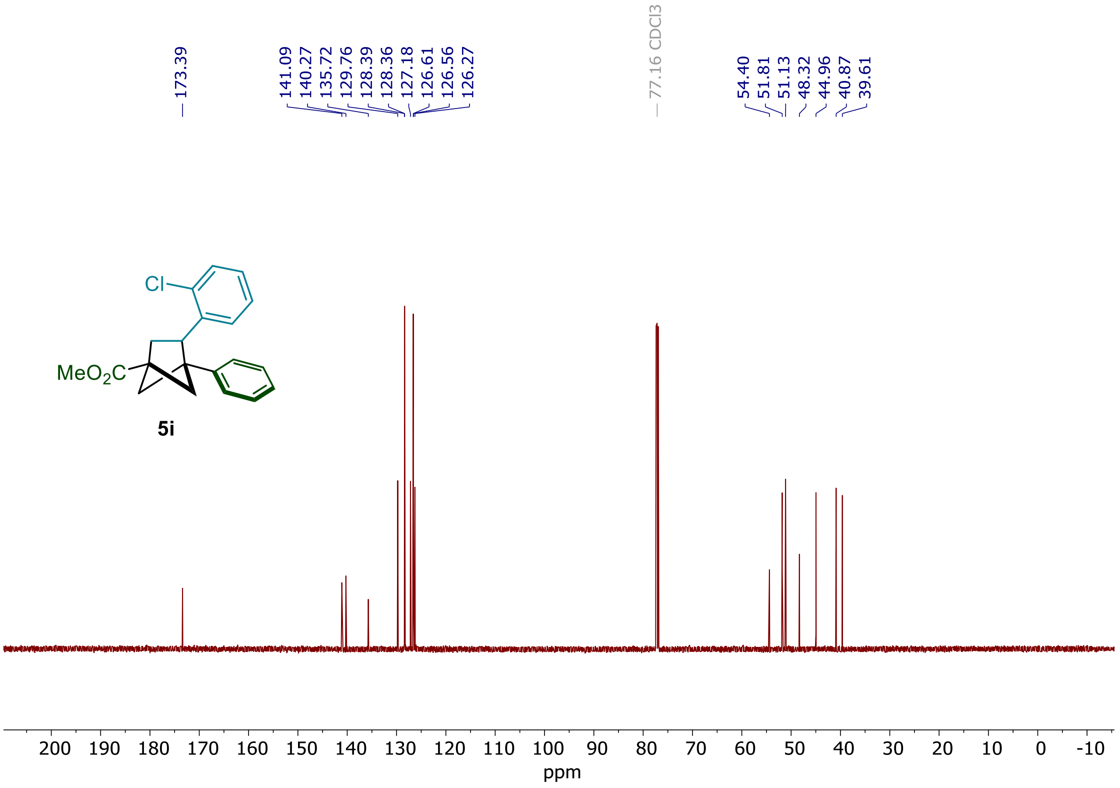

^{1}H NMR (400 MHz, $CDCl_3$) of **5j** (*see procedure*)

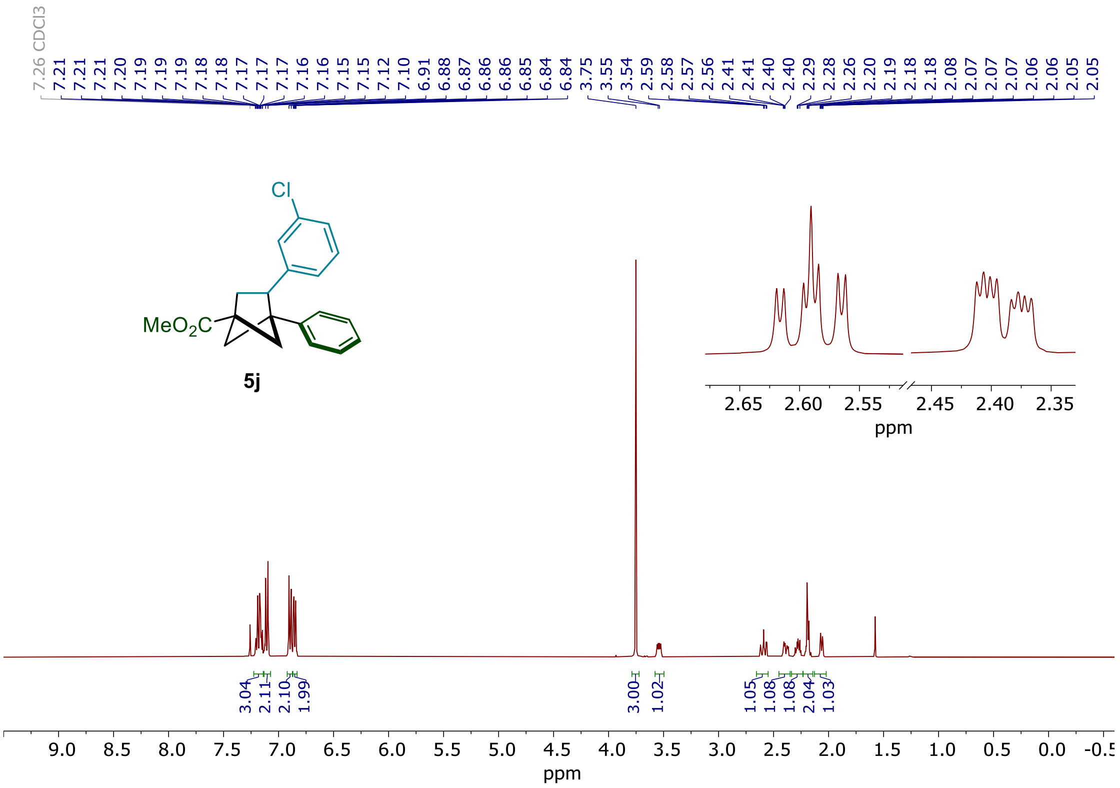

^{13}C NMR (126 MHz, $CDCl_3$) of **5j**

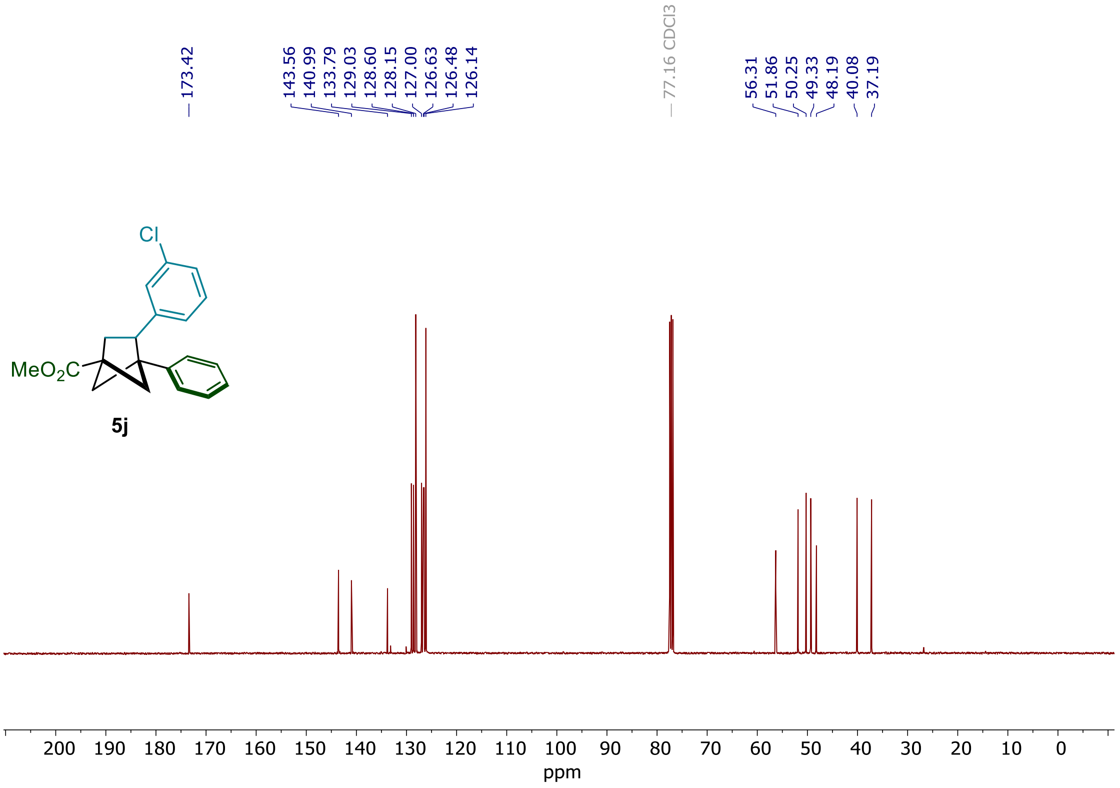

^{1}H NMR (400 MHz, $CDCl_3$) of **5k** (*see procedure*)

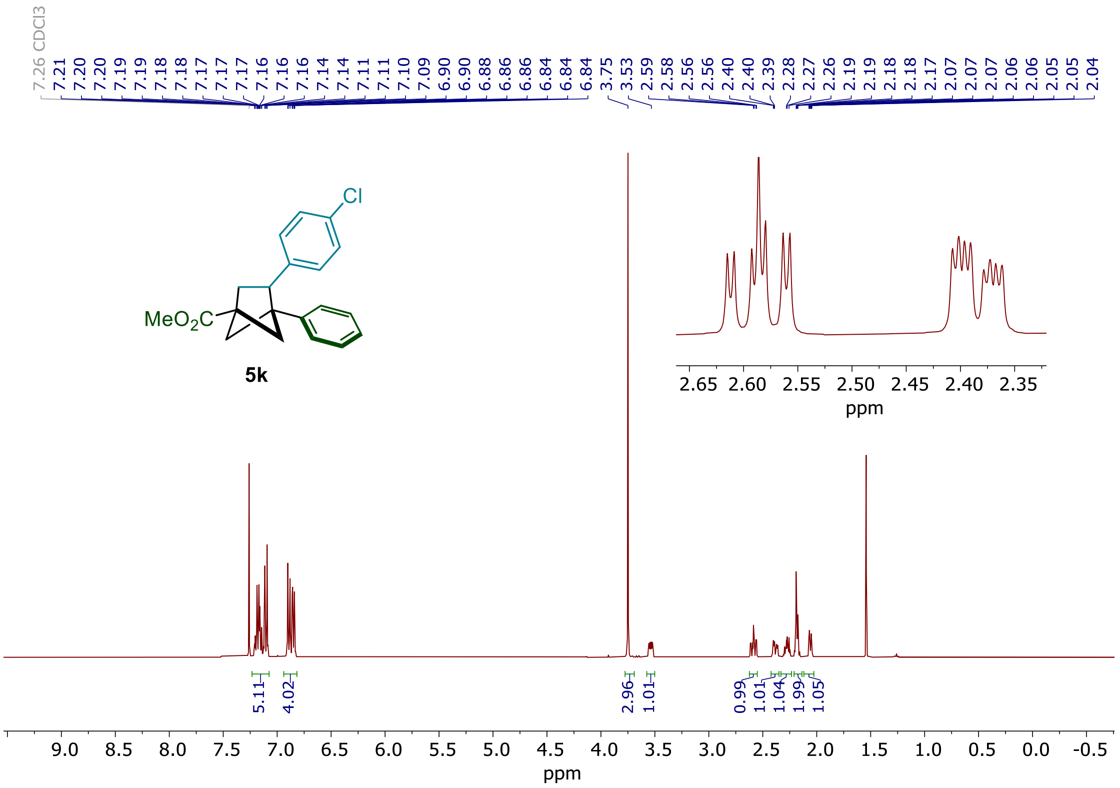

^{13}C NMR (101 MHz, $CDCl_3$) of **5k**

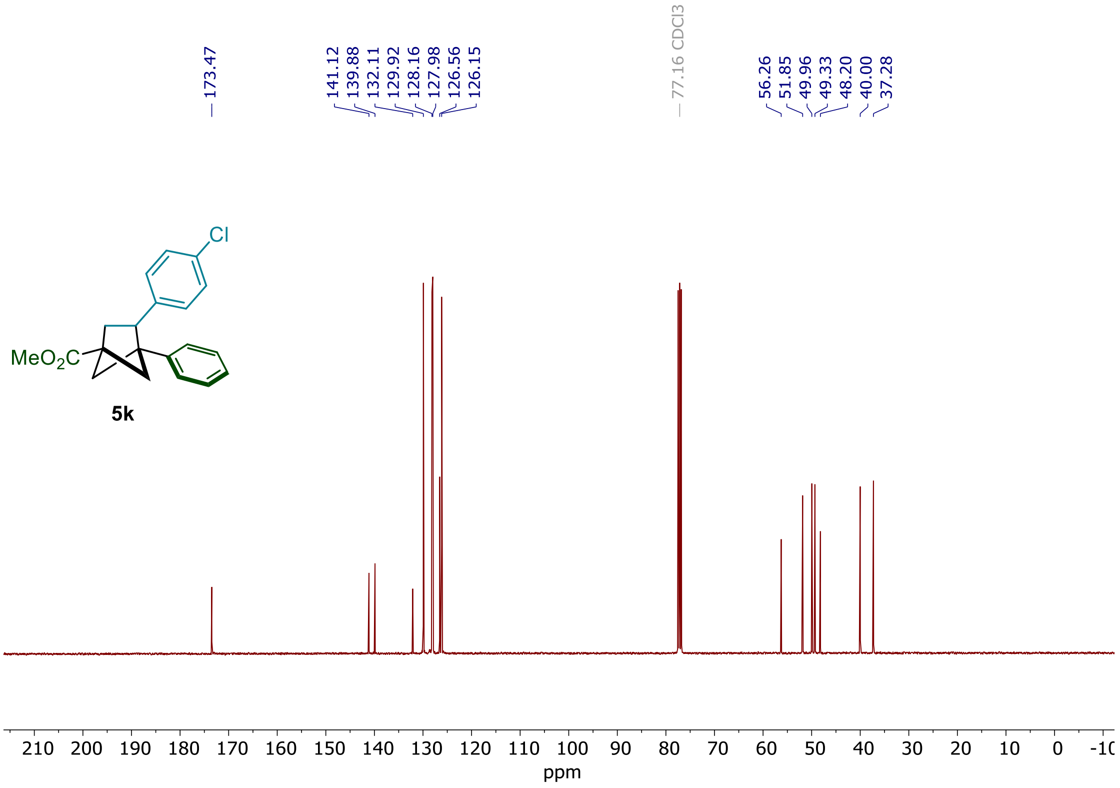

^{1}H NMR (400 MHz, $CDCl_3$) of **5l** (*see procedure*)

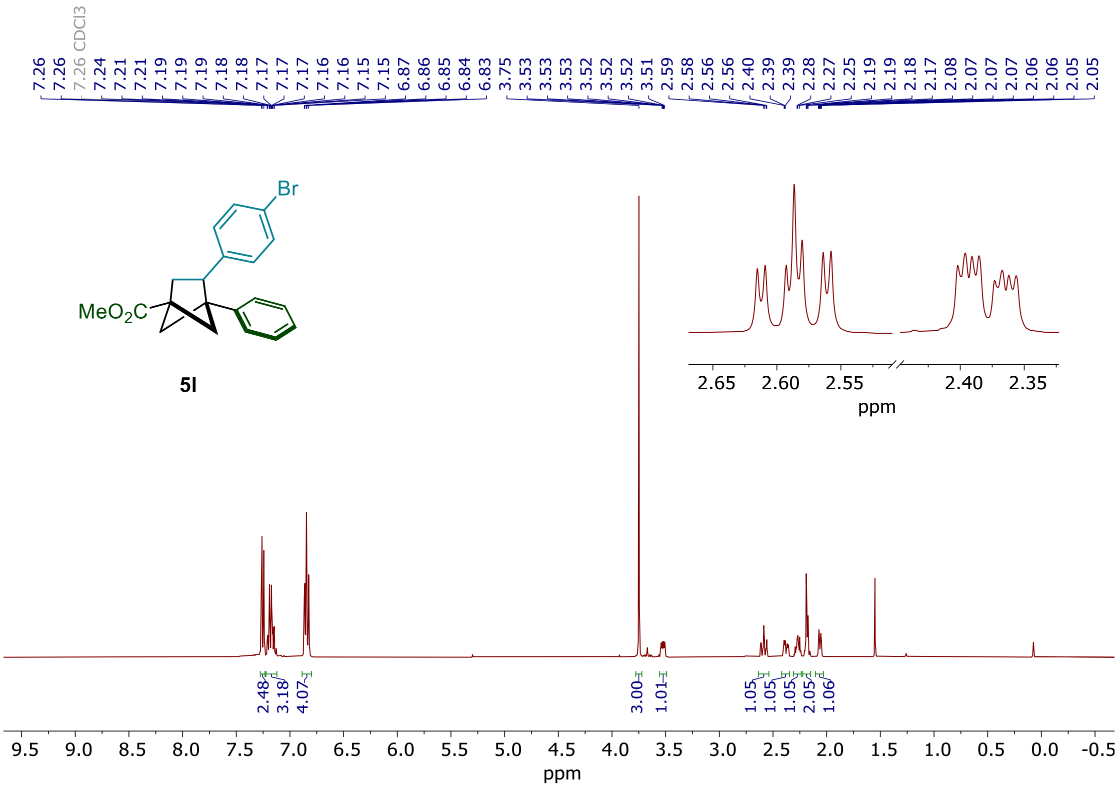

^{13}C NMR (101 MHz, $CDCl_3$) of **5l**

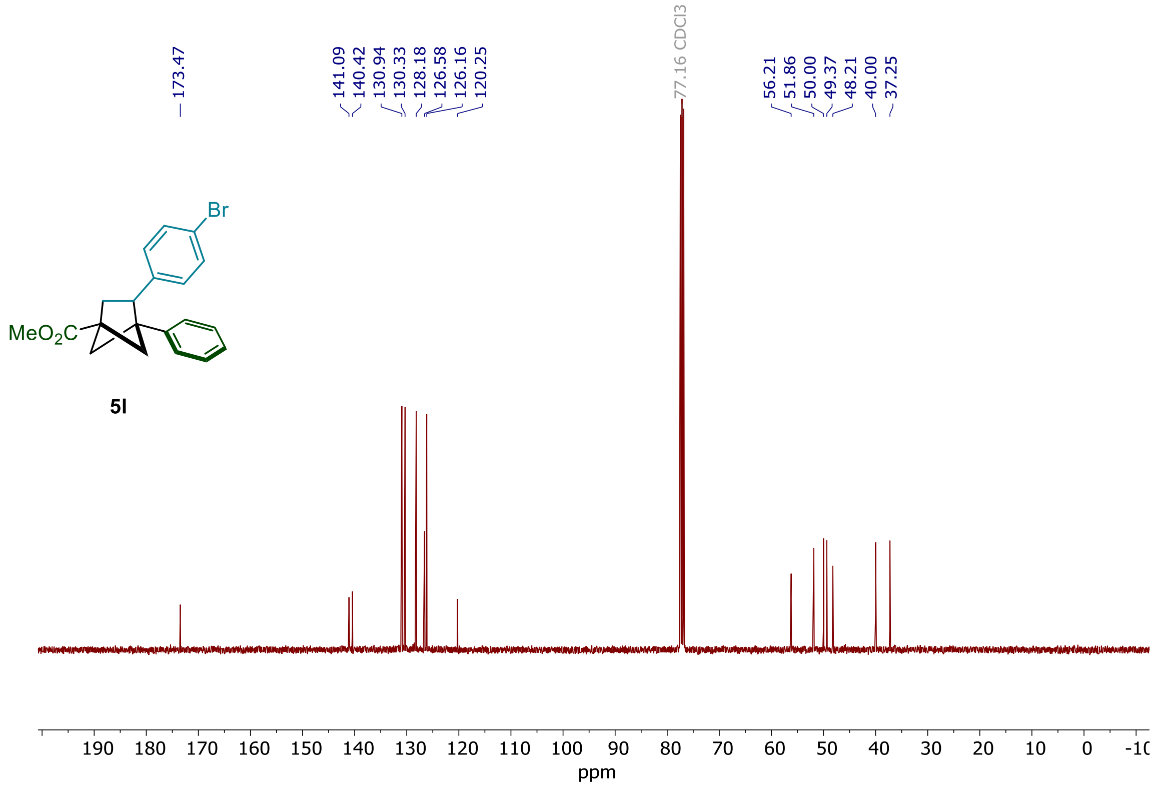

^{1}H NMR (500 MHz, $CDCl_3$) of **5m** (<u>*see procedure*</u>)

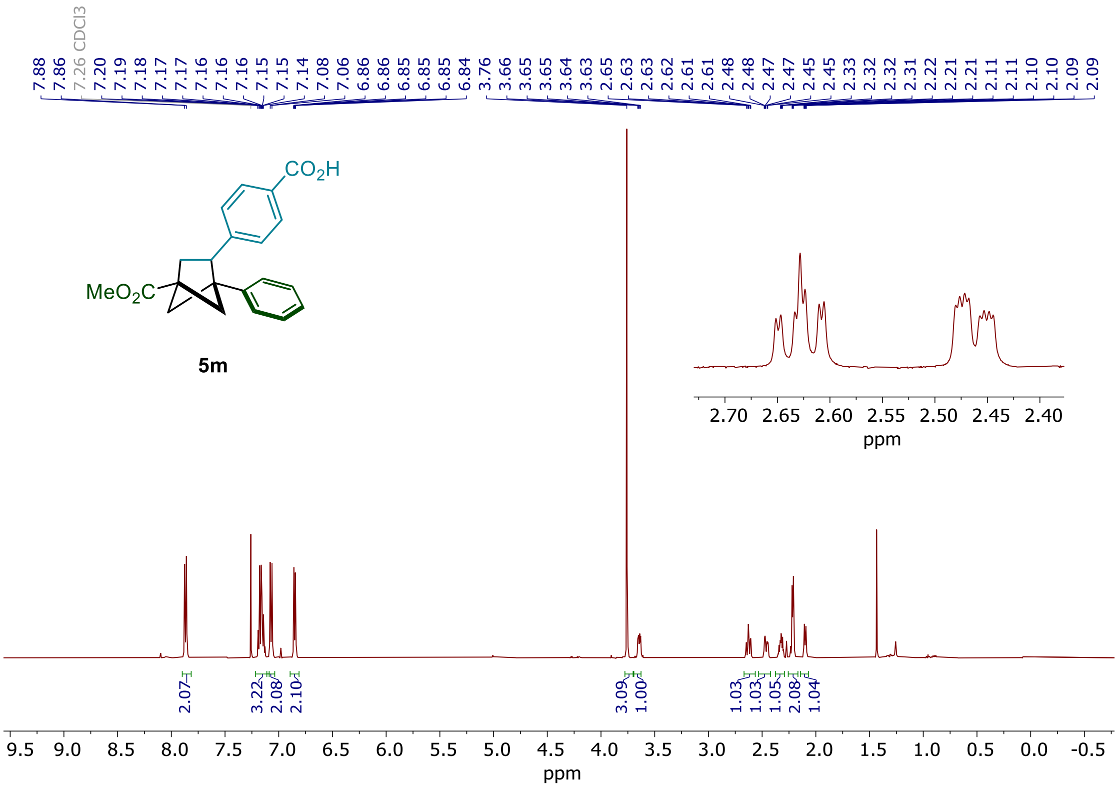

^{13}C NMR (126 MHz, $CDCl_3$) of **5m**

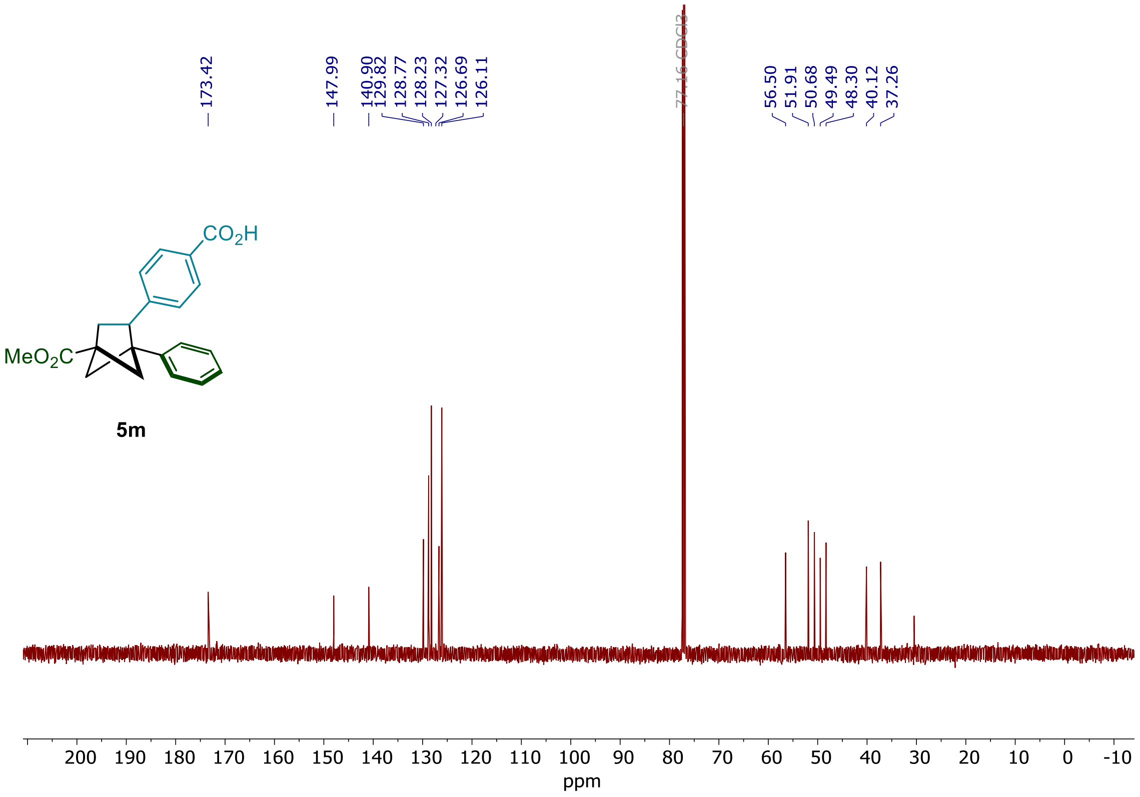

^{1}H NMR (500 MHz, $CDCl_3$) of **5n** (*see procedure*)

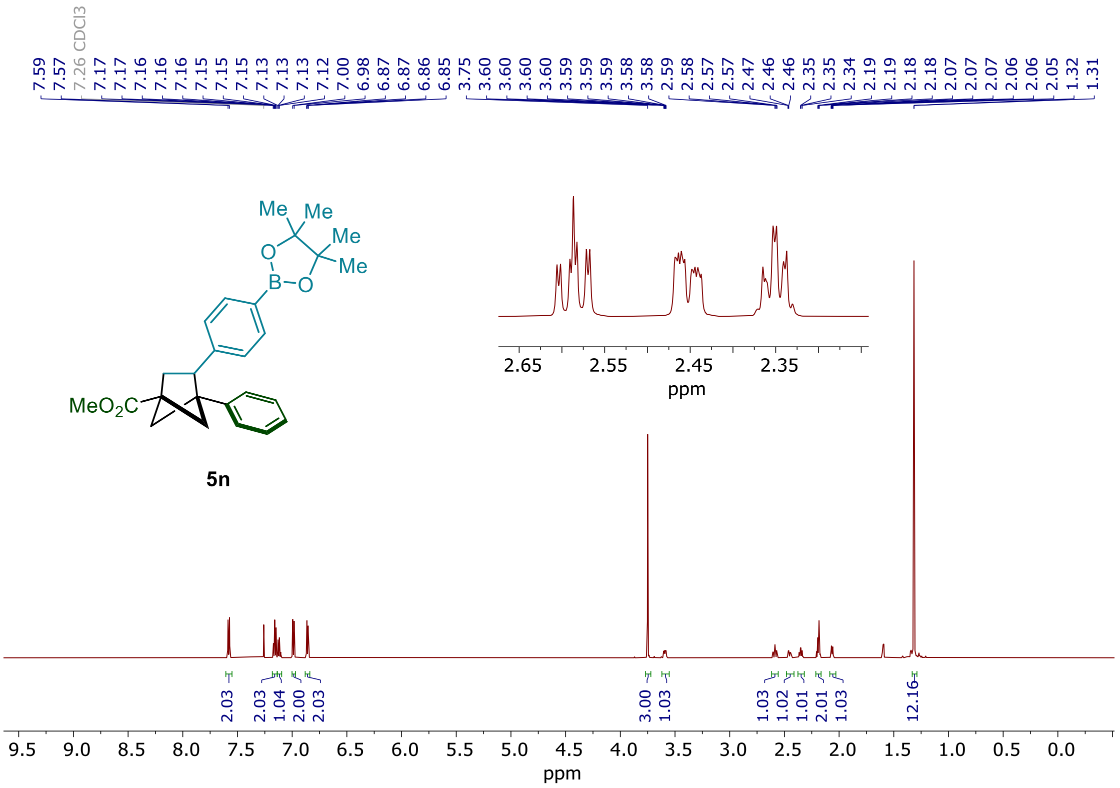

^{13}C NMR (126 MHz, $CDCl_3$) of **5n**

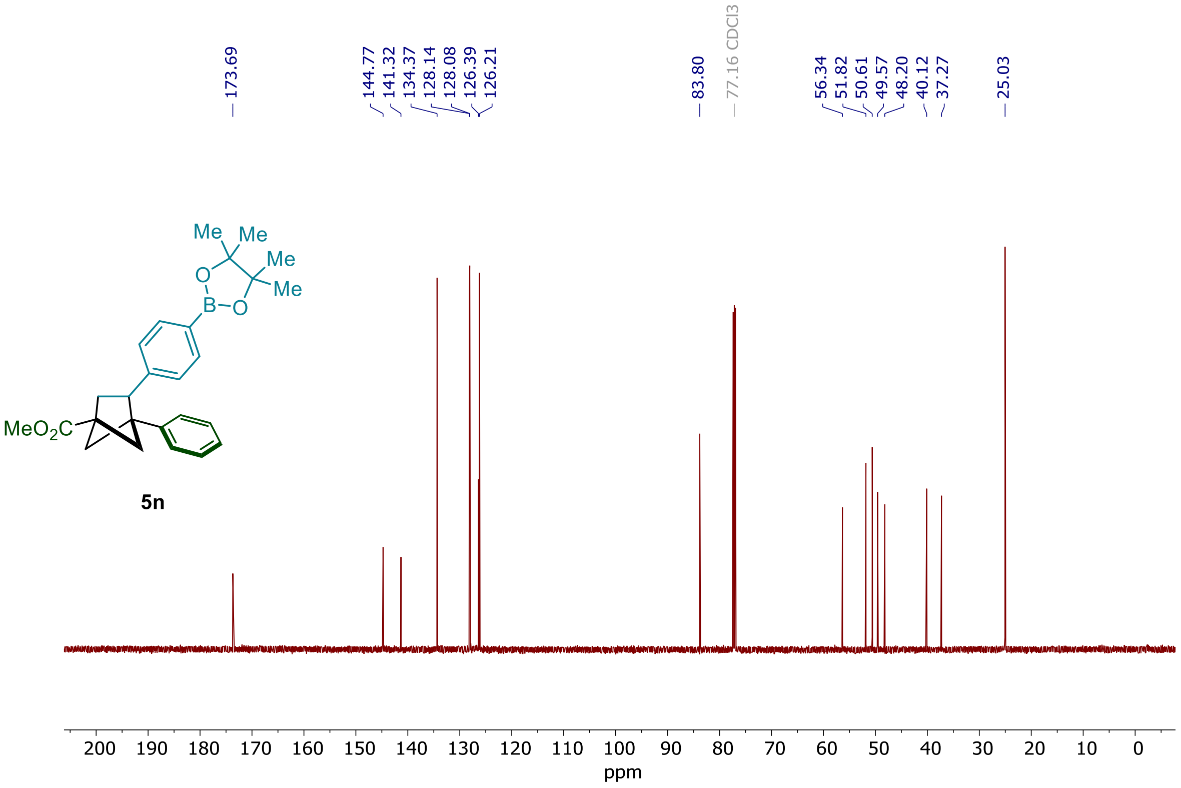

^{1}H NMR (500 MHz, $CDCl_3$) of **5o** (see procedure)

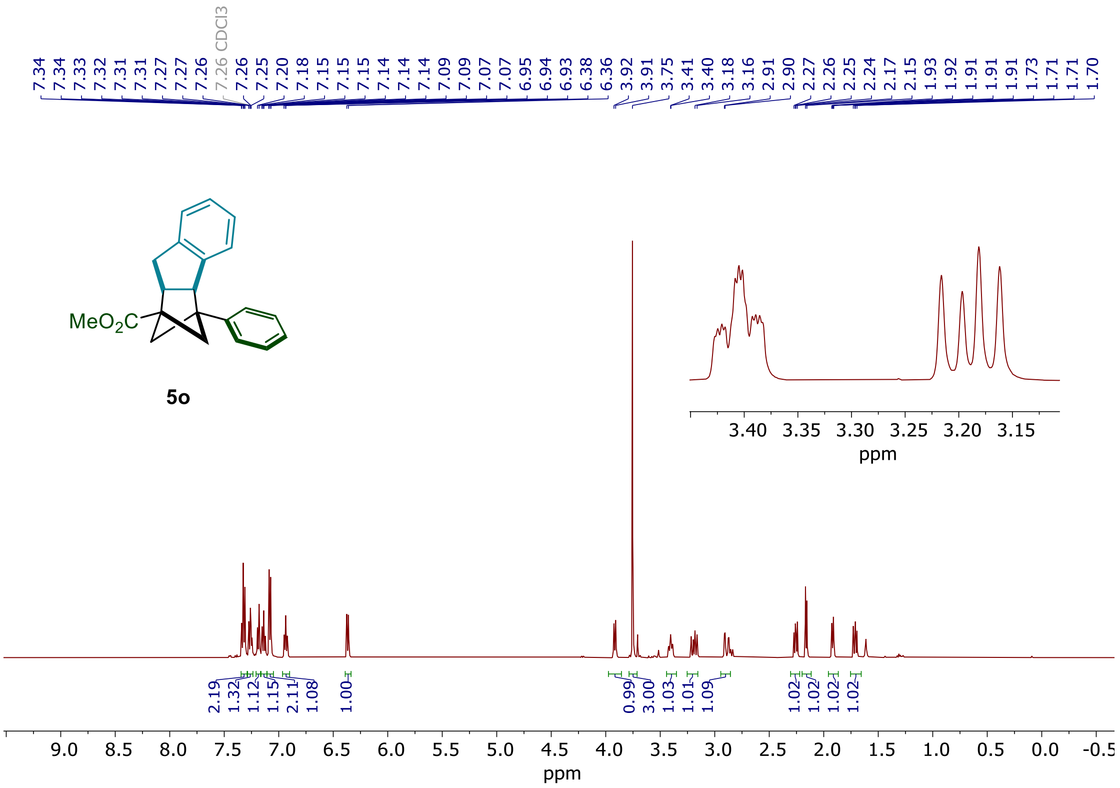

^{13}C NMR (126 MHz, $CDCl_3$) of **5o**

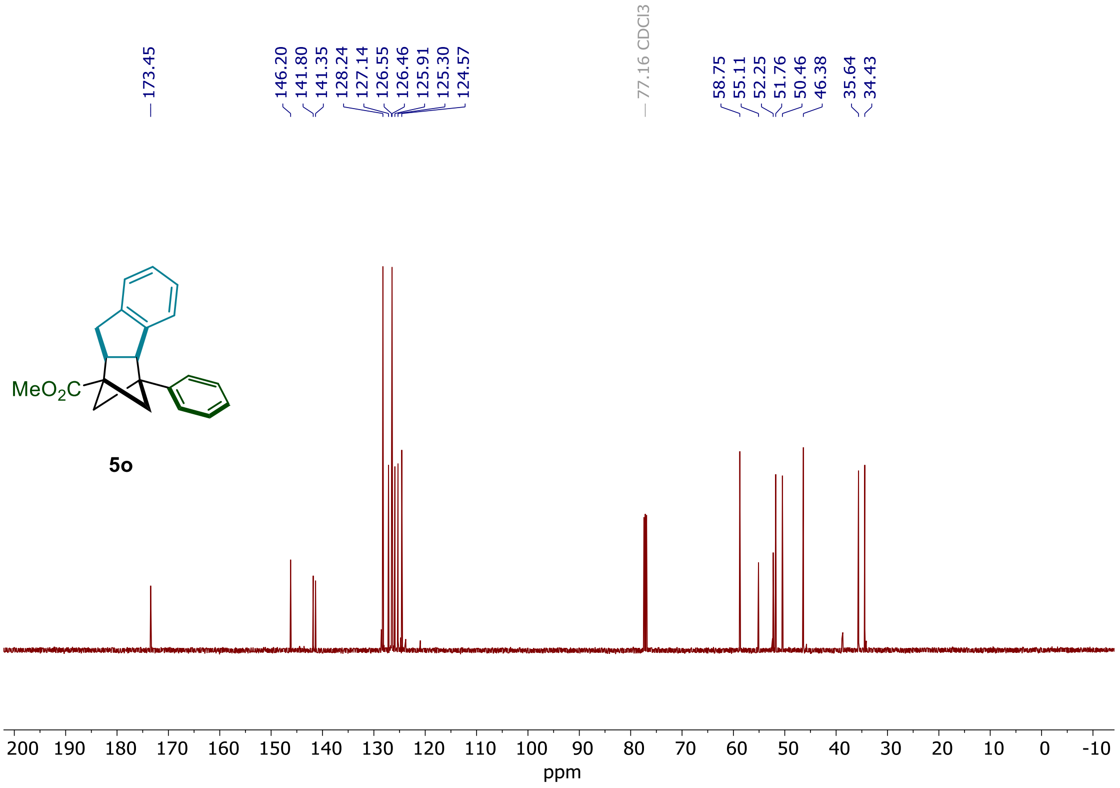

^{1}H NMR (500 MHz, $CDCl_3$) of **5p** (*see procedure*)

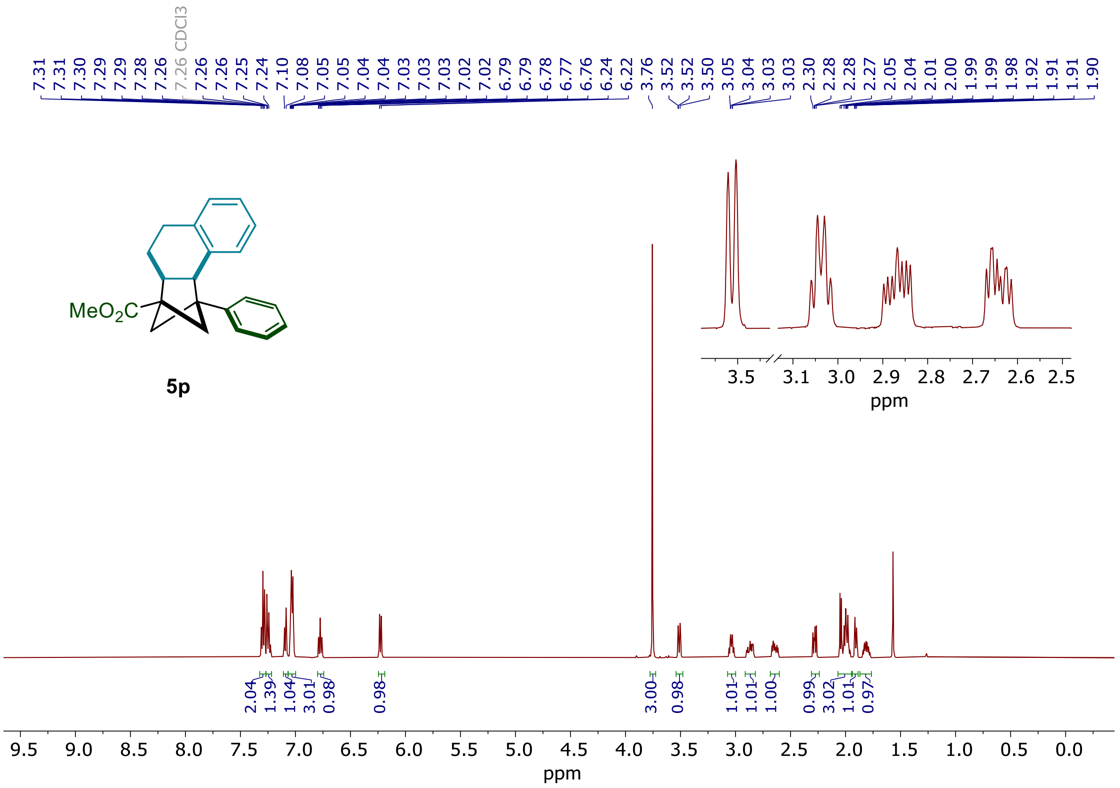

^{13}C NMR (126 MHz, $CDCl_3$) of **5p**

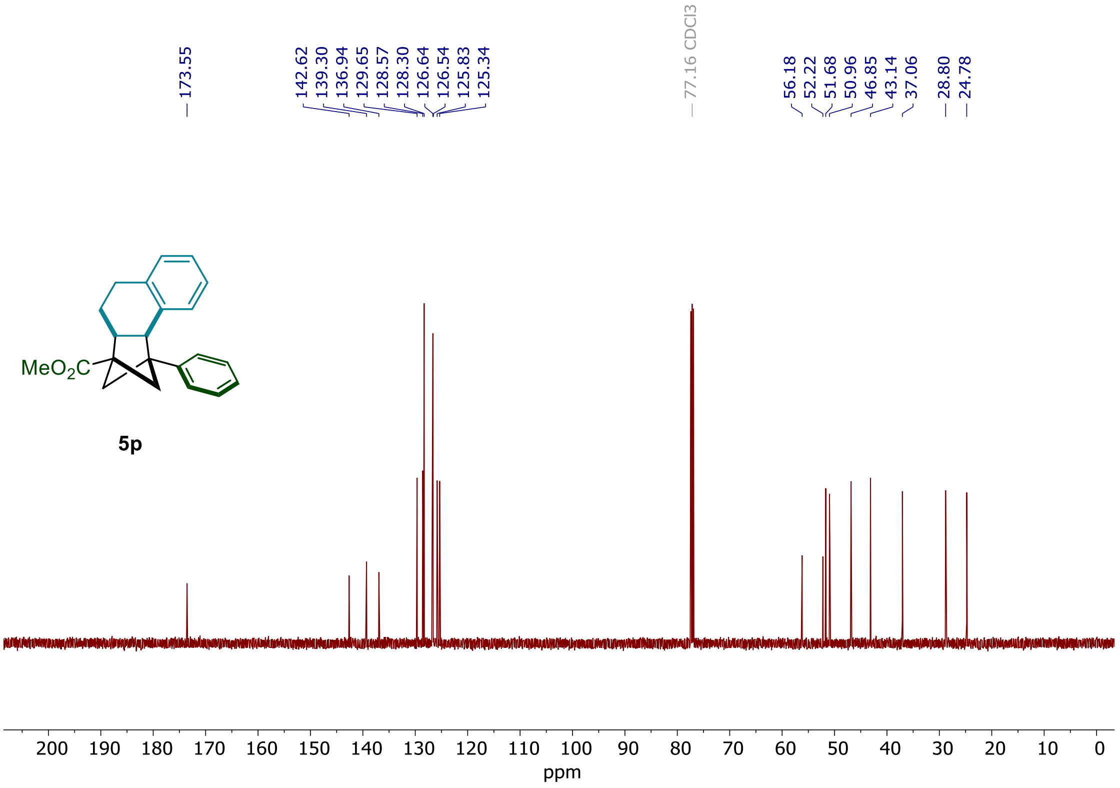

^{1}H NMR (500 MHz, $CDCl_3$) of **5q** (*see procedure*)

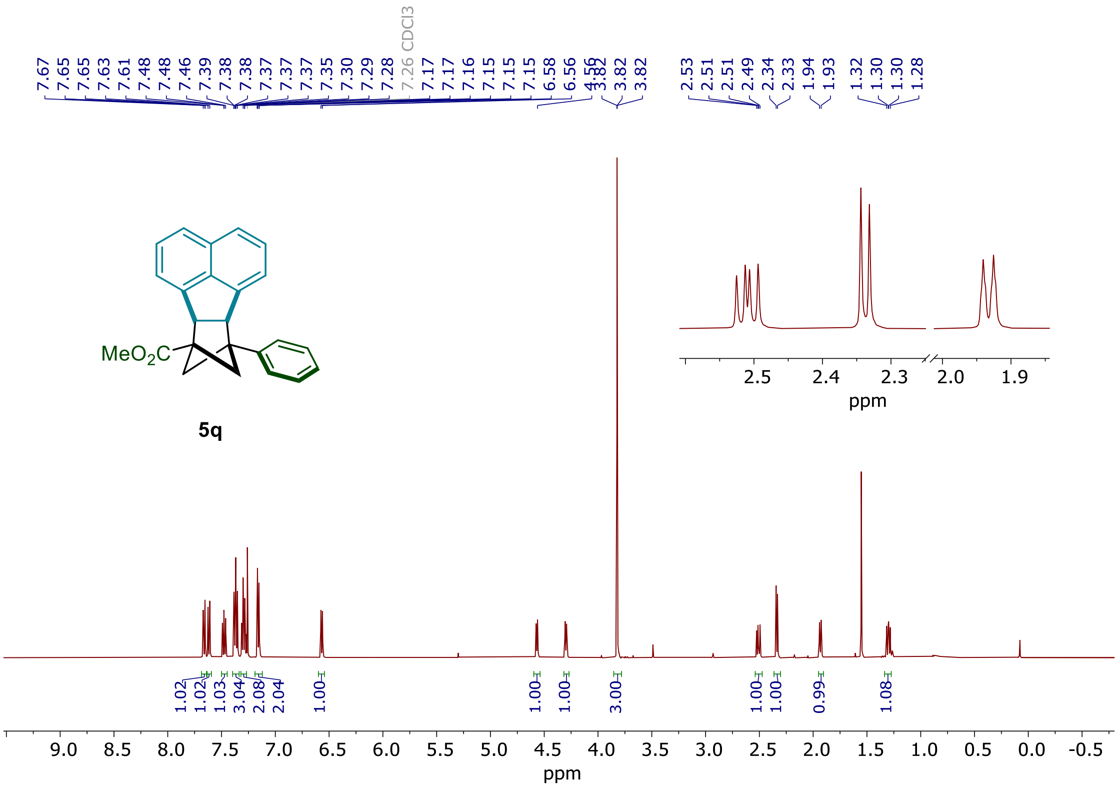

^{13}C NMR (126 MHz, $CDCl_3$) of **5q**

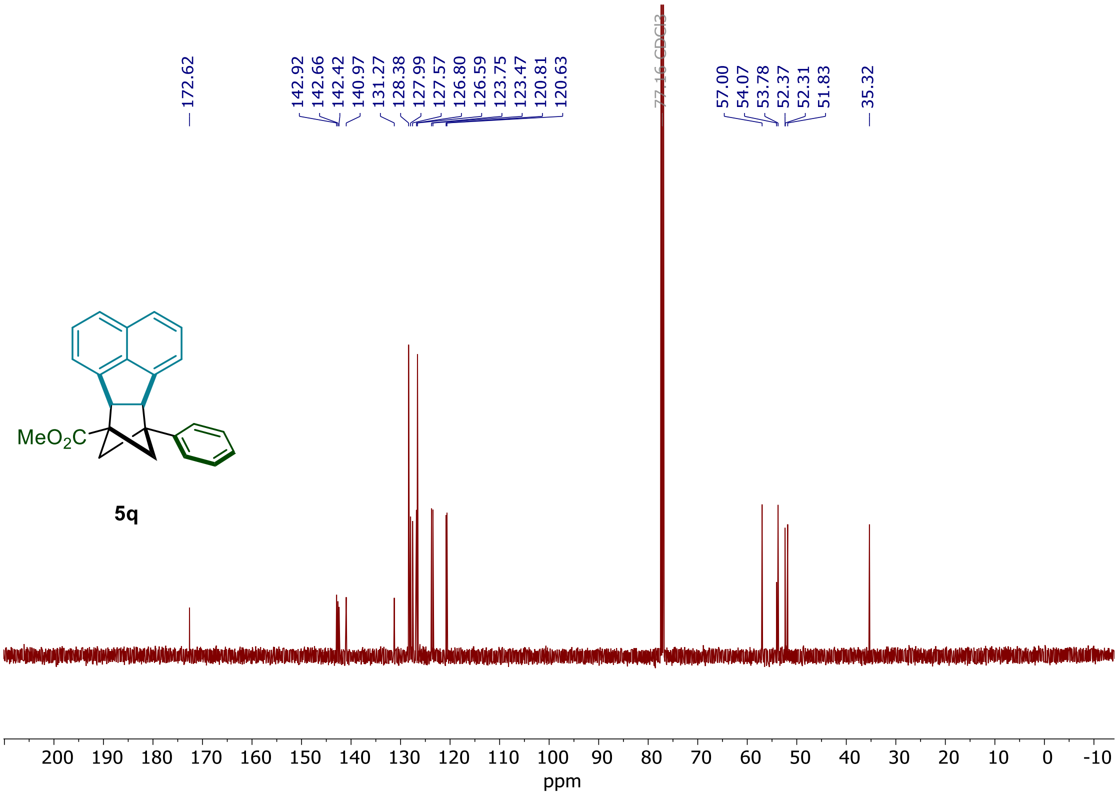

^{1}H NMR (500 MHz, $CDCl_3$) of **5r** (*see procedure*)

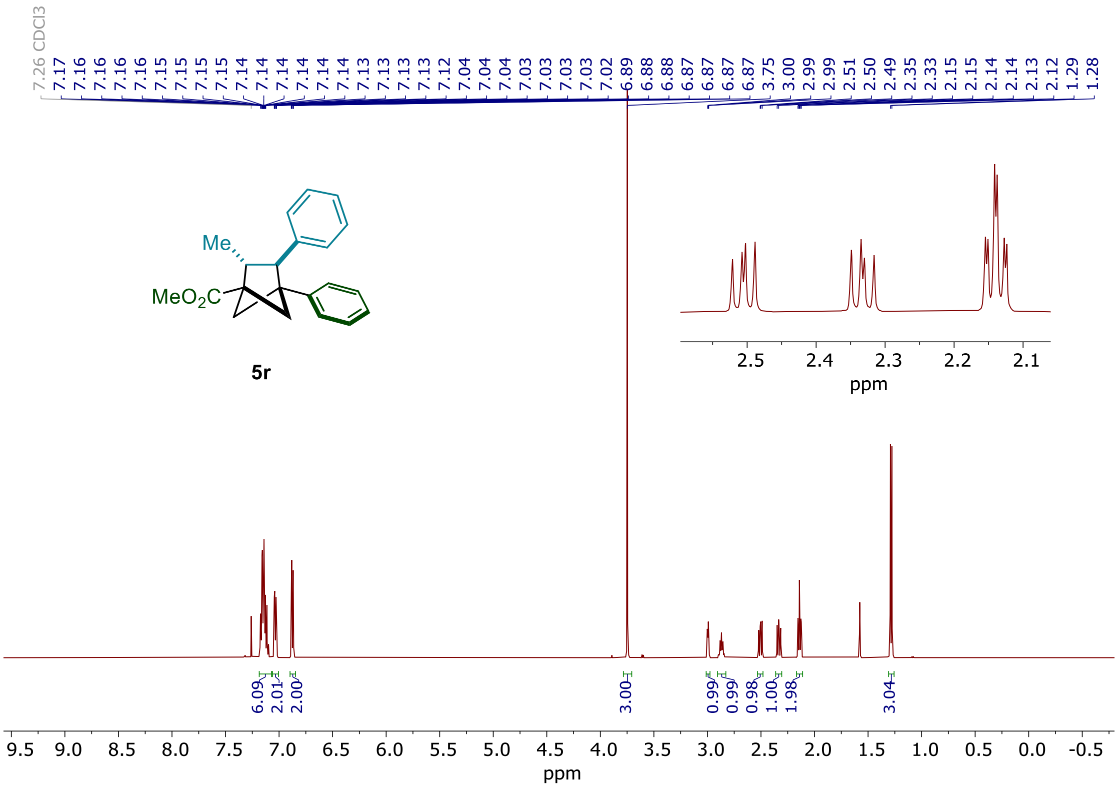

^{13}C NMR (126 MHz, $CDCl_3$) of **5r**

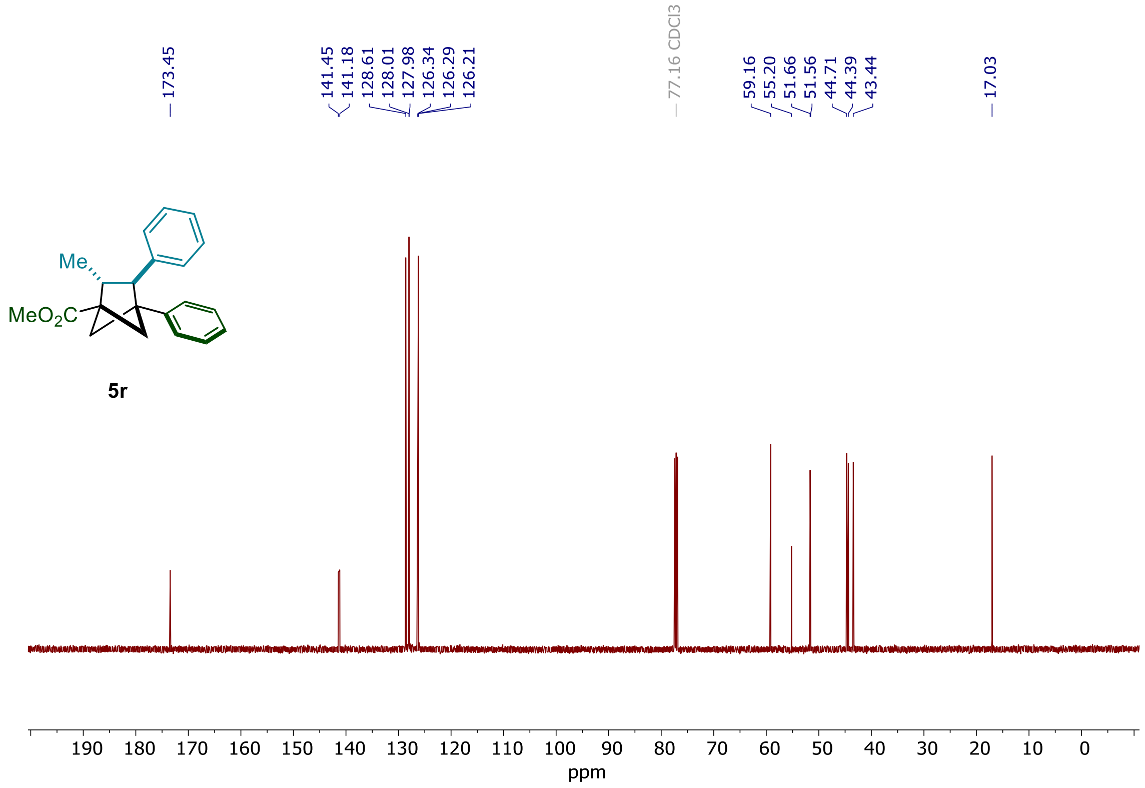

^{1}H NMR (500 MHz, $CDCl_3$) of **5s** (*see procedure*)

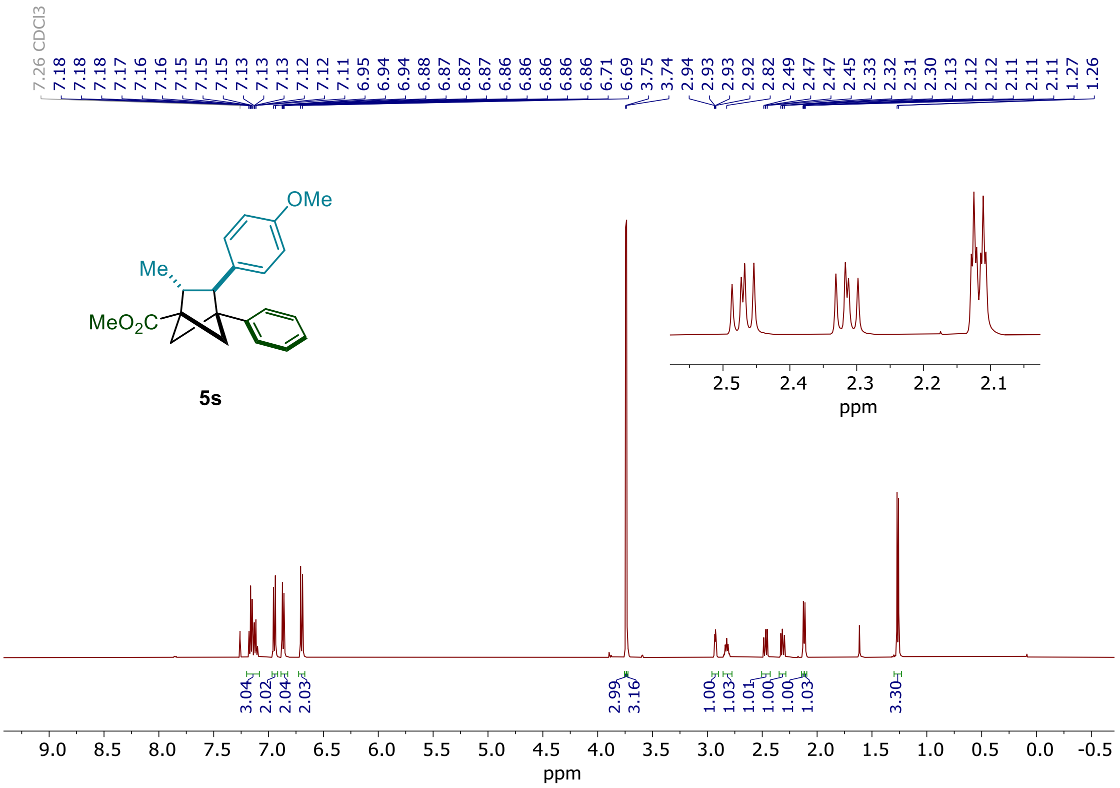

^{13}C NMR (126 MHz, $CDCl_3$) of **5s**

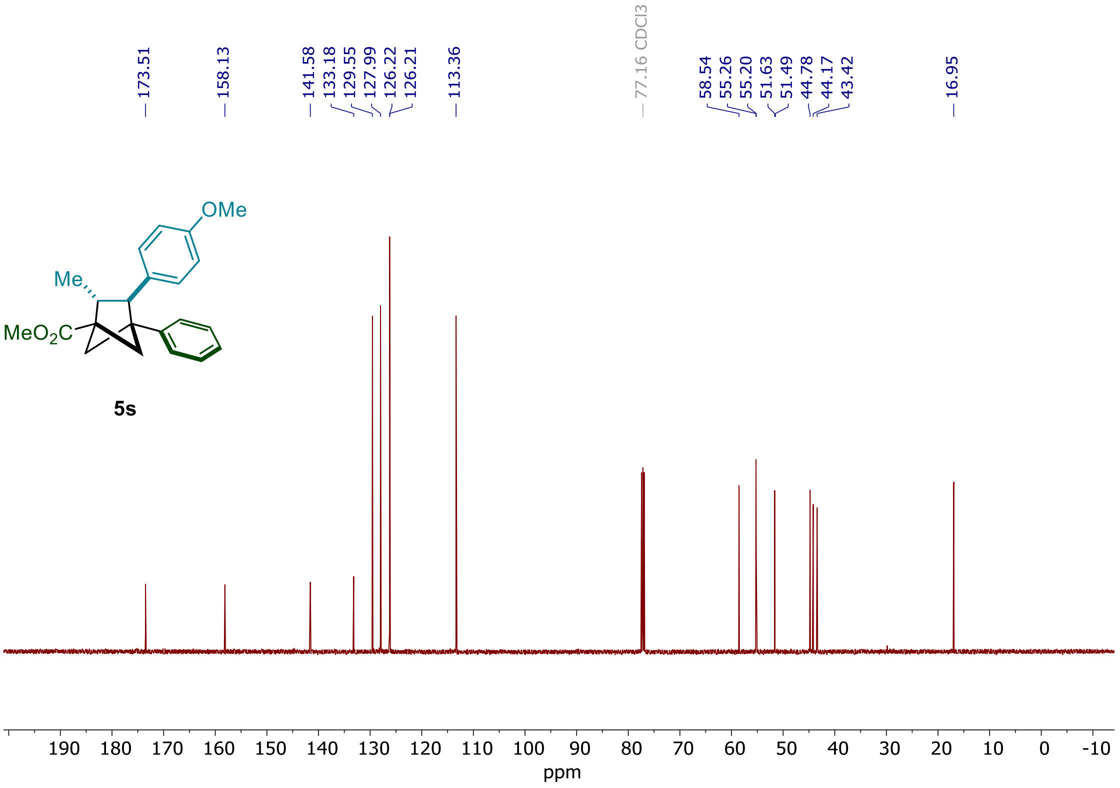

^{1}H NMR (500 MHz, $CDCl_3$) of **5u** (*see procedure*)

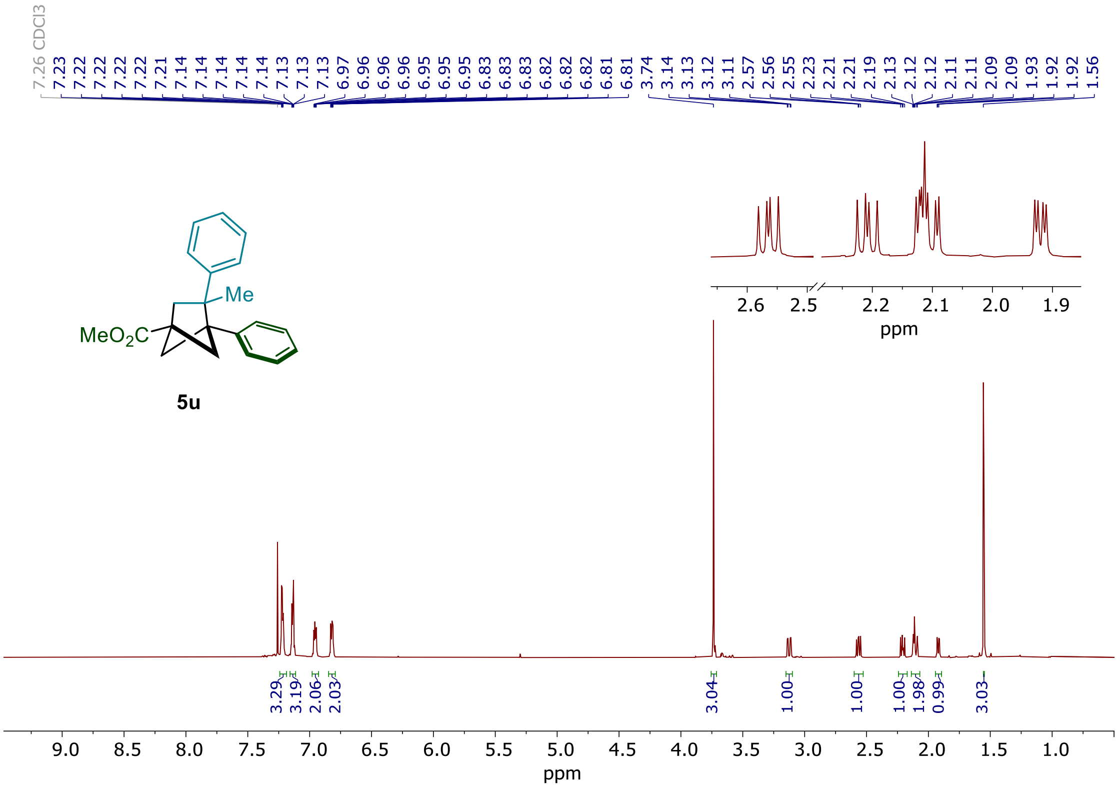

^{13}C NMR (126 MHz, $CDCl_3$) of **5u**

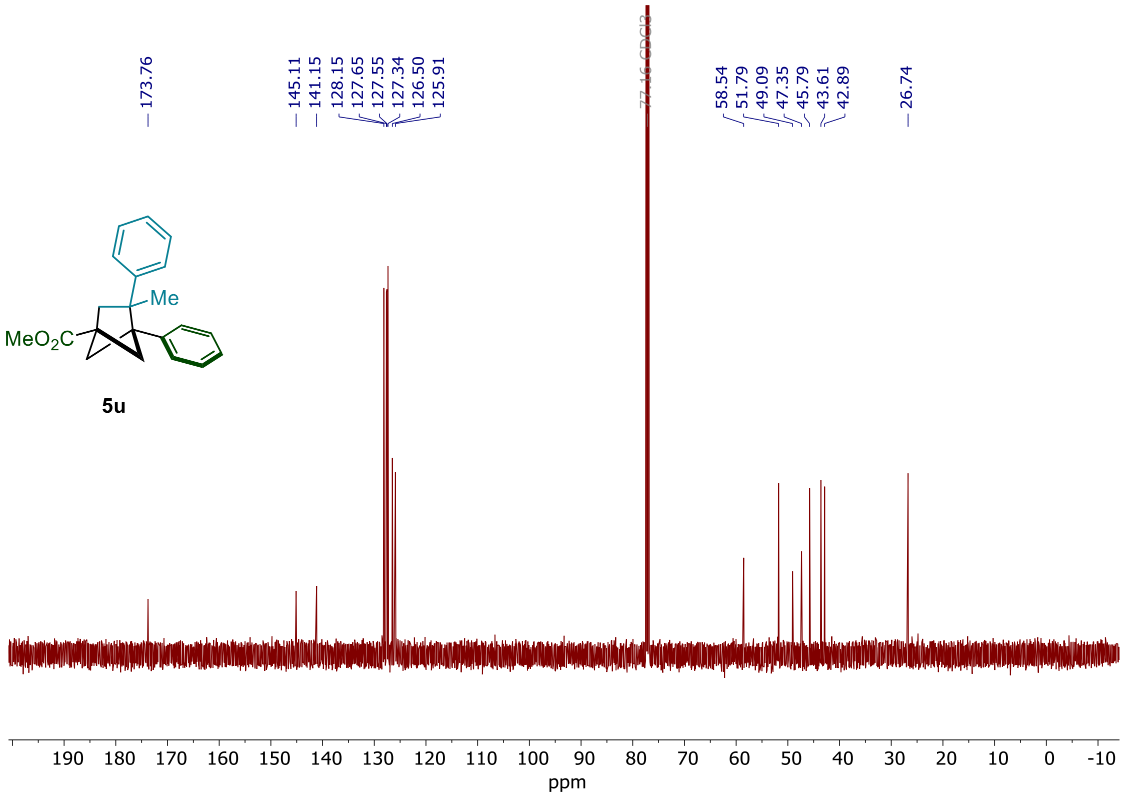

^{1}H NMR (500 MHz, $CDCl_3$) of **5v** (*see procedure*)

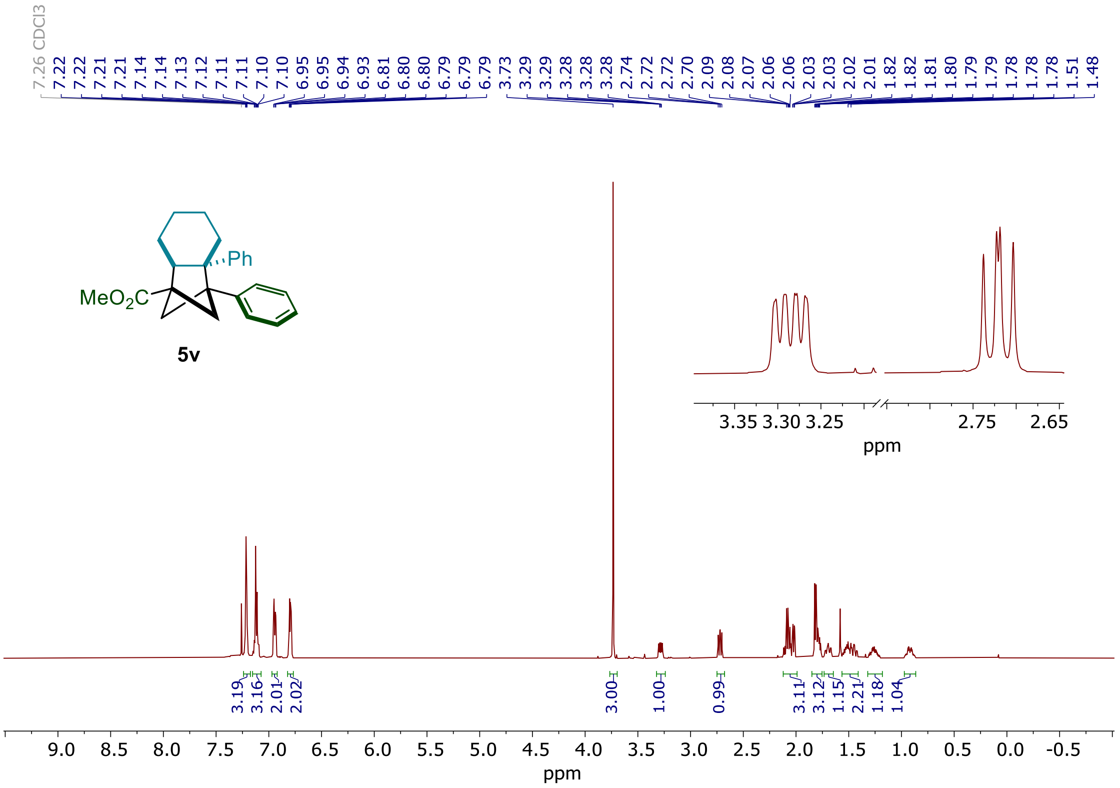

^{13}C NMR (126 MHz, $CDCl_3$) of **5v**

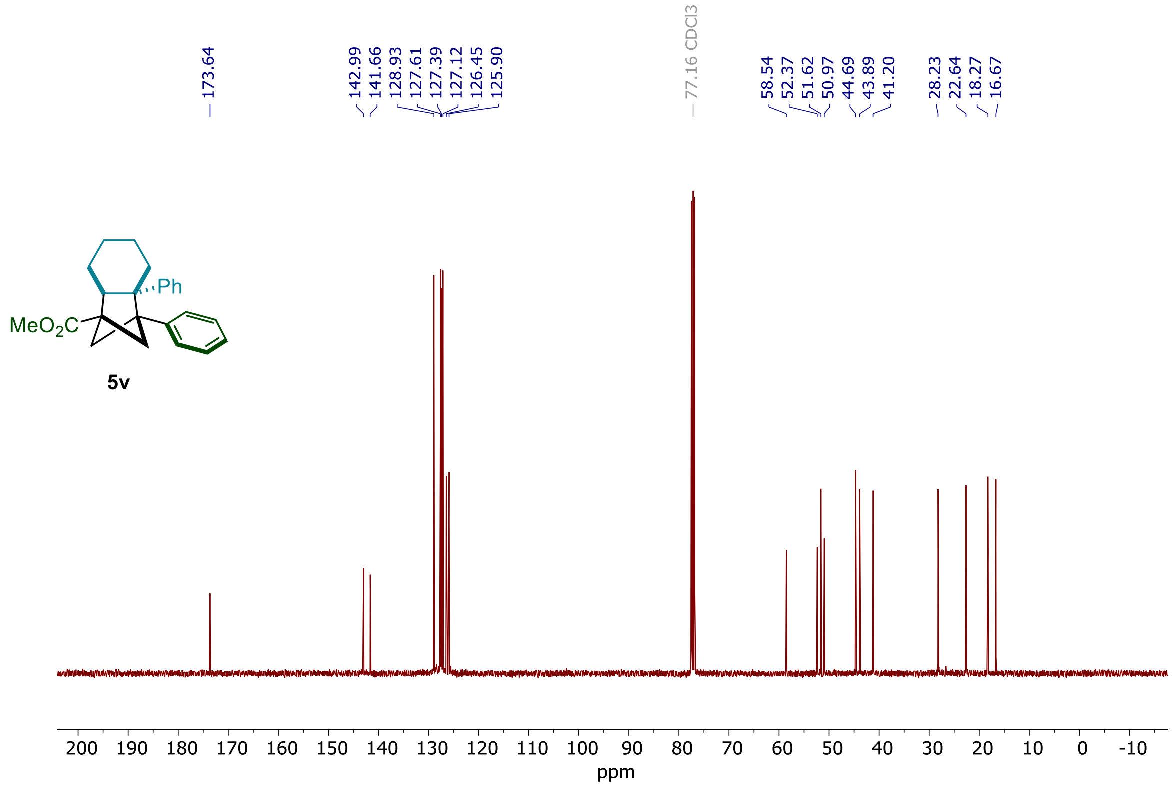

^{1}H NMR (500 MHz, $CDCl_3$) of **5w** (see procedure)

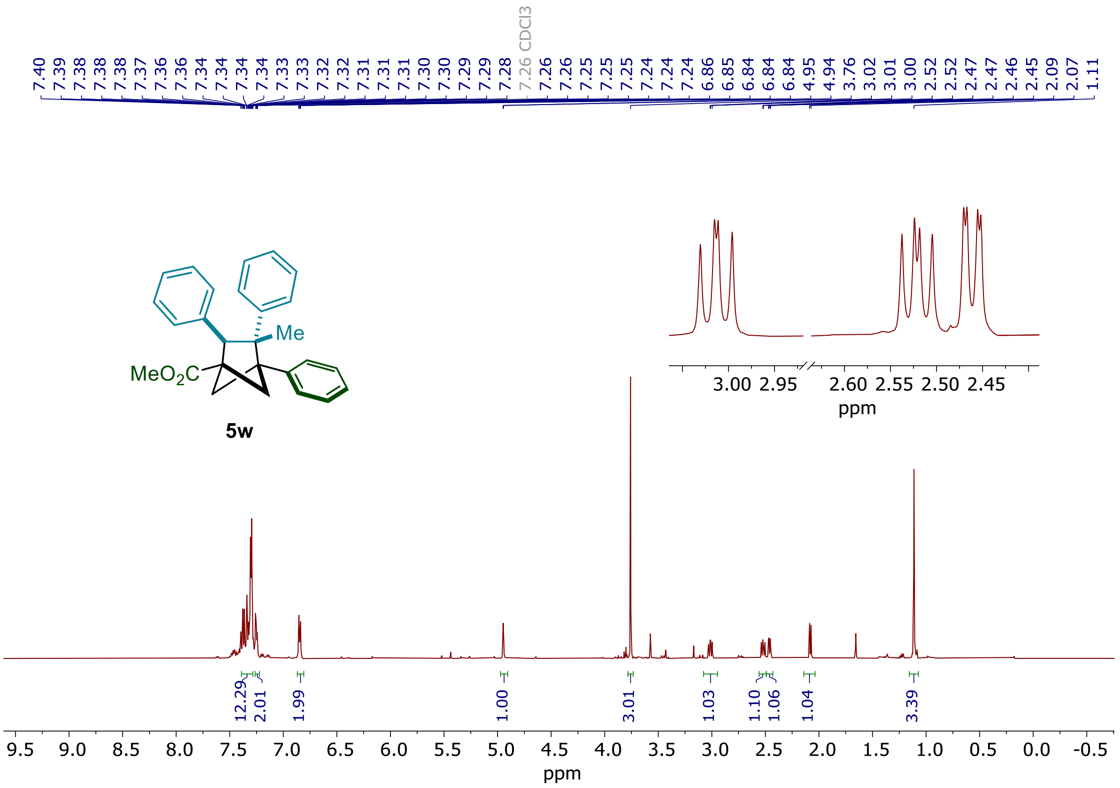

^{13}C NMR (126 MHz, $CDCl_3$) of **5w**

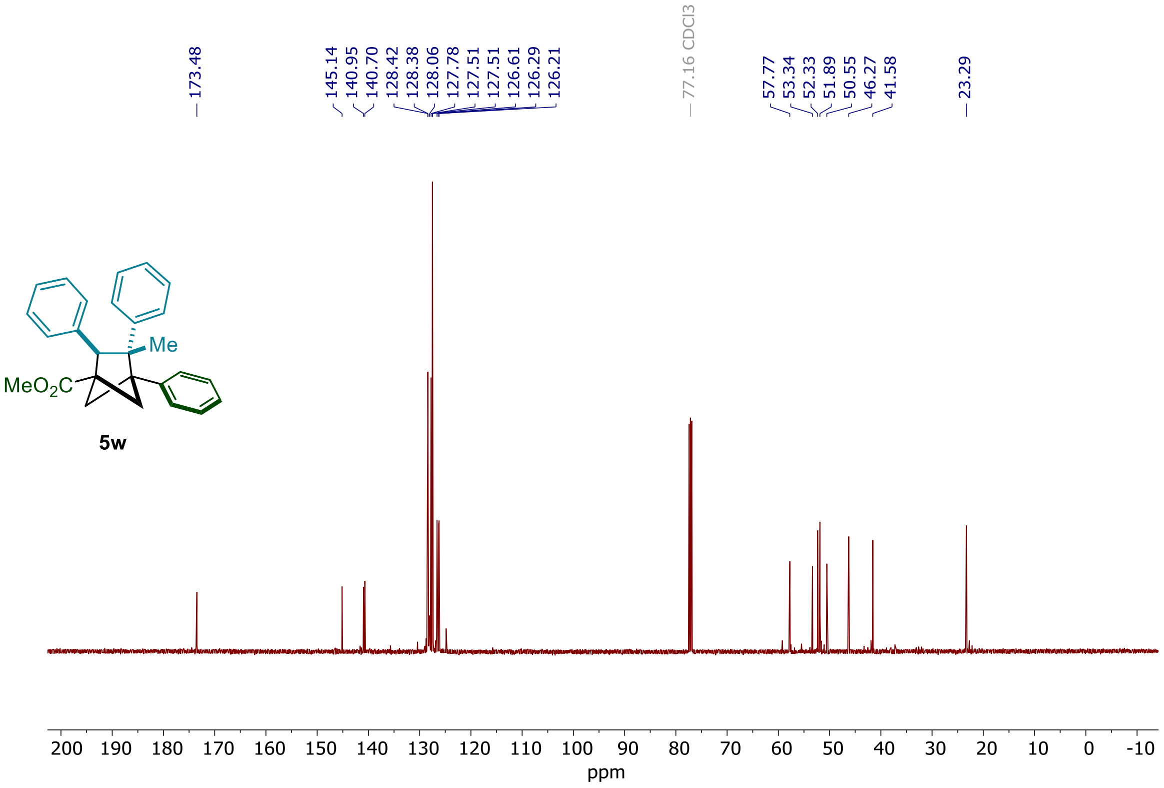

^{1}H NMR (500 MHz, $CDCl_3$) of **5y** (*see procedure*)

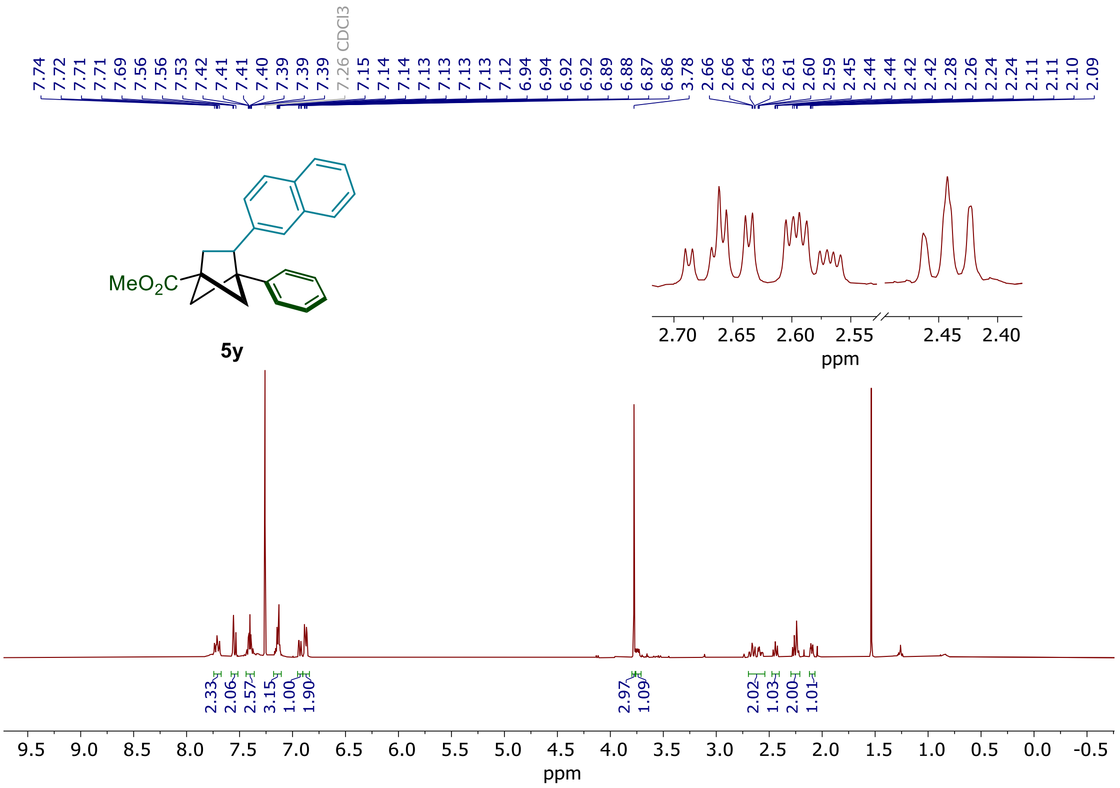

^{13}C NMR (126 MHz, $CDCl_3$) of **5y**

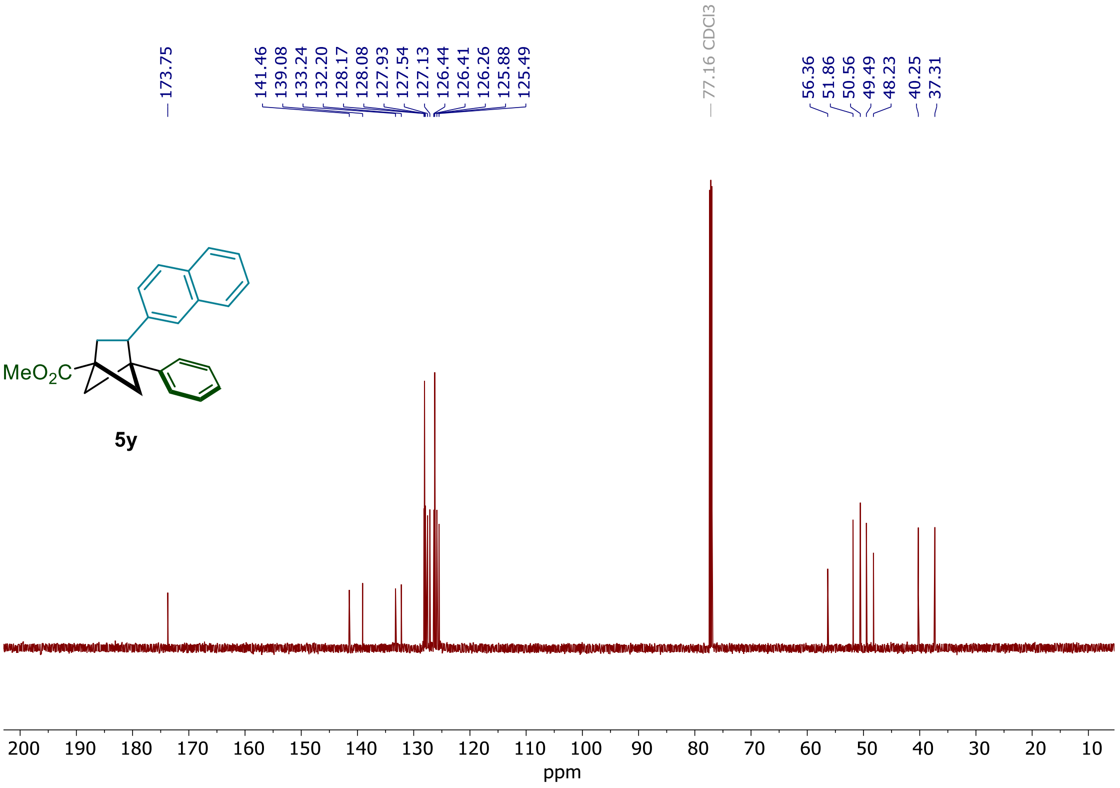

^{1}H NMR (500 MHz, $CDCl_3$) of **5z** (*see procedure*)

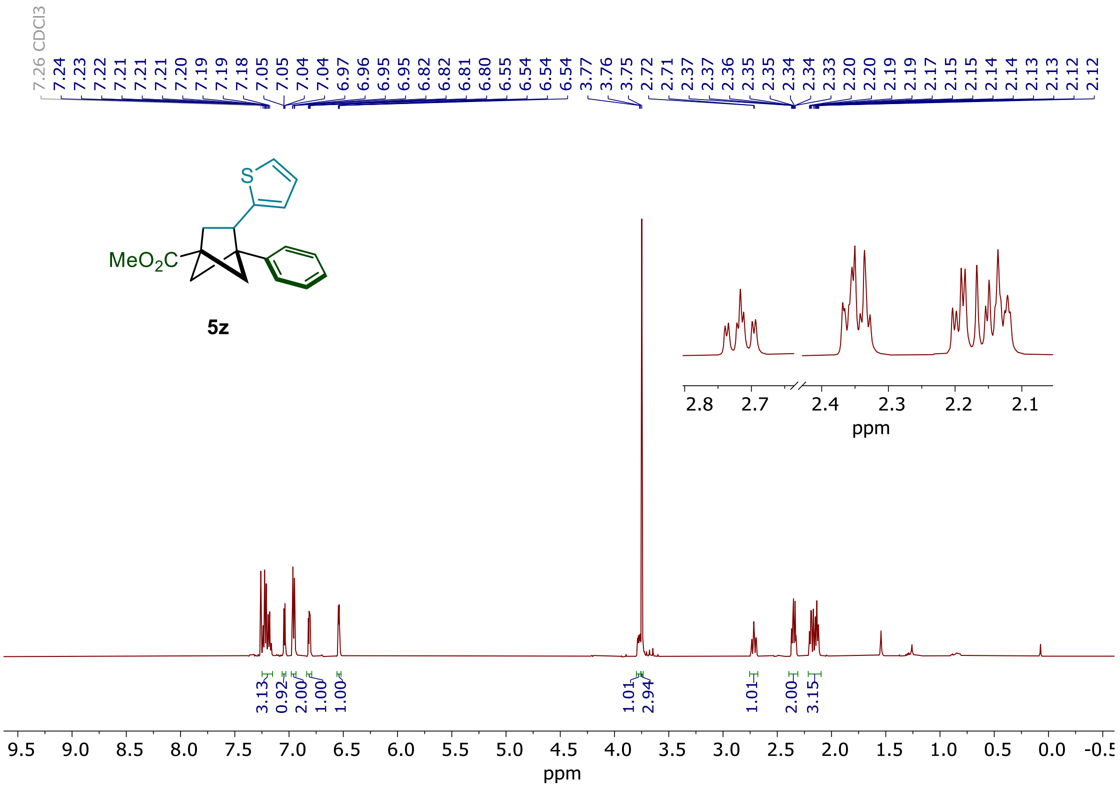

^{13}C NMR (126 MHz, $CDCl_3$) of **5z**

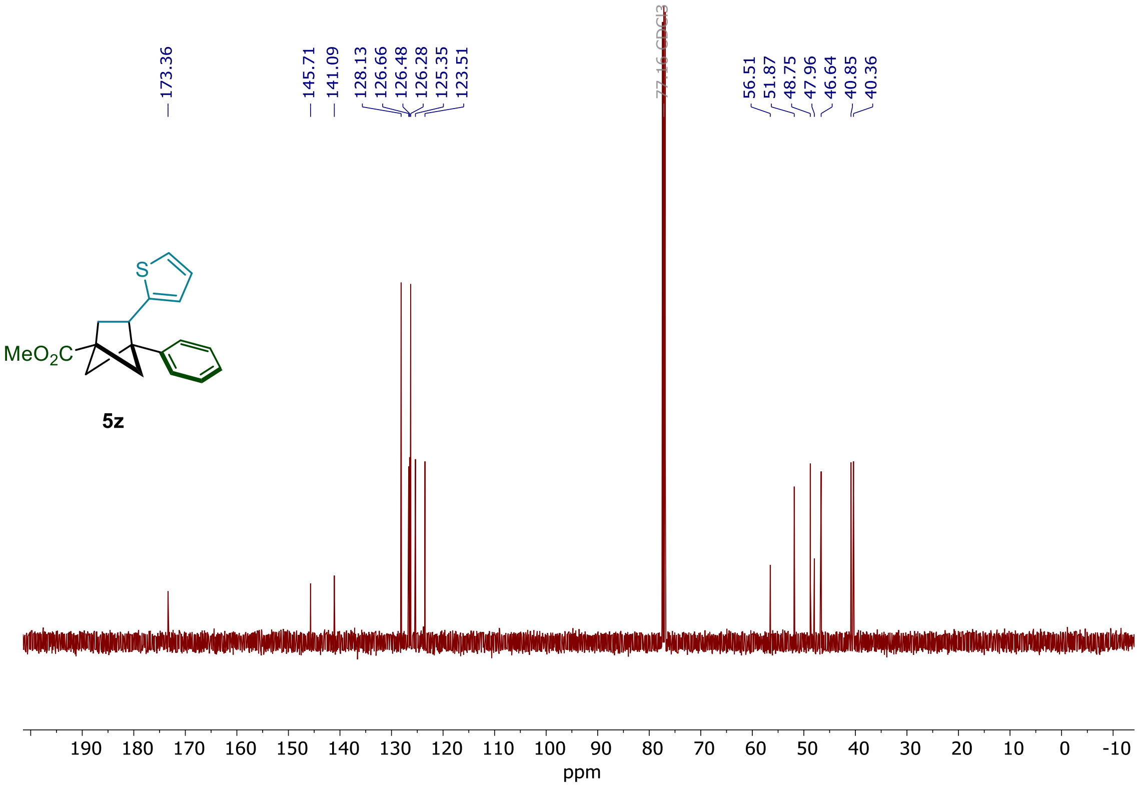

^{1}H NMR (500 MHz, $CDCl_3$) of **5aa** (*see procedure*)

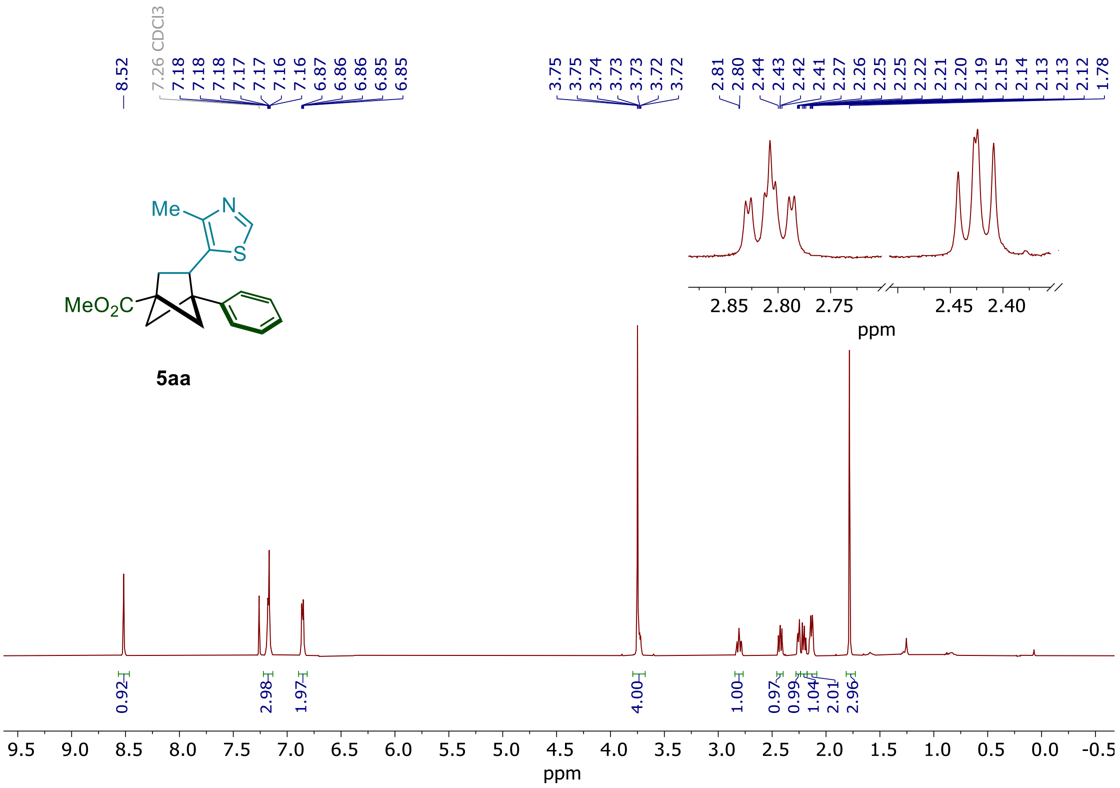

^{13}C NMR (126 MHz, $CDCl_3$) of **5aa**

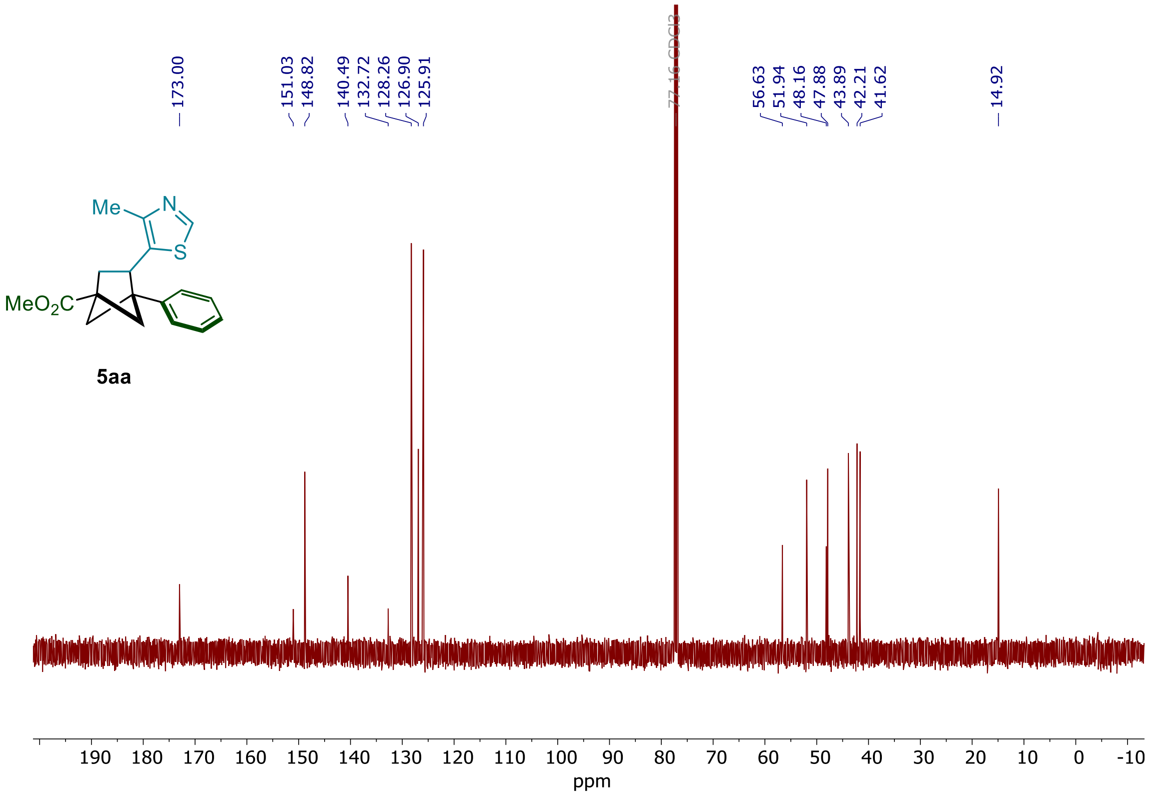

^{1}H NMR (400 MHz, $CDCl_3$) of **5ac** (see procedure)

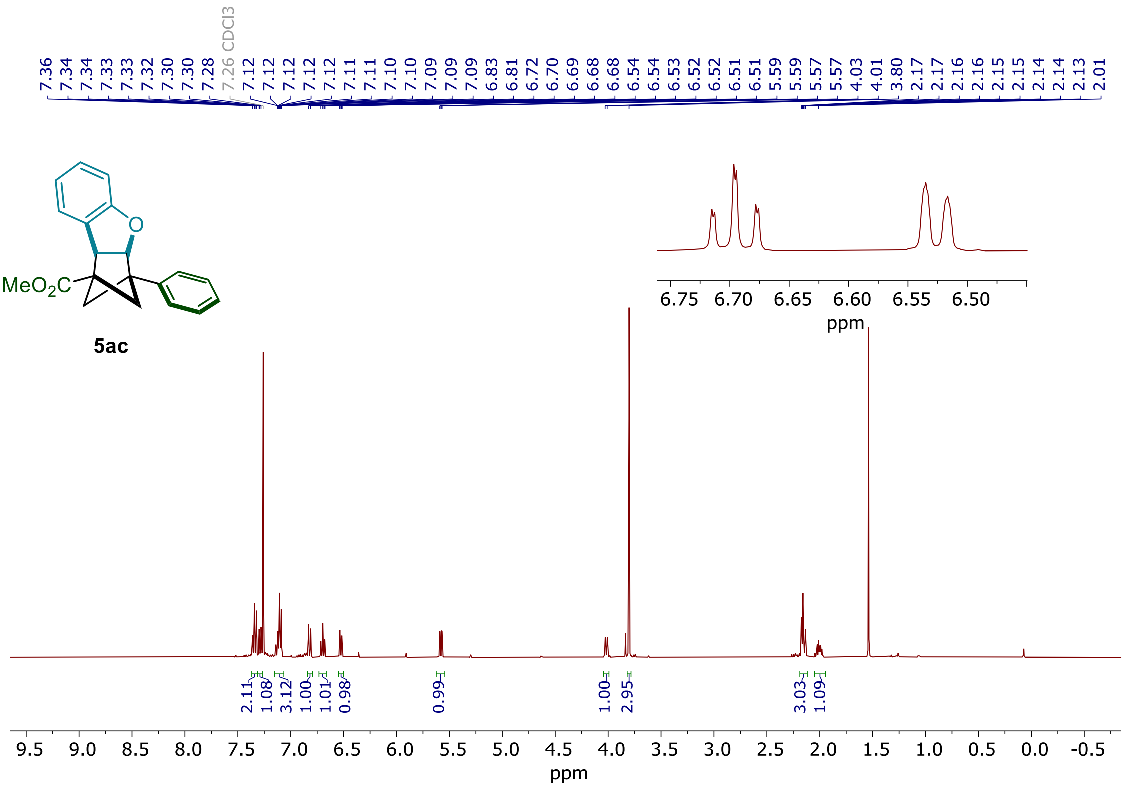

^{13}C NMR (126 MHz, $CDCl_3$) of **5ac**

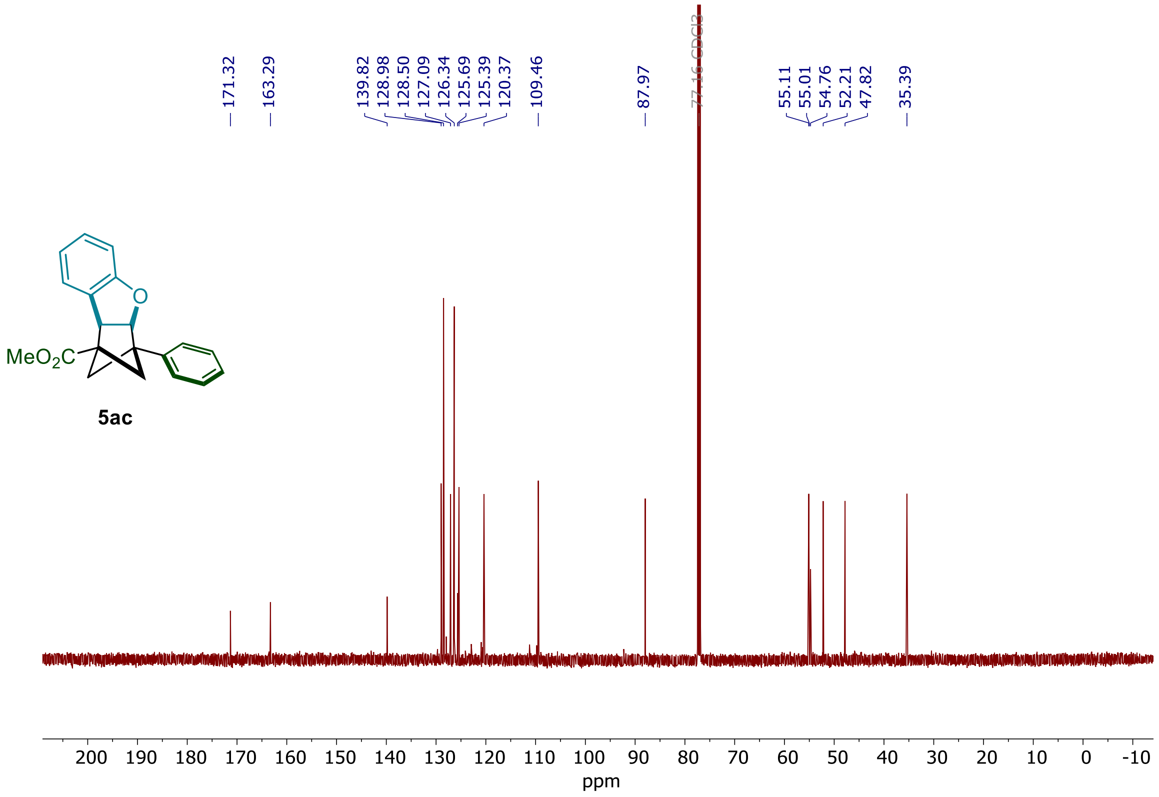

^{1}H NMR (400 MHz, $CDCl_3$) of **5ad** (see procedure)

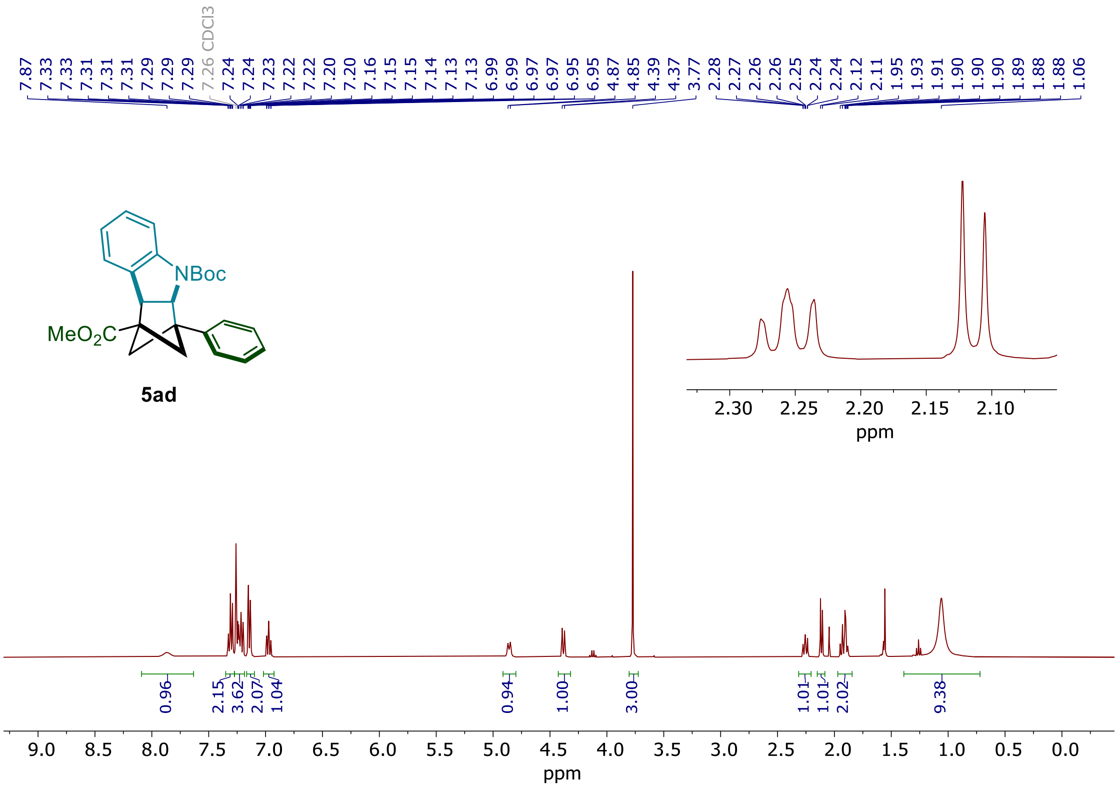

^{13}C NMR (126 MHz, $CDCl_3$) of **5ad**

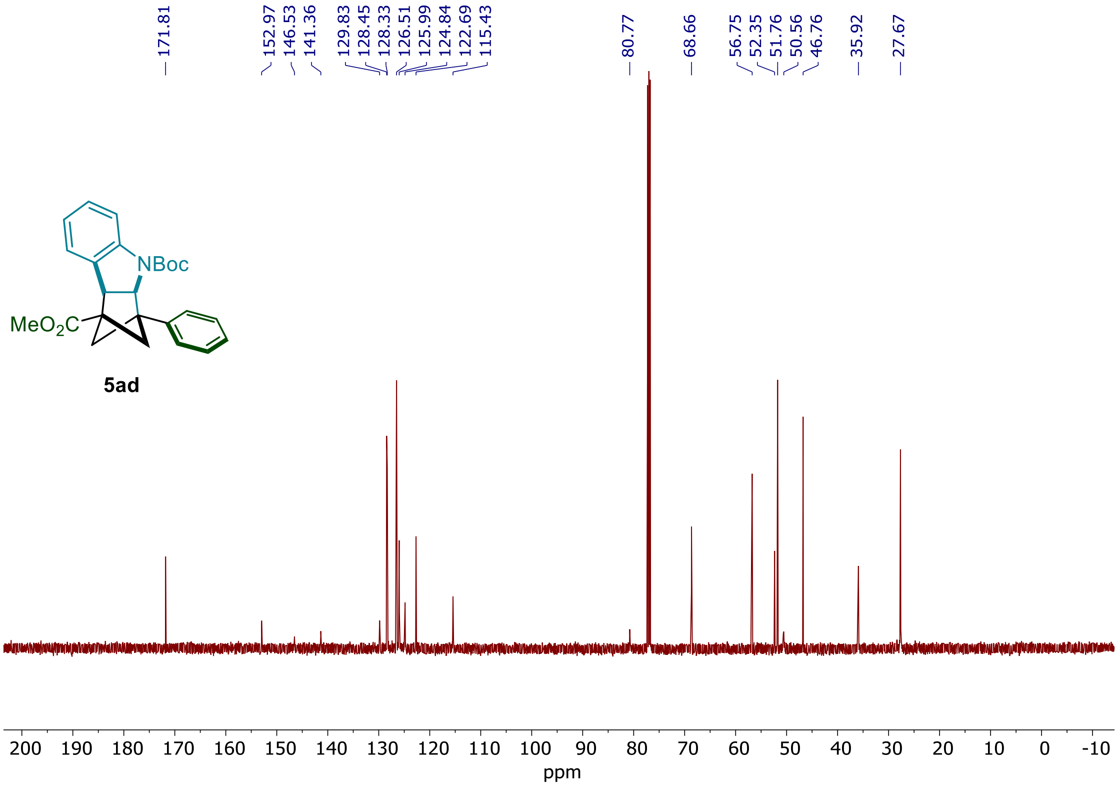

^{1}H NMR (500 MHz, $CDCl_3$) of **5ad'** (*see procedure*)

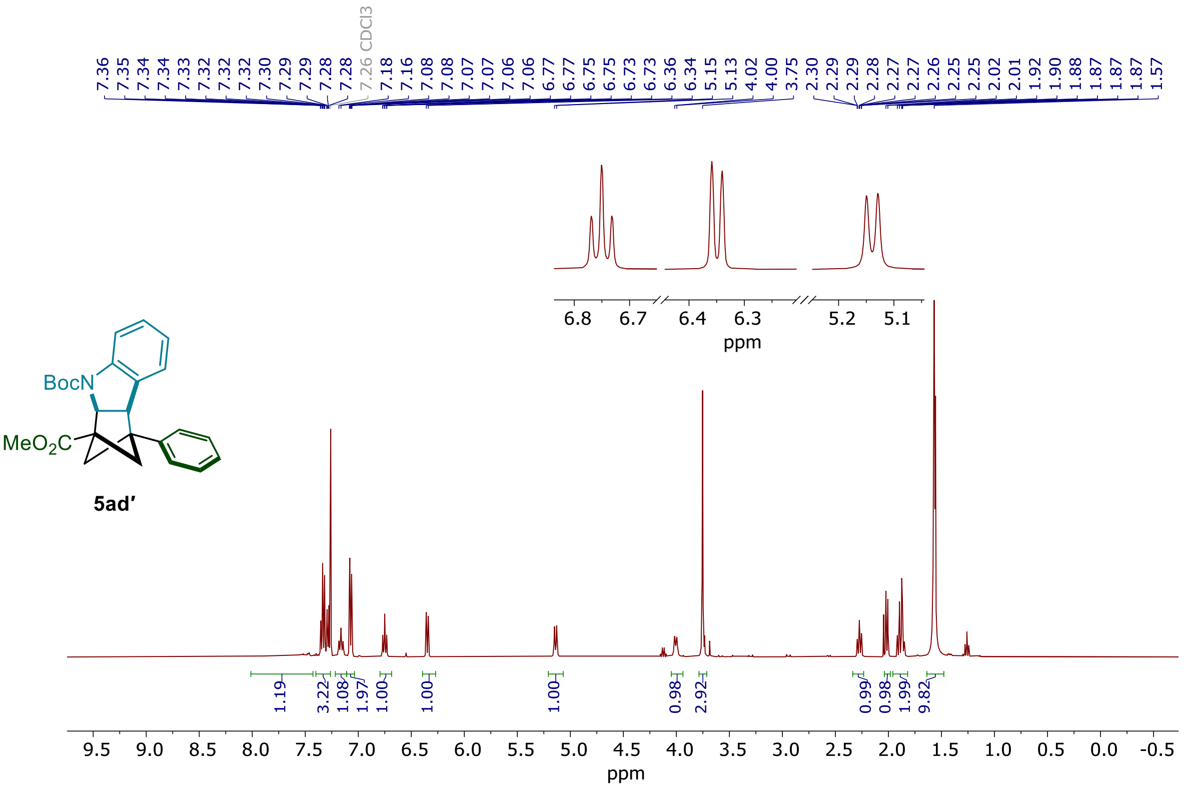

^{13}C NMR (126 MHz, $CDCl_3$) of **5ad'**

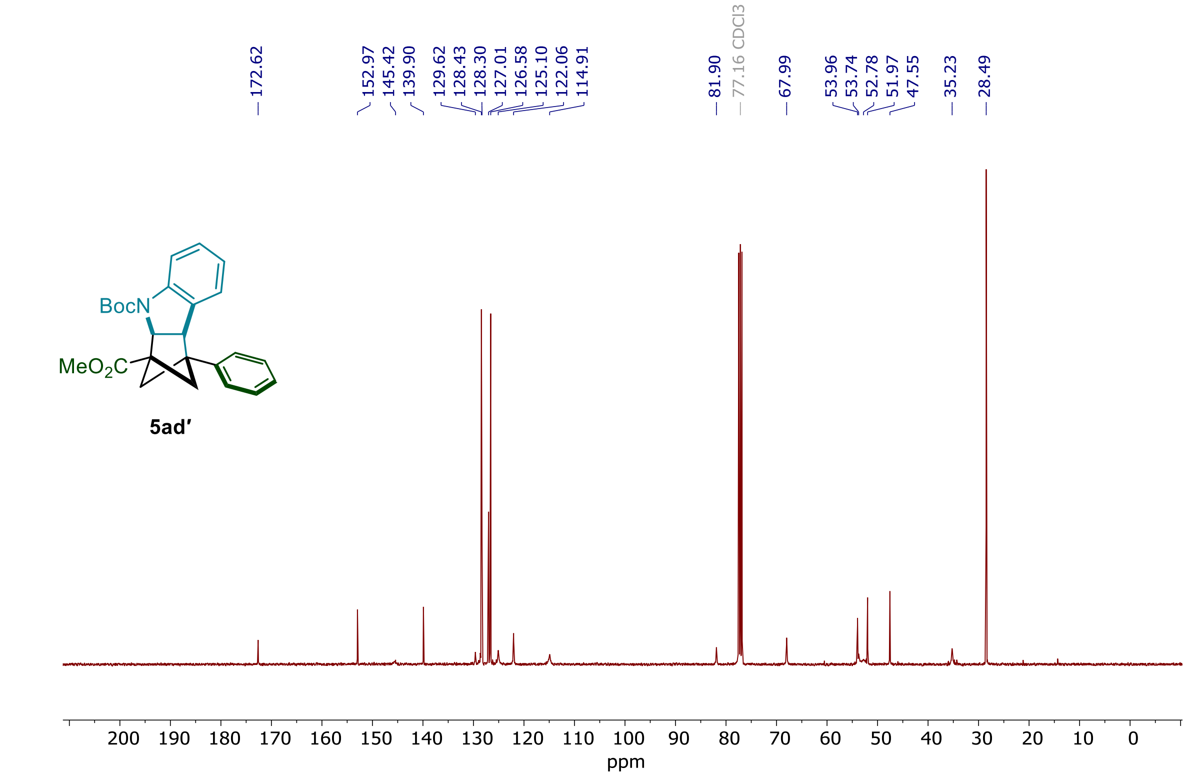

^{1}H NMR (400 MHz, $CDCl_3$) of **5ae** (*see procedure*)

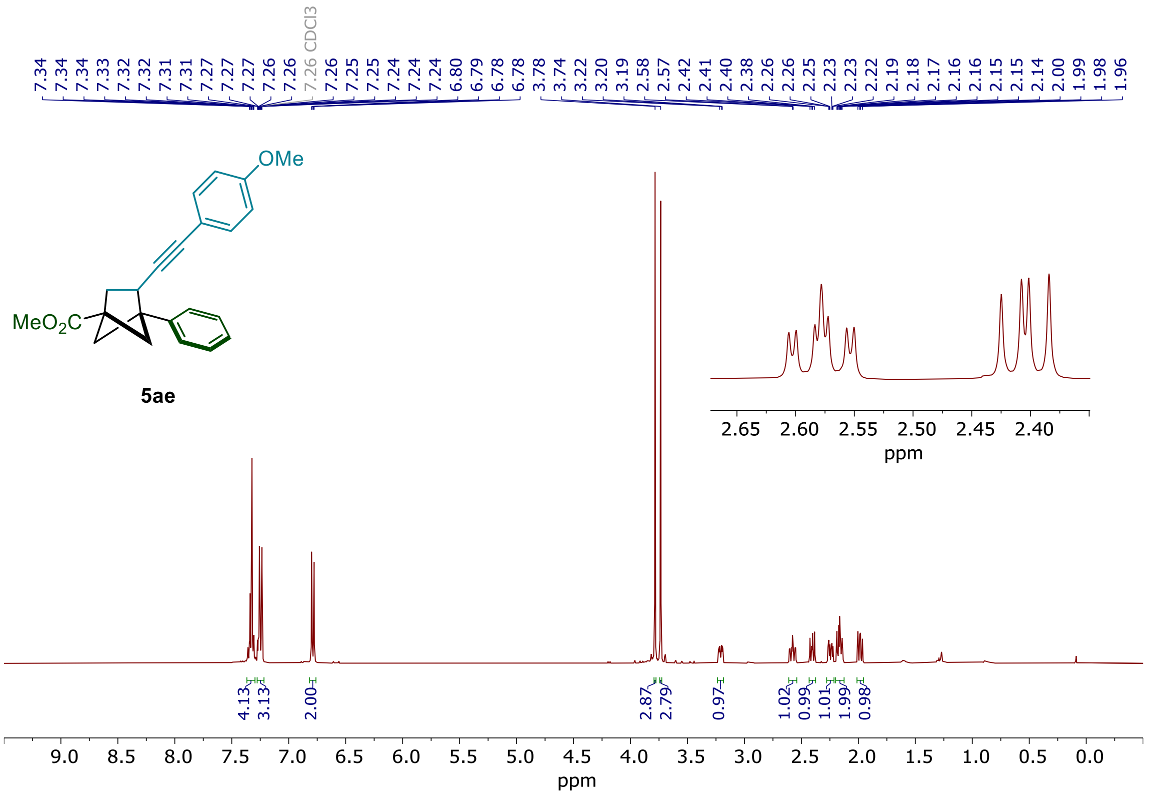

^{13}C NMR (101 MHz, $CDCl_3$) of **5ae**

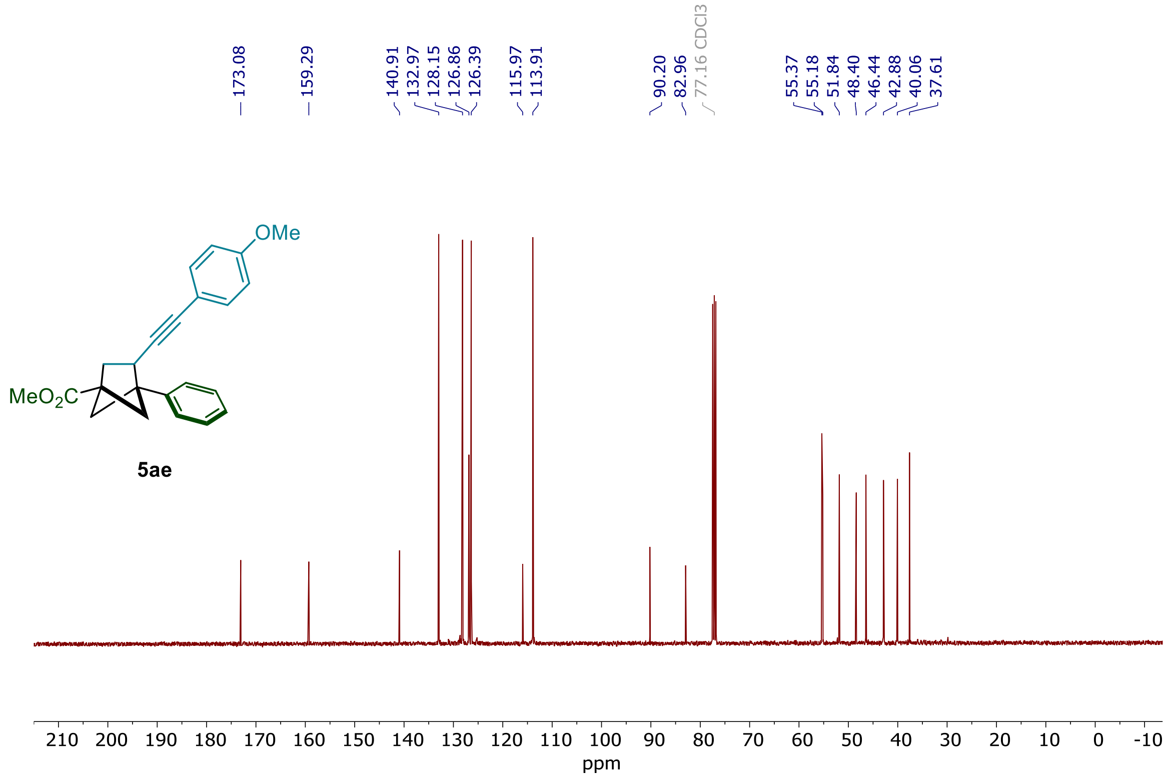

^{1}H NMR (400 MHz, $CDCl_3$) of **5af** (*see procedure*)

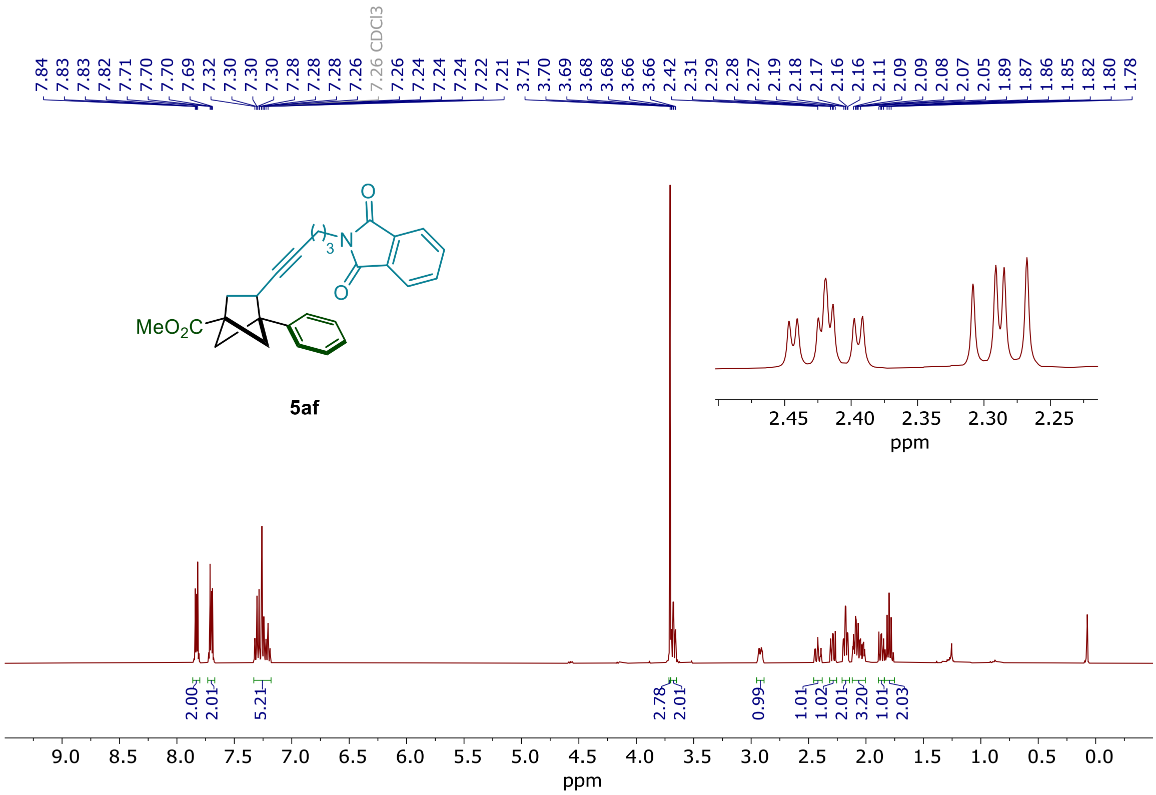

^{13}C NMR (101 MHz, $CDCl_3$) of **5af**

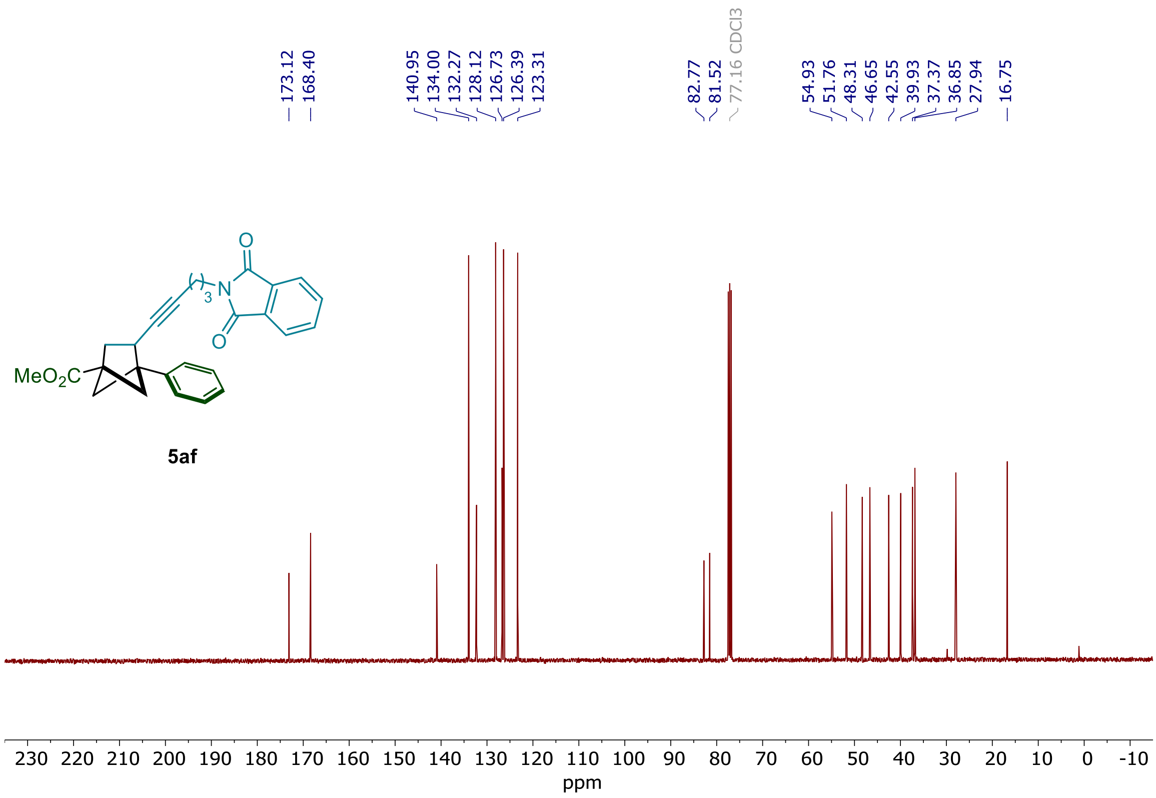

^{1}H NMR (500 MHz, $CDCl_3$) of **5ag** (*see procedure*)

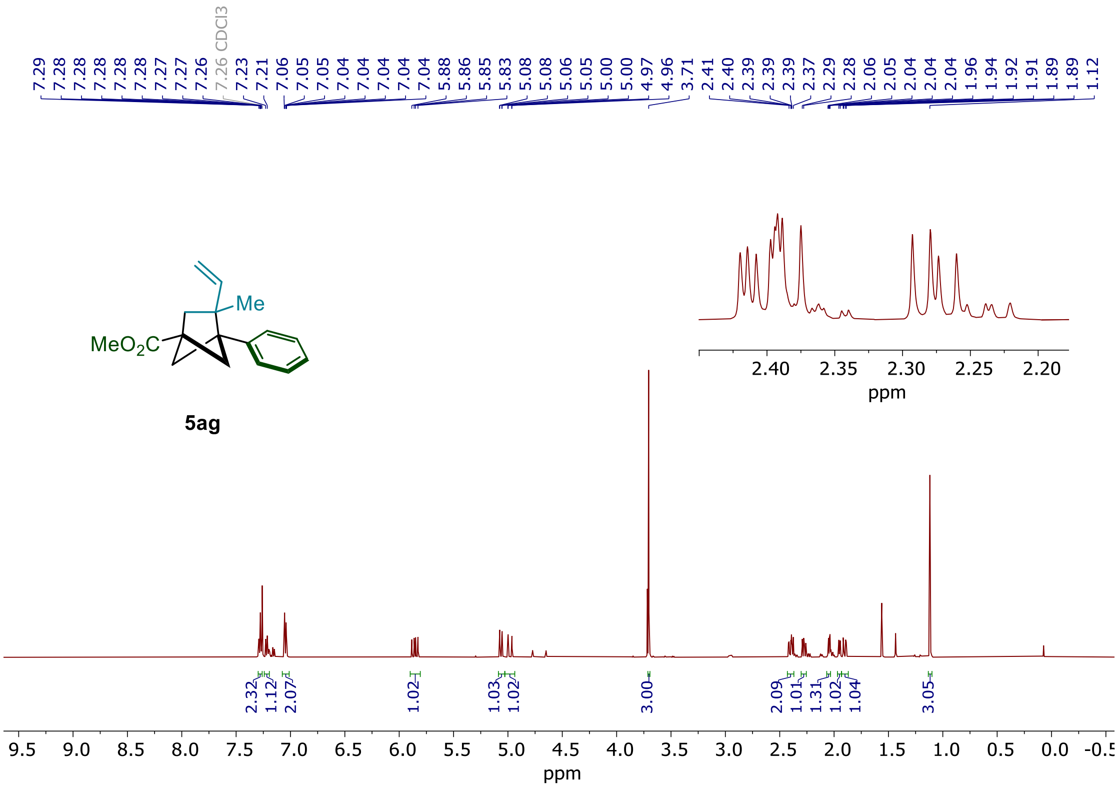

^{13}C NMR (126 MHz, $CDCl_3$) of **5ag**

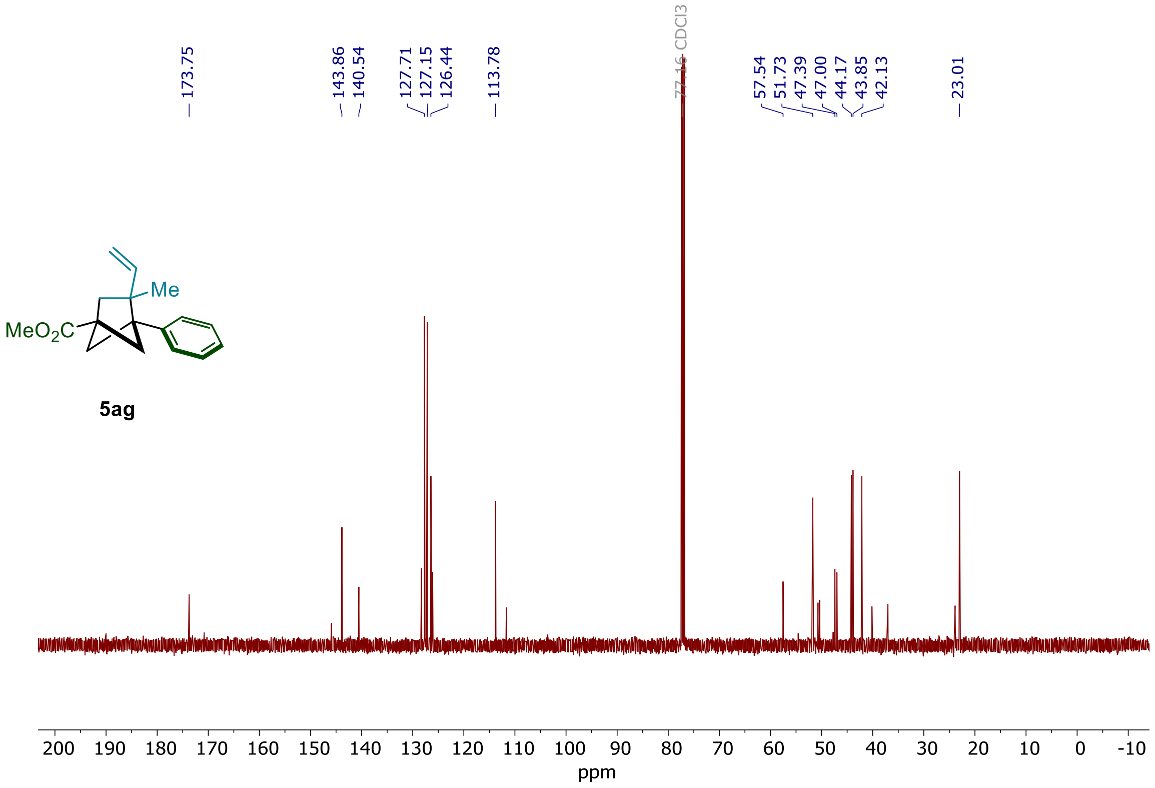

^{1}H NMR (500 MHz, $CDCl_3$) of **5ah** (*see procedure*)

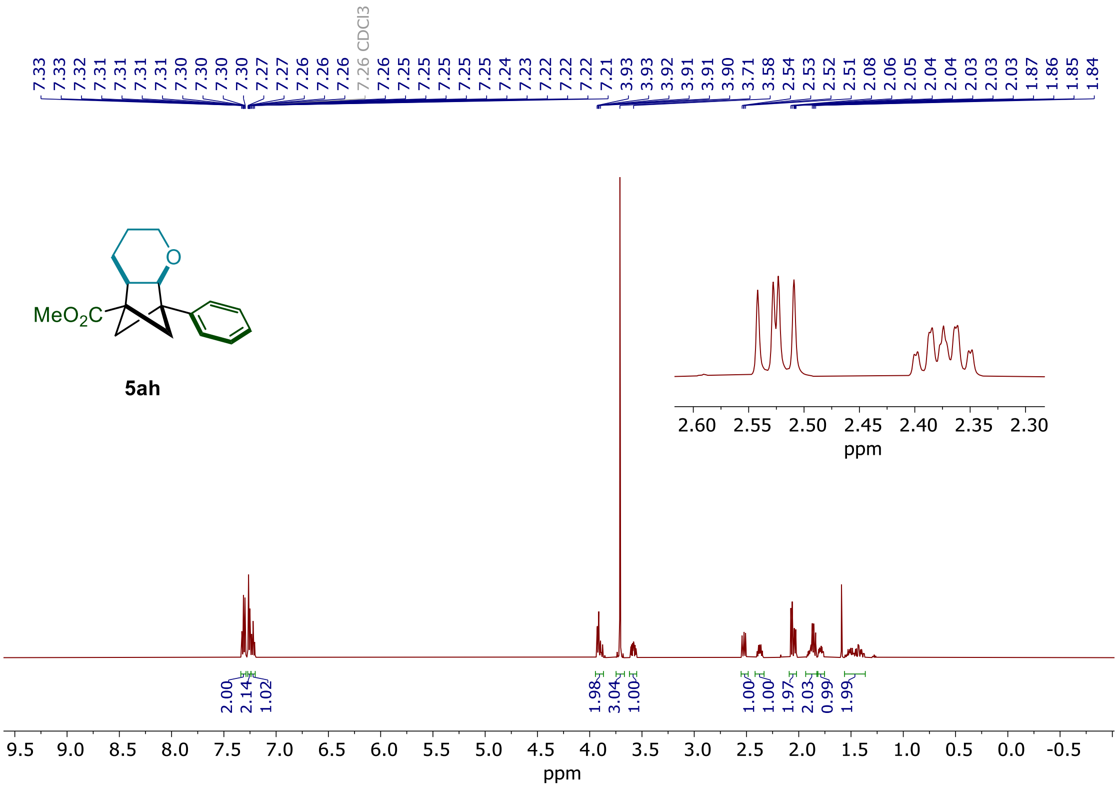

^{13}C NMR (126 MHz, $CDCl_3$) of **5ah**

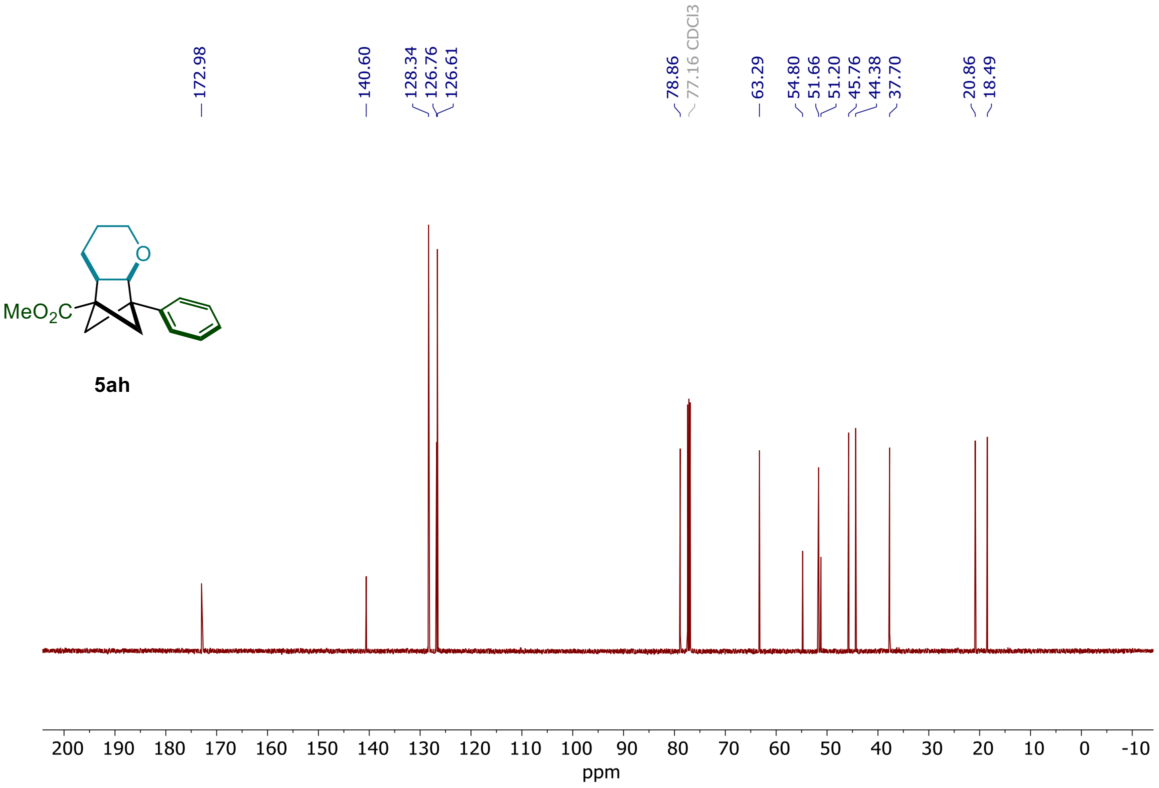

^{1}H NMR (500 MHz, $CDCl_3$) of **5ai** (*see procedure*)

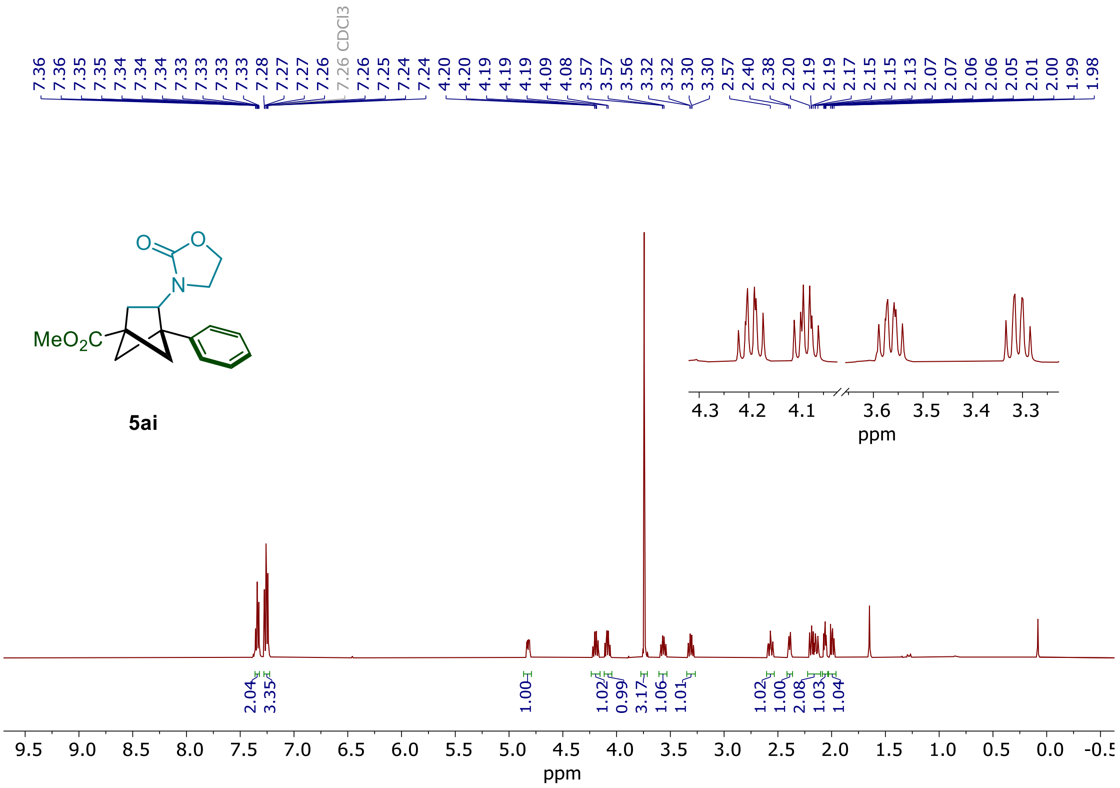

^{13}C NMR (126 MHz, $CDCl_3$) of **5ai**

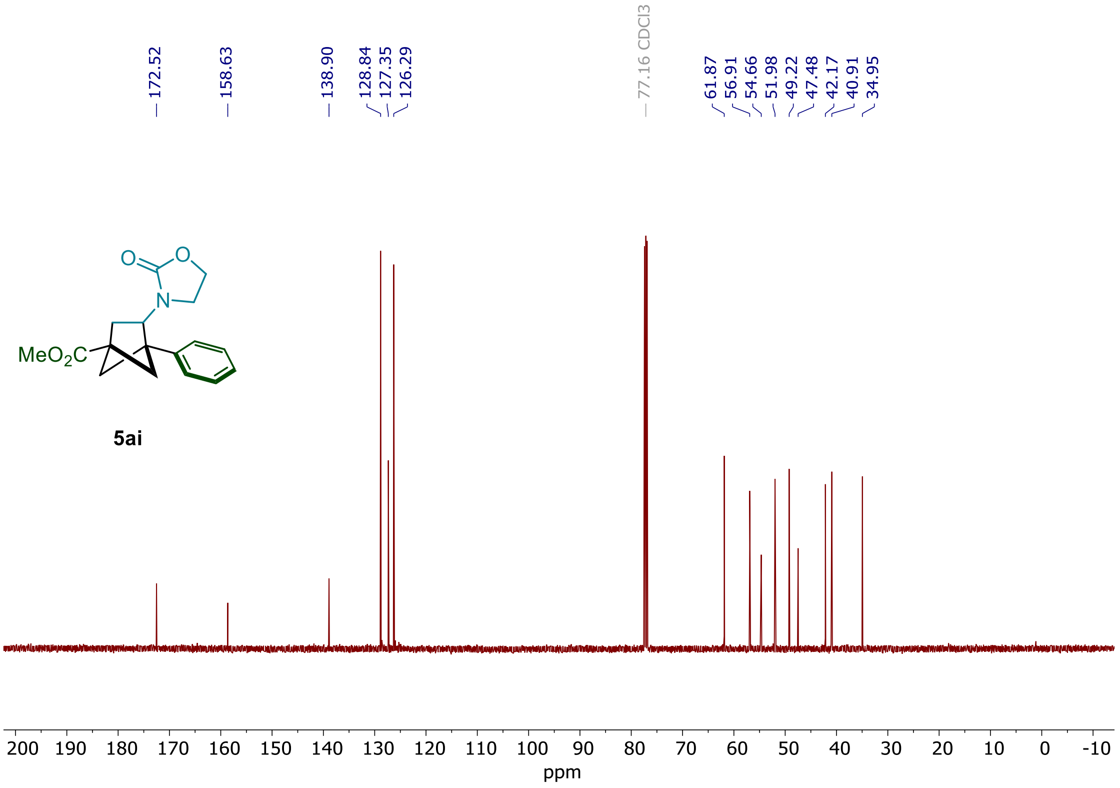

^{1}H NMR (600 MHz, $CDCl_3$) of **5ak** (*see procedure*)

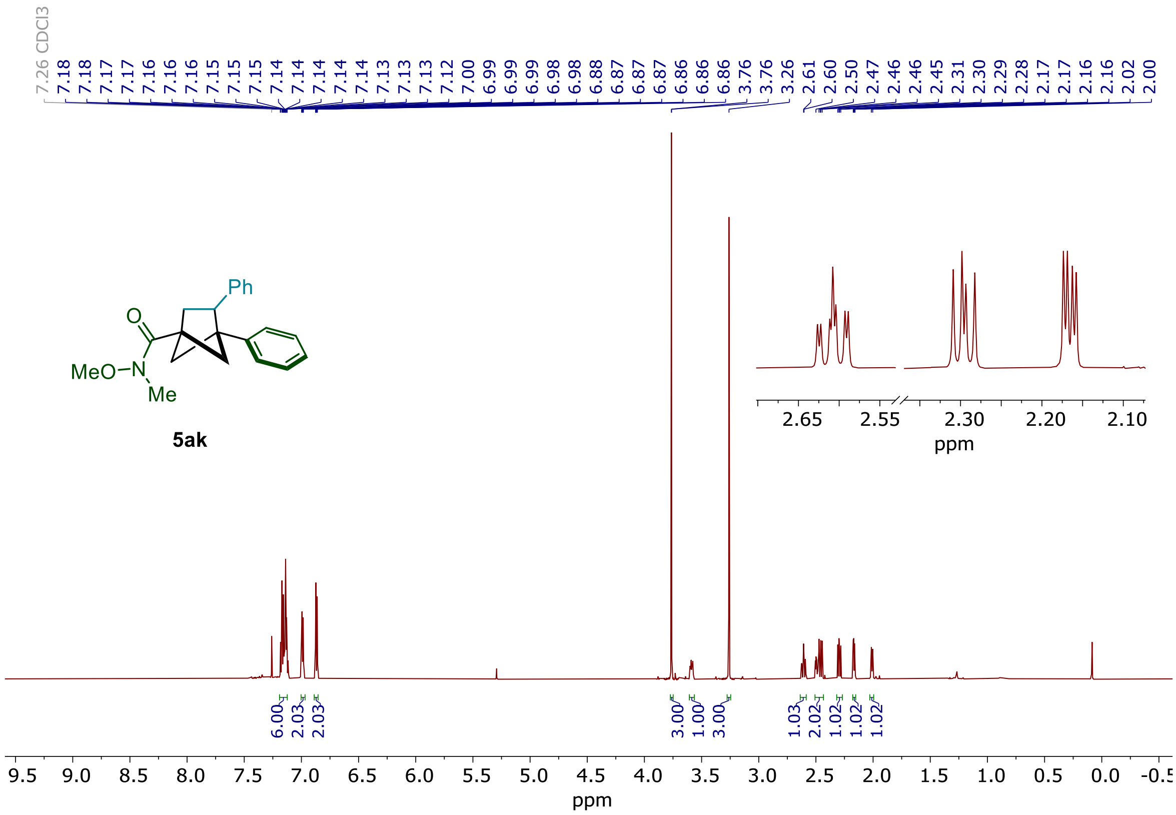

^{13}C NMR (151 MHz, $CDCl_3$) of **5ak**

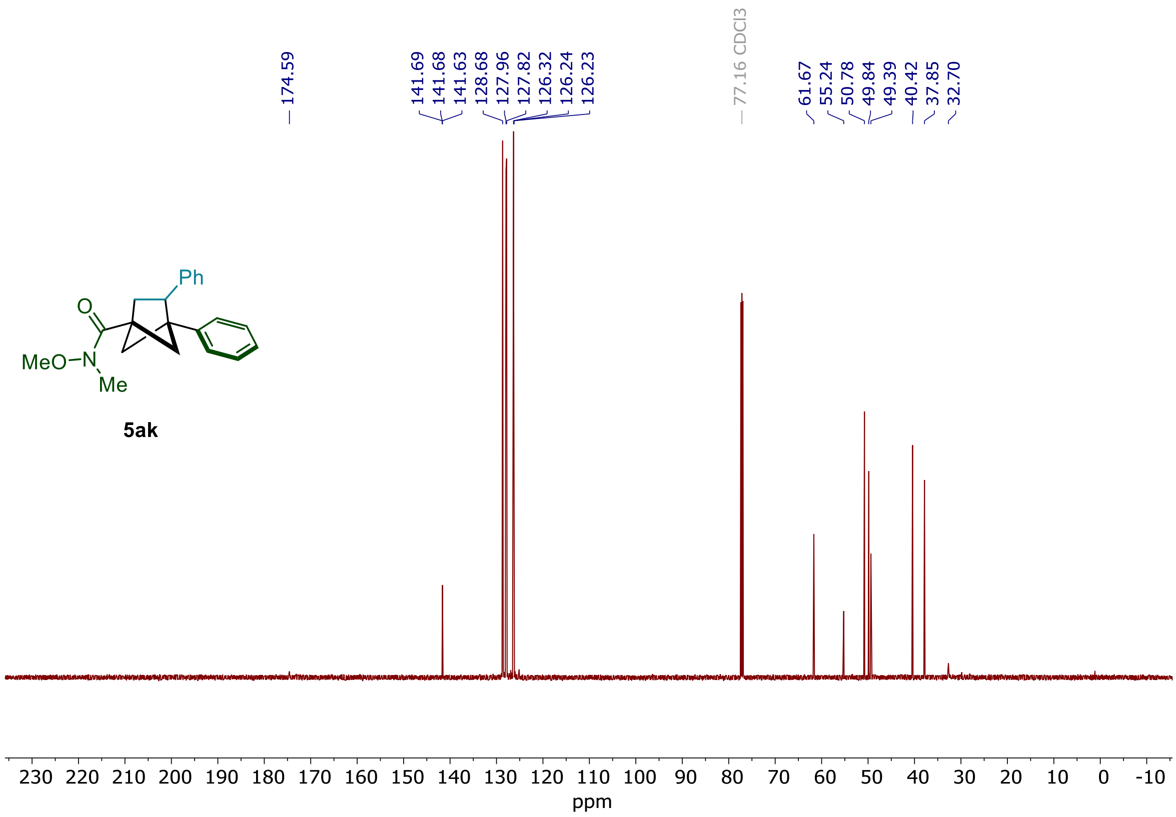

^{1}H NMR (500 MHz, $CDCl_3$) of **5al** (*see procedure*)

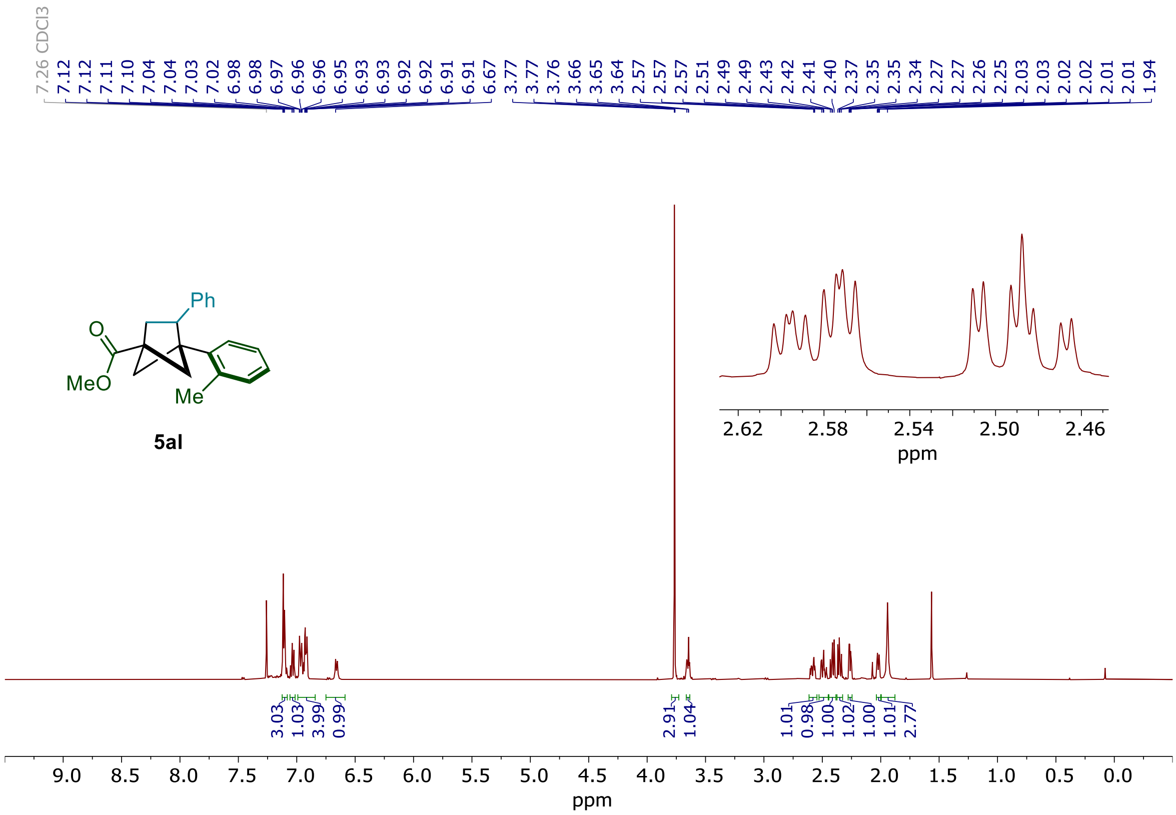

^{13}C NMR (101 MHz, $CDCl_3$) of **5al**

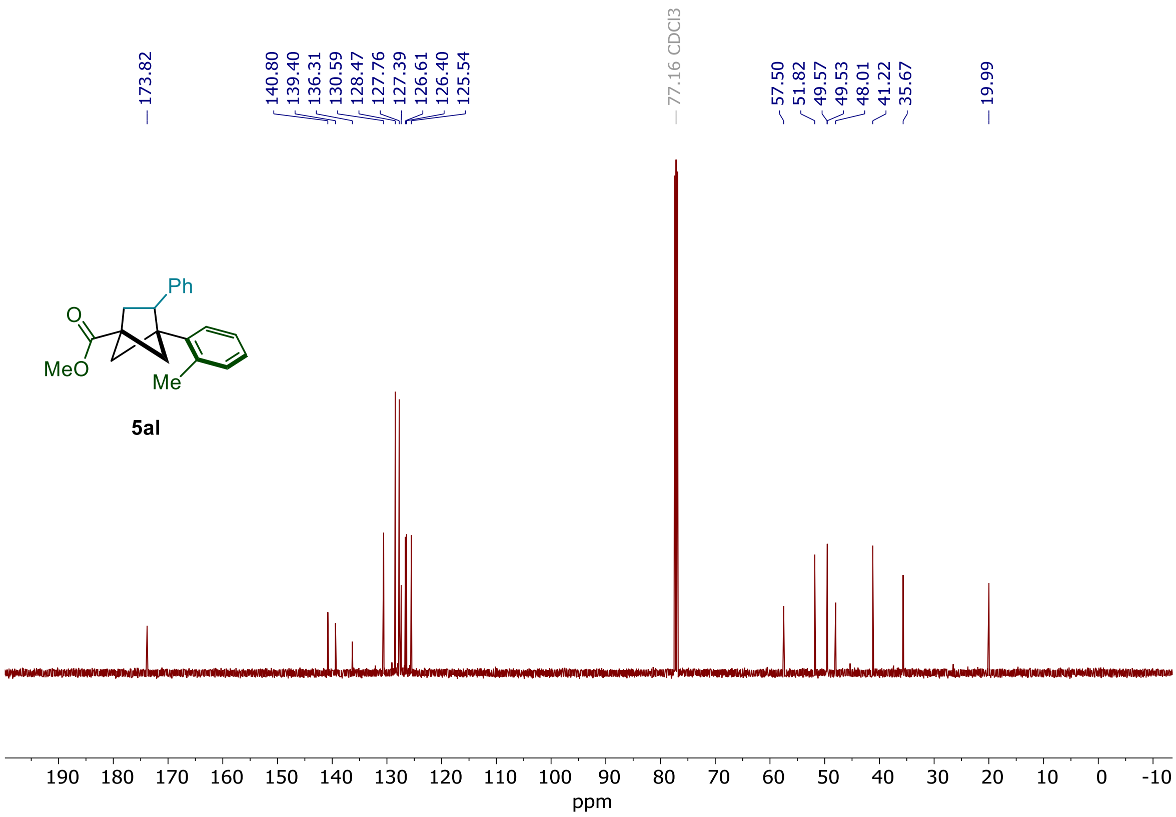

^{1}H NMR (400 MHz, $CDCl_3$) of **5am** (*see procedure*)

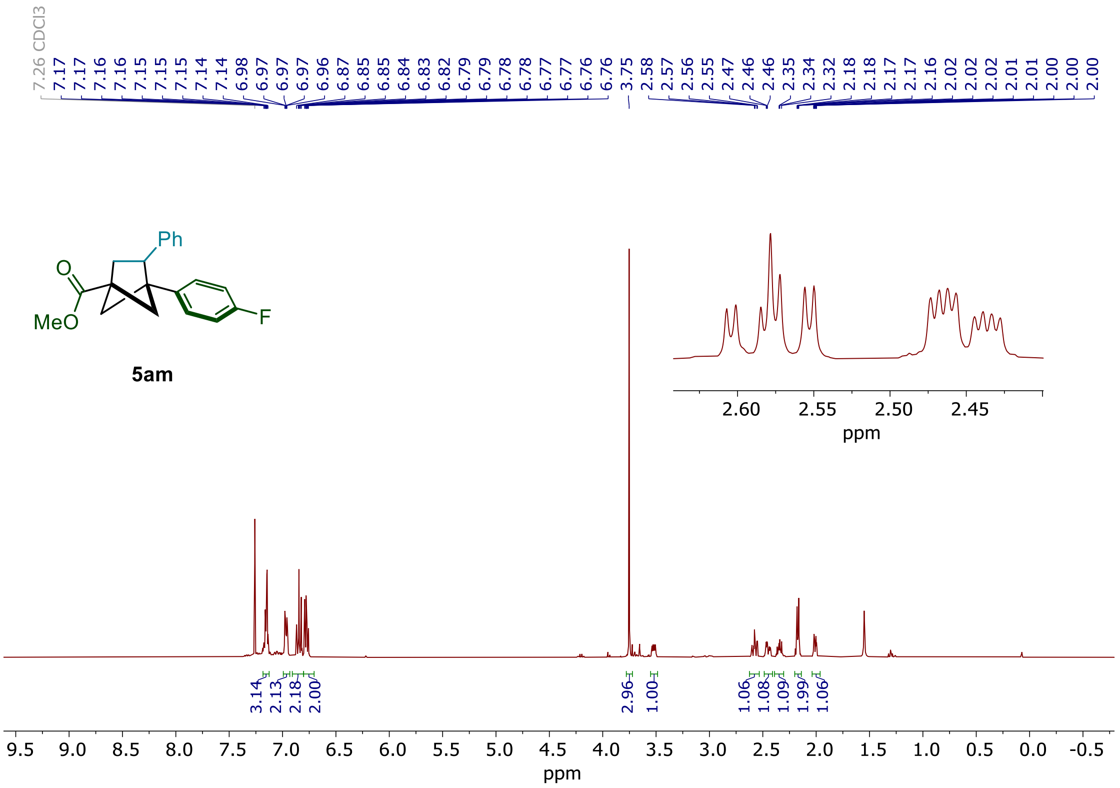

^{13}C NMR (126 MHz, $CDCl_3$) of **5am**

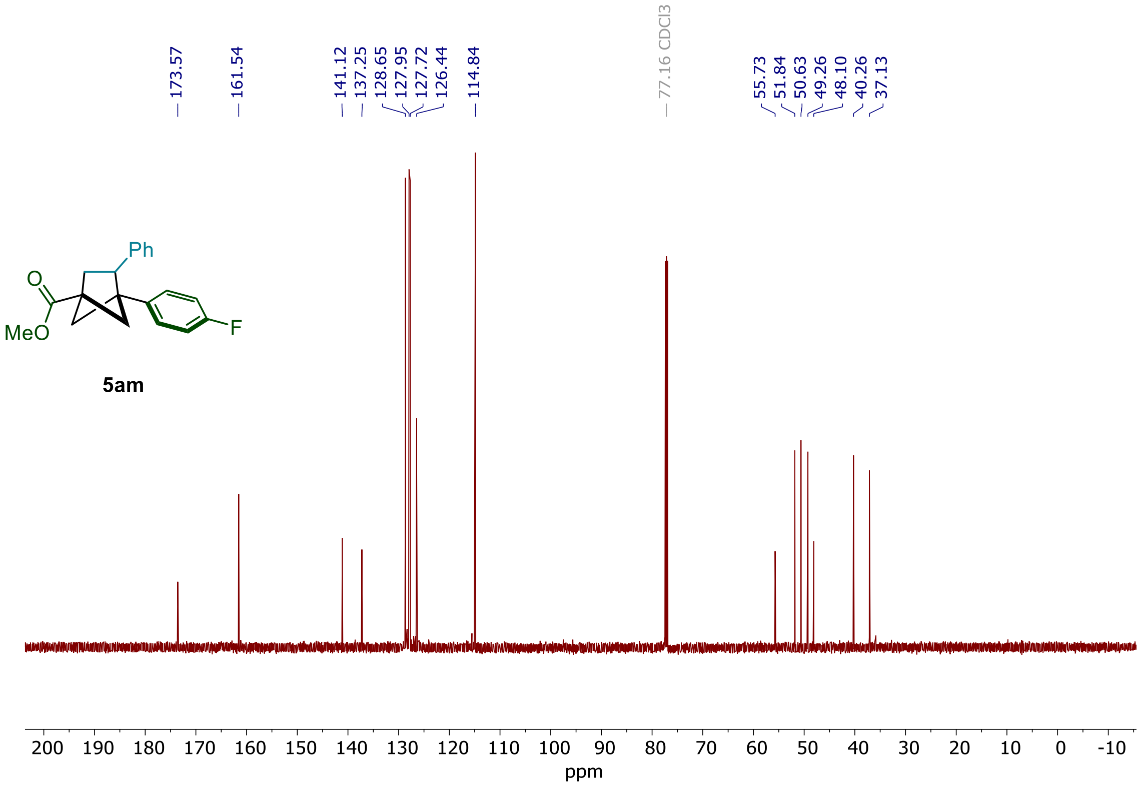

^{19}F NMR (376 MHz, $CDCl_3$) of **5am**

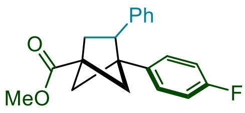

5am

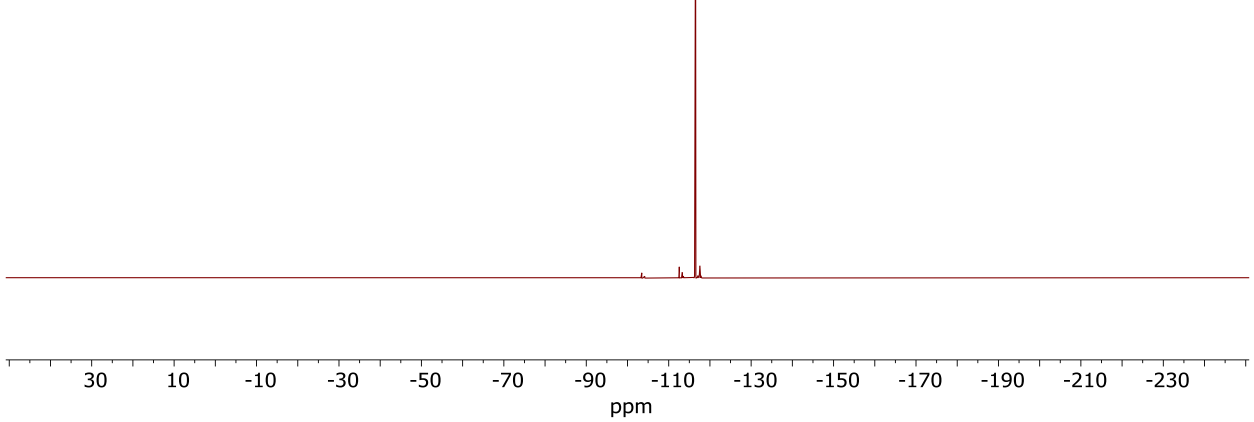

^{1}H NMR (600 MHz, $CDCl_3$) of **5ap** (*see procedure*)

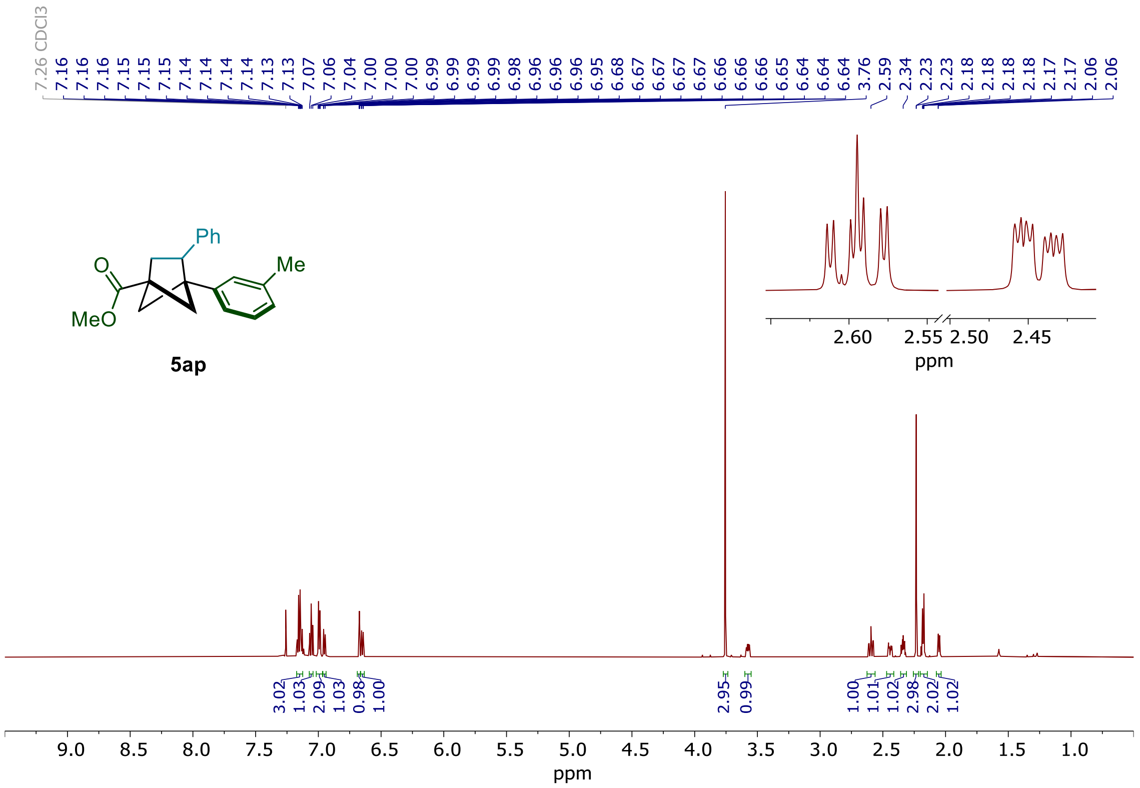

^{13}C NMR (151 MHz, $CDCl_3$) of **5ap**

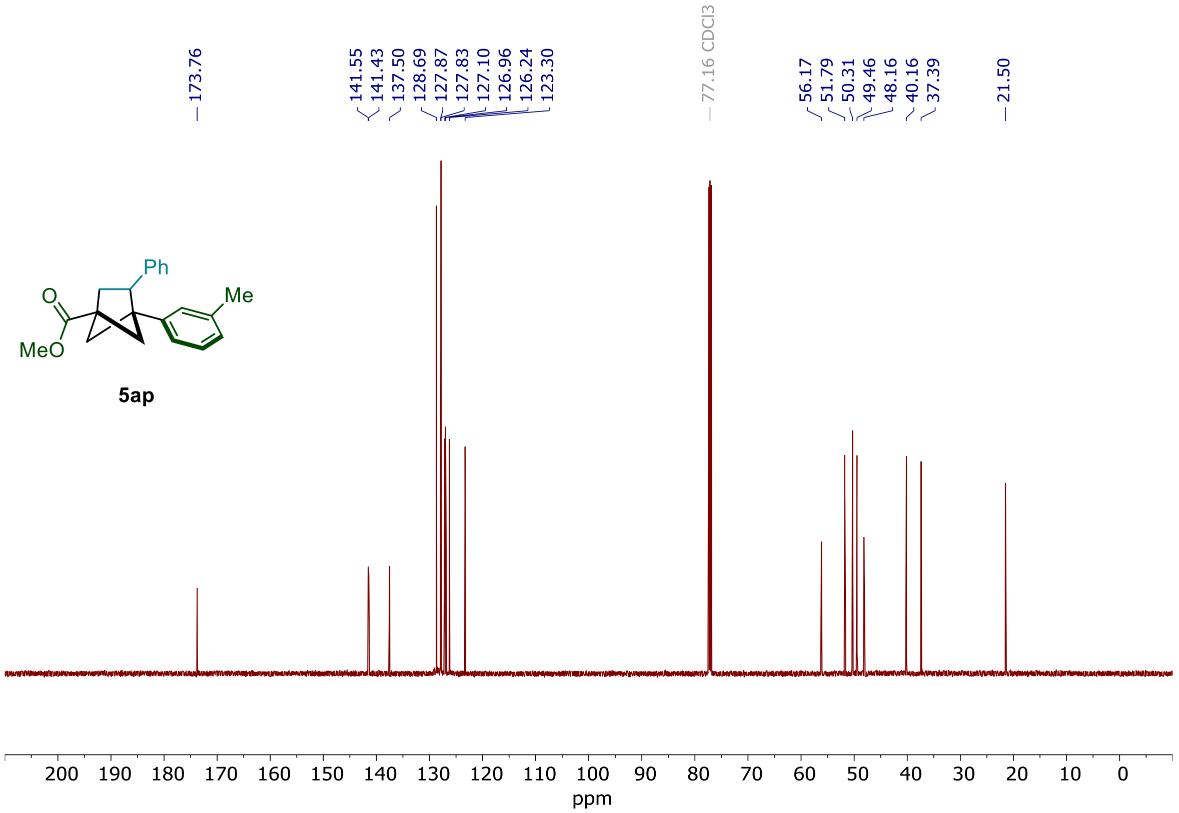

^{1}H NMR (600 MHz, $CDCl_3$) of **5aq** (*see procedure*)

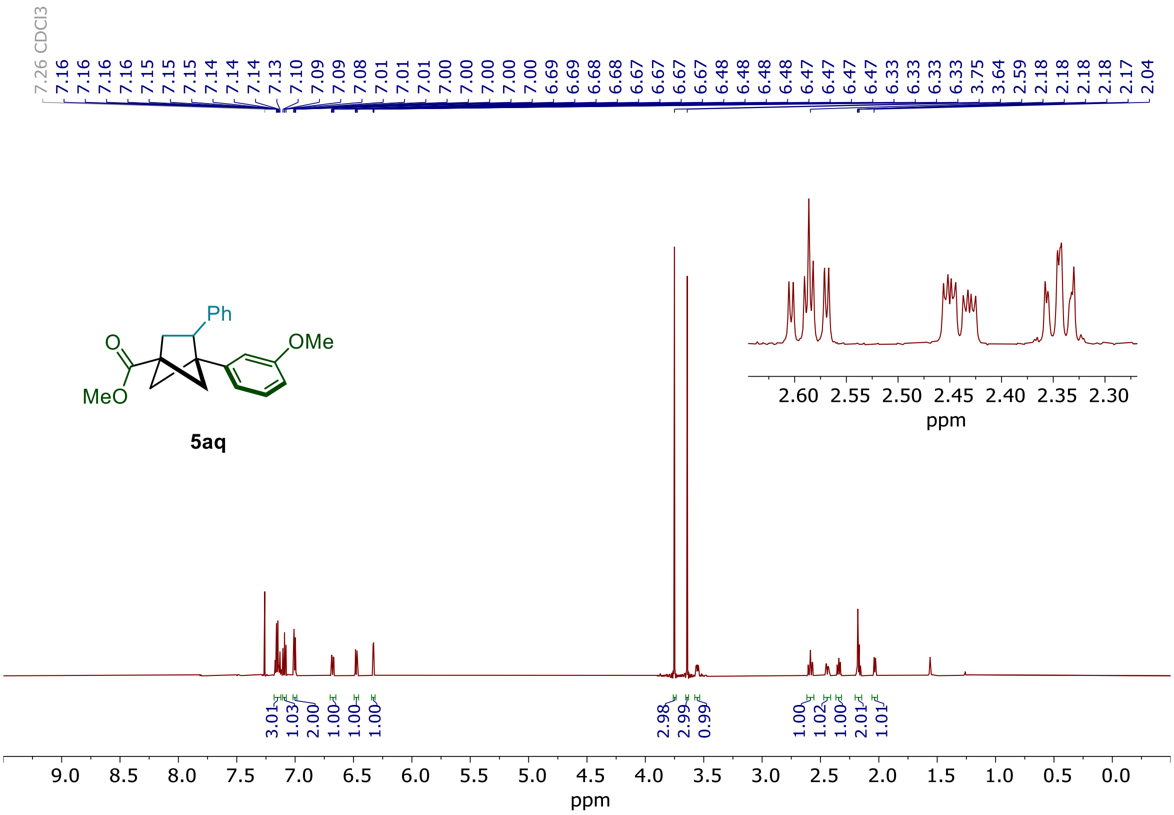

^{13}C NMR (151 MHz, $CDCl_3$) of **5aq**

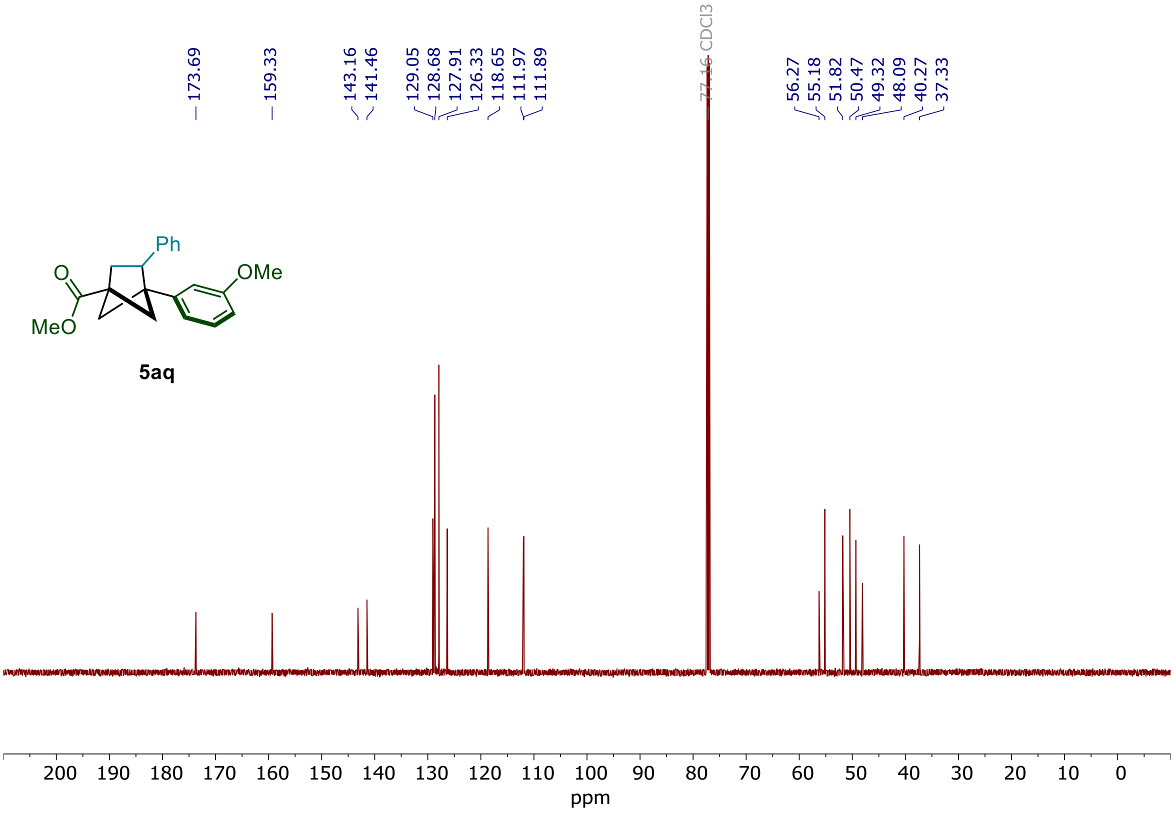

^{1}H NMR (400 MHz, $CDCl_3$) of **5ar** (*see procedure*)

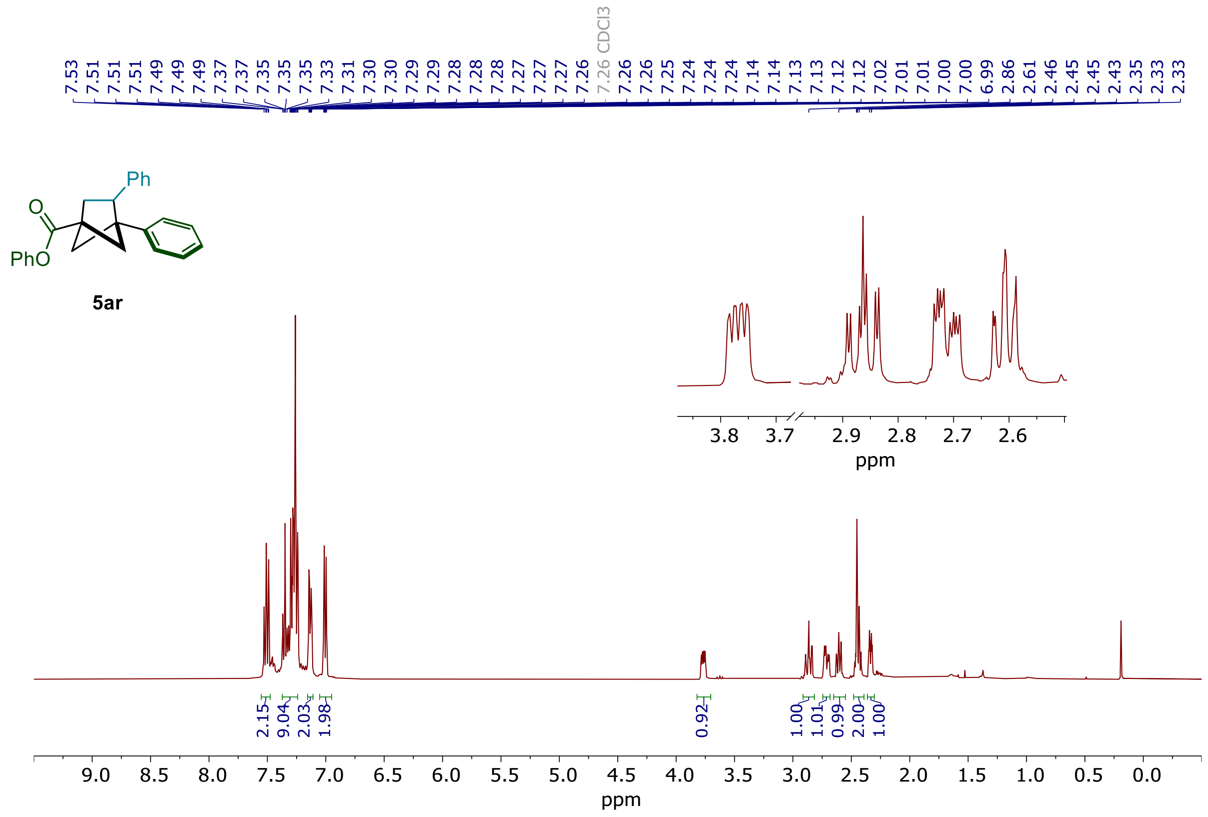

^{13}C NMR (101 MHz, $CDCl_3$) of **5ar**

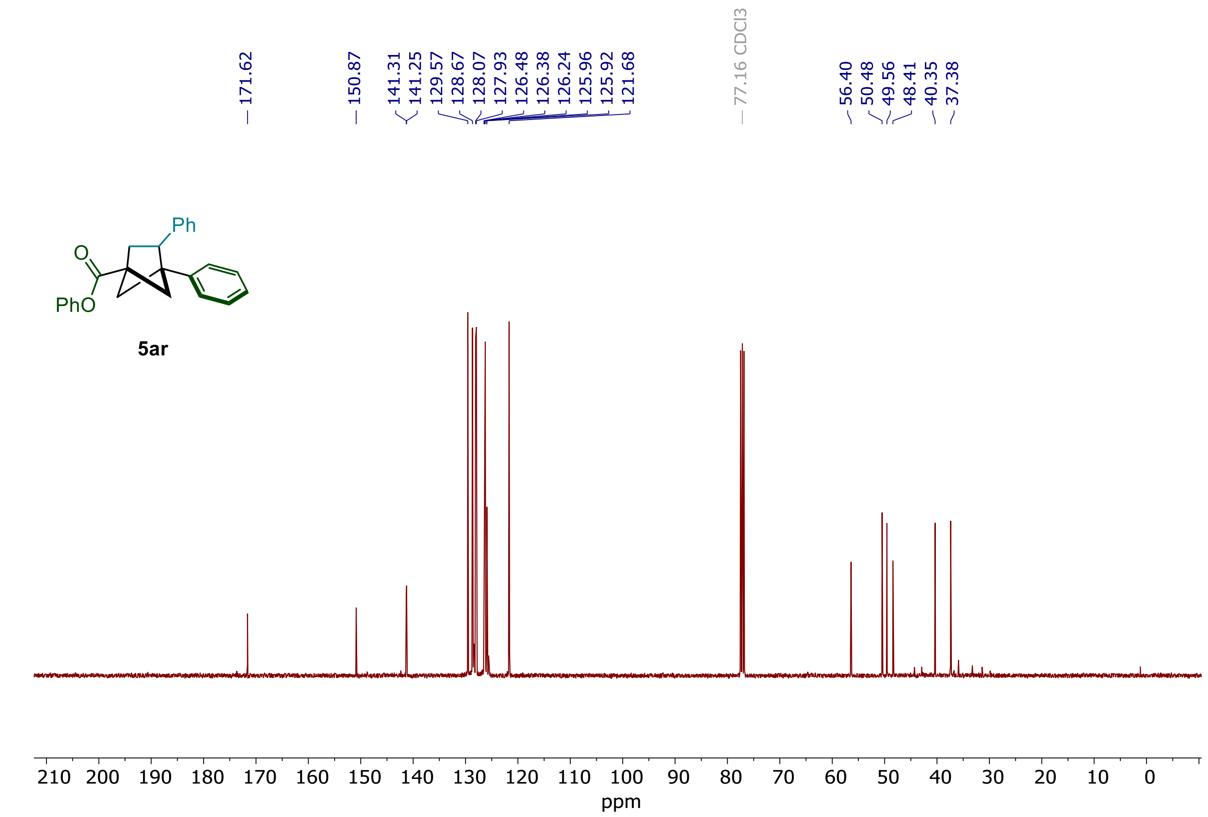

^{1}H NMR (400 MHz, $CDCl_3$) of **5as** (*see procedure*)

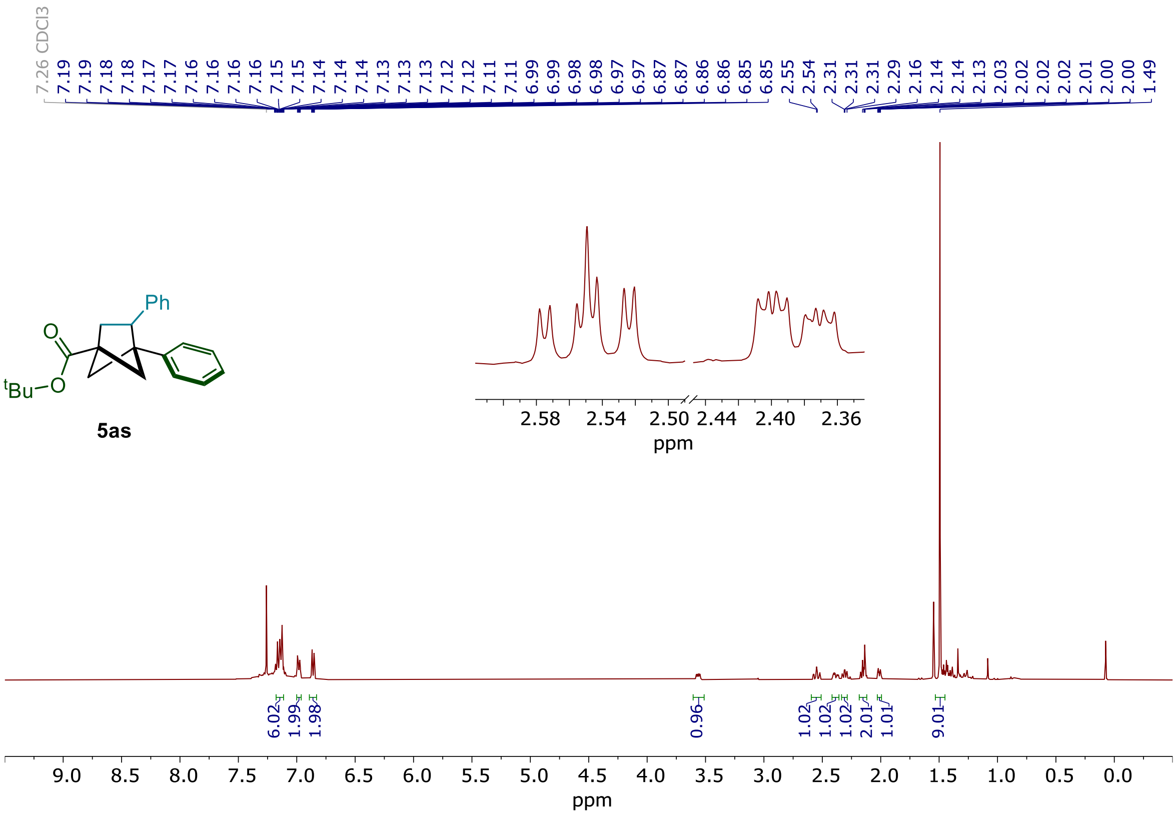

^{13}C NMR (101 MHz, $CDCl_3$) of **5as**

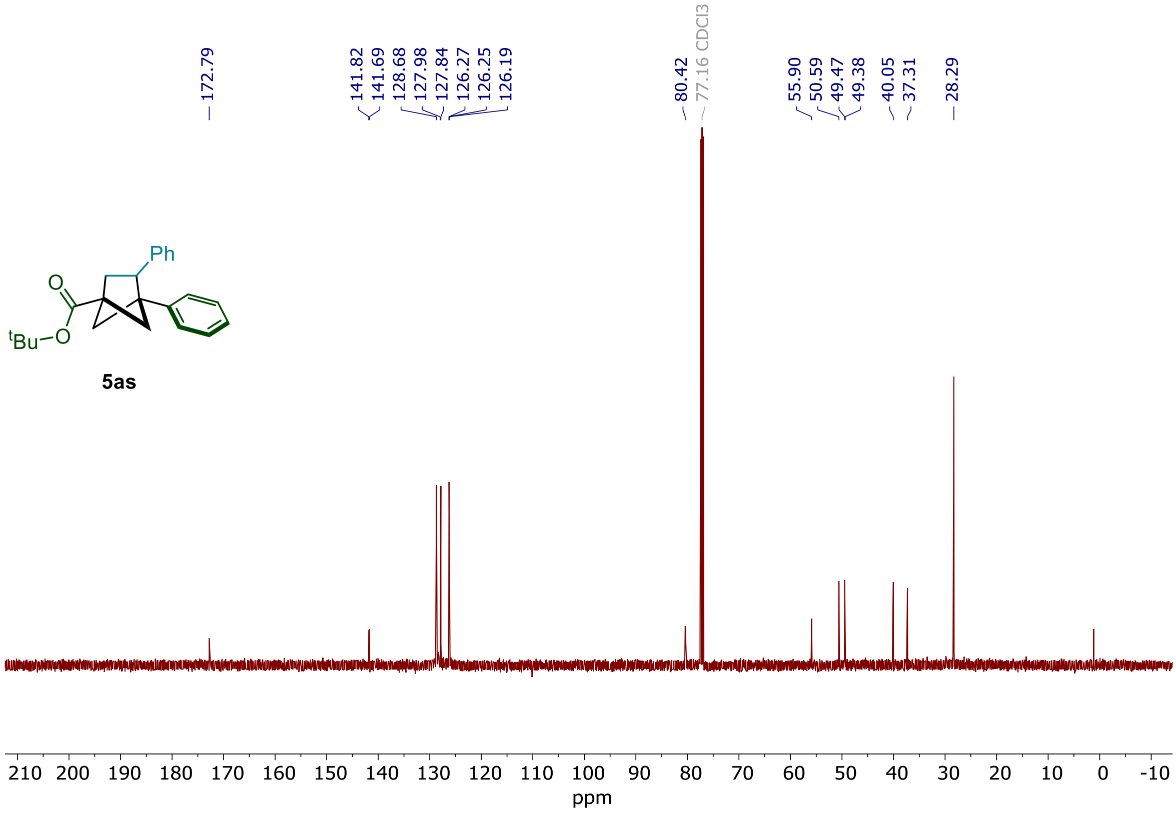

^{1}H NMR (600 MHz, $CDCl_3$) of **5at** (*see procedure*)

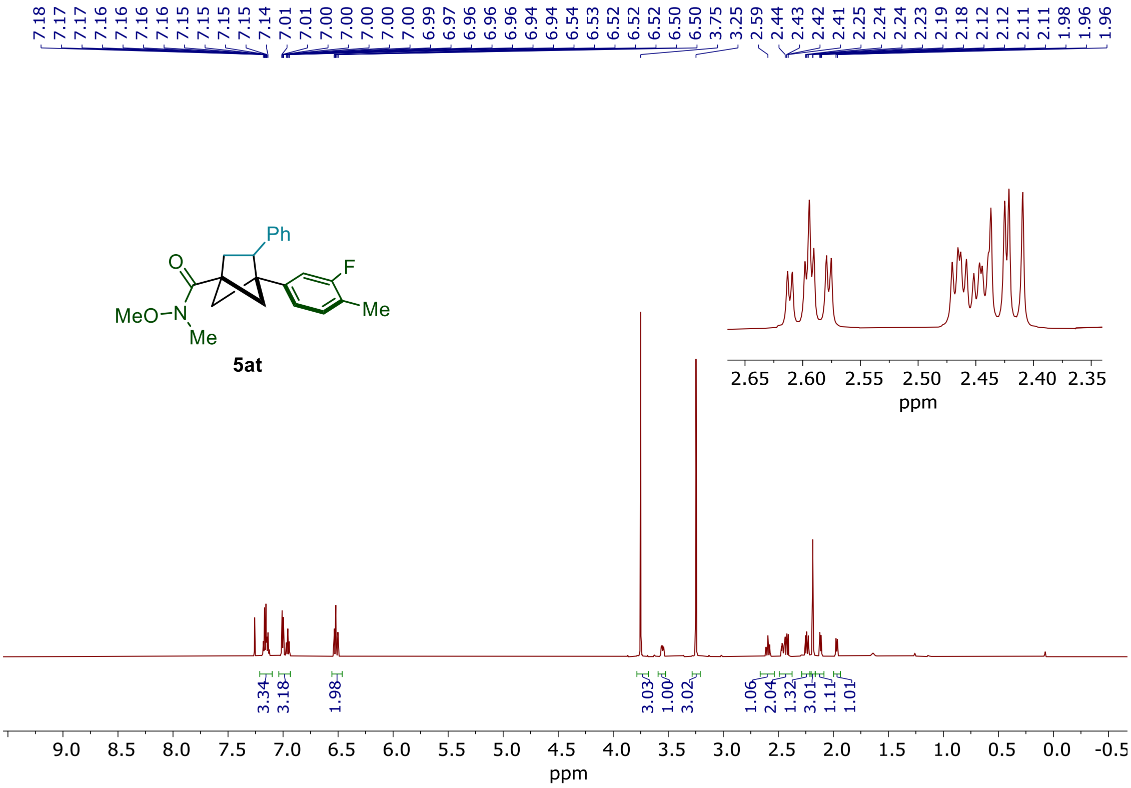

^{13}C NMR (126 MHz, $CDCl_3$) of **5at**

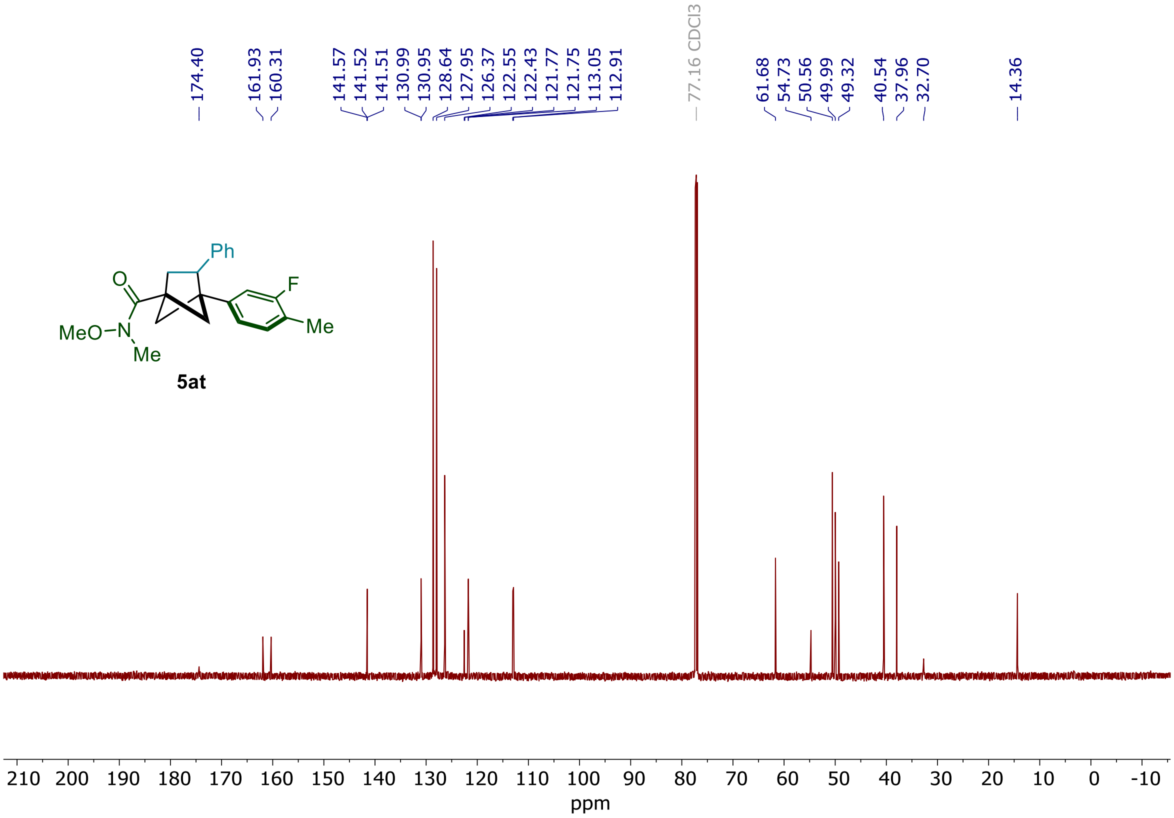

^{19}F NMR (376 MHz, $CDCl_3$) of **5at**

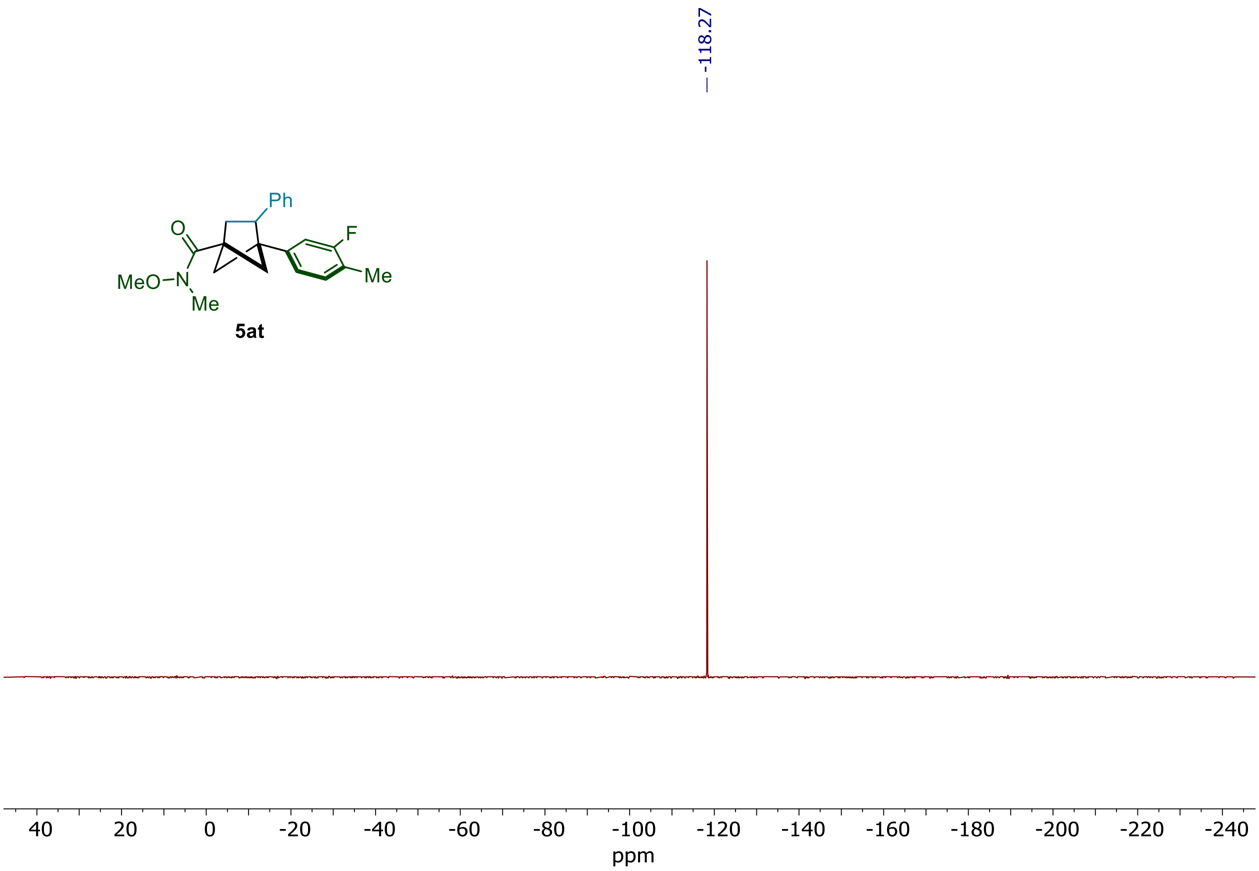

5at

^{1}H NMR (400 MHz, $CDCl_3$) of **5au** (*see procedure*)

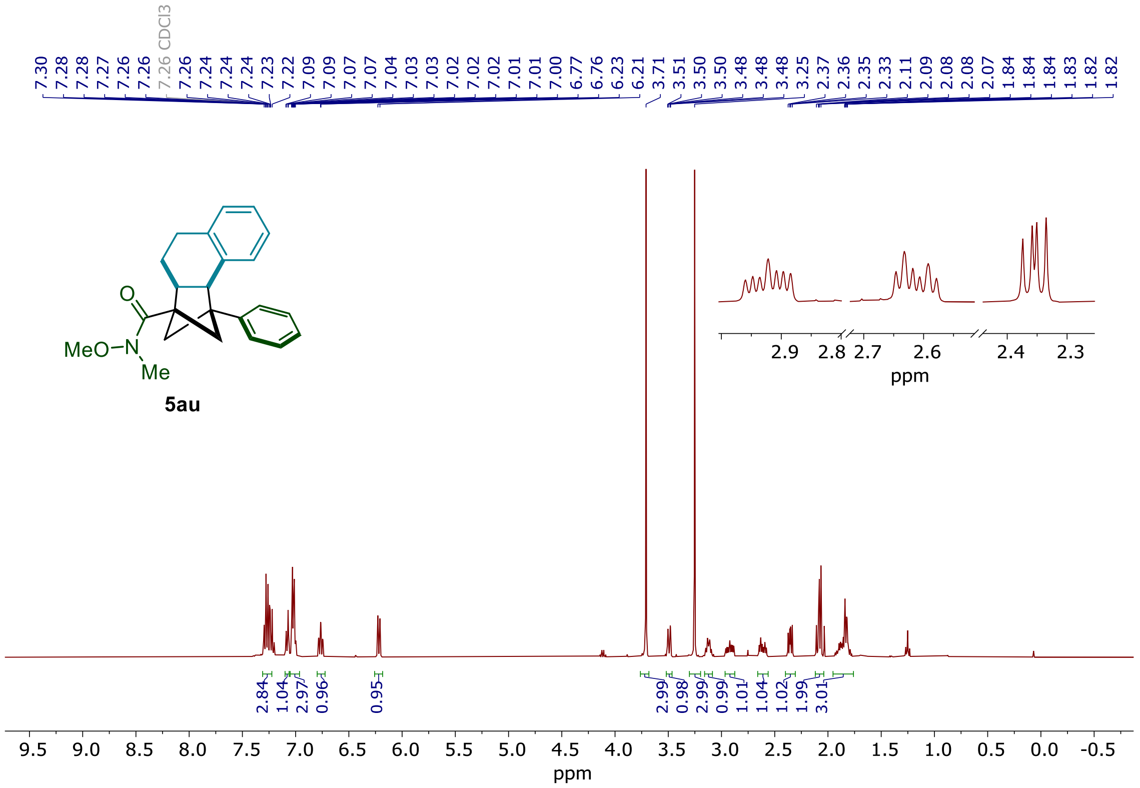

^{13}C NMR (101 MHz, $CDCl_3$) of **5au**

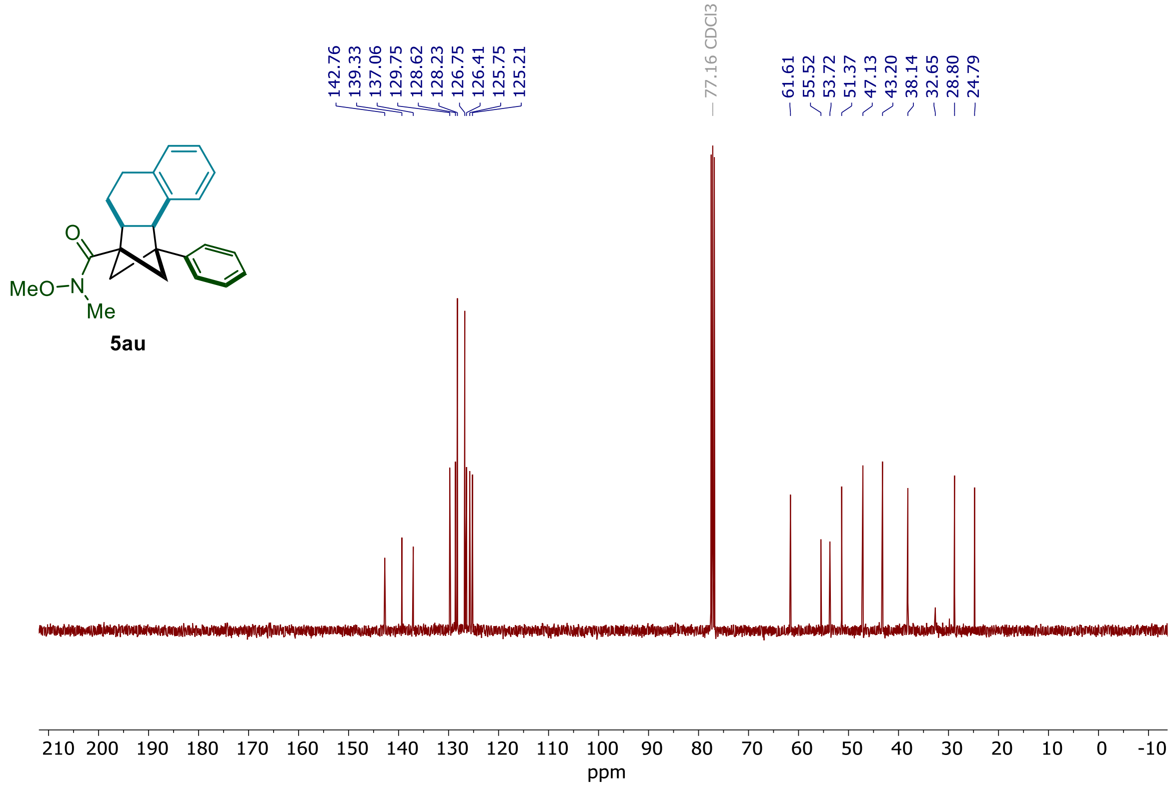

^{1}H NMR (500 MHz, $CDCl_3$) of **5av** (*see procedure*)

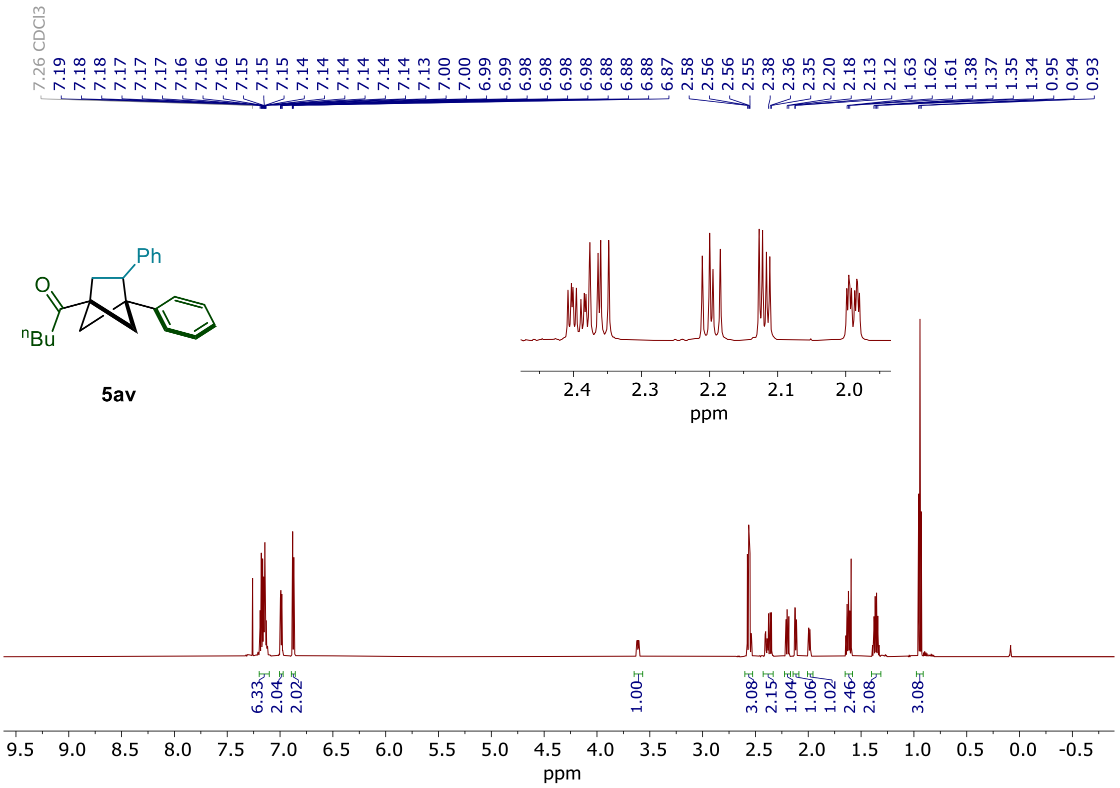

^{13}C NMR (126 MHz, $CDCl_3$) of **5av**

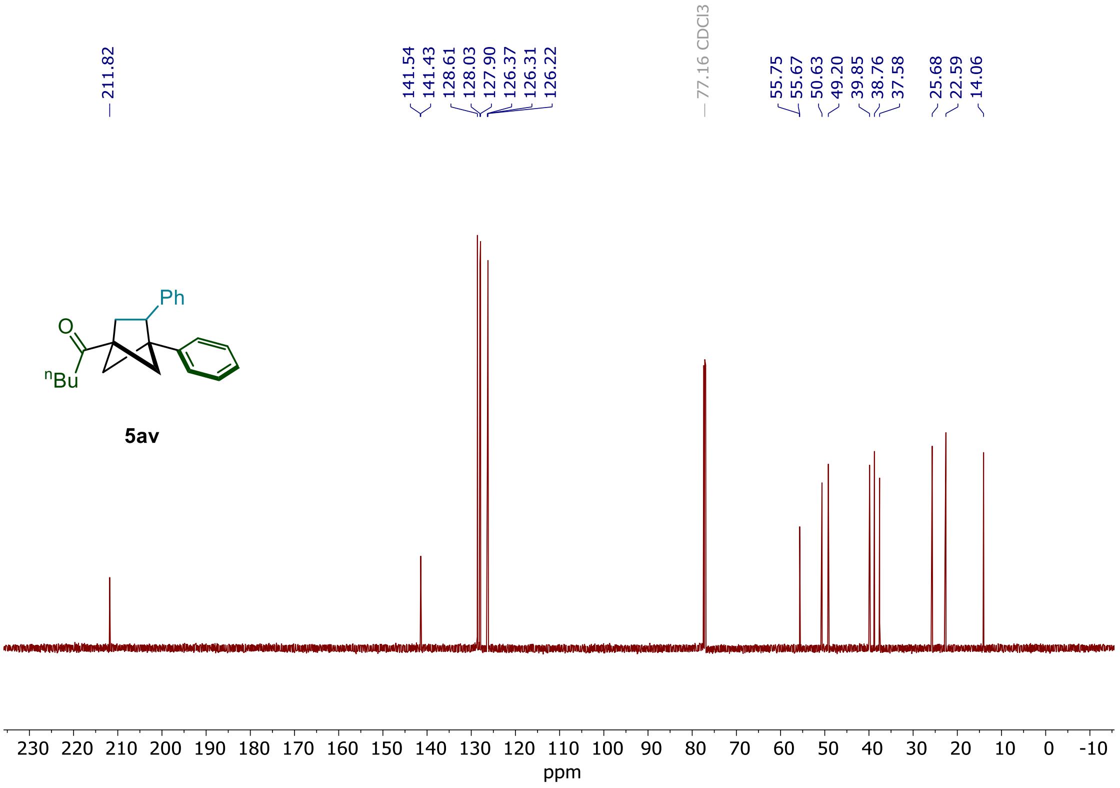

^{1}H NMR (500 MHz, $CDCl_3$) of **5aw** (*see procedure*)

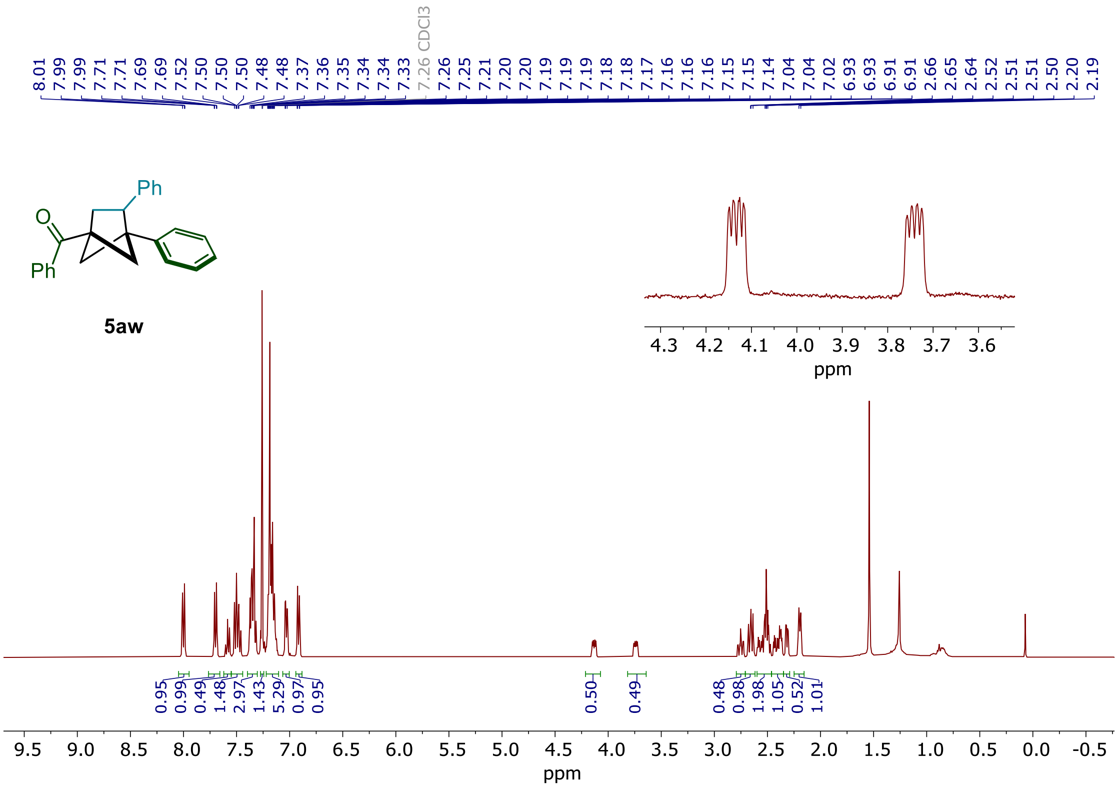

^{13}C NMR (126 MHz, $CDCl_3$) of **5aw**

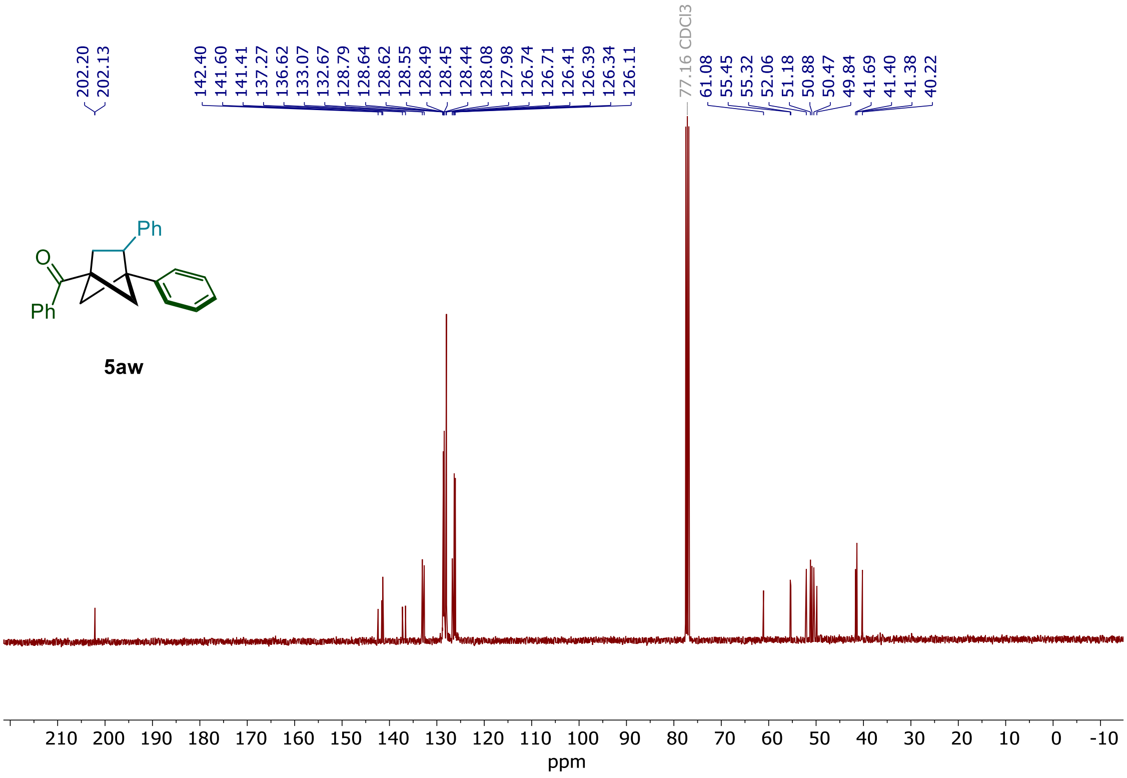

^{1}H NMR (400 MHz, $CDCl_3$) of **5ax** (see procedure)

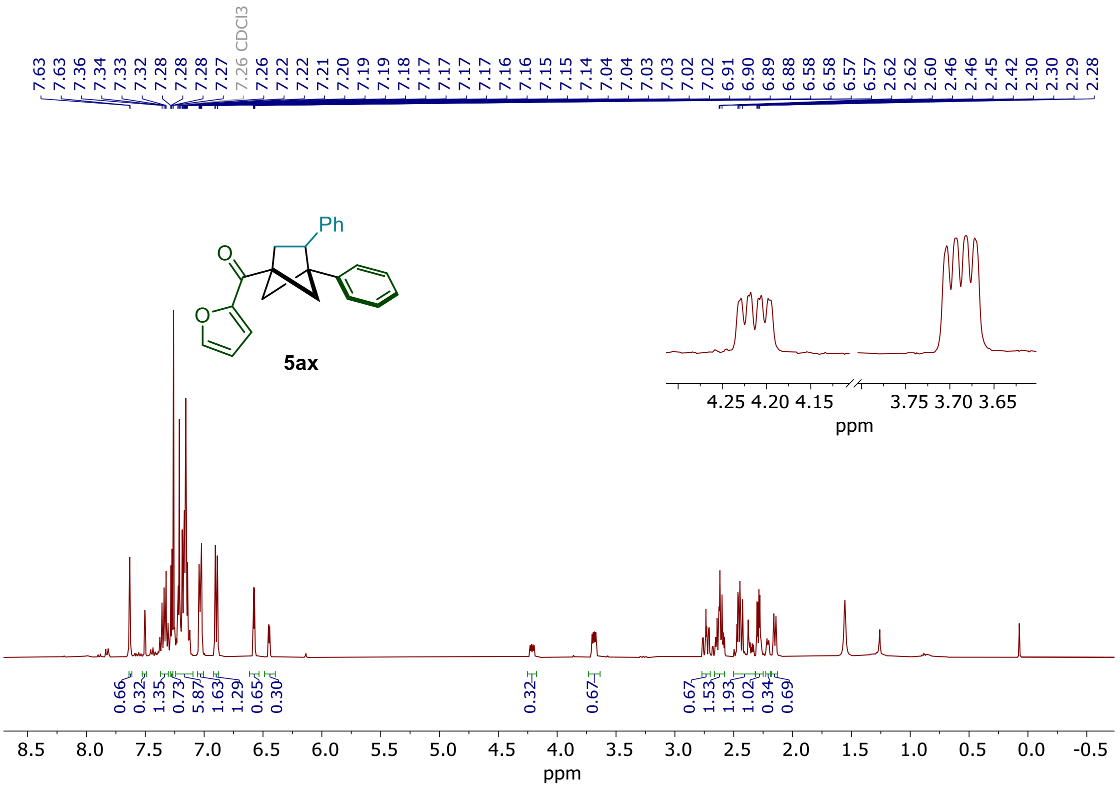

^{13}C NMR (126 MHz, $CDCl_3$) of **5ax**

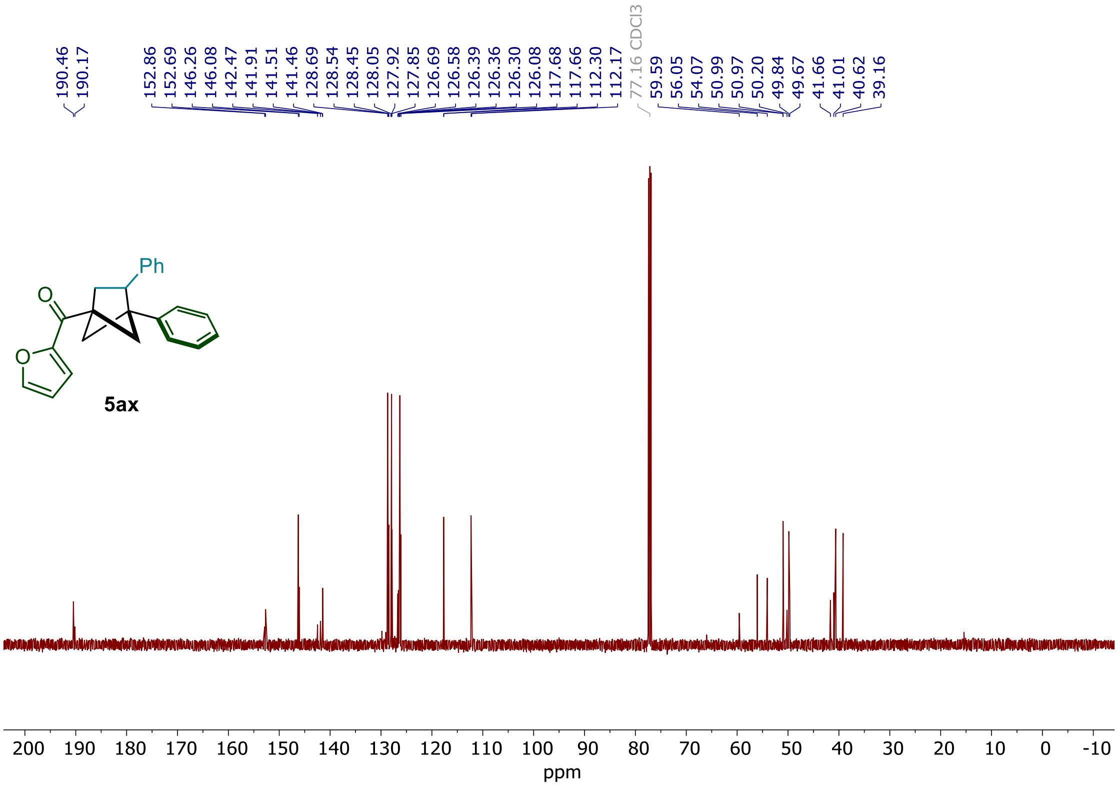

^{1}H NMR (500 MHz, $CDCl_3$) of **5ay** (see procedure)

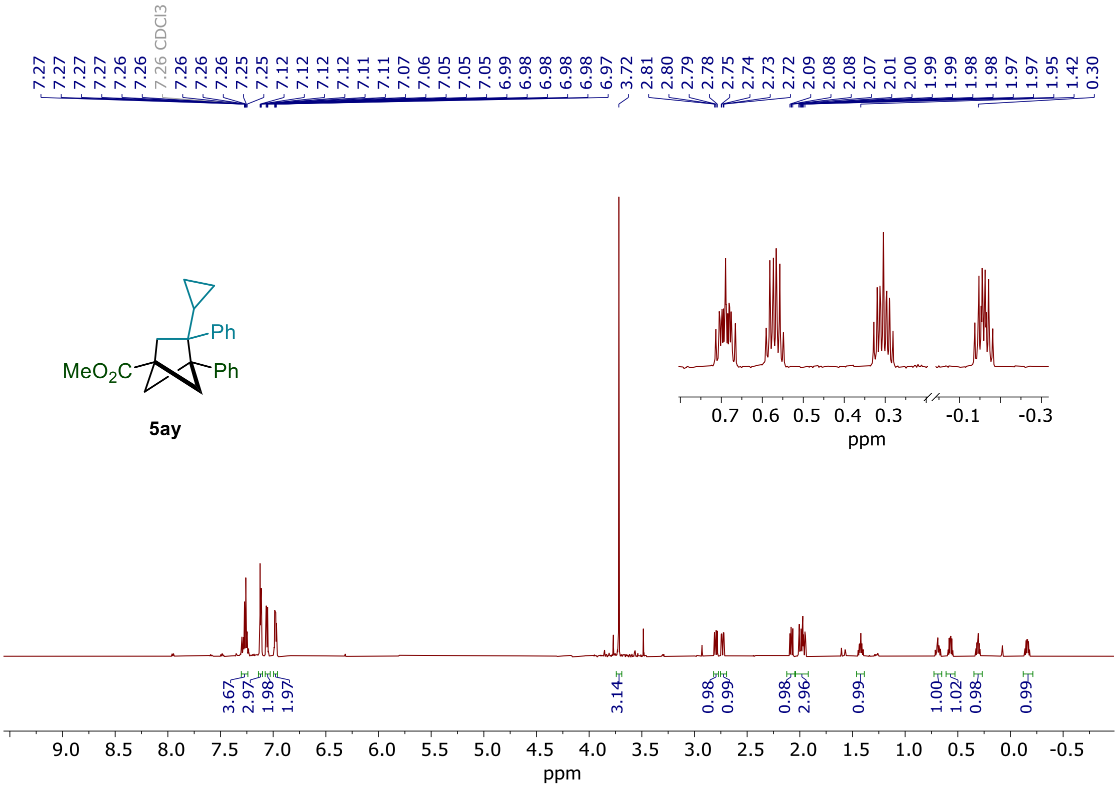

^{13}C NMR (126 MHz, $CDCl_3$) of **5ay**

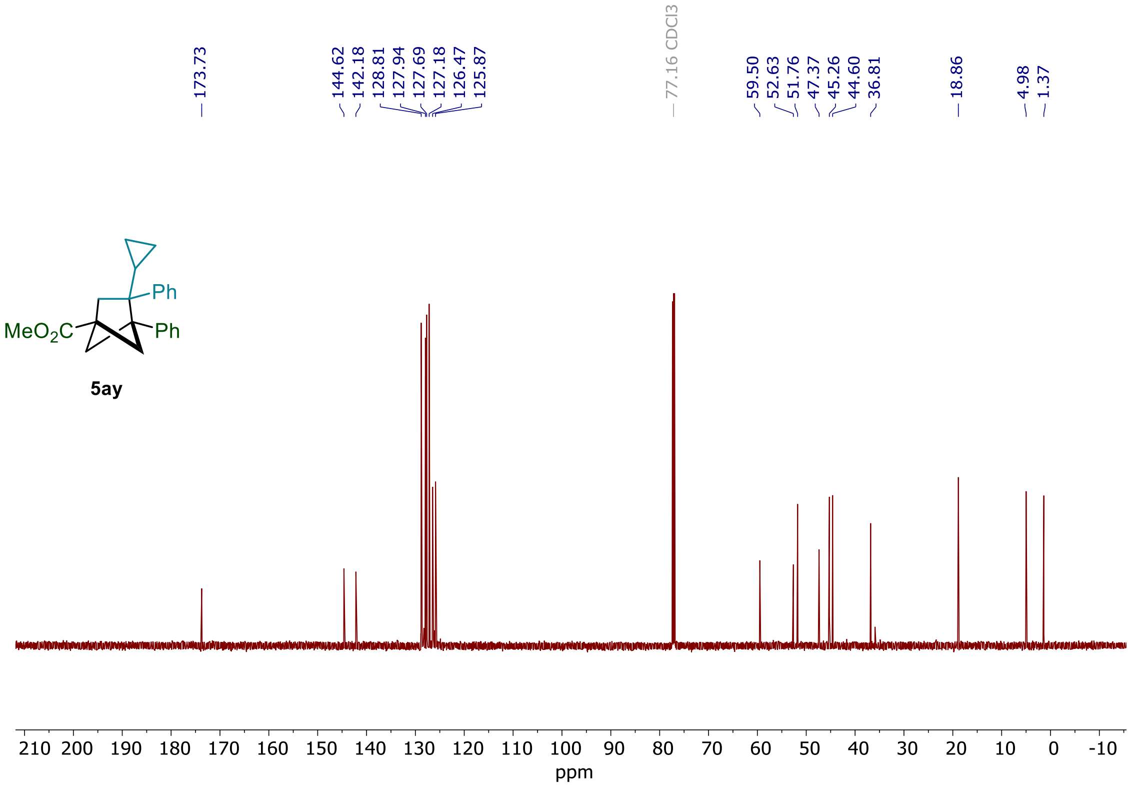

^{1}H NMR (600 MHz, $CDCl_3$) of **9** (*see procedure*)

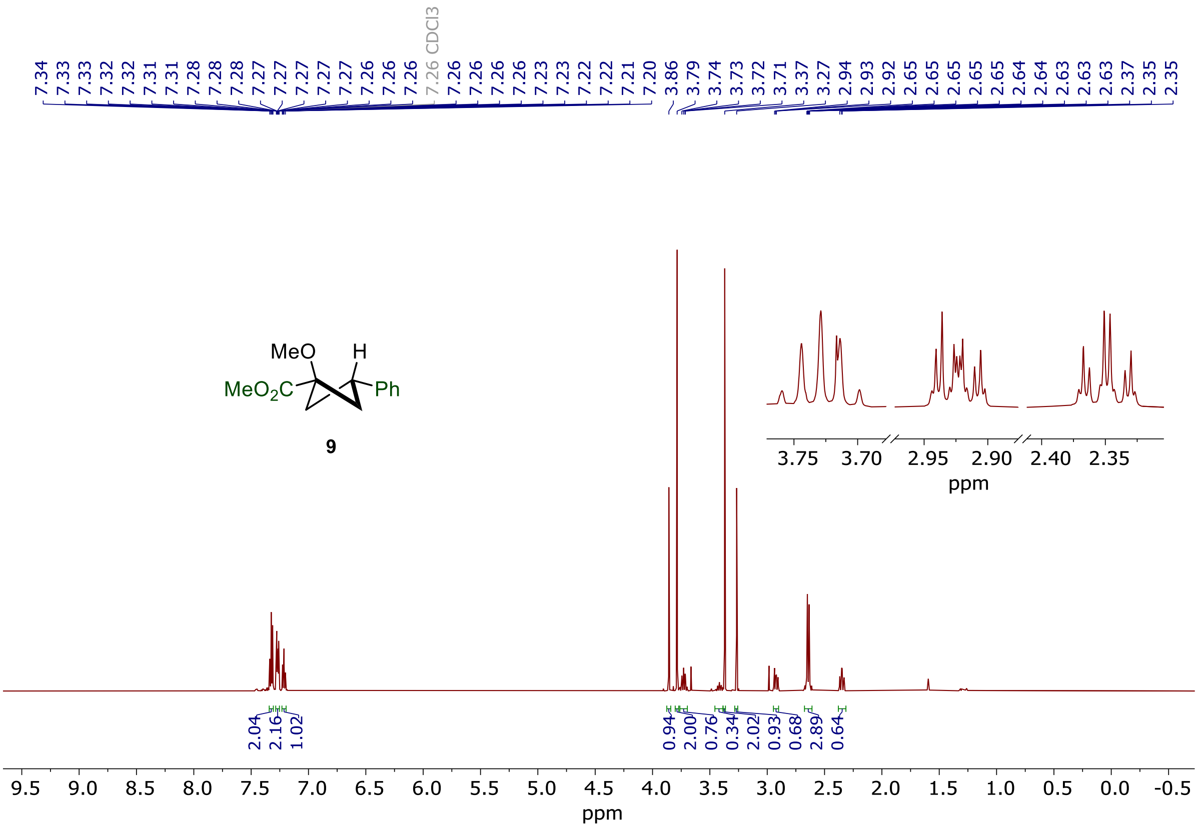

^{13}C NMR (151 MHz, $CDCl_3$) of **9**

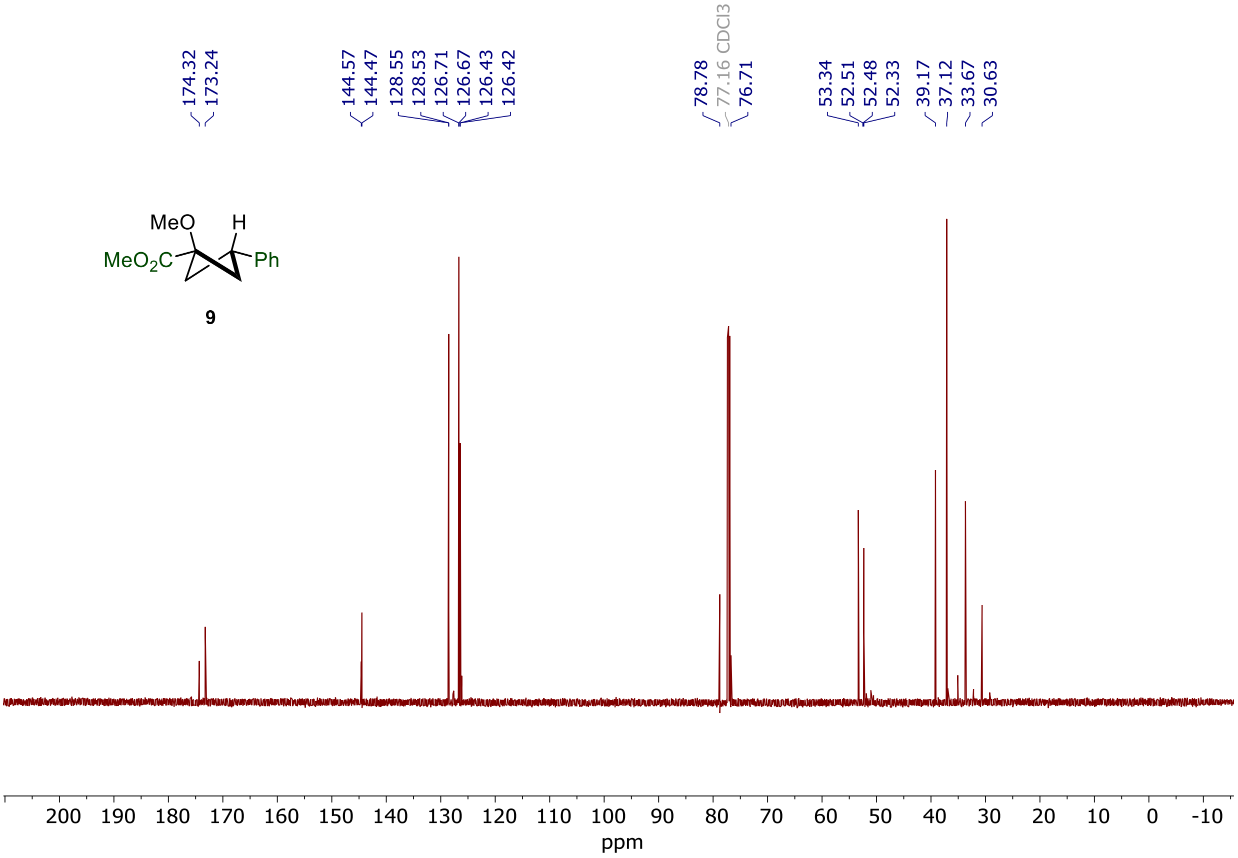

7. REFERENCES

(1) Kalyuzhny, G.; Buda, M.; McNeill, J.; Barbara, P.; Bard, A. J. Stability of thin-film solid-state electroluminescent devices based on tris(2,2'-bipyridine)ruthenium(II) complexes. *J. Am. Chem. Soc.* **2003**, *125*, 6272–6283.

(2) Lowry, M. S.; Goldsmith, J. I.; Slinker, J. D.; Rohl, R.; Pascal, R. A.; Malliaras, G. G.; Bernhard, S. Single-layer electroluminescent devices and photoinduced hydrogen production from an ionic Iridium(III) complex. *Chem. Mater.* **2005**, *17*, 5712–5719.

(3) Luo, J.; Zhang, J. Donor–acceptor fluorophores for visible-light-promoted organic synthesis: Photoredox/Ni dual catalytic C(sp 3)–C(sp 2) cross-coupling. *ACS Catal.* **2016**, *6*, 873–877.

(4) Tanaka, K.; Kishimoto, M.; Sukekawa, M.; Hoshino, Y.; Honda, K. Green-light-driven thioxanthylium-based organophotoredox catalysts: Organophotoredox promoted radical cation Diels-Alder reaction. *Tetrahedron Lett.* **2018**, *59*, 3361–3364.

(5) Pitzer, L.; Sandfort, F.; Strieth-Kalthoff, F.; Glorius, F. Carbonyl-olefin cross-metathesis through a visible-light-induced 1,3-diol formation and fragmentation sequence. *Angew. Chem. Int. Ed.* **2018**, *57*, 16219–16223.

(6) Wang, H.; Bellotti, P.; Zhang, X.; Paulisch, T. O.; Glorius, F. A base-controlled switch of SO2 reincorporation in photocatalyzed radical difunctionalization of alkenes. *Chem* **2021**, *7*, 3412–3424.

(7) Quach, L.; Dutta, S.; Pflüger, P. M.; Sandfort, F.; Bellotti, P.; Glorius, F. Visible-light-initiated hydrooxygenation of unactivated alkenes—A strategy for anti-Markovnikov Hydrofunctionalization. *ACS Catal.* **2022**, *12*, 2499–2504.

(8) Erchinger, J. E.; Hoogesteger, R.; Laskar, R.; Dutta, S.; Hümpel, C.; Rana, D.; Daniliuc, C. G.; Glorius, F. EnT-mediated N-S bond homolysis of a bifunctional reagent leading to aliphatic sulfonyl fluorides. *J. Am. Chem. Soc.* **2023**, *145*, 2364–2374.

(9) Tan, G.; Das, M.; Keum, H.; Bellotti, P.; Daniliuc, C.; Glorius, F. Photochemical single-step synthesis of β-amino acid derivatives from alkenes and (hetero)arenes. *Nat. Chem.* **2022**, *14*, 1174–1184.

(10) Brice, J. L.; Meerdink, J. E.; Stahl, S. S. Formation of enamides via palladium(II)-catalyzed vinyl transfer from vinyl ethers to nitrogen nucleophiles. *Org. Lett.* **2004**, *6*, 1845–1848.

(11) Bellotti, P.; Huang, H.-M.; Faber, T.; Laskar, R.; Glorius, F. Catalytic defluorinative ketyl-olefin coupling by halogen-atom transfer. *Chem. Sci.* **2022**, *13*, 7855–7862.

(12) Ming, X.-X.; Wu, S.; Tian, Z.-Y.; Song, J.-W.; Zhang, C.-P. Pd/Cu-catalyzed vinylation of terminal alkynes with (2-bromoethyl)diphenylsulfonium triflate. *Org. Lett.* **2021**, *23*, 6795–6800.

(13) Wang, Y.; Zhang, W.; Colandrea, V. J.; Jimenez, L. S. Reactivity and rearrangements of dialkyl- and diarylvinylsulfonium salts with indole-2- and pyrrole-2-carboxaldehydes. *Tetrahedron* **1999**, *55*, 10659–10672.

(14) Dutta, S.; Lee, D.; Ozols, K.; Daniliuc, C. G.; Shintani, R.; Glorius, F. Photoredox-enabled dearomative [2π + 2σ] cycloaddition of phenols. *J. Am. Chem. Soc.* **2024**, *146*, 2789–2797.

(15) Lin, S.-L.; Chen, Y.-H.; Liu, H.-H.; Xiang, S.-H.; Tan, B. Enantioselective synthesis of chiral cyclobutenes enabled by Brønsted acid-catalyzed isomerization of BCBs. *J. Am. Chem. Soc.* **2023**, *145*, 21152–21158.

(16) Nguyen, T. V. T.; Bossonnet, A.; Wodrich, M. D.; Waser, J. Photocatalyzed [2σ + 2σ] and [2σ + 2π] cycloadditions for the synthesis of bicyclo[3.1.1]heptanes and 5- or 6-membered carbocycles. *J. Am. Chem. Soc.* **2023**, *145*, 25411–25421.

(17) Kleinmans, R.; Pinkert, T.; Dutta, S.; Paulisch, T. O.; Keum, H.; Daniliuc, C. G.; Glorius, F.

Intermolecular [2π+2σ]-photocycloaddition enabled by triplet energy transfer. *Nature* **2022**, *605*, 477–482.
(18) Bychek, R.; Mykhailiuk, P. K. A practical and scalable approach to fluoro-substituted bicyclo[1.1.1]pentanes. *Angew. Chem. Int. Ed.* **2022**, *61*, e202205103.
(19) Pitzer, L.; Schäfers, F.; Glorius, F. Rapid assessment of the reaction-condition-based sensitivity of chemical transformations. *Angew. Chem. Int. Ed.* **2019**, *58*, 8572–8576.
(20) Cismesia, M. A.; Yoon, T. P. Characterizing chain processes in visible light photoredox catalysis. *Chem. Sci.* **2015**, *6*, 5426–5434.
(21) Hatchard, C. G.; Parker.C. A. A new sensitive chemical actinometer - II. Potassium ferrioxalate as a standard chemical actinometer. *Proc. R. Soc. Lond. A Math. Phys. Sci.* **1956**, *235*, 518–536.
(22) Pozdnyakov, I. P.; Kel, O. V.; Plyusnin, V. F.; Grivin, V. P.; Bazhin, N. M. Reply to “Comment on ‘New insight into photochemistry of ferrioxalate’”. *J. Phys. Chem. A* **2009**, *113*, 8820–8822.
(23) Wegner, E. E.; Adamson, A. W. Photochemistry of complex ions. III. Absolute quantum yields for the photolysis of some aqueous Chromium(III) complexes. Chemical actinometry in the long wavelength visible region. *J. Am. Chem. Soc.* **1966**, *88*, 394–404.
(24) Chai, J.-D.; Head-Gordon, M. Long-range corrected hybrid density functionals with damped atom-atom dispersion corrections. *Phys. Chem. Chem. Phys.* **2008**, *10*, 6615–6620.
(25) Weigend, F.; Ahlrichs, R. Balanced basis sets of split valence, triple zeta valence and quadruple zeta valence quality for H to Rn: Design and assessment of accuracy. *Phys. Chem. Chem. Phys.* **2005**, *7*, 3297–3305.
(26) Marenich, A. V.; Cramer, C. J.; Truhlar, D. G. Universal solvation model based on solute electron density and on a continuum model of the solvent defined by the bulk dielectric constant and atomic surface tensions. *J. Phys. Chem. B* **2009**, *113*, 6378–6396.
(27) Grimme, S. Supramolecular binding thermodynamics by dispersion-corrected density functional theory. *Chem. Eur. J* **2012**, *18*, 9955–9964.
(28) Luchini, G.; Alegre-Requena, J. V.; Funes-Ardoiz, I.; Paton, R. S. GoodVibes: automated thermochemistry for heterogeneous computational chemistry data. *F1000Res* **2020**, *9*, 291.
(29) Frisch, M. J.; Trucks, G. W.; Schlegel, H. B.; Scuseria, G. E.; Robb, M. A.; Cheeseman, J. R.; Scalmani, G.; Barone, V.; Petersson, G. A.; Nakatsuji, H.; Li, X.; Caricato, M.; Marenich, A. V.; Bloino, J.; Janesko, B. G.; Gomperts, R.; Mennucci, B.; Hratchian, H. P.; Ortiz, J. V.; Izmaylov, A. F.; Sonnenberg, J. L.; Williams; Ding, F.; Lipparini, F.; Egidi, F.; Goings, J.; Peng, B.; Petrone, A.; Henderson, T.; Ranasinghe, D.; Zakrzewski, V. G.; Gao, J.; Rega, N.; Zheng, G.; Liang, W.; Hada, M.; Ehara, M.; Toyota, K.; Fukuda, R.; Hasegawa, J.; Ishida, M.; Nakajima, T.; Honda, Y.; Kitao, O.; Nakai, H.; Vreven, T.; Throssell, K.; Montgomery Jr., J. A.; Peralta, J. E.; Ogliaro, F.; Bearpark, M. J.; Heyd, J. J.; Brothers, E. N.; Kudin, K. N.; Staroverov, V. N.; Keith, T. A.; Kobayashi, R.; Normand, J.; Raghavachari, K.; Rendell, A. P.; Burant, J. C.; Iyengar, S. S.; Tomasi, J.; Cossi, M.; Millam, J. M.; Klene, M.; Adamo, C.; Cammi, R.; Ochterski, J. W.; Martin, R. L.; Morokuma, K.; Farkas, O.; Foresman, J. B.; Fox, D. J. *Gaussian 16 Rev. C.01*, **2016**.
(30) Towns, J.; Cockerill, T.; Dahan, M.; Foster, I.; Gaither, K.; Grimshaw, A.; Hazlewood, V.; Lathrop, S.; Lifka, D.; Peterson, G. D.; Roskies, R.; Scott, J. R.; Wilkins-Diehr, N. XSEDE: Accelerating Scientific Discovery. *Comput. Sci. Eng.* **2014**, *16*, 62–74.
(31) Bruker AXS. APEX4 Version 2021.4-0, SAINT Version 8.40B and SADABS Bruker AXS area detector

scaling and absorption correction Version 2016/2; Bruker AXS Inc., **2021**.

(32) Sheldrick, G. M. SHELXT - integrated space-group and crystal-structure determination. *Acta Crystallogr. A: Found. Adv.* **2015**, *71*, 3–8.

(33) Sheldrick, G. M. Crystal structure refinement with SHELXL. *Acta Crystallogr. C: Struct. Chem.* **2015**, *71*, 3–8.

(34) Bruker AXS. XP – Interactive molecular graphics, Version 5.1; Bruker AXS Inc., **1998.**
